# A revision of the Dulcamaroid Clade of *Solanum* L. (Solanaceae)

**DOI:** 10.3897/phytokeys.22.4041

**Published:** 2013-05-10

**Authors:** Sandra Knapp

**Affiliations:** 1 Department of Life Sciences, The Natural History Museum, Cromwell Road, London SW7 5BD, United Kingdom

**Keywords:** Asia, classification, monograph, new species, Solanum, South America, taxonomy, vines, USA

## Abstract

The Dulcamaroid clade of *Solanum* contains 45 species of mostly vining or weakly scandent species, including the common circumboreal weed *Solanum dulcamara* L. The group comprises members of the previously recognised infrageneric groupings sect. *Andropedas* Rusby, sect. *Californisolanum* A. Child, sect. *Dulcamara* (Moench) Dumort., sect. *Holophylla* (G.Don) Walp., sect. *Jasminosolanum* (Bitter) Seithe, sect.*Lysiphellos* (Bitter) Seithe, subsect. *Nitidum* A.Child and sect. *Subdulcamara* Dunal. These infrageneric groups are not monophyletic as traditionally recognised, and the complex history of the classification of the dulcamaroid solanums is reviewed. Many of the species in the clade are quite variable morphologically; plants are shrubs, herbaceous vines or woody canopy lianas, and habits can vary between these states in a single locality. Variation in leaf shape and pubescence density and type is also extreme and has lead to the description of many minor morphological variants as distinct species. The flowers of members of the group are generally very showy, and several species (e.g., *Solanum crispum* Ruiz & Pav., *Solanum laxum* Spreng., *Solanum seaforthianum* Andrews) are popular ornamental plants that have occasionally escaped from cultivation and become naturalised. The clade is here divided into five morphologically and geographically delimited species groups to facilitate further study. One new species from southern Ecuador, *Solanum agnoston* S.Knapp **sp. nov.**, is described here. Full descriptions and synonymies (including designations of lectotypes or neotypes), preliminary conservation assessments, illustrations, distribution maps, and an extensive list of localities are provided for all species.

## Introduction

*Solanum* L. is one of the ten most species-rich genera of flowering plants (Frodin 2004). With approximately 1400 species (J. Bennett and S. Knapp, unpubl.) occurring on all temperate and tropical continents, the genus occupies an incredibly wide range of habitats and habits, but the highest diversity of both groups and species occurs in circum-Amazonian tropical South America. *Solanum* is recognised by its usually pentamerous flowers with fused sepals and petals, stellate to pentagonal corollas, and stamens with short filaments and anthers opening by terminal pores. The genus was one of Linneaus’s (1753) larger, with 23 species mostly described from European or African material. The French botanist Michel-Félix Dunal included 235 species in his thesis (Dunal 1813), mostly the result of extensive European exploration of the Americas. By 1816, when Dunal revised this work (Dunal 1816), this number had risen to 321; many of these additional taxa were based on specimens collected by Alexander von Humboldt and Aimé Bonpland in tropical South America (see Knapp 2007b). The last time the genus was monographed in its entirety was in Candolle’s *Prodromus* (Dunal 1852) which included 900 *Solanum* species. Subsequent work on the taxonomy of *Solanum* has largely been limited to rearrangements of infrageneric taxa, or to the species-level revisions of smaller groups within the genus (see references in Table 1) and floristic treatments. The combination of large numbers of species with relatively poorly circumscribed groups within the genus has meant that *Solanum* taxonomy has proceeded in a piecemeal fashion until relatively recently. A project funded by the United States National Science Foundation’s Planetary Biodiversity Inventory program begun in 2004 sought to redress this situation by attempting to accelerate species-level taxonomic work across the genus as a whole and at the same time providing a robust phylogenetic framework for this taxonomy. Work by participants of the ‘PBI Solanum’ project (see http://www.solanaceaesource.org ) will result in a modern monographic treatment of the entire genus available on-line. This treatment is part of this collaborative effort.

**Table 1.** The major clades of *Solanum* (after Bohs 2005). For revisions of individual species published on-line see Solanaceae Source (http://www.solanaceaesource.org ).

**Clade name (Bohs 2005)**	**Approx. # of species**	**Recent taxonomic monographs**
Thelopodium clade	3	Knapp 2001
Regmandra clade	11	Bennett 2008
Archaesolanum clade	9	Symon 1994
Normania clade	3	Bohs and Olmstead 2001
African Non-Spiny clade	ca. 15	in progress, Knapp and Vorontosova
Potato clade	ca. 200	Knapp and Helgason 1997; Spooner et al. 2004; Peralta et al. 2008; Tepe and Bohs 2011
Morelloid clade	ca. 75	in progress, Barboza and Särkinen
Dulcamaroid clade	43	This treatment
Wendlandii/Allophyllum clade	ca. 12	Bohs 1990; Clark et al. in prep.
Cyphomandra clade	50	Bohs 1994; Bohs 2001
Geminata clade	145	Knapp 2002a; Knapp 2008a
Brevantherum clade	ca. 80	Roe 1967, 1972; Carvalho 1996; Stern et al. 2012
Leptostemonum clade	ca. 450	Whalen 1979; Whalen et al. 1981, Vorontosova and Knapp 2012, in review; many other groups with monographs in progress

## History of *Solanum* Classification

Dunal (1813, 1816) divided the genus into two major groups, *Inermia* (unarmed solanums) and *Aculeata* (armed solanums), based on presence or absence of prickles. He renamed these at the sectional level (but illegitimately, as he cited groups he had previously named as sections, e.g., *Pteroidea* Dunal [Dunal 1816] in synonymy) “*Pachystemonum*”, for species with stout anthers and no prickles, and “*Leptostemonum*”, for species with tapering anthers, usually with stellate hairs and usually possessing prickles (Dunal 1852); these divisions were essentially the same as his *Inermia* and *Aculeata*. In Candolle’s *Prodromus*, Dunal (1852) erected a classification of subsections and ambiguous grades to reflect morphology, mostly of leaf division and inflorescence position. Dunal (1852) maintained as separate the genera *Lycopersicon* Mill. and *Cyphomandra* Sendtn., both now subsumed in a monophyletic *Solanum* (see Bohs 2005; Peralta et al. 2008). The German botanist Georg Bitter, who worked in Berlin, Bremen and Göttingen in the years between the two World Wars, published extensively in *Solanum* (see complete bibliography in Weber 1928) but never constructed a classification of the entire genus. In the mid-20^th^ century, Seithe (1962) compiled Bitter’s voluminous work on *Solanum* classification in a comprehensive system based largely on hair types; she recognised two major divisions, “chorus subgenerum *Solanum* (L.) Seithe”, the simple and branched hair solanums, and “chorus subgenerum *Stellatipilum* Seithe”, the stellate haired solanums. Danert (1967, 1970) used his own research on growth form and branching patterns in Solanaceae to propose a new subgeneric classification system for *Solanum*; he largely kept the same groupings as Seithe (1962) but recognised them at different ranks. D’Arcy (1972) assembled all the subgeneric names in *Solanum* and provided lectotypes for those that remained untypified. He divided the genus (in what he called a “provisional conspectus”) into seven subgenera (*Solanum* L., *Archaesolanum* Marzell, *Bassovia* (Aubl.) Bitter, *Brevantherum* (Seithe) D’Arcy (=*Minon* Raf.), *Lyciosolanum* Bitter, *Potatoe* (G.Don) D’Arcy and *Leptostemonum* Bitter [cited by D’Arcy as *Leptostemonum* (Dunal) Bitter]) and 52 sections based on combinations of characters he felt were more realistic than those used in the past. His subgenus *Leptostemonum* is equivalent to that of previous workers, but his divisions of the “non-spiny solanums” were quite different. Like Dunal (1852), but not Seithe (1962), D’Arcy excluded *Lycopersicon* and *Cyphomandra* from *Solanum*. Nee (1999) used only the New World species of *Solanum* to construct a simplified system in which he recognised 3 subgenera – *Bassovia* (comprised of the species previously recognised as *Cyphomandra* and their relatives, in which he included section *Pteroidea*, see Knapp and Helgason 1997), *Solanum* (the rest of the “non-spiny” solanums) and *Leptostemonum* (the “spiny” solanums). He recognised 21 sections with numerous subsections, and listed component taxa in each. Since the largest species diversity of *Solanum* occurs in the New World, Nee’s (1999) system has been very useful for focusing in-depth taxonomic studies on smaller, putatively monophyletic groups. Child and Lester (2001) provided a synopsis of infrageneric classification of *Solanum*, based largely on the work of Bitter and Seithe (1962). They did not indicate component species of any of their recognised sections, which comprise fewer species than those of Nee (1999).

DNA sequence data has revolutionised hypotheses of angiosperm relationships (APG III 2009) and *Solanum* is no exception. Based on cladistic analyses of DNA sequence data *Solanum* can be divided into 13 well-supported monophyletic clades (Bohs 2005; Weese and Bohs 2007; see Table 1), the largest of which is the group commonly known as the spiny solanums (the Leptostemonum clade), which is largely coincident with Dunal’s (1852) “*Leptostemonum*”. The largest non-spiny solanum clades (see Table 1) are the Geminata clade, the Potato clade, the Brevantherum clade and the Morelloid/Dulcamaroid clade; this last comprises all the herbaceous species previously placed in section *Solanum* (see Weese and Bohs 2007) and allied groups, plus the species previously placed in sections *Jasminosolanum* Bitter, *Dulcamara* (Moench) Dumort., *Lysiphellos* Bitter, *Nitidum* A.Child and *Andropedas* Rusby plus a variety of other taxa (see Bohs 2005 for discussion). Most current taxonomic work in *Solanum* is being undertaken in this cladistic framework underpinned by molecular data and concomitantly, more taxa are being added to molecular analyses to test its stability and robustness (e.g., Stern et al. 2011). As more taxa have been added the thirteen clades have generally been supported, but many species have not yet been included in molecular analyeses and their relationships remain to be tested.

## History of taxonomy of species of the Dulcamaroid Clade

The Dulcamaroid clade as recognised by Bohs (2005) and treated here is comprised of elements from several previously recognised subgenera and sections of *Solanum*. Species of the group are morphologically quite variable (see Morphology below); this in part accounts for these species’ chequered classification history and extensive synonymy. The common European woody nightshade or bittersweet, *Solanum dulcamara* L., has a long history of use in local medicine (Dunal 1813) in Eurasia (see species treatment of *Solanum dulcamara*). It was the only member of this group described by Linnaeus (1753), who recognised its extreme variability but did not explicitly name any of these variants, although he did explicitly name variants of other highly variable taxa. He did differentiate an African polynomial taken from Dillenius (1732) as “ß”. This polynomial refers to the South African species *Solanum africanum* Mill. (long known as *Solanum quadrangulare* Thunb.), provisionally a member of the African Non-Spiny clade (sensu Bohs 2005), further underlining the morphological similarities between the Dulcamaroid clade as here recognised and the unrelated “African Non-Spiny” (ANS) clade (see below).

Dunal (1813) reviewed the medicinal history of *Solanum dulcamara* in his thesis, and included it in his *Inermia* in a group characterised as “Foliis lobatis….” together with *Solanum lyratum* Thunb. and *Solanum triquetrum* Cav. In another group – “Foliis integerrimus…” he included an additional eight species now included in the Dulcamaroid clade as defined here; *Solanum dichotomum* Lour. (=*Solanum lyratum*) was put into a subgroup with lateral inflorescences, while the rest were said to have terminal inflorescences. Dunal had only seen herbarium specimens or live plants of three of these taxa (*Solanum dulcamara*, *Solanum triquetrum* and *Solanum pubigerum* Dunal) and relied on published descriptions for the rest. The greatly increased number of species (19 names recognised as members of the Dulcamaroid clade as treated here) included in the updated synopsis (Dunal 1816) were scattered over three groups based on leaf division. *Solanum seaforthianum* Andrews was part of Dunal’s (1816) group containing members of section *Pteroidea* (sensu Knapp and Helagson 1997, part of the Potato clade, Bohs 2005), *Solanum cyrrhosum* Dunal (=*Solanum seaforthianum*) was included in a group with *Solanum dulcamara* and *Solanum lyratum*; and *Solanum nitidum* Ruiz & Pav. (together with other members of the *Solanum nitidum* species group sensu Knapp 1989) with various members of the Geminata clade (sensu Knapp 2002a, 2008a) such as *Solanum sessile* Ruiz & Pav. and *Solanum oblongifolium* Dunal. Dunal’s division of *Solanum* into groups was quite detailed, but definition of the groups themselves was not completely parallel (see Knapp 1983 for a discussion of sectional nomenclature in *Solanum*). It is clear that the species now recognised here as members of the Dulcamaroid clade were in different groups due to their highly variable leaf morphology that can be pinnatifid to simple on a single stem (see below). Dunal’s (1852) *Prodromus* treatment included many more species of the Dulcamaroid clade as defined here; he mostly treated these taxa as members of his subsections *Dulcamara* (in the groups *Dulcamara* and *Subdulcamara*) and *Micranthes* (in the group *Oppositifolia* * *Indubitaria*). He included *Solanum viscosissimum* Sendtn. in his section *Tuberarium* (group *Potatoe* with the potatoes and tomatoes), based on its deeply pinnatifid leaves. Difficulty in grouping these taxa which today we recognise as closely related is perhaps understandable as label data from 19^th^ century herbarium specimens is sparse and usually does not include any mention of habit. Dependence upon leaf morphology and inflorescence position to determine relationships in Dunal’s systems would easily be foiled by the extremes of variability in leaf shape and inflorescence size found in the Dulcamaroid clade.

Seithe (1962) examined 36 species (representing 24 of the species recognised here and 12 species relegated to synonymy here) of the Dulcamaroid clade in her analyses of hair types in the genus. Of these, 11 were classified only as members of subgenus *Solanum* without assignment to a section; Seithe was unsure as to the affinities of these taxa. The rest of the species were included in her sections *Dulcamara* (e.g., *Solanum dulcamara*, *Solanum lyratum*), *Lysiphellos* (e.g., *Solanum decorticans* Sendtn. [=*Solanum inodorum* Vell.]), *Jasminosolanum* (e.g., *Solanum seaforthianum*, *Solanum jasminoides* Paxt. [=*Solanum laxum* Spreng.]), *Anthoresis* (e.g., *Solanum aureum* Dunal, *Solanum storkii* C.V.Morton & Standl.) and *Pseudocapsicum* (e.g., *Solanum triquetrum*). Those taxa included in *Anthoresis* are all the members of the *Solanum nitidum* species group (sensu Knapp 1989) and were grouped together based on the possession of highly branched trichomes. Danert (1970) recognised the same set of sections (renaming section *Anthoresis* as “section *Holophyllum* Walp.”), but did not list any of the component species in his system, so understanding his concepts of specific relationships is difficult. He did suggest a close relationship between sections *Dulcamara*, *Jasminosolanum* and *Aculeigerum* Seithe (equivalent in part to the Wendlandii/Allophyllum clade of Bohs 2005) and the vining taxa from Africa (recognised as sections *Afrosolanum* Bitter, *Lemurisolanum* Bitter, *Benderianum* Bitter, *Macronesiotes* Bitter, and *Quadrangulare* Bitter). He pointed out the extreme morphological similarity between members of his section *Holophyllum* and section *Brevantherum* Seithe; species in both these groups have dichasial branching, but they differ in their trichome morphology. Danert (1970), despite his emphasis on architecture in classification, put more weight on the trichome characters and agreed with Seithe (1962) that his sections “*Holophyllum*” (the same as Seithe’s *Anthoresis*) and *Brevantherum*, while similar morphologically, were not close evolutionarily.

D’Arcy (1972) included most of the vining dulcamaroid species in his subgenus *Potatoe* (G.Don) D’Arcy, along with the potatoes (section *Petota* Dumort.) and section *Regmandra* (Dunal) Ugent, in sections *Dulcamara* and *Jasminosolanum* (typified with *Solanum dulcamara* and *Solanum jasminoides* [=*Solanum laxum*] respectively). This placement was based on their possession of a scandent habit, pinnate leaves and articulation of pedicels above the base (D’Arcy 1972). Species such as *Solanum pulverulentum* Pers. (=*Solanum cutervanum* Zahlbr.) were included in his subgenus *Brevantherum* (=subgenus *Minon*) in section *Holophylla* (G.Don) Walp. due to their possession of complex branched trichomes (D’Arcy 1972). *Solanum decorticans* (=*Solanum inodorum*) was segregated as section *Lysiphellos* of subgenus *Solanum*. He included all of the African vining species (which all have simple leaves) in subgenus *Solanum* as Bitter’s original sections (sensu Seithe 1962). D’Arcy’s separation of the members of the Dulcamaroid clade in two different subgenera was the first explicit hypothesis as to the affinities of these taxa on a higher level.

Knapp (1989) studied the members of section *Holophylla* and recognised that the section was morphologically heterogeneous and almost certainly not monophyletic. She segregated a group of 11 species, the *Solanum nitidum* species group, and identified the unusual pedicel insertion morphology (see below) as a morphological synapomorphy for the group. Her cladistic treatment of the *Solanum nitidum* species group based on morphology was one of the first explicitly phylogenetic analyses of an entire clade in *Solanum*. *Solanum pubigerum* Dunal and *Solanum aligerum* Schltdl. were suggested as sister taxa to the *Solanum nitidum* group, but Knapp (1989) suggested the entire group was more closely related to other woody tree solanums such as the members of section *Geminata* (G.Don) Walp. (the Geminata clade, see Knapp 2002a, 2008a) rather than to species associated with *Solanum dulcamara* and its relatives. Child (1998) established subsection *Nitidum* A.Child based on this monograph (Knapp 1989), but did not suggest further relationships or examine material in detail. Child (1998) also segregated the Californian species *Solanum xanti* A.Gray (=*Solanum umbelliferum* Eschsch.) and its relatives as section *Californisolanum* A.Child and followed D’Arcy (1972) in suggesting these species were members of subgenus *Potatoe* and related to “dulcamaroid taxa”.

In his treatment *Solanum* in the New World Nee (1999) included the species of the Dulcamaroid clade in sections *Dulcamara* (the majority of species included here) and *Holophylla* (members of the *Solanum nitidum* species group sensu Knapp 1989). His circumscription of section *Holophylla* also includes all of the members of the Geminata clade as treated by Knapp (2008). All the vining species (plus other species previously recognised as section *Parasolanum* Bitter, e.g. *Solanum corymbosum* Jacq.) were combined into a single section *Dulcamara* by Nee (1999); included in his circumscription are the African *Solanum terminale* Forssk. and its relatives, which in molecular analyses form the distinct African Non-Spiny clade (Bohs 2005). Although the two groups are morphologically quite similar, in that both contain species that climb with the aid of petioles (see below), sequence differences thus far clearly differentiate them into two clades. *Solanum corymbosum* and other members of section *Parasolanum* group are resolved unambiguously as part of the Morelloid clade in all molecular analyses (Bohs 2005; Weese and Bohs 2007).

Two years after the publication of Nee’s (1999) system for New World taxa, Child and Lester (2001) provided a synopsis of infrageneric taxa in *Solanum* worldwide. This system is an attempt to bring together information from the scattered publications on *Solanum* taxonomy at the time, rather than a comprehensive reanalysis of infrageneric classification based on new data. Child and Lester (2001) were inspired by Bitter’s many works (see above) and their system is in large part based on his divisions of the genus. No component species in any groups are listed, and although “type” species are given, these are not validly published. They treat members of the Dulcamaroid clade (as far as one can tell from the designations of “types”) in their subgenus *Solanum*, section *Holophyllum* (as subsection *Nitidum* and the “species group *Solanum valdiviense* sensu A. Child”) and subgenus *Potatoe* (as “Dulcamaroid taxa”, sections *Dulcamara*, *Jasminosolanum* and *Californisolanum*). Following D’Arcy (1972), they treated all of the African vining species as members of subgenus *Solanum*.

The unexpected relationship of the *Solanum nitidum* species group (sensu Knapp 1989) with the vining dulcamaroid solanums was revealed in analyses of DNA sequence data (Bohs and Olmstead 1999; Bohs 2005; Weese and Bohs 2007). The monophyly of the Dulcamaroid clade as recognised here had previously never been suggested. Earlier ideas about the relationships of these groups had always emphasised their habit differences and segregated them clearly, but the presence of a “pedicel sleeve” (sensu Knapp 1989) is a clear synapomorphy uniting these seemingly very different taxa. Knowledge of plants in the field also has helped to find characters uniting these taxa; many of the shrubby taxa are scandent or scrambling, a character only apparent with good field notes or observations. The distant relationship of these taxa and the vining species from Africa and Madagascar (the African Non-Spiny clade sensu Bohs 2005) is somewhat surprising, given that they share the unusual characters of twining petioles and pedicel insertion into a small “sleeve”. Only three species (albeit from three of Bitter’s sections!) from the African Non-Spiny clade have been included in molecular analyses to date and with more in-depth sampling relationships are likely to become clearer. What is clear from analyses using molecular sequence data is that the members of the Dulcamaroid clade are not closely related to the potatoes as had suggested D’Arcy (1972) and Child and Lester (2001). In the analysis using the plastid gene *ndhF* the sister group to the Dulcamaroid clade is the Morelloid clade, comprising members of section *Solanum* and relatives (Bohs 2005). A subsequent analysis (Weese and Bohs 2007) using two plastid regions (*ndhF* and *trnT-F*) and a nuclear region (*waxy*, GBSSI or Granule Bound Starch Synthetase) resolved the Dulcamaroid clade (with 93% bootstrap support) as part of a polytomy involving the Morelloid, Normania, Archaesolanum and African Non-Spiny (ANS) clades (see Figure 1 in Weese and Bohs 2007).

Although the structure of relationships within the Dulcamaroid clade is very poorly resolved and the taxa sampled relatively few, a few sister relationships are apparent within the clade. In the *ndhF* analysis (Bohs 2005) five groups form a polytomy: (*Solanum ipomoeoides* Chodat & Hassl. [=*Solanum uncinellum* Lindl.]) + (*Solanum calileguae* Cabrera+ *Solanum jasminoides* [=*Solanum laxum*]) + (*Solanum crispum* Ruiz & Pav. + *Solanum amygdalifolium* Steud.) + (*Solanum aligerum* + *Solanum nitidum*) + (*Solanum dulcamara* + (*Solanum seaforthianum* + *Solanum wallacei* (A.Gray) Parish)). Weese and Bohs (2007) used fewer species in their three-gene analysis, and recovered *Solanum pubigerum* + (*Solanum nitidum* + *Solanum aligerum*) as sister to a polytomy of (*Solanum calileguae* + *Solanum ipomoeoides* [=*Solanum uncinellum*]) + (*Solanum crispum* + *Solanum amygdalifolium*) + (*Solanum dulcamara* + *Solanum seaforthianum*). These relationships are consistent with those recovered using only *ndhF* sequences and support the distinction of a group corresponding to section *Holophylla* in the narrow sense including *Solanum nitidum*, *Solanum pubigerum* and *Solanum aligerum*, but not *Solanum crispum*. Relationships amongst the vining dulcamaroid species are less clear, but additional sampling will certainly help with the delimitation of smaller groups within the clade. Most of these relationships are very poorly supported in both analyses, so any sister taxon relationships should be regarded as preliminary.

## Morphology

**Figure 1.**
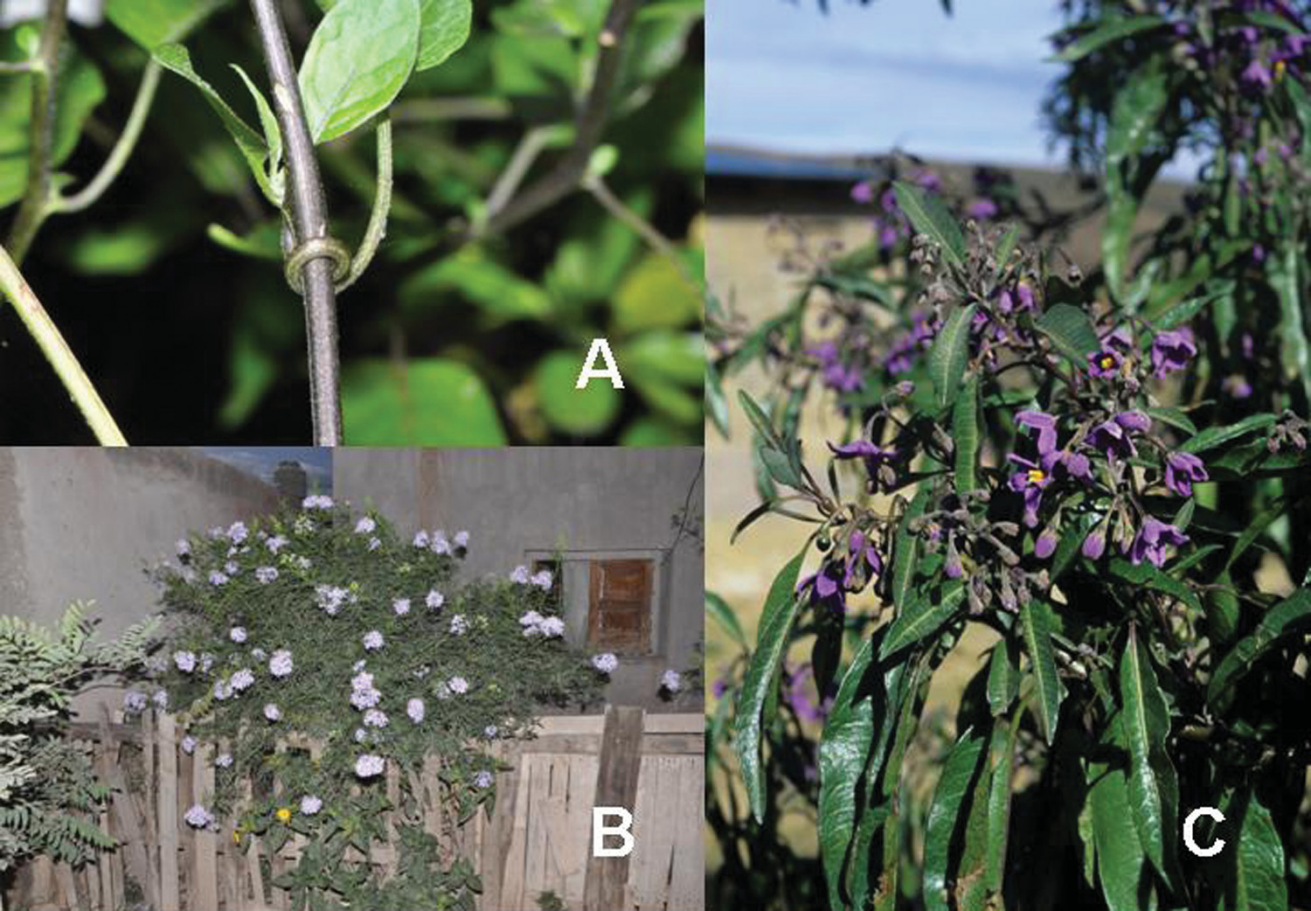
Habit of members of the Dulcamaroid clade. **A** Twining petioles that aid in climbing (*Solanum laxum*, cultivated in London UK) **B** *Solanum angustifidum* (*Barboza et al. 3489*) as a lax shrub on a fence in Argentina **C** Erect shrub of *Solanum nitidum* (*Nee et al. 51755*, Cochabamba, Bolivia).

*Habit*. Members of the Dulcamaroid clade are all woody plants and vary from shrubs or lax shrubs to vines (see Figure 1). Some species (e.g., *Solanum dulcamaroides* Poir., *Solanum uncinellum*) are large canopy lianas, while other vining species are woody only at the base (e.g., *Solanum dulcamara*, *Solanum lyratum*), especially in temperate climates. *Solanum umbelliferum* in California has sprawling herbaceous stems arising from a thick woody rootstock or base. Some species spread by underground stems; *Solanum dulcamara* is often considered a pest of gardens in Europe for this reason. Many of the species of the group are small shrubs that only become vining as they grow (i.e., *Solanum viscosissimum*) while others spread over adjacent vegetation, but never truly climb (e.g., *Solanum angustifidum* Bitter) and others are erect and never twine (e.g., *Solanum aligerum*, *Solanum endoadenium* Bitter, *Solanum storkii*).

Climbing members of the group have petioles that coil around supports (*Solanum laxum* [as *Solanum jasminoides*] was characterised as a “leaf-climber” by Darwin 1865) anchoring the ascending stems (Figure 1). This climbing mechanism is not found elsewhere in *Solanum* except in the African Non-Spiny (ANS) clade, which in molecular phylogenetic analyses is not closely related to the Dulcamaroid clade (Weese and Bohs 2007; T. Särkinen and S. Knapp, unpublished data). Darwin (1865) measured the time taken for petioles to clasp supports and found it very slow compared to other twining plants; once a petiole has clasped a support it accumulates woody tissue and thickens considerably.

*Stems*. The main axis of the plant is a typical *Solanum* sympodium formed by a succession of lateral axes with alternate leaves arranged in a 1/3 phyllotaxic spiral, and inflorescences are terminal at the end of each sympodial unit (Danert 1958). Danert (1958, 1967, 1970) and Child and Lester (1991) documented sympodial units and anthoclades (patterns of foliar lateral branches and associated inflorescences) in the Solanaceae. The number of leaves per sympodium is very regular and of major taxonomic significance in the entire genus *Solanum* (see Knapp 2001 for a further discussion).

In the Dulcamaroid clade sympodial units are almost always plurifoliate with many (or an irregular number) of leaves between each inflorescence in contrast to other groups such as the tomatoes (Peralta et al. 2008) or the Geminata clade (Knapp 2008) in which sympodial units are composed of 3 or fewer leaves. Plurifoliate sympodial units have the leaves arranged in a spiral fashion along the stems, and the leaves in members of the dulcamaroids are never geminate (paired, as seen in the Geminata clade, Knapp 2008). Most members of the group have monochasial branching, with a single axillary branch arising from below the inflorescence, giving the stems a zig-zag appearance (Danert 1958). Members of the *Solanum nitidum* group are either monochasial or some species have dichasial branching (see Knapp 1989); in species with dichasial branching two axillary shoots develop and the branching is dichotomous (e.g., *Solanum stenophyllum* Dunal). Most of these species have a mixture of the two branching types but are predominantly one or the other.

**Figure 2.**
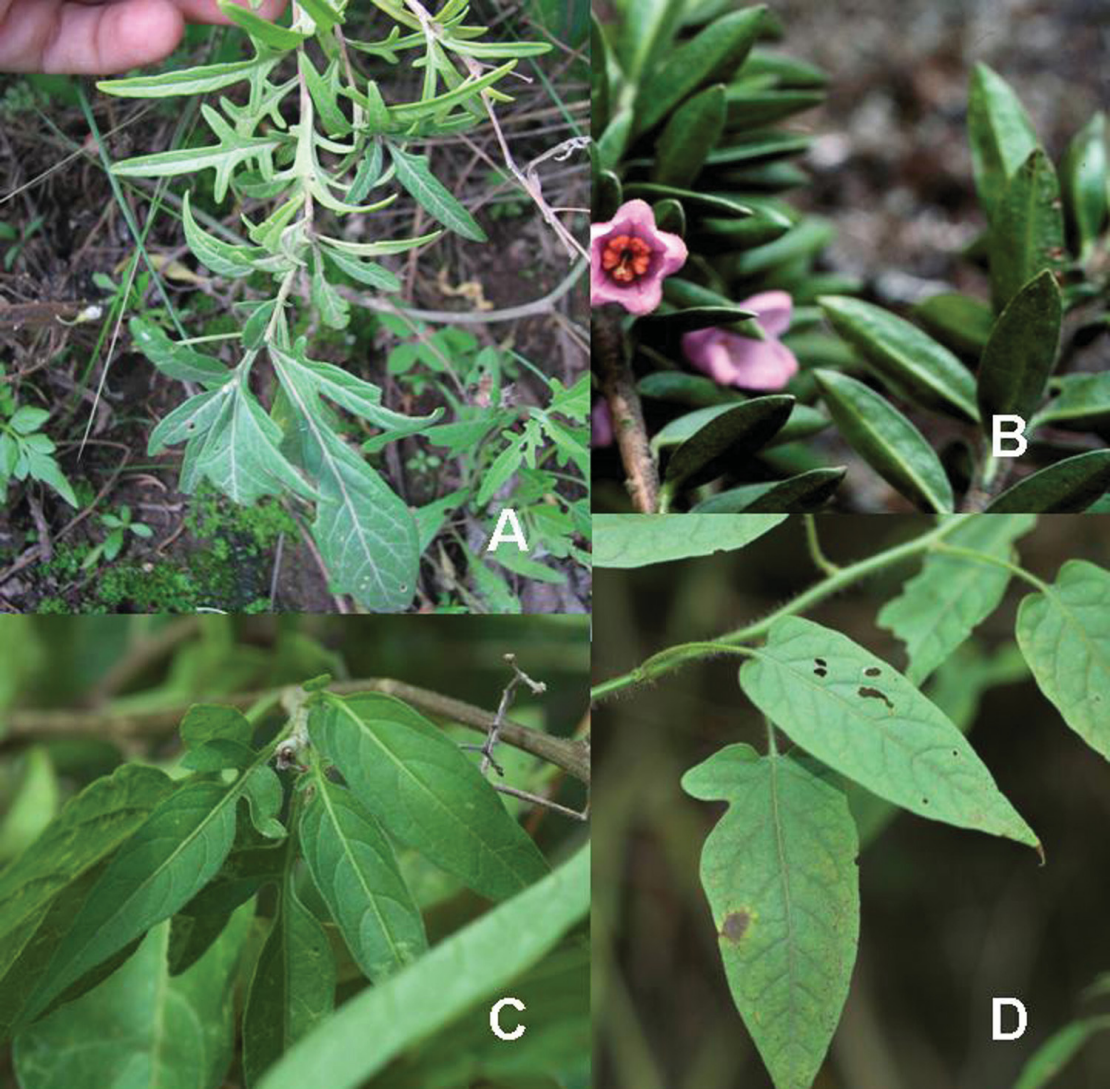
Leaf variation in members of the Dulcamaroid clade. **A** Leaf variation along a single short stem, *Solanum salicifolium* (*Barboza et al. 3467*, Catamarca, Argentina) **B** Thick leathery leaves of *Solanum macbridei* (*Nee & Solomon 30182*, La Paz, Bolivia) **C** Uneven leaf lobing in *Solanum dulcamara* (*Knapp IM-10102*, Tuscany, Italy) **D** Variable membranous leaves of *Solanum lyratum* (*Knapp et al. 10139*, Jiangxi, China).

*Leaves*. The leaves of members of the Dulcamaroid clade are extremely variable, both across the group and within individual plants (see Figure 2 and individual species illustrations). Leaves on reproductive stems are either simple or variously pinnatifid/pinnate. There is always a wing of leaf tissue along the midrib between the lobes connecting all the dissections that is more pronounced than that found in the tomatoes and potatoes (see Peralta et al. 2008 for discussion of tomato leaf morphology), but some species such as *Solanum angustifidum* and *Solanum viscosissimum* or some forms of *Solanum lyratum* have such deeply divided leaves that they are best characterised as pinnate as is commonly done in other such taxa (see Peralta et al. 2008).

**Figure 3.**
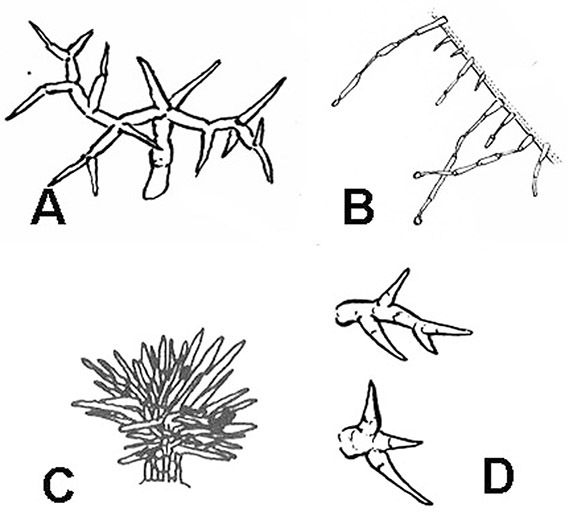
Trichome types found in members of the Dulcamaroid clade. **A** Loose dendritic **B** Simple glandular **C** Echinoid **D** Furcate.

Leaf polymorphism is common in the group and in many species individual stems bear both simple and pinnatifid leaves (e.g., *Solanum dulcamara*, *Solanum lyratum*, *Solanum salicifolium* Phil.). The control of this has not been investigated, but may have to do with hormonal balance due to light or nutrients. In other species, most notably in some plants of *Solanum viscosissimum*, lateral inflorescence-bearing shoots have simple leaves while main axis leaves are pinnatifid or pinnate. This variability has lead to the many synonyms in some species; different leaf morphologies collected from different parts of the plant have been described as separate taxa. In some taxa there seems to be some genetic control of leaf morphology; variants with deeply dissected leaves of *Solanum lyratum* all come from a few localities and may represent local populations with fixed genetic differences. A set of specimens of the otherwise consistently simple-leaved *Solanum uncinellum* from near Iquitos have deeply dissected leaves but are identical in flower and fruit characteristics. Leaf morphology in pinnately leaved species of *Solanum* is complex, and governed by a set of interacting genes (Holtan and Hake 2003); this variation can make identification difficult in some species.

Leaf divisions are narrowly elliptic, elliptic to broadly elliptic, or obovate; the divisions rarely are petiolulate. The base is usually asymmetric, and varies from truncate or rounded, to cordate or lyrate. The apex (of leaves and leaflets) is rounded, acute or acuminate. The margins are entire, never serrate or crenate and the margins are straight or revolute.

The juvenile leaves of most of the species for which they have been observed are usually pinnate (pinnatifid), but because botanists usually only collect reproductive stems, this is not known for many species. Where juvenile leaves are known I have included this information in species descriptions. A sterile specimen with pinnate leaves from Iquitos (*Williams 8481*, F) was identified by MacBride (1964) as *Solanum dulcamara*. In my view this is a specimen of the pinnate-leaved seedling of *Solanum uncinellum*, not a distinct species, nor is it a southern range extension of *Solanum dulcamara*.

Leaf size and texture can also vary enormously depending on the environment or position on the stem. In *Solanum triquetrum*, which grows in arid environments in the southwestern USA, leaves formed in wet periods are larger, sometimes by several times, than those from dry periods (see Figure 97). Leaf texture varies across the group from very delicate and membranous in some temperate species (e.g., *Solanum lyratum*) to thick and coriaceous in taxa from high elevations (e.g., *Solanum macbridei* Hunz. & Lallana). Turreson (1922) grew thick-leaved plants of *Solanum dulcamara* from seaside habitats in Sweden in common inland gardens with thin-leaved plants from more inland forest areas and found that the coastal forms developed thinner (and less pubescent) leaves away from their natural habitat. Clough et al. (1979), also working with *Solanum dulcamara*, found that light levels were a primary factor influencing leaf morphology and physiological performance; a major effect was on leaf specific weight, or thickness. Leaf texture and morphology are both important species specific characters in the group, although within species variation in some taxa is very large.

*Pubescence*. Trichome types and density are very useful for species recognition in *Solanum*. Previous classifications of the genus (Seithe 1962) have been constructed using primarily trichome types, with groups defined by possession of stellate, branched or simple trichomes. Later research (e.g., Knapp 2002a; Bohs 2005) has shown that trichome types do not define monophyletic groups in *Solanum*, but that they can be useful at the species level. As with many of the vegetative features of members of the Dulcamaroid clade, trichomes are very variable (see Figure 3 for trichome types). Trichomes of members of the Dulcamaroid clade are either uniseriate (consisting of a single file of cells) and either simple or branched, or multiseriate and echinoid and densely dendritic (Roe 1971; Knapp 1989; Mentz and Stehmann 1992). Species of the *Solanum nitidum* group (sensu Knapp 1989) have branched or densely branched trichomes; some of these approach the echinoid and tree-like trichomes characteristic of the Brevantherum clade (see Roe 1971) with numerous very short branches clustered at the tip (e.g., *Solanum stenophyllum* and *Solanum storkii*).

Simple trichomes are either eglandular or glandular, with the glands usually consisting of a single cell. *Solanum kulliwaita* S.Knapp has unusual bead-like terminal cells on the trichomes of the inflorescence that all appear to be glandular (Figure 52); this type of trichome has also been observed in *Nicotiana* L. in Australia (Marks et al. 2011). Glandular trichomes are very consistently present in some species (e.g., *Solanum lyratum*, *Solanum viscosissimum*), while in others possession of glandular or eglandular trichomes appears to be polymorphic within species (e.g., *Solanum endoadenium*, *Solanum umbelliferum*). Possession of glands has often been used as an infraspecific marker in other groups of *Solanum* (for example some species of section *Solanum*, Edmonds 1982) but molecular work with these taxa has shown that the trichome types do not define monophyletic groups within these species but instead are polyphyletic and represent polymorphism (Oyet 2004; Manoko 2007).

**Figure 4.**
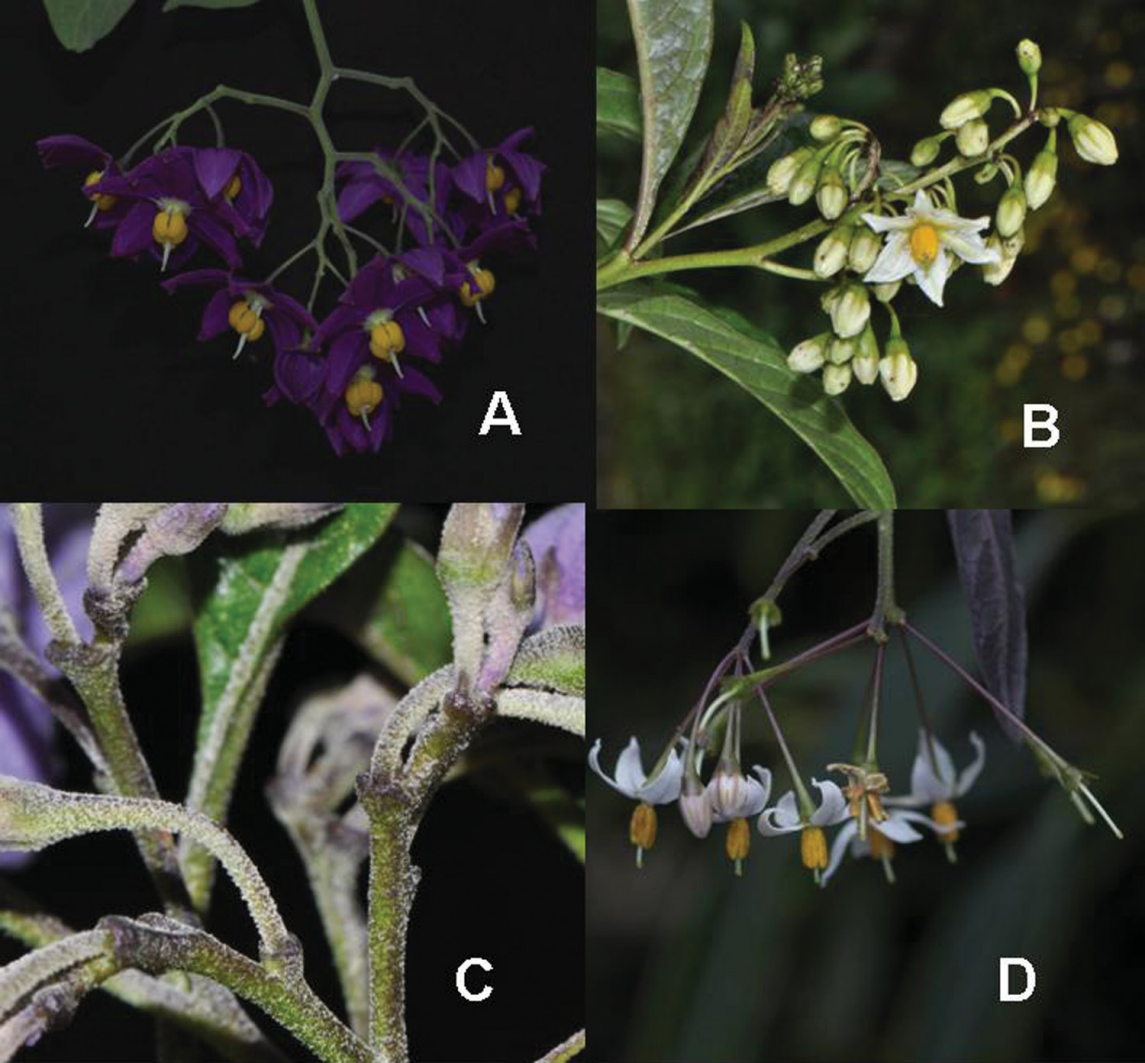
Inflorescences of members of the Dulcamaroid clade. **A** Open cyme of *Solanum dulcamaroides* (cultivated in Nijmegen, The Netherlands A44750197) **B** Grouped flowers on platforms of *Solanum aligerum* (*Knapp et al. 10436*, Cusco, Peru) **C** Pedicel sleeve of *Solanum nitidum* (*Knapp et al. 10222*, Huancavelica, Peru) **D** Contrasting pedicel colour in *Solanum valdiviense* (cultivated in Royal Botanic Garden, Edinburgh, UK, *Knapp IM-10105*).

Terminal glands also occur on branched trichomes, but these trichomes are more typically eglandular. Several different branched trichome types occur in the Dulcamaroid clade: 1) uniseriate trichomes with few branches (e.g., *Solanum sanchez-vegae* S.Knapp, Figure 84), 2) uniseriate trichomes with many congested branches (e.g., *Solanum aureum*, Figure 20), 3) multiseriate or uniseriate trichomes with densely congested very short branches (tree-like or “Tannenbaumartig” sensu Seithe 1962, e.g., *Solanum stenophyllum*, Figure 94) and 4) multiseriate echinoid trichomes (e.g., *Solanum storkii*, Figure 95). These latter two types have very short, often unicellular branches and are found only in the *Solanum nitidum* group.

*Inflorescences*. The inflorescences of members of the Dulcamaroid clade, as in most Solanaceae, are terminal with a subtending lateral bud. The inflorescence usually occupies a position at the end of branches, but if growth continues from the axillary bud it can appear lateral. In some species the inflorescences are borne on what appear to be short shoots with very reduced leaves (e.g., *Solanum inodorum*, *Solanum valdiviense* Dunal); the flowering and non-flowering shoots of these plants are very divergent morphologically and the short shoots often have smaller leaves (see Figures 49, 104). *Solanum inodorum* is extreme in this regard with the flowering shoots seeming to be axillary in larger leaves. In fact, the inflorescence is terminal on a short shoot with much reduced, almost bract-like, leaves.

The basic inflorescence, as in all other species of *Solanum*, is a scorpoid cyme with a variety of branching patterns. In the Dulcamaroid clade inflorescences are usually very large and highly branched. Turrill (1935) made crosses between a form of *Solanum dulcamara* with 1-flowered inflorescences he called “uniflora” and the more typical many-branched form, and suggested that the branches of the complex inflorescence were dichotomous and that subtending bracts were suppressed. Lippman et al. (2008) found that the highly branched inflorescence of *Solanum crispum* was developmentally similar to *compound inflorescence* (*s*) mutants of tomato; each shoot inflorescence meristem produces multiple flowers in a proliferating manner. They suggest that delays in floral termination (perhaps mediated by *S*, or the genetic pathway that *S* defines) coupled with sequential expression of *AN* (*anantha*, another tomato inflorescence mutant) provide a developmental framework for the modulation of sympodial branching in the entire family. In the Dulcamaroid clade flower number varies from 2-3 (*Solanum salicifolium*, Figure 82) to more than 100 (*Solanum sanchez-vegae*, Figure 84); within a plant inflorescence size can vary greatly with inflorescence age as each inflorescence continues to branch and produce flowers throughout its “life”.

In any given inflorescence only a few flowers are open at a time (up to about 40-50 in species with large inflorescences like *Solanum sanchez-vegae*). Flowers are generally more congested near the inflorescence branch tips; in *Solanum aligerum* and *Solanum pubigerum* the flowers are grouped onto tiny “platforms”, or highly congested groups of 3-5 flowers (see Knapp 1989: Figure 2b; Figures 10, 74). The pedicel scars are irregularly and relatively widely spaced, and the space between scars is generally greater more basally in the inflorescence. Differentiation between the peduncle and the end of the shoot is difficult to determine in most species, but in some species (e.g., *Solanum kulliwaita*), the pubescence of the inflorescence is distinct from that of the shoot.

*Pedicels*. The pedicels of species in the Dulcamaroid clade are distinctive in being inserted into a tiny sleeve of tissue that appears to arise from the inflorescence rhachis (see Figure 2c in Knapp 1989); this is one of the few morphological synapomorphies for the clade. This is most pronounced in the species of the *Solanum nitidum* group, in which the sleeve is sometimes up to 2 mm long and forms a small cup in which the base of the pedicel sits (Figure 2C). In most species the pedicel is articulated slightly above the base, leaving a small peg or cup on the inflorescence axis; this accentuates the zig-zag appearance of the inflorescence architecture that results from sympodial growth. The pedicel articulation in *Solanum boldoense* Dunal & A.DC. of Cuba is unusual in being in the distal third of the pedicel, often directly beneath the calyx tube (see Figure 22). The colour of the pedicel sleeve is sometimes (e.g., *Solanum dulcamara*) different from the pedicel (see Figure 4D), further supporting the idea that the sleeve is anatomically part of the inflorescence rhachis.

Pedicel orientation in the species of the group is generally deflexed or slightly nodding at anthesis. In fruit, those species with pendent inflorescences generally have deflexed pedicels, but shrubby species such as those of the *Solanum nitidum* group have erect pedicels in fruit (e.g., *Solanum coalitum* S.Knapp).

*Calyces*. The calyx is typically sympetalous and 5-merous with the tube and lobes more or less equal in size. The shape of the calyx lobes varies considerably and is a useful character for species identification. Lobes vary from mere undulations of the calyx rim (e.g., *Solanum boldoense*, *Solanum uncinellum*) to deltate (many species), quadrate (e.g., *Solanum aligerum*, *Solanum pyrifolium* Lam.) or lanceolate (e.g. *Solanum imbaburense* S.Knapp); apices are usually acute to acuminate, but are occasionally rounded (e.g., *Solanum angustifidum*) or elongate (e.g., *Solanum viscosissimum*). Pubescence of the calyx tube and lobes usually parallels that of the pedicels and inflorescence rhachis, but is generally sparser.

**Figure 5.**
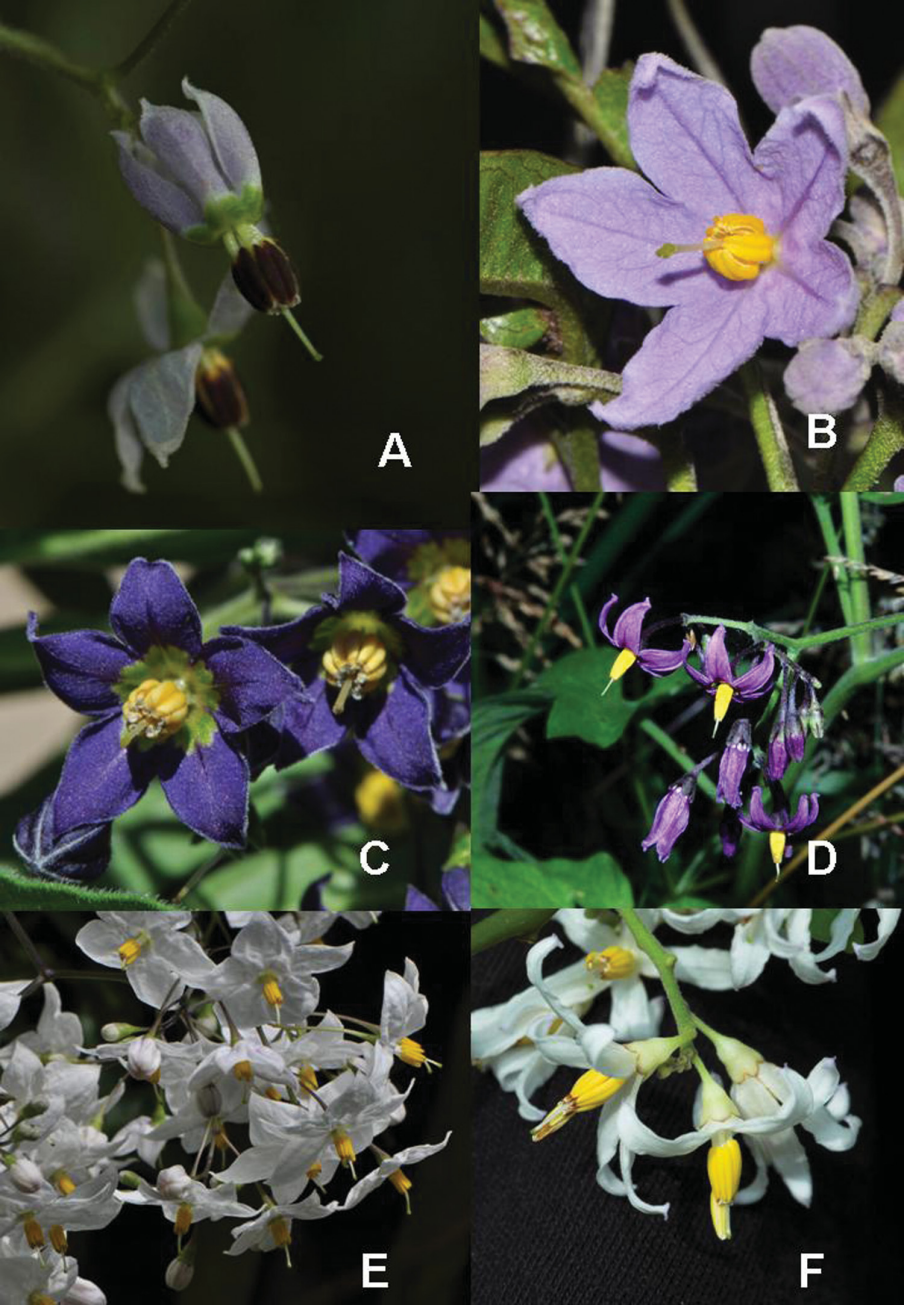
Flowers of members of the Dulcamaroid clade. **A** *Solanum lyratum* (*Knapp et al. 10142*, Hubei, China) **B** *Solanum nitidum* (*Knapp et al. 10222*, Huancavelica, Peru) **C** *Solanum endoadenium* (*Barboza et al. 3476*, Catamarca, Argentina) **D** *Solanum dulcamara* (Kent, UK) **E** *Solanum laxum* (cultivated in London, UK) **F** *Solanum uncinellum* (cultivated in Nijmegen, The Netherlands 994750113).

*Corollas*. The corolla of members of the Dulcamaroid clade is sympetalous, 5-merous and regular, in common with the vast majority of *Solanum* species. Color is either white or varying shades of purple; many species have populations of both color forms, and in *Solanum dulcamara* these have been described many times at the infraspecific level. *Solanum laxum* growing in full sun often has pale violet rather than the more commonly encountered white flowers, but the degree to which flower color is environmentally or genetically determined is not clear. At the base of the corolla tube is often a ring or irregular area of differently colored tissue refered to as the eye. In the group of species found in the temperate zone (e.g., *Solanum dulcamara*, *Solanum lyratum*, *Solanum umbelliferum*) at the base of each corolla lobe is a small pair of shiny green dots often ringed with darker green or white tissue. These have been variously interpreted as “pseudo-nectaries” functioning in pollinator attraction (Buchmann et al. 1977), but specific field studies have not been undertaken to test this. This character can be difficult to see in herbarium specimens. The center part of *Solanum* flowers is usually UV absorbent and seen by bees as a dark guide to the prominent anther cone (Buchmann et al. 1977). *Solanum endoadenium* has a prominent green eye, but the tissue is not shiny, perhaps indicating it is not homologous with the similar structure in *Solanum dulcamara* (see Figure 5).

Corolla shape varies from deeply stellate (*Solanum uncinellum*, Figures 5, 101) to pentagonal (*Solanum wallacei*, Figure 108). The lobes are triangular and usually either spreading (*Solanum dulcamaroides*, Figures 5, 38) or strongly reflexed (*Solanum lyratum*, Figures 5, 61) at anthesis. *Solanum macbridei* is unusual in having campanulate corollas (Figure 63). The tips are usually somewhat cucullate (e.g., Figure 14D) and are papillose to densely pubescent. In those species with dense overall pubescence, the abaxial surface of the corolla lobes is also usually densely pubescent. Corolla diameter varies from approximately 1 cm (*Solanum endoadenium*) to almost 5 cm (*Solanum wallacei*); most species have corollas that range from 1.5–3 cm in diameter.

*Anthers*. Anthers of members of the Dulcamaroid clade conform to the typical poricidal morphology of all other species of “non-spiny” *Solanum* (see Knapp 2001, 2002a). In most taxa the pore usually “unzips” during anther dehiscence to form a tear-drop shaped slit (Figure 5 and species illustrations), from which pollen is shed during vibratile pollination (Buchmann 1986). Some species of the Dulcamaroid clade have pores that do not elongate at all (e.g., *Solanum dulcamaroides*, *Solanum seaforthianum*), or only elongate slightly with age (e.g., *Solanum valdiviense*). Anthers in the group are generally ellipsoid, but those of *Solanum dulcamara* and *Solanum uncinellum* are elongate and tapering and those of *Solanum dulcamaroides* and *Solanum boldoense* are almost spheroidal. Anthers of members of most species are loosely connivent, while those of *Solanum dulcamara* are tightly connivent, and are held together physically with a sticky “glue” (Glover et al. 2004) such that the pores act as a single opening to a visiting bee. The anthers of *Solanum aureum* occasionally appear pubescent due to the prescence of elongate papillae on the dorsal surface (see Figure 20).

All five anthers in most of the species of the group are morphologically identical; some taxa, however, have filaments of different length, making the flowers zygomorphic. This filament length difference ranges from quite subtle (*Solanum seaforthianum*, Figure 86) to very conspicuous (*Solanum uncinellum*, Figure 5F, 101). Flowers of *Solanum flaccidum* Vell. that have just opened have filaments of equal length; the long filament apparently elongates over the course of anthesis. This has been observed in other species of *Solanum* (*Solanum turneroides* Bitter and *Solanum evolvuloides* Giacomin & Stehmann, members of section *Gonatotrichum* Bitter of the Brevantherum clade, see Stern et al. in press) and in the genus *Lycianthes* Hassl. (Dean 2001).

Pollen of members of the group is similar in size and shape to that of other species of *Solanum* (Buchmann 1986); it is tricolporate with a relatively smooth exine and held in yellow “pollen shamming” anthers. Some plants of *Solanum lyratum* have dark purple anthers; this appears not to be related to light or exposure but may be genetic. I have not seen dark anthers in any other member of the group, but anther color is variable in the Cyphomandra clade (section *Pachyphylla* Dunal, Bohs 2004). Nitrogen and protein content of pollen grains is the same as for other species of *Solanum* (Buchmann 1986), and is typical for a buzz-pollinated plant.

*Gynoecium*. The gynoecium is typically bicarpellate; the carpels are fused in a superior ovary with axillary placentation. The ovary is usually conical to globose or slightly ellipsoid and usually glabrous; if pubescent; the trichomes are only sparsely present at the apex (e.g., *Solanum cutervanum*). The flowers lack nectaries, as do all other species of *Solanum*. The style is simple, straight or slightly curved, glabrous or variously pubescent, and is exserted beyond the anthers in all but *Solanum luculentum* S.Knapp (see below). The stigma is capitate (e.g., *Solanum valdiviense*, *Solanum umbelliferum*) to clavate (e.g., *Solanum amygdalifolium*) and is sometimes distinctly bilobed (e.g., *Solanum uncinellum*). The ovules are anatropous and non-arillate.

**Figure 6.**
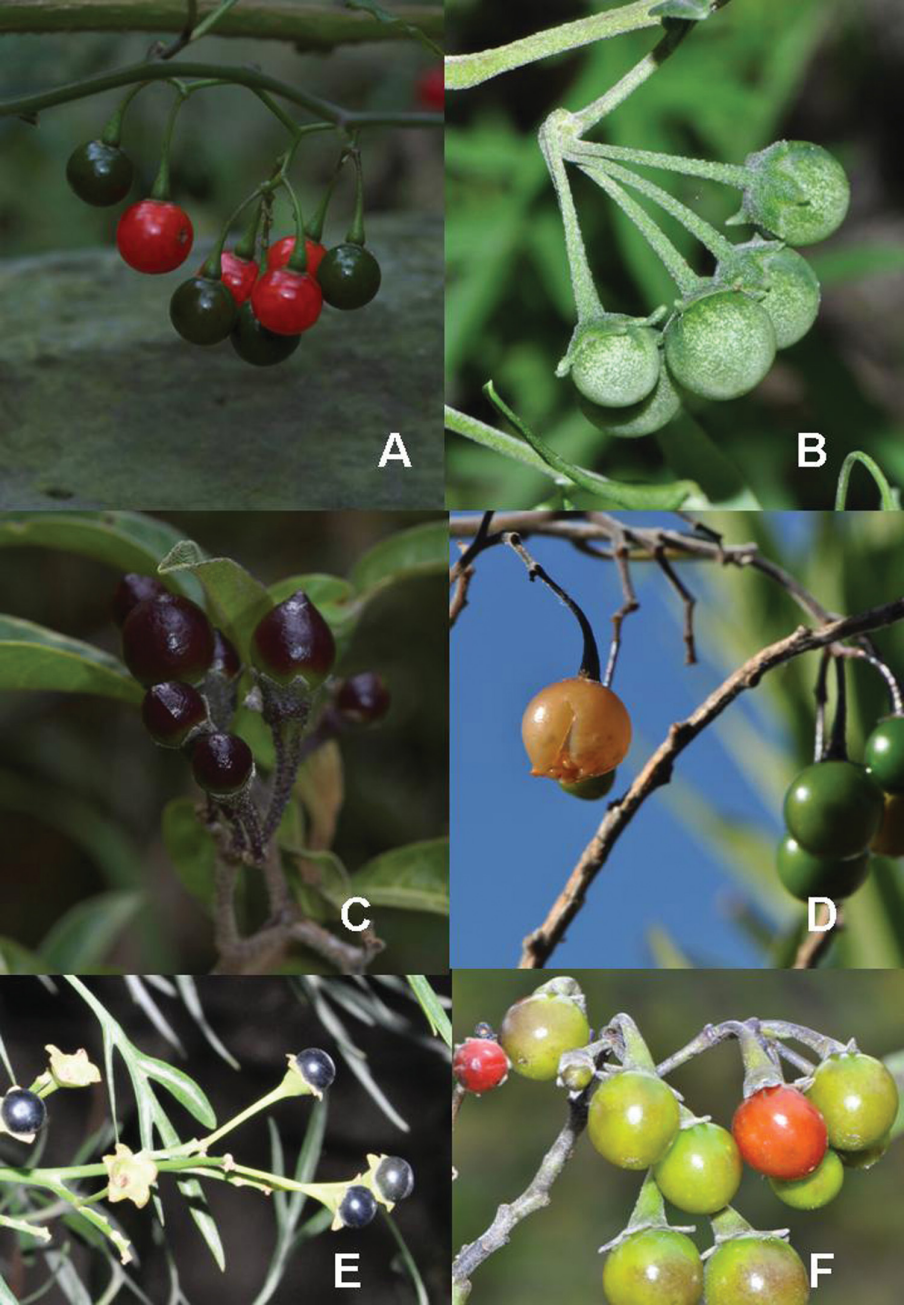
Fruits of members of the Dulcamaroid clade. **A** *Solanum pittosporifolium* (*Knapp et al. 10136*, Jiangxi, China) **B** *Solanum salicifolium* (*Barboza et al. 3467*, Catamarca, Argentina) **C** *Solanum storkii* (*Knapp & Monro 10053*, Bocas del Toro, Panama) **D** *Solanum angustifidum* (*Barboza et al. 3489*, Argentina) **F** *Solanum nitidum* (*Knapp et al. 10136*, Cusco, Peru).

*Fruits*. As with all species of *Solanum*, the fruit is a bicarpellate berry. Fruits of members of this group are usually brightly colored and juicy (see Figure 6 for respresentative photographs). Many species change fruit color through maturation; for example, *Solanum nitidum* has green immature fruits that pass through a stage of being bright red before turning dark purple at full maturity. This makes it difficult to use fruit color as an identification aid, and in *Solanum uncinellum* fruit color has been variously recorded on labels as blue, dark purple, red and white. Without in-depth field studies it is unclear whether these represent populational or regional differences or maturational changes. Species here regarded as synonyms of *Solanum umbelliferum* in California have sometimes been distinguished using “mature” fruit color (either green or black; Nee 1993b), but such color polymorphisms are found in other *Solanum* species (most notably *Solanum nigrum* L. in Europe, see Manoko 2007). Fruit surface morphology is usually shiny, but in some species the pericarp is distinctly dull and non-shiny (e.g., *Solanum sanchez-vegae*). *Solanum endoadenium* has bright orange fruits that appear to dry on the plant and release the seeds (see Figure 6D); this has not been studied in detail. Fruit diameter varies from less than 1 cm (*Solanum angustifidum*, *Solanum viscosissimum*, *Solanum pittosporifolium* Hemsl.) to approximately 4 cm (*Solanum wallacei*).

*Seeds*. Seed morphology has proved very useful in *Solanum* taxonomy. Enzymatic digestion of the outer testal walls reveals great variety in the morphology and structure of the lateral testal cell walls, varying both between and within groups (Whalen 1979; Lester and Durrands 1984; Knapp and Helgason 1997). Souèges (1907) provided an early study of *Solanum* seeds, including *Solanum dulcamara*. He described the development of the integument and recognised outer and inner layers of the seeds. Seeds of members of the Dulcamaroid clade are oval, obovate or kidney-shaped in outline and flattened laterally. The cells of the outer epidermal layer in some species (e.g., *Solanum dulcamaroides*, *Solanum lyratum*, *Solanum uncinellum*, *Solanum umbelliferum*) develop radial wall thickenings that form as “hair-like outgrowths” or “pseudohairs” in mature seeds (Lester and Durrands 1984; Lester 1991). These hair-like outgrowths often greatly enlarge the outer layer of the integument and the seed coat appears pubescent; seed measurements here include these projections. In seeds from mature fruits the pseudohairs are translucent, connate or fused laterally to each other and tightly adpressed to the epidermis giving a silky appearance to the seed surface, or if long and distinct produce a hairy and shaggy seed surface. In some species (e.g., *Solanum dulcamaroides*) the pseudohairs form a prominent wing around the margin of the seed. The testal cells form a reticulate or honeycomb pattern with cell outlines at the basal portions deeply sinuous and irregular (e.g., *Solanum boldoense*), pentagonal/rectangular (e.g., *Solanum seaforthianum*, *Solanum pubigerum*) or somewhat intermediate (e.g., *Solanum triquetrum*).

Seed number per berry varies from few (fewer than 10, *Solanum leiophyllum* Benth., *Solanum triquetrum*) to many (more than 100, *Solanum wallacei*), but most species fall in the range of 20-50 seeds per berry. Seed size varies from 1.5 mm length and 1 mm width to 8 mm length and 6 mm width. Color varies from yellow or pale brown to dark brown.

## Biology and natural history

*Habitats and distribution*. Members of the Dulcamaroid clade are both tropical and temperate, and occur in a wide range of habitats from deserts to lowland rainforest. Taxa occurring in the temperate areas of Asia and Europe occupy a wide range of habitats and elevations; *Solanum dulcamara*, for example, is found on sea coasts and extends into high elevations in the mountains of Italy and the Alps. Genetic work done in the early 20^th^ century (Turesson 1922) revealed some hereditary component to the immense morphological variation in this species, but habitat is also important (Turrill 1935). *Solanum lyratum* has been collected from sea level to more than 2000 m and is found in many forest types and even in weedy gardens in cities. Most of the species of the group are not of conservation concern (see Table 3 and species treatments) but some have narrow distributions and are assessed as endangered or vulnerable.

Most species in the group are South American, and in the Americas the species of the group range from southern Oregon (USA) to central Argentina and onto the islands of the Caribbean. In South America highest species diversity occurs in two areas, the forests of Atlantic coastal Brazil and the eastern slope of the Andes from Colombia to Argentina (see Table 2). This circum-Amazonian pattern of species richness is common in other groups of *Solanum* (Knapp 2002b); endemism follows the same pattern. Argentina has the highest species diversity in the Dulcamaroid clade (with Peru and Ecuador very close behind), due in part to the mixing of species from these two centres of species richness, in addition to several endemics or near endemics along the Andean flanks.

**Table 2.** Geographical distribution of New World species of the Dulcamaroid clade (species in square brackets are introduced or cultivated).

**Country**	**Species**
United States of America	[*Solanum dulcamara*], [*Solanum laxum*], *Solanum triquetrum*, *Solanum umbelliferum*, *Solanum wallacei*
Mexico	*Solanum aligerum*, *Solanum dulcamaroides*, *Solanum pubigerum*, *Solanum sousae*, *Solanum triquetrum*, *Solanum umbelliferum*
Guatemala	*Solanum dulcamaroides*, *Solanum muenscheri*, *Solanum pubigerum*, *Solanum aligerum*
Honduras	*Solanum pubigerum*, *Solanum aligerum*
El Salvador	*Solanum dulcamaroides*, *Solanum pubigerum*, *Solanum aligerum*
Nicaragua	*Solanum dulcamaroides*, *Solanum pubigerum*, *Solanum aligerum*
Costa Rica	*Solanum aligerum*, *Solanum pubigerum*, *Solanum seaforthianum*, *Solanum storkii*, *Solanum uncinellum*
Panama	*Solanum aligerum*, *Solanum uncinellum*
Colombia	*Solanum aspersum*, *Solanum aureum*, *Solanum dichroandrum*, [*Solanum laxum*], *Solanum luculentum*, *Solanum seaforthianum*, *Solanum stenophyllum*
Venezuela	*Solanum dichroandrum*, *Solanum luculentum*, *Solanum seaforthianum*, *Solanum uncinellum*
Guyana	*Solanum uncinellum*
Surinam	*Solanum uncinellum*
French Guiana	*Solanum uncinellum*
Ecuador	*Solanum agnoston*, *Solanum aspersum*, *Solanum aureum*, *Solanum coalitum*, *Solanum imbaburense*, *Solanum leiophyllum*, *Solanum nitidum*, *Solanum stenophyllum*, *Solanum uncinellum*
Peru	*Solanum aligerum*, *Solanum aureum*, *Solanum cutervanum*, *Solanum kulliwaita*, *Solanum macbridei*, *Solanum nitidum*, *Solanum ruizii*, *Solanum sanchez-vegae*, *Solanum uncinellum*
Bolivia	*Solanum aligerum*, [*Solanum angustifidum*], *Solanum endoadenium*, *Solanum nitidum*, *Solanum uncinellum*
Brazil	*Solanum amygdalifolium*, *Solanum flaccidum*, *Solanum inodorum*, *Solanum odoriferum*, *Solanum laxum*, *Solanum uncinellum*, *Solanum viscosissimum*
Paraguay	*Solanum amygdalifolium*, *Solanum flaccidum*, *Solanum laxum*, *Solanum uncinellum*
Argentina	*Solanum aligerum*, *Solanum amygdalifolium*, *Solanum angustifidum*, *Solanum calileguae*, *Solanum crispum*, *Solanum endoadenium*, *Solanum laxum*, [*Solanum odoriferum*], *Solanum salicifolium*, *Solanum uncinellum*, *Solanum valdivien*se
Uruguay	*Solanum amygdalifolium*, *Solanum laxum*
Chile	*Solanum alphonsei*, *Solanum crispum*, *Solanum valdiviense*
Cuba	*Solanum boldoense*, *Solanum seaforthianum*
Haiti/Dominican Republic	*Solanum pyrifolium*, *Solanum seaforthianum*
Lesser and Greater Antilles	*Solanum seaforthianum*

**Table 3.** Conservation assessments for members of the Dulcamaroid clade (calculated using methods in Moat 2007). For details see individual species treatments.

**Species**	**Preliminary Conservation Assessment**
*Solanum agnoston* S. Knapp	DD (Data Deficient)
*Solanum aligerum* Schltdl.	LC (Least Concern)
*Solanum alphonsei* Dunal	EN (Endangered)
*Solanum amygdalifolium* Steud.	LC (Least Concern)
*Solanum angustifidum* Bitter	LC (Least Concern)
*Solanum aspersum* S. Knapp	VU (Vulnerable)
*Solanum aureum* Dunal	LC (Least Concern)
*Solanum boldoense* Dunal & A.DC.	NT (Near Threatened)
*Solanum calileguae* Cabrera	EN (Endangered)
*Solanum coalitum* S. Knapp	CR (Critically Endangered)
*Solanum crispum* Ruiz & Pav.	LC (Least Concern)
*Solanum cutervanum* Zahlbr.	LC (Least Concern)
*Solanum dichroandrum* Dunal	LC (Least Concern)
*Solanum dulcamara* L.	LC (Least Concern)
*Solanum dulcamaroides* Poir.	LC (Least Concern)
*Solanum endoadenium* Bitter	LC (Least Concern)
*Solanum flaccidum* Vell.	LC (Least Concern)
*Solanum imbaburense* S. Knapp	DD (Data Deficient)
*Solanum inodorum* Vell.	LC (Least Concern)
*Solanum kulliwaita* S.Knapp	NT (Near Threatened)
*Solanum laxum* Spreng.	LC (Least Concern)
*Solanum leiophyllum* Benth.	NT (Near Threatened)
*Solanum luculentum* S. Knapp	LC (Least Concern)
*Solanum lyratum* Thunb.	LC (Least Concern)
*Solanum macbridei* Hunz. & Lallana	NT (Near Threatened)
*Solanum muenscheri* Standl. & Steyerm.	EN (Endangered)
*Solanum nitidum* Ruiz & Pav.	LC (Least Concern)
*Solanum odoriferum* Vell.	LC (Least Concern)
*Solanum pittosporifolium* Hemsl.	LC (Least Concern)
*Solanum pubigerum* Dunal	LC (Least Concern)
*Solanum pyrifolium* Lam.	NT (Near Threatened)
*Solanum ruizii* S. Knapp	VU (Vulnerable)
*Solanum salicifolium* Phil.	LC (Least Concern)
*Solanum sanchez-vegae* S. Knapp	NT (Near Threatened)
*Solanum seaforthianum* Andrews	LC (Least Concern)
*Solanum septemlobum* Bunge	LC (Least Concern)
*Solanum sousae* S. Knapp	NT (Near Threatened)
*Solanum stenophyllum* Dunal	LC (Least Concern)
*Solanum storkii* C.V.Morton & Standl.	VU (Vulnerable)
*Solanum triquetrum* Cav.	LC (Least Concern)
*Solanum umbelliferum* Eschsch.	LC (Least Concern)
*Solanum uncinellum* Lindl.	LC (Least Concern)
*Solanum valdiviense* Dunal	LC (Least Concern)
*Solanum viscosissimum* Sendtn.	LC (Least Concern)
*Solanum wallacei* (A. Gray) Parish	CR (Critically Endangered)

*Floral biology and pollination*. Most *Solanum* species have hermaphroditic flowers, but in some groups derived sexual systems are common (Whalen and Costich 1986; Anderson and Symon 1989; Knapp et al. 1998; Martine et al. 2009). Andromonoecy is very common in the Leptostemonum clade (Whalen and Costich 1986; Levin et al. 2006), as is dioecy (Anderson and Symon 1989; Martine et al. 2009). Dioecy is phylogenetically widespread in *Solanum*, with dioecious species occurring primarily in the spiny solanums (subgenus *Leptostemonum* Bitter, Anderson and Symon 1989), but also in the non-spiny solanums in the Potato (Anderson and Levine 1982), the Geminata (Knapp et al. 1998) and Brevantherum (S. Knapp, pers. obs., *Solanum punctulatum* Dunal from Jamaica) clades. *Solanum luculentum* of the Dulcamaroid clade has all the morphological correlates of a dioecious species, but has not yet been examined in detail to determine whether or not it is indeed truly dioecious. If so, *Solanum luculentum* represents the independent evolution of dioecy in an otherwise completely hermaphroditic group, rather than within a predominantly andromonoecious clade, as is common in the spiny solanums (Martine et al. 2009). The evolution of dioecy in a group with strictly hermaphroditic flowers has also occurred in the Potato clade (e.g., *Solanum appendiculatum* Dunal, see Anderson and Levine 1982).

Self-compatibility (SC) is widespread in *Solanum* (Whalen and Anderson 1981) and is essentially irreversible once acquired from the ancestral self-incompatible (SI) state ( 2003, 2006). Only three species of the Dulcamaroid clade have been assessed for their compatibility status: *Solanum dulcamara*, *Solanum laxum* and *Solanum wallacei* (Goldberg et al. 2010). Of these, only *Solanum laxum* has been shown to be SI (Whalen and Anderson 1981), but *Solanum dulcamara* flowers failed to set seed when isolated from visiting insects (Turrill 1935) and Cruden and Lyon (1985) scored *Solanum dulcamara* from Iowa as SI (“xenogamous”). Other authors, however, have stated that *Solanum dulcamara* is SC (Eijlander and Stiekema 1995; Vallejo-Marín and O’Brien 2006). In greenhouses and field plots in Nijmegen (Netherlands) self-crosses of *Solanum dulcamara* proved difficult and set few seed (G. van der Weerden, pers. comm., October 2010). There may be a geographic component to self-compatibility in *Solanum dulcamara*; those reporting SC have used American collections (naturalised) and those reporting SI have used European accessions. Buchmann et al. (1977) report that bagged flowers of *Solanum umbelliferum* (as *Solanum xanti*) failed to set fruit and concluded it was SI; this species, however, has not been included in recent large scale analyses of SI in the family (Goldberg et al. 2010). *Solanum crispum* grown in European gardens rarely sets fruit, and attempts to self flowers resulted in 0% fruit set (pers. obs.), but these experiments were not done in a controlled manner. That *Solanum wallacei*, an island endemic with a very narrow range, is SC is not at all surprising; it will be interesting to assess again the compatibility status of its close relative *Solanum umbelliferum*, which has been thought to be SI (Buchmann et al. 1977). Goldberg et al. (2010) have shown that lineages with self-incompatibility in the Solanaceae are more species-rich, due in part to higher extinction rates in SC lineages. It appears from the limited data available in the Dulcamaroid clade that widespread, clonal species (e.g., *Solanum laxum*, *Solanum dulcamara*, *Solanum crispum*) are SI, supporting the results of Vallejo-Marín and O’Brien (2006).

Flowers of all members of the Dulcamaroid clade are typical for *Solanum* and are buzz-pollinated by a wide variety of bees. In temperate regions species are visited by various species of bumblebees (*Bombus*); *Solanum dulcamara* and cultivated *Solanum laxum* and *Solanum crispum* in Europe are both visited by several species of bumblebees (pers. obs.). I have also observed bumblebees visiting several of the montane tropical species of the group (e.g., *Solanum nitidum* in Peru and Bolivia, *Solanum storkii* in Costa Rica), and *Bombus* species were common vibratile pollinators of *Solanum umbelliferum* (as *Solanum xanti*) in southern California (Buchmann et al. 1977). Pollen grains left on the anthers or near the pores by vibratile bees were collected by the gleaning bees from *Solanum umbelliferum* (*Andrena* spp., Andrenidae; *Stenodynerus cochisensis* (Viereck), Eumenidae; Buchmann et al. 1977). These bees are not as efficient at effecting pollination, however, as they are not necessarily in contact with the stigma on every visit.

**Figure 7.**
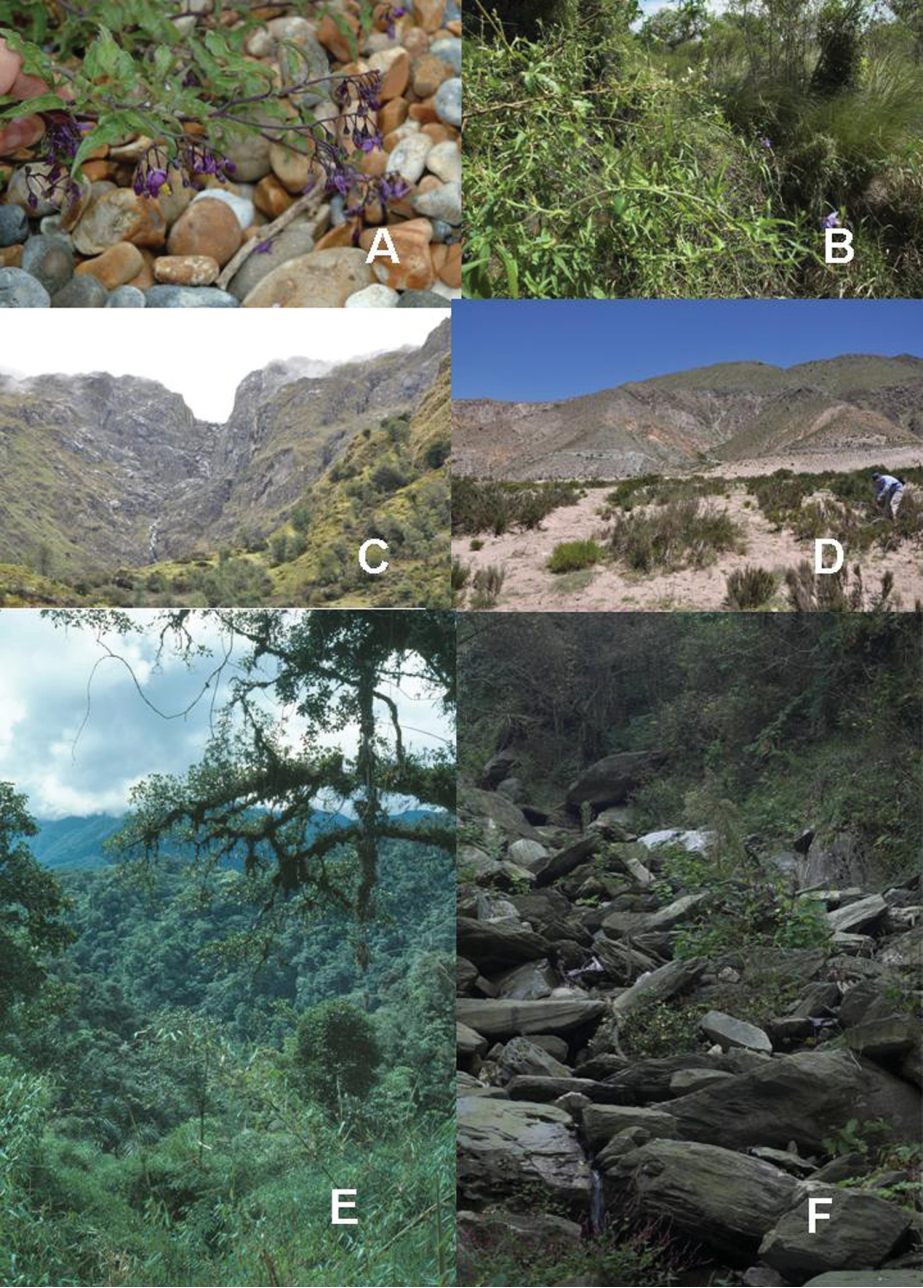
Habitats of members of the Dulcamaroid clade. **A** Gravel shingle at Rye Harbour Nature Reserve, coastal Sussex, UK (with *Solanum dulcamara*) **B** Dry forest grassland chaco habitat in Formosa, Argentina (with *Solanum amygdalifolium*) **C** Ceja de selva (near timberline) in Andean Peru (Cusco above Ollantaytambu) **D** High elevation dunes in Catamarca, Argentina (with *Solanum endoadenium*) **E** Eastern Andean slopes of Peru, premontane cloud forest **F** Rocky river course in the Lushan Mountains of Jiangxi, China.

## Species concepts

My goal for this revision has been to provide species circumscriptions for the members of this large and morphologically variable clade, while clearly highlighting those taxa where further in-depth research would be useful. Delimitation of species here basically follows what is known as the “morphological cluster” species concept (Mallet 1995): i.e., “assemblages of individuals with morphological features in common and separate from other such assemblages by correlated morphological discontinuities in a number of features” (Davis and Heywood 1963). Biological (Mayr 1982), phylogenetic (Cracraft 1989) and the host of other finely defined species concepts (see Mallet 1995) are almost impossible to apply in practice when dealing with large, complex groups such as the clades of *Solanum*, and are therefore of little utility in a practical sense. It is important, however, to clearly state the criteria for the delimitation of species, rather than dogmatically follow particular ideological lines (see Luckow 1995; Davis 1997). My decisions relied on clear morphological discontinuities to define the easily distinguished species. Specific characters used for recognition are detailed with each species description and in the keys. Potential reasons for variability and intergradation are recent divergence and hybridization. In this revision I have tried to emphasise similarities between populations instead of differences, which so often reflect incomplete collecting or local variation. I have not recognised subspecies or varieties, as I do not feel these are useful categories, either in a taxonomic or evolutionary sense. The variation is better described and documented, rather than formalised with a name which then encumbers the literature. I have been conservative in my approach, recognising as distinct entities those population systems (sets of specimens) that differ in several morphological characteristics. Minor differences in morphology, distribution, habitat, and ecology are important in some groups, where the common groundplan for the species is very similar. On the other hand, I have recognised several extremely widespread, polymorphic species, e.g., *Solanum dulcamara*, *Solanum lyratum*, *Solanum umbelliferum*, *Solanum uncinellum* and *Solanum viscosissimum*. In these species variation exists in certain characters, but the pattern of variation is such that no reliable units can be consistently extracted. In these cases the variability within and between populations seems more important than the variations of the extremes other taxonomists have recognised as distinct. In these cases I have described the variation, realizing that others may wish to interpret it differently.

## Materials and methods

Approximately 9000 collections were examined for this monograph from herbaria worldwide; specimens were seen at the following herbaria (all abbreviations from Index Herbariorum, http://sciweb.nybg.org/science2/IndexHerbariorum.asp ): A, AAU, B, B-W, BH, BM, BOLV, BRI, C, CAS, CICY, COL, CORD, DS, E, EA, ECON, EIU, F, FCQ, G, G-DC, GH, GOET, HA, HIB, HITBC, HBR, HUA, JBSD, JEPS, K, L, LAGU, LE, LIL, LOJA, LPB, MA, MBM, MEXU, MO, MOL, NY, OXF, P, PE, PMA, POM, Q, QCA, QCNE, RSA, S, SGO, SI, TI, UC, US, USM, UT, W, WIS, WU. Many of the species of the Dulcamaroid clade are common and widespread (e.g., *Solanum dulcamara*, *Solanum laxum*, *Solanum uncinellum*), so only selected specimens are cited; these represent the entire species range. All numbered collections are cited in the Index to Numbered Collections. Any collections without specific numbers are cited as s.n. (sine numero) in the Specimens examined sections of each species. Collection details for all specimens used for this monograph can be found on the Solanaceae Source website (http://www.solanaceaesource.org ) and in the data file available with this paper (see Supplementary Material). The specimens of *Solanum* at the New York Botanical Garden (NY) were databased as part of the PBI project; those specimens are best accessed through the NY Virtual Herbarium (http://sciweb.nybg.org/science2/VirtualHerbarium.asp ).

The JStor Plant Science website (http://plants.jstor.org ), established to display the images of type specimens digitised as part of the GPI (Global Plants Initiative) project funded by the Andrew W. Mellon Foundation, was an invaluable resource for images of type specimens, and many of the isotypes identified here were discovered via the website. Many type specimens in European herbaria were photographed in the early 20^th^ century by J.F. Macbride of the Field Museum (http://fieldmuseum.org/explore/our-collections/berlin-negatives ), including those at B, since destroyed. These photographs are cited here with a F negative number (F neg. #), but herbaria in which I have seen the photographs (usually mounted on sheets) are not cited. Conrad V. Morton of the Smithsonian also took photographs of many type and other nomenclaturally important specimens, these are cited as Morton negative numbers.

With all specimens of types I have cited the barcode or accession number of each sheet if possible. Barcodes are cited with no space between the herbarium acronym and the number (e.g., BM000845345), while for accession numbers I have inserted a hyphen between the acronym and the number (e.g., MO-00678987). Some herbaria such as MA, have barcodes and accession numbers that are the same, these are cited as on the barcode label (e.g., MA-634567); other herbaria such as MO have barcodes and accession numbers that differ, here I have cited the accession number as it is the only element visible without a barcode reader.

In the specimen citations I have selected specimens that represent the species range, and present these citations in alphabetical order by country rather than a geographical order. For those taxa that are introduced and often naturalised (*Solanum laxum* and *Solanum seaforthianum*) I have separated the specimen citations into those from countries in the native range (or presumed native range) and those from countries where the species is introduced or naturalised.

Citation of literature follows BPH-2 (Bridson 2004) with alterations implemented in IPNI (International Plant Names Index, http://www.ipni.org ) and Harvard University Index of Botanical Publications (http://kiki.huh.harvard.edu/databases/publication_index.html ). In particular, I have used the square bracket convention for publications in which a species is described by one author in a publication edited or compiled by another – the traditional “in” attributions such as Dunal in DC. for those taxa described by Dunal in Candolle’s *Prodromus Systematis Naturalis Regni Vegetabilis*; this work is cited here as Prodr. [A.P de Candolle] and the names thus attributed only to Dunal. For “ex” attributions I have cited only the publishing author, as suggested in the *Code* (McNeill et al. 2012). Author names are abbreviated according to IPNI (International Plant Names Index, http://www.ipni.org ).

Each species treated here has been assessed for a preliminary conservation status using the GIS protocols of Moat (2007) that involve calculation of AOO (Area of Occupancy) and EOO (Extent of Occurrence) from georeferenced specimen data. A grid cell size of 10% of the EOO was used (see Moat 2007); additional analyses using the 2 km^2^ grid size as recommended by IUCN (2001) were done, but specimen collection density made the results difficult to interpret. When threat status as determined by EOO and AOO differ, I have been conservative and selected the higher (more threatened) assessment as the overall assessment of the species (for an example, see *Solanum alphonsei*), and have used my knowledge of the biology of these species to suggest amendments to these calculated values. These assessments are not complete in the sense of IUCN (2001), but are indicative of the threat status for the taxa treated here. Any species known from fewer than 5 collections has been assessed as Data Deficient (DD); this does not mean these are not of conservation concern, but that the available data are not sufficient for their status to be modelled using the AOO/EOO method.

## Taxonomic treatment

### Generic and clade descriptions

#### Solanum

L., Sp. pl. 184. 1753.

http://species-id.net/wiki/Solanum

##### Lectotype species

designated by Henderson 1974: *Solanum nigrum* L. [I accept here the generic synonymy of D’Arcy (1972, 1974) with the addition of *Amatula* Moench, *Cyphomandra* Sendtn., *Lycopersicon* Mill., *Normania* Lowe, and *Triguera* Cav.].

##### Description.

Herbs, shrubs, trees, or vines, with or without prickles, glabrous or pubescent with unbranched or branched (including stellate), often glandular hairs. Leaves alternate or paired and frequently unequal in size, simple to pinnately lobed or compound, petiolate or sessile, without stipules, but sometimes with “pseudostipules” (Potato clade). Inflorescences cymose, branched or unbranched. Flowers usually perfect, (4-) 5-merous, actinomorphic or zygomorphic; calyx campanulate, sometimes accrescent in fruit, corolla rotate, campanulate, stellate, or urceolate, white, green, yellow, pink, or purple; stamens equal or unequal, the filaments generally short and inserted at the corolla base, the anthers basifixed, equal or unequal, blunt or tapered toward apex, opening by terminal pores, these sometimes expanding into longitudinal slits, or introrsely longitudinally dehiscent with age in sect. *Lycopersicon*; ovary 2-carpellate; ovules many; style articulated at base or above the base, usually slender; stigma capitate to elongate-clavate. Fruit a berry, usually fleshy but occasionally dry, usually many-seeded, the seeds often flattened; embryo curved, embedded in abundant endosperm. Chromosome number: n = 12, 23, 24, 48.

##### Discussion.

The generic description applies to *Solanum* including all those genera traditionally segregated from it: *Cyphomandra* (Bohs 1995), *Lycopersicon* (Spooner et al. 1993; Peralta et al. 2008), *Normania*, and *Triguera* (Bohs and Olmstead 2001). Data from chloroplast DNA sequences strongly support the inclusion of these segregates in a monophyletic *Solanum* (Bohs 2005). Some workers (e.g., Hunziker 2001) maintain these taxa as distinct genera.

### The “Dulcamaroid clade” sensu Bohs (2005)

*Dulcamara* Moench, Meth. 514. 1794.

Lectotype species, designated by D’Arcy 1972: *Dulcamara flexuosa* Moench (=*Solanum dulcamara* L.)

*Solanum* section *Dulcamara* (Moench) Dumort., Fl. Belg. 39. 1827.

Lectotype species, (designated by D’Arcy 1972: *Dulcamara flexuosa* Moench (=*Solanum dulcamara* L.)

*Solanum* subsection *Holophylla* G.Don, Gen. Hist. 2: 414.1832.

Lectotype species, designated by Seithe 1962: *Solanum cervantesii* Lag. (=*Solanum pubigerum* Dunal); superfluous lectotype designated by D’Arcy 1972: *Solanum pulverulentum* Pers. (=*Solanum cutervanum* Zahlbr.)

*Solanum* section *Holophylla* (G.Don) Walp., Repert. Bot. Syst. 3: 51. 1844.

Lectotype species, designated by Seithe 1962: *Solanum cervantesii* Lag. (=*Solanum pubigerum* Dunal); superfluous lectotype designated by D’Arcy 1972: *Solanum pulverulentum* Pers. (=*Solanum cutervanum* Zahlbr.)

*Solanum* subsection *Dulcamara* (Moench) Dunal, Prodr. [A.P. de Candolle] 13 (1): 28. 1852.

Lectotype species, designated by D’Arcy 1972: *Dulcamara flexuosa* Moench (=*Solanum dulcamara* L.)

*Solanum* grad. ambig. *Dulcamara* (Moench) Dunal, Prodr. [A.P. de Candolle] 13 (1): 28. 1852.

Lectotype species, designated by D’Arcy 1972: *Dulcamara flexuosa* Moench (=*Solanum dulcamara* L.)

*Solanum* grad. ambig. *Anthoresis* Dunal, Prodr. [A.P. de Candolle] 13(1): 28, 84. 1852.

Lectotype species, designated by Seithe 1962: *Solanum cervantesii* Lag. (=*Solanum pubigerum* Dunal); superfluous lectotype designated by D’Arcy 1972: *Solanum pulverulentum* Pers. (=*Solanum cutervanum* Zahlbr.)

*Solanum* grad. ambig. *Subdulcamara* Dunal, Prodr. [A.P. de Candolle] 13(1): 28, 84. 1852.

Lectotype species, designated by D’Arcy 1972: *Solanum ipomoea* Sendtn. (=*Solanum uncinellum* Lindl.)

*Solanum* section *Andropedas* Rusby, Mem. Torrey Bot. Club 4: 231. 1895.

Type species: *Solanum styracioides* Rusby (=*Solanum uncinellum* Lindl.)

*Solanum* section *Dulcamara* (Moench) Bitter, Bot. Jahrb. 54: 428. 1917, non section *Dulcamara* (Moench) Dumort. 1827.

Lectotype species, designated by D’Arcy 1972: *Dulcamara flexuosa* Moench (=*Solanum dulcamara* L.)

*Solanum* section *Anthoresis* (Dunal) Bitter, Bot. Jahrb. Syst. 54: 489. 1917, pro parte.

Lectotype species, designated by Seithe 1962: *Solanum cervantesii* Lag. (=*Solanum pubigerum* Dunal); superfluous lectotype designated by D’Arcy 1972: *Solanum pulverulentum* Pers. (=*Solanum cutervanum* Zahlbr.)

*Solanum* subsection *Lysiphellos* Bitter, Repert. Spec. Nov. Regni Veg. 16: 90. 1919.

Type species: *Solanum decorticans* Sendtn. (=*Solanum inodorum* Vell.)

*Solanum* section *Lysiphellos* (Bitter) Seithe, Bot. Jahbr. Syst. 81: 288. 1962.

Type species: *Solanum decorticans* Sendtn. (=*Solanum inodorum* Vell.)

*Solanum* section *Jasminosolanum* Seithe, Bot. Jahbr. 81: 291. 1962.

Type species: *Solanum jasminoides* Paxton (=*Solanum laxum* Spreng.)

*Solanum* subsection *Nitidum* A.Child, Feddes Repert. 109: 409. 1998.

Type species: *Solanum nitidum* Ruiz & Pav.

*Solanum* section *Californisolanum* A.Child, Feddes Repert. 109: 410. 1998.

Type species: *Solanum umbelliferum* Eschch.

**Description.** Vines or lax shrubs, woody at the base; stems weak and clambering or erect, sometimes winged, pubescent or glabrous, the trichomes unbranched or branched, never strictly stellate. Sympodial units plurifoliate. Leaves simple or pinnatifid, sometimes deeply so such that the leaves appear pinnate, green, glabrous or variously pubescent with branched or unbranched trichomes, the branched trichomes sometimes very compact and echinoid (multangulate); petioles well developed, in vines often twining to aid in climbing. Inflorescences terminal, sometimes appearing lateral due to stem growth, simple or more usually many times branched, with up to 100 flowers, not bracteate; peduncle present, often poorly distinguished from the leafy stem; pedicels inserted in a short sleeve of inflorescence tissue, articulated at the base inside the sleeve or just beneath the calyx (*Solanum boldoense*). Flowers 5-merous, actinomorphic or slightly zygomorphic, usually perfect, one species probably dioecious (*Solanum luculentum*); calyx 5-parted, glabrous or pubescent; corolla 5-parted, rotate to stellate, white to purple, sometimes with spots at the base of the lobes; stamens 5, the filaments equal or unequal, glabrous or pubescent; anthers yellow or occasionally purple, ellipsoid or tapering, separate to tightly connivent, dehiscing by terminal pores, these usually elongating to slits with age; ovary globose to conical, usually glabrous, occasionally pubescent; style straight or slightly curved; stigma capitate to clavate. Fruit a globose berry, yellowish green to red, orange or black when ripe, glabrous, the pericarp thin, shiny or matte; calyx lobes in fruit not accrescent. Seeds flattened, often appearing hairy from outgrowths of the lateral cell walls.

**Discussion.** As with many of the major clades of *Solanum* morphological synapomorphies in the Dulcamaroids are few (Bohs 2005), but members of the clade all have a distinctive pedicel “sleeve” (Knapp 1989) that appears to be an extention of the inflorescence axis in which the pedicel base sits. This “cushion of tissue” was described by Turrill (1935) in *Solanum dulcamara* as being of the color of the rhachis rather than the pedicel (see Figure 4D); this is the case in most species of the group, lending support to the idea that the “sleeve” is part of the inflorescence axis. Most members of the clade possess a combination of other characters that, although occurring elsewhere in *Solanum*, together distinguish the group; these are, 1) vining habit, 2) twining petioles, 3) large, terminal, branched inflorescences, 4) brightly colored fruits, 5) pinnatifid leaves at least in juvenile plants.

In the absence of a well-sampled phylogenetic study (see above), I have divided the Dulcamaroid clade into a series of morphologically and geographically delimited groups (see Table 4). These should not be interpreted as monophyletic groups or taxa, and future phylogenetic analyses including more species will certainly reveal structure within this large and variable clade. Preliminary phylogenetic analyses with a number of plastid regions (T. Särkinen, pers. comm.) have revealed that the division between the Morelloid and Dulcamaroid clades is not completely clear; for example, *Solanum salicifolium* and *Solanum valdiviense* are of ambiguous position. I have included them here on gross morphological grounds whilst realising their evolutionary relationships may be with other taxa. Species are presented here in alphabetical order without regard to putative evolutionary relationships.

**Table 4.** Informal species groups in the Dulcamaroid clade. For country distribution see Table 2 and individual species descriptions.

**Species group**	**Component taxa**	**Distribution**
“dulcamara group”	*Solanum dulcamara*, *Solanum lyratum*, *Solanum pittosporifolium*, *Solanum septemlobum*, *Solanum triquetrum*, *Solanum umbelliferum*, *Solanum wallacei*	Northern Hemisphere
“South American oddball group”	*Solanum amygdalifolium*, *Solanum aspersum*, *Solanum endoadenium*, *Solanum inodorum*, *Solanum luculentum*, *Solanum odoriferum*	Andes, SE Brazil
“aureum group”	*Solanum agnoston*, *Solanum aureum*, *Solanum calileguae*, *Solanum dichroandrum*, *Solanum kulliwaita*, *Solanum sanchez-vegae*	Andes
“laxum group”	*Solanum alphonsei*, *Solanum laxum*, *Solanum pyrifolium*, *Solanum sousae*, *Solanum viscosissimum*	SE Brazil, West Indies, Mexico
“dulcamaroides group”	*Solanum boldoense*, *Solanum dulcamaroides*, *Solanum seaforthianum*	West Indies, Mexico and Central America
“Andropedas”	*Solanum angustifidum*, *Solanum flaccidum*, *Solanum uncinellum*	South and Central America
“nitidum group”	*Solanum coalitum*, *Solanum crispum*, *Solanum cutervanum*, *Solanum imbaburense*, *Solanum leiophyllum*, *Solanum macbridei*, *Solanum muenscheri*, *Solanum nitidum*, *Solanum ruizii*, *Solanum stenophyllum*, *Solanum storkii*	Montane South and Central America
“aligerum group”	*Solanum aligerum*, *Solanum pubigerum*	Mexico to southern South America, large disjunction
Incertae sedis (possibly Morelloid clade)	*Solanum salicifolium*, *Solanum valdiviense*	Southern South America

In the species treatments here I have cited all type specimens I have seen with barcodes or accession numbers if possible (see Materials and Methods for citation style). Those specimens I have not seen but whose existence is known from the literature or from others are indicated as n.v. (non visi, not seen). For many names I have been unable to find specimens and in many cases specimens were not cited explicitly, but if an author worked primarily in a particular herbarium, I have indicated types with ‘?’ to help in future tracing of type specimens; many of these are for the synonyms of the widespread *Solanum dulcamara*, synonymy decisions in these cases were based on descriptions. Some of the infraspecific taxa, particularly in the widespread European species *Solanum dulcamara* were described from floristic accounts and did not have specimens cited in their original descriptions; I have not neotypified these.

### Artificial key to species of the Dulcamaroid Clade

**Table:** 

1	Stems and/or leaves pubescent with variously branched trichomes	2
–	Stems and leaves glabrous or the majority of trichomes simple and uniseriate (not branched)	28
2	Trichomes congested (tightly branched or echinoid) on stems and leaf undersides	3
–	Trichomes loosely branched on stems and leaf undersides	9
3	Trichomes of stems and leaves dendritic with short branches, not echinoid	4
–	Trichomes of stems and leaves echinoid (many branches from a single point or along an apparent “stem”)	5
4	Vines with golden trichomes; inflorescences many times branched; Andes	*Solanum aureum*
–	Shrubs with white or greyish white trichomes; inflorescences simple or at most furcate; Pacific coast of North America	*Solanum umbelliferum*
5	Stem not winged from decurrent leaf bases; style pubescent	6
–	Stem winged from decurrent leaf bases; style glabrous (if style pubescent, then the leaves densely golden pubescent abaxially)	7
6	Flowers 4–4.5 cm in diameter; bark of older stems pale grey or white; central Peru	*Solanum ruizii*
–	Flowers 1.5–2 cm in diameter; bark of older stems reddish brown; northern Peru and Ecuador	*Solanum cutervanum*
7	Leaves strongly discolorous when dry, the abaxial lamina surfaces completely obscured by the golden pubescence	*Solanum stenophyllum*
–	Leaves not strongly discolorous when dry, the abaxial lamina surface clearly visible	8
8	Calyx lobes deltate; trichomes on abaxial leaf surfaces in shallow pits; Central America	*Solanum storkii*
–	Calyx lobes long-triangular; trichomes scattered on stems and leaf surfaces, not in pits abaxially; Andes (southern Ecuador)	*Solanum stenophyllum*
9	Leaves coriaceous and shiny adaxially; venation often impressed or not easily visible above	10
–	Leaves membranous or somewhat fleshy, not markedly coriaceous and shiny; venation not impressed above	15
10	Trichomes sparse, confined to the leaf margins or in the axils near the midveins abaxially	11
–	Trichomes sparse to dense on the leaf lamina, at least abaxially	14
11	Leaves less than 2 cm long; corolla campanulate at anthesis; high Andes, Peru and Bolivia	*Solanum macbridei*
–	Leaves longer than 2 cm long; corolla spreading at anthesis; Andes of Ecuador	12
12	Vines; leaves with dendritic pubescence in the primary vein axils beneath	*Solanum agnoston*
–	Shrubs; leaves glabrous or with trichomes only at the margins	13
13	Calyx lobes long-triangular; northern Ecuador	*Solanum imbaburense*
–	Calyx lobes deltate; southern Ecuador	*Solanum coalitum*
14	Stem winged; corolla with dendritic trichomes abaxially; shrubs or possibly lax shrubs	*Solanum leiophyllum*
–	Stem not winged; corolla papillate abaxially; woody vines	*Solanum sanchez-vegae*
15	Trichomes of leaf undersides confined to the axils of the primary veins	*Solanum agnoston*
–	Trichomes variously distributed on leaf undersides, not in tufts in primary vein axils	16
16	Buds turbinate and pointed; anthers elongate	17
–	Buds ellipsoid or globose, not pointed; anthers ellipsoid or globose, not tapering at the tips	18
17	Anthers borne on unequal filaments, free; corolla densely papillate externally; Neotropics	*Solanum uncinellum*
–	Anthers borne on equal filaments, tightly fused into a cone; corolla variously papillate on margins; Eurasia, North America	*Solanum dulcamara*
18	Stems strongly angled or winged	19
–	Stems round, not angled or winged	20
19	Inflorescences terminal, many times branched; berries black; Mexico to Argentina	*Solanum aligerum*
–	Inflorescences borne on short shoots, simple or at most furcate; berries green or red; Chile	*Solanum valdiviense*
20	Glandular trichomes present (on any part of the plant)	21
–	Glandular trichomes absent	22
21	Glandular trichomes on inflorescence only (these simple); Andes	*Solanum kulliwaita*
–	Glandular trichomes on stems and leaves (these dendritic or simple); Pacific coast of North America	*Solanum umbelliferum*
22	Pubescence of lax transparent trichomes, not greyish white when dry	23
–	Pubescence greyish white when dry (probably pale beige on live plants), the trichome walls not transparent	24
23	At least some leaves lobed to pinnatifid (some appearing completely pinnate); flowers white; stem smooth; Argentina	*Solanum calileguae*
–	Leaves simple; flowers violet or white; stem warty from persistent leaf bases; Venezuela and Colombia	*Solanum dichroandrum*
24	Flowers with shiny green and white spots at the base of the corolla lobes; Pacific coast of North America	*Solanum umbelliferum*
–	Flowers uniformly purple or white (occasionally with a white “eye”, but not with shiny green spots); tropical Mexico to southern South America	25
25	Calyx lobes very small, broadly deltate; corolla fleshy, the tips of the lobes cucullate; anthers globose; style glabrous; woody vines; Mexico to Central America	*Solanum dulcamaroides*
–	Calyx lobes of various sizes, not minute; corolla membranous; anthers ellipsoid; style pubescent; shrubs or lax shrubs	26
26	Mature berries black; leaves densely pubescent on both surfaces; Guatemala	*Solanum muenscheri*
–	Mature berries red; leaves sparsely pubescent on both surfaces, or the abaxial surfaces more densely pubescent; South America	27
27	Leaf bases acute; leaf blades with 11–25 pairs of veins, these strongly parallel; Andes from Ecuador to Bolivia	*Solanum nitidum*
–	Leaf bases truncate to cordate; leaf blades with 6–12 pairs of veins, not strongly parallel; coastal Chile to Andean Argentina, widely cultivated	*Solanum crispum*
28	Leaves glabrous on the lower surface (usually both surfaces glabrous)	29
–	Leaves with at least some simple uniseriate trichomes on the surfaces (usually denser beneath or of equal density on both surfaces, if trichomes branched go to 1a)	47
29	Leaves of reproductive stems lobed or divided (if some leaves simple, then rarer than lobed leaves)	30
–	Leaves of reproductive stems simple (if some leaves lobed or divided, then rarer than simple leaves, both leaf types occasionally present in equal numbers in *Solanum viscosissimum*)	37
30	Anthers borne on filaments of differing lengths	31
–	Anthers borne on equal filaments	33
31	Flower buds turbinate and pointed; anthers elongate; abaxial surface of corolla lobes densely papillate (glabrous plants with lobed leaves extremely rare)	*Solanum uncinellum*
–	Flower buds not pointed; anthers ellipsoid; abaxial surface of corolla lobes glabrous or sparsely papillate at the tips	32
32	Vines; leaf lobe apices acute; berries red; anthers 2–3 mm long; Caribbean, widely cultivated	*Solanum seaforthianum*
–	Shrubs; leaf lobe apices rounded; berries black; anthers 5–6 mm long; Argentina and Bolivia	*Solanum angustifidum*
33	Inflorescences simple or at most once-forked, with < 10 flowers; shrubs	34
–	Inflorescences many times branched, usually open, with > 10 flowers; vines or lax shrubs	35
34	Leaf base attenuate; leaves lanceolate to narrowly elliptic; Andes of Argentina	*Solanum salicifolium*
–	Leaf base acute; leaves elliptic to ovate; Pacific coast of North America	*Solanum umbelliferum*
35	Leaves shallowly lobed, rhombic in outline; Chile	*Solanum alphonsei*
–	Leaves deeply lobed (pinnatifid), ovate or elliptic in outline	36
36	Plants completely glabrous; leaf lobe apices rounded; Argentina and Bolivia	*Solanum angustifidum*
–	Plants with some hyaline simple trichomes (if lower surface glabrous then upper leaf surface with simple uniseriate trichomes); leaf lobe apices acute; Brazil	*Solanum viscosissimum*
37	Leaves coriaceous and shiny; venation usually not easily visible on upper surface	38
–	Leaves membranous; venation visible on both surfaces	41
38	Shrubs or small trees; stems warty from raised leaf bases; flowers purple, fleshy; Ecuador	*Solanum coalitum*
–	Vines; stems not warty; flowers white, membranous	39
39	Bark green and shiny, not exfoliating; calyx truncate or with minute lobes; southeastern Brazil	*Solanum odoriferum*
–	Bark exfoliating; calyx variously lobed, never truncate	40
40	Inflorescence apparently axillary; flowers all perfect; southeastern Brazil	*Solanum inodorum*
–	Inflorescence terminal; flowers with either short or long styles, these apparently on different plants and the species dioecious; northern Colombia and Venezuela	*Solanum luculentum*
41	Inflorescences simple or at most once-forked, with < 10 flowers	42
–	Inflorescences many times branched, with > 10 flowers	43
42	Leaf base acute; Pacific of coast North America	*Solanum umbelliferum*
–	Leaf base attenuate, winged onto the stem; Andes of Argentina	*Solanum salicifolium*
43	Leaves linear to lanceolate (narrowly elliptic), more than twice as long as wide; leaf base acute or attenuate	44
–	Leaves ovate to elliptic, not more than twice as long as wide; leaf base truncate or cordate (occasionally acute)	46
44	Stems strongly angled or winged; flowers rotate	*Solanum amygdalifolium*
–	Stems rounded; flowers stellate	45
45	Inflorescences open, the flowers widely spaced; delicate often herbaceous vines; southeast Asia	*Solanum pittosporifolium*
–	Inflorescences congested, with the flowers clustered; robust vines or lax shrubs; Chile, widely cultivated	*Solanum crispum*
46	Pedicel articulation in the distal half, often near the base of the calyx tube; calyx truncate, with minute undulations rather than lobes; Cuba	*Solanum boldoense*
–	Pedicel articulation at the base in a small cup; calyx lobes deltate to narrowly deltate, irregular in size and shape; Brazil	*Solanum viscosissimum*
47	Trichomes glandular (at least in part) on leaves and/or inflorescences	48
–	Trichomes not glandular on any part of plant	53
48	Glandular trichomes on inflorescence only, absent from leaves; Andes	*Solanum kulliwaita*
–	Glandular trichomes present on stems and leaves	49
49	Shrubs; inflorescences simple or once branched; flowers purple with green spots at the base of the corolla lobes	50
–	Vines or lax shrubs; inflorescences many times branched; flowers usually lacking green spots, if purple with green spots, the lobes strongly reflexed	52
50	Flowers stellate; berries orange; leaves narrowly elliptic; northern Argentina and Bolivia	*Solanum endoadenium*
–	Flowers rotate to rotate stellate; berries green or purplish black; leaves elliptic to ovate in outline; Pacific coast of North America	51
51	Flowers 1–2.6 cm in diameter; berry 1–2 cm in diameter, with 50–60 seeds; mainland	*Solanum umbelliferum*
–	Flowers 3–4.5 cm in diameter; berry 3–4 cm in diameter, with >100 seeds; Santa Catalina Island, California	*Solanum wallacei*
52	Corolla 0.8–1.3 cm in diameter, stellate; berry bright red; southeast Asia	*Solanum lyratum*
–	Corolla 1.5–1.8 cm in diameter, rotate-stellate; berry black or purple-black; southeastern Brazil	*Solanum viscosissimum*
53	Flower buds turbinate; anthers elongate and tapering	54
–	Flower buds ellipsoid or globose, not pointed and turbinate; anthers ellipsoid	55
54	Anthers tightly fused in a cone; filaments equal; corolla lobes with green spots at the base; Eurasia and North America	*Solanum dulcamara*
–	Anthers not tightly fused; filaments unequal; corolla lobes uniform in colour; widespread in Neotropics	*Solanum uncinellum*
55	Leaves linear to lanceolate (narrowly elliptic), more than twice as long as wide	56
–	Leaves elliptic to ovate, less than twice as long as wide	60
56	Delicate vines or small lax shrubs; seeds appearing “pubescent”; southeast Asia	*Solanum pittosporifolium*
–	Shrubs, usually erect; seeds with the testa various, not apparently “pubescent”; Neotropics	57
57	Stems warty from prominent leaf bases; berries orange	*Solanum endoadenium*
–	Stems rounded, angled or winged, not warty; berries red	58
58	Stems rounded; inflorescences many times branched; fruiting pedicels erect; Mexico to Costa Rica	*Solanum pubigerum*
–	Stems strongly angled or winged; inflorescences simple or at most once-forked; fruiting pedicels nodding, not erect	59
59	Inflorescences lateral or leaf opposed; calyx lobes long-triangular; Argentina	*Solanum salicifolium*
–	Inflorescences borne on short shoots; calyx lobes deltate or quadrate; Chile	*Solanum valdiviense*
60	Leaves lobed on reproductive stems (if leaves simple then the stems winged)	61
–	Leaves simple on reproductive stems (if lobed leaves occur then only rarely and most leaves simple)	63
61	Stems rounded, not angled	*Solanum alphonsei*
–	Stems angled or slightly winged	62
62	Leaves hastate or basally lobed; inflorescences simple; corolla stellate; North America	*Solanum triquetrum*
–	Leaves deeply pinnatifid; inflorescence branched; corolla rotate-stellate; northern Asia	*Solanum septemlobum*
63	Leaf surfaces more or less equally and densely pubescent abaxially and adaxially	64
–	Leaf surface pubescence density on adaxial and abaxial surfaces differing, the abaxial surface usually more densely pubescent	65
64	Upper leaf surfaces bullate; flower buds narrowly ellipsoid; Andes	*Solanum aspersum*
–	Upper leaf surfaces not bullate; flower buds inflated, globose; Mexico and Central America	*Solanum dulcamaroides*
65	Anthers borne on unequal filaments	66
–	Anthers borne on equal filaments	67
66	Corolla 2–2.5 cm in diameter; Brazil	*Solanum flaccidum*
–	Corolla 1.5–2 cm in diameter; Mexico	*Solanum sousae*
67	Flower buds strongly inflated; corollas fleshy; anthers globose	68
–	Flower buds ellipsoid or slightly inflated; corollas membranous; anthers ellipsoid	69
68	Pedicel articulation in distal half, often at base of calyx tube; Cuba	*Solanum boldoense*
–	Pedicel articulation at base; Mexico and Central America	*Solanum dulcamaroides*
69	Pubescence of leaf undersides in distinct tufts in the vein axils; calyx lobes long-triangular; southern South America, widely cultivated	*Solanum laxum*
–	Pubescence of leaf undersides along veins, not concentrated in tufts in the vein axils; calyx lobes quadrate (square) or lacking (the calyx rim merely undulate)	70
70	Shrubs or small trees; flowers 1–1.4 cm in diameter; berries red; fruiting pedicels erect	*Solanum pubigerum*
–	Woody vines; flowers 1.5–2.5 cm in diameter; berries black or purplish black; fruiting pedicels pendent	71
71	Calyx lobes quadrate (square); style glabrous; Hispaniola	*Solanum pyrifolium*
–	Calyx lobes minute to lacking; style pubescent; central Mexico	*Solanum sousae*

### Synoptic character list for species of the Dulcamaroid Clade

This synoptic set of observations is intended to simplify the task of identifying a member of this large and highly variable group. Sterile plants of members of the Dulcamaroid clade are often difficult to identify, and even plants with either flowers or fruit can be difficult. I have included many leaf and whole plant characters so that sterile plants can be in many cases identified to a choice of a few taxa. Once this step has been done the user can read the descriptions and match distributions considering other characters not used in this list to the plant in hand. In the following list, character states are followed by a list of species epithets in alphabetical order. Epithets in parentheses indicate that the state is relatively uncommon in that species. Species that vary in a particular character, for example in leaf trichome distribution, will be listed for each state present in that species. Not all relevant characters are considered; for example, I have listed tapering and globose anthers, but not ellipsoid anthers – common in most species in the group. In this way diagnostic states can be easily seen. A question mark (?) following a species epithet indicates that the character is likely to occur, but has not been absolutely verified. Many species of the group are rather narrow endemics, occurring in only one country or region. Table 2 lists species recorded for each country of the Neotropics; this list must be used with care, as new collecting is constantly revealing range extensions.

Shrubs or small trees: *aligerum*, *angustifidum*, *coalitum*, *crispum*, *cutervanum*, *endoadenium*, *imbaburense*, *leiophyllum*, *macbridei*, *muenscheri*, *nitidum*, *pubigerum*, *ruizii*, *salicifolium*, *stenophyllum*, *storkii*, *umbelliferum*, *valdiviense*, *viscosissimum*, *wallacei*

Canopy lianas: *dulcamaroides*, *uncinellum*

Stems winged or strongly angled: *aligerum*, *amygdalifolium*, *valdiviense*, *salicifolium*

Stems warty or knobbly from persistent leaf bases: *coalitum*, *dichroandrum*, *endoadenium*

Bark shiny and exfoliating: *inodorum*, *luculentum*

Stems and leaves with echinoid and/or tree-like trichomes: *cutervanum*, *ruizii*, *stenophyllum*, *storkii*

Stems and leaves with golden trichomes: *aureum*, *ruizii*, *cutervanum*, *stenophyllum*, *storkii*

Glandular trichomes present anywhere on the plant (incl. only on the inflorescences): *coalitum*, *endoadenium*, *kulliwaita*, *lyratum*, *umbelliferum*, *wallacei*, *viscosissimum*

Dendritic (deer-antler-like) trichomes present: *agnoston*, (*aligerum*), *aureum*, *calileguae*, *coalitum*, (*crispum*), *dichroandrum*, *imbaburense*, (*kulliwaita*), *leiophyllum*, *macbridei*, *muenscheri*, (*nitidum*), *sanchez-vegae*, (*stenophyllum*), (*umbelliferum*), (*uncinellum*), (*valdiviense*)

Most leaves pinnatifid or lobed on mature reproductive stems: *alphonsei*, *angustifidum*, *calileguae*, *lyratum*, *seaforthianum*, *septemlobum*, *salicifolium*, *triquetrum*, *uncinellum*, *umbelliferum*, *viscosissimum*

Leaves coriaceous and shiny: *agnoston*, *coalitum*, *imbaburense*, *inodorum*, *leiophyllum*, *luculentum*, *macbridei*, (*odoriferum*), *sanchez-vegae*

Venation not visible on upper surface: *agnoston*, *inodorum*, *luculentum*

Lower surface of leaves with tufts of trichomes in vein axils: *agnoston*, *aligerum*, *flaccidum*, *laxum*

Both leaf surfaces densely pubescent with simple (never branched) trichomes: *aspersum*, *endoadenium*, *umbelliferum*, *wallacei*

Inflorescences simple: *umbelliferum*, *salicifolium*, *valdiviense*

Flower buds inflated: *boldoense*, *dulcamaroides*, *seaforthianum*, *laxum*

Flower buds narrowly ellipsoid: *aspersum*, *uncinellum*

Flowers with shiny green spots at the base of the corolla lobes: *dulcamara*, *endoadenium*, *lyratum*, *pittosporifolium*, *septemlobum*, *umbelliferum*, *wallacei*

Calyx lobes long-triangular or with long tips: *aligerum*, *alphonsei*, *crispum*, *imbaburense*, *kulliwaita*, *leiophyllum*, *muenscheri*, *nitidum*, *ruizii*, *salicifolium*, *stenophyllum*, *triquetrum*

Calyx lobes square (quadrate): *aligerum*, *pyrifolium*, *valdiviense*

Calyx lobes lacking (mere enations of the calyx rim): *boldoense*, *dulcamaroides*, *sousae*

Filaments unequal: *angustifidum*, *flaccidum, seaforthianum*, *sousae*, *uncinellum*

Anthers globose: *boldoense, dulcamaroides*, *seaforthianum*

Anthers tapering (buds turbinate): *dulcamara*, *uncinellum*

Mature berries > 2 cm in diameter: *dichroandrum*, *dulcamaroides*, *luculentum*, *uncinellum*, *wallacei*

Mature berries red: *boldoense*, *crispum*, *dulcamara*, *dulcamaroides*, *inodorum*, *lyratum*, *nitidum*, *pittosporifolium*, *pubigerum*, *salicifolium*, *seaforthianum*, *septemlobum*, *triquetrum*, (*uncinellum*), *valdiviense*

Mature berries orange or yellowish green: *aspersum*?, (*crispum*), *endoadenium*, *luculentum*?, (*umbelliferum*), *valdiviense*, (*wallacei*)

Mature berries black or purple: *aligerum*, *amygdalifolium*, *angustifidum*, *aureum*, *coalitum*, *cutervanum*, *dichroandrum*, *flaccidum*, *imbaburense*, *kulliwaita*, *laxum*, *leiophyllum*, *macbridei*, *muenscheri*, (*nitidum* immature), *odoriferum*, *pyrifolium*, *ruizii*, *sanchez-vegae*, *sousae*?, *stenophyllum*, *storkii*, (*umbelliferum*), (*uncinellum*), *viscosissimum*, (*wallacei*)

North America and Mexico (native or naturalised, not in cultivation): *aligerum*, *dulcamara*, *dulcamaroides*, *pubigerum*, *sousae*, *triquetrum*, *umbelliferum*, *wallacei*

Caribbean islands: *boldoense*, *pyrifolium*, *seaforthianum*

Central America: *aligerum*, *dulcamaroides*, *muenscheri*, *pubigerum*, *seaforthianum*, *storkii*

SE Brazil: *flaccidum*, *inodorum*, *laxum*, *odoriferum*, *viscosissimum*

Above treeline in Andes: *aureum*, *coalitum*, *imbaburense*, *leiophyllum*, *macbridei*, *nitidum*, *stenophyllum*

Eurasia: *dulcamara*, *lyratum*, *pittosporifolium*, *septemlobum*

Cultivated for ornament in both New and Old World (often escaped): *laxum*, *seaforthianum*

### Species descriptions

#### 1. Solanum agnoston

S.Knapp sp. nov.

urn:lsid:ipni.org:names:77128393-1

http://species-id.net/wiki/Solanum_agnoston

Figure 8

##### Diagnosis.

Differs from *Solanum sanchez-vegae* S. Knapp in bearing tufts of dendritic trichomes in the vein axils of leaf undersides and elongate, densely pubescent buds; differs from *Solanum laxum* Spreng. in having shiny upper leaf surfaces, dendritic trichomes rather than simple trichomes in the vein axils of leaf undersides, and densely pubescent buds.

##### Type.

Ecuador. Loja: km 86 de Saraguro, localidad entre Susudel y el Progreso, 3°20'S, 79°15'W, 5 Aug 1986, *J. Jaramillo* et al. *8832* (holotype: QCA [QCA183731]; isotypes: MO [MO-5203618], NY [NY00854692]).

##### Description.

Woody vine or lax shrub, 1–3 m long/tall; stems glabrous, thin and flexuous; new growth glabrous, with a few dendritic trichomes abaxially. Bark of older stems pale greyish brown, glabrous. Sympodial units plurifoliate. Leaves simple, 2–6.5 cm long, 0.8–3.5 cm wide, elliptic to narrowly elliptic, coriaceous or somewhat fleshy, the upper surfaces glabrous and shiny, occasionally with sparse simple uniseriate trichomes to 0.5 mm long along the midvein and the petiole groove, the lower surfaces sparsely pubescent with dendritic trichomes, the trichomes to 0.5 mm long, confined to tufts in the primary vein axils near the midrib; primary veins 3–4 pairs, in the type completely obscured on the upper surface, the midrib yellowish green; base obtuse to truncate; margins entire, sometimes slightly revolute; apex acute; petiole 0.5–1.5 cm long, sparsely pubescent with simple uniseriate trichomes 0.5–1.5 mm long in the groove adaxially, possibly twining. Inflorescences terminal or lateral on short shoots, 2–5 cm long, branched 2–3+ times, with 7–10 flowers, glabrous of with a few dendritic trichomes at the branching points; peduncles ca. 1 cm long or less; pedicels 1–1.2 cm long, < 0.5 mm in diameter at the base, ca. 1.5 mm in diameter at the apex, filiform, erect to nodding, glabrous, articulated at the base and inserted into a short sleeve; pedicel scars spaced 0.5–1 cm apart in clusters of 2 or 3. Buds narrowly ellipsoid, the corolla strongly exserted from from calyx before anthesis. Flowers all perfect, 5-merous. Calyx tube 1–1.5 mmm long, conical, the lobes 0.5–1.5 mm long, triangular and keeled, scarious and transparent, glabrous, but with a tuft of simple uniseriate trichomes < 0.5 mm long at the apex. Corolla known only from buds, ca. 2 cm in diameter, violet, stellate, lobed ca. 3/4 of the way to the base, the lobes not known from mature flowers, densely pubescent abaxially with weak, simple uniseriate trichomes on exposed bud surfaces, glabrous adaxially. Filaments with the tube ca. 0.5 mm long, the free portions minute, glabrous; anthers ca. 5 mm long, 1–1.5 mm wide, ellipsoid, sagittate at the base, minutely pubescent within between the sutures. Ovary glabrous; style and stigma not seen. Fruit a globose berry, ca. 1 cm in diameter, purple or violet when ripe, the pericarp thin and somewhat shiny; fruiting pedicels 1.8–2 cm long, ca. 1 mm in diameter the base, slightly expanded at the apex, pendent. Seeds ca. 5.5 mm long, 5 mm wide, flattened reniform, pale brown, the surfaces minutely pitted, the testal cells pentagonal in outline. Chromosome number not known.

**Figure 8.**
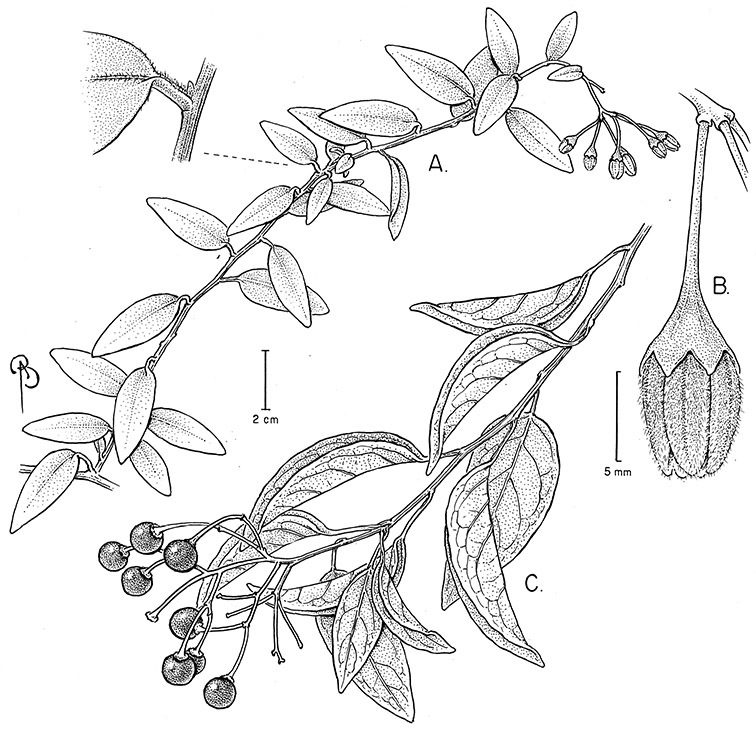
*Solanum agnoston* S.Knapp. (**A, B** drawn from *Holm-Nielsen et al. 5115* **C** drawn from *Jaramillo et al. 8832*). Illustration by Bobbi Angell.

##### Etymology.

The specific epithet comes from the Greek “agnostos”, meaning unknown, referring to the paucity of specimens and information on this species.

##### Distribution

**Figure 9.**
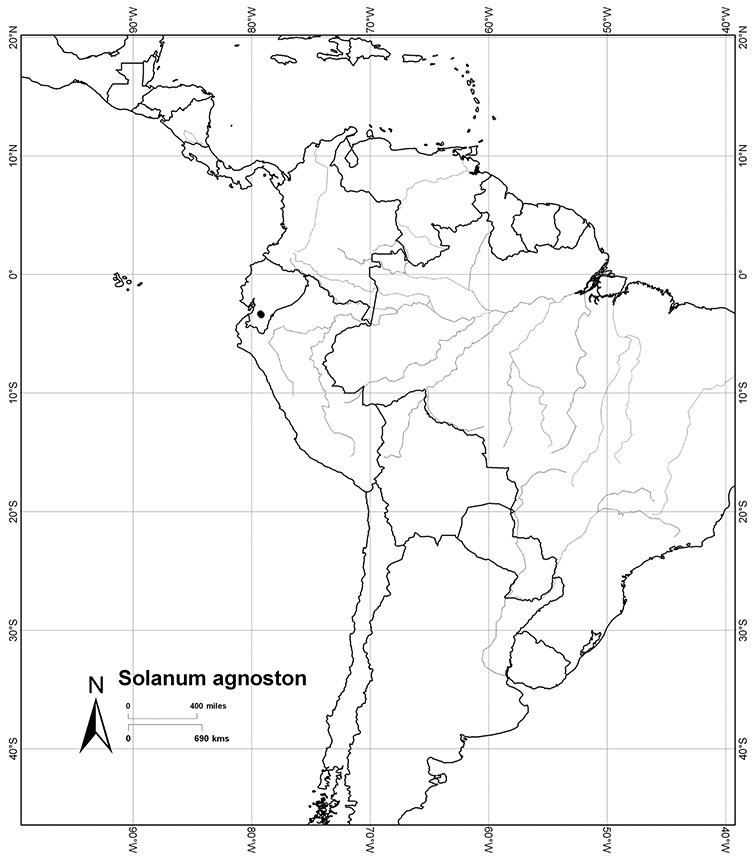
Distribution of *Solanum agnoston* S.Knapp

(Figure 9). Southern Ecuador in the provinces of Loja and Azuay, between Loja and Cuenca.

##### Ecology.

Growing in dry scrub and high elevation forests in interAndean valleys at near 3000 m elevation.

##### Conservation status.

Data deficient (DD); known from only two specimens, assessment not possible.

##### Discussion.

*Solanum agnoston* is known from only two localities, both along the main highway from Loja to Cuenca in southern Ecuador. It superficially resembles *Solanum laxum*, a species of southern Brazil that is widely cultivated in tropical and subtropical environments, but differs from it in the dendritic trichomes of the lower leaf surfaces and the elliptic, pubescent buds. The leaves of the paratype specimen (*Holm-Nielson et al. 5115*) are thick and very shiny above, such that the venation of the upper surface is not visible; the leaves of *Jaramillo et al. 8832* are not as shiny, but match in all other aspects. Label data on both specimens indicate the flowers are violet, but both duplicates of *Jaramillo et al. 8832* I have been able to find are in fruit.

*Jaramillo et al. 8832* is the only collection of this species with duplicates in Ecuadorian herbaria, and have therefore chosen this specimen as the type so the holotype is in an Ecuadorian institution.

##### Specimens examined.

Ecuador. Azuay: km 91 of Pan-American Highway N of Loja, 2900 m, 3°25'S, 79°10'W, 5 May 1973, *L. Holm-Nielson et al. 5115* (S [S-11-33845]).

#### 2. Solanum aligerum

Schltdl., Linnaea 19: 301. 1847.

http://species-id.net/wiki/Solanum_aligerum

Figure 10

Solanum pterocladum van Heurck & Müll.-Arg., Observ. Bot. 44. 1870. Type: Bolivia. La Paz: Prov. Larecaja, Sorata, 3000 m, May 1858, *G. Mandon 415* (lectotype, designated here: G [G00016904]; isolectotypes: AWH [n.v.], F [F-760412, F-876198, F-1588536], G-DC [Morton neg. 8550], GH [GH00077740], BM [BM000815930], K [K000590235], NY [NY00172146], P [P00445070, P00445071, P00445072], S [S04-2969]).
Solanum manicatum Bitter, Bot. Jahrb. Syst. 50, Beibl. 111: 63. 1913. Type: Peru. Ayacucho: Prov. Huanta, road to Tambo above Osno, Río Apurímac, 2600-2700 m, *A. Weberbauer 5643* (holotype: B [F neg. 2620], destroyed; lectotype, designated here: MOL; isolectotypes: F [F-647977, frag.], G [G00016963], GH [GH00077711]).
Solanum dotanum C.V. Morton & Standl., Publ. Field Mus. Nat. Hist., Bot. Ser. 18: 1079. 1938. Type: Costa Rica. San José: Laguna de La Chonta, NE of Santa María de Dota, 2000-2100 m, 18 Dec 1925, *P. Standley 42265* (holotype: US [US-1252694]).
Solanum grossum C.V. Morton, Revis. Argentine Sp. Solanum 178. 1976. Type: Argentina. Tucumán: Dpto. Chicligasta, Estancia Las Pavas, 2000 m, 22 Nov 1926, *Solanum Venturi 4632* (holotype: US [US-1548932, barcode US0027006]; isotypes: F, GH [GH00077675, GH00077676], NY [NY00172009, NY0017210], SI, US [US-1343321, barcode US01014178]).

##### Type

. Mexico. Michoacán: Angangueo, Oct, *C. Schiede* s.n. (holotype: B, destroyed, no duplicates found). Mexico. Michoácan: Wooded slopes 8–10 miles NW and WNW of Ciudad Hidalgo, among mountains west of Cerro San Andrés and 6-7 miles N of village of San Pedro Aguaro, in steep ravine along brook, 2850-3000 m, 19 48'N, 100 40'W, 18 Mar 1949, *R. McVaugh & R.L. Wilbur 9917* (neotype, designated here: MEXU [MEXU-92021]; isoneotypes: BM [BM000578989], NY [NY00961953], US [US-2452307]).

##### Description.

Small tree or shrub, 1.5–5 m tall, the branches arching. Stems glabrous to densely pubescent with simple or dendritic uniseriate trichomes 0.5–1 mm long, glabrescent, often strongly winged from the decurrent leaf bases, the wings to 0.5 cm wide; new growth pubescent with simple or dendritic uniseriate trichomes 0.5–1 mm. Bark of older stems dark reddish brown, shiny. Sympodial units plurifoliate. Leaves simple, (3.5-)6–15 cm long, 1.2–5 cm wide, narrowly elliptic to lanceolate, membranous to slightly fleshy, the upper surfaces glabrous or sparsely (to occasionally densely) pubescent with simple or less often dendritic trichomes on the veins and lamina, the lower surfaces with dense tufts of dendritic trichomes 0.5–1 mm long in the vein axils, these trichomes occasionally extending along the midrib or to the lamina and the leaves more densely pubescent; primary veins 10–15(-20) pairs, the midrib keeled above, the veins often drying yellowish brown; base attenuate, often winged onto the stem; margins entire, sometimes revolute; apex acuminate; petioles 0.7–2 cm long, glabrous or pubescent like the stems, never twining. Inflorescences terminal or lateral, 4–15 cm long, many times branched, with 10–60 flowers, glabrous to pubescent, the trichomes simple or dendritic, ca. 0.5 mm long, denser at branch tips and at flower insertion points; peduncle 1.5–3.5 cm long; pedicels 1–1.2 cm long, ca. 0.5 mm in diameter at the base, ca. 1 mm in diameter at the apex, slender, erect to nodding, glabrous, articulated at the base and inserted into a short sleeve; pedicel scars mostly clustered at the tips of inflorescence branches on small platforms or irregularly spaced 2–5 mm apart more basally. Buds pointed ovoid or turbinate with an apical nipple, the corolla strongly exserted from the calyx tube before anthesis. Flowers all perfect, 5-merous. Calyx tube 1.5–2.5 mm long, cup-shaped, narrowing abruptly to the pedicel, the lobes 1.5–2 mm long, quadrate with an apiculate tip to 0.5 mm long, the tips densely pubescent with simple uniseriate trichomes, these extending adaxially, the adaxial surface densely papillate. Corolla 1.8–2 cm in diameter, white, often tinged with purple, stellate, lobed 2/3 to 3/4 of the way to the base, the lobes ca. 7 mm long, 5 mm wide, spreading, minutely pubescent abaxially, the tips cucullate, densely pubescent, the sinuses broad and thin, glabrous adaxially. Filament tube minute, the free portion of the filaments 0.5–1 mm long, glabrous; anthers 4.5–5.5 mm long, 1–1.5 mm wide, ellipsoid, loosely connivent, poricidal at the tips, the pores lengthening to slits with age. Ovary glabrous; style 6.5–7 mm long, glabrous; stigma minutely capitate to slightly clavate, the surface minutely papillose. Fruit a globose berry, 1–1.2 cm in diameter, black or greenish black when ripe, the pericarp thin and shiny, glabrous; fruiting pedicels 1.5–1.7 cm long, ca. 1.5 mm in diameter at the base, woody, more or less nodding with the weight of fruits. Seeds 20–40 per berry, 2.5–3 mm long, 2–2.5 mm wide, flattened reniform, reddish brown or pale yellow, the surfaces minutely pitted, the testal cells square, the cell walls disintegrating and the seeds appearing hairy. Chromosome number: not known.

**Figure 10.**
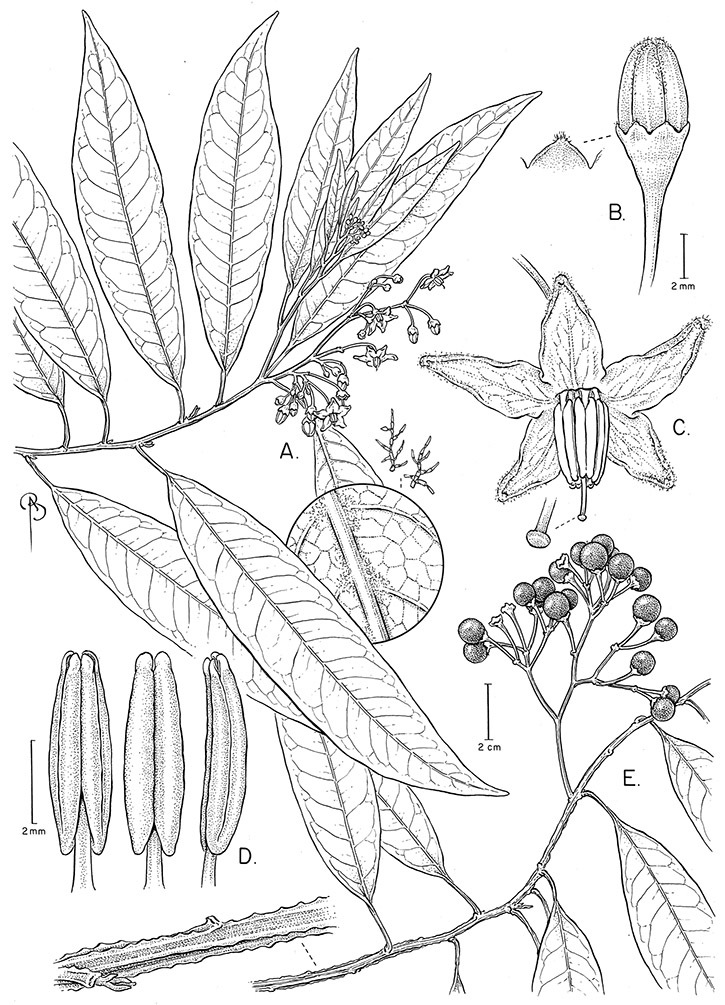
*Solanum aligerum* Schltdl. (**A** drawn from *Martinez S. 4741* **B–D** drawn from *Torres B. 2022* **E** drawn from *Nee 26870*). Illustration by Bobbi Angell.

##### Distribution

**Figure 11.**
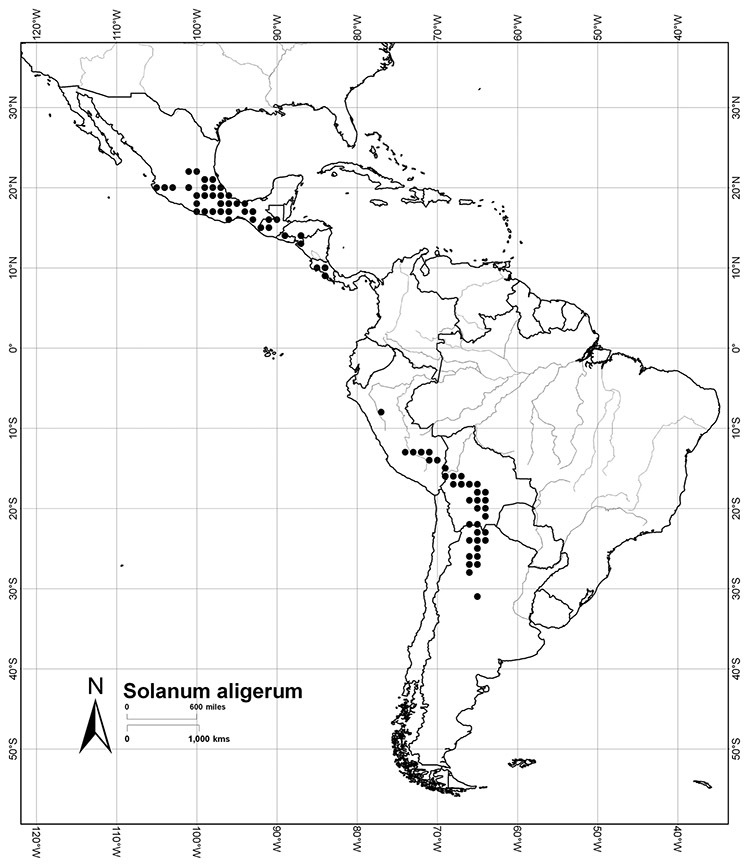
Distribution of *Solanum aligerum* Schltdl.

(Figure 11). Common from Mexico to Argentina, from 1500–3300 m. A gap in the distribution of *Solanum aligerum* occurs from Panama to Peru (see discussion).

##### Ecology.

*Solanum aligerum* has been collected most commonly in pine-oak forests in Mexico and adjacent Central America, and in cloud forests in the Andes.

##### Common names.

Guatemala: seconillo (*Steyermark 34672*).

##### Conservation status.

Least Concern (LC); EOO >50,000 km^2^ (LC) and AOO >5,000 km^2^ (LC). See Moat (2007) for explanation of measurements.

##### Discussion.

*Solanum aligerum* has the widest latitudinal range of all the New World members of the Dulcamaroid clade, occurring all down the cordilleras of both continents, but with a distinct gap between western Panama and Peru (see below). In spite of this huge range, the species is remarkably uniform in general aspect, but varies considerably in pubescence. In Bolivia, several populations have been found with plants that are uniformly and densely pubescent with dendritic trichomes over all plant parts; at one time I thought these were a new species and annotated some material as such, but in studying the Dulcamaroid clade in detail it has become apparent that these plants are merely pubescence extremes in this widespread species. They agree with other Bolivian material of *Solanum aligerum* in all other respects. It would be interesting to investigate if this pubescence variation is due to some habitat variation not apparent from herbarium labels.

*Solanum aligerum* is extremely similar to *Solanum pubigerum*, with which it broadly overlaps in central Mexico and Central America. The two species can be very difficult to distinguish, but *Solanum aligerum* has dendritic trichomes concentrated in the vein axils or over the entire lamina, while *Solanum pubigerum* has simple trichomes found all along the midrib on the leaf undersides. Flowers are larger in *Solanum aligerum* with the calyx lobes quadrate rather than deltate (this can be difficult to see), and the berries of *Solanum aligerum* are also larger and usually black or blackish green, rather than red, when ripe. The stems of S. *aligerum* are often (but not always) prominently winged, while those of *Solanum pubigerum* are never so. In general the two species appear to not occupy the same forest types where their ranges overlap.

The absence of *Solanum aligerum* in Colombia and Ecuador could indicate the northern and southern populations are in the process of differentation. This disjunction is unusual, but has also been found in the potato species *Solanum morelliforme* Bitter that is disjunct between Guatemala and Bolivia (Simon et al. 2011). *Solanum morelliforme* populations from south of the disjunction were morphologically (as here in *Solanum aligerum*) and molecularly identical. Without the locality being revealed, it is impossible to distinguish a plant of *Solanum aligerum* as coming from any particular area, so I am confident that these all represent a single taxon. Molecular studies at the population and geographical level may reveal some differentiating characters.

The holotype of *Solanum aligerum* was destroyed in Berlin and no duplicates have been traced. I have used a widely distributed collection (*McVaugh & Wilbur 9917*) from the same region of Mexico as a neotype; this typification also honors Rogers McVaugh, whose many years of collecting and study have so improved our knowledge of the flora of Mexico. Many syntypes exist for *Solanum pterocladum*, as Mandon’s collections were widely distributed and no herbarium was cited in the original publication. I have selected that in G as a lectotype, as that is where Mueller worked.

##### Specimens examined.

**Argentina**. **Catamarca**: Andalgalá, sobre la ruta no. 65, muy cerca del limite ca. de Tucumán, 4 Nov 1967, *Hunziker 19509* (CORD); Andalgalá, Esquina Grande, 29 Apr 1915, *Jorgensen 88791* (SI, US); Andalgalá, Valle del Río Chacra, 1650 m, 12 Feb 1950, *Sleumer 18* (B). **Jujuy**: Ledesma, Abra de las Cañas, Parque Nacional Calilegua, 1750 m, 27 Nov 2004, *Barboza et al. 1070* (CORD); Ledesma, Abra Colorado, 23 km de la entrada del Parque Nacional Calilegua, 16 Feb 2012, *Barboza et al. 3549* (BM, CORD); Valle Grande, camino a Valle Grande, Río Jordan, 1400 m, 18 Oct 1964, *Cabrera & Fabris 16142* (SI); Capital, Lagunas de Yala, 11 Nov 1978, *Cabrera et al. 29778* (MO, SI); Capital, Sierra de Zapia, Mina 9 de octubre, camino a la antena, 16 Nov 1980, *Cabrera et al. 32061* (SI); Santa Bárbara, S de El Fuerte, Finca Confines, 17 Nov 1992, *Kiesling et al. 8332* (MO, SI); Palpalá, above Quebrada “Los Tomates”, on road to Cerro Zapla, 1850 m, 13 Apr 2000, *Nee et al. 50757* (NY); Santa Bárbara, Centinela, 11 Dec 1983, *Rotman et al. 930* (SI). **Salta**: Parque Nacional El Rey, Cerro Chañar, 20 Nov 1981, *Brown et al. 1638* (NY); Cafayate,1400 m, 24 Nov 1949, *Araque M. & Barkley 19Ar 353* (F); Santa Victoria, Los Toldos, Rio Toldos, frente a Quebrada del Astillero, 3-5 km al E del pueblo, 30 Oct 1987, *Novara 7100* (CORD, G); Santa Victoria, Los Toldos, cerros al E del pueblo, margen S del Río Toldos, 1700 m, 2 Nov 1989, *Novara et al. 9107* (G); Santa Victoria, Camino al Lipeo, a 15 km de Los Toldos, 1800 m, 8 Nov 1975, *Schiavone et al. 11901 C*. (F, US); Capital, Quebrada de San Lorenzo, Oct 1926, *Venturi 5080* (NY, US); Guachipas, Alemania, 1500 m, 16 Dec 1929, *Venturi 9934* (US). **Tucumán**: Tafí, Apeadero Muñoz, 13 Feb 2012, *Barboza et al. 3498* (BM, CORD); Trancas, Quebrada de Rearte, 1800 m, 15 Jan 1946, *Bellonio 253* (LE, W); Cuesta del Clavillo, algo más abajo de La Banderita, 1750 m, 10 Nov 1952, *Hunziker 10005* (CORD); Monteros, camino a Tafí del Valle, km 41-42, 12 Feb 1986, *Hunziker 24873* (CORD); Chichigasta, Las Pavas, Oct 1911, *Jorgensen 36423* (SI); Chichigasta, Cuesta Rio Cochuna, 1200 m, 28 Jan 1937, *O’Donell 18158* (F); Chichigasta, Las Lenguas, cuesta del Rio Cochuma, 1400 m, 3 Nov 1930, *Schreiter 6470* (B, SI, US); Tafí, Garabatal, Siambón, 1600 m, 25 Nov 1930, *Schreiter 6552* (SI, US); Tafí, La Hoyada, Casa Arce, 1460 m, Nov 1932, *Schreiter 8812* (CORD, GH); Tafí, Cumbre de Garabatal (Porteruelo), 1750 m, 23 Oct 1939, *Schreiter 9809* (F, GH); Burruyacú, Cerro del Campo, 1800 m, 14 Dec 1928, *Venturi 7755* (US); Tafí, cerca de Taficillo, 1200 m, 5 Oct 1929, *Venturi 9609* (LE, NY, US).

**Bolivia**. **Chuquisaca**: Punilla, Guerrabamba, 3200 m, Feb 1949, *Cárdenas 4136* (US); Azurday, La Angostura, ca. 15 km de Azurduy hacia el cañon de la Angostura, 2500 m, 11 Jan 2004, *Huaylla & Guachalla 676* (K); Azurday, Tarvita, 2 km SW on Tarabuco-Azurday road, 2750 m, 25 Sep 1991, *Kessler 3235* (GOET); Oropeza, entre el km 11 y Ravelo, 3300 m, 19 Nov 1993, *Kiesling & Metzing 8425* (NY); Oropeza, Maragua, cercanias de Sucre, 3062 m, 4 Aug 2007, *Velayos et al. 11136* (MA); ca. 8 km from Punilla towards Ravelo, 3300 m, 2 Oct 1994, *Wood 8708* (K, NY); Oropeza, 2 km de la cruce a Potolo y Punilla, 2994 m, 29 Mar 2005, *Wood et al. 22011* (K). **Cochabamba**: Carrasco, Montepunco, entrando por los Yungas, 2700 m, 18 Oct 1984, *Beck 8931* (F, NY); Carrasco, ca. 75.5 km E of Epizana on Carretera Fundamental 4, 2439 m, 21 Nov 1976, *Davidson 5095* (MO, US); Carrasco, Serrania Siberia, 20-35 km W of Comarapa (Prov. Santa Cruz) on the old Cochabamba-Santa Cruz Road (Hwy 4), 2000 m, 14 Jan 1990, *Dorr & Barnett 7009* (MO, NY, US); Carrasco, Aguarrica, a 43 km de Epizana por la carretera que conduce a Santa Cruz, cerca de Aguarrica, 3030 m, 26 Dec 1982, *Fernández Casas 7793* (MA, NY); Ayopaya, 10 km Cocapata-Cotacajes, 2750 m, 8 May 1997, *Kessler et al. 9340* (NY); Carrasco, 5 km (by air and road) SE of bridge at Lopez Mendoza, 19 km by road NW of Epizana, on road from Comaropa to Cochabamba, 2900 m, 11 Feb 1987, *Nee & Solomon 34093* (CORD, MO, NY, US); Carrasco, 5 km (by air and road) SE of bridge at Lopez Mendoza, 19 km by road NW of Epizana, on road from Comaropa to Cochabamba, 2900 m, 11 Feb 1987, *Nee & Solomon 34093* (NY); Carrasco, narrow canyon of Rio Monte Puncu, 5 km NE of Monte Puncu, 10 km (by air) NW of Epizana, 2700 m, 10 Mar 1988, *Nee & Solomon 36607* (CORD, NY, US); Chapare, trail leading NW from the road to tablas Monte along Río Corani Mayu, 2280 m, 2 May 1994, *Ritter 919* (GH, MO); Chapare, 23.8 km N of Colomi (junction of the road to Candelaria) on road to Chapare, then 2.2 km NW (left) on side road. Upper Rio Cayani, 2700 m, 19 Oct 1985, *Solomon 14386* (G, MO, NY); Chapare, km 104 del camino a Chapare, 3100 m, 2 Dec 1966, *Steinbach 563* (F, MO, NY, S, US); Carrasco, Totora-Duraznillo, 2700 m, 30 Mar 1920, *Steinbach 3826* (NY); Chapare, Sacaba, Cerro de Incachaca, 2500 m, 4 Sep 1921, *Steinbach 5752* (F, G, MO, NY); Pojos, 2600 m, 5 Nov 1928, *Steinbach 8621* (BM, E, MO, NY, S); Totora-Pocona, 2700 m, 7 Nov 1928, *Steinbach 8685* (BM); Chapare, Incachaca-La Aduana (Sanctutonis), 2700 m, 8 Mar 1929, *Steinbach 9550* (BM, E, F, G, MO, NY, S); Chapare, Durazno, Quebrada de Coraní, 2800 m, 18 Jun 1928, *Steinbach 9854* (BM, E, F, G, MO, NY, S); Ayopaya, Independencia, c. 1 km before La Mina, 38 km north of Independencia along road to Sailapata, 2856 m, 15 May 2002, *Wood et al. 8479* (BOLV, K); Chapare, descent from the dam towards Villa Tunari on road from Cochabamba to the Chapare, 3000 m, 25 Jun 1994, *Wood 8545* (K, LPB). **La Paz**: Larecaja, Laripata, 2995 m, 13 Aug 2007, *Aedo et al. 14644* (MA); Larecaja, Sorata, 2439 m, Feb 1886, *Rusby 781* (NY); Murillo, Zongo, 2760 m, 15 Aug 2007, *Aedo et al. 14734* (MA); Inquisivi, Rio Calachaca Jahuira-down river 1 km from Aguas Calientes de Calachaca, 10 km NE of Choquetanga, 3200 m, 17 Jul 1991, *Lewis 39251* (G, MO); Larecaja, along road to Consata, ca. 4-15 km above (N of) Sorata, 2896 m, 24 May 1990, *Luteyn & Dorr 13794* (NY, NY, US); Sud Yungas, La Paz Calacoto 69 km hacia el Este, pasando el Nevado Illimani, Estacion general Ikiko, 3100 m, 31 Dec 1980, *Beck 3904* (F); Inquisivi, 3 km (by air) and 8 km (by road) SW of Inquisivi, above road from Quime to Inquisivi, 2950 m, 12 Mar 1988, *Nee & Solomon 36672* (CORD, F, G, MEXU, MO, NY, US); Inquisivi, 6.5 km SE of Inquisivi, 1.2 km SW of Machacamarca, 3000 m, 18 Mar 1988, *Nee 36718* (CORD, MO, NY, US); Larecaja, above Rio Lakhathiya, just above junction with Rio Tusca Jahuira, 19 May 2001, *Nee et al. 51833* (NY); Inquisivi, comunidada Coquetanga-Aguas Calientes-Calachaca, cuenca del rio Calachaca-Jahura, 9 km de Choquetanga, 3400 m, 20 Jun 1994, *Salinas 3275* (K, NY); Sud Yungas, Mina Chojilla camino de acceso de vehiculos a Kacapi, 2700 m, 4 Jul 2000, *Siñani RS 230* (NY); Pedro Domingo Murillo, Rio Zongo Valley, 22.5 km below dam at Lago Zongo, 3000 m, 9 Oct 1982, *Solomon 8431* (K, MO, NY); Inquisivi, Camino de Quime a La Paz a unos 2–3 km arriba de Quime, 3221 m, 16 Mar 2003, *Wood & Ortuño 19411* (K, LPB). **Santa Cruz**: Florida, Comarapa Road, 28 km from Comarapa, 2650 m, 15 Aug 1991, *Acevedo et al. 4623* (NY, US); Caballero, Siberia, 2–2.5 km debajo de la escuela de Siberia, camino al valle de Saipina, 2850 m, 30 Nov 2003, *Jordan & Vargas 253* (MO); Vallegrande, on road from Guadalupe to Pucará, 2725 m, 4 May 2001, *Nee et al. 51736* (BM, NY); Caballero, loc 2-3 km S de Siberia sobre el camino a Larkapampa, 3000 m, 25 Jul 1996, *Saldías & Fernández 4607* (NY); Caballero, Parque Nacional Amboró, Siberia, 25 km al NW sobre la carretera Comarapa-Cochabamba, 3000 m, 5 May 1993, *Vargas C. et al. 2302* (NY); Caballero, Parque Nacional Amboró, proximadades de Cerro Bravo a 10 km al N de Comarapa, alrededores de la Parcela permanente, 2400 m, 18 Oct 1993, *Vargas C. & Jardim 3002* (NY); Caballero, Siberia, ca. 1–2 km arriba de la comunidad de Siberia, sobre un camino vecinal, entrando hacia el Parque Nacional Amboró, 3001 m, 12 Apr 2003, *Wood et al. 19700* (BOLV, K);. **Tarija**: route Villa Montes to Tarija, 15 km apres Entre Rios, 1850 m, 4 Nov 1993, *Billiet & Jadin 6067* (K, MO); Aniceto Arce, 39.9 km S of jct of road to Entre Rios, on road to Padcaya, 2100 m, 29 Apr 1983, *Solomon 10249* (MO, NY).

**Costa Rica**. **Cartago**: Volcán Irazu and Volcán Turrialba, along road from Pacayas to Hacienda Central, 31 Mar 1982, *Barringer et al. 2233* (F); SE slope of Cerro de la Muerte, Cordillera del Talamanca, along Interamerican Hwy 63 miles from downtown San Jose, 2700 m, 23 May 1976, *Croat 35427* (MO); Cantón Paraíso, Reserva Forestal Los Santos, Cueva del Savegre, carretera Interamericana de la Georgina hasta la entrada a San Gerardo de Dota, 3180 m, 18 Mar 1997, *González & Hammel 1840* (G, MO); Cantón de El Guarco, Cordillera de Talamanca. Carretera Interamericana, entre E1 Empalme y La Chonta, 2400 m, 10 May 1995, *Hammel 19815* (BM, MO); Irazú, 2134 m, 24 Jun 1974, *Kuntze 2300* (NY); Macho Gap Camp, 39 kilometers S. of Cartago and 3 miles N of Copey, 2500 m, 19 Feb 1943, *Little 6022* (F, US);. **Heredia**: Cantón de Barva, P.N. Braulio Carrillo, Cordillera Central, potreros La Georgina, 2600 m, 29 Oct 1993, *Fernández & García 1503* (MO); **Puntarenas**: Monteverde, at edge of Continental Divide and of Pacific side of slope, 1300 m, 18 Aug 1976, *Solomon 5365* (MO); N. San Isidro del General, 12 Aug 1971, *Spellman et al. 653* (MO); **San José**: northern Cordillera Talamanca, region of Cerro de la Muerte, on carretera Nacional 34.5 km N. of San Isidro and 2.6 km S of La Georgina Inn, 2652 m, 4 Apr 1978, *Davidson 7235* (F, MO, NY); Cantón de Pérez Zeledón, Cordillera de Talamanca. Estación Cuericí, 2600 m, 29 Aug 1995, *Gamboa 281* (BM, MO); Villa Mills, Cerro de la Muerte, Carretera Panamericana Sur, 2800 m, 26 Feb 1965, *Jiménez M. 2989* (F, NY); Z.P. Cerros de Escazú, Cedral, falda norte del cerro Rabo de Mico, cuenca del Río Poás, 1600 m, 9 Oct 1991, *Morales 168* (MO); Santa María de Dota, Laguna de la Chonta, northeast of Santa María, 2000 m, 18 Dec 1925, *Standley 42265* (F, US); near Finca La Cima, above Los Lotes, North of El Copey, 2100 m, 21 Dec 1925, *Standley 42764* (US); San Gerardo de Dota, 8 to 10 km down from the Interamerican Highway, 18 Sep 1975, *Utley & Utley 3083* (F, MEXU, MO, NY).

**El Salvador**. **Santa Ana**: Near summit of Cerro Monte Cristo, 2134 m, 18 Jan 1959, *Allen 7172* (F, NY, US); Montecristo, P.N. Montecristo, plan de los helechos, 2051 m, 13 Mar 2002, *Carballo et al. 281* (LAGU); Cordillera Miramundo, mountain of Montecristo, 2000 m, 27 Jan 1966, *Molina R. et al. 16706* (F, NY, US); P.N. Montecristo, al final del atajo para el Trifinio, 2000 m, 24 Jan 2002, *Monterrosa et al. 215* (B, BM, LAGU).

**Guatemala**. **Chimaltenango**: Chichavae, 2400 m, 5 Dec 1933, *Skutch 738* (US). **Huehuetenango**: San Juan Ixcoy, Sierra de los Cuchumatanes, along road to Huehuetenango, 5 miles S of San Juan Ixcoy, 4 Feb 1965, *Breedlove 8542* (F); San Mateo Ixtatán, Sierra de los Cuchumatanes, 4 miles east of San Mateo Ixtatan on road to Barillas, 7 Feb 1965, *Breedlove 8744* (F); Cerro Cananá, between Nucapuxlac and Cananá, Sierra de los Cuchumatanes, 2500 m, 18 Jul 1942, *Steyermark 49046* (F); wet cloud forest at Cruz de Limon, between San Mateo Ixtatan and Nuca’, Sierra de los Cuchamatanes, 2600 m, 31 Jul 1942, *Steyermark 49870* (F, NY). **Quetzaltenango**: highway km 172 junction Quetzaltenango, Huehuetenango and Totonicapan, 10 Jan 1974, *Molina R. et al. 30200* (F, MO); Volcán Zunil, 2500 m, 22 Jan 1940, *Steyermark 34672* (F). **Quiché**: sin. loc., 1942, *Aguilar* s.n. (F). **Totonicapán**: Sierra Madre Mountains south of Totonicapán, near Mirador (km 170), 2800 m, 20 Dec 1972, *Williams et al. 41447* (BM). **Zacapa**: Jones, 2020 m, 5 Apr 2000, *CDC-CECON 1535* (BM).

**Honduras**. **Francisco Morazán**: along road to Parque Nacional La Tigra, 22-25 km NE of Tegucigalpa, 1850 m, 1 Feb 1987, *Croat & Hannon 64047A* (MO, NY); Tegucigalpa, 11 km NE of Tegucigalpa. La Tigra National Park Summit, 2000 m, 26 May 1992, *D’Arcy 18006* (MO); La Tigra, 25 km NE de Tegucigalpa, 2105 m, 24 May 1986, *Manzano 177* (MA); Montaña La Tigra S.O. de San Juancito, 2000 m, 23 Apr 1964, *Molina R. 13744* (F, NY, US); Cerro Uyuca, 8 km O. del Zamorano, 1100 m, 27 Apr 1984, *Soihet M. 142* (MO).

**Mexico**. **Chiapas**: Chamula, Yalal Chin, 1829 m, 3 Apr 1965, *Breedlove 9540* (MEXU); San Cristóbal de las Casas, SW slope of Zontehuiz, 2774 m, 21 Jun 1965, *Breedlove 10435* (F, MEXU, US); Chenalho, in paraje Los Angeles Chiste, 1700 m, 22 Oct 1976, *Breedlove 40863* (MEXU, MO); La Independencia, 6-10 km north-northeast of La Soledad along logging road from Las Margaritas to Campo Alegre, 1600 m, 26 Nov 1980, *Breedlove & Almeda 47819* (MEXU, MO, NY); Zinacantán, on NW side of Muk’ta vits (Cerro Huitepec), 2743 m, 18 Feb 1966, *Laughlin 125* (MEXU, US). San Cristóbal de las Casas, Santa Cruz en San Felipe, 15 Nov 1986, *Mendez Ton 9743* (MEXU, NY); Pueblo Nuevo Solistahuacán, on ridge north of Clinica Hierba Buena near Pueblo Nuevo Solistahuacán, 1981 m, 25 Jan 1965, *Raven & Breedlove 19978* (F); Tenejapa, at Colonia Ach’lum, 2652 m, 27 Dec 1965, *Shilom Ton 454* (F, MEXU, NY, US); San Andres, NW of San Cristobal, 579 m, 26 Feb 1931, *Souviron & Erlanson 81* (BH, US); Tenejapa, a 20 km al SW de Tenejapa camino a San Cristóbal de las Casas, 25 Sep 1983, *Téllez V. & Pankhurst 7262* (MEXU, MO); Mazapa de Madero, Granada de Tlalcanaque, 10 km al Noreste, 2500 m, 22 Feb 1987, *Ventura & López 4352* (MO, NY). **Colima**: Nevado de Colima, Nevado de Zapotlan, a few miles south of Ciudad Guzman (Zapotlan), 3000 m, 2 Jul 1956, *Gregory & Eiten 292* (P); **Guerrero**: Taxco de Alarcón, Taxco. 19.9 km al NO, 2360 m, 28 May 1998, *Cruz Durán 2260* (MEXU). Chilpancingo de los Bravo, a 6 km al W de Omiltemi, camino a la Soledad La Joyas, 27 Mar 1982, *Martínez S. & Téllez V. 272* (MO); Chilpancingo, laderas superiores del Cerro Alquitran, cerca de Mazatlan, 2600 m, 11 Feb 1970, *Rzedowski 27036* (F, MO x2); Chichihualco, a 8 km al SO de Filo de Caballo, 2435 m, 21 Apr 1985, *Soto N. & Aureoles C. 8345* (MEXU). **Hidalgo**: Lolotla, carr. Pachuca-Huejutla, 3.5 km al N de Ixtlahuaco, 1670 m, 5 Apr 1986, *Baker et al. 054* (NY); 17 mi N of Jacala, ca 56 mi from Zimapan, 1 Aug 1972, *Dunn 19059* (MO); Zacualtipán, 1 km al SSE de Zacualtipan, 2400 m, 19 May 1976, *Flores F. 234* (MO, US); Zacualtipán, Zacualtipán, 1 km al SSE, 2400 m, 19 May 1976, *Flores M. 234* (CORD, MEXU); Tenango de Doria, Cirio, 8 km al este de Tenango de Doria, 1700 m, 27 Mar 1980, *Hernández M. & Hernández V. 4189* (MEXU); Tianguistengo, 10 km al oeste de Tianguistengo (Tepeoco), 2100 m, Apr 1981, *Hernández M. et al. 5806* (MEXU, MO); Metztitlán, noreste de Hidalgo, Rincón de los Ahuajes, 3.5 km de la desviación a Zoquizoquipan, 6 Jul 1992, *López García 113* (MEXU); Zacualtipán, Paraje Cumbre de Tlahuelompa, 2 km al SW del ejido de Tlahuelompa, 18 Dec 1992, *López García 392* (MEXU); El Alcalaque, 2 km al NW del pobaldo Eloxochitlán, 29 Dec 1992, *López G. 433* (MEXU); Tlanchinol, a 4 km al E de Tlanchinol, camino a Apantlasol, 3 Sep 1997, *Martínez S. 28444* (MEXU, NY); Palo Hueco, km 301 on highway between Jacala and Santa Ana, 1524 m, 28 Apr 1947, *Moore 2672* (BH); Barranca below Trinidad Iron Works, 1524 m, 10 May 1904, *Pringle 8835* (BH, BM, F, GOET, K, LE, MEXU, MO, US); Molango, Ismolinta, 2 km S de Ismolinta rumbo a Eloxochitlán, 1580 m, 19 Jun 1995, *Sousa Peña 577* (MEXU); Zacualtipán, vereda entre El Reparo y Zahuastipan, 3 km de la Majonera, 29 Jun 1988, *Vázquez et al. 4626* (F, NY); Zoquizoquipan, 1940 m, 2 Sep 1962, *Vela G. 977* (MEXU). **Jalisco**: Yolox, Sierra de Juárez, Carretera Ciudad de Oaxaca. a Tuxtepec, cerca de Cerro Pelon, a 300 m. antes de San Pedro Yolox, 2850 m, 18 Jan 1989, *Chazaro et al. 5815* (MEXU); Cuatitlán, 19-20 km al NE de Cuatitlan, 500 m al W de Llanos de San Miguel, 2200 m, 29 Apr 1988, *Cuevas & Guzmán 2871* (MEXU); along lumber road, 0.3 km E of fork Cerro La Cumbre/Rincón de Manantlán, at crossing of first small stream, N of Sierra de Manantlán Central, 17.7 km S of El Chante, 2250 m, 6 Jan 1980, *Iltis et al. 2336* (F x2, MEXU, US); northwestern slopes of Nevado de Colima, above Jazmín, 2-3 km above settlement of El Isote, 2600 m, 26 Mar 1949, *McVaugh & Wilbur 10066* (BM); Sierra de Manantlán (15-20 miles southest of Autlán), about 2 miles from Aserradero San Miguel Uno, west and south of divide towards Manzanillo, 2250 m, 4 Nov 1952, *McVaugh & Sooby 13923* (BM); Venustiano Carranza, Puerto El Floripondio, camino a la Est. de Microondas Las Viboras, 2450 m, 22 Aug 1987, *Rodríguez C. & Suárez J. 939* (MEXU). **Michoacán**: Zinapécuaro, Los Azufres, alrededores de la laguna larga, 31 May 1988, *Díaz Barriga 4680* (MEXU); Zinapécuaro, Cañada La Hierbabuena, al SW de la Presa Laguna Larga, 2750 m, 17 Nov 1988, *Jasso 532* (MEXU, MO); Melchor Ocampo, Cerro Camacho, Los Remedios, a 4 km al W de Ocampo, 2320 m, 26 Apr 1982, *Martínez S. et al. 386* (BH, MEXU); Tzitzio, En Mil Cumbres a 31 km al SW de Cd Hidalgo, carretera a Morelia, 2500 m, 11 Oct 1983, *Martínez S. et al. 4741* (MEXU, NY). **Morelos**: Tepoztlán, Santo Domingo Ocotitlán, en el cerro al N de la población, 2200 m, 26 Apr 1984, *Gutiérrez 203* (MEXU). **México**: Temasceltepec, Rincón, 3 Oct 1932, *Hinton 365* (BM, K, US); Temascaltepec, Comunidad, 2480 m, 5 Oct 1933, *Hinton 3852* (BM, F, K, MO, US); **Oaxaca**: Comaltepec, immediately to the right of Hwy. 175, just beyond first major switchback on decent from Mirador below Cerro Humo Chico, in quebrada of Río Cerro Pelón, 2740 m, 1 Nov 1993, *Boyle & Massart 2512* (BM, MEXU); Sierra Juárez, 2.5 miles by road. SW of summit at Cerro Pilon, on hwy 175 between Ixtlan de Juarez and Valle Nacional, 2550 m, 19 Jul 1972, *Breckon & Breckon 1364* (F); San Martín Peras, Dto. Juxtlahuaca, a 2.7 km adelante de la desviacion de San Martin Peras, carretera para Coycoyan de las Flores, 2490 m, 20 Jun 1993, *Calzada 18460* (MEXU, NY); Comaltepec, 46.5 km al NE de Istlán, carretera Oaxaca-Tuxtepec, 2650 m, 17 Dec 1987, *Campos V. & Torres 886* (MEXU); Mixistlán, 30 km al S de Totontepec, Distr. Mixe, 2600 m, 10 Nov 1983, *García Mendoza et al. 1287* (MO, NY); Patio de Arena, vicinity of Cerro Zempoaltepetl, ca. 5 km E of summit, 2800 m, 9 Aug 1950, *Hallberg 879* (MEXU); Ixtlán, Sierra de Juárez, parte alta y cima de Cerro Pelon, 2900 m, 5 Aug 1985, *Lorence et al. 4754* (MEXU); Yolox, distrito de Ixtlan, 8 km E of Yolox on road between Yolox and highway 175, 2900 m, 13 Apr 1981, *Martín 531* (MO); Ixtepeji, La Botuda, Dto. Ixtlán, 10 May 2001, *Santiago 49* (MEXU); Totontepec, Villa de Morelos, distr.: Mixe Tsa, 19 Aug 1991, *Rivera Reyes 2814* (MEXU, NY); Santa María Teopoxco, Sierra Mazateca, near Plan de Guadalupe, 39 km by road (16 km by air) west o Huatla on road to Teotitlan, 2200 m, 17 Jan 1984, *Solheim & Reisfield 1400* (NY); Puerto de la Soledad, 3 km al S de Huautla, sobre el camino a Teotitlán del Camino-Huautla, 10 Sep 1981, *Téllez V*. & *Elisens 4698* (MEXU); Totontepec, 13 km al O de Totontepec, carretera a Villa Alta, Dto. Mixe, 28 Mar 1986, *Torres C*. & *Ramírez 8450* (MEXU, MO, US); San Juan Tepeuxila, San Juan Tepeuxila, Dto. Cuicatlan, arroyo la primera toma, hacia Llano Chiflido, por Arroyo Paloma, 2369 m, 18 May 2002, *Torres Colin et al. 16202* (MEXU). **Puebla**: Tlatlauqui vertiente norte, 1800 m, 31 Apr 1971, *Boege 1738* (MEXU); Equimita, km 35 de la carretera que va hacia Cuetzalan, 22 Jun 1976, *Inzunza 114* (MO); Huauchinango,1951 m, 7 Oct 1944, *Sharp 441218* (MEXU, US); Coxcatlán, L y Griega, desviación a Coyomeapan, de La Brecha a Zoquitlán, 2540 m, 28 Sep 1984, *Tenorio L. & Romero de T. 7502* (MEXU); Tezuitlán, Coaxisco 12 km al NW de Tezuitlan, 2030 m, 12 Apr 1985, *Tenorio L. et al. 8630* (MEXU); Pahuatlán, Acahuales, 1900 m, 22 May 1986, *Tenorio L. & Romero de T. 11383* (MEXU); Tezuitlán, Río Frío, 12 km al N de Teziutlan, carr. a Nautla, 1500 m, 7 Jul 1987, *Tenorio L. et al. 14020* (MEXU, NY). **Querétaro**: Pinal de Amoles, El Ranchito, 1 km de El Rnachito camino a San Pedro Escanela, 1980 m, 10 Nov 1988, *Carranza 1251* (MEXU). **San Luis Potosí**: 22 km W of Santa Catarina on highway 86 at km 49, 2200 m, 29 Sep 1965, *Roe & Roe 2191* (BM). **Veracruz**: Xico, Tonalasco, 25 Jun 1986, *Arriaga C. 389* (MEXU); Huayacocotla, Vereda Tzimentey, cerca del limite SE de la Reserca propuesta, 1750 m, 25 Apr 1981, *Bastelleros & Ballesteros 421* (MEXU); Acultzingo, La Laguna, carretera Puerto del Aire, poblado, 2320 m, 16 Dec 1977, *Calzada & Delgado 4181* (MEXU); Huayacocotla, Agua de la Calabaza, carretera Huayacocotla-Chicontepec, 1800 m, 20 Jul 1979, *Calzada 5464* (F); Coatepec, Cerro Huilotepec entre Mesa de los Laureles y Tierra Grande, 2500 m, 27 Jul 1987, *Cházaro B. et al. 5275* (MEXU, NY); Las Minas, al SE de Rinconada por el Cerro La Tolva, 2300 m, 11 Aug 1988, *Duran E. & Burgos 570* (MEXU); Huayacocotla, vicinity of a large shrine and “Bienvenidos a Huayacocotla” arch over the highway, SW entrance to Huayacocotla on road from Palo Bendito, 2150 m, 21 Jul 1982, *Diggs & Nee 2958* (BH, CORD, F); Huayacocotla, Agua de la Calabaza, 16 km NE de Huayacocotla, carretera a Zilacatipan, 1750 m, 24 Apr 1981, *Juárez G. & Vasquez B. 68* (F); Huayacocotla, Xalapa, INIREB, along Huayacocotla-Zontecomatlan road, between Barro Colorado and Tepozanes, 2 km by road NE of Agua de la Calabaza and 5 km by road SW of Zilacatipan, 1800 m, 27 Apr 1983, *Nee 26894* (F, MO, NY); Puerto del Aire, just W and high above Acultzingo, top of very steep pass on Highway 150 (Tehuacán to Orizaba) near boundary between states of Veracruz and Puebla. Cumbres de Acultzingo, 2409 m, 27 Sep 1962, *Ugent & Flores C. 2466* (BM); entre Los Ocotes y Helechales, 1 Jun 1980, *Vargas et al. 306* (MEXU); Jalacingo, Ocotepec, cerca de cerro, 1750 m, 4 Mar 1970, *Ventura A. 1008* (F, MO, NY); Acajete, Acajete, 1850 m, 1 Apr 1974, *Ventura A. 9810* (MEXU, MO); Atzalán, La Florida, 1650 m, 16 Jun 1976, *Ventura A. 12871* (F, MEXU); Altotonga, San Miguel Tlalpoalan, 1950 m, 2 Jun 1981, *Ventura A. 18538* (MEXU); Acajete, La Joya, 2100 m, 1 Apr 1982, *Ventura A. 19577* (MEXU, MO); Las Vigas, Rancho San Miguel, barranca El Cable, 2400 m, 11 Sep 1989, *Zamora C. 1096* (MEXU);

**Peru**. **Cusco**: Urubamba, Ollantaytambo, Dist. Ollantaytambo, Abra de Malaga, 3600 m, 2 Jun 2002, *Galiano et al. 4225* (MO); Hacienda Huyucalla, valle del Paucartambo, 3400 m, Jul 1930, *Herrera 2986* (US); Paucartambo, 10 km from Puesto de Vigilancia (PN Manu) towards Paucartambo, 3313 m, 15 Mar 2012, *Knapp et al. 10436* (BM, USM); Quispicanchis, near Marcapata, 24 Jul 1942, *Metcalf 30729* (G, MO, US); Quispicanchis, entre Abra Walla Walla y Marcapata a 210 km de Cusco, 2800 m, 21 Apr 1988, *Núñez V. et al. 8994* (F, MO x2); Pautarcambo to Tres Cruces, Cerro de Cusilluyoc, 3200 m, 2 May 1925, *Pennell 14148* (F, NY); Calca, Dist. Lares, Mantto, 2100 m, 23 Jan 2003, *Valenzuela et al. 1236* (MO); La Convención, Espiritupampa, Dist. Vilcabamba, 3300 m, 14 Oct 2003, *Suclli et al. 1277* (BM, NY); La Convención, San Luis, valley of Lucumayu River, on road from Ollantaytambu to Quillabamba, environs of San Luis (former resturan) above Alfamayo, along roadside with 2 km of San Luis back up towards Abra de Malaga, 2800 m, 3 Apr 1997, *Tupayachi & Emshwiller 3424* (BH); Paucartambo, Challabamba, Pesto Grande, 2900 m, 20 Apr 1986, *Zimmerer 80* (NY). **Junín**: Tarma, Carpapata, al este de Carpapata, 8 Dec 2005, *Marcelo Peña et al. 1920* (MOL). **Puno**: Carabaya, Ollachea to San Goban road, 2.5 km north of Ollachea, 15 Aug 1980, *Boeke 3049* (F, MO, NY, US); Ollachea to San Gabon, 17 Jul 1978, *Dillon et al. 1107* (BM, MO, NY).

#### 3. Solanum alphonsei

Dunal, Prodr. [A.P. de Candolle] 13(1): 70. 1852, as “alphonsi”

http://species-id.net/wiki/Solanum_alphonsei

Figure 12

Solanum alphonsei Dunal var. *taguatagua* Dunal, Prodr. [A.P. de Candolle] 13(1): 70. 1852. Type: Chile. Sin. loc., *C. Bertero 635* (holotype: P [P00319395, Morton neg. 8142, G neg. 39141]).
Solanum germainii Phil., Linnaea 29: 23. 1858, as “*germaini*” Type: Chile. Región Metropolitana: Prov. Santiago, mountains near Aculeo [“Aculco”], *R.A. Philippi* s.n. (holotype: SGO [SGO-55447, Dept. Invest. Agrícolas neg. s.n.]; isotypes: B [destroyed, F neg. 2732], LE, MA [MA-533038]).
Solanum tenuicaule Phil., Anales Univ. Chile 91: 13. 1895. Type: Chile. sin. loc., *Anon*. [*R.A. Philippi?*] *s.n.* (lectotype, designated here: SGO [SGO-55451, barcode SGO000004604]).

##### Type.

Switzerland. Cultivated in Geneva, 1834, originally from Chile (lectotype, designated here: G-DC [G00144607].

##### Description.

Woody vine or lax shrub to 1 m tall. Stems erect or spreading, glabrous or with simple uniseriate glandular trichomes to 0.5 mm, these denser at the nodes, the glands 1-celled and soon deciduous; new growth glabrous to minutely glandular puberulent. Bark of older stems green to reddish brown, glabrous. Sympodial units plurifoliate. Leaves simple or more often shallowly and irregularly pinnatifid, (0.5-)1.5–5 cm long, (0.5-)1–3(-5) cm wide, narrowly elliptic to elliptic or rhomboid, membranous, occasionally slightly fleshy, both surfaces glabrous or with scattered simple uniseriate trichomes on the lamina, these denser along the margins; primary veins 4–5(-7) pairs, often drying reddish brown; base acute or truncate; margins entire or shallowly lobed in the basal half, the lobes divided less than halfway to the midrib; apex acute to more often rounded; petioles 0.5–1 cm long, glabrous to pubescent with simple uniseriate glandular trichomes, apparently twining. Inflorescences terminal or later lateral, 2–6.5 cm long, many times branched, openly divaricate, with up to 30 flowers, glabrous or pubescent with glandular simple uniseriate trichomes to 1 mm long; peduncle 0.5–3 cm long; pedicels 0.6–1 cm long, ca. 0.5 mm in diameter, slender, nodding at anthesis, glabrous, articulated at the base from a small sleeve, leaving a tiny peg on the inflorescence axis; pedicel scars irregularly spaced 2–12 mm apart. Buds globose when young, later ellipsoid, the corolla strongly exserted from the calyx before anthesis. Flowers all perfect, 5-merous. Calyx tube ca. 1.5 mm long, shallowly cup-shaped, the lobes 1–1.5 mm long, deltate with elongate tips, the sinuses splitting irregularly, glabrous or minutely glandular puberulent. Corolla 1–1.5 cm in diameter, white, stellate, lobed nearly to the base, the lobes 5–7 mm long, 3–4 mm wide, planar or slightly cupped at anthesis, densely papillate-puberulent on the tips and margins, the trichomes sometimes extending over entire abaxial surface. Filament tube minute, the free portion of the filaments ca. 0.5 mm long, glabrous; anthers ca. 3 mm long, 1.5 mm wide, ellipsoid, loosely connivent, yellow, poricidal at the tips, the pores lengthening to slits with age. Ovary glabrous; style 6–7 mm long, glabrous; stigma capitate, to ca. 0.5 mm in diameter, the surface minutely papillose. Fruit a globose berry, ca. 0.8 cm in diameter, orange or reddish orange when ripe, glabrous, the surface shiny and thin; fruiting pedicel ca. 1 cm, deflexed to spreading. Seeds 15–20 per berry, ca. 1.5 mm long, 2 mm wide, flattened reniform, pale tan, the surfaces minutely pitted, the testal cells broadly sinuate in outline. Chromosome number: not known.

**Figure 12.**
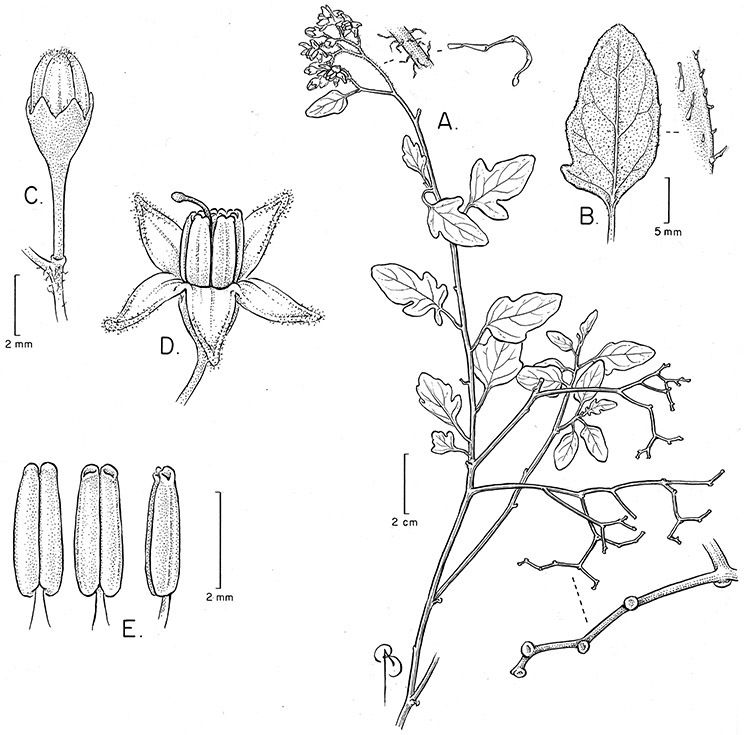
*Solanum alphonsei* Dunal. (**A–H** drawn from *Anon.* s.n., Mar 1858, BM). Illustration by Bobbi Angell.

##### Distribution

**Figure 13.**
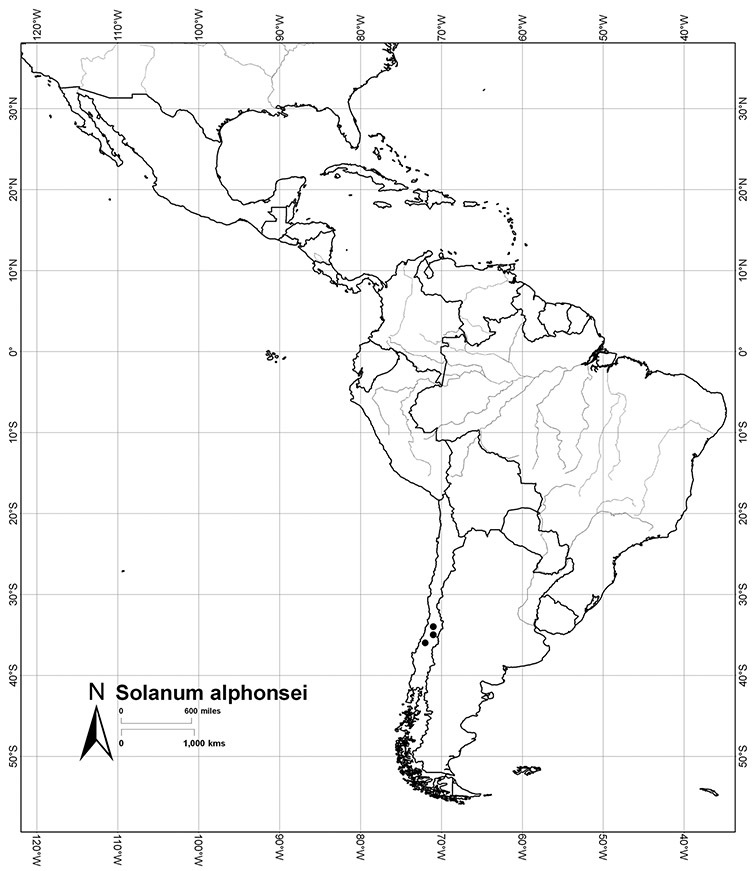
Distribution of *Solanum alphonsei* Dunal.

(Figure 13). *Solanum alphonsei* occurs in southern Chile and possibly also adjacent Argentina, from sea level to the summit of the Andes at ca. 3000 m.

##### Ecology.

In *Nothofagus* Blume (Nothofagaceae) forest and forest margins.

##### Conservation status.

Vulnerable (VU); EOO <20,000 km^2^ (EN) and AOO <2,000 km^2^ (VU). See Moat (2007) for explanation of measurements.

##### Discussion.

*Solanum alphonsei* is one of three species of the Dulcamaroid group found in the *Nothofagus* forests of southern Chile (and probably also from adjacent Argentina); the others are *Solanum valdiviense* and *Solanum crispum*. *Solanum alphonsei* differs from *Solanum valdiviense* in being a vine with non-angled stems and twining petioles, in having open and divaricately branched inflorescences and in its leaves that are rhombic or deltate in outline. The corollas of *Solanum alphonsei* are in general smaller than those of *Solanum valdiviense*, and are less deeply lobed. *Solanum crispum* is much more common than either of the other two species, and can be distinguished from *Solanum alphonsei* by its larger flowers, denser pubescence and larger inflorescences. *Solanum alphonsei* is much less commonly collected than is *Solanum valdiviense*. An extreme form of *Solanum alphonsei* with tiny leaves was given the herbarium name of “myrtilloides” by Witasek on a specimen in Vienna.

Dunal named *Solanum alphonsei* for Alphonse de Candolle, the editor of the *Prodromus* and son of his mentor from Montpellier, Agustin Pyramus de Candolle. Philippi named *Solanum germainii* after Philibert Germain, the Chilean botanist and collector. Both original spellings are correctable (McNeill et al. 2012).

No specimens were cited in the original description of *Solanum alphonsei*, but the material was said to have been cultivated in Geneva in 1834. The specimen in G-DC [G00144607] is dated 1834, and is chosen here as the lectotype. Other sheets in P, G, G-DC and BM from plants cultivated in Geneva are dated differently and are thus not type material. They could, however, have been collected from the same individual plant, and could be considered topotypes.

##### Specimens examined.

**Chile. Región VI (O’Higgins)**: O’Higgins, Rancagua, La Leonera, 760 m, 9 Dec 2001, *Aedo 7072* (MA); Rancagua, Mar 1828, *Bertero 639* (P); San Fernando, *Philippi* s.n. (G, K). **Región VII (Maule)**: Cauquenes, Hacienda de Cauquenes, , 20 Aug 1896, *Dusén 57* (S); prov. Curico, Cordillera de la Costa, Sep 1897, *Witasek* s.n. (W).

#### 4. Solanum amygdalifolium

Steud., Nomencl. Bot. ed. 2, 2: 600. 1841

http://species-id.net/wiki/Solanum_amygdalifolium

Figure 14

Solanum persicifolium Mart., Flora 21, Beibl. 2: 78. 1838, as “*persicaeifolium*”, non *Solanum persicifolium* Dunal, 1813. Type: Brazil. Rio de Janeiro: São Christovão near Sebastianopolis, *C. Martius 255* (lectotype, designated here: M [M0171803]; isolectotypes: BR, LE, K [K000196335]).
Solanum angustifolium Lam., Tabl. Encycl. 2: 18. 1794, non *Solanum angustifolium* Mill., 1768. Type: Argentina. Buenos Aires: Buenos Aires, *P. Commerson* s.n. (holotype: P-LA [P00357623, Morton neg. 8363]; isotypes: G [G00070233, G00070234], P [Morton neg. 8143], P).
Solanum angustifolium Lam. var. *macrophyllum* Dunal, Prodr. [A.P. de Candolle] 13(1): 90. 1852. Type: Brazil. Bahia: sin. loc. [possibly Rio de Janeiro], *J. Guillot* s.n. (holotype: P [P00319635, Morton neg. 8144]).
Solanum brittonianum Morong, Ann. New York Acad. Sci. 7: 174. 1893. Type: Paraguay. Pilcomayo River, 10 Jan 1888-1890, *T. Morong 1531* (holotype: NY [NY00139076]; isotypes: MO [MO-1787131], NDG, US [US-1324465], WIS).
Solanum handelianum Morong, Ann. New York Acad. Sci. 7: 175. 1893. Type: Based on *Solanum angustifolium* Lam., non *Solanum angustifolium* Mill., 1768.

##### Type.

Based on *Solanum persicifolium* Mart., non *Solanum persicifolium* Dunal, 1813.

##### Description.

Woody vine to 5+ m long, scrambling in low vegetation, semi-aquatic and along water courses. Stems strongly ridged with 4 whitish green wings along the entire length, completely glabrous; new growth minutely papillose, occasionally pubescent with tangled simple uniseriate trichomes, these soon deciduous. Bark of older stems green to pale yellowish green, the bark not markedly exfoliating. Sympodial units plurifoliate, not geminate. Leaves simple, 2–6 cm long, 0.5–2 cm wide, lanceolate to linear (very occasionally with a few shallow lobes, but these lobed leaves always accompanied by simple ones on the same stem), somewhat fleshy to chartaceous, glabrous on both surfaces; primary veins 4–6 pairs, not prominent on either surface; base attenuate; margins entire, not markedly revolute; apex tapering to acute, the ultimate tip rounded; petiole 0.1–0.5 cm long, glabrous or occasionally with a few scattered simple trichomes adaxially, twining to aid climbing. Inflorescences terminal, becoming lateral and sometimes leaf-opposed, 4–13 cm long, usually 4–5 times branched, with 8–15 flowers, glabrous except for a few weak simple uniseriate trichomes at the tips of the branches; peduncle 0.5–2.5 cm long, occasionally absent and the branching beginning at the base of the inflorescence; pedicels 1–1.5 cm long, ca. 1 mm in diameter at the base and apex, slender, spreading at anthesis, glabrous, articulated at the base from a small sleeve and leaving a peg to 1.5 mm high on the inflorescence axis; pedicel scars widely spaced 3–10 mm apart. Buds ellipsoid, the corolla ca. 3/4 exserted from the calyx tube before anthesis. Flowers all perfect, 5-merous. Calyx tube 1.5–3 mm long, conical, the lobes 1–1.5 mm long, deltate to broadly semi-circular, glabrous, the tips papillate. Corolla 2.5–4 cm in diameter, violet, rotate-stellate, lobed ca. 1/2 of the way to the base, the lobes 8–10 mm long, 8–9 mm wide, planar to spreading at anthesis, abaxially densely pubescent with minute simple uniseriate trichomes ca. 0.2 mm long, adaxially glabrous with a few simple trichomes along the midvein. Filament tube minute, the free portion of the filaments ca. 1 mm long, densely pubescent with tangled weak simple uniseriate trichomes to ca. 0.5 mm adaxially so the ovary obscured; anthers 5–6 mm long, 1–1.5 mm wide, ellipsoid, loosely connivent, yellow, poricidal at the tips, the pores usually lengthening to slits with age. Ovary glabrous; style 11–15 mm long, sparsely pubescent in the lower 1/3 to 1/2; stigma clavate, the surface minutely papillate. Fruit a globose to ellipsoid berry, 1–1.2 cm in diameter, to 1.5 cm long, black and dull when mature (yellowish *fide* Cabrera 1983), glabrous, the pericarp thin; fruiting pedicels 1.2–1.5 cm long, ca. 1.5 mm in diameter, more or less woody, pendent from the weight of the berry. Seeds > 40 per berry, 1.5–2 mm long, 1–1.5 mm wide, rounded to flattened-reniform, pale yellow, the surfaces minutely pitted, the testal cells circular. Chromosome number: not known.

**Figure 14.**
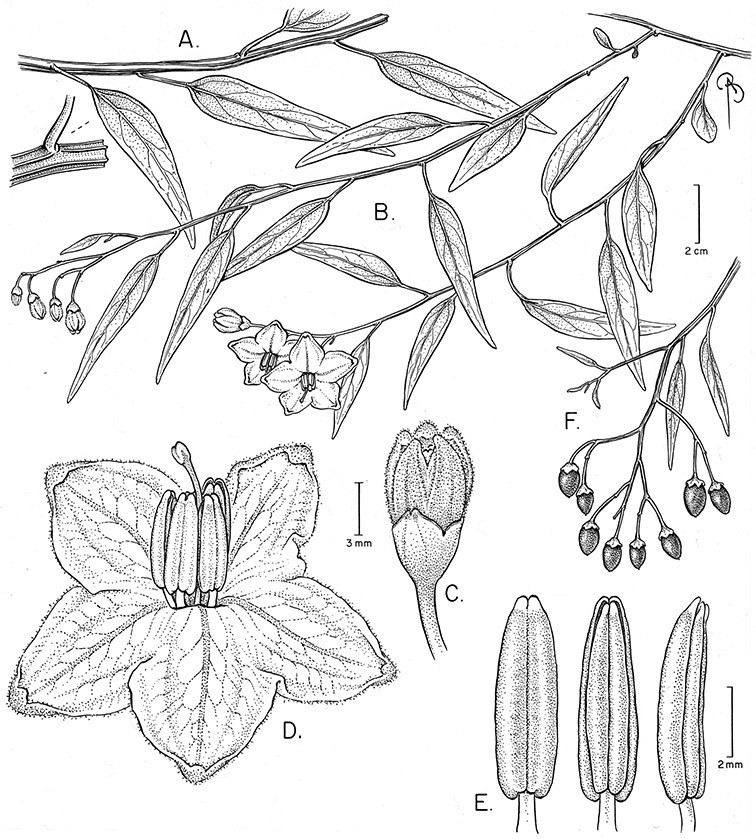
*Solanum amygdalifolium* Steud. (**A, C–E** drawn from *Nee 52046* **B** drawn from *Mendoza 102*). Illustration by Bobbi Angell.

##### Distribution

**Figure 15.**
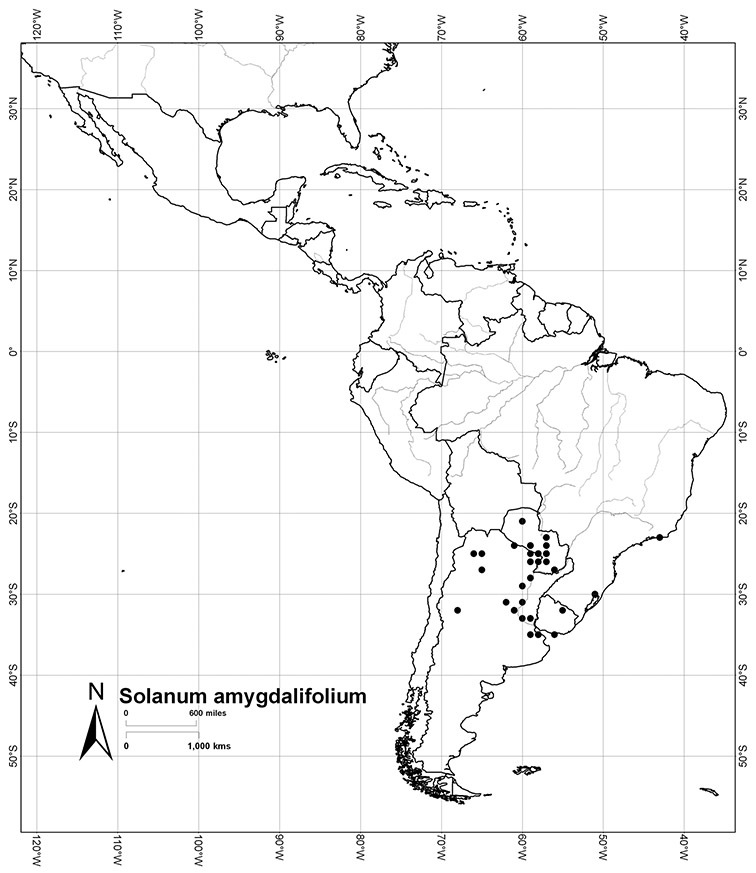
Distribution of *Solanum amygdalifolium* Steud.

(Figure 15). In the Río de la Plata drainage from Buenos Aires, Argentina and adjacent Uruguay to the upper Río Pilcomayo in Paraguay, and in coastal Brazil from Bahia south to Rio Grande do Sul, from 0-700 m elevation. *Solanum amygdalifolium* is also cultivated outside of its native range for its showy flowers (Bolivia, Andean Argentina).

##### Ecology.

Occurs in chaco vegetation along streams and rivers, in thickets and in open vegetation.

##### Common names:

Argentina. duraznillo (Morton 1976); Jujuy: jazmín (Cabrera 1983); Tucumán: amor porteño (Morton 1976).

##### Conservation status.

Least Concern (LC); EOO >50,000 km^2^ (LC) and AOO >5,000 km^2^ (LC). See Moat (2007) for explanation of measurements.

##### Discussion.

*Solanum amygdalifolium* occurs over a very broad geographical range in association with fresh (non-brackish) water, and has been characterised as semi-aquatic by some authors (Mentz and Oliveira 2004). In chaco habitats in Paraguay it always grows in riverside thickets, and forms loose scrambling tangles. With its very large, showy flowers, strongly angled stems and narrow, simple leaves, it is not easily confused with any other species of the Dulcamaroid clade; it is somewhat similar to other dulcamaroids from southern South America, particularly narrow-leaved specimens of *Solanum flaccidum*, but the smaller flowers, more pubescent leaves and unequal filaments of the latter species are distinguishing features. *Solanum flaccidum* grows in completely different types of habitats than does *Solanum amygdalifolium*, so confusion in the field is unlikely.

In general, *Solanum amygdalifolium* is quite monomorphic vegetatively over its broad range, varying only in degree of pubescence between individuals and somewhat in inflorescence size depending on plant age, but the flowers of plants from the Chaco regions of Argentina and Paraguay are much larger than those of plants from more coastal populations near the mouth of the Río de la Plata in Buenos Aires and adjacent Uruguay. Flower size may be related to water availability, as plants collected from near streams and wet places in the wet season all appear to have larger flowers than those from drier areas. The specimens collected in the foothills of the Andes in the provinces of Jujuy and Salta, Argentina appear to have all been from cultivated plants (Cabrera 1983).

Two collections were cited in Martius’s original description of *Solanum persicifolium*; *Martius 255* appears to be the more widely distributed of the syntypes cited in the protologue and as it is a numbered collection is more reliable for tracing duplicates. The duplicate in M here selected as the lectotype has annotations in Martius’s hand. Morton annotated his photograph [Morton neg. 8668] of *Martius* s.n. (São Paulo, the other syntype) at M as holotype, but there is no evidence on the specimen or in the protologue that this is the case.

Morong (Morong and Britton 1893) clearly intended *Solanum handelianum* as a replacement name for *Solanum angustifolium* Lam.; he cites “S. angustifolium Lam., Illus. no. 2343, not Miller” and although he cites a collection of his own from near Asunción in Central Paraguay (*Morong 818*), he does not append “n.sp.” to the epithet as he does for all new taxa he described in 1893 from his own collections.

##### Specimens examined.

**Argentina**. **Buenos Aires**: Buenos Aires, 1831, *Bacle 55* (F,G, G-DC); La Plata, Berisso, 15 Dec 1941, *Beffon* s.n. (SI); Barrancas de Belgrano, Apr 1946, *Castellanos 822* (CORD); Buenos Aires, 10 Apr 1900, *Debeaux 70* (F, GH, US); Río Negro, Allen, Feb 1939, *Hunziker 161* (CORD); Martín Coronado, 8 Apr 1942, *Hunziker 3616* (CORD); Río de la Plata, Reserva Natural Costanera Sur, 9 Jan 1993, *Liede & Conrad 3003* (MO); Avellaneda, Feb 1918, *Molfino 25707* (US); Isla Martín García, Apr 1935, *Pastore 326* (SI); Quilmes, *Rodríguez 179b* (A, SI); Barrancas al Sud, 12 Mar 1902, *Venturi 40* (CORD); **Catamarca**: Ancasti, Las Palomas, 5 Mar 1950, *Brizuela 884* (CORD); Pomán, Dec 1910, *Spegazzini* s.n. (SI); **Chaco**: La Fidelidad, 1918, *Jörgensen 2827* (SI); 1 de Mayo, Colonia Benítez, 30 Sep 1971, *Martínez et al.* s.n. (SI); Colonia Benítez, orilla Río Tragadero, 24 Mar 1948, *Schulz 7251* (F, MO); **Corrientes**: Bella Vista, Paraje Rincón del Ambrosio, 12 Oct 1975, *Irigoyen 248* (MO); Esquina, Islas frente a Esquina, 30 Nov 1974, *Krapovickas et al. 26860* (G, MO); Esquina, Colonia Libertador, arroyo Barrancas, 15 Mar 1975, *Krapovickas et al. 27773* (G, MO); Bella Vista, Bella Vista, nameless island in Río Paraná, some 2 km above Bella Vista, 28 Jan 1956, *Pedersen 3716* (G, GH, US); Concepción, Carambola, 29 Feb 1972, *Pedersen 10075* (A, CORD, L, MO, S); Empedrado, Estancia La Yela, 2 Jun 1984, *Pedersen 13917* (G, MO); **Córdoba**: San Justo, San Francisco, 7 Dec 1946, *Baliguo 944* (B); Capital, Córdoba, estación de ferrocarril Manuel Belgrano, 15 Mar 2000, *Chiarini 316* (CORD); Capital, Córdoba, Apr 1916, *Stuckert 23259* (CORD); Río Segundo, Pilar, en un cerco sobre calle 25 de Mayo, 20 Oct 1991, *Subils 4520* (CORD); **Entre Ríos**: Uruguay, Concepción del Uruguay, alrededores del Banco Pelay, 2005, *Barboza et al. 1566* (CORD); Gualeguaychú, alrededores, Dec 1936, *Cabrera 3966* (F); Victoria, Isla del Pillo, Río Paraná, 20 Apr 1984, *Oberti* s.n. (CORD); Victoria, Isla del Pillo, casa de Sr. Tomas A. Nuñez, 7 Apr 1996, *Oberti* s.n. (CORD); Delta de Paraná y Medanos (Sud de Entre Rios), costas del Río Paraná-Mirri, 7 Jan 1904, *Pennington 128* (CORD); Concepción del Uruguay, Banco Pelay, en camino a Banco Pelay, 5 Apr 1994, *Solís Neffa et al. 59* (GH); La Paz, Isla Curuzú-Chalí, 30 Jan 1981, *Troncoso & Bacigalupo 3127* (MO); **Formosa**: Pirané, Palo Santo, NE de Palo Santo, entre Palo Santo et le Riacho Pilagá, 85 m, 8 Nov 1986, *Charpin & Eskuche 20253* (G); Los Matacos, 15 km east, 600 m, 11 Oct 1938, *Eyerdam & Beetle 22940* (GH); Pilcomayo, Paraíso, 8 Oct 1948, *Morel 6155* (B); Laishí, Herradura, 12 Oct 1950, *Pedersen 1231* (G, US); **La Rioja**: Estación Cebollar, 13 Jan 1910, *Spegazzini* s.n. (SI); **Mendoza**: Guaymallén, El Borbollón, 28 Feb 1947, *Villafaño 828* (B); **Misiones**: Posadas, in ripa limosa fluminis Alto Paraná, 16 Nov 1907, *Ekman 816* (G, S, US); Capital, Nemesio Parma, 30 Feb 1994, *Guillén 393* (CORD); San Pedro, El Alcázar, 6 Apr 1949, *Schwindt 1499* (CORD); **Salta**: Anta, Puerto Verde, 19 Dec 1947, *Luna 551* (B); Rosario de Lerma, Campo Quijano, 1200 m, Feb 1941, *Meyer 3654* (F, GH); Capital, Ciudad de Salta, Av. Chile, proximo al Río Arenales, 12 Jun 1977, *Novara et al. 443* (CORD); Capital, Ciudad de Salta, vias del FFCC entre la estación y calle Olavarria, 1190 m, 25 Oct 1989, *Novara 9053* (CORD, G, S); General Güemes, Ojo de Agua, ruta 10, 6-9 km al NE de Gral. Guemes, 650 m, 12 May 1990, *Novara & Bruno . 9873* (CORD, G, S); **San Juan**: Rawson, Médano de Oro, 650 m, 16 Jan 1987, *Kiesling & Maglioli 6455* (SI); **Santa Fe**: General Obligado, Arroyo Malabrigo, entre Reconquista y Fortín Olmos, ca. 25 km de Reconquista, 10 Nov 1954, *Hunziker 10372* (CORD); Canal Viejo de Santa Fé a Colastiné, Jan 1936, *Job 734* (F, GH); Isla Mascota, La Reconquista, Río Paraná, Jan 1936, *Job 948* (F); General Obligado, Villa Ocampo, (Isla), 29 Jan 1964, *Panigatti 483* (CORD); Las Colonias, entre Esperanza y Santa Fé, 11 Apr 1984, *Penseiro 1510* (SI); San Cristóbal, Arroyo Las Conchas, Ruta 13, 4 Apr 1984, *Prado 625* (CORD); D.P.Malabrigo, F.C. [ferrocarril] a Reconquista, 13 m de Chico Malabrigo, 20 Nov 1906, *Schroeter 164* (SI); **Santiago del Estero**: Rivadavia, Ruta Nac. 34, 100 m, 17 Jul 1964, *Hunziker & Cocucci 17773* (CORD); Quebracho, Paso de la Cina, a orillas de Río Dulce, a unos pocos km al norte de Limache, 6 Jul 1991, *Hunziker 25443* (CORD); Robles, Colonia Jaímez, 12 Sep 1948, *Luna 1354* (CORD); **Tucumán**: Villa Luján, 11 Feb 1919, *Venturi 272* (SI, US).

**Bolivia**. **Santa Cruz**: Cordillera, Bañados de Izozog, Cachari, ‘ACARARENDA’, 340 m, 11 Mar 1991, *Navarro et al. 294* (MO); Florida, Samaipata, 1700 m, 17 Jul 2000, *Wood 16448* (K).

**Brazil**. **Mato Grosso do Sul**: margens do Rio Miranda, 2 Jul 1983, *Conceicao 1441* (MO); **Rio Grande do Sul**: Guaíba, 24 Jan 1949, *Rambo 40159* (B, F); Porto Alegre, Rio Guahyba, Mar 1899, *Reineck & Czermack 379* (G); Morro do Côco, Viamao, 22 Nov 1979, *Soares 175* (F); **Rio de Janeiro**: environs de Rio de Janeiro, *Glaziou 13087* (K, LE); Botafogo, *Guillot* s.n. (P); Itaipu, near Rio de Janeiro, 28 Oct 1828, *Lund* s.n. (BM); Rio de Janeiro, *Macrae* s.n. (GOET); Eugenho Velho, Rio de Janeiro, *Miers 3885* (K); Rio de Janeiro, May 1832, *Riedel 407* (US); Rio de Janeiro, 1844, *Widgren* s.n. (S).

**Paraguay**. **Alto Paraguay**: Parque Nacional Defensores de Chaco, alrededores del Madrejòn, 17 Jul 1985, *Brunner 1218* (G); Madrejón, 20 Jun 1983, *Hahn 1474* (BH, G); **Boquerón**: Estancia Toro Mocho, 17 Feb 2006, *Egea et al. 886* (BM); **Central**: Asunción, Jun 1874, *Balansa 2012* (G); Ypacaray, Cuervo Cui (Cordillera de Altos), Sep 1913, *Hassler 11756* (BM, E, G, GH, L); Lago Ypacaraí, Feb 1903, *Fiebrig 939* (E, F, G, GH, K, L); Asunción, Bahia, Jun 1988, *Mereles 1193* (G); Río Salado, cruce en el camino Linguio-Embocada, Jul 1971, *Schinini 4030* (G); Limpio, Rincón El Peñon, 27 May 1987, *Zardini 2672* (G); Estero de Ypoá, Puerto Guyrati on Río Paraguay, 22 Jun 1993, *Zardini & Guerrero 36385* (G); **Concepción**: Puerto Risso, on Rio Paraguay, between Rio Apa & Rio Aquidaban, 1908, *Fiebrig 4079* (G, K); prope Concepción, Aug 1901, *Hassler 7193* (BM, G, GH, K, P, S); Río Napegue, Aug 1988, *Mereles 1311* (G); Concepción, S. Chaco, 91 m, Jun 1944, *Sandeman 4834* (K); **Cordillera**: Río Salado [Palado], Aug 1901, *Hassler 3207* (BM, G, GH, K, S); San Bernardino, Feb 1915, *Rojas 1624* (S); San Bernardino, ufer des Río Salado, Feb 1916, *Rojas 8563* (K); western side of Río Piribebuy basin, 27 km W of Arroyos y Esteros, 19 May 1990, *Zardini & Velázquez 20317* (G); **Guairá**: Yataití, river Tebacuary, Mar 1933, *Jörgensen 4657* (GH); **Itapúa**: Trinidad, 1914, *Chodat 39* (G); **Misiones**: Llanuras de Santa Ana, *Anonymous 76* (G); Villa Florida, Río Tebicuary, en banco de arena frente a la cuidad, 20 Jul 2000, *Mereles & González Parini 7990* (G); **Paraguarí**: Estero de Ypoá, northern part, 10 km above Nueva Italia on Arroyo Cañabe, 23 Jun 1990, *Zardini & Velázquez 21657* (G); **Presidente Hayes**: Carretera trans-Chaco, km 320, 15 May 1984, *Billiet & Jadin 3063* (MO); Estancia Vanguardia, 29 Mar 2004, *Egea & Centrón 376* (BM); Estancia La Golondrina, Villa Hayes, 9 Sep 1982, *Hahn 673* (BH, G); Estancia Santa Asunción, 25 Oct 2004, *Peña-Chocarro & Egea 1940* (BM); Ruta Ñ, frente a la entrada a Vanguardia, 3 Feb 2005, *Peña-Chocarro & Egea 2404* (BM).

**Uruguay**. **Colonia**: Colonia Valdense, near Gallant, Feb 1957, *Dubugnon 213* (G); **Montevideo**: Montevideo, *Courbon* s.n. (G); Montevideo, *Fruchard* s.n. (F); Atahualpa, 25 m, Apr 1925, *Herter 190* (BH, F, G, GH, MO, S, SI); **Tacuarembó**: Rincón de la Laguna, 15 Feb 1947, *Castellanos 17776* (CORD); ruta 26 km 300, pasando el puente sobre el arroyo Yaguarí, 28 Nov 2001, *Seijo et al. 2541* (MO).

#### 5. Solanum angustifidum

Bitter, Repert. Spec. Nov. Regni Veg. 12: 544. 1913

http://species-id.net/wiki/Solanum_angustifidum

Figure 16

##### Type.

Argentina. Córdoba: San Vicente, *T. Stuckert 4021* (lectotype, designated by Morton 1976, pg. 62: CORD [CORD00004111]; isolectotypes: G [G00070138], GOET [GOET-5966]).

##### Description.

Erect or lax, scandent shrubs, 1–2 m. Stems erect, glabrous; new growth glabrous. Bark of older stems reddish brown to grey. Sympodial units plurifoliate, not geminate. Leaves deeply pinnatifid (pinnate) with 1–4 pairs of leaflets, 2–6.5 cm long, 2–4 cm wide, elliptic in outline, membranous, both surfaces glabrous; primary veins 4–6 pairs; base attenuate onto the petiole; margins deeply pinnatifid, the leaflets (0.5-)2–2.5 long, 0.3–0.4 cm wide at their widest point; apex rounded; petioles 0.5–1.5 cm long, glabrous, sometimes twining. Inflorescences terminal or occasionally lateral, 2–6 cm long, several times branched, with 20–50 flowers, glabrous but with a few simple uniseriate trichomes at the point of pedicel insertion; peduncle 1–3 cm; pedicels 0.7–1(-1.7) cm long, slender, ca. 0.5 mm in diameter at base and apex, glabrous, spreading at anthesis, articulated at the base in a short sleeve, leaving a tiny peg on the inflorescence axis; pedicel scars irregularly spaces 1–4 mm apart. Buds ellipsoid, the corolla exserted from the calyx tube before anthesis. Flowers all perfect, 5-merous. Calyx tube 1–1.5 mm long, cup-shaped, the lobes 0.5–1 mm long, deltate, glabrous or minutely papillate at the tips. Corolla 1.6–2 cm in diameter, lilac, purple or occasionally white, stellate to deeply stellate, lobed 2/3 to 3/4 of the way to the base, the lobes 6–9 mm long, 3–4 mm wide, spreading at anthesis, densely papillate on the margins and midvein abaxially, glabrous adaxially. Filament tube minute, the free portion of the filaments variable, with one filament longer than the rest 2–2.5 mm long, the other four 1–1.5 mm long, all glabrous; anthers 5–6 mm long, 1–1.5 mm wide, ellipsoid, yellow, poricidal at the tips, the pores opening to slits with age. Ovary glabrous; style 9–11 mm long, glabrous; stigma capitate, minutely papillose. Fruit a globose berry, 0.7–1 cm in diameter, black or violet-black when ripe, the pericarp thin and shiny, glabrous; fruiting pedicels 1–1.5 cm long, ca. 1.5 mm in diameter at the base, woody, pendent; fruiting calyx tightly investing the lower portion of the berry and slightly accrescent. Seeds 10–20 per berry, ca. 3 mm long, 2.5 mm wide, flattened reniform, pale brown, the surfaces minutely pitted, the testal cells square or circular. Chromosome number: not known.

**Figure 16.**
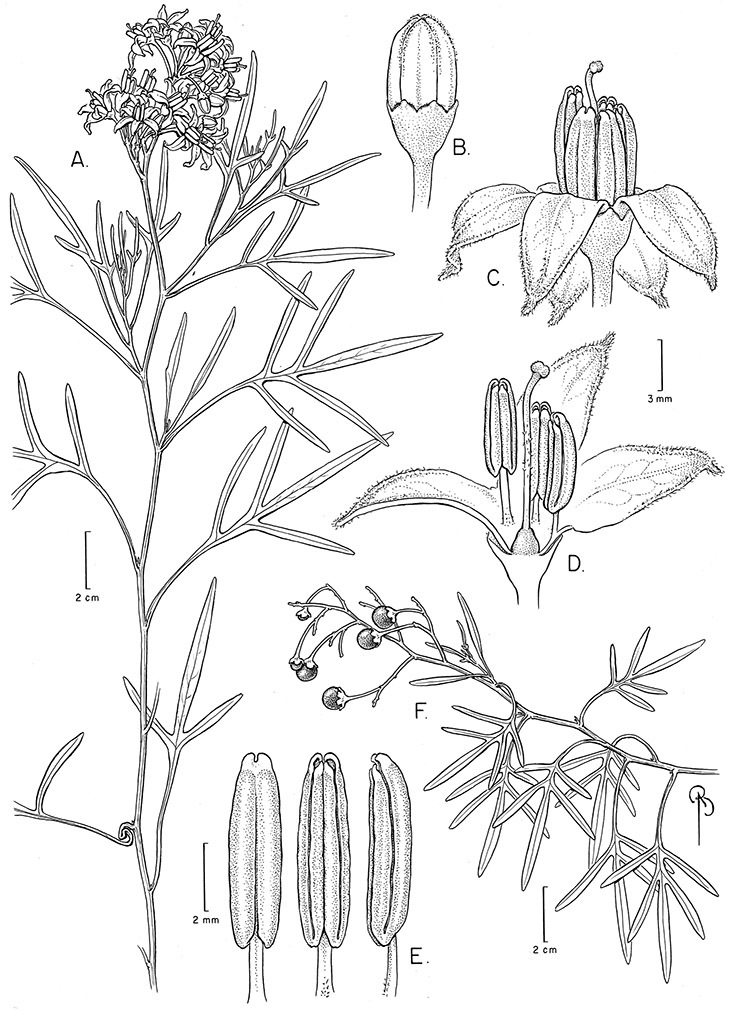
*Solanum angustifidum* Bitter. (**A** drawn from *Tressens et al. 560* **B–E** drawn from *Pedersen 7150* **F** drawn from *Cristóbal et al. 1618*). Illustration by Bobbi Angell.

##### Distribution

**Figure 17.**
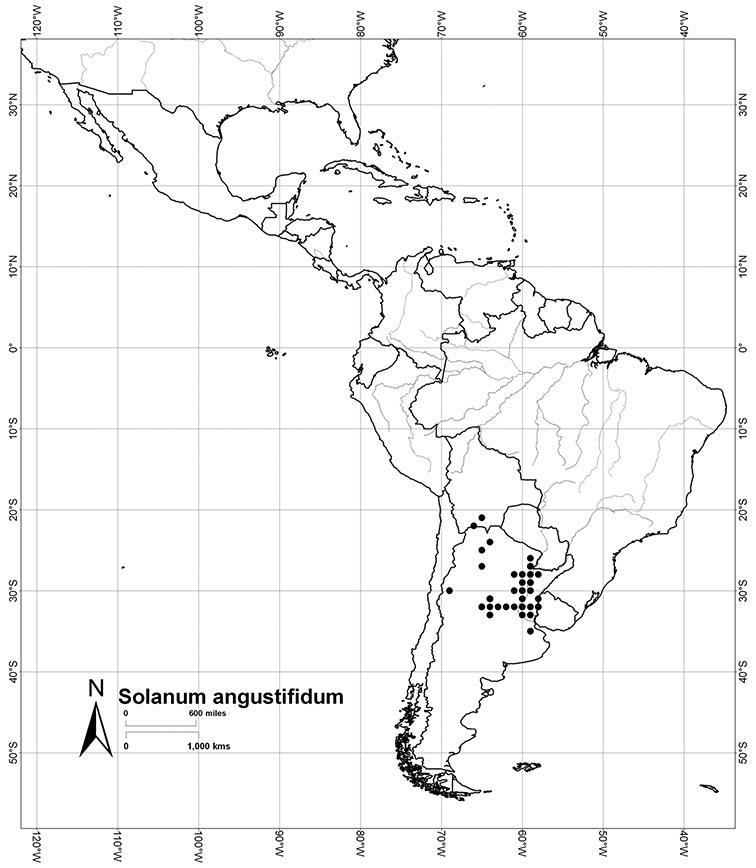
Distribution of *Solanum angustifidum* Bitter.

(Figure 17). Central and northern Argentina, with a few collections from Bolivia; 45–2000 m. Often cultivated (see Figure 1) and found escaped; Bolivian specimens are thought to be escapes from cultivation (Morton 1976).

##### Ecology.

Widely distributed in many vegetation types, from chaco habitats to high elevation dry forests.

##### Common names.

Argentina. jazmín, jazmín de Córdoba, jazmín de cielo (Morton 1976); Córdoba: yerba vaca (*Stuckert 2416*); Jujuy: jazmín (Cabrera 1983).

##### Conservation status.

Least Concern (LC); EOO >50,000 km^2^ (LC) and AOO >5,000 km^2^ (LC). See Moat (2007) for explanation of measurements.

##### Discussion.

*Solanum angustifidum* is distinctive amongst members of the Dulcamaroid clade in having consistently pinnatifid leaves that are divided nearly to the midrib into usually three pairs of linear lobes; in general in the group pinnatifid and simple leaves both occur on individual plants. Although normally a shrubby plant, the petioles of leaves on terminal branches occasionally are elongate and twine around other vegetation. This led Morton (1976) to suggest that this characteristic made *Solanum angustifidum* a linking species between the sections *Dulcamara* and *Jasminosolanum* (both now recognised as part of the larger more inclusive Dulcamaroid clade, Weese and Bohs 2007).

This species is not easily confused with any other in the region; *Solanum salicifolium* has simple or only shallowly pinnatifid leaves and simple inflorescences of usually white flowers, and *Solanum endoadenium* has densely glandular pubescent leaves and orange (rather than black) fruits. *Solanum viscosissimum* of southeastern Brazil has deeply pinnatifid leaves, but is strictly vining and also always has simple leaves on the same stems. *Solanum seaforthianum* is the only other member of the Dulcamaroid clade to have leaves that are almost always pinnate and anthers on unequal filaments, but that species has much broader leaf divisions, rather than the narrow lobes of *Solanum angustifidum*, and is always a vine.

Cabrera (1983) states that *Solanum angustifidum* is commonly cultivated in Argentina for its abundant flowers (see Figure 1), and material from Bolivia appears to have been in association with habitations (Morton 1976; M. Nee, pers. comm.), suggesting that *Solanum angustifidum* is not native there, but is cultivated for its showy purple flowers.

##### Specimens examined.

**Argentina**. **Buenos Aires**: Jardín Botánico, 25 Nov 1922, *Anon.* s.n. (SI); Distrito Federal, Buenos Aires, en el jardin de la estancia Prack, *Hunziker 2267* (CORD); Pergamino, *Parodi 8832* (GH). **Catamarca:** Santa María, La Soledad, 12 Feb 2012, *Barboza et al. 3489* (BM, CORD). **Chaco**: Resistencia, Margarita Belén, 15 Oct 1946, *Aguilar 920* (G); Barranqueras, 35 m, 12 Nov 1913, *Curran 31* (US); Tapenagá, Enrique Urien, campo Bonazzola, lote 9, Nov 1940, *Rodrigo 2405* (GH); San Fernando, 15 km W de Ruta 12 por Ruta 89, 29 Dec 1976, *Schinini 13864* (G, MO); Colonia Benítez, Apr 1932, *Schulz 2066* (MO); Colonia Benítez, 23 Sep 1941, *Schulz 3906* (MO); bords du Río Las Garzas, 100 m, 17 Nov 1902, *Wagner* s.n. (P); **Corrientes**: Paso López, ruta 12, 1.2 km NW de Paso Lopez, 22 Nov 1969, *Carnevali 1746* (MO); Sauce, Río Guayquiraró, 18 km S de Sauce, 9 Oct 1977, *Cristóbal et al. 1618* (MO); Emechado, El Pallo, 2 leguas al Este, 29 Aug 1945, *Ibarrola 3188* (G); Esquina, Ruta 27, 27 km S de Esquina, 1 Dec 1974, *Krapovickas et al. 27062* (G, MO); Esquina, Arroyo Saturno, 14 Mar 1975, *Krapovickas et al. 27684* (G, MO); Empedrado, on the road from San Luis de Palmar to Mburucuyá, just north of the ford across the Río Empedrado, 20 Sep 1952, *Pedersen 1834* (G, K, MO, US); Empedrado, Estancia La Yala, 20 May 1956, *Pedersen 3916* (G, LE, US); Curuzú Cuatiá, Ruta 12 km 716, 20 Dec 1957, *Pedersen 4785* (G, US); Saladas, Santo Domingo, 21 Jan 1950, *Schwarz 9313* (CORD); Saladas, Estancia Pancho, 14 Feb 1950, *Schwarz 9702* (CORD); Curuzú Cuatiá, Paso López, 17 km E, ruta 12, 29 Oct 1974, *Tressens et al. 560* (MO); **Córdoba**: Capital, Córdoba, camino que une la Evenida Colon al 6000 con la ruta provincial 28 (ex. nac. 20), por detras de la Escuela de Aviación, 2 Dec 1973, *Ariza Espinar 2857* (CORD); Punilla, Valle Hermoso, Valle de Punilla, Vaquerias, en camino de tierra que va de Casa Grande a Valle Hermoso, despues del segundo paso a nivel (a la derecha), 14 Dec 1972, *Barrera et al. 8244* (CORD); Sierra Chica, Cerro Negro cerca de Cerro Azul, 9 Mar 1955, *Castellanos 3353* (CORD); Ischilín, Deán Funes, 5 Nov 1945, *Cuezzo 873* (B, CORD); Punilla, Salsipuedes, 6 Apr 1947, *Dawson 1744* (K); Colón, entre Salsipuedes y Bello Horizonte, en el camino a La Falda, 29 Oct 1974, *Hunziker 22611* (CORD); La Falda, Jan 1936, *Job 548* (S, US); Alta Gracia, 600 m, 30 Mar 1930, *King 631* (BM); Capital, Córdoba, 1 Jan 1885, *Kurtz 964* (CORD); San Alberto, Villa Cura Brochero, Sierra Grande, falda O, 2 Feb 1948, *Meyer 13518* (CORD); Colón, Jesús María, 530 m, 23 Dec 1947, *Meyer 13602* (CORD); Colón, Salsipuedes, 26 Mar 1944, *O’Donell & Rodríguez 690* (A); San Alberto, Nono, 22 Mar 1944, *O’Donell & Rodríguez 721* (A, CORD); Colón, Ascochinga, 14 Mar 1944, *O’Donell & Rodríguez 884* (F); Hendiolaza, 16 Mar 1944, *Ruiz de Huidrobo 69* (GH); Río Segundo, Pilar, 2 May 1999, *Subils 4662* (CORD); **Entre Ríos**: Capital, Río Paraná, 1891, *Anetto* s.n. (CORD); ruta Villaguaey a C. del Uruguay, bajos del Río Gualaguaey, 10 Nov 1973, *Burkart et al. 30139* (MO, SI); La Paz, Paso Yunque, Río Guayquiraró, 7 Nov 1973, *Burkart et al. 30151* (SI); Río Gualeguay, ruta Villaguay a C. del Uruguay, 10 Nov 1973, *Burkart 30319* (GH, K); Las Barrancas, *Doering* s.n. (CORD); Puerto Nuevo, barranca del Paraná, 1 Dec 1912, *Hicken* s.n. (SI); Feliciano, San José de Feliciano, El Caraya, Feb 1948, *Martínez Crovetto 4850* (K); Villaguay, Estancia Santa Martha, 1937, *Museh* s.n. (SI); Federación, Estancia Buena Esperanza, 15 Nov 1964, *Pedersen 7150* (A, K); N of La Paz, Estancia Santa Cruz Cué, 7 Nov 1965, *Walter & Walter 331* (B); **Formosa**: Jujuy, Pilagá, 5 Oct 1945, *Pierotti 4201* (B); Pilagá, Pilagá, 3 Oct 1945, *Pierotti 4271* (BM); **La Rioja**: Sanagasta, Sanagasta, 1500 m, 18 Feb 1944, *Hunziker 4752* (CORD); Famatina, Famatina, Ruto 40, 9 Jan 1947, *Hunziker 1825* (CORD); Famatima, Aguaditas, 7 Apr 1949, *Toscani 51* (K); **Salta**: La Caldera, ruta 9, 18 km camino a Jujuy, 15 Nov 1947, *Dawson 2032* (K); La Caldera, Dique Campo Alegre, ruta 9, km 1232, proximo a ruinas abandonadas de antiguo camping, 10 Jan 2005, *Novara 12197* (CORD); **San Juan**: Iglesia, Arrequintín, 15 Mar 1989, *Pedersen 15243* (G); **San Luis**: San Martín, San Martín, 1000 m, 13 Feb 1944, *Varela 519* (CORD, G); **Santa Fe**: San Martín, Piamonte, alrededores, 24 Nov 1974, *Astegiano 35* (CORD); General Obligado, Berna, ruta 11, km 759, 25 Jan 1992, *Bernadello & Galetto 790* (CORD); San Cristóbal, Arroyo Las Conchas, Ruta 13, 26 Nov 1983, *D’Angelo & Penseiro 611* (CORD); Cuty Lai, 6 Apr 1917, *Hosseus 75* (CORD); Vera, entre Margarita y Vera, 8 Nov 1954, *Hunziker 10357* (CORD); Capital, Santa Fe, en el Río Paraná, 7 Apr 1901, *Kurtz 11857.5* (CORD); Sorrento, 7 Apr 1901, *Kurtz 11857 bis* (CORD); Villa Guillermina, Nov 1939, *Meyer 3067* (US); General Obligado, camino entre Villa Guillermina y El Rabón, 18 Feb 1988, *Penseiro & Tivano 3262* (CORD); San Justo, Fives Lille, 4 Jan 1937, *Ragonese 2680* (US); Gral. Obligado, Mocoví, 1904, *Venturi 51* (CORD, K); **Tucumán**: Capital, Tucumán, 450 m, 17 Oct 1897, *Lillo 2030* (A); Camino Madillal, road Tucumán to Racas, 760 m, 27 Dec 1935, *Mexia 4333* (MO).

**Bolivia**. **Potosí**: Suipacha, 2800 m, Aug 1946, *Cárdenas 3731* (US). **Tarija**: Tarija, Jul 1932, *Cárdenas 188* (GH); Mendez, Tomatitas, ca. 5 km N of Tarija, 2050 m, 21 Jan 2000, *Wood et al. 15824* (K).

#### 6. Solanum aspersum

S.Knapp, PLoS ONE 5(5): e10502. 2010

http://species-id.net/wiki/Solanum_aspersum

Figure 18

##### Type.

Colombia. Putumayo: vertiente oriental de la Cordillera, entre Sachamates y San Francisco de Sibundoy, 1600-1750 m, 30 Dec 1940, *J. Cuatrecasas 11471* (holotype: COL; isotypes: F [F-1335119], US [US-1799731]).

##### Description.

Woody vine, of unspecified length or height. Stems densely and evenly pubescent with antrorsely curved simple uniseriate trichomes 0.5–1.5 mm long, these few-celled with a large basal cell, arising from expanded bases and eventually deciduous; new growth densely pubescent with simple uniseriate trichomes to 1.5 mm long, these pale straw-colored in herbarium specimens; bark of older stems greenish brown, minutely tuberculate from the bases of the deciduous trichomes. Sympodial units plurifoliate. Leaves simple, (1-)3.5–9 cm long, (0.6-)1.5–4.6 cm wide, ovate to narrowly ovate, widest in the basal third, membranous or chartaceous, strongly discolorous, the upper surfaces evenly pubescent on veins and lamina, the trichomes to 2 mm long, simple, uniseriate, arising from expanded bases giving the lamina surface a tuberculate appearance, the lower surfaces densely and evenly pubescent with simple uniseriate trichomes to 2 mm long, these 2–3-celled with the basal cell largest, denser on the veins; primary veins 7–9 pairs, impressed above in herbarium specimens; base truncate or shallowly cordate; margins entire, not revolute; apex acute to acuminate; petioles 0.7–2 cm long, densely pubescent with simple trichomes like those of the stems and leaves. Inflorescences terminal on leafy short shoots, 3–15 cm long, globose to ellipsoid in outline, branching many times, with 2 principal basal branches, with 12–60 flowers, densely pubescent with simple trichomes; peduncle 0.5–3 cm long, the branching very near the junction with the stem; pedicels 0.5–0.8 cm long, < 0.5 mm in diameter at the base and apex, pubescent with 1–2-celled simple trichomes to 1.5 mm long, spreading at anthesis, articulated near the base from a small sleeve, leaving a small peg on the axis; pedicel scars irregularly spaced 1–10 mm apart, the inflorescence rachis bending at almost right angles at articulation points. Buds narrowly ellipsoid, the corolla strongly exserted from the calyx tube. Flowers all perfect, 5-merous. Calyx tube ca. 2 mm long, conical, the lobes 0.5–1 mm long, deltate to broadly deltate, pubescent with simple trichomes, these sparser than on the rest of the inflorescence. Corolla 1.2–1.7 cm in diameter, white, pink or “pale blue” (violet?), narrowly stellate, lobed nearly to the base, the lobes 6–7 mm long, 1.5–2 mm wide, reflexed at anthesis, densely pubescent abaxially with weak simple papillate trichomes to 0.5 mm long, these denser on tips and margins, glabrous adaxially. Filament tube < 0.5 mm, the free portion of the filaments ca. 0.5 mm long, glabrous; anthers 4–4.5 mm long, ca. 1 mm wide, ellipsoid, poricidal at the tips, the pores lengthening to slits with age. Ovary glabrous; style 5–6 mm long, pubescent with weak simple trichomes to 0.5 mm, more densely pubescent in the basal half; stigma capitate-truncate, the surface minutely papillose. Fruit a globose berry, ca. 1.3 cm in diameter (immature?), green or yellowish green, the pericarp thin and shiny, glabrous; fruiting pedicels 0.9–1 cm long, 1–1.5 mm in diameter at the base, woody and spreading. Seeds not seen from mature berries, apparently 10+ per berry and flattened reniform. Chromosome number: not known.

**Figure 18.**
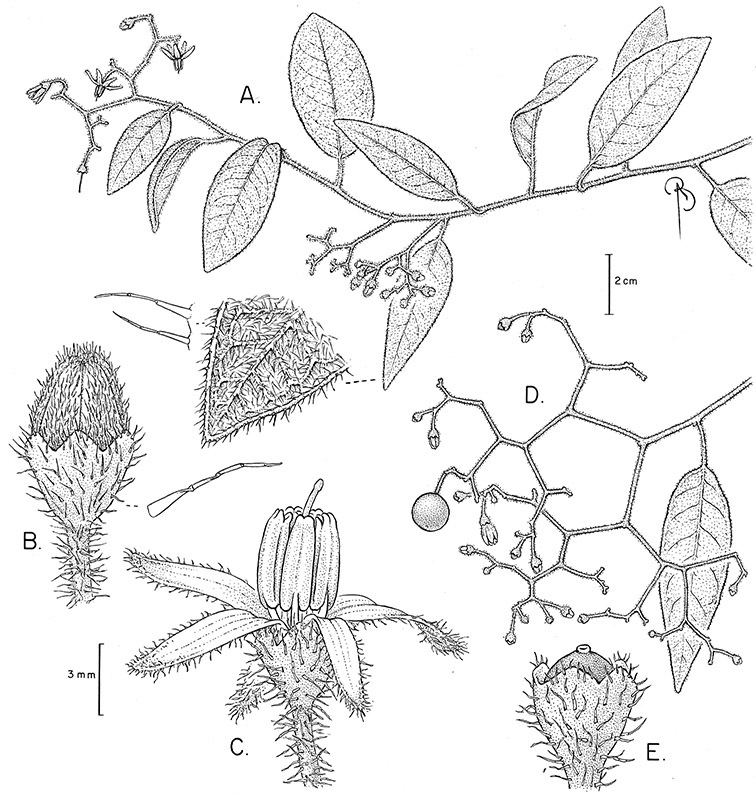
*Solanum aspersum* S. Knapp. (**A–C** drawn from *MacDougal et al. 4251* **D–E** from *Zak 1857A*). Originally published in Knapp (2010). Illustration by Bobbi Angell.

##### Distribution

**Figure 19.**
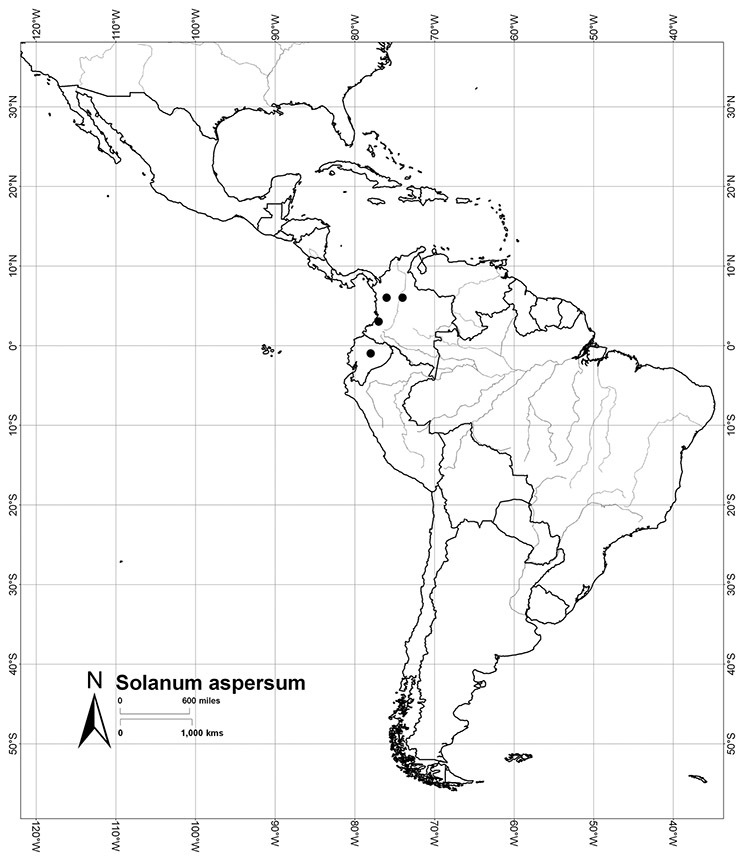
Distribution of *Solanum aspersum* S.Knapp

(Figure 19). *Solanum aspersum* occurs in widely separated and isolated populations along the Andes from central Ecuador into Colombia, from 1600 to 2500 m.

##### Ecology.

Montane forests and forest margins.

##### Conservation status.

Vulnerable/Near Threatened (VU/NT); EOO >50,000 km^2^ (LC) and AOO >5,000 km^2^ (LC); the widely separated, isolated populations and few collections, however, mean that this species is of conservation concern. See Moat (2007) for explanation of measurements.

##### Discussion.

The few specimens of *Solanum aspersum* have usually been annotated as the more common and widely distributed *Solanum aureum*, also from Andean Ecuador. *Solanum aspersum* differs from that species in its simple uniseriate, rather than congested-dendritic, pubescence and in the elongate buds that open to deeply stellate flowers. Specimens of *Solanum aureum* from Azuay province in Ecuador have shiny adaxial leaf surfaces like those of *Solanum aspersum*, but always have the characteristic golden dendritic pubescence of that species rather than the simple pubescence of *Solanum aspersum*. The leaves of *Solanum aspersum* are usually more cordate than those of *Solanum aureum*, but some populations of *Solanum aureum* approach *Solanum aspersum* in overall leaf morphology at first glance. *Solanum aspersum* has a very scattered distribution all along the Andes from northern Colombia to central Ecuador and is likely to be found in more of the intervening parts of the cordilleras, but it is apparently rare and easily overlooked.

A single collection from Cajamarca in northern Peru (*Diaz et al. 9717*, NY) with simple trichomes on the inflorescence and completely glabrous leaves may also be this species; it shares the elongate buds and simple trichomes, but may represent a different taxon.

##### Specimens examined.

**Colombia**. **Antioquia**: Frontino, Nutibara, region Muri, camino hasta La Blanquita, 1440 m, 10 Jul 1986, *Acevedo et al. 1213* (US); Urrao, between Urrao and caicedo, 21 km E of Urrao, near high point on road, 2710 m, 27 Feb 1989, *MacDougal et al. 4251* (MO). **Cauca**: between La Cumbre and Quebrada La Isla, headwaters of Río Dinde, 28 Aug 1944, *Core 1111* (US); **Cundinamarca**: Chiquinquirá, 2500 m, 17 Feb 1950, *Sneidern 5825* (S).

**Ecuador**. **Napo**: Parroquia Cosanga, 6 kms de la carretera Cosanga-El Aliso, 2200 m, 23 Aug 1990, *Jaramillo et al. 12110* (MO); Cantón Quijos, Reserva Ecológica Antisana, Río Aliso, 8 km al suroeste de Cosanga, 2500 m, 15 Nov 1998, *Vargas et al. 3043* (MO). **Pichincha**: Reserva Florística-Ecológica “Río Guajalito”, km 59 de la carretera antigua Quito-Santo Domingo de los Colorados, a 3.5 km al NE de la carretera, 2200 m, 28 Mar 1987, *Zak 1857A* (F, MO).

#### 7. Solanum aureum

Dunal, Solan. Syn. 16. 1816.

http://species-id.net/wiki/Solanum_aureum

Figure 20

Solanum loxense Dunal, Solan. Syn. 16. 1816. Type: Ecuador. Loja: Loja, *A. Humboldt & A. Bonpland* s.n. (holotype: P [P00136336]).
Solanum clematideum Bitter, Repert. Spec. Nov. Regni Veg. 16: 86. 1919. Type: Ecuador. Azuay: Cuenca, Pindilic [Pindilig], 2600–3000 m, 11 Oct 1888, *F. Lehmann 4948* (holotype: B [F neg. 2654], destroyed; lectotype, designated here: K [K000438685]).
Solanum aureum Dunal var. *riobambense* Werd., Notizbl. Bot. Gart. Berlin-Dahlem 12: 379. 1935. Type: Ecuador. Chimborazo: Ríobamba, N of Sanancajas, 3300 m, 28 Jul 1933, *L. Diels 355* (holotype: B, destroyed; no duplicates traced).

##### Type.

Ecuador. Base of Chimborazo, June 1802, *A. Humboldt & A. Bonpland 3142* (holotype: P [P00136318, Morton neg. 8151]; isotypes: B-W [B-W-4318, IDC microfiche 271-315.297:5, F neg. 2890], P [P00136319]).

##### Description.

Woody vines or lax shrubs to 5 m tall, often in open areas. Stems densely pubescent with stiff, golden dendritic trichomes to 1 mm long, the trichome branches numerous and very short; new growth densely golden-pubescent with congested dendritic trichomes. Bark of older stems pale brownish yellow, glabrescent. Sympodial units plurifoliate, not geminate. Leaves simple, 1.5–7(-11) cm long, 0.9–3(-6) cm wide, elliptic to narrowly elliptic, membranous to slightly chartaceous or fleshy, discolorous, the upper surfaces shiny, sparsely and evenly pubescent with erect dendritic trichomes to 0.5 mm long, these with short and congested branches, occasionally glabrous with only a few trichomes along the midvein, the lower surfaces densely pubescent on veins and lamina with erect, golden, dendritic trichomes to 1 mm long, with short, congested branches, the surface appearing golden; primary veins 7–10 pairs, paler than the lamina, golden beneath; base truncate to acute, occasionally oblique; margins entire, not markedly revolute; apex acute; petioles 0.5–2 (3.5) cm long, densely golden pubescent with dendritic trichomes like those of the stems, occasionally twining to aid in climbing. Inflorescences terminal, occasionally becoming lateral and internodal, 5–10 cm long, globose in outline, many times branched, leafy near the basal branches, with 20–40 (+) flowers, densely pubescent with golden dendritic trichomes like those of the stems; peduncle very short, the transition between stem and inflorescence not always clear; pedicels 0.5–0.6 cm long, stout or appearing so from dense pubescence, ca. 0.5 mm in diameter at the base, ca. 1 mm in diameter at the apex, spreading at anthesis, densely golden-pubescent with stiff dendritic trichomes to 1 mm long, articulated at the base leaving a small sleeve or peg 0.5–1 mm long on the axis; pedicel scars irregularly spaced 1–10 mm apart, obscured by the pubescence. Buds globose to broadly ellipsoid, the corolla strongly exserted from the calyx tube before anthesis. Flowers all perfect, 5-merous. Calyx tube 2–2.5 mm long, conical, the lobes 1.5–2 mm long, deltate to narrowly deltate, the apex blunt, densely to sparsely pubescent with golden dendritic trichomes to 1 mm, the pubescence usually sparser than that of the pedicel. Corolla 1.5–1.7 cm in diameter, violet or purple to deep purple, stellate, lobed ca. 2/3 of the way to the base, the lobes 5–7 mm long, 3.5–5.5 mm wide, planar at anthesis, densely pubescent abaxially with a mixture of simple and dendritic trichomes to 0.5 mm long, these weak and tangled, denser on the tips and margins, the interpetalar sinuses glabrous, glabrous adaxially. Filament tube ca. 0.5 mm long, the free portion of the filaments ca. 1 mm long, glabrous; anthers 4–5 mm long, 1.2–1.5 mm wide, glabrous, ellipsoid, loosely connivent, yellow, poricidal at the tips, the pores lengthening to slits with age. Ovary glabrous; style 7–8 mm long, glabrous or densely and minutely papillate in the basal 1.3; stigma capitate, minutely papillate. Fruit a globose berry, ca. 1 cm in diameter, purplish black and shiny when ripe, glabrous, the pericarp thin; fruiting pedicels 1–1.2 cm long, ca. 1 mm in diameter at the base, woody and spreading, the calyx lobes woody and persistent around the base of the berry. Seeds 20–30 per berry, ca. 3 mm long, 2.5–3 mm wide, flattened-reniform, the margins undulate, reddish brown, the testal surface minutely pitted, the cells pentagonal in outline. Chromosome number: not known.

**Figure 20.**
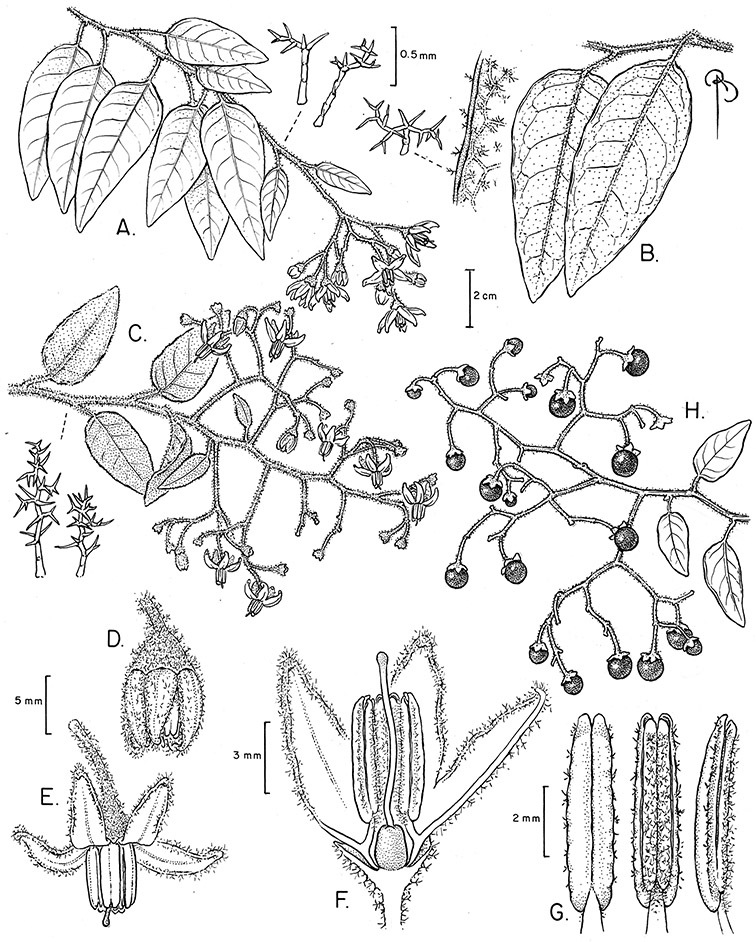
*Solanum aureum* Dunal. (**A** drawn from *Juncosa 2335*, NY **B** drawn from *Zak 1845* **C–G** drawn from *Rose 22963* **H** drawn from *Cerón et al. 11842*). Illustration by Bobbi Angell.

##### Distribution

**Figure 21.**
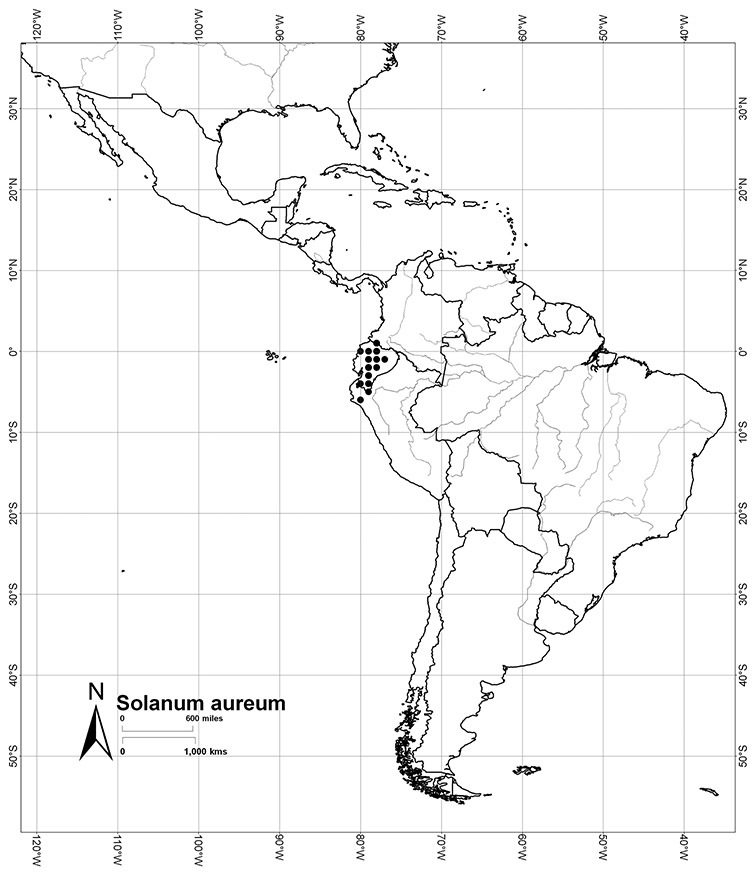
Distribution of *Solanum aureum* Dunal.

(Figure 21). In Andean Ecuador and Peru, from 2500 to 3700 m elevation, most commonly collected around 3000 m.

##### Ecology.

Most commonly collected in montane forests, páramos and páramo margins (“ceja de selva” or “jalca”).

##### Conservation status.

Least Concern (LC); EOO >50,000 km^2^ (LC) and AOO >5,000 km^2^ (LC). See Moat (2007) for explanation of measurements.

##### Discussion.

*Solanum aureum* is widespread in Ecuador and has been considered to include several species here recognised as distinct: *Solanum sanchez-vegae* of northern Peru, *Solanum dichroandrum* of northern Colombia and Venezuela and *Solanum aspersum* of Ecuador and Colombia, with which *Solanum aureum* is sympatric. *Solanum aureum* is almost always described as a vine, but occasionally as shrubby, a habit variously common in the Dulcamaroid clade. *Solanum sanchez-vegae* and *Solanum dichroandrum* differ from *Solanum aureum* in their looser, less dense leaf pubescence and somewhat larger flowers. All three taxa have dark purplish black berries, but those of *Solanum aureum* are somewhat smaller. *Solanum aureum* and *Solanum sanchez-vegae* may overlap in distribution in northern Peru in the Huancabamba depression; *Solanum dichroandrum* is only known Colombia and Venezuela. Specimens now recognised as *Solanum aspersum* had long been considered as conspecific with *Solanum aureum*, but differ from it in their simple trichomes from bullate bases, elliptic buds and flowers with more lanceolate corolla lobes. These two species are sympatric in northern Ecuador. *Solanum cutervanum* is also sympatric with *Solanum aureum* through much of its range and is easily confused with it. The most obvious difference in the two taxa is habit, with *Solanum aureum* being a vine and *Solanum cutervanum* a shrub, but also the leaf and stem trichomes of *Solanum cutervanum* are beige, more elongate and the branches shorter than those of *Solanum aureum*, in which the trichomes are golden and more classically dendritic.

In Prov. Azuay, Ecuador, *Solanum aureum* has markedly shiny upper leaf surfaces and is superficially very like *Solanum aspersum*, but the trichomes from these plants are always dendritic with short branches and the flowers have deltate corolla lobes. The type of *Solanum clematideum* comes from among these populations.

In describing both *Solanum aureum* and *Solanum loxense*, Dunal cited specimens in the Humboldt and Bonpland herbarium (“v.s.h. H. et B.”); no sheets of either are presently in P-Bonpl., but material collected by Humboldt and Bonpland is housed in the general collection and is certainly that used by Dunal in describing these taxa. Many specimens from the original Humboldt and Bonpland collection were apparently re-integrated into the general collections after use in the 19^th^ century rather than being returned to the special collection (see Knapp and Spooner 1999; Knapp 2007b).

##### Specimens examined.

**Colombia**. **Cauca**: Pitayó, Prov. of Popayán, *Anonymous* s.n. (K); **Putumayo**: road from Sibundoy to Pasto, between La Maria and Páramo de San Antonio, 2900 m, 1 Jun 1946, *Schultes & Villarreal 7536* (GH, K, US); **Valle del Cauca**: Cuchilla de Barragán, Cordillera Central, vertiente occidental, hoya del Río Bugalagrande, 3250 m, 12 Apr 1946, *Cuatrecasas 20605* (F, US).

**Ecuador**. **Azuay**: Cantón Cuenca, Yanasacha, Parroquia Baños, 2925 m, 26 Dec 1976, *Boeke 595* (GH, MO); Cruz Pamba region above Baños, ca. 15 km southwest of Cuenca, 2743 m, 29 Jun 1945, *Camp E-3940* (MO); Campamento Molón, road in construction Sigsig-Gualaquiza, 2900 m, 11 Apr 1968, *Harling et al. 8218* (MO x2); Cumbe, 24 Sep 1918, *Rose et al. 22963* (GH, US); Bulán, Padrehurco-Amapala, en las riberas del río Chanín, 2776 m, 18 Oct 2000, *Verdugo et al. 285* (HA); Cantón Girón, Girón, El Chorro, 2700 m, 22 Jun 2001, *Verdugo et al. 353* (HA); **Bolívar**: Río Tataguazó, carretera Santiago-San Vicente-Alizo “San Agustin”, 2500 m, 24 Feb 1987, *Zak 1741* (F); **Carchi**: Páramo del Ángel, 3300 m, 19 Jul 1945, *Acosta Solís 10571* (F); El Ángel, 1 Jan 1931, *Benoist 3630* (P); La Rincónada, ranch between Ibarra and Tulcán, 3000 m, 10 Aug 1923, *Hitchcock 20947* (GH, US); ca. 2 km along the road El Angel-Tulcán, 3150 m, 14 May 1973, *Holm-Nielsen et al. 5207* (F, MO); Road Julio Andrade-Palestina, 3300 m, 27 Dec 1980, *Holm-Nielsen et al. 29707* (BM); near El Angel on old road to Tulcán, 3060 m, 23 Feb 1984, *Juncosa 2335* (MO); Cantón Espejo, Reserva Ecológica El Ángel, Libertad-Morán, El Salado, 3300 m, 30 Oct 1993, *Palacios 11547* (MO); Quebrada del Río Angel, 3 km NE of San Isidro on road to El Angel, 28 Jan 1967, *Sparre 14280* (S); **Cañar**: north of Biblian, 2900 m, 6 Apr 1974, *Harling & Andersson 13239* (MO); carretera Alausi-Cañar, desvio a Oyashig-Hacda. El Carmen al N de Cañar, 3270 m, 12 Aug 1987, *Zak 2400* (F, MO); carretera Alausi-Cañar, desvio a Oyashig-Hacda. El Carmen al N de Cañar, 3270 m, 12 Aug 1987, *Zak 2403* (F, S); **Chimborazo**: Cubillín, Cordillera Oriental, 3300 m, 1 Mar 1944, *Acosta-Solís 7575 A* (F); Cantón Riobamba, Parque Nacional Sangay, páramo de Pinlilligue, entre Alao y La Tranca, 3300 m, 18 Aug 1990, *Cerón et al. 11842* (MO); 3 km E of Alao, above Río Alao, on road to Huamboya (Morona-Santiago), 2 Dec 1988, *Dorr & Barnett 6207* (MO); Cantón Riobamba, Riobamba, 3300 m, 22 Mar 1934, *Schimpff 896* (G); Bayushig, carretera Riobamba-Penipe-Bayushig, 3400 m, 18 Feb 1986, *Zak 1635* (F); Cantón Riobamba, Parque Nacional Sangay, carretera Chimborazo-Chambo-Pungalá-Alao, 3150 m, 9 Aug 1987, *Zak 2360* (F, F, MO); **Cotopaxi**: Pilalo, 2400 m, 9 Jul 1968, *Holm-Nielsen, & Jeppesen 1541* (S); Road Pilaló-Zumbagua, 10 km above Pilaló, 3150 m, 28 Jul 1980, *Holm-Nielsen & Quintana 24654* (BM); **Imbabura**: Shanshipamba-La Esperanza, 2950 m, 16 Nov 1949, *Acosta-Solís 14422* (F); Laguna Cuicocha, crater lake 30 W of Ibarra, steep slopes around lake and on the smaller island in the lake, 3100 m, 24 May 1973, *Holm-Nielsen et al. 6321* (S); Cantón Otavalo, Otovalo, Páramo de Mojanda, 3200 m, *Lehmann K-222* (K); Lake Cuicocha, island in lake, 3100 m, 29 May 1939, *Penland & Summers 761* (F, GH, US); Cantón Cotacachi, Reserva Ecológica Cotacachi-Cayapas, islote Teodoro Wolff, laguna de Cuicocha, 3100 m, 30 Aug 1991, *Peñafiel et al. 313* (BM, MO); Carretera Pimampiro-Chuga-Palmar Chico; entre Chuga y Palmar Chico, 2800 m, 16 Jan 1988, *Zak & Jaramillo 3353* (BM, F x2, GH, MO, MO); **Loja**: between Loja and San Lucas, 2100 m, 6 Sep 1923, *Hitchcock 21497* (GH, US); Cerro de Celica. Celica-Guachanamá, Km 14-18, 2700 m, 13 Apr 1994, *Jørgensen et al. 157* (BM); El Cisne-Zaruma, unfinished road km 2.9, 2340 m, 12 Dec 1994, *Jørgensen et al. 1397* (MO); Catamayo-Catacocha, km 25, turnoff at Las Chinchas towards Piñas, km 2.3, 2250 m, 13 Dec 1994, *Jørgensen et al. 1463* (MO); **Pichincha**: Cantón Cayambe, Oyacachi, Cordillera Oriental, 3200 m, 26 Sep 1945, *Acosta-Solís 11148* (F); entre Oyacachi y Comenia, Cordillera Oriental, 3000 m, 27 Sep 1945, *Acosta-Solís 11204* (F); El Llalo, 3201 m, 29 Dec 1938, *Balls B-5826* (BM, E, K, US); base du Pichincha, 23 Mar 1930, *Benoist 2211* (P); Paluguillo, 19 Feb 1931, *Benoist 3908* (P); Cantón Cayambe, Reserva Cayambe-Coca, zona de amortiguamiento, 3200 m, 2 Jan 2000, *Cuamacás et al. 579* (MO); Cantón Quito, Pifo, Hacienda Los Andes, 3500 m, 24 Jan 1991, *Palacios 6906* (MO); Lloa, *Sodiro* s.n. (P); Hac. Paluguillo, on road Pifo-Papallacta, 3200 m, 19 Jul 1967, *Sparre 17727* (S); carretera Quito-Guantopugro-Yanacocha, 3400 m, 22 Mar 1987, *Zak 1845* (F, MO); Cantón Mejía, Parroquia El Chaupi, faldas del Volcán El Corazón, 3300 m, 23 May 1988, *Zak & Jaramillo 3698* (BM, MO); **Tungurahua**: entre Leito y La Cima, Cordillera Oriental, 2700 m, 15 Nov 1944, *Acosta-Solís 8994* (F); Cantón Mocha, Mocha, 6 Jul 1896, *André 3922* (K); road between El Triunfo and Patate, summit of road that descends to Patate, 3140 m, 26 Feb 2003, *Bohs et al. 3132* (UT); Cantón Mocha, Mocha, base of Chimborazo, 2743 m, *Jameson 46* (K); Cantón Ambato, Ambato, 3048 m, Jul 1939, *Sandeman 37* (BM, K); Cantón Mocha, Mocha, alrededores del pueblo, 2900 m, 30 Sep 1995, *Villacres 373* (F).

**Peru**. [perhaps Ayabaca?], 1909, *Weberbauer 6411* (F); **Cajamarca**: San Ignacio, San José de Lourdes. Base del Cerro Picorana, 25 Aug 1999, *Díaz et al. 10743* (MO); **Piura**: Huancabamba, carretera entre Canchaque y Huancabamba, 2800 m, 14 Jan 1988, *Díaz et al. 2719* (MO); Huancabamba, Cruz Blanca-Turmalina, 2600 m, 15 Sep 1981, *López M. et al. 8926* (BM, MO); Huancabamba, Huancabamba, 2591 m, Aug 1943, *Sandeman 4262* (K).

#### 8. Solanum boldoense

Dunal & A.DC., Prodr. [A.P. de Candolle] 13(1): 679 1852

http://species-id.net/wiki/Solanum_boldoense

Figure 22

Solanum cardiophyllum Dunal, Prodr. [A.P. de Candolle] 13(1): 89. 1852, non *Solanum cardiophyllum* Lindl., 1848. Type: Cuba. Havana: “in Havana”, *B.M. Boldo* s.n. (holotype: MPU).
Solanum scandens Sessé & Moc., Fl. Mexic., ed. 2, 53. 1894. Type: Cuba. Sin. loc., *Sessé & Mociño* s.n. (lectotype, designated by Knapp 2008b, pg. 20: MA [MA-604676, F neg. 48318]; isolectotype: MA [MA-604675, F neg. 48343, 5381, upper frag. in photo, sheet now divided]).

##### Type:

Based on *Solanum cardiophyllum* Dunal, non *Solanum cardiophyllum* Lindl., 1848.

##### Description.

Woody vining shrub or liana. Stems flexuous, glabrous; new growth minutely pubescent, the trichomes ca. 0.4 mm, simple, uniseriate, later glabrous. Bark of older stems dark reddish brown. Sympodial units plurifoliate. Leaves simple or occasionally very shallowly 3-lobed, 4.5–7 cm long, 3–5 cm wide, ovate to cordate or narrowly cordate, glabrous on both surfaces or with a few minute uniseriate simple trichomes along the midrib above; primary veins 5–7 pairs, drying reddish; base cordate or truncate and oblique; margins entire; apex acuminate; petioles 2.3–3.5 cm long, glabrous, twisting to aid in climbing up supports. Inflorescences leaf-opposed or terminal, 10–30 cm long, ovoid to ellipsoid in overall shape, branching to 5 times, with 50–100 flowers, glabrous; peduncle 1.5–9 cm long, glabrous; pedicels slender, 1.2–1.7 cm long, ca. 0.5 mm in diameter at the base, ca. 1 mm in diameter at the apex, nodding, glabrous, articulated in distal quarter just below the calyx tube leaving an elongate peg, occasionally articulated in the basal half of the pedicel, but always leaving a distinct remnant; pedicel scars widely spaced ca. 1 cm apart, appearing as a series of elongate pegs due to articulation point. Buds globose and somewhat inflated, the corolla strongly exserted from the calyx tube. Flowers all perfect, 5-merous. Calyx tube 3.5–4 mm long (to articulation), the upper part 2–2.5 mm long atop a conical receptacle-like structure, the lobes absent or mere undulations on the rim of the tube, glabrous. Corolla 2–2.3 cm in diameter, purple or violet, stellate, lobed ca. 3/4 of the way to the base, the lobes 0.8–1 mm wide, 0.4–0.6 cm wide, planar (or slightly cupped?) at anthesis, glabrous, the margins and cucullate tips densely papillose. Filament tube < 0.1 mm, glabrous; free portion of the filaments ca. 0.75 mm long; anthers 4–4.5 mm long, 2–2.5 mm wide, stout, poricidal at the tips, the pores lengthening to slits with age. Ovary conical, glabrous; style 0.8–0.9 cm long, glabrous, the stigma capitate, the surface minutely papillose. Fruit a globose berry, 1–1.2 cm in diameter, red when ripe, the pericarp thin and shiny; fruiting pedicels 1.1–1.3 cm long, ca. 0.5 mm in diameter at the base, ca. 1 mm in diameter at the apex, not particularly woody, deflexed, the basal portion of the calyx tube expanding in fruit to be clearly differentiated above the articulation point, appearing somewhat swollen. Seeds ca. 10 per berry, 3–3.5 mm long, 1.5–2 mm wide, flattened reniform, pale brown, the surfaces minutely pitted, the testal cells sinuate in outline. Chromosome number: not known.

**Figure 22.**
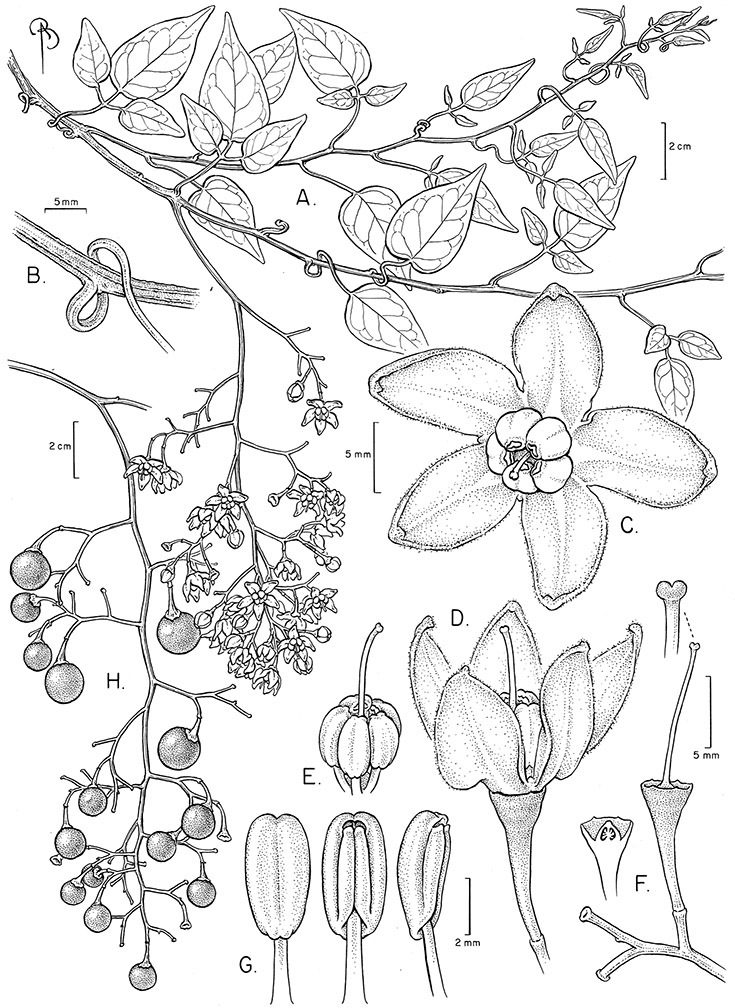
*Solanum boldoense* Dunal & A.DC. (**A–G** drawn from *Clark 10597* and J. Clark photographs taken in the field **H** drawn from *Wright 381*, NY). Illustration by Bobbi Angell.

##### Distribution

**Figure 23.**
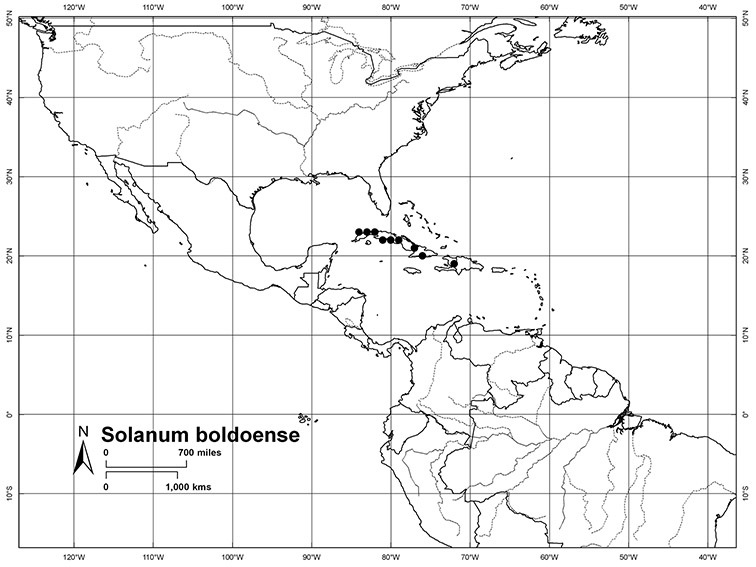
Distribution of *Solanum boldoense* Dunal & A.DC.

(Figure 23). Known only from Cuba and a single collection from Haiti, at low to middle elevations. Several very old single collections seen from Jamaica (K000196458) and Puerto Rico (*Plée* s.n., P00549340) may mislabelled as to locality or from cultivated plants.

##### Ecology.

A relatively rare plant in forests and along forest edges.

##### Conservation status.

Possible Near Threatened (possible NT); EOO <100,000 km^2^ (possible NT) and AOO <100,000 km^2^ (LC). See Moat (2007) for explanation of measurements.

##### Discussion.

*Solanum boldoense* is very similar to, and has been confused with (see below), *Solanum dulcamaroides* of Mexico. The pedicel articulation point serves to easily distinguish the two species: in *Solanum boldoense* it is the distal quarter of the length and long pegs are left when flowers fall, while in *Solanum dulcamaroides* the pedicel articulates at or very near the base.

Dunal used the name *Solanum cardiophyllum* for this species, but before publication realised it had already been used by Lindley for a species of potato. His replacement name is based on the same type, collected by B.M. Boldo near Havana.

Considerable confusion exists over the use of the epithet “scandens” in Sessé and Mociño’s works, but it is clear that on page 53 of *Flora Mexicana*, they were using *Solanum scandens* in the sense of a new name, different from that of *Solanum scandens* L., which they re-named *Solanum nutans*. The specimen (MA-604676) labelled “S.scandens IC.” in the Sessé and Mociño herbarium at MA with the locality “Hava. et Queretaro” demonstrates the confusion over the identity of all these plants. MA-604676 is a specimen of *Solanum boldoense*, suggesting [confirming?] the localities were added to the labels and the labels to the specimens after the fact, and that botanists in Madrid were not treating these two species are different. Another sheet of the same plant, MA-604675, appears to be from the same gathering. The “IC.” referred to on the label of MA-604676 may be plate 6331.1503 of the Torner Collection, that was used by Dunal to describe *Solanum dulcamaroides* (see discussion below). The plate is only partly finished and could be either species, but the pedicel articulation near the rhachis suggests it is *Solanum dulcamaroides*; epitype material is selected in the treatment of *Solanum dulcamaroides* to fix the usage this name.

##### Specimens examined.

**Cuba**. **Havana**: Havana, *Delessert* s.n. (G); Havana, 1841, *Karwinski* s.n. (LE); **Las Tunas**: In Cuba Orientali [note on US sheet - “fl. purple, Cliffs, Rio Sta. Cruz, July”], 1856, *Wright 381* (G, G, GH, GOET, K, LE, MO, S, US); **Las Villas**: Gavinas, Trinidad Mountains, San Blas-Buenos Aires, 18 Sep 1941, *Gonzales 177* (GH); **Pinar del Río**: San Diego de los Baños, 31 Aug 1910, *Britton et al. 6674* (F, US); La Palma, Pan de Guajaibón, trail on or near summit of Pan de Guajaibón, 700 m, 20 Dec 2008, *Clark et al. 10597* (BM); San Cristobal, Loma del Pimiento, 29 Sep 1920, *Ekman* s.n. (S); on the road between Mameyar and El Toro, 14 Sep 1923, *Ekman 17544* (S); Santa Cruz de los Pinos, vicinity of Taco-Taco River, 29 Oct 1925, *Brother León 12543* (GH); **Santa Clara**: Las Lagunas, Buenos Aires, 762 m, 5 Dec 1928, *Jack 6823* (A).

**Haiti**. La Hotte, between La Cueva and Placer Bonita, 1067 m, 1 Aug 1950, *Howard 12255* (GH).

**Jamaica**. Sin. loc, *Anonymous* s.n. (K, LE).

**Puerto Rico**. “Antilles. Porto Rico”, *Plée* s.n. (P).

#### 9. Solanum calileguae

Cabrera, Hickenia 1: 162. 1978

http://species-id.net/wiki/Solanum_calileguae

Figure 24

##### Type.

Argentina. Jujuy: Dpto. Valle Grande, road to Altos de Calilegua, 31 Oct 1974, *A.L. Cabrera, N.B. Deginani, A. Giaioti, R. Kiesling, E. Zardini & F.O. Zuloaga 25639* (holotype: SI; isotype: LP [n.v.]).

##### Description.

Woody vine. Stems scandent, glabrous to pubescent with a mixture of transparent simple and dendritic uniseriate trichomes to 1 mm long; new growth densely pubescent with simple and mostly dendritic trichomes. Bark of older stems yellowish brown. Sympodial units plurifoliate. Leaves simple to deeply 5-lobed, the lower lobes complete and the leaves apparently pinnate, 5.5–9 cm long, 2.5–3.5 cm wide, narrowly elliptic, the divided leaves to 10 cm long, 10 cm wide, elliptic in outline, membranous, the upper surfaces sparsely pubescent on the veins and lamina with dendritic 3–4-celled trichomes to 0.5 mm long, the lower surfaces densely pubescent along the veins with dendritic trichomes to 0.5 mm long like those of the upper surface, these sometimes extending to the lamina; primary veins 7–9 pairs; base truncate, oblique and asymmetric; margins entire or lobed, the lobes to 5 cm long, 2 cm wide; apex acute; petioles (1-)1.5–3(-6+) cm long, sparsely to densely pubescent with dendritic trichomes, especially adaxially, twining. Inflorescences terminal, later lateral, to 13 cm long, many times branched, with up to 30+ flowers, sparsely to densely pubescent with a mixture of simple and dendritic uniseriate trichomes to 0.5 mm long; peduncle to 8 cm long; pedicels 0.9–1.1 cm long, ca. 0.5 mm in diameter at the base, ca. 1 mm in diameter at the apex, slender, spreading at anthesis, sparsely to densely pubescent with a mixture of simple and dendritic trichomes like those of the inflorescence axis, articulated at the base from a small sleeve, leaving a small peg on the inflorescence axis; pedicel scars irregularly spaced up to 9 mm apart. Buds broadly ellipsoid, the corolla about halfway exerted from the calyx before anthesis. Flowers all perfect, 5-merous. Calyx tube 2–3 mm long, conical, irregularly splitting into lobes 2–4 mm long, densely pubescent with dendritic trichomes, these denser on the tube. Corolla 2–3 cm in diameter, white, stellate-rotate, lobed 1/2 to 2/3 of the way to the base, the lobes 9–12 mm long, 4–5 mm wide, planar at anthesis, densely pubescent abaxially with minute simple trichomes to 0.5 mm long, pubescent adaxially along the distal half of the midvein with minute simple trichomes to 0.5 mm long, the tips cucullate. Filament tube minute, the free portion of the filaments 0.5–1 mm long, minutely puberulent within with tangled, weak simple uniseriate trichomes; anthers ca. 5 mm long, 2 mm wide, ellipsoid, yellow, loosely connivent, poricidal at the tips, the pores lengthening to slits with age. Ovary glabrous; style ca. 9 mm long, densely pubescent in the basal half with weak, tangled simple uniseriate trichomes; stigma minutely clavate, the surface minutely papillose. Fruit a globose berry, ca. 1 cm in diameter (fide Cabrera 1983). Seeds not seen from mature berries.

**Figure 24.**
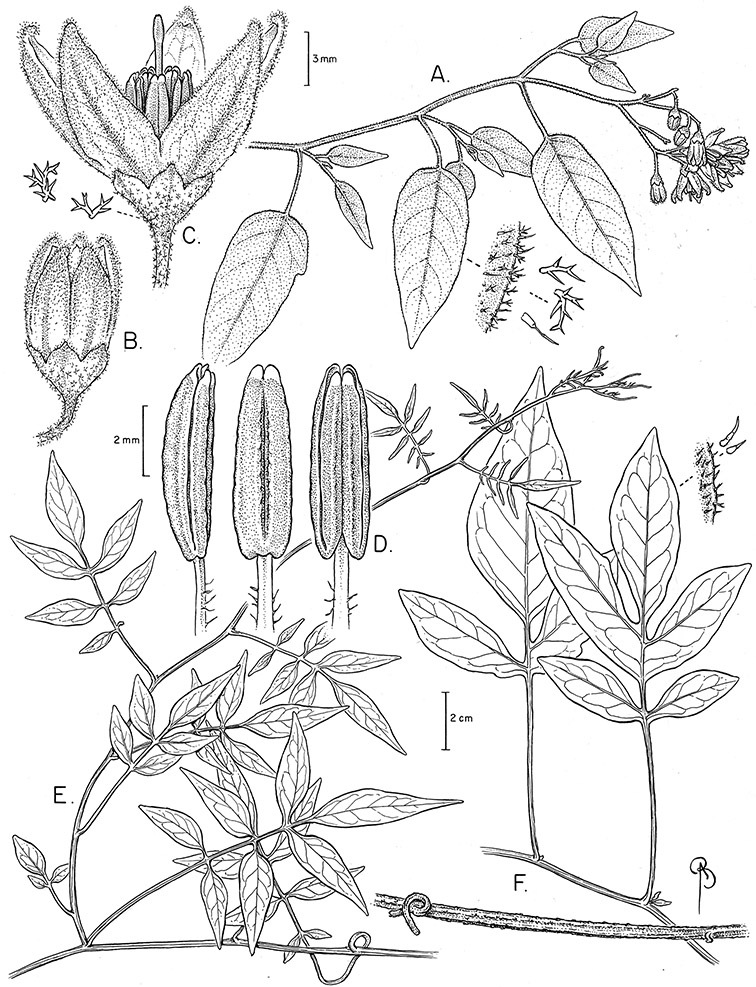
*Solanum calileguae* Cabrera.(**A–D** drawn from *Nee & Bohs 50809* **E** drawn from *Nee & Bohs 50768*). Illustration by Bobbi Angell.

##### Distribution

**Figure 25.**
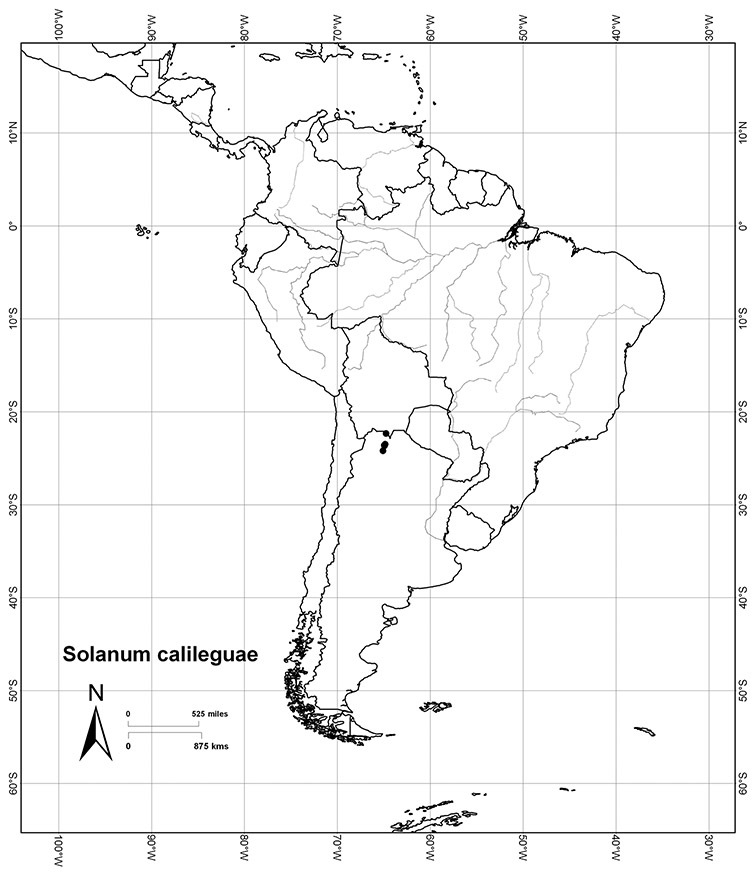
Distribution of *Solanum calileguae* Cabrera.

(Figure 25). Known only from the foothills of the Andes in the Provinces of Jujuy and Salta, Argentina, 1600–1850 m; possibly also occurring (sterile voucher only) in adjacent Bolivia.

##### Ecology.

In premontane forests dominated by *Juglans* L. (Juglandaceae) and *Podocarpus* Pers. (Podocarpaceae).

##### Conservation status.

Endangered (EN); EOO <5,000 km^2^ (EN) and AOO <2,000 km^2^ (VU). See Moat (2007) for explanation of measurements.

##### Discussion.

*Solanum calileguae* is a distinctive species with its dense dendritic pubescence and large, white flowers. It has a restricted Andean distribution, and is easily distinguished from other members of the group potentially occurring in the area. It differs from *Solanum uncinellum*, which also has dendritic pubescence on the stems and leaves, in its ellipsoid (rather than long pointed) buds, its rotate-stellate rather than deeply stellate flowers and in its anthers borne on equal filaments. *Solanum uncinellum*, although widespread over most of South America, has only once been collected in the Andean foothills of Argentina, but not from the same area where *Solanum calileguae* occurs. *Solanum calileguae* is most similar and perhaps most closely related to *Solanum flaccidum*, from which it differs in the dendritic pubescence and anthers borne on equal filaments. *Solanum flaccidum* occurs in southeastern Brazil, and the two species are unlikely to be found together in the field.

##### Specimens examined.

**Argentina**. **Jujuy**: Ledesma, Abra de las Cañas, camino a Velle Grande, 24 km NW de Libertador Gral. S. Martín, 1600 m, 8 Nov 1974, *Krapovickas et al. 26583* (G, MO); Palpalá, Cerro Zapla, above Quebrada Los Tomates, on road to Cerro Zapla, 1850 m, 13 Apr 2000, *Nee et al. 50756* (CORD, NY); Valle Grande, 2 km N of Abra de Cañas on road from Calilegua to Valle Grande, 14 km by road S of San Francisco, 1600 m, 14 Apr 2000, *Nee & Bohs 50768* (CORD, MO, NY); Valle Grande, ca. 1 km (by air) E on trail from San Francisco towards Altos de Calilegua, 1700 m, 18 Apr 2000, *Nee & Bohs 50809* (BM, MO); Valle Grande, ca. 1 km (by air) E on trail from San Francisco towards Altos de Calilegua, 1700 m, 18 Apr 2000, *Nee 50809* (MO); **Salta**: Santa Victoria, camino al Río San José, desde el desvio del camino de Los Toldos a Lipeo, 1823 m, 28 Sep 1998, *Ahumada & Agüero 8178* (CORD).

#### 10. Solanum coalitum

S.Knapp, Novon 17: 212. 2007

http://species-id.net/wiki/Solanum_coalitum

Figure 26

##### Type.

Ecuador. Loja: Yangana-Valladolid, km 1.1, track to Sierra Toledo, km 18.5, 3250 m, c. 4°23'S, 79°06'W, 14 Nov 1997, *G. Lewis & B. Klitgaard 3719* (holotype: LOJA; isotypes: AAU, BM [BM000846493], K [K000585495], NY [NY00888405], QCA, QCNE [QCNE-740]).

##### Description.

Subshrubs to 1 m tall, sometimes scandent and trailing. Stems glabrous and shining, usually appearing warty from the prominent leaf scars; young stems and leaves completely glabrous or sometimes with a few scattered loose white branched trichomes to 0.5 mm long. Bark of older stems dark brown, shining. Sympodial units plurifoliate. Leaves simple, 2.5–10.4 cm long, 0.7–3.5 cm wide, narrowly elliptic to less commonly elliptic, the upper surfaces glabrous and shiny, sometimes with scattered branched trichomes at the edge where the margin is revolute, the lower surfaces glabrous or sparsely papillate, the papillae drying reddish brown, perhaps glandular; primary veins 5–10 pairs, drying darker than the lamina; base acute to attenuate; margins strongly revolute, pubescent on the upper surfaces where turned under; apex acute; petiole 0.3–1.6 cm long, glabrous and shiny. Inflorescences terminal, 2.5–6 (-10) cm long, branched 4–6 times, with 3–15(-20) flowers, glabrous and shining, or with scattered loosely branched trichomes along the axes; peduncle 2–5.5 cm long; pedicels in flower 0.8–1.3 cm long, stoutish, ca. 1 mm in diameter at the base, ca. 2 mm in diameter at the apex, nodding, glabrous, minutely papillate or sparsely pubescent with loosely branched white trichomes ca. 0.3 mm long, articulated at the base and inserted in a short sleeve ca. 1 mm long; pedicel scars closely spaced and clustered at inflorescence branch tips. Buds globose when very young, soon elliptic and strongly exerted from the calyx tube. Flowers all perfect, 5-merous. Calyx tube 2.5–4 mm long, cup-shaped, strongly constricted at the base, thick and coriaceous, glabrous or with a few branched trichomes like those of the inflorescence axis, the lobes 1–1.5 mm long, broadly deltate or minute, glabrous, with the margins glabrous or densely pubescent with branched trichomes ca. 0.3 mm long. Corolla 2–2.6 cm in diameter, violet to dark mauve-purple, lobed 3/4 of the way to the base, stellate, the lobes 0.9–1.3 mm long, 0.5–0.8 cm wide, slightly campanulate or planar at anthesis, densely pubescent with simple or dendritic trichomes ca. 0.5 mm long on the margins and tips, sometimes with scattered simple trichomes on the abaxial lobe surface, these denser on the petal midvein, the adaxial surface glabrous. Filament tube less than 0.5 mm long; free portion of the filaments 1–1.5 mm long, glabrous; anthers 5–6 mm long, 1.5–2 mm wide, loosely connivent, poricidal at the tips, the pores lengthening to slits with age. Ovary conical, glabrous; style 1–1.2 cm long, straight, glabrous; stigma clavate or 2-lobed, bright green (fide *Lewis & Klitgaard 3719*), the surface minutely papillate. Fruit a globose berry, 1.2–1.5 cm in diameter, shiny and black when mature, the pericarp thin; fruiting pedicels 2–2.2 cm long, 2–2.5 mm in diameter at the base, 2–3 mm in diameter at the apex, erect, thick and woody. Seeds ca. 10 per berry, 4–5 mm long, 3–4 mm wide, flattened reniform, reddish brown, the margins not enlarged, the surfaces minutely pitted, the testal cells sinuate in outline. Chromosome number unknown.

**Figure 26.**
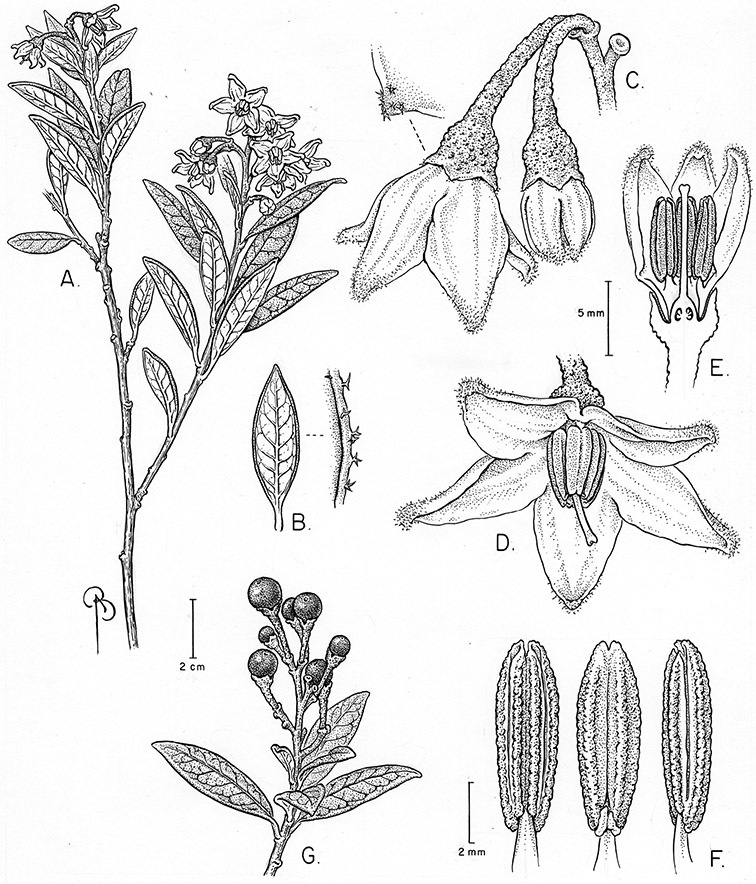
*Solanum coalitum* S. Knapp. (**A, B** drawn from *Jorgensen 2188* **C** D drawn from G. Lewis photographs of *Lewis 3179*). Illustration by Bobbi Angell.

##### Distribution

**Figure 27.**
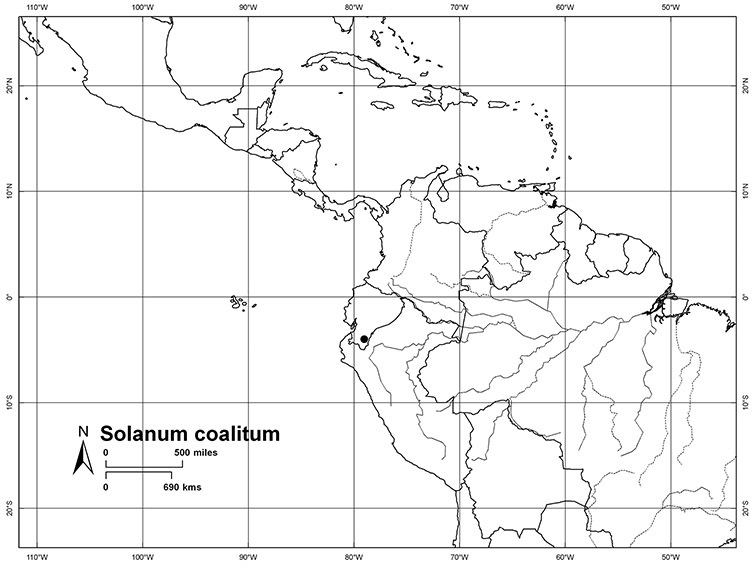
Distribution of *Solanum coalitum* S.Knapp.

(Figure 27). Endemic to Ecuador, only known from the páramo of Cerro Toledo S of Loja along the road leading to the Peruvian border, on ridges between the towns of Yangana and Valladolid, at 3150-3460 m in southwestern corner of Parque Nacional Podocarpus.

##### Ecology.

Páramos and forest edges.

##### Conservation status.

Critically Endangered (CR); EOO <100 km^2^ (CR) and AOO <10 km^2^ (LC). See Moat (2007) for explanation of measurements. Knapp (2007c) gave a preliminary conservation status of EN (endangered) or critically endangered for this species. The fact that *Solanum coalitum* occurs only within the boundaries of the Parque Nacional Podocarpus is good news for its ultimate protection and conservation, but its very restricted distribution in an isolated habitat means it certainly is of concern.

##### Discussion.

*Solanum coalitum* is a striking species with its large, fleshy bright purple flowers and black fruits. Some specimens of *Solanum coalitum* have been identified as *Solanum stenophyllum*, with which it is very similar. *Solanum coalitum* differs from *Solanum stenophyllum* in its subshrubby, sometimes trailing habit, its glabrous stems and leaves (except for the peculiar marginal trichome band), its cup-shaped (rather than conical) calyx and its slightly larger flowers that are glabrous abaxially. Trichomes of *Solanum coalitum* when they occur are looser and more openly dendritic than the almost echinoid trichomes that are distinctly yellowish of *Solanum stenophyllum*. The fruiting pedicels of *Solanum stenophyllum* appear to be nodding when fruit are mature, while those of *Solanum coalitum* are erect. Specimens of *Solanum stenophyllum* have been collected from the province of Loja (i.e., *Jørgensen et al. 477, 1068*, BM) from further north and at slightly lower (2600-3000 m) elevations than *Solanum coalitum* and in drier and/or degraded forests. *Solanum stenophyllum* grows as a shrub or small treelet, usually in disturbed situations. Some individuals of *Solanum stenophyllum* in southern Ecuador are very sparsely pubescent, but the conical calyx and yellowish closely branched trichomes serve to distinguish these plants.

*Solanum coalitum* is distinguished from the very similar *Solanum imbaburense* by its broadly deltate, rather than long triangular calyx lobes, and its leaves with sparsely papillate undersides.

The sole locality in which *Solanum coalitum* has been encountered is the páramo of Cerro Toledo in the extreme southwestern corner of Parque Nacional Podocarpus, one of the largest protected areas in Ecuador. Cerro Toledo is a mixed páramo of tussock grasses and shrubby vegetation on the divide of the Cordillera de Sabanillas; the area is a pathway used by local people to take cattle from one drainage to another, and as such has a medium level of disturbance (Hofstede et al. 2002). Roads constructed by the military to allow access to radio towers have opened the area to others. Hofstede et al. (2002) suggest that the inhospitable nature of the climate in the region (wet, cold and windy) will limit human incursion on a large scale. Cerro Toledo is isolated from other páramo regions of southern Ecuador, and represents one of the southernmost extensions of the páramo habitat in the Andes (Luteyn 1999).

##### Specimens examined.

**Ecuador**. **Loja**: Yangana, Cerro Toledo, ascending road from Yangana to Numbala toward the antennas. Area next to antennas at summit of cerro, 3400 m, 27 Mar 2005, *Bohs et al. 3320* (BM, LOJA, QCNE); carretera Yangana-Toledo, 3420 m, 28 Dec 1988, *Jaramillo 10606* (AAU); road from Yangana to Cerro Toledo, km 18-22 to antennas, 3460 m, 3 Nov 2000, *Jørgensen et al. 2188* (BM, NY); Yangana-Cerro Toledo, páramo of Cerro Toledo, 3420 m, 28 Dec 1988, *Jørgensen et al*. (AAU, BM); Cerro Toledo, Parque Nacional Podocarpus, 3350 m, 1 Dec 1988, *Madsen et al. 75641* (BM); Cerro Toledo, 2500 m, 30 Oct 1989, *Madsen 86333* (BM); Cerro Toledo, E of Yangana, Parque Nacional Podocarpus, 3400 m, 26 Feb 1985, *Øllgaard et al. 58162* (BM).

#### 11. Solanum crispum

Ruiz & Pav., Fl. Peruv. 2: 31, t. 158a. 1799

http://species-id.net/wiki/Solanum_crispum

Figure 28

Solanum ligustrinum Lodd., Bot. Cab. 1963. 1833. Type: Chile. Sin. loc., seeds sent by H. Cuming in 1831 (lectotype, designated here: Loddiges, Bot. Cab. No. 1963. 1833).
Solanum concavum Lindl., Edwards’s Bot. Reg. 28(Misc.): 57. 1842. Type: Chile. sin. loc., *H. Cuming 263* (lectotype, designated here: CGE; isolectotypes: BM [BM000935822], E [E00057570], GH [GH00077603], K [K000585719]).
Solanum laetum Kunze, Linnaea 16: 352. 1842. Type: Germany. Cultivated in Leipzig, 1837, *Anon.* s.n. (no specimens cited or found; synonymy ex descr.).
Solanum syringaefolium Kunth & C.D.Bouché, Ind. Sem. Hort. Berol. 10. 1845. Type: Chile. sin. loc., *T.C. Bridges* s.n. (holotype: B, destroyed [F neg. 2746]).
Witheringia berteroana J.Rémy, in Gay, Fl. Chil. 5: 65. 1849, as “*berterianum*” Type: Chile. Tagua-Tagua, *C.G.L. Bertero* s.n. (lectotype, designated by Knapp 1989, pg. 74: P [P00324728]).
Witheringia crispa (Ruiz & Pav.) J.Rémy, in Gay, Fl. Chil. 5: 63. 1849. Type: Based on *Solanum crispum* Ruiz & Pav.
Witheringia gayana J.Rémy, in Gay, Fl. Chil. 5: 67. 1849. Type: Chile. sin. loc., *C. Gay* s.n. (lectotype, designated by Knapp 1989, pg. 74: P [P00335390]; isolectotype: K [K000585718]).
Witheringia tomatillo J.Rémy, in Gay, Fl. Chil. 5: 64. 1849. Type: Chile. sin. loc., *C. Gay* s.n. (lectotype, designated by Knapp 1989, pg. 74: P [P00325721]; isolectotype: K [K000585722]).
Solanum angustifolium Lam. var. *brevifolium* Dunal, Prodr. [A.P. de Candolle] 13(1): 90. 1852. Type: Chile. Sin. loc., 1828, *E. Poeppig 74 [diar. n. 34]* (holotype: G-DC [G00144942, Morton neg. 8402, IDC microfiche 800-61.2068:II.7]; isotypes: BM [BM000935826], LE, P [P00319643, Morton neg. 8145]).
Solanum congestiflorum Dunal, Prodr. [A.P. de Candolle] 13(1): 92. 1852. Type: Chile. Aviluco, *C.G.L. Bertero 634* (lectotype, designated by Knapp 1989, pg. 74: P [P00325722]; isolectotype: MO [MO-3461608]).
Solanum congestiflorum Dunal var. *longifolium* Dunal, Prodr. [A.P. de Candolle] 13(1): 92. 1852. Type: Chile. Región × (Los Lagos): Valdivia: “in Chile australis provincia Valdivia”, *C. Gay herb. 3e envoi 211* (lectotype, designated by Knapp 1989, pg. 74: P [P00325724]).
Solanum crispum Ruiz & Pav. var. *elaeagnifolium* Dunal, Prodr. [A.P. de Candolle] 13(1): 92. 1852. Type: Chile. Región VI (Liberador): Prov. O’Higgins: circa Rancagua, 1833, *C.G.L. Bertero 640* (lectotype, designated by Knapp 1989, pg. 74: G-DC [G00144948, Morton neg. 8406]; isolectotype: P [P00325727**,** Morton neg. 8175]).
Solanum crispum Ruiz & Pav. var. *ligustrinum* (Lodd.) Dunal, Prodr. [A.P. de Candolle] 13(1): 92. 1852. Type: Based on *Solanum ligustrinum* Lodd.
Solanum pyrrhocarpum Phil., Anales Univ. Chile 21(2): 383. 1862. Type: Chile. Región VIII (Bío-Bío): Prov. Ñuble: Chillan, *sin. coll*. (lectotype, designated by Knapp 1989, pg. 74: SGO [SGO-55460, barcode SGO000004551]).
Solanum sadae Phil., Linnaea 33: 203. 1864–1865. Type: Chile. Región VI (O’Higgins): Colchagua, *sin. coll*. (lectotype, designated by Knapp 1989, pg. 74: SGO [SGO-55450, barcode SGO000004595]; isotype: E).
Solanum landbeckii Phil., Linnaea 33: 204. 1864–1865. Type: Chile. Región VI (O’Higgins): Colchagua, *C.L. Landbeck* s.n. (lectotype, designated by Knapp 1989, pg. 74: SGO).
Solanum izquierdii Phil., Anales Univ. Chile 43: 522. 1873. as “*izquierdi*” Type: Chile. Región Metropolitana: Prov. Santiago, Aculeo (ca. 33°50'S, 70°55'W), *V. Izquierdo* s.n. (lectotype, designated by Knapp 1989, pg. 74: SGO [SGO-55455, barcode SGO000004573]; isolectotype: K [K000585727]).
Solanum gayanum (J.Rémy) F.Phil., Cat. Pl. Vasc. Chil. 228. 1881. Type: Based on *Witheringia gayana* J.Rémy
Solanum tomatillo (J.Rémy) F.Phil., Cat. Pl. Vasc. Chil. 229. 1881. Type: Based on *Witheringia tomatillo* J.Rémy
Solanum pugae Phil., Anales Univ. Chile 91: 7. 1895. Type: Chile. Región VIII (Bío-Bío): Prov. Ñuble, Cerro Centinela, *F. Puga* s.n. (holotype: SGO, specimens not located).
Solanum pannosum Phil., Anales Univ. Chile 91: 9. 1895. Type: Chile. Región VII (Maule): Prov. Curicó, Los Maquis, ca. 35°43'S, 70°47'W, *M. Vidal* s.n. (holotype: SGO, specimens not located; isotype: W [W-1908-10271]).
Solanum berteroanum (J.Rémy) Phil., Anales Univ. Chile 91: 8. 1896. Type: Based on *Witheringia berteroana* J.Rémy
Solanum tagua Kuntze, Revis. Gen. Pl. 3(2): 226. 1898. Type: Based on *Witheringia berteroana* J.Rémy
Solanum congestiflorum Dunal var. *syringaefolium* (Kunth & Bouché) Reiche, Anales Univ. Chile 123: 721. 1908. Type: Based on *Solanum syringaefolium* Kunth & C.D.Bouché
Solanum congestiflorum var. *pannosum* (Phil.) Reiche, Anales Univ. Chile 123: 722. 1908. Type: Based on *Solanum pannosum* Phil.

##### Type.

Chile. Región VIII (Bío-Bío): Concepción: “in Chile ruderatis copiosé in Conceptionis urbis sepibus, et ad Carcamo et Palomares tractus”, *H. Ruiz & J. Pavón* s.n. (lectotype, designated by Knapp 2008c, pg. 312: MA [MA-747012]; isolectotype: MA [MA-747101]).

##### Description.

Shrubs or small trees, often lax and scrambling, 0.4–5 m tall. Stems glabrous or pubescent with tiny dendritic trichomes; leaf scars somewhat prominent; new growth glabrous to densely pubescent with fine, dendritic trichomes. Bark of older stems pale brownish-yellow, glabrous, and shiny. Sympodial units plurifoliate. Leaves simple, 2.7–7.5 (10) cm long, 1–3 (7) cm wide, ovate to narrowly ovate, occasionally somewhat elliptic, larger and broader in plants growing in shade and in juvenile plants (see discussion), the adaxial surfaces glabrous or with a few dendritic trichomes along the main veins, the abaxial surfaces glabrous or puberulent with dendritic trichomes, these usually denser along the veins; primary veins 6–12 pairs, pubescent below; base truncate or somewhat cordate, not winged on to the petiole; margins entire, undulate or crispate; apex acute to acuminate; petiole 0.5–1 (2.2) cm long. Inflorescences terminal, later appearing lateral from overtopping of shoots, 2–10 cm long, flat-topped or pyramidal, branching 5–7 times, with 10–20 flowers, glabrous or sparsely pubescent with dendritic trichomes like those of the stems and leaves; peduncle 1–5 cm long; pedicels 1–1.3 cm long, tapering from a basal diameter of ca. 0.5 mm to an apical diameter of ca. 1 mm, nodding at anthesis, glabrous or with a few scattered dendritic trichomes, articulated at the base and inserted in a sleeve ca. 0.5 mm long; pedicel scars closely spaced in clusters. Buds globose when young, later elliptic, the corolla strongly exserted from the calyx tube. Flowers all perfect, 5-merous. Calyx tube 1–1.5 (2) cm long, conical, the lobes 0.5–1 mm long, deltate to long-triangular, glabrous or with a few scattered dendritic trichomes abaxially, adaxially glabrous. Corolla 1.2–2.5 cm in diameter, violet or occasionally white, lobed 3/4 to 7/8 of the way to the base, the lobes 5–9 mm long, 3–5 mm wide, planar or somewhat reflexed at anthesis, densely pubescent with simple (in glabrous plants) or dendritic (in pubescent plants) trichomes abaxially, the trichomes denser at the tips of the lobes, glabrous adaxially. Filament tube less than 0.5 mm long; free portion of the filaments 1–1.5 mm long, glabrous; anthers 3.5–5 mm long, 1–2 mm wide, loosely connivent, poridical at the tips, the pores becoming slit-like with age. Ovary glabrous or with a few dendritic and simple trichomes at the apex, especially in otherwise pubescent plants; style 0.6–1 cm long, pubescent with dendritic or simple trichomes along the entire length; stigma clavate or capitate, the surface minutely papillose. Fruit a globose or ellipsoid berry, 0.8–1 cm in diameter, bright red when ripe, changing from green to yellow or orange during ripening, with thin pericarp; fruiting pedicels 1.2–1.6 cm long, ca. 1 mm in diameter at the base, woody, deflexed. Seeds ca. 11 per berry, 2–3 mm long, 1.5–2 mm wide, flattened lenticular, reddish-brown, the surfaces minutely pitted. Chromosome number: n = 12 (vouchers: *Knapp 8632, Knapp 8633* cultivated material).

**Figure 28.**
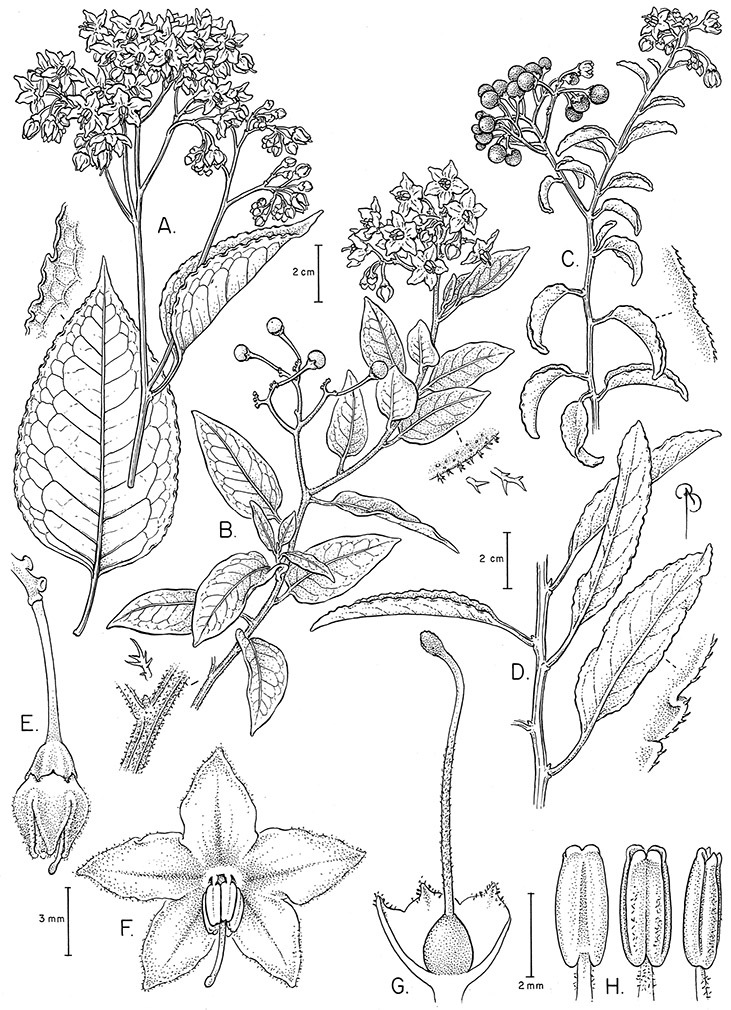
*Solanum crispum* Ruiz & Pav. (**A** drawn from *Taylor 10235* **B** drawn from *Nee 54654* **C** drawn from *Landrum 8216* **D** drawn from *Biese 78* **E–H** drawn from *Landrum 4459*). Illustration by Bobbi Angell.

##### Distribution

**Figure 29.**
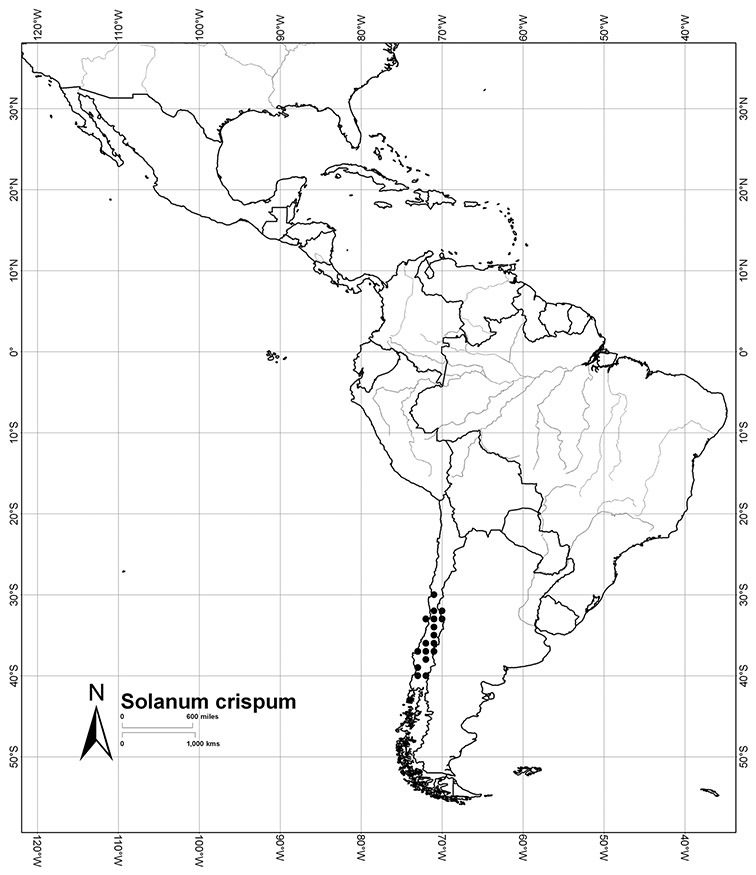
Distribution of *Solanum crispum* Ruiz & Pav.

(Figure 29). Chile from Quillota south to the island of Chiloé, from 10–2500 m elevation. *Solanum crispum* is also known from scattered collections in Argentina along the border with Chile; in the Neuquén area these plants are usually found in association with villages and it is suspected that rather than being native, they have been brought from Chile and cultivated for their medicinal properties (C. Ezcurra, pers. comm.).

##### Ecology.

*Solanum crispum* grows in *Nothofagus* (Nothofagaceae) forest, often in second growth, and in a wide variety of moist microsites in otherwise dry habitats.

##### Common names.

Chile: tomatillo, natre, natrien, natri, tomatilla (see Knapp 1989).

##### Conservation status.

Least Concern (LC); EOO >50,000 km^2^ (LC) and AOO >5,000 km^2^ (LC). See Moat (2007) for explanation of measurements.

##### Discussion.

*Solanum crispum* is one of the most variable and is the most southerly species of the *Solanum nitidum* species group, occurring to 43 degrees S latitude. It also has a huge elevational range, occurring from sea level to nearly 3000 m in a wide variety of habitats. Two pubescence forms occur throughout the range of *Solanum crispum*: glabrous plants were traditionally called *Solanum crispum* and pubescent ones *Solanum congestiflorum* (see Knapp 1989). Specimens of intermediate pubescence are rare, but the new growth of glabrous plants is always dendritic-pubescent. In a cladistic analysis (Knapp 1989) the two forms were treated as separate, but were strongly resolved as sister taxa. Pubescence may be related to habitat, but this effect has not been studied in any detail; polymorphism in pubescence is extremely common in members of the Dulcamaroid clade and elsewhere in *Solanum*. The pubescent form often has larger, more repand leaves than does the glabrous form. This raises the intriguing possibility that the pubescent form is paedomorphic, retaining the shape and indument of juvenile leaves.

Medicinal uses of *Solanum crispum* have been recorded for over two centuries, beginning with Ruiz and Pavón in *Flora Peruviana* (1797), where the plant was reported to be used as a febrifuge. It has been reported as used against the fevers called ‘congo’ and ‘chavalongo’.

*Solanum crispum* has been cultivated in the United Kingdom since the early part of the 19th century. Specimens were introduced to Kew Gardens from the island of Chiloé (Chile) by Mr. Anderson (Hooker 1844) and a variety still in cultivation today was developed at the Glasnevin Botanic Gardens in Dublin. *Solanum crispum* is usually classed as a climber, but this is due to its lanky habit in the British climate. It does not possess the twining stems, petioles or tendrils of a true climber, but it is not a robust, erect plant.

Many monographers in *Solanum* have stated that holotypes or lectotypes for Ruiz and Pavón names were in the Madrid herbarium (MA), but without specifying a particular sheet. In a few cases, only one sheet exists, thus making lectotypification relatively straightforward, but in others multiple sheets exist in the Ruiz and Pavón herbarium at MA, meaning that previous type designations are not sufficiently precise. It is unlikely that any of these specimens are actually holotypes; the dispersal of specimens at the time of the expedition and subsequently through sale and loss means lectotypification is essential even if only a single sheet is present at MA. Knapp (1989) lectotypified *Solanum crispum* citing only a sheet in MA; this was rectified in 2008 by citation of the particular sheet (Knapp 2008c) as the lectotype.

**Figure 30.**
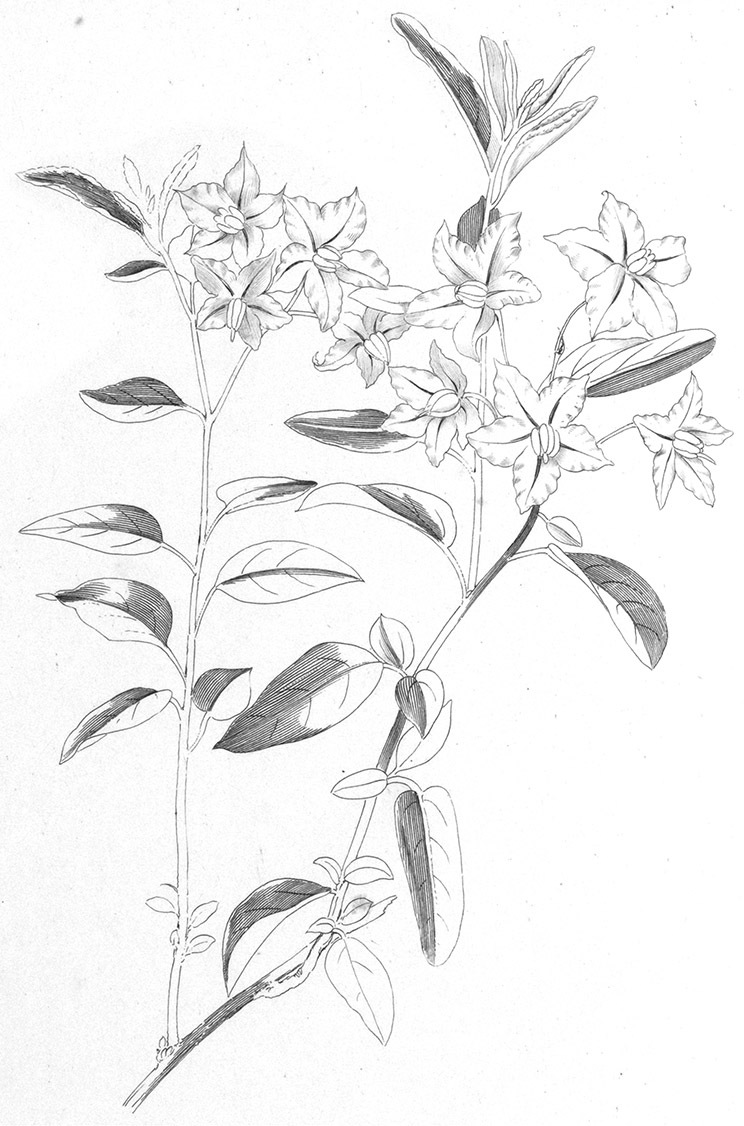
Lectotype of *Solanum ligustrum* Lodd. (Loddiges 1833: tab. 1963). Reproduced with permission of the Natural History Museum Botany Library.

Knapp (1989) erroneously lectotypified *Solanum ligustrinum* using one of the specimens cited by Dunal (1852) when making the new combination rather than the plate from Loddiges’ (1833) original description (in which seeds from an un-numbered collection sent by H. Cuming are cited). The epithet *ligustrinum* is correctly lectotypified here using Loddiges’ plate (Figure 30), as it is the only extant element unambiguously related to the protologue.

##### Specimens examined.

**Argentina**. **Mendoza**: Santa Rosa de los Andes to Uspallato Pass, *Moseley* s.n. (BM, LE); **Neuquén**: Minas, a 18 km de las Ovejas camino a las lagunas Epu-Lauquén, mallin de la Culebra, 1450 m, 14 Jan 1964, *Boelcke et al. 10786* (SI).

**Chile**. **Región IV (Coquimbo)**: Elqui, sector bordering road to Pachon, 38 km SE of Guard Control Post at Cerro Tololo, 2416 m, 16 Jan 2004, *Acosta-Solís & León BB 181* (K); Illapel, Dec 1862, *Landbeck* s.n. (W); Elqui, Baño de Pangue, 1800 m, 20 Sep 1947, *Sparre 2628* (S); Ovalle, Ovalle, ca. 86 km from Ovalle on road Caren to Río Mostazal, 1800 m, 11 Nov 1938, *Worth Morrison 16448* (G); **Región Metropolitana**: Rio Colorado, Paso Uspallata, 6 Jan 1886, *Philippi & Borchers* s.n. (BM); Santiago, Oct 1933, *Grandjot* s.n. (GOET); Melipilla, Las Vizcachas, ca. 10 km from La Dormida, 1920 m, 7 Dec 1938, *Morrison 16760* (G); Santiago, 1876, *Philippi* s.n. (G); Santiago, 1862, *Philippi* s.n. (G); Farallones, 2500 m, 15 Apr 1969, *Plowman 2683* (LE); ); Santiago, Cerro San Cristobal, Nov 1869, *Reed 1869* (BM); Santiago, Prov. Santiago, Cord de Santiago, Rio San Francisco, 2200 m, Dec 1924, *Werdermann 439* (BM); Río San Francisco, Cordillera de Santiago, 2200 m, Dec 1927, *Werdermann 479* (G); **Región V (Valparaíso)**: Santa Rosa de los Andes, May 1882, *Ball* s.n. (LE); Los Señales, Dec 1829, *Bertero 1323* (G-DC); Quillota, Aug 1829, *Bertero 1327* (G-DC); Llaillai, “Atacama”, 9 Oct 1884, *Borchers* s.n. (F); **Región VI (O’Higgins)**: prov. Colchagua, Cerro Echaurrina, San Fernando, 13 Oct 1926, *Montero 33 A* (F); Colchagua, 1862, *Philippi* s.n. (G); **Región VII (Maule)**: Talca, Cordillera de Los Andes: 18 km on road to Laguna del Maule from first border post, 4 Feb 1998, *Baxter et al. 26* (BM); Cordillera de Curicó, Valle del Toro, 1500 m, 1903, *Bürger 48* (GOET); **Región VIII (Bío-Bío)**: Baños de Chillán, Jan 1878, *Anonymous* s.n. (W); Concepción, 26 Sep 1939, *Bailey 941* (BH); Concepción, Quebrada Honda, entre Liriquén y Tomé, 50 m, 16 Oct 1986, *Basualto et al. 37* (MA); Ñuble, Termas de Chillán, Refugio El Aserradero, 1240 m, 13 Nov 1986, *Basualto et al. 156* (MA); Talcahuano, 12 Nov 1950, *Brooke 6951* (F); Biobío, Antuco, Cordillera de los Andes, Fundo Los Ciervos, passing into El Toro hydroelectric central and crossing the Río Polcura, 860 m, 28 Jan 2004, *Brownless et al. DCI-1045* (BM); Ñuble, Chillán, road to Termas Chillán at Puente Torrealba, 1514 m, 25 Dec 2003, *Gardner & Knees 6776* (BM); Ñuble, Chillán, 1856, *Germains.n.* (G); Prov. Ñuble, Chillán, 1856, *Germain* s.n. (F); Concepción, 1825, *Macrae* s.n. (G); Coronel, 1864, *Oschenius* s.n. (GOET); Chillán, *Philippi* s.n. (K); Ñuble, Termas de Chillán, 1450 m, 11 Dec 1987, *Rechinger & Rechinger 64317* (B); Ñuble, Río Ñuble, 40-50 km desde San Fabian hacia la Cordillera, entre rio Nuble y rio Los Sauces, 26 Feb 1968, *Zalensky III 81-82* (LE); **Región IX (Araucanía)**: Malleco, Victoria, Hotel El Bosque on Ruta 5 south-bound from Victoria, 338 m, 24 Jan 2004, *Brownless et al. DCI-903* (BM); Cautín, Villarrica, road from Meseta San Judas to western edge of Lago Colico, 500 m, 20 Dec 2003, *Gardner & Knees 6734* (BM); Malleco, road from Victoria to Termas de Tolhuaca before Parque Nacional Tolhuaca near to Puente Tacadero, 942 m, 30 Dec 2003, *Gardner & Knees 6870* (BM); Cautín, Río Zuerpe, 28 Sep 1905, *Middleton* s.n. (G x2); **Región X (Los Lagos):** Island of Chilóe, *Anonymous 7966* (BM); Island of Chilóe, *Anonymous 7967* (BM); Archipelago de Chiloe, *Downton 6* (BM); **Región XIV (Los Ríos)**: Valdivia, 20 Oct 1904, *Buchtien* s.n. (G); Valdivia, 22 Oct 1905, *Buchtien* s.n. (G); Valdivia, 28 Sep 1896, *Buchtien* s.n. (G); Chiloé, Chiloé, *Caldeleugh* s.n. (G); Valdivia, Valdivia, coastal road from Curiñanco to Niebla, 19 Dec 2003, *Gardner & Knees 6723* (BM); Valdivia, Panguipulli, 150 m, Oct 1924, *Hollermayer 324* (BM); Cordillera de Ranco, *Lechler 827* (GOET); Valdivia, road from La Union to El Mirador, 700 m, 30 Mar 1969, *Plowman 2640* (GH); Valdivia, Corral, Amargos, San Carlos, 20 m, 6 Jan 1954, *Sparre & Smith 399* (G); Valdivia, Panguipulli, 150 m, Oct 1924, *Werdermann 324* (G);

#### 12. Solanum cutervanum

Zahlbr., Ann. K. K. Naturhist. Hofmus. 7: 7. 1892

http://species-id.net/wiki/Solanum_cutervanum

Figure 31

Solanum angustifolium Ruiz & Pav., Fl. Peruv. 2: 33, t. 163b. 1799, non *Solanum angustifolium* Miller, 1768, nec *Solanum angustifolium* Lam., 1793. Type: Peru. Huánuco: Acomayo, *H. Ruiz & J. Pavón* s.n. (lectotype, designated by Knapp 2008c, pg. 310: MA [MA-747093]; isolectotypes: F, MA [MA-747094, MA-747095]).
Solanum pulverulentum Pers., Syn. 1: 223. 1805. Type: Based on *Solanum angustifolium* Ruiz & Pav., Fl. Peruv. 2: 33, t. 163b. 1799, non *Solanum angustifolium* Miller, 1768, nec *Solanum angustifolium* Lam., 1793.
Solanum aureum Dunal var. *angustelanceolatum* Bitter, Bot. Jahrb. Syst. 54, Beibl. 119: 13. 1916. Type: Peru. Huánuco: Chaglla, 3100-3200 m, c. 9°46'S, 1909-1914, *A. Weberbauer 6700* (holotype: B, destroyed; lectotype, designated by Knapp 1989: 90: F [F-628476, F neg. 69667]; isolectotypes: GH [GH00077582], MOL, US [US-1444961]).
Solanum aureum Dunal var. *latelanceolatum* Bitter, Bot. Jahrb. Syst. 54, Beibl. 119: 13. 1916. Type: Peru. Huánuco: Chaglla, 3100-3200 m, c. 9°46'S, 1909-1914, *A. Weberbauer 6700* (holotype: B, destroyed; lectotype, designated here: US [US-1444961]; isolectotypes: F, MOL, US).

##### Type.

Peru. Cajamarca: Cutervo, *C. von Jelski 30* (holotype: W [W 1891-0004325, F neg. 33065]).

##### Description.

Shrubs to small trees, 1–7 m tall. Stems and leaves densely covered with loosely branching golden tree-like trichomes; leaf scars somewhat raised, the stem not winged; new growth densely pubescent with golden tree-like trichomes above and below. Bark of older stems dark reddish-brown, sparsely pubescent with the tree-like trichomes of the young stems. Sympodial units plurifoliate. Leaves simple, 6.5–13 cm long, 1.7–5 cm wide, elliptic or occasionally narrowly elliptic (type), the upper surfaces of the blades drying dark, sparsely pubescent with golden tree-like trichomes, these mostly along the veins, the lower surfaces pubescent with golden trichomes like those of the upper surfaces, the pubescence denser than that above; primary veins 8–12 pairs, sparsely pubescent; base acute, not winged on to the petiole; margins entire, not markedly revolute; apex acute; petiole 0.5–2 cm long, densely golden pubescent. Inflorescences terminal, later appearing lateral or in the fork of the branches, 4–10 cm long, pyramidal, branching ca. 10 times, with 10–20 flowers densely pubescent with loose golden tree-like trichomes like those of the young stems; peduncle 1–4 cm long; pedicels 0.7–1.3 cm long, ca. 0.5 mm in diameter at the base tapering to an apical diameter of 1 mm, slightly nodding at anthesis, densely pubescent with golden tree-like trichomes, articulated at the base and inserted in a sleeve ca. 0.5 mm long; pedicel scars closely spaced and congested at the inflorescence branch tips. Buds ellipsoid, the corolla strongly exserted from the calyx tube. Flowers all perfect, 5-merous. Calyx tube 1.5–2 mm long, conical, the lobes deltate, 1.5–2 mm long, densely pubescent abaxially with golden tree-like trichomes, densely pubescent adaxially with dendritic and simple trichomes. Corolla 1.5–1.8 cm in diameter, violet or occasionally white, lobed 3/4 of the way to the base, the lobes 7–8 mm long, 4–5 mm wide, planar at anthesis, densely pubescent abaxially with tiny dendritic trichomes, these denser at the tips of the lobes, adaxial surfaces glabrous. Filament tube absent; free portion of the filaments 1–1.5 mm long, occasionally slightly pubescent near the base; anthers 3.4–4 mm long, 1–1.5 mm wide, loosely connivent, poricidal at the tips, the pores becoming slit-like with age. Ovary glabrous or with a few dendritic trichomes at the apex, glabrate in fruit; style 5–7 mm long, sparsely to densely pubescent at the base or along its entire length with golden dendritic trichomes; stigma bilobed, the surface minutely papillose. Fruit a globose, purplish-black berry, with thin pericarp, 1–1.2 cm in diameter; fruiting pedicels 1–1.5 cm long, woody, nodding to more or less erect, ca. 1 mm in diameter at the base. Seeds ca. 8–10 per fruit, 3–4 mm × 2.5–3.5 mm, flattened lenticular, reddish-brown, the surfaces minutely pitted. Chromosome number: not known.

**Figure 31.**
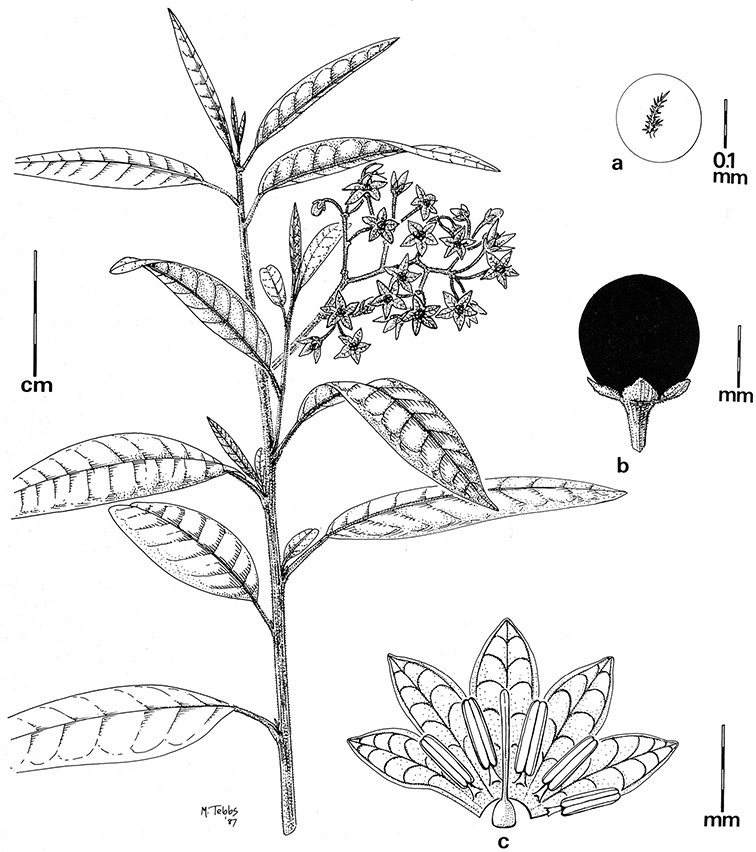
*Solanum cutervanum* Zahlbr. (drawn from *Hutchison & von Bismarck 6571*). Reproduced from Knapp (1989) with permission of the Natural History Museum Botany Library. Illustration by Margaret Tebbs.

##### Distribution

**Figure 32.**
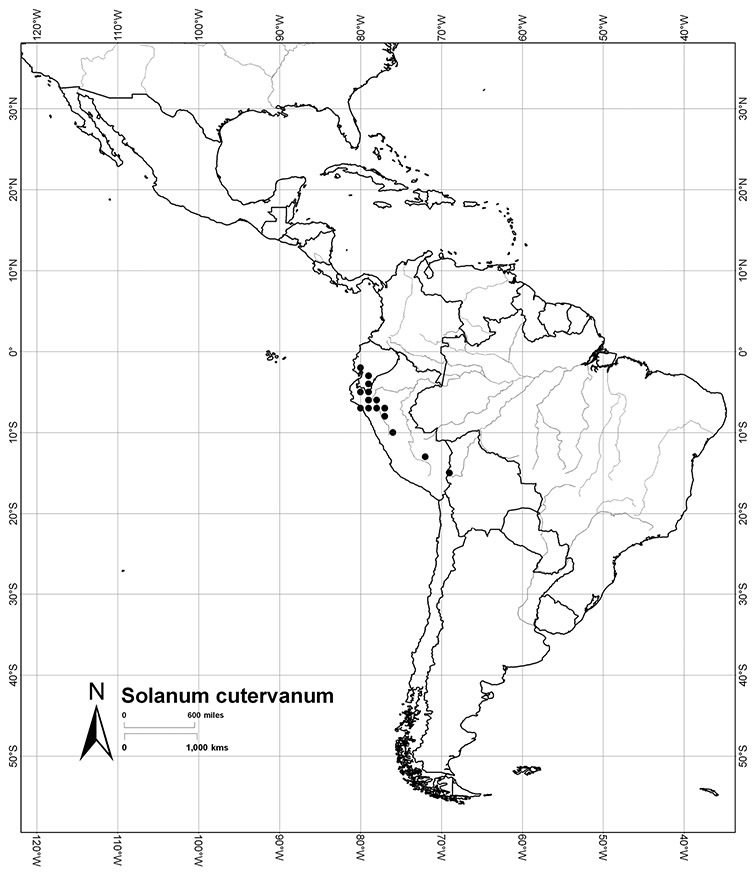
Distribution of *Solanum cutervanum* Zahlbr.

(Figure 32). Andean Peru from Piura to Puno with a single collection known from Bolivia; 2500–3300 m.

##### Ecology.

In rocky uplands, cloud forests and along trails in forest, usually growing in the open.

##### Common names:

Peru: rama de serrano (Knapp 1989).

##### Conservation status.

Least Concern (LC); EOO >100,000 km^2^ (LC) and AOO >10,000 km^2^ (LC). See Moat (2007) for explanation of measurements.

##### Discussion.

*Solanum cutervanum* had long been confused with and placed in the synonymy of S. *nitidum*. Specimens of both these species were previously annotated as *Solanum pulverulentum* Pers., perhaps due to their superficial similarity and sympatric distribution. The two species, however, are very different. *Solanum cutervanum* has golden or brownish tree-like trichomes on stems, leaves and inflorescences and black berries, while *Solanum nitidum* has more delicate, greyish, strictly dendritic trichomes and red ripe berries.

*Solanum cutervanum* is closely related to *Solanum ruizii*, also from central Peru. It differs from that species in its often dichasial branching, rounded leaf bases, smaller flowers and deltate calyx lobes. These sister taxa are sympatric and flower size may contribute to reproductive isolation. Some populations of *Solanum cutervanum* from N Peru have broadly elliptical leaves superficially reminiscent of *Solanum aureum*, a vining species with golden strictly dendritic pubescence.

Unfortunately the species name *Solanum pulverulentum* Pers., so long in use for this species, is a later homonym of *Solanum pulverulentum* L., itself a superfluous name for *Solanum tomentosum* L., an African prickly species (see Vorontsova and Knapp, in review). Linneaus only used the epithet *pulverulentum* in the 1759 edition of his *Systemae Naturae*, and in later publications reverted to the use of his original epithet *tomentosum* for the South African species.

Many monographers in *Solanum* have stated that holotypes or lectotypes for Ruiz and Pavón names were in the Madrid herbarium (MA), but without specifying a particular sheet. This is incorrect when several sheets are present in MA. Knapp (1989) lectotypified *Solanum angustifolium* citing only a sheet in MA; this was rectified in 2008 by citation of the particular sheet (Knapp 2008c).

In describing *Solanum aureum* vars. *angustelanceolatum* and *latelanceolatum* Bitter (1916) used sheets of *Weberbauer 6700* at B for both names; he stated that within a branch leaf morphology was relatively uniform, but that between branches (probably sheets, as he never saw these or any other South American *Solanum* in the field) there was a great deal of variation. He compared *Weberbauer 6700* to a sheet at B collected by Humboldt (an isotype of *Solanum aureum*) and clearly stated (Bitter 1916) that the Humboldt sheet represented the typical variety, and that various sheets of *Weberbauer 6700* were his new taxa. I have selected the F duplicate as the lectotype of var. *angustelanceolatum* (Knapp 1989) and here selected the US sheet of the same number with slightly wider leaves as the lectotype of var. *latelanceolatum* as no duplicate material annotated by Bitter has been found. It is likely he was using material at Berlin (now destroyed) for his description.

##### Specimens examined.

**Bolivia**. **La Paz**: Franz Tamayo, Parque Nacional Madidi, Puina Viejo, ca. 3 km río abajo por camino al E del río, 3345 m, 20 Jun 2005, *Fuentes 8507* (NY).

**Ecuador**. **Azuay**: Cantón Sevilla de Oro, Sevilla de Oro, eastern Cordillera, 4-6 km N of village, 2743 m, 14 Aug 1945, *Camp E-4698* (MO); Cantón Cuenca, Cuenca, Cuenca, Parroquia Cumbe, 2682 m, 3 Jul 1991, *Cerón 15542* (MO); Cantón Sevilla de Oro, Sevilla de Oro, 2950 m, 18 Apr 1968, *Harling et al. 8454* (MO); **Azuay/Morona Santiago**: road Gualaceo-Limón, at the pass point, La Virgen, 3500 m, 26 Feb 1993, *Harling & Ståhl 26713* (MO); **Loja**: Las Chinchas, region central, 2250 m, 12 Apr 1944, *Acosta-Solís 7786* (F); Cordillera de Las Lagunitas. Amaluza-Jimbura-Zumba, Km 36, 3390 m, 22 Nov 1994, *Jørgensen et al. 747* (BM, MO); Parque Nacional Podocarpus, above Cajanuma, trail from ‘Centro de Información’ toward ‘Lagunas de Compadre’, 3100 m, 19 Jan 1989, *Madsen 85561* (BM).

**Peru**. **Amazonas**: Luya, Camporedondo, Tullanya, base Cerro Huicsocunga, 3075 m, 7 Dec 1996, *Díaz & Peña 8853* (MO); Chachapoyas, Cerros de Calla-calla, uppermost slopes and summit, near kms 403-407 of Balsas-Leimebamba road, 3400 m, 18 Aug 1962, *Wurdack 1704* (USM); **Cajamarca**: Jaén, Sallique, 3300 m, 26 Jun 1998, *Campos et al. 5102* (MO); Jaén, Sallique. Quebrada Grande, camino entre La Cocha y Tablón, 2750 m, 2 Jul 1998, *Campos et al. 5183* (BM, MO); Jaén, Lanchal, La Concha, Dist. Sallique, 2960 m, 16 Jun 1998, *Díaz et al. 9597* (MO, USM); Jaén, Sallique, Quebrada grande, ruta entre La Cocha y Tablón, 2770 m, 30 Jul 1998, *Díaz et al. 9779* (BM, MO, MOL); Jaén, Paramillo de Pomahuaca, antes del pajonal, 3200 m, 8 Nov 1999, *Díaz & Campos 10908* (USM); El Pargo, 42 km E of Llama, ca. 14 km SE of Tunas Pampas, 3000 m, 8 Sep 1991, *Gentry et al. 74570* (MO, USM); El Pargo, 16 km E of Tunas Pampa, ca. 42 km E of Llama on road to Huambos, 3000 m, 18 Sep 1991, *Gentry et al. 74897* (MO, USM); Chota, Bosque El Pargo, entre Llama y Huambos, 3010 m, 12 Aug 1994, *Leiva G. et al. 1485* (BM); **Cusco**: Calca, Choquecancha, Azulcocha, Dist. Lares, 3832 m, 18 Feb 2005, *Valenzuela et al. 4978* (NY); **Huánuco**: Huamalíes, Monzón, cerros al sudoeste de Monzón, 3500 m, *Weberbauer 3310* (MOL); **Lambayeque**: Ferrañafe, Cañariaco, Distrito Cañaris, sector Cañariaco, 3083 m, 16 Aug 2008, *Marcelo Peña et al. 3688* (MOL); **Pasco**: Oxapampa, Distrito Huancabamba, Lanturachi, sector Santa Barbara, camino a Milpo, 2824 m, 10 Oct 2003, *Perea et al. 702* (BM); **Piura**: Huancabamba, El Tambo, 3 Jun 1961, *Acleto 294* (USM); Huancabamba, Carmen de la Frontera, alturas de Nueva York, 3280 m, 27 Jul 2006, *Cano et al. 16766* (USM); Huancabamba, carretera entre Canchaque y Huancabamba, 2800 m, 14 Jan 1988, *Díaz et al. 2719* (MO); Huancabamba, Mitopampa (Huancabamba-Cuello del Indio), 2650 m, 22 Jul 1975, *Sagástegui et al. 8251* (MO); **San Martín**: Mariscal Caceres, Parque Nacional Rio Abiseo, cerro al sur de campamento Chochas, 3500 m, 30 Jun 1996, *Cano et al. 7439* (USM); Distrito de Huicongo, valle de Ruibarbos, 3650 m, 12 Jun 2001, *León & Ramírez 5200* (BM); Mariscal Cáceres, Parque Nacional Rio Abiseo, Puerta del Monte, 3200 m, *Young 1573* (K); Mariscal Cáceres, Parque Nacional Rio Abiseo, Chochos, 3400 m, 14 Feb 1986, *Young 2809* (K, MOL, USM); Mariscal Cáceres, Parque Nacional Rio Abiseo, Chochos, 3450 m, 13 Jun 1986, *Young 3763* (K); Mariscal Cáceres, Parque Nacional Rio Abiseo, 3400 m, 3 Jul 1986, *Young 3866* (K, MOL, USM); Mariscal Cáceres, Parque Nacional Rio Abiseo, along trail to El Mirador, Puerta del Monte, NW corner of Park, 3100 m, 11 Jul 1987, *Young & León 4475* (USM).

#### 13. Solanum dichroandrum

Dunal, Prodr. [A.P. de Candolle] 13(1): 86. 1852

http://species-id.net/wiki/Solanum_dichroandrum

Figure 33

Solanum sanctaenevadae Dunal, Prodr. [A.P. de Candolle] 13(1): 678. 1852. Type: Colombia. “Venezuela, Mérida, Sa. Nevada”, 8000, 1847, *N. Funck & L.J. Schlim 1621* (holotype: G-DC [G00144896]; isotypes: BM [BM000849512], G [G00070229, F neg. 6742, IDC microfiche 800-61.2067:III.6], P [P00325803, Morton neg. 8183]).
Solanum dichroandrum Dunal var. *glabrisculum* Dunal, Prodr. [A.P. de Candolle] 13(1): 679. 1852. Type: Colombia. “Venezuela, Prov. de Mérida, Sa. [Sierra] Nevada”, 8000 ft, Feb 1846, *N. Funck & L*.*J. Schlim 1126* (holotype: G-DC [G00144978, IDC microfiche 800-61.2068:I.5]; isotypes: BM [BM000778190], G [G00070156], P [P00325804, Morton neg. 8181], P [P00325805], W).
Solanum endotrichum Bitter, Repert. Spec. Nov. Regni Veg. 12: 161. 1913. Type: Colombia. Cundinamarca: Miquinquirá near Bogotá, Jul 1909, *Bro. Idinaël 57* (holotype: MPU).
Solanum schlimii Bitter, Repert. Spec. Nov. Regni Veg. 16: 85. 1919. Type: Colombia. Magdalena or Guajira: Prov. Río Hacha, Sierra Nevada de Santa Marta, 3100 m, *L. Schlim* [*“Linden”*] *831* (lectotype, designated here: G [G0070139, Morton neg. 8553]; isolectotypes: BM [BM000887368], BR, G [G00070140, F neg. 23165; G00070216], P [P00371281, Morton neg. 8322]).

##### Type.

Venezuela. “Caracas”, 1842, *J. Linden 433* (holotype: G [G00070155, F neg. 8583]; isotypes: BM [BM000778189], G [G00104266], K [K000545358, K000545359], P [P000326802, Morton neg. 8182]).

##### Description.

Woody vine to 8 m long. Stems flexuous and appearing somewhat warty from prominent leaf scars, almost glabrous to densely pubescent with loose dendritic trichomes 1–1.5 mm long, these with few, long branches; new growth sparsely to densely pubescent with loose transparent dendritic trichomes to 1.5 mm long. Bark of older stems pale brown or pale reddish brown, glabrescent. Sympodial units plurifoliate. Leaves simple, 3–7(-11.5) cm long, 1–3.5 cm wide, elliptic to narrowly elliptic, membranous, the upper surfaces glabrous with a few scattered dendritic trichomes along the veins to sparsely pubescent with loose dendritic trichomes to 0.5 mm long, these occasionally mixed with a few simple uniseriate trichomes of the same size, the lower surfaces almost glabrous to densely pubescent with loose dendritic trichomes on the veins and lamina, usually also with sparse glandular papillae on the lamina; primary veins 8–9 pairs, usually reddish brown beneath; base attenuate to acute; margins entire, not revolute; apex acute to acuminate; petioles 0.5–2 cm long, pubescent like the stems, in herbarium specimens usually drying darker and more pubescent adaxially, occasionally twining. Inflorescences terminal, 4–8 cm long, globose to depressed-elliptic in outline, many times branched, with 10–50 flowers, glabrous to pubescent with loose, dendritic trichomes like those of the stems; peduncle 0.3–1.2 cm long, usually branching very near the base; pedicels 1–1.5 cm long, slender, ca. 0.3 mm in diameter at the base, ca. 1 mm in diameter at the apex, glabrous to loosely pubescent with dendritic trichomes, spreading at anthesis, articulated at the base, leaving a swollen area on the axis; pedicel scars irregularly spaced 1–10 mm apart, the inflorescence rachis bent at the articulation points. Buds ellipsoid, the corolla strongly exserted from the calyx tube in early bud. Flowers apparently all perfect (although the type has no long-styled flowers), 5-merous. Calyx tube 1–2.5 mm long, conical, the lobes 1–1.5 mm long, deltate with thickened margins and an elongate apex, sparsely to densely pubescent with loose dendritic trichomes. Corolla (1.2)1.5–2.5 cm in diameter, white or white tinged with lilac, stellate, lobed ca. 3/4 of the way to the base, the lobes 7–10 mm long, 4–6 mm wide, planar at anthesis, densely papillate on the tips and margins, the papillae sometimes extending to the lobes abaxially. Filament tube minute, the free portion of the filaments 1–1.5 mm long, glabrous or minutely pubescent with simple uniseriate trichomes less than 0.2 mm long; anthers ca. 4 mm long, 1 mm wide, glabrous, ellipsoid, loosely connivent, yellow, poricidal at the tips, the pores lengthening to slits with age. Ovary glabrous; style 6–9 mm long, minutely puberulent with tiny simple uniseriate trichomes, these denser in the basal half; stigma capitate, the surface minutely papillose. Fruit a globose berry, ca. 2 cm in diameter, black when ripe, green when immature, dull and matte, glabrous, the pericarp thin; fruiting pedicels 1.5–2 cm long, ca. 1 mm in diameter at the base tapering to an apical diameter of 1.5–2 mm, woody and somewhat deflexed. Seeds 10–12 per berry, ca. 5 mm long, 4.5 mm wide, flattened-reniform, dark brown, the surfaces minutely pitted, the testal cells small and rectangular. Chromosome number: not known.

**Figure 33.**
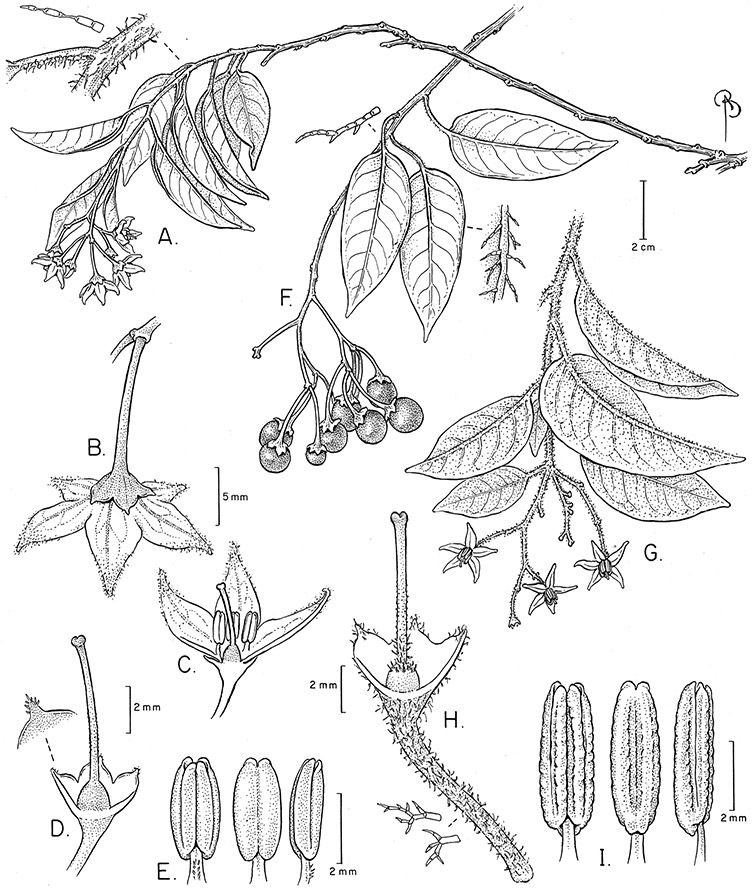
*Solanum dichroandrum* Dunal. (**A–F** drawn from *Riina et al. 784* **G–I** drawn from *Killip & Smith 17916*). Illustration by Bobbi Angell.

##### Distribution

**Figure 34.**
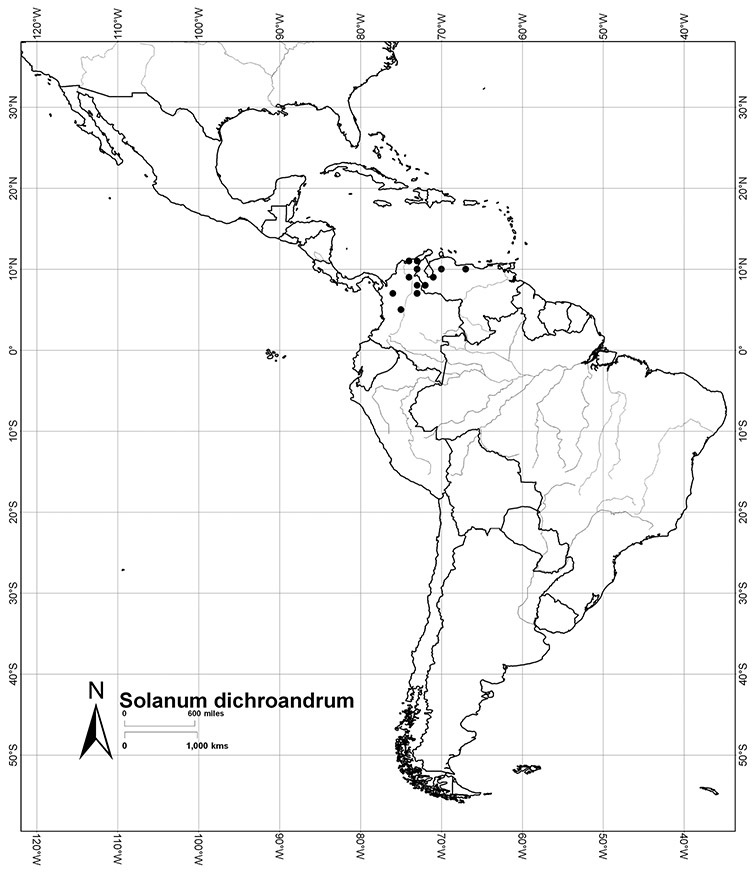
Distribution of Solanum*dichroandrum* Dunal.

(Figure 34). In northern South America in the coastal and western ranges of northern Colombia and Venezuela, extending to the Andes in Venezuela; 2200 to 3300 m.

##### Ecology.

Growing in cloud forests, probably in open areas.

##### Conservation status.

Least Concern (LC); EOO >100,000 km^2^ (LC) and AOO >10,000 km^2^ (LC). See Moat (2007) for explanation of measurements.

##### Discussion.

*Solanum dichroandrum* is very similar morphologically to a number of other Andean members of the Dulcamaroid clade such as *Solanum sanchez-vegae*, *Solanum aureum* and *Solanum luculentum*. It is most similar to *Solanum sanchez-vegae*, sharing with that species large flowers and loose dendritic pubescence. The two taxa differ in their seed number (with *Solanum dichroandrum* having twice as many of seeds), style pubescence (glabrous in *Solanum sanchez-vegae*, minutely puberulent in *Solanum dichroandrum*), and in flower size, with *Solanum dichroandrum* having somewhat smaller flowers. The two taxa are not sympatric, and neither is *Solanum dichroandrum* sympatric with *Solanum aureum*, from which it differs in having larger flowers, looser leaf and stem pubescence and fewer seeded berries. *Solanum dichroandrum* is sympatric with *Solanum luculentum*, from which it differs in leaf and stem pubescence (*Solanum luculentum* is completely glabrous), flower size and sex expression (*Solanum dichroandrum* has perfect flowers, whereas *Solanum luculentum* is almost certainly dioecious).

Specimens collected in the Colombian Department of Norte de Santander around Vetas (*Killip & Smith 17267, 17313, 17388, 17910, 17916*) are very pubescent and have slightly smaller flowers than the type, but otherwise fall within the range of variation seen in *Solanum dichroandrum*.

In describing *Solanum schlimii*, Bitter cited both BR and the herbarium of Barbey-Boissier (now part of the general collections at G) in the protologue; the lectotype selected (G0070139) is the cited sheet from the Barbey-Boissier herbarium and is annotated by Bitter.

##### Specimens examined.

**Colombia**. **Antioquia**: San José de San Andrés, 1 May 1948, *Correa V.& Velásquez V. 35* (US); **Boyacá**: Pauna, carretera a Muxo, Las Curcubitas, km 114-117, 2850 m, 12 Nov 1948, *García-Barriga 13236* (US); **Cesar**: Páramo de Sabana Rubia, 3250 m, 22 Jul 1987, *Cuadros 3726* (MO); **Cundinamarca**: Bogotá, 1919, *Brother Ariste-Joseph A-418* (US); **La Guajira**: Cerro del Espejo, N slopes, Serrania de Perijá, Venezuela border, 2560 m, 28 Apr 1987, *Gentry & Cuadros 57183* (MO); **Magdalena**: Quebrada de Floridablanca, east of Manaure, Sierra de Perijá, 2700 m, 10 Nov 1959, *Cuatrecasas & Romero-Castañeda 25199* (F); Río Garaban, headwaters of Río Aracataca, Sierra Nevada de Santa Marta, 2860 m, 23 Jul 1944, *Kernan 159* (US); Mamancanaca, and vicinity, 3300 m, 27 May 1977, *White & Alverson 611* (MO); **Norte de Santander**: Cerro de Oroque, limites entre los Departmentos Norte de Santander y Cesar, Cordillera Oriental, 3700 m, 22 Jul 1974, *García-Barriga & Jaramillo 20721* (MA, US); **Santander**: Vetas, 3100 m, 16 Jan 1927, *Killip & Smith 17267* (GH, US); Vetas, 3100 m, 16 Jan 1927, *Killip & Smith 17388* (A, GH, US); Vetas, 3100 m, 16 Jan 1927, *Killip & Smith 17916* (A, GH, US).

**Venezuela**. **Lara**: Parque Nacional Dinira, Páramo de Jabón, laderas nororientales, 3000 m, 28 Dec 1999, *Riina et al. 867* (BM); **Mérida**: near Laguna de Coromoto, Sierra Nevada, 3200 m, 7 Aug 1958, *Dennis 2185* (K); Pueblo Hondo, carretera a Mérida km 115-117, 2350 m, 24 Nov 1948, *García-Barriga 13295* (US); Laguna de Coromoto, 3200 m, Jun 1958, *Schwabe* s.n. (B); **Táchira**: La Grita, Páramo el Rosal, 2800 m, 8 Oct 1965, *Bernardi 10898* (K, LE); Zumbador, hacia Queniquea, 2500 m, 31 Jul 1984, *Bono 4066* (MO); Páramo el Pantano, 2500 m, 16 Nov 1976, *Charpin & Jacquemond 13451* (MO); Páramo de la Negra, slopes below páramo, above La Grita, 2430 m, 7 Jul 1944, *Steyermark 57101* (F).

#### 14. Solanum dulcamara

L., Sp. Pl. 185. 1753

http://species-id.net/wiki/Solanum_dulcamara

Figure 35

Solanum scandens Neck., Delic. Gallo-Belg. 1: 119. 1768. Type: Based on *Solanum dulcamara* L.
Dulcamara lignosa Gilib., Fl. Lit. Inch. 1: 37. 1782. Type: Based on *Solanum dulcamara* L.
Lycopersicon dulcamara (L.) Medik., Beobacht. 245. 1783. Type: Based on *Solanum dulcamara* L.
Solanum rupestre F.W.Schmidt, Fl. Boem. 2: 96, tab. 241. 1793. Type: Czech Republic. “Termas Carolinas, Dorotheenau” [Karoly Vary] (no specimens found, plates unpublished; synonymy ex descr.).
Dulcamara flexuosa Moench, Meth. 514. 1794, nom. illeg. superfl. Type: Based on *Solanum dulcamara* L.
Solanum ruderale Salisb., Prodr. Stirp. Chap. Allerton 133. 1796, nom. illeg. superfl. Type: Based on *Solanum dulcamara* L.
Solanum dulcamara L. var. *villosissimum* Desv., Observ. Fl. Angers 111. 1818. Type: France. Anjou: environs de Brissac, *N.A. Desvaux* s.n. (possible type specimen: P [P00582490]).
Solanum dulcamara L. var. *rupestre* (H.W.Schmidt) Roem. & Schult., Syst. Veg., ed. 15 bis [Roemer & Schultes] 4: 581. 1819. Type: Based on *Solanum rupestre* F.W.Schmidt
Solanum persicum Willd., Syst. Veg., ed. 15 bis [Roemer & Schultes] 4: 662. 1819. Type: “In Persia, *Pallas*”(neotype, designated here: BM [BM000942806]).
Solanum littorale Raab, Flora 2: 414. 1819. Type: Switzerland. Vaud: Lac Leman “inter Vidy et Pully; prope Ouchy et Champlande”, *C.W. Raab* s.n. (no specimens found; synonymy ex descr.).
Solanum kieseritzkii C.A.Mey., Verz. Pfl. Cauc. 113. 1831. Type: Azerbaijan. Lenkoran, 23 May 1830, *C.A. Meyer* s.n. (holotype: LE; isotypes: LE (3 sheets), OXF).
Solanum assimile Friv., Flora 19: 439. 1836. Type: Greece. “Turkey in Rumelia”, *I. Frivaldszky* s.n. (holotype: BP [n.v.]; isotype: NY [NY00169744]).
Solanum dulcamara L. var. *tomentosum* W.D.J.Koch, Syn. Fl. Germ. Helv. 2: 508. 1837. Type: Based on *Solanum littorale* Raab
Solanum dulcamara L. var. *album* G.Don, Gen. Hist. 4: 409. 1838. Type: “Dulcis amara flor albo”, Besler, *Hortus Eystettensis*, fol. 46, t. ii. 1613. (lectotype, designated here: Besler, *Hortus Eystettensis*, fol. 46, t. ii. 1613 [illustration on p. 180 in reprints]).
Solanum dulcamara L. var. *carneum* G.Don, Gen. Hist. 4: 409. 1838. Type: Sweden. “Celsius, Ups. 32, Linnaeus, Fl. Suec. p. 66” (no specimens found).
Solanum dulcamara L. var. *hirsutum* G.Don, Gen. Hist. 4: 409. 1838. Type: “Plant hairy, or downy. Flowers violaceous. On the sea-coast.” (no specimens cited).
Solanum dulcamara L. var. *violaceum* G.Don, Gen. Hist. 4: 409. 1838. Type: “Dulcis amara flor coeruleo vulgatica”, Besler, *Hortus Eystettensis*, fol. 46, t. iii. (lectotype, designated here: Besler, *Hortus Eystettensis*, fol. 46, t. iii on folio 46 t. iii. 1613 [illustration on p. 180 in reprints]).
Solanum dulcamara L. var. *cordifolium* Peterm., Fl. Lips. Excurs. 173. 1838. Type: Germany. Sin. loc. (no specimens found).
Solanum dulcamara L. var. *ovatum* Peterm., Fl. Lips. Excurs. 173. 1838. as *ovata*. Type: Germany. Sin. loc. (no specimens found).
Solanum dulcamara L. var. *marinum* Bab., Man. Brit. Bot. 210. 1843. Type: Ireland. County Galway: Connemara, Renvyle [“Renville”] (lectotype, designated here: CGE; isolectotype: BM [BM000941486]).
Dulcamara flexuosa Moench var. *cordata* Opiz, in Bercht. & Opiz, Oekon.-techn. Fl. Böhm. 3: xv. 1843. Type: Based on *Solanum dulcamara* L. var. *cordifolium* Peterm.
Dulcamara flexuosa Moench var. *ovata* Opiz, in Bercht. & Opiz, Oekon.-techn. Fl. Böhm. 3: xv. 1843. Type: Based on *Solanum dulcamara* L. var. *ovatum* Peterm.
Dulcamara flexuosa Moench var. *hastiifolia* Opiz, in Bercht. & Opiz, Oekon.-techn. Fl. Böhm. 3: xvi. 1843. as *hastaefolia*. Type: Czech Republic [?]. sin. loc., *P.M. Opiz* s.n. (holotype: PR?).
Solanum lignosum Sloboda, Rostlinnictví 358. 1852. Type: “Dulcamara Marina, Solanum lignosum Rayi” (no specimens found).
Solanum dulcamara L. subsp. *cordatum* (Opiz) Dunal, Prodr. [A.P. de Candolle] 13(1): 78. 1852. Type: Based on *Solanum dulcamara* L. var. *cordifolium* Peterm.
Solanum dulcamara subsp. *ovatum* (Peterm.) Dunal, Prodr. [A.P. de Candolle] 13(1): 78. 1852. Type: Based on *Solanum dulcamara* L. var. *ovatum* Peterm.
Solanum dulcamara L. var. *rupestre* (F.W.Schmidt) Dunal, Prodr. [A.P. de Candolle] 13(1): 79. 1852. Type: Based on *Solanum rupestre* F.W.Schmidt
Solanum dulcamara L. var. *palustre* Dunal, Prodr. [A.P. de Candolle] 13(1): 79. 1852. Type: Greece. “Lernacicis”, *J. Sartori* s.n. (no specimens found, see discussion).
Solanum dulcamara L. var. *hirsutum* Dunal, Prodr. [A.P. de Candolle] 13(1): 79. 1852. Type: Greece. “Lernacicis”, *J. Sartori 84* (lectotype, designated here: G-DC [G00144899]).
Solanum dulcamara L. var. *laciniatum* Dunal, Prodr. [A.P. de Candolle] 13(1): 78. 1852. Type: United States of America.Massachusetts: Boston, 1827, *Mrs. Dutton* s.n. (lectotype, designated here: G-DC [G00144856]).
Solanum dulcamara L. subsp. *hastiifolia* (Bercht. & Opiz) Dunal, Prodr. [A.P. de Candolle] 13(1): 78. 1852. Type: Based on *Dulcamara flexuosa* Moench var. *hastiifolia* Opiz in Bercht. & Opiz
Solanum dulcamara L. var. *alpinum* Schur, Enum. Pl. Transsilv. 478. 1866. Type: Romania. (Transylvania) Siebenbürgen [Sibiu], ”Arpaser-Kerzesor Alpen” [Arpas], Jul-Aug,* J.F. Schur* s.n. (holotype: GOET?, not found).
Solanum dulcamara L. var. *macrocarpum* Maxim., Index Seminum [St. Petersburg] suppl. 1869: 26. 1870. Type: japan. Jezo Island: Hakodate, 1861, *C. Maximowicz* s.n. (lectotype, designated here: LE; isolectotypes: G [G00301664], LE).
Solanum dulcamara L. var. *integrifolia* Willk. in Willk. & Lange, Prodr. Fl. Hispan. 2: 526. 1870. Type: Spain. Aragón: Jaca, Jun 1850, *H.M. Willkomm 313* (lectotype, designated here: BM [BM000847923]; isolectotype: G [G00357859]).
Solanum dulcamara L. var. *indivisum* Boiss., Fl. Orient. 4: 285. 1879. Type: Based on *Solanum persicum* Willd.
Solanum dulcamarum St.-Lag., Ann. Soc. Bot. Lyon, 7: 135. 1880, nom. illeg. superfl. Type: Based on *Solanum dulcamara* L.
Solanum dulcamara L. forma *subglabrum* Kuntze, Trudy Imp. S.-Peterburgsk. Bot. Sada 10: 222. 1887. Type: Turkmenistan. Asgabat [As’chabad], May 1886, *O. Kuntze [Gen. Komarov]* s.n. (holotype: B, destroyed; lectotype, designated here: NY [NY00172263]).
Solanum rupestre Waisb., Oesterr. Bot. Z. 45: 143. 1895, non *Solanum rupestre* F.W.Schmidt, 1793. Type: Austria. “ad pagum Rödlschlag solo serpentino”, 700-750m, Jun-Jul 1894, *A. Waisbecker* s.n. (no specimens found, holotype: BP?).
Solanum dulcamara subvar. *villosissimum* (Desv.) Bornm., Beih. Bot. Centralbl. 33: 305. 1915. Type: Based on *Solanum dulcamara* L. var. *villosissimum* Desv.
Solanum macrocarpum (Maxim.) Kudô, in Kudô & Susaki, Hokkaido-Yakuyo-Shokub t. 84. 1922. Type: Based on *Solanum dulcamara* L. var. *macrocarpum* Maxim.
Solanum dulcamara L. var. *canescens* Farw., Papers Mich. Acad. Sci. 2: 39. 1923. Type: United States of America. Michigan: Rochester, 15 Aug 1909, *O.A. Farwell 2105* (holotype: BLH [n.v.]).
Solanum dulcamara L. forma *albiflora* Farw., Papers Mich. Acad. Sci. 2: 39. 1923. Type: United States of America. Michigan: Trenton, 26 Jul 1921, *O.A. Farwell 5944* (holotype: BLH [n.v.]).
Solanum dulcamara L. forma *albiflorum* House, Bull. New York State Mus. 254: 613. 1924. Type: United States of America. New York: Livingston County, Caledonia, *F. Beckwith* s.n. (holotype: NYS [n.v.]).
Solanum maximowiczii Koidz., in Mayebara, Fl. Austrohigo 50, 85. 1931. Type: Based on *Solanum dulcamara* L. var. *ovatum* Peterm.
Solanum macrocarpum (Maxim.) Koidz., Acta Phytotax. Geobot. 1: 23. 1932, nom. illeg., non *Solanum macrocarpum* (Maxim.) Kudô Type: Based on *Solanum dulcamara* L. var. *macrocarpum* Maxim.
Solanum megacarpum Koidz., Acta Phytotax. Geobot. 4: 159. 1935. Type: Based on *Solanum dulcamara* L. var. *macrocarpum* Maxim.
Solanum depilatum Kitag., Lin. Fl. Manshur. 390. 1939. Type: China. [Heilongjiang]: “Chéjché, Manchur. bor.”, *M. Kitagawa* s.n. (type not located).
Solanum asiaemediae Pojark., Not. Syst. Herb. Inst. Bot. Acad. Sci. URSS 17: 330. 1955. Type: Uzbekistan. Kokaidskii region, Tamariskovii Krai, [lower Najman-Saj River], 21 Jul 1934, *A.V. Prozorovskii 187* (holotype: LE).
Solanum pseudopersicum Pojark., Not. Syst. Herb. Inst. Bot. Acad. Sci. URSS 17: 328. 1955. Type: Russian Federation. Starvropolskii Krai: Pyatigorsk, Mashukh [protologue Ciscaucasia], 3 Jul 1896, *D. Litvinov* s.n. (holotype: LE).
Solanum marinum (Bab.) Pojark., in Komarov, Fl. URSS, 22: 16. 1955. Type: Based on *Solanum dulcamara* L. var. *marinum* Bab.
Solanum persicum Roem. & Schult. subsp. *pseudopersicum* (Pojark.) Schönb.-Tem., Fl. Iranica 100: 14: 1972. Type: Based on *Solanum pseudopersicum* Pojark.
Solanum kitagawae Schönb.-Tem., Fl. Iranica 100: 15. 1972. Type: Based on *Solanum depilatum* Kitag.
Solanum dulcamara L. luc. *atroviolaceum* Máthé, Bot. Közlem. 59(2): 129. 1972. Type: Hungary. Nógród: “ad pagum Nótines”, *I. Máthé* s.n. (holotype: VBI [n.v.]).
Solanum dulcamara L. forma *lucidum* Máthé, Bot. Közlem. 59(2): 129. 1972. Type: Hungary. Pest: “ad viam pagi Vácrátot”, *I. Máthé* s.n. (holotype: VBI [n.v.]).
Solanum dulcamara L. var. *pusztarum* Soó, Acta Bot. Acad. Sci. Hung. 18(1-2): 172. 1973. Type: Hungary. Mittelungarn, Kom. Bács-Kiskun, Bugacpuszta, *R. Sóo* s.n. (holotype: BP [n.v.]).
Solanum dulcamara subsp. *pusztarum* (Soó) Soó, Acta Bot. Acad. Sci. Hung. 23(3-4) : 382. 1978 [“1977”]. Type: Based on *Solanum dulcamara* L. var. *pusztarum* Soó
Solanum borealisinense C.Y.Wu & S.C.Huang, Fl. Reipubl. Popul. Sin. 67(1): 84, tab. 21, Figure 1–7. 1978. Type: Based on *Solanum depilatum* Kitag.

##### Type.

“Europa”, *Anon*. (lectotype, designated by Knapp and Jarvis 1990, pg. 335: LINN 248.7).

##### Description.

Herbaceous or woody vine, above ground stems trailing to 8–10 m long and with spreading or creeping underground stems. Stems very rarely glabrous, more often pubescent with simple uniseriate or dendritic trichomes with short branches, or a mixture of the two types, these often tangled, 4–8-celled, to 1.5 mm long, usually white, pubescence density extremely variable; new growth usually white pubescent. Bark of older stems grey to yellowish grey. Sympodial units plurifoliate. Leaves simple to ternately pinnatifid, extremely variable in shape and size, even along a single stem, 2.5–7 cm long, 1.2–6 cm wide, elliptic or ovate to cordate in outline, membraneous, the upper surfaces glabrous to moderately pubescent with simple uniseriate or dendritic trichomes to 1.5 mm long on the veins and lamina, the lower surfaces sparsely to densely pubescent with trichomes like those of the upper surfaces, but usually denser; primary veins 6–9 pairs, usually pubescent; base truncate or cordate; margins entire or the leaves lobed, the lobes most commonly 2, rarely more, basal, narrowing near the sinuses; apex acute to acuminate; petiole 0.5–2 (+) cm long, pubescent like the stems. Inflorescences terminal or lateral, not leaf-opposed, (1-)4–15 cm long, many times branched, with up to 40 flowers, only a few open at a time, glabrous to moderately pubescent, the rachis often purplish in hue; peduncle (0.5-)1–7 cm long; pedicels 6–12 mm long at anthesis, ca. 1 mm in diameter, slender, spreading, often purplish green, glabrous to sparsely pubescent with simple unieriate or more rarely dendritic trichomes to 0.5 mm long, articulate at the base in a small sleeve leaving a prominent swollen peg on the axis; pedicel scars irregularly spaced 1–5 (-10) mm apart, the axis zig-zag. Buds turbinate, the corolla long-exserted from the calyx tube before anthesis. Flowers all perfect, 5-merous. Calyx tube 1–1.5 mm long, broadly conical, the lobes < 0.5 mm long, broadly triangular, glabrous or pubescent with uniseriate white trichomes, the apex pointed, the margins papillate. Corolla 1.5–2 cm in diameter, purple, violet or white, with green and white shiny spots at each lobe base, deeply stellate, lobed 3/4 of the way to the base, the lobes 6–8 mm long, 2.5–3 mm wide, strongly reflexed at anthesis, glabrous or minutely papillate on tips and margins, occasionally densely pubescent with simple uniseriate trichomes abaxially, glabrous adaxially. Filament tube minute, the free portion of the filaments to 0.5 mm long, glabrous; anthers 4.5–6 mm long, ca. 1 mm wide, fused into a single column and tightly connivent, poricidal at the tips, the pores not lengthening to slits with age. Ovary glabrous; style 5–9 mm long, glabrous; stigma minutely capitate, the surface papillose. Fruit a globose to ellipsoid berry, 0.6–1.1 cm long, 0.6–1.5 cm wide, bright red when ripe, the pericarp thin and shiny; fruiting pedicels to 1.3 cm long, 1–1.5 mm in diameter, not markedly woody, spreading. Seeds >30 per berry, ca. 3 mm long, ca. 2 mm wide, flattened reniform, pale yellow or tan, the surfaces minutely pitted, the cells of the testa pentagonal. Chromosome number: n=12 (many counts available at ICPN, http://mobot.mobot.org/W3T/Search/ipcn.html)

**Figure 35.**
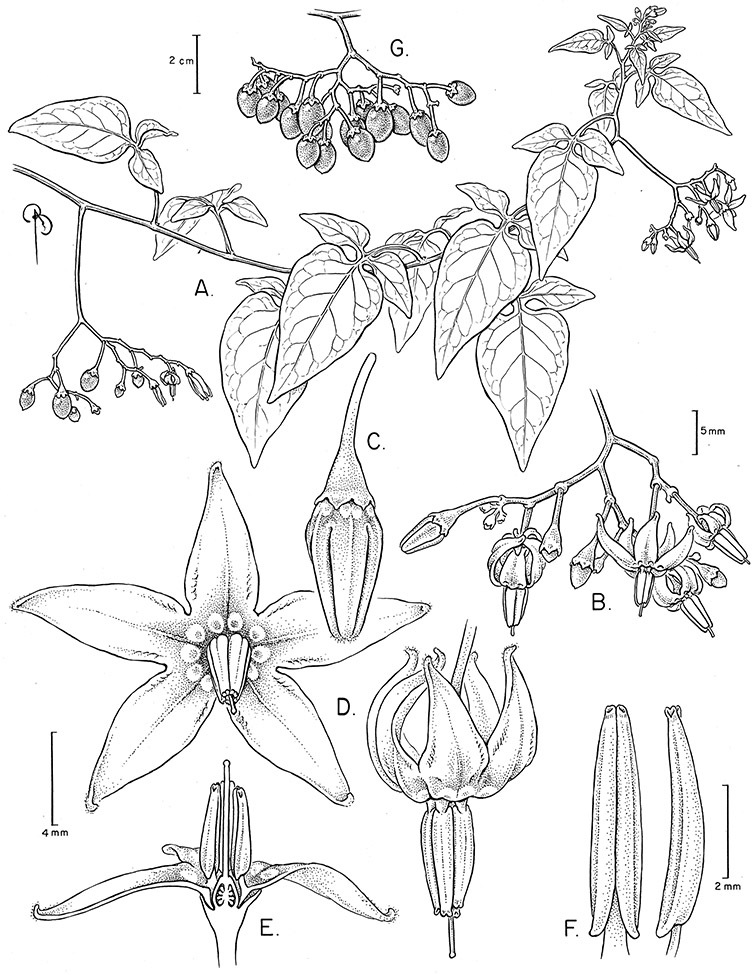
*Solanum dulcamara* L. (All drawn from live plants in Battleboro, Vermont, USA). Illustration by Bobbi Angell.

##### Distribution

**Figure 36.**
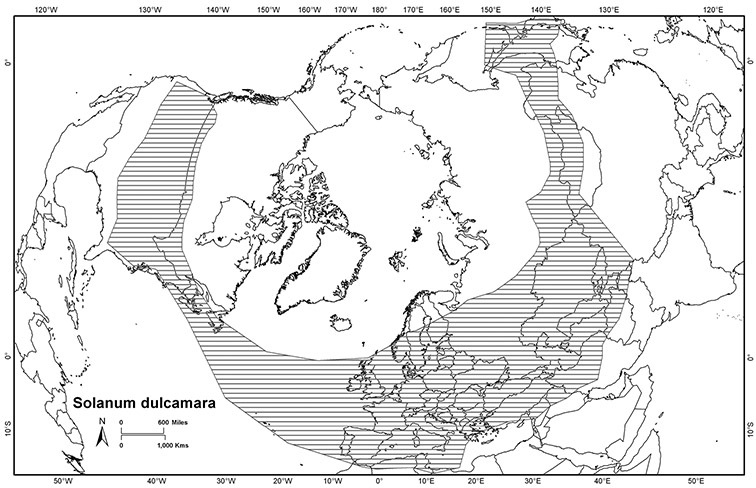
Distribution of *Solanum dulcamara* L.

(Figure 36). *Solanum dulcamara* is widely distributed across Eurasia and northern North America, where it is also common; sea level to ca. 2000 m. The North American populations are thought to be introductions, but it is possible that the species has a truly circumboreal distribution.

##### Ecology.

*Solanum dulcamara* is a weedy species and grows in a wide variety of temperate habitats, often associated with water and open places with abundant light. Although somewhat woody, it rarely reaches into the canopy but is more often found in thickets and sprawling in other low vegetation.

##### Common names.

Douce-amére (French); woody nightshade, bittersweet (English speaking Europe)

##### Conservation status.

Least Concern (LC); EOO >100,000 km^2^ (LC) and AOO >10,000 km^2^ (LC). See Moat (2007) for explanation of measurements. *Solanum dulcamara* has a circumboreal distribution and is very common so it is not of conservation concern. Peripheral populations such as those in eastern Russia, however, may harbour interesting genetic variation (see Knapp 2011).

##### Discussion.

*Solanum dulcamara* is one of the most variable species in the Dulcamaroid group, all of which show extremes of phenotypic plasticity. It is also the most widespread, and is found throughout the northern hemisphere, in Europe and Asia from Spain to Siberia and into extreme northern Japan, and across the northern United States (reported from Florida on the PLANTS database [http://plants.usda.gov/java/profile?symbol=SODU], from where I have seen no specimens), where it is presumed to have been introduced from Europe. I can find no documentary evidence for the introduction of *Solanum dulcamara* into the New World, but early collections from North America held in BM (e.g., Bartram, Clayton) do not contain specimens of this species. The earliest collections I have seen are from the Boston area in the early 19^th^ century (e.g., *Dutton* s.n.); it is possible that *Solanum dulcamara* was introduced to North America for its medicinal properties.

Some of the variation in *Solanum dulcamara* appears to be related to habitat (e.g., coastal forms with succulent leaves that have been recognised as var. *marinum* and *Solanum littorale*) but much of the variation is in leaf size and division, pubescence and flower color; I have been able to find no pattern related to either geography or habitat in this variation. Turesson (1922) suggested that differences in morphology related to habitat were genetic, despite his finding that in non-coastal environments thick-leaved coastal forms developed thinner, less pubescent leaves. Sun and shade leaves of *Solanum dulcamara* have been studied for physiological and some morphological properties (Clough et al. 1979a, b). Differences in morphology can occur along a single stem depending upon the shade environment. Unlike some other species in the Dulcmaroid clade (e.g., *Solanum umbelliferum*) variation does easily sort into distinguishable units, but the sheer range and complexity of the variation has led to the description of many taxa within what I recognise here as a single highly polymorphic species, especially in floristic treatments from Central Asia (Pojarkova 1955; Schönbeck-Temesy 1972), where much of the variation exists.

Chemical variation in *Solanum dulcamara* has also been intensively studied in eastern Europe (Máthé and Máthé 1972, 1979); populations with pubescent individuals from the northen part of the eastern European range tended to have higher percentages of the alkaloid aglycone tomatidinol while more glabrous populations had higher concentrations of soladulcinine. The concentrations of various alkaloids were affected by the environmental conditions under which plants were grown (Máthé et al. 1975) as well as by what were presumed to be genetic (ecotypic) factors (Bernáth and Tétényi 1977). Clough et al. (1979a) and Pegtel (1985) both suggested that ecotypic differentiation did not explain the variation observed, but instead that the variability was the result of short-term phenotypic acclimatization to differing environments. A study using AFLP (Associated Fragment Length Polyomorphisms) data from Europe-wide accessions of *Solanum dulcamara* found almost no genetic variation or population structuring either geographically or in association with habitat (Golas et al. 2010).

A few of the more extreme forms have recognised at the species level, but the variation in the diagnostic features of these forms is continuous across the range of the species. Identifications of individual specimens by botanists describing these variants were occasionally inconsistent from duplicate to duplicate.

Plants occurring in the swampy areas around the hot springs at Lenkoran in what is now Azerbaijan were described as *Solanum kieseritzkii*; all of the collections from this locality are composed of small erect shoots connected with creeping stems and have very small inflorescences of only a few flowers.

Glabrous plants with simple leaves have been recognised as *Solanum pseudopersicum*; glabrous plants with divided leaves as S. *kitagawae*.

Pubescent plants with simple leaves were described as *Solanum persicum*; these tended to be from the southern part of the species range in Eurasia, but occur throughout the species’ range.

Large-fruited plants from the eastern margins of the Asian range have been called var. *macrocarpum*; these fruits also tend to be more ellipsoid than globose, but as with other chracters, this occurs sporadically throughout the range of the species.

White-flowered plants have been given varietal or subspecific status both in Europe and the United States; polymorphism in flower color is common in *Solanum* in general.

*Solanum dulcamara* has a long history of use in medicine in Europe and in the United States. Gerard (1597:350) says “The leaves and fruit of the Bitter-sweet are in temperature hot and dry, clensing and wasting away” and cites as its “Vertues” effects on liver and spleen, against jaundice and bruising, and of its use to help breathing difficulties and as a restorative after childbirth. The common name bittersweet (or douce-amère) comes from the Latin ‘Amaradulcis’, and refers to the fact that when chewed, the twigs taste at first bitter, then sweet (Grieve 1931). Bittersweet had many uses ranging from inflammatory diseases of all kinds (such as asthma and rheumatism) to nymphomania and syphilis (Dunal 1813; Felter and Lloyd 1898); Grieve (1931: 590) states “there are few complaints for which it has not at some time been recommended.” As ‘Dulcamara’ or ‘Dulc’ (van Zandvoort 1996), the stems are used still in homeopathic medicine (Bharatan et al. 2002). It is today listed as a poisonous plant in many countries and US states (see for example the FDA at http://www.accessdata.fda.gov ). Michel-Félix Dunal treated the uses and superstitions associated with this species (Dunal 1813) and concluded that the many properties attributed to bittersweet were mostly not substantiated in fact. The steroidal alkaloids responsible for the active properties of *Solanum dulcamara* were not discovered until the late 19^th^ century, they are principally dulcamarine and solanine, the first of which is responsible for the bittersweet taste and the second for the narcotic effects (Grieve 1931).

*Solanum dulcamara* has been thought to be an intermediate host and possible source of primary infections for several agronomically important potato diseases such as late blight (caused by the oomycete *Phytophthora infestans* (Mont.) de Bary) and brown rot/bacterial wilt (caused by the bacterium *Ralstonia solanacearum* (Smith) Smith). Eradication of *Solanum dulcamara* has been attempted as a control of brown rot (Persson 1998), and use of water from ditches in which *Solanum dulcamara* grows is a known cause of brown rot spread in irrigated potato fields. The role of *Solanum dulcamara* in the spread of late blight, however, has been shown to be minimal (Golas et al. 2010b), as most plants are resistant, and those that are susceptible to the disease do not harbour it over winter. Even in very favourable conditions only sporadic infections occur (Cooke et al. 2002; Flier et al. 2003; Dandurand et al. 2006). *Solanum dulcamara* has novel genes for resistance to late blight, and there is scope for its use in new potato breeding programs in Europe (Golas et al. 2010b). Little work has been done to investigate the mechanisms or genetics of resistance to the major diseases of potato in species outside the Potato clade, but new breeding methods have stimulated the investigation of disease in wild species such as *Solanum dulcamara* (Golas et al. 2010b) and *Solanum nigrum* L., a European hexaploid member of the Morelloid clade (Lebecka 2008, 2009).

Typification of the many synonyms of *Solanum dulcamara* has been very difficult. Many of the early names are found in early 19^th^ century floristic accounts, and while validly and effectively published, usually do not cite specimens. I suspect many of these were not necessarily based on specimens, but rather on field observations. I have not neotypified any of the synonyms for which I could not find specimens directly linked to the original description. In addition, many of the European floras coined complex series of replacement names in the early parts of the 19^th^ century, before the codes of nomenclature became firmly established. Where I have been able to trace the identity and derivation of epithets at varying ranks through citation of specimens I recognised these names as homotypic, but for many of the early names (e.g., those coined by George Don in 1838) the links are very tenuous and I have thus recognised them as heterotypic (and often have not designated types).

The type of *Solanum persicum* was a Pallas specimen held in Berlin, now destroyed. A Pallas specimen in BM that matches the description perfectly has been selected as a neotype, although it may not be a duplicate of the original.

**Figure 37.**
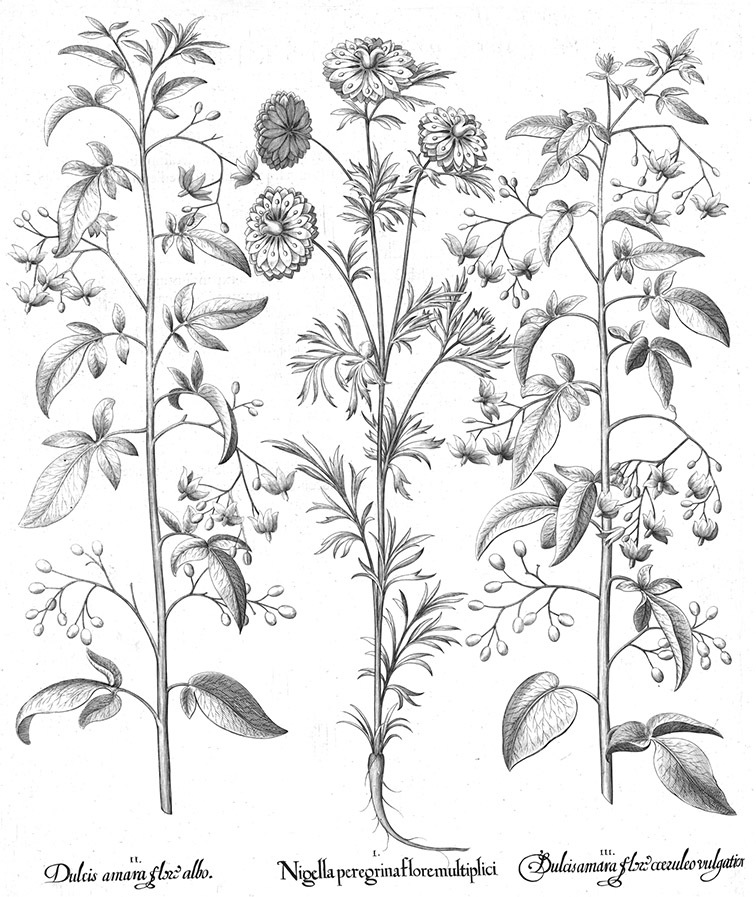
“Dulcis amara” from *Hortus Eystettensis* (Besler 1613). The centre plant is clearly Nigella (Ranunculaceae), but was described as a variety of *Solanum dulcamara* by David Don. Reproduced with permission of the Natural History Museum Botany Library.

The many varietal names coined by George Don (1838) were based not on specimens, but on literature references (e.g., var. *album*) or personal observations (var. *hirsutum*). His var. *hirsutum* referred to plants that were “Hairy or downy. .. On the sea coast”, with no specimens or plates cited. I have not neotypified this name. Varieties *album*, *violaceum* and *plenum* were based on one plate (plate 46) in *Hortus Eystettensis* (Besler 1613) which is composed of three plants (see Figure 37): one white-flowered *Solanum dulcamara* that I have designated as the lectotype of var. *album*, one violet flowered *Solanum dulcamara* that I have designated the lectotype of var. *violaceum*, and a central plant, used by Don as the basis for var. *plenum*, that is a drawing of *Nigella sativa* L. (Ranunculaceae) (see Figure 37). He cannot have been looking very carefully!

Petermann (1838) coined several new infraspecific taxa in his local flora of the Leipzig region; I have found no material related to these names. *Solanum maximowiczii* was based on “*Solanum dulcamara* var. *ovatum* Maxim.” and was coined at the specific level to represent that variety. In his discussion of *Solanum dulcamara*, however, Maximowicz (Regel and Maximowicz 1870) refered explicity to Dunal’s variety *ovatum* and did not coin a new name. Thus, this specific epithet is homotypic with the chain of epithets linked to the variety *ovatum* treated by Maximowicz, ultimately traceable to the (presumably) first instance of this varietal name being used in Petermann’s floristic treatment (Petermann 1838). Dunal (1852), cited by Maximowicz, cites Opiz (Berchtold and Opiz 1843) and Opiz cites Petermann as the origin of the varietal name. I have found no specimens associated with these names.

Dunal’s (1852) treatment of *Solanum dulcamara* for the *Prodromus* has a broad range of variation recognised as a single taxon; he recognised 14 infraspecific entities at two levels, mostly based on literature citations, his final words in the description - “Multum variat.” - couldn’t be more true. He recognised as A, B and C forms previously recognised by Opiz (Berchtold and Opiz 1843) and Petermann (1838) – *cordatum*, *ovatum* and *hastiifolia* (as *hastaefolia*). Below them on the page he recognised a series of 11 taxa (“Hae tres formae vicissum variant”) that he listed with Greek letters (α to ξ, leaving out ι, κ and λ), some of which had binomial and others polynomial epithets. Infraspecific taxa denominated with Greek letters in Dunal (1852) have always been recognised at the varietal rank, and no where else in the treatment of *Solanum* in the *Prodromus* does Dunal use an apparently nested set of ranks as he does with *Solanum dulcamara*. I am therefore assuming that he intended his A, B and C ranks to be hierarchically above the varietal epithets and have treated them as subspecies in the synonymy. It is possible that var. *hastiifolia* of Opiz is in fact an illegitimate name for var. *flexuosa*, as it seems from the text that Opiz (Berchtold and Opiz 1843) treated this variety as the typical one, but I cannot be certain and so have recognised it as legitimate. The polynomial varietal epithets (e.g., “*corollis violaceis*”, “*corollis albis*”, “*corollis carneis*”, “*flore pleno*”, *corollis variegatis*”) are not legitimate names; I have lectotypified the rest based on either herbarium material or literature citations cited by Dunal. For var. *laciniatum*, Dunal cited specimens in both P and G-DC; I have found only the G-DC sheet, so have selected it as the lectotype.

The holotype of *Solanum dulcamara* forma *subglabrum* was destroyed in Berlin and a duplicate of Kuntze’s collection held in NY has been selected as the lectotype.

In describing *Solanum dulcamara* var. *macrocarpum*, Maximowicz (Regel and Maximowicz 1870) cites as a locality “Circa Hakodate insulae Jezo pluribus locis”. Several collections of Maximowicz’s from Hakodate dated 1861 are held at LE; others are in BM, GH and NY. It is very unclear whether any of these are true duplicates, so I have selected one of the flowering and fruiting sheets at LE as the lectotype and another at LE that is almost identical in morphology as a possible isolectotype.

Wasibecker’s (1895) *Solanum rupestre* is a later homonym of *Solanum rupestre* H.W.Schmidt. It is possible that the name *Solanum serpentini* Borbas & Waisb., apparently coined in 1897 (Soó 1968: 153) is a replacement name for the homonym, as the epithet indicates an origin on serpentine soils (like the type of *Solanum rupestre* Waisb.). I have been unable to locate the place of publication of *Solanum serpentini*, and so treat *Solanum rupestre* Waisb. as a synonym of *Solanum dulcamara* and *Solanum serpentini* as a doubtful name (see Doubtful and excluded names and Names not validly published).

Máthé and Máthé (1972) undertook an exhaustive study of the variation of *Solanum dulcamara* in Hungary and described two new variants, one at the form level (forma *lucidum*) and the other (luc. *atroviolaceum*) at the rank ‘lucus’ that was used in by Hungarian botanists between variety and forma. He followed Soó (1968) who transfered many earlier epithets to the rank of lucus, however, none of these are validly published, as no explicit reference to the basionym’s place of publication was made as is required by the *Code*.

I here cite specimens I have examined; these do not cover all European countries, although *Solanum dulcamara* is recorded from throughout Europe. I have selectively cited material from the range edges, as that is where I anticipated taxonomic problems might lie. *Solanum dulcamara* is very commonly collected and the specimens cited here do not completely represent the range.

##### Specimens examined.

**Afghanistan**. Kurrum valley, at Zabardastkalla and Alikhél, Dec 1879, *Aitchison 861* (BM, GH, K, LE, S); Sere Tange, south of Marshad valley, Keshan, 1250 m, 30 Jun 1969, *Carter 375* (K); Parwan, 33 miles from main Salang road, by side of Gharband River, 1920 m, 13 Jun 1969, *Hewer 1274* (K, LE); Gulbahar, 14 May 1937, *Koelz 11428* (LE, US).

**Albania**. Shkoder, North Albania, environs of the city of Shkoder near the river Umri, 14 Jul 1959, *Kuvaev 95 4* (LE).

**Algeria**. Clemcen, vers les Cascades, 800 m, 24 Aug 1932, *Faure* s.n. (S); Elenicens?, Aug 1849, *Romain* s.n. (BM).

**Andorra**. Santa Julia de Loria, de borda del Sabater a Tolse, 840 m, 27 Aug 2002, *Aedo et al. 8428* (MA).

**Armenia**. Kafanskii Region, near Ang, 24 Aug 1980, *Avetisyan* s.n. (MO); Karassan, 1837, *Koch* s.n. (LE); Arpa, between Iahichevan and Yerevan River Arpa to the right of the bridge, 21 Jun 1974, *Mamakli* s.n. (LE); Kirovakan station, 26 May 1953, *Tamamschian, S*., *s.n.* (LE); Megra, Megrinskii Region, 21 Jul 1954, *Tamamshek & Deiisova* s.n. (LE); Kirovakai, 26 Aug 1953, *Tamaschira* s.n. (LE); Alaverdskii reg.**,** S. Sanain, Uzunar, 25 Jul 1960, *Avegisyan & Gabrielian* s.n. (LE).

**Austria**. **Tyrol**: Tirolia centralis, in valle Gschnitz prope pagum Trins, *Kerner 3290* (BM, LE); **Vienna**: Wien, erster Bezirk, an der Grundmauer des Naturhistorischen Museums auf der Seite der Ballariastrasse, 200 m, 2 Oct 1992, *Wallnöfer 4212* (BM).

**Azerbaijan**. Distr. Schemacha, Fagraüksch, Prope st. Scharodilskaja, ad lacum Fagraüksch, 792 m, 8 Aug 1900, *Alexeenko 11304* (LE); Bakinskaya Oblast’, Kusary, Kubinskii region, 20 Jul 1900, *Grigoreev 20* (LE); Lenkoran, 1834, *Hohenacker* s.n. (G-DC, K, LE, MO); Distr. Kuba, Seput, 24 Jul 1930, *Kasumova* s.n. (LE); Eshakchi, 3 Jul 1909, *Kirichenko* s.n. (LE); Shirvan steppe, inter Kerzi-Kent et Kotoran, 24 Aug 1930, *Kolakovskii* s.n. (LE); Gandzha Oblast’, District Kazakh, near the road to Poyly, 13 Jun 1928, *Kolakovskii* s.n. (LE); Lenkoranskiy Okrug, [near village of Nipolaevna, on river Vilyazh-chai], 30 Jul 1931, *Matveeva 791* (LE); Gandzha Oblast’, Bardy, Agdam District, on the Karabkh steppe, near town, by the River Terter, 17 Aug 1927, *Prilipko* s.n. (LE); distr. Saljany, Saljany, prope st. Dzhafarchan, 6 Jul 1927, *Prilipko* s.n. (LE); distr. Lenkoran, Tangovan, near village Tangovan, 26 Jun 1931, *Shipchinskii 275* (LE); Talysh, Lenkoranskii, 16th km on the road from Lenkoran to Leric, left bank of the river Lenkoran-chai, 100 m, 1 Jul 1952, *Vasiliev & Vasilieva* s.n. (LE); 18 km from Sheka, 26 Jun 1972, *Vlasov* s.n. (LE).

**Belgium**. **Brussels**: garden on Chaussee Pas in Auderghem, near a forest east of Brussels area, Jul 1987, *Geerinck-Coutrez 4343* (BM); **West Flanders**: Knokke, Le Zwin, 21 Jun 1971, *Auquier et al.* s.n. (LE); Knokke, Zrvin, 8 Aug 1955, *Lonvalrée 6781* (BM).

**Bosnia-Herzegovina**. Mostar, 1886, *Bornmüller 1405* (B).

**Bulgaria**. Varna, Aug 1886, *Bornmüller* s.n. (B); 8-9 km W of Velingrad, along the stream Lukovica, 980 m, 8 Jul 1982, *Frost-Olsen 4401* (MA); Vitosa, 10 Jun 1927, *Georgieff* s.n. (LE); Malkija Kozuh, Struma valley by the hill Malkija Kozuh, 9 Jun 1980, *Kuzmanov 801405* (B); Samokor, 30 Aug 1912, *Post 277* (G); Sliven, 16 Jul 1907, *Schneider 710* (BM); Prilap, May 1917, *Stribrny* s.n. (LE).

**Canada**. **British Columbia**: Vancouver, Vancouver 8: Alma. Roadside, 18 Jul 1954, *Bird 43* (BM);, S. Okanagon, White Lake, Oliver road, 480 m, 6 Jul 1961, *Vrugtman & Campbell 610348* (BM); **New Brunswick**: Kent County, Newcastle, rocky shore of Miramichi River, 17 Jul 1945, *Dore & Gorham 45555* (G); St. John County, Fairville, near St. John River, 19 Sep 1926, *Fernald et al. 394* (GH); Bass River, Kent County, *Fowler* s.n. (LE); **Newfoundland**: Salmonier, 1931, *Ayre* s.n. (GH); **Nova Scotia**: Annapolis County, Granville, North Mtn, 18 Jul 1921, *Bartram & Long 24435* (GH, LE); Yarmouth County, Markland, Cape Forchu, 13 Jul 1921, *Fernald et al. 24434* (K, LE); Shelburne County, East Jordan, 4 Aug 1921, *Fernald & Long 24436* (GH); Halifax County, Halifax, 30 Sep 1924, *Jack 3644* (GH); Guysborough County, Guysborough, 14 Aug 1930, *Rousseau 35472* (K); **Ontario**: Plevna, 26 Jul 1902, *Fowler* s.n. (GH); Hastings County, Marmora, Concession 3-4, Twin Sister Lakes, 8.5 miles NNE of Marmora, 14 Aug 1952, *Gillett & Hammond . 6922* (G); Bruce County, Tobermory, 2 Jul 1933, *Krotkov 7758* (GH); Waterloo County, Westmount Woods, 1 mi. W of Kitchener, 16 Jun 1939, *Montgomery 338* (GH); Brittania, 18 Jun 1921, *Rolland 15680* (GH); Halton County, Great Lakes Region, Lake Medad, 25 Jun 1940, *Soper & Burcher 2011* (BM, GH); **Prince Edward Island**: Queens County, Charlottetown, 28 Aug 1912, *Fernald et al. 7789* (GH); **Quebec**: Iberville County, Ile aux Noix, 12 Aug 1928, *Adrien 2098* (K, LE); Missisquoi, Noyan, 23 Jun 1959, *Gervais & Lavigne* s.n. (G); Pontiac County, Beech Grove, 19 Aug 1943, *Lamarre* s.n. (MA).

**Croatia**. Dalmatia, Spalolo, Mossor, 1886, *Bornmüller* s.n. (B); Golubevec coal district, 1924, *Leathes* s.n. (BM); island of Veglia (Crk-Venica), Sep 1924, *Leathes* s.n. (BM).

**Denmark**. Copenhagen, Lyngby Mose, 14 Jul 1941, *Dahl D-45 a* (BM); Odder, Sondrup Plantage, ca. 11km S of Odder, W part of the plantation, 25 Jul 1978, *Frost-Olsen 1748* (LE); Lake Tissø, D42, Jul 1968, *Hjorih-Olsen 10* (BM); Jutland, Silkeborg, Borre Lake, 1 Aug 1968, *Jensen et al. 464* (BM, MA); North Jutland, Stenbjerg, 9 Jul 1964, *Larsen & Laegaard* s.n. (LE); Skagen, 9 Aug 1973, *Larsen et al. S.L. 6847* (BM, MA).

**Egypt**. Sin. loc, *Anon*., *s.n.* (LE).

**Estonia**. At village Oiu, 12 Sep 1967, *Aasamaa 11616* (MO); Dorpet [Dorpat = Tartu], 12 Jul 1860, *Gruner* s.n. (BM);Vijlandi County**,** Tipu, River Halliste, 14 Jul 1994, *Reier* s.n. (B).

**Finland**. Pyhäranta, Rihtniemi point and village, 21 Aug 1968, *Alava et al.* s.n. (LE); Nauvo, Seili, near church, 4 Aug 1976, *Arkkola et al.* s.n. (MA); Åland, Nafsby, Prov. Hammarland, 28 Jul 1963, *Haakana* s.n. (LE); Kupittaa, Turku, Varsinais-Suomi, 14 Aug 1924, *Kari* s.n. (LE); South Häme, Heinola, Tommola, Maitiaislahti, 7 Aug 1980, *Kemppainen* s.n. (MA); Kärkölä, Järvelä, middle part of the NE shore of Lake Hähkäjärvi, just NW of the man-made cape, 97 m, 29 Jun 1992, *Lampinen 14764* (MA); Kuitia, Parainen, Lemlaxö, 7 Aug 1971, *Kukkonen 9106* (LE); Sauvo, Karona, near the church, 13 Jul 1976, *Lempiäinen & Ravanko* s.n. (MA); Broby, Nylandia, par. Pyttis, 28 Jun 1963, *Majander & Nordström* s.n. (LE); Norra byn, par. Sottunga, 11 Jul 1973, *Nordström* s.n. (LE); Uusimaa, Jätkäsaari, Helsinki, harbour, 28 Jul 1968, *Oinonen* s.n. (LE); Versinais-Suomi, Iniö, Norrby, 13 Jul 1929, *Valle* s.n. (MA); Jyddo-ojen, Foglo, Ahvenanmaa (Åland), 29 Aug 1907, *Wahlberg* s.n. (LE).

**France**. **Alsace**: at the edge of the river at Haguenau, 24 Jul 1846, *Billot 160* (BM); **Auvergne**: Auliac, 26 Sep 1835, *Anonymous* s.n. (BM); chemin de Surat à Maringues; chemin de Bussière à St.Clement, Jun 1906, *Caumel 334* (BM); **Brittany**: chemin de Chantenay à Rochemaurice, 12 Aug 1901, *Gadeceau 5106* (BM); **Corsica**: zwischen Pte d’Abatesco und Mignattaja S von Ghisonaccia, 10 Aug 1933, *Aellen 4601* (LE); **Languedoc-Rousillon**: Bouches du Rhone, Camargue, Tour du Valat, SE of Mas, 11 Jun 1968, *Kendrick & Moyes 231* (BM); Bouche du Rhone, Camargue, Etang de la Dame, 2 miles SE of Salin de Giraud, 21 Jun 1968, *Kendrick & Moyes 346* (BM); **Loire-Atlantique**: Ouest de France, environs de Vertou, Jun 1880, *Gadeceau* s.n. (BM); **Nord Pas de Calais**: Beuvrequent, ruisselet de Slack, 24 Jul 1986, *Coutrez 3780* (MA); **Pays-de-la Loire**: Nantes, loin le Fevre, 18 Jul 1847, *Genevier* s.n. (BM); **Picardy**: Aisne, St. Quentin, Jun 1871, *Moignier* s.n. (BM); **Poiteau Charente**: Rochel, Plaisance en Oléron (Charente-Inferieure), 20 Oct 1892, *Reau* s.n. (BM); **Rhône-Alpes**: Bonneville, bords d’Arve, *Depierre* s.n. (BM); Haute Savoie, route d’Etrumbiern a Mornex, 14 Jun 1859, *Déséglise 281* (BM); Hirault, Mont Blanc, Station Crenugua, 7 Jul 1954, *Witte 14547* (MA).

**Georgia**. Dusheti District, south and east of Fudauri, north of Pasanauri, slopes along Georgian M3 Road (Georgian Military Highway), 1784 m, 5 Aug 2005, *Atha et al. 5005* (US); Chevsurya, between Barshak and Reshka, 1 Jul 1903, *Busch* s.n. (LE); Akmolinsk prov., Kokczetav District, 4 May 1913, *Drobov 203* (S); South of the Krestovyy Pereval, Georgian Military Highway, Belaya Aragvi valley, between Ordzhonikidze and Tbilisi, Gruzinskaya S.S.R. along walls in the village of Mleti, 4 Jul 1971, *Jenkins 2946* (BM); Somchetya, Teleti, near Tiblisi, Oct 1923, *Juzepczuk* s.n. (LE); Klukhorskii, between villages of [illegible] and Madniekheva, 13 Aug 1946, *Kemularia-Nathadze & Chinthibidze* s.n. (LE); Kutaisi, Ozur, 15 Jun 1914, *Kikodse* s.n. (LE); Abkhasia, Teberdinskii, Protected Area [Terbeda], 1938, *Komarov & Komarova* s.n. (LE); Kazbek, Dushetskii region, near the 31st field, Tifliskaya Province, 1916, *Krylov & Steinberg* s.n. (LE); Megrelia, near village Inghuri towards city Tobashi, 14 Aug 1952, *Madenova & Kuthatheladze* s.n. (LE); along the Georgian Military road in the area of Krestovi Pass N of Tibilisi, 31 Jul 1984, *McNeal et al. 332* (MO); Atskhur, Akhaltsikhskii region, vicinity of village Atskhur, 16 Jul 1926, *Meffert 314* (LE); Kazbegi, Aug 1971, *Menitskii* s.n. (LE); Abkhasia, Sukhumsk, District of Tsebeld, in the Peukirskoe gorge on open slopes of the mountain Agysh near the road, 23 Aug 1937, *Pobedimova 383* (LE); Abkhasia, Gagra, on the lower road towards Zhoevarskii waterfall, 4 Nov 1915, *Sakharov* s.n. (LE); Mtiuleti, Kasbegi Region, Dariali Gorge, Greater Caucasus Mountain Range; ca. 12 km N of Kasbegi village, on footpath to Gveleti village to local waterfalls, 1600 m, 24 Aug 1998, *Schmidt et al. 2891* (G); Tiblisi, by the River Vera, 26 May 1917, *Shishkin* s.n. (LE); Adzharskaya, Nizovaya Natanebi, 4 Aug 1948, *Sochava & Semenova* s.n. (LE); Adzharskaya, gorge of the river Bakhvis-tskali, 9 May 1941, *Tatishvili* s.n. (LE); valley of the River Terek to the south of the Station Lars, 25 Aug 1949, *Vasilchenko et al. 926* (LE); Sukhum, on the right bank of the River Besleta, near the famous factory, 29 Jul 1951, *Vasiliev* s.n. (LE).

**Germany**. **Bayern**: Oberbayern, München, obhere Speilbahn des Golfplatzes Thalkirchen 1 km sudlich des Asam-Schlössels, 550 m, 12 Jul 1992, *Förther 48* (B); Lkr. Ostallgau, NW Trauchgau, 845 m, 24 Jul 2001, *Herb. Willing [Willing & Willing] 13418 D* (B); Lkr. Garmisch-Partenkirchen, SW Krün, 911 m, 5 Aug 2001, *Herb. Willing [Willing & Willing] 15543 D* (B); **Berlin**: Pichelswerder, Bez. Spandau, 35 m, 4 Jun 1998, *Herb. Willing [Eisenblatter & Willing] 4624* (B); **North Rhine-Westphalia**: Münster in Westfalen, *Wilms* s.n. (BM); **Rheinland-Pfalz**: Westerwaldkreis, Seeburg, westerwalder Seenplatte, Haidenwalder bei Seeburg, 421 m, 2 Jul 1978, *Kalheber 78-424* (B); **Schleswig-Holstein**: Rendsburg, *Larsen et al. 106* (BM, LE, MA); **Thüringen**: Am Ufer des Altenberger Teiches bei Eisenach, 28 Jul 1947, *Launert* s.n. (BM).

**Greece**. Thracia, prov. Xanthi, in valle infra pagum Dhamari, 4 km infra junctionem torrentium, 500 m, 16 Aug 1978, *Greuter 16361* (B); Dhrama, prope phylacion nom. Praseki, a pago Ano Vrondous, ca. 6 km boreo-occidentum versus, 1320 m, 22 Aug 1978, *Greuter 16703* (B); Mt. Olympe, Thess, Jun 1929, *Guiol 642* (BM); Etolia-Akarnania, Arahova (Ar 358), 900 m, 8 Jul 1996, *Herb. Willing [Eisenblatter & Willing] 48029* (B); Thesprotia, Ep. Souliou, NW Paramithia (The 209), 190 m, 26 Jun 1997, *Herb. Willing [Eisenblatter & Willing] 55434* (B); Trikala, Ep. Kalambakas, Notia Pindhos, NW Kalambaka (Tri 200), 330 m, 18 Jul 1997, *Herb. Willing [Eisenblatter & Willing] 60744* (B); Nom. Ionnanina, Ep. Dhodhonis, Tsepelovo (Ioa 574), 1000 m, 17 Jul 1998, *Herb. Willing [Eisenblatter & Willing] 67904* (B); Nom. Kastoria, Ep. Kastorias, SW Vyssinia (KAS 164), 820 m, 31 Jul 1998, *Herb. Willing [Eisenblatter & Willing] 71725* (B); Pella, Ep. Edhessis, W Loutraki (Pel 115), 260 m, 27 Jul 2000, *Herb. Willing [Eisenblatter & Willing] 86148* (B); Arkadhia, Elati, 1180 m, 27 Sep 2003, *Herb. Willing [Willing & Willing] 118384* (B); Paranestion, Nomos Drama, Dimos Paranestion, river Nestos ca. 1 km SW of Paranestion, 100 m, Aug 1996, *Liebfritz & Schuler* s.n. (B); Nomos Kavala, Dimos Keramoti, mouth of river Nestos, 1 m, 31 Aug 1998, *Schuler 98/447* (B); Ipiros, prob. Ioannina, distr. Konitsa, Konitsa, on both sides of the Aoos River above the bridge, 460 m, 23 Jul 1971, *Stamatiadou 13382* (BM).

**Hungary**. Sin. loc., Jul 1877, *Anonymous* s.n. (BM); Kozragohid, Budapest, 26 Aug 1904, *Degen* s.n. (LE).

**India. Jammu and Kashmir**. Kashmir, Kishtwar, 1829 m, 17 Sep 1876, *Clarke 31361* (BM); above Barangalla, 2000 m, 25 Jun 1902, *Drummond 13886* (E); Gandarbal, Sind valley, 1585 m, 1 Jun 40, *Ludlow & Sherriff 8101* (BH, BM, E); Hajipir pass, Poonch to Uri, 2134 m, 4 Jul 1934, *Stewart 14000* (A); Naranag, Wangat valley, 2134 m, 8 Aug 1939, *Stewart & Stewart 18104* (GH); Poonch & Azad Kashmir, below Hajiperi, 2134 m, 3 Jul 52, *Stewart & Nasir 24062* (BM).

**Iran**. Ghilan, Rescht, 13 Jul 1902, *Alexeenko 47* (LE); Fars, Tang-e-Sorkh, between Ardakan and Yasuj, 2450 m, 18 Jun 1977, *Bokhari & Edmondson 2159* (G); Meshed, 1941, *Mrs Donaldson* s.n. (K); Amol, 25 miles S of Amol, Central Elburz N. slopes, 1219 m, 21 Aug 1966, *Furse 9071* (K); . Keredj, 29 Sep 1934, *Gauba 829* (B); Kurdestan, 31 km N of Sanandaj, 2000 m, 8 Jul 1964, *Grant 16064* (MO); Dozein, 5 km N of Dozein, which is a village 55 km SE of Gonbad-e-Kabus, 950 m, 19 Jun 1977, *Hewer 3961* (K); Khairat, Mazenderan, 2439 m, 25 Jul 1940, *Koelz 16569* (US); Kuhi, near Teheran, Pidjehek, Jul, *Kotschy* s.n. (BM); Fars, 36 km NW Ordekam, 14 Jul 1959, *Pabot 2446* (G); Qotur River,W Khvoy towards the Turkish border, 1800 m, 10 Jul 1971, *Rechinger 41735* (B, G); Keredj, Elburs Mountains, Keredj River, 6 Jun 1937, *Rechinger 733* (S); Hamadan, 2134 m, 7 Sep 1929, *Rogers 538* (K); Joistan, Talighan Valley, 1829 m, Jul 1953, *Trott 24* (K); Astava, shoreline of Caspian Sea, 21 Aug 1966, *Wright 65* (K).

**Iraq**. Baghdad, Pig Island, 10 May 1960, *Agnew et al. W-1679* (K); Tigris Plain. near Mosul, 200 m, 5 May 1936, *Low 284* (BM); Penjiwin, 1200 m, 8 Jun 1948, *al Rawi 12159* (K, US); Pushtashan, 15 km NE of Rania, lower slope of Qandil Range, 1150 m, 29 Jul 1957, *al Rawi & Serhang 23865* (K); Erbil, Pushtashan, Montes Qandil, (Kurdistan), 1000 m, 28 Jul 1957, *Rechinger 11027* (G).

**Ireland**: West Galway, Connemara, Renville, *Babington 299* (BM); Cork, Bishopsbrook, 1849, *Herb. J. Carroll* s.n. (BM); South Kerry, Dooks, 15 Jul 1903, *Marshall* s.n. (BM); Meath, Drogheda, Aug 1956, *McClintock* s.n. (BM); North Kerry, Walls between Killarney and Mucross, 24 Aug 1883, *Ridley* s.n. (BM); South Kerry, Rossbeigh Dunes, 5 Aug 1935, *Simpson 35620* (BM); South-East Galway, near Dalystown, 24 Jun 1952, *Simpson 52108* (BM).

**Israel**. Gilead, Burmah to Gerashi, 4 May 1886, *Anonymous* s.n. (K).

**Italy**. **Abruzzo**: Teramo, cerca Castelli, Findo de al Salsa, 1100 m, 2 Jul 2002, *Navarro et al. 4348* (MA); **Apulia**: Puglia, farm about 7 km NE of Taranto, 1881, *Vitantonio 1675* (BM); **Emilia-Romagna**: Parma, *Anonymous* s.n. (BM); **Friuli-Venezia Guilia**: Trieste, Sistiana, 25 May 1980, *Greuter et al. 17348* (B); **Sicily**: Nebroda Mtns., ad pedes montium Madoniarum prope Polizzi, 600 m, 6 Jul 1874, *Strobl* s.n. (BM); Catania, Randazzo, Lago de Gurrida, 820 m, 5 Jun 2000, *Ávarez et al. 1490* (MA); **Tuscany**: Agro Lucchese, Lucca country side, Jun 1835, *Puccinelli* s.n. (BM).

**Japan**. **Hokkaido**: Sapporo, 4 Jul 1903, *Arimoto* s.n. (GH, MO); Iburi, Tomakomai, 30 Jul 1930, *Akiyama* s.n. (S); between Kotoni and Sapporo, 22 Aug 1929, *Dorsett & Morse 1128* (US); Yezo, prov. Nemuro, to Attoko-too from Attoko-Nemuro-gun, 8 Jul 1959, *Furuse* s.n. (A, S); on the coasts vicinity and Oikamanai-numa Bansei, Taiki-choo, Hirowo-gun, Province Tokachi, 19 Jul 1974, *Furuse 6434* (K); Wakkanai-Park. Wakkanai-shi. Province Kitami. Hokkaido ((Yezo)), 18 Aug 1975, *Furuse 9465* (K); Jyuusam-mura. Nishi-tsugaru-gun. Province Mutsu. Hondo, 20 Sep 1955, *Furuse 29993* (K); Watsakinai. Toyotomi-choo. Teshio-gun. Province Teshio. Hokkaido (Yezo), 2 Jul 1975, *Furuse 8980* (K); prov. Iburi, between Tomakomai-shi and Lake Shikotsu, 15 Aug 1981, *Takahashi 1650 -A* (NY); **Honshu**: Shimura, Itabashi-ku, Tokyo Prefecture, Jun 1907, *Makino 75314* (LE); Shiga, Yotsugawa, Adogawa Cho, Takashima gun, Lake Biwa, 86 m, 17 Sep 1991, *Kanda & Kuribayashi 1* (A); Hondo, Oze-ga-hara, prov. Kozuke, 26 Aug 1950, *Mizushima 417* (A); Aomori-shi, Arakawa, below Jogakura Spa, 810 m, 8 Aug 2000, *Yonekura 5960* (A).

**Kazakhstan**. West Kazakhstan Prov., Right side of river Ural, 2 km downstream of Kopseharov, 15 Feb 1955, *Grubov & Lynbarskii 32* (MO); South Kazakhstan Oblast, Shymkent, prov. Syr-Darja, Distr. Tschimkent, Tchiluk, 1908, *Knorring 604* (S); Tselinogradskaya Oblast’, near village of Kurgaljinskii, on the lower eastern shore of the Lake Kuraljin, in the surroundings of the settlement of Karazhar, 20 Jun 1980, *Sidorova 155* (LE).

**Lebanon**. Ain Zahatallah, Mt. Lebanon, Aug 1912, *Letchworth* s.n. (K).

**Lithuania**. Holonety, dist. Wilkomierz, 30 Jul 1895, *Rudiminowna* s.n. (B, BM).

**Luxembourg**. Jardin Botanique, 20 Aug 1837, *Gay* s.n. (K).

**Macedonia**. Popovo, NW foot of Krusa Balkan, 3 m E of Lake Doiran [Doirani], 1918, *Gooding* s.n. (BM); Kojnsko, distr. Duditza-et Saharupa-planina, 550 m, Jun 1917, *Schultze-Jena 138* (B).

**Mongolia**. southwestern Mongolia, Khovdinskii Aymak, southern slope of the Mongolian Altai, 45 km to the E of the villageg of Bulgan, along the River Bulgan, on the border with KNR [China], 1979, *Gubanof 7321* (LE); “ad Mongolia borealis, Altai australis ad Totyask nigrum”, 1876, *Potanin* s.n. (K).

**Morocco**. Xauen, 650 m, 28 May 1928, *Font Quer 354* (BM); Tazzeka, 13 km from Taza on minor road, Ras-El-Ma, 990 m, 5 Jul 1993, *Jury et al. 11718* (K).

**Norway**. Oslo, 24 km hinter, am fjord, 8 Sep 1980, *Meyer* s.n. (B); Stjordal, near Hagra, 1926, *Trethewy* s.n. (BM); Astre Biarum, 6 Sep 1902, *Triatz* s.n. (BM).

**Poland**. Wolka wysoka prope Sanniki, distr. Gostyn, 22 Jul 1895, *Drymmer* s.n. (BM); Orawicza, in Banat, 6 Aug 1840, *Wierzbicki* s.n. (GOET).

**Portugal**. Salvador, as margenes do rio Sever, Sera de S. Maccede, 12 Jun 1959, *Beliz & Raimundo 5046* (MA); inter Garvas et Panvies, Jun 1875, *Daneau 1184* (BM); Alto Alentejo, Elvas, Frequisia de Varche, Horto do Carlos Ranita, margem da Ribereira do Cancão, 30 Sep 1984, *Guerra 1518* (MA); Beira Alta, Vizeu, Santa Comba-Dão, Pinheiro de Azer, descida para a capela de Sra. do Pranto, 11 Jun 1982, *Marques 2087* (MA); Rangel prope a Coimbra, Jun 1889, *Moller* s.n. (BM); *Oliveira, M.P. de*, *s.n.* (BM); Entremadura, Obidos nas margens dos vales na La Goa de Obidos, 14 Jun 1917, *Rainha 377* (MA); Idanha a Nova, Ribeira do Poasul, Jul 1883, *Ricardo da Cunha* s.n. (BM); Alijó, Pinhão, beira-Doura, foz do Rio Pinhão, 30 Jun 1070, *Rozeira et al.* s.n. (MA); Douro Litoral, near Oporto, 2 Jul 1887, *Murray* s.n. (BM); Madeira, sin. loc, 1804, *Turner* s.n. (K); Tagum, 1848, *Welwitsch 202* (LE).

**Romania**. Comm. Balcani, prope pagum Frumoasa, 550 m, 12 Jul 1972, *Barabas 487* (BM).

**Russian Federation**. **Central Federal District**: Moscow, 30 Aug 1992, *Croat 73991* (MO); **Far Eastern Federal District**: Amurskaya Oblast’, Radde, around the village, on the River Amur, 25 Sep 1926, *Selivanova* s.n. (LE); Amurskaya Oblast’, Zea, Zeyskii region, around the town of Zea, 30 Jul 2000, *Shaligin* s.n. (LE); Dalnevostochnii Krai, near Valdivostock, 12 Jul 1929, *Transhel 284* (LE); Kurilskii Islands, Iturup [illegible], 914 m, 29 Aug 1946, *Vorobeev* s.n. (LE); Archangelsk Oblast, Archangelsk, Beresnik, 1919, *Grantham* s.n. (BM); around Vladivostock, on the bank of Lake Khanka, 10 Aug 1929, *Shishkin 1306* (LE); island of Sakhalin, village of Vladimirovskoe, 26 Jun 1899, *Shestunov 53* (LE); Primorskaya Oblast’, around 25 verst [measure of distance] to the west of Vladivostock, on the slope of a mountain, 29 Jun 1926, *Ivanova* s.n. (LE); Primorskaya Oblast’, Khabarovsk, left bank of the River Amur, near Khabarovsk, 25 Jul 1926, *Solokhin* s.n. (LE); Primorskaya Oblast’, Petrovolikova Bay, island of Furugelma, 21 Sep 1922, *Kozlov 483* (LE); Primorskaya Oblast’, the village of Chernshovekoe [Chernyshevka], 30 Jul 1902, *Paltzevskii* s.n. (LE); Sakhalin Oblast, on the sea slope of southern Sakhalin, between the settlements of Khvostovo and Altasovo, 13 Aug 1959, *Pobedinova & Konovalova 1347* (LE); **North Caucasian Federal District**: Tegenekli, basin of the River Baksan, right side of the gorge Prielbrusye, 23 Aug 2000, *Menitskii & Popova* s.n. (LE); Nalchik, to the south of the settlement of the village Belaya Rechka, 2 Aug 2000, *Menitskii & Popova* s.n. (LE); [Chechnya] Itun-kale, Argun, left side of river Argun, above the village, 23 Aug 1988, *Menitskii & Nikolaev 38* (LE); [Dagestan] Machachkala, by the river Terstagay, 26 Sep 1926, *Orlov* s.n. (LE); [Dagestan] Gergebilskii region, between villages of Arakani and Mogok, 14 Jun 1961, *Tsvelev et al. 914* (LE); [Dagestan] Levashinskii region, between villages of Gersibil and Levashy, 27 Jul 1961, *Tsvelev et al. 4175* (LE); Chechyna, Terekaya Oblast’, Beslan, May 1901, *Alexeenko 14374* (LE); Kabardino-Balkar, Bezeniyskoe gorge, on the slope of the mountain Ushtensk, 12 Aug 1995, *Shkhagapsoev & Korchevskaya 675-2000* (LE); Ordzhonikidzevskii, “Chechnya-Ingushetia”, 22 Jul 1966, *Vlasov & Kuvas* s.n. (LE); slope of the left bank of the River Bizhgon 10 km S of the station Storozhevaya, 31 Aug 1947, *Borisova 369* (LE); Stavropolskii Krai, bank of River Podkushok in the region of Pyatigorsk, 8 Aug 1990, *Yagushevskii* s.n. (LE); Terekaya Oblast’, Nalchik, near town, 30 Jun 1917, *Paltseva* s.n. (LE); **Northwestern Federal District**: Ladozhskoe Lake, town Lakhdempokhye, 6 Jul 1961, *Pobedimova & Gladkova 97* (LE); near St. Petersburg, pag. Olgino, in litore sinus Fennici, Jun 1919, *Czernjakovskaja* s.n. (BM); **Siberian Federal District**: Siberia, regio transbaicalensis, Utotschkina, Selenga River, 500 m, Jul 1900, *Ehnberg* s.n. (G); Irkutsk Oblast, Irkutsk, *Besser* s.n. (K); Krasnoyarsk Krai, Yeniseysk, “Siberia, Jenisei, Jeniseisk”, 23 Jun 1876, *Arnell* s.n. (S); Central Siberia, region of Novosibirsk, near railway station Seyatel’ ca. 3 km Nw of Akademgorodok near river Ob’, 2-3 km below hydroelectric dam, ca. 20 km SSE of Novosibirsk, 140 m, 24 Jul 1979, *Iltis et al. 1115* (G, S); Podkova, Yekaterinava, 21 Jun 1906, *Yálovaya* s.n. (LE); **Southern Federal District**: Mineralnie Vody, eastern slope of the mountain Zmeyka, 7 Aug 1996, *Menitskii & Popova 122* (LE); River Mzymty [Myzmyta] near the mouth of River Agipse, 7 Sep 1928, *Leskov* s.n. (LE); Tuapsinsk, Black Sea Region, 8 Jul 1915, *Litvinov* s.n. (LE); [Adygea] Kubanskii Oblast’, Maykopsk, between the stations of Dondukovkskaya and Sergieskaya, in the valley of the River Fars, May 1915, *Kozo-Polyanski & Preobrasheiski* s.n. (LE); Krasnodavskii Krai, Novorossika, Black Sea region, near the road on the high shore of the sea, 25 Jun 1923, *Pojarkova 156* (LE); **Urals Federal District**: Goygol Rayon, Goygol, territory of Elizabethapolis [Yekaterinburg], 8 Jun 1844, *Kolenati 1753* (LE).

**Serbia**. mountain above Jviato Petka, 22 Aug 1921, *Yermoloff* s.n. (BM).

**Slovenia**. within 6 miles of Bled (in Slovenja, at the borders with Austria), which includes the lower slopes of Mt.Triglav, 304 m, 1923, *Leathes* s.n. (BM).

**Spain**. **Alcabete**: Reolid, 800 m, 19 Aug 1985, *Figuerola* s.n. (MA); **Andalucia**: Granada, Castril, pr. Cortijo del Nacimiento, 1100 m, 21 Jul 1978, *Charpin et al. 15109* (MA **Asturias**: Villaviciosa, playa de España, 5 m, 5 Oct 1992, *Aedo 2469* (MA); **Avila**: Conabeltrán (?), Jul 1918, *Cogolludo* s.n. (MA); **Baleares**: Mahón, San Sancho, 18 Apr 1899, *Pons*, *Gereau* s.n. (MA); **Burgos:** Carazo, junto a la Fuente de la Mora, Peñas de Cervera y aledaños, 1200 m, 24 Jul 1979, *Pons Sorolla & Susanna 616* (MA); **Caceres**: Baños de Montemayor, 15 May 1944, *Cabellero* s.n. (MA); **Cadiz**: collected in a farm named ‘Marcellino’ in a rough country between Los Barrios and Algeciras, 60 m, 14 Jun 1973, *Brinton-Lee 1346* (BM); **Cantabria**: Santander, Cueto, El Panteón del Inglés, 22 May 1992, *Busqué* s.n. (MA); **Castellón**: Villafarnés, on same sheet several specimens, also from Castielfavib and Vallanca (Valencia, 1792, 1793), and Jardin de la Chevette (France, 1781), 1792, *Anonymous* s.n. (MA); **Castilla**: Bujedo, 28 Jul 1920, *Elías* s.n. (BM); **Catalunya**: Castello, Castillo de Villamalefa, Río Villahermosa, 540 m, 22 Oct 1998, *Riera et al. 19505* (MA); Cerdagne, Llivia, val de l’Estahuja, 1300 m, 10 Aug 1918, *Sennen* s.n. (BM); **Cuenca**: Hoz de Cañizares, 10 Jul 1932, *Cabellero* s.n. (MA); Tarancón, 7 Apr 1977, *López 8* (MA); **Cuidad Real**: Frencaliente, margenes del Río Pradillo, 600 m, 7 Jul 1997, *García Río* s.n. (MA); **Galicia**: Lugo, Riberas de Lea, 25 Jul 1956, *Carreira* s.n. (MA); **Guadalajara**: Zaorejas, Hoz del Tajo, piscifactoria del Campillo, 900 m, 18 Jul 1981, *Muñoz 512* (MA); **Huelva**: Almonte, Coto de Donaña, Reserva de La Rocina, 28 Jun 1977, *Castroviejo & Porta 733* (MA); **Madrid**: San Agustín de Guadalquix, riberas del Río Guadalquix, 3 Aug 1982, *Moreno* s.n. (MA); **Navarro**: Pamplona, 16 Jun 1971, *Moriyóu* s.n. (MA); **Salamanca**: alrededores de Bejar, Aug 1914, *Cogolludo* s.n. (MA); **Segovia**: Granja de San Ildefonso, desde la finca La Sauca hacia la carretera a Torrecabelleros, 30TVL1529, 1150 m, 9 Sep 1985, *García Adá & Egido 1816* (MA); **Soria**: Cañon del Río Lobos, 14 Jul 1980, *Buades* s.n. (MA); **Tarragona**: 20 km SW of Tarragona, between Cambrils and Hospitalet, 29 May 1962, *Brummitt et al. 298* (BM); **Toledo**: ribera del Río Tajo, 500 m, 18 Jun 1983, *Egido 947* (MA); **Valencia**: Bco. Bocairente, 510 m, 7 Jul 1980, *Palasí* s.n. (MA); **Valladolid**: Encinos de Esqueva, en Arroyo de los Frios, 11 Jul 1983, *Fernández Alonso 2071* (MA); **Zamora**: Parque Natural Lago de Sanabria, Forcadura, 1100 m, 1 Jul 1987, *Anonymous* s.n. (MA).

**Sweden**. Uppland, ad sopes Upsalia passim, Aug 1870, *Ahlberg* s.n. (BM); Uppland, Funbo Parish, southern shore of Lake Goren, 6 Jul 1944, *Alm & Smith 272* (BM); Bohnslam, Väderöarna, 10 Jul 1909, *Almquist* s.n. (BM); Brohuslan, Fjalibacha, 3 Jul 1910, *Almquist* s.n. (BM); Uppsala, *Andersson* s.n. (MA); Skafto, Gaso, 20 Jun 1948, *Ankarsward* s.n. (BM); Södermanland, Toiosocken, Stora Grasskar, 6 Aug 1922, *Asplund* s.n. (BM); Västergötland, Goteborg, 14 Aug 1959, *Blom* s.n. (BM); Skåne, Buutofha, Jul 1893, *Johansson* s.n. (BM); Scania, Kivik, Aug 1911, *Johansson* s.n. (BM); Malmo, Aug 1886, *Johansson* s.n. (BM); Östergötland, Linköping, Sep 1912, *Johansson* s.n. (BM); Sm. Oscasthaven, 4 Aug 1909, *Köhler* s.n. (BM); Yonstrup, Jul 1861, *Mortensen* s.n. (BM); Södermanland, Paroecia Strängnäs, Sundby, 27 Jun 1925, *Samuelsson 1371* (BM); Bohuslän, Högås Parish, the innermost part of the bay Svältekilen, 14 Aug 1960, *Santesson 13962* (BM).

**Switzerland**. **Vaud**: Montherod pres d’Aubonne, *Anonymous* s.n. (BM); inter Nyon et Lausanne ad lacum Lemanni, Jul 1838, *Sander* s.n. (BM); **Zürich**: Rümlang, Gemeinde Rümlang, Näherer Einschlag, 420 m, 20 Jun 1947, *Bührer* s.n. (MA).

**Syrian Arab Republic**. Monts Arnanis, 762 m, Aug 1906, *Haradjian 481* (K); Zahleh, Cacle [coll. Prof. Post, ex. Herb. J.T. Rothrock], *Post* s.n. (F).

**Tajikistan**. Giesar, Pamiro-alay, near the village of Giesar on the river Khanaka, 31 Aug 1942, *Grigoriev 757* (LE).

**Turkey**. Ouchak, Phrygie, Bords du ruisseau d’Ouchak (Phrygie), 910 m, 9 Jul 1858, *Balansa 1137* (A, B, BM, K); Cumasia, 24 Apr 1889, *Bornmüller 344* (B); Prov. Konya distr., Ermeneh (Cilican tracks), Sorivandt, 1200 m, 15 Aug 1949, *Davis 16221* (K); Ankara prov., Hacikacum Valley, 11 Jun 1952, *Davis & Dodds 18783* (BM, K); Cankiri-Kalecik, 9 Jun 1954, *Davis & Polunin 21737* (BM); B9 Agri, Murst Valley, 3-5 km from Tutak to Hamur, 1600 m, 2 Jun 1966, *Davis 44044* (K); Suluçem, (Musum) S edge of Balik G, 2300 m, 23 Jul 1966, *Davis 47185* (K); C10 Hakkari, Nehil çayi, 48-55 km from Hakkari to Yuksekova, 1600 m, 14 Jun 1966, *Davis 44905* (K); Cankiri-Kalecik, 9 Jun 1954, *Davis 21737* (K); Tokat-Artova, 4 Sep 1954, *Davis & Polunin 24854* (K); Abant Gol, Prov. Bolu, 1400 m, 11 Jul 1962, *Davis & Coode D-37291* (K); Prov. Zonguldak, Bartin-Amasra, 25 Aug 1960, *Khan et al. 796* (K); Çibuktal, bei Ankara, 10 Jun 1932, *Kotte* s.n. (K); Adapazari, *Kuntay 8735* (MO); Dorukhan geçidi, Nord-seite (A4 Zonguldak), 800 m, 8 Aug 1982, *Raus 6964* (B); Takiansi, 304 m, 29 Aug 1878, *Regel* s.n. (LE); Yagan, Erzerumskii sandzhak, river Arakas above Yagan, 9 Jul 1916, *Sapozhnikov & Shishkin* s.n. (LE); River Fol-chai, Trapezundskii sandzhak, 28 Jun 1917, *Shishkin* s.n. (LE);Vitse, Lazistan, 12 Aug 1912, *Shishkin* s.n. (LE); Pamphylia, Antalya, 23 Jun 1936, *Tengwall 48* (GH, K, S); Bolu, Koru Motel, 850 m, 30 Aug 1972, *Uotila 20137* (G); Ismid, (Nicaea), 1834, *Wiedemann* s.n. (LE); vicinity of Tortum Lake. East Turkey, 30 Jul 1961, *Winter 278* (BM).

**Turkmenistan**. Germad, 8 Jun 1899, *Antonov* s.n. (LE); gorge Verkhanyaya Firyuza, central Kopet Dag, 31 Aug 1945, *Blinoskii* s.n. (LE); going up the river Dyuynee near the spring Autet, 1200 m, 25 May 1928, *Bobrov & Yarmelenko 168* (LE); Karasashinskii region, river Gebe-Zvud, 14 Jul 1931, *Borieova 472* (LE); Zakaspiiskaya Oblast, Shorkala [Shor-Kala], Alta-Yaba, aul Shorkala, 23 Jun 1924, *Bukinich 45* (LE); Kopet Dag, road Utsch-Kuyu Nukhur, 28 Jun 1925, *Fedchenko et al. 790* (LE); Germad, on the banks of the river Saki[l]yad, near village of Germad, 13 Jul 1934, *Gnezdillo 97* (LE); Zakaspiiskaya Oblast’, Askabadskii area, mountains of Kopet-Dag, gorge of Chuli, 4 May 1912, *Lipskii 1807* (LE); Zakaspiiskaya Oblast’, Askabadskii area, mountains of Kopet-dag, Chuli, 17 Jun 1911, *Mickelson 487* (LE); Zakaspiiskaya Oblast’, [Finshchi], Jun 1895, *Minkevich* s.n. (LE); Tilutschi, 4000 m, 23 Jul 1879, *Regel* s.n. (K); Zakaspiiskaya Oblast’, Chuli Mountains, 26 Jun 1912, *Samokish 1373* (LE); Zakaspiiskaya Oblast’, Chuli Mountains, near Askabad, 9 Jun 1911, *Sevdturabov* s.n. (LE); Kisil-arwat, Karakala, by a little river the valley of [Foltere], 10 Jul 1901, *Sintenis 2035* (B, LE).

**Ukraine**. Kiev, 28 Aug 1906, *Lonaczewsky* s.n. [*7272a*](BM, MO); Zakarpatskaya Oblast’, Uzhgorodsky Raion, road to Imina (Transcarpatia Province, Uzhgorod District), 26 Jul 1967, *Apentyeva* s.n. (K).

**United Kingdom**. **Channel Isles**: Guernsey, Petit Bo, Jul 1883, *Fawcett* s.n. (BM); Jetou, 26 Jun 1932, *Meinertzhagen* s.n. (BM); Sark, 8 Jul 1953, *Sowerby 4* (BM); **England**: Huntingdonshire, Woodwalton, 7 Jun 1914, *Adamson* s.n. (BM); Leicestershire, Scraptoft, 15 Aug 1949, *Bangerter* s.n. (BM); Warwickshire, Between Pillerton Priors and Eatington; Ettington parish, 9 Jul 1961, *Bangerter 1058* (BM); North Wiltshire, Calne, 27 Aug 1914, *Barton* s.n. (BM); Nottinghamshire, Cresswell Gorge, east of Cresswell, 60 m, 22 Jul 1963, *Bowden & Hillman 137* (BM); Yorkshire, Spern Point, 18 Aug 1070, *Castroviejo* s.n. (MA); Buckinghamshire, Aston Clinton parish, Dancers End Nature Reserve, 16 Aug 1942, *Dandy, J.E*., *943* (BM); Herefordshire, Startop’s End Reservoir. Tring Rural parish, 20 Jun 1943, *Dandy 974* (BM); East Suffolk, Corporation Marshes, Walberswick; Walberswick parish, 6 Jun 1961, *Elword 19* (BM); Kent, Wrotham, 21 Aug 1913, *Fox 1200* (BM); West Cornwall & Scillies, Tresco harbour, Isles of Scilly, 9 Jun 1963, *Gerrans 1098* (BM); Cornwall, Daymer Bay, Trebetharick, near Weybridge, 8 Jul 1967, *Halliday 163 /67* (LE); East Gloucestershire, Clapton, 9 Aug 1884, *Hanbury* s.n. (BM); West Kent, Woolwich Arsenal, 23 Aug 1894, *Herb. A.H. Wolley-Dod* s.n. (BM); Oxfordshire, Banbury, 3 Aug 1863, *Herb. Alfred French* s.n. (BM); South Essex, Romford, Jun 1914, *Herb. H. Stanley Redgrove* s.n. (BM); East Cornwall, Tintagel, 26 Jun 1907, *Herb. L.B. Hall 306-1134* (BM); West Cornwall & Scillies, Lizard, 27 May 1886, *Herb. R.P. Murray* s.n. (BM); West Gloucestershire, over Sapperton Tunnel, 18 Jun 1908, *Herb. W.C. Barton* s.n. (BM); Lancashire, road to Newland, *Hodgson 738* (BM); Berkshire, Speen Moors near Newbury, 8 Sep 1894, *Jackson* s.n. (BM); East Norfolk, Lopham Middle Fen, 3 Jul 1973, *Jones & Vickery 20* (BM); Hampshire, Southampton, edge of The Avenue, Southampton Common, 17 Jul 1964, *Kerr 220* (LE); South Lancashire, Nr. Freshfield, Ainsdale Dunes Reserve, 26 Jul 1966, *Kendrick 30* (BM); Suffolk, Brome, 19 Jul 1832, *Kirby* s.n. (BM); Durham, Hartlepool, North sands, Sep 1872, *Lees* s.n. (BM); West Lancashire, Lancaster, Shingle at Bare, Morecambe Bay, 27 May 1912, *Lees* s.n. (BM); Lincolnshire, Skegness, 24 Aug 1904, *Linton* s.n. (BM); North Essex, Frinton to Clacton, 14 Sep 1889, *Linton* s.n. (BM); South Hampshire, Lymington, Pilewell, 24 Aug 1893, *Linton* s.n. (BM); East Kent, Wittersham, May 1927, *Meinertzhagen* s.n. (BM); Norfolk, Sutton Broad, Sep 1928, *Meinertzhagen* s.n. (BM); North Devon, Woolacombe, 14 Sep 1931, *Meinertzhagen* s.n. (BM); South Somerset, Knowle, Jul 1881, *Painter* s.n. (BM); Staffordshire, Knypersley, Jul 1886, *Painter* s.n. (BM); Hertfordshire, Hadley Wood, 16 Jul 1882, *Parker* s.n. (BM); West Norfolk, Thompson, 12 Aug 1917, *Robinson* s.n. (BM); South Devon, Dartmouth, 30 Jul 1952, *Robson 350* (BM); Dorset, Pentridge, Cranborne Chase, 19 Jun 1934, *Simpson 34161* (BM); West Sussex, Chichester, Kingley Vale, West Stoke, 12 Aug 1905, *Standen* s.n. (BM); Cheshire, Parkgate, 12 Oct 1965, *Valdés* s.n. (MA); Surrey, Farncombe near Godalming, edge of R. Wey, 14 Aug 1952, *Welch 4613* (BM); North Somerset, Bristol, Clifton Down, 15 Jul 1929, *White* s.n. (BM); Derbyshire, Monsal Dale, near the station, 21 Jun 1916, *Wilmott 708* (BM); Middlesex, Buckingham Palace garden, 9 Jun 1995, *Wiltshire* s.n. (BM); **Isle of Man**: Andreas, 22 Jun 1929, *Paton* s.n. (BM); **Scotland**: East Lothian, *Anonymous* s.n. (BM); Fife, Burntisland, 12 Jun 1847, *Anonymous* s.n. (BM); East Sutherland, Creich, road between Invershin and Bonarbridge, 20 Aug 1958, *Groves 2838* (BM); East Lothian, Haddington, Longniddry, 1850, *Herb. J.T.I. Boswell-Syme* s.n. (BM); West Perthshire, Clackmannanshire, Dollar [v.c.87], 1851, *Herb. J.T.I. Boswell-Syme* s.n. (BM); Mid Ebudes, Mull, near Nun’s Pass, west of Carsaig, 24 Sep 1967, *Kenneth & Stirling* s.n. (BM); Renfrewshire, Crossmyloof, 14 Aug 1953, *Mackechnie* s.n. (BM); Easterness, Beauly, 5 Aug 1892, *Marshall* s.n. (BM); near Mull of Kintyre, 2 Sep 1961, *McClintock* s.n. (BM); South Aberdeenshire, Jul 1947, *Robson 349* (BM); **Wales**: Pembrokeshire, Pembroke, Lydstep, 28 Aug 1907, *Bickham 1134* (BM); Radnorshire, Gladestry, Jul 1951, *Edwards 5* (BM); Glamorgan, Sully Island, Jul 1905, *Herb. H.J. Riddelsdell* s.n. (BM); Merionethshire, Dolgelley, [v.c.48], 15 Aug 1923, *Herb. W.C. Barton* s.n. (BM); Radnorshire, Between Erwood and Aberedw, 17 Aug 1885, *Ley* s.n. (BM); Holyhead, Anglesey, 4 Aug 1955, *Robertson D-11* (LE).

**United States of America**. **California**: Shasta County, Fall River Hills, 3 miles SW, 4 Jul 1934, *Howell 12411* (GH); **District of Columbia**: Washington DC, National Arboretum, 15 Jul 1965, *Mazzeo & Meyer 1090* (G); **Idaho**: Boise, 18 Jul 1911, *Clark 345* (G, GH); Power County, Meader’s Trout Farm, 28 May 1961, *Thiel* s.n. (LE); **Illinois**: Kane County, Elgin, north part, 24 Jul 1928, *Benke 4878* (GH); McHenry County, Fox River Grove, 18 Jun 1934, *Benke 5661* (GH); Lake County, Lake Villa, 3 Aug 1906, *Gleason & Shore 123* (GHDu Page County, Morton Arboretum, near Lisle, 26 Oct 1922, *Palmer 22347* (A); **Indiana**: Wells County, Harrison TP, 31 May 1903, *Deam* s.n. (GH); Noble County, Kendalle, Martin Farm about 2 miles southeast of Kendalle, 23 Aug 1928, *Dean 46131* (GH); Berrien County, Niles, 17 Jun 1933, *Palmer 40391* (A); Putnam County, Greencastle, East Seminary Street, 1 Sep 1940, *Welch 6120* (GH); **Maine**: Washington County, Lake View Campground, Moose Island, along western passage into Passamaquoddy Bay, N of East Port, 10 m, 30 Aug 2000, *Nee & Atha 50937* (BM); **Maryland**: Cumberland, Jul 1849, *Prior* s.n. (K); **Massachusetts**: Hampden County, Knightville Dam, vicinity of overlook on Hwy. 112, about 1.3 mi N of Knightville, 23 Jun 1984, *Bates & Elsik 83* (LE); Berkshire County, Tyringham Valley, 10 Sep 1926, *Meredith* s.n. (LE); Suffolk County, Franklin Park, 7 Jul 1883, *Seymour* s.n. (USM); **Michigan**: Mason County, Hamlin Lake, Ludington, 25 Jul 1910, *Chaney 91* (GH); Flint, Aug 1877, *Clarke 2195* (K); Cheboygan County, Douglas Lake, northern end of Lower Peninsula, *Gleason 269* (K); Clinton County, East Lansing, 7 miles northeast near route 78, 9 Sep 1955, *Perdue 2016* (GH); **Montana**: Powell County, Grant Kohrs Ranch National Historic Site, Deerlodge on US Hwy 10, 1373 m, 12 Aug 1983, *Ray 194* (GH); **Nevada**: Washoe County, Truckee River, 3-7 miles W of Sparks, 1219 m, 6 Aug 1937, *Moore & Franklin 929* (GH); **New Jersey**: Union County, Summit, Passiac River, 5 Jun 1969, *Hellquist* s.n. (GH); Cape May County, Five-mile Beach, 3 Oct 1899, *MacElwee 1448* (GH); **New York**: Tompkins County, Ithaca, Dryden Road, near Dwyer’s Bridge, 12 Jun 1916, *Bechtel 7126* (GH); Washington County, Lake George, N. Beaver Creek, Vaughn’s, north of Hudson Lake, 12 Jun 1913, *Burnham* s.n. (GH); Westchester County, Yonkers, Oatskill Aqueduct, 10 Jul 1934, *Gleason et al. 1404* (LE, MA); Tompkins County, in creekbed of Taughannock Creek upstream from the falls, 24 Sep 1983, *Kearney 132* (BM); Bronx, New York Botanical Garden, in the Swale, 28 m, 9 Jun 2006, *Nee & McClelland 54448* (G); Cayuga County, Lake Como, 15 Jun 1919, *Randolph & Wiegand 12864* (GH); Orange County, Aleck Meadow Reservoir, Black Rock Forest, 6 Jul 1936, *Raup 7502* (GH); Yates County, Canandaigua Lake, south end, 19 Aug 1933, *Swartley 66* (A); Delaware County, North Harpersfield, 11 Jun 1906, *Topping 137* (A); **North Carolina**: Haywood County, Waynesville, US 276 near city limits, W side of highway, 823 m, 16 Aug 1969, *Pittillo & Hensley 3102* (GH); **Ohio**: Butler County, Oxford, south of Oxford, 13 Jul 1958, *Cobbe m-52* (G); Portage County, Garrettsville, 15 Jun 1895, *Webb 68* (GH); **Oregon**: Multnomah County, St. Johns, near slough below St. Johns, 23 Jun 1915, *Gorman 3575* (K); Polk County, vacant lot back of Taylor’s, 30 May 1960, *Murata 71* (GH); Willamette County, Salem, 16 Jul 1917, *Nelson 1684* (GH); Washington County, Hillsboro, 23 Aug 1911, *Smith 4093* (GH); **Pennsylvania**: Northumberland County, Turbotville, 4 mi NNW of town, 13 Aug 1940, *Adams 5416* (A); Berks County, McKnight’s Gap, 3/4 mile SW of town, 164 m, 12 Jun 1941, *Berkheimer 2565* (GH); Buchs County, Riegelsville, 3 mi below (Kintnerville), 27 Jun 1925, *Dreisbach 3573* (LE); Pike County, Kimble, along Lackawanna River, 24 Jun 1936, *Fogg 10784* (A); Montgomery County, Jenkintown, 28 Jun 1938, *Frazier* s.n. (A); Somerset County, Tire Hill, beside Pa. Highway 53, 0.9 km SSE of Tire Hill, 427 m, 9 Aug 1956, *Shetler 266* (LE); Fulton County, Needmore, 15 Jun 1947, *Wahl 2514* (A); **Utah**: Tooele County, Open Reventment Area, zone 12, 23 Jul 2000, *Long 1090* (GH); **Vermont**: Bellamy Falls, *Anonymous* s.n. (K); Leicester, 18 Aug 1907, *Dutton* s.n. (G); **Virginia**: Arlington County, Clarendon, Allard’s garden, 10 May 1938, *Allard 4653* (GH); Frederick County, Belle Grove, near Middletown, 23 May 1943, *Hunnewell 17854* (GH); Rappahannock County, Piney River, 27 Jun 1949, *Hunnewell 19218* (GH); **Washington**: Yakima County, Silah Valley, 29 Aug 1902, *Cotton 882* (GH); Skamania County, White Salmon, along Hwy 830 just W of White Salmon,, 10 Aug 1961, *Dennis 2323* (A, G, GH); King County, Seattle, 9 Aug 1935, *Eyerdam* s.n. (BM, G); Spokane County, Chattaroy, 12 Jul 1923, *Lackey* s.n. (G); Thurston County, Mima Prairie, along the Black River 3 miles south of Littlerock, 22 Jun 1939, *Meyer 1650* (GH); Whatcom County, Northwood Swamp, 26 Jun 1937, *Muenscher 8394* (GH); Whitman County, Bonnie Lake, north of Rock Lake, 29 May 1938, *Sharsmith 3570* (GH); **West Virginia**: Pendleteon County, Snowy Mountains, east slope, 13 Aug 1931, *Core* s.n. (GH); Smyth County, Marion, 29 Jun 1892, *Small* s.n. (K); Sweet Spring, 8 Sep 1903, *Steele & Steele 246* (GH); **Wisconsin**: Dane County, Rice Lake, 19 Jun 1926, *Fassett 2783* (GH); Iron County, Mercer, near outlet of Grand Portage Lake, 17 Sep 2000, *Nee & Atha 50993* (BM); Brown County, Kellogg’s Work Farm, 6 Aug 1902, *Schuette 96* (GH); **Wyoming**: Fremont County, Lander, along the Popo Agie River just south of city limits; overflow channel of river, 1615 m, 2 Aug 1965, *Scott 588* (GH).

**Uzbekistan**. on the right bank of the Amu-dari [Amu-Darya River] between two rocks, near [Kumksntau], 15 Jul 1873, *Karolkov & Krause* s.n. (LE); Karakalpakiya, near Amu-dari [Amu-Darya River], 22 Aug 1874, *Smirnov* s.n. (LE).

#### 15. Solanum dulcamaroides

Poir., Encycl. Suppl. 3: 751. 1814

http://species-id.net/wiki/Solanum_dulcamaroides

Figure 38

Solanum macrantherum Dunal, Solan. Syn. 16. 1816, nom. superfl. Type: Based on *Solanum dulcamaroides* Poir.
Solanum nutans Sessé & Moc., Fl. Mex., ed. 2, 50. 1894. Type: Mexico. “urbe Queretaro”, *M. Sessé & J. Mociño* s.n. (neotype, designated by Knapp, 2008b, pg.18: MA [MA-604674, F neg. 48343]).
Solanum sarmentosum Sessé & Moc., Fl. Mex., ed. 2, 51. 1894. Type: Mexico. “Habitat in Queretari et Temescaltepec hortis” *M. Sessé & J. Mociño* s.n. (lectotype, designated by Knapp, 2008b, pg. 18: MA [MA-604621, F neg. 48321]; isolectotypes: G [G00071088, F neg. 34123], MA [MA-604620, F neg. 48317], LE).
Solanum megalospermum J.L.Gentry & Child, Brenesia 16: 147. 1979. Type: Guatemala. Baja Verapaz: Sierra de las Minas, 5 km S of Purulhá, 1600 m, 2 Jan 1973 [“1963” on label at MO], *L.O. Williams, A. Molina R. & T.P. Williams 41964* (holotype: F [F-1986736, F neg. 62207]; isotypes: K [K000488211], MO [MO-2270289], NY [NY00139007]).

##### Type.

Mexico. Based on an unpublished illustration in the Sessé and Mociño collection (lectotype, designated here: Hunt Botanical Institute 6331.1503); Mexico. Sin. loc., *M. Sessé & J. Mociño* s.n.. (epitype, designated here: MA [MA-602624]).

##### Description.

Woody vine, often reaching into the canopy. Stems when young sparsely to densely pubescent with tangled, very weak simple uniseriate trichomes to 0.5 mm long, some trichomes furcate or dendritic in more pubescent individuals; new growth sparsely to densely pubescent with simple and furcate uniseriate trichomes, these tangled and weak, ca. 0.5 mm long. Bark of older stems yellowish brown, glabrate, corky on very large stems (fide *Nee 23735*). Sympodial units plurifoliate. Leaves usually simple, occasionally pinnatifid, especially on younger stems, (2.5-)4–10 cm long, (1-)2–8 cm wide, elliptic to obovate, usually widest in the basal third, slightly thick and fleshy, the upper surfaces glabrous and shiny with the trichomes confined to the veins or uniformly pubescent on the veins and lamina with simple uniseriate trichomes to 0.5 mm long, the lower surfaces glabrous or the pubescence similar to that of the upper surfaces, but the trichomes denser along the veins; primary veins 6–7 pairs, connected by a prominent marginal vein ca. 0.5 cm from the margin; base acute to truncate or very occasionally somewhat cordate; margins entire or with 1–2 pairs of basal lobes, the lobes 0.5–0.7 cm long, each with a petiolule to 0.4 cm long; apex acuminate, often with an elongate tip; petioles to 5 cm long, glabrous or pubescent like the stems and leaves, the trichomes denser on the channelled adaxial groove, twining to aid in climbing. Inflorescences terminal or lateral, 7–20(+) cm long, longer in fruit, many times branched, with up to 80 flowers, glabrous or pubescent with simple uniseriate and occasionally furcate trichomes like the stems; peduncle 1.5–5 cm long; pedicels 1–1.6 cm long, slender, ca. 1 mm in diameter at the base, 1–1.5 mm in diameter at the apex, deflexed or nodding at anthesis, glabrous or sparsely pubescent with simple uniseriate trichomes adaxially, minutely papillate abaxially, articulated at the base from a tiny sleeve, leaving a small peg on the inflorescence axis; pedicel scars irregularly spaced 5–10 mm apart. Buds globose and inflated with prominent angles at the petal margins, the corolla strongly exserted from the minute calyx tube long before anthesis. Flowers all perfect, 5-merous. Calyx tube 1.5–2 mm long, flattened to somewhat conical, the lobes < 0.5 mm long, forming mere teeth on the rim of the tube, glabrous to sparsely pubescent with simple trichomes, these denser on the minute apices. Corolla (2-)2.5–4 cm in diameter, violet to deep purple, very showy, stellate, lobed ca. 2/3 of the way to the base, the lobes 0.9–1.5 mm long, 0.6–0.7 cm wide, spreading to slightly cupped at anthesis, densely papillate all long the margins and on the cucullate tips, otherwise glabrous. Filament tube ca. 0.5 mm, the free portion of the filaments 1.5–3 mm long, glabrous; anthers 4.5–5 mm long, 3–3.5 mm wide, ellipsoid to almost globose, yellow, loosely connivent, the abaxial surfaces thickened and enlarged and the thecae not visible, drying papillate, poricidal at the tips, the pores not lengthening to slits with age. Ovary glabrous; style 14–16 mm long, glabrous; stigma small capitate, the surface minutely papillose. Fruit a globose berry, 2–2.5 cm in diameter, bright red and juicy when ripe, the pericarp thin and shiny; fruiting pedicels 1.5–2.5 cm long, 1–1.5 mm in diameter at the base, hanging from the weight of the fruit; fruiting calyx a flattened plate. Seeds ca. 20 per berry, 5–6 mm long, 4–5 mm wide, flattened reniform, reddish brown or pale brown, in immature fruit the surfaces minutely pitted, in mature fruits the lateral testal cell walls prominent and giving the seed a hairy appearance, these to 1 mm long and creating a prominent wing around the seed, the testa cells rectangular in outline. Chromosome number: not known.

**Figure 38.**
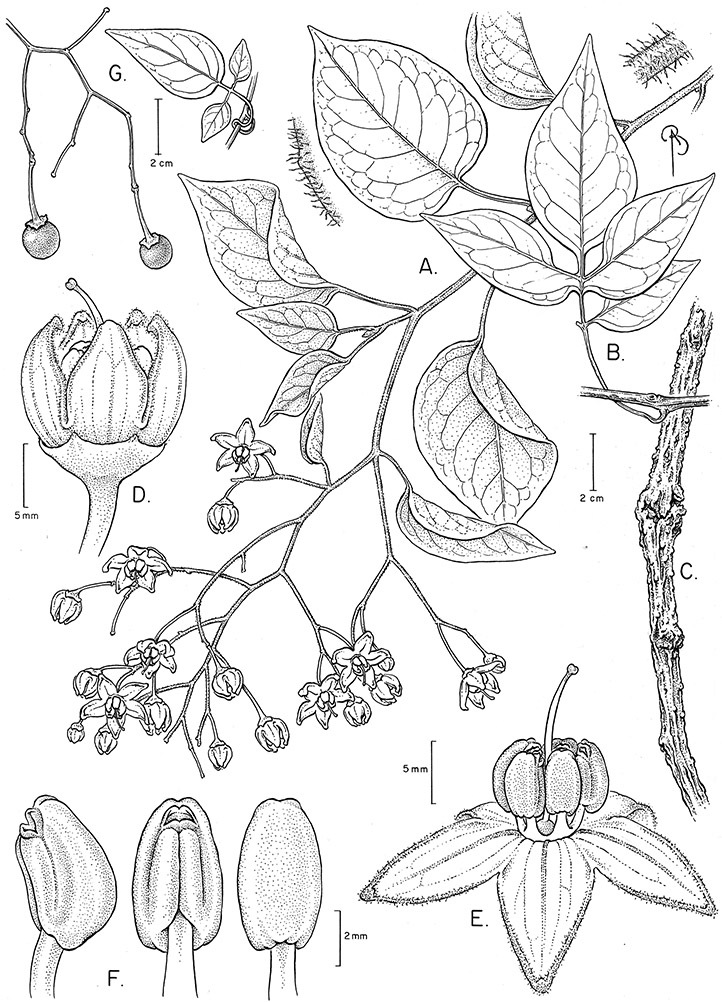
*Solanum dulcamaroides* Poir. (**A** drawn from *Saunders 8220* **B** drawn from Nee 23837 **C** drawn from *Nee 23735* **D–F** drawn from *Cortés 29* **G** drawn from *Lopez 340*). Illustration by Bobbi Angell.

##### Distribution

**Figure 39.**
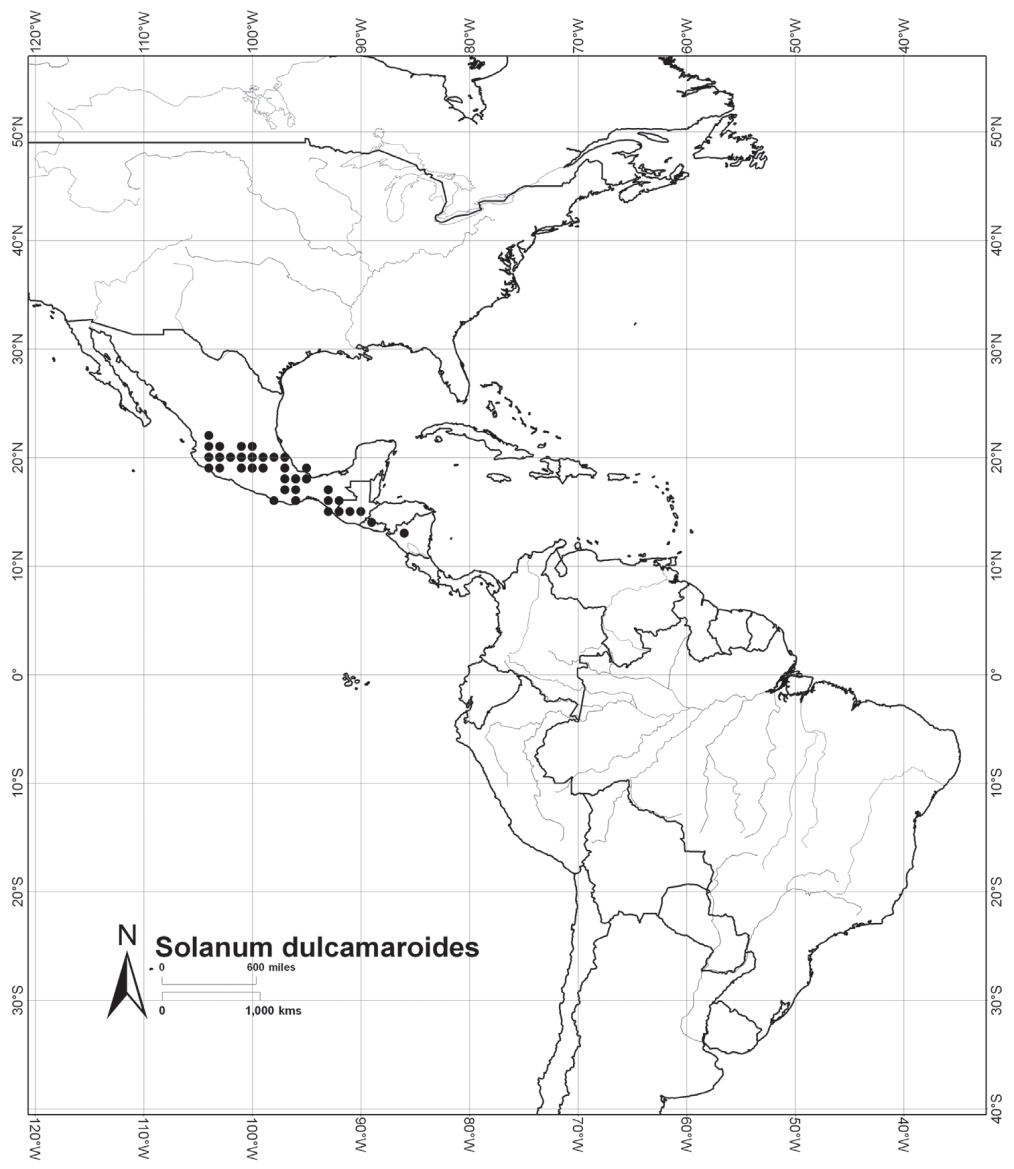
Distribution of *Solanum dulcamaroides* Poir.

(Figure 39). From central Mexico (Estado Colima) to Nicaragua; from almost sea level to 2100 m.

##### Ecology.

In deciduous and evergreen forests, often a canopy vine.

##### Conservation status.

Least Concern (LC); EOO >100,000 km^2^ (LC) and AOO >10,000 km^2^ (LC). See Moat (2007) for explanation of measurements.

##### Discussion.

*Solanum dulcamaroides* is a beautiful plant, with large, fleshy flowers and bright red fruits. It is one of the larger vining members of the Dulcamaroid clade, often growing well into the canopy of rainforests. Although it in some ways resembles *Solanum dulcamara* (as mentioned by Dunal 1813), it can be easily distinguished from that species by its inflated, globose angular buds (rather than the turbinate buds of *Solanum dulcamara*) and the much larger flowers (to 4 cm in diameter) without green spots at the corolla lobe bases. It also is somewhat similar morphologically to *Solanum boldoense* of Cuba (see Knapp 2008b for a discussion of the mix-up associated with these two species in the collections of Sessé and Mociño) and the more widespread and commonly cultivated *Solanum seaforthianum*. *Solanum dulcamaroides* differs from both those species in its distinctive anther morphology, with the abaxial surface thickened so that the thecae are not visible and the anthers appear as small football-shaped structures, and with pores that do not markedly lengthen to slits with age. The filaments of *Solanum dulcamaroides* and *Solanum boldoense* are equal, while in *Solanum seaforthianum* one filament is longer than the rest. *Solanum boldoense* has the articulation point of the pedicels just below the base of the calyx tube, while the pedicels of both *Solanum dulcamaroides* and *Solanum seaforthianum* are articulated at the base from a small sleeve. Pinnatifid leaves are rare in flowering or fruiting specimens of *Solanum dulcamaroides* (see below) and *Solanum boldoense*, while they are common in *Solanum seaforthianum*.

Although very few specimens of *Solanum dulcamaroides* have pinnatifid leaves, Nee (1993) records that divided leaves are borne on lower parts of the plant, and that plants cultivated from seed first produced profoundly pinnatifid leaves and eventually produced only simple leaves once stems became flowering. This developmental transition from pinnatifid juvenile to simple adult leaves appears to be relatively common in the Dulcamaroid clade (see *Solanum flaccidum*), but is often not noticed as herbarium specimens are usually made only from the terminal portions of reproductive stems. From herbarium specimens, it is apparent that leaf size decreases as the stem makes the transition to the inflorescence.

Leaf pubescence varies considerably in *Solanum dulcamaroides*, as it does in many other members of the group. Some specimens (especially those from the west of Mexico) are densely pubescent, with some of the trichomes furcate or with a few branches, but the majority of the trichomes are simple. Other plants, among them the type of *Solanum megalospermum* (*Williams et al. 41964*), are almost entirely glabrous. Plants from Nicaragua have smaller flowers (to 2.5 cm in diameter) than do plants from Mexico, flowering collections of *Solanum dulcamaroides* from Guatemala are a priority to see if this is a simple cline or due instead to environmental differences.

The epithet “dulcamaroides” has long been attributed to M.-F. Dunal rather than J.L.M. Poiret, the editor of the supplement to Lamarck’s encyclopaedia (see Nee 1982). While most of the 41 *Solanum* epithets published in the supplement are indeed attributable to Dunal, *Solanum dulcamaroides* has the diagnosis ascribed to Dunal, but the epithet was not, and was therefore probably coined by Poiret himself. Dunal later cites “*Solanum dulcamaroides* Poir.” in synonymy with his *Solanum macrantherum*, attributing the epithet “dulcamaroides” to Poiret only, thus indicating that Poiret did not use the epithet Dunal had wanted for this plant.

*Solanum dulcamaroides* (and its later homonym *Solanum macrantherum*) was described based on an illustration seen by Dunal in the collection of original drawings done for the Sessé and Mociño expedition to Mexico and Central America (see McVaugh 2000) and on his own, unpublished illustration, now held in the collections at MPU. Dunal is likely to have seen this illustration when Mocino was in Montpellier between 1812 and 1817 (see McVaugh 2000: 12); this set of original illustrations was then copied for Candolle, but no images of *Solanum* species are held in the set of copies at G. The original held in the Hunt Botanical Institute is thus the only material associated with the name. Material linked to this name was described by Sessé and Mociño as *Solanum sarmentosum* and *Solanum nutans*, and herbarium sheets linked with those names can be found in MA (see Knapp 2008b), duplicates of these are also held in G. An illustration in the Torner Collection of the Hunt Botanical Institute (accession number 6331.1503, Figure 40) that clearly shows the distinctive bud morphology of this species is here chosen as the lectotype of *Solanum dulcamaroides*. Epitype material is held in MA amongst the collections of the Sessé and Mociño expedition: I have selected as the epitype the sheet best matching the leaf morphology in the watercolour. Considerable confusion over the identities of *Solanum dulcamaroides* and the very similar Cuban endemic *Solanum boldoense* is apparent in the labelling of sheets at MA; this is discussed under *Solanum boldoense* above and in Knapp (2008b).

**Figure 40.**
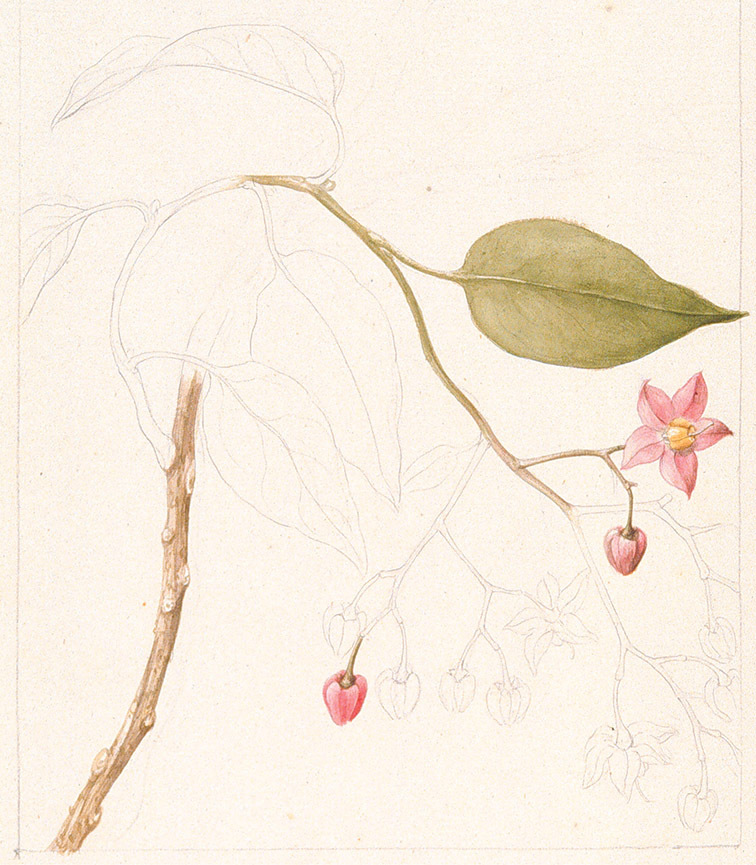
Lectotype of *Solanum dulcamaroides* Poir. (Torner Collection plate 6331.1503). Reproduced with permission of the Hunt Institute for Botanical Documentation, Carnegie Mellon University, Pittsburgh, PA. Torner Collection of Sessé and Mociño Biological Illustrations. Rights reserved.

##### Specimens examined.

**Guatemala**. **Baja Verapaz**: Reserva Municipal Los Cerritos, Baja Verapaz, Salamá. Cerro el Portezuelo, Reserva Municipal Los Cerritos, 1020 m, 10 Mar 2009, *Christenhusz et al. 5662* (BM); **El Quiché**: Sacapulas, 1600 m, 12 Jan 1974, *Molina R. et al. 30376* (F); **Huehuetenango**: Sierra de los Cuchumatanes, 15 Sep 1941, *Johnston 1978* (F).

**Mexico**. **Chiapas**: Ocozocoautla de Espinosa, 32 km N of Ocozocoautla along road to Mal Paso, 762 m, 19 Oct 1965, *Breedlove & Raven 13575* (F, MO, US); Motozintla de Mendoza, Huixtla, 45-50 km NE along road to Motozintla, 1900 m, 28 Dec 1972, *Breedlove & Thorne 31051* (MO); Siltepec, El Porvenir, 10 km NW, por camino a Siltepec, 2750 m, 17 Aug 1985, *Hernéndez & Ramírez H. 901* (MEXU); Chiapa de Corzo, El Chorreadero, 5.6 miles east of Chiapa de Corzo along Mexican Highway 190, 762 m, 15 Jul 1966, *Laughlin 1285* (US); Ocozocoautla de Espinosa, Chapapote to Viente Casas, 13 Nov 1964, *MacDougall 449s* (MEXU); Unión Juárez, en el camino Talquina-Cima de Volcán Tacana, rumbo a la linea divisoria con Guatemala, 1800 m, 20 Oct 1985, *Martínez S. 14166* (MEXU); bewteen Mazapa and Motozintla, 19 Jul 1941, *Matuda 4812* (F, GH, MEXU); Ocuilapa, 1036 m, 21 Aug 1895, *Nelson 3031* (GH, US); **Colima**: Comala, Rancho El Jabalí, 20 km (airline) N of Colima in the SW foothills of the Volcán de Colima, on main ranch road near the stables, 1400 m, 21 Sep 1991, *Sanders et al. 11609* (MEXU); Comala, Rancho El Jabalí, 22 km (ariline) N of Colima in the SW foothills of the Volcán de Colima, Colima/Jalisco line passes through ranch; 100 m from Lago El Epazote on the road from the ranch headquarters, 1400 m, 3 Aug 1991, *Vázquez V. 1001* (MO); **Guanajuato**: Cerro Capulín, western slopes, just E of Mexico 43, ca. 8 km NNE of Uriangato on road to Salamanca, 1900 m, 17 Sep 1977, *Iltis & Doebley 111* (F, MEXU); Puerto Nieto, 13 Aug 1947, *Kenoyer 2032* (GH); San Miguel de Allende, 28 Nov 1967, *Lape 1* (GH); Allende, Microondas Calderón, en el cerro de Alcoer, 2180 m, 12 Aug 1987, *Mora Benítez 827* (MEXU); Moroleón, Piñícuaro, 2120 m, 26 Jul 1986, *Zamudio 4162* (MEXU); **Guerrero**: Taxco, 14 Jun 1937, *Abbott 210* (GH); Taxco, 12 Aug 1937, *Abbott 358* (ECON); Mina, Manchón, 6 Jul 1937, *Hinton 10516* (GH, K, US); **Hidalgo**: Ajacuba, Ejido Santiago Texontlale, camino que va rumbo a la Mesa Chat, cerro al NW del poblado Santiago Textontlale, sierra de Mexe, 2440 m, 14 Sep 1988, *Díaz V. 207* (MEXU); Ajacuba, La Peñitas Blancas, NE del panteón de Emiliano Zapata, ladera S de la sierra Chicavasco, ejido San Nicolas Tecomatlán, 2260 m, 23 Aug 1988, *Díaz V. & Valverde G. 91207* (MEXU); Metzquititlán, Barranca de Venados, 1800 m, 6 Aug 1979, *Hernández M. 3610* (MEXU); Carretera Federal a Pachuca 3 km de la caseta, 9 Jul 1977, *Martínez Marino 44* (MEXU); Pueblo Viejo, 9 Jul 1977, *Martínez Marino 50* (MEXU); **Jalisco**: Autlán de Navarro, El Chante to Guisar, Sierra de Manantlán, 2160 m, 17 Aug 1980, *Breedlove & Almeda 45756* (MEXU); Zapopan, Arroyo Las Coronillas, 6 km al ONO de Nexitipac, sobre el cauce del arroyo, 1500 m, 9 Aug 2001, *Carrilo-Reyes 2479* (MEXU); Tuxpán, Atenquique, 1 km S por carr. a Colima, luego 9 km al O por brecha al Volcán de Colima, 1700 m, 23 Aug 1988, *Fuentes O. 546* (MO); Santa Cruz de Astillero, Santa Cruz de Astillero, 1500 m, 12 Aug 1984, *Hernández M. & Hernández V. 9537* (MEXU); Tonila, Cerro Alto, en al microondas, 2000 m, 12 Jul 1992, *Huerta M. et al. 230* (MEXU); 1.5 km S of Rincón de Manantlán, at very base of Sierra de Manantlán, exactly 13 km due S of El Chante, 1560 m, 9 Oct 1980, *Iltis & Guzmén M. 3164* (F, MEXU); Tepatitlán, Aug 1882, *Kerber* s.n. (A); Cihuatlán, adelante del aserradero, camino a La Cumbre, 1460 m, 15 Jun 1988, *López F. et al. 733* (MEXU); Atenquique, along logging road 5 km S of Atenquique, 1829 m, 4 Aug 1965, *Mertz 191* (MEXU); brecha Chalapa-Mezcala, 1590 m, 14 Jul 1974, *Puga 6739* (MEXU); Tapalpa, km 22 de la brecha Tapalpa-Ve. Carranza, 2100 m, 24 Aug 1986, *Rodríguez C. & Suárez J. 591* (MEXU); **Michoacán**: Morelia, N of Zapote, 4 Aug 1910, *Arsène s.n. [5970]* (K, L, US); Punguato, vicinity of Morelia, 2100 m, 16 Jul 1909, *Arsène 2894* (US); Lago de Pátzcuaro, 1 km al W de San Andres Tizorondaro, 2060 m, 27 Nov 1977, *Cabellero & Mapes 40* (MEXU); Cuitzeo, Jéruco, ca. 3 km al WNW, 1890 m, 14 Sep 1999, *Carranza & Silva 5824* (MEXU); Lagunillas, Cerro El Águila, 3 km del poblado de Huatzanguio, 2415 m, 15 Aug 2008, *Cornejo Tenorio & González Castañeda 2986* (MEXU); Jiquilpan, in and around a stone quarry on Route 110 between Km 25 and ca. 25.5 km W of Jiguilpan, 29 Aug 1966, *Cruden 1231* (GH); Erongarícuaro, Oponguio, 2100 m, 5 Jul 1990, *Díaz Barriga 6201* (MEXU); 2 km de Pomoca, carretera a Maravatio, 2100 m, 21 Mar 1991, *Díaz Barriga et al. 6854* (MA, MEXU); Quiroga, Santa Fe de Laguna, 2200 m, 23 Oct 1985, *Escobedo 465* (MEXU); Zitácuaro, Zitácuaro-Loma Larga, 15 May 1938, *Hinton 11857* (GH, K, US); Coalcomán, S. Naranjillo, 15 Jul 1939, *Hinton et al. 13951* (F, GH, US); Morelia, La Concepción, approx. 500 m al N, 2200 m, 10 Sep 1985, *Huerta B. 160* (MEXU); Ruta 15 Morelia a Zitácuaro, ca. 10 km al E de Morelia, 2100 m, 22 Oct 1981, *Lorence & Ramamoorthy 3742* (F, MEXU); Lake Cuitzeo, 9 Aug 1892, *Pringle 4188* (BM, F, G, GH, GOET, K, LE, MEX, US); Tlazazalca, Tlazazalca, camino a Cerro La Cruz, 1800 m, 5 Jul 1990, *Pérez & García 1344* (MEXU); Coeneo, 3 km al NE de Azajo, 2110 m, 14 Jul 1988, *Ramos 142* (MEXU); Susupato, La Ziranda, 1650 m, 26 Sep 1989, *Rzedowski 49012* (MEXU); Indaparapeo, 4 km al S, sobre el camino a Las Peras, 2100 m, 27 Sep 1989, *Rzedowski 49037* (MO); Huaniqueo, Tendeparacua, 3 km al S, 2150 m, 21 Oct 1990, *Rzedowski 50418* (MEXU); Alvaro Obregón, El Cerrito, cerca de Tzintzimeo, 1900 m, 21 Jul 1986, *Santos Martínez 1579* (MEXU); Huaniqueo, Coeperio, SE del pedregal grande, 1.4 km al NE de Coeperio, 2150 m, 14 Oct 1992, *Silva Saenz 406* (MEXU); 17 km al E de Morelia, en la desv. a Union Progreso, 1850 m, 24 Sep 1979, *Soto N. & Ramírez S. 1748* (MEXU); Tuxpán, La Providencia, 5.3 km al SO de Malacate, 1750 m, 1 Nov 1989, *Torres & Ramírez 13501* (MEXU); Contepec, El Tambor, aprox. 3 km al E de Tuxtepec, 2350 m, 25 Oct 1986, *Zamudio R. & Murillo 4971* (MEXU); **Morelos**: Tepoztlán, bajada en el camino del Parque a Texpotlán, 21 Jul 1940, *Miranda 507* (MEXU); Cuernavaca, 10 Oct 1840, *Moore* s.n. (K); Cerro Tepozteco, 20 km NO de Cuernavaca, 4 Jun 1987, *Torres C. & Miller 9690* (F, MEXU); Tepozteco, 1800 m, 1 Oct 1971, *Vázquez 3437* (MEXU); **México**: Temascaltepec, Real de Arriba, 1930 m, 4 Jun 1932, *Hinton 835* (K, MA); Temascaltepec, Cerro Muñeca, 2300 m, 15 Aug 1932, *Hinton 1360* (BM, F, G, K, MEXU, US); Temascaltepec, Temascaltepec, 1750 m, 18 Sep 1932, *Hinton 1697* (BM, G, GH, K, K); Ixtapán de la Sal, Valle de Mexico, 1800 m, 2 Sep 1951, *Matuda 20864* (MEXU); 3 km N of Ixtapan de la Sal along Highway 55, 1650 m, 28 Jul 1964, *Mick & Roe 344* (BM); Puerta del Gato, 7 km al NW de Zitácuaro, 1950 m, 20 Jul 1964, *Rzedowski 18351* (MEXU); Malinalco, carretera Malinalco-Tenancingo, poco despues de Malinalco, cima de cerros, 2180 m, 15 Jul 2001, *Vibrans 7366* (MEXU); **Oaxaca**: San Bartolo, Aug, *Andrieux 185* (G-DC, GH, K); Soyaltepec, Presa de Temazcal, a 2 km al N de los vertederos de la presa, 23 Sep 1984, *Cabrera & Torres 7255* (MEXU); San Lucas Ojitlán, del poblado de El Zapotal a Mata de Caña, 60 m, 23 Jan 1989, *Calzada 14291* (MEXU, MO); San Felipe Usila, Poblado Pas Escalera, senda para la cima del Cerro Paso Escalera, 450 m, 19 Oct 1989, *Calzada 14997* (MEXU); Suchixtepec, 19 km al S de San José Pacifico, carretera Miahuatlán-Pochutla, Dto. Miahuatlán, 2180 m, 1 Oct 1988, *Campos V. 2457* (MEXU); Presa Miguel Alemán, lado SO de la presa, 13 Feb 1982, *Cedillo Trigos & Torres 1031* (MEXU); Cerro San Antonio, 1550 m, 26 Jun 1906, *Conzatti 1410* (BH, GH, MEXU); San Miguel Soyaltepec, a 4 km de Temazcal camino a vertador, Dto. Tuxtepec, 60 m, 8 Mar 1986, *Cortés et al. 266* (MEXU); San Pedro Teutila, El Faro, 670 m, 19 Jan 2005, *Cruz Espinosa 2431* (MEXU); Tuxtepec, 21 miles S on Hwy. 175 to Oaxaca, 40 m, 30 Oct 1980, *Fryxell & Lott 3224* (BH, MEXU, MO); San Juan Mixtepec, Dto. Miahuatlán, 2050 m, 13 Jul 1997, *Hunn OAX-1493* (MEXU); Santa María Jacatepec, La Joya, Cerro Quemado, 500 m, 6 Nov 1986, *López Vargas 363* (MEXU); San Miguel del Puerto, Llano de Horno, limite de los terrenos comunales, Dto. Pochutla, 360 m, 20 Jul 2000, *López 340* (MEXU); Quiechapa, cercanias, 23 Oct 1953, *MacDougal* s.n. (MEXU); Chiltepec, and vicinity, Dto. Tuxtepec, 20 m, Jul 1940, *Martínez Calderón 333* (A, MEXU, US); San Miguel Soyaltepec, Cerro Tepezcuintle, Torre 38 de la L.T. Temascal II-Oaxaca Potencia, 203 m, 11 Nov 2004, *Martínez Feria & Juárez García 78* (MEXU); San Miguel del Puerto, 30 m de la desviación camino a Santa Catarina Janmixtepec, orilla Río Zimatán, Dto. Pochutla, 450 m, 29 Sep 2001, *Pascual 16* (MEXU); San Miguel del Puerto, 3.4 km al oeste del pueste San Lorenzo, sobre la brecha a Monte Carlo, Dto. Pochutla, 690 m, 25 Sep 2001, *Salas M. et al. 4001* (MEXU); San Juan del Estado, 18 Jun 1894, *Smith* s.n. (MEXU); San Pablo, Santa Cruz, 10-12 km al NW de Oaxaca, Distrito de Etla, 1680 m, 2 Nov 1978, *Solano & Vara 305* (MEXU, MO); Temascal, 13 Dec 1961, *Sousa 1093* (MEXU); Santa María Jacatepec, El Águila, al O de San Agustin, entrando por La Reforma, 28 km al SO de Tuxtepec, 550 m, 19 Jan 1988, *Torres C. & Martínez S. 11034* (MEXU); San Pedro Teutila, a 400 m de la brecha a El Faro, 1 km hacia el este de Faro, 571 m, 7 Oct 2002, *Velasco Gutiérrez & Juárez 83* (MEXU); Santa Cruz Itundujia, Dto. Putla, La Laguna a 2.88 km en LR (SW) de Santa Cruz Itundujia, 2181 m, 20 Jun 2008, *Velasco Gutiérrez et al. 2837* (MEXU); **Puebla**: Puebla, Jardín del Calvarío, 2175 m, 1 Sep 1907, *Arsène 2330* (GH, US); Morelia, environs, 2073 m, Aug 1840, *Galeotti 1183* (G); Tehuacán, 29 Jul 1897, *Pringle 7496* (BH, F, GH, GOET, K, L, US); El Riego, Jul 1905, *Purpus 1285* (F, GH); Cerro de Chicamole, Aug 1909, *Purpus 3969* (BM, F, GH, US); **Querétaro**: km 16-17 camino entre carretera a Mexico y Amealco, 2300 m, 2 Sep 1977, *Argüelles 802* (MEXU); Los Cues, 2200 m, 20 Jul 1981, *Argüelles 1666* (MEXU); Hacienda Ciervo, near Cadereyta, 26 Aug 1905, *Rose et al. 9846* (US); Amealco, Quiotillos, 2200 m, 18 Jul 1989, *Rzedowski 48594* (MEXU); **Veracruz**: Catemaco, ca. 3 km al E de Laguna Catemaco en el camino al Bastonal, 550 m, 11 Aug 1972, *Beaman 6436* (F); Orizaba, 10 Sep 1866, *Bourgeau 3054* (G, GH, K, L, LE, US); San Andrés Tuxtla, Laguna Encantada, 8 km N de San Andres Tuxtla, 300 m, 26 Jan 1978, *Calzada 4223* (F, MEXU); San Andrés Tuxtla, 2 km al norte, 20 Jan 1987, *Cedillo Trigos 3780* (MEXU); La Luz près Córdoba, 27 Sep 1882, *Kerber 68* (BM, G, GOET, K, LE, US); Catemaco, at highest point on road from Catemaco to Sontecomapán, 5 km N of junction with road around Laguna de Catemaco, 8 km (by air) NE of Catemaco, 510 m, 6 Dec 1981, *Nee 23735* (F, MO); Ixtaczoquiatlán, Orizaba, ca. 2 km N of town, south of old (non-cuota) road between Fortín and Orizaba, 1000 m, 7 Dec 1981, *Nee 23837* (BH, F, GH, MEXU, MO); Jilotepec, Vista Hermosa, 1200 m, 20 Oct 1977, *Ortega O. 726* (MEXU); San Andrés Tuxtla, Estación Biológica Las Tuxtlas, camino Corte Ruiz Cortinez, lote 71, 160 m, 3 Oct 1986, *Sinaca Colin 1016* (MEXU); Catemaco, Cerro de Buenavista, N de Catemaco, 490 m, 3 Oct 1998, *Torres R. & Campos V. 46* (MEXU); Jilotepec, Rincón del Muerto, 1400 m, 26 Apr 1971, *Ventura A. 3505* (F); La Concepción, 4 km de la desviacion carretera Concepción, 1050 m, 22 Jul 1976, *Zola B. 552* (F, MEXU).

**Nicaragua**. **Estelí**: Mesas Moropotente, ca 16.0 km (by road) NE of Hwy. 1 at Estelí, 1310 m, 11 Jun 1981, *Henrich & Stevens 454* (BM); Estelí, km 144.5, Fundacion CECALII, 782 m, 21 Jun 2001, *Rueda et al. 16497* (MO).

#### 16. Solanum endoadenium

Bitter, Repert. Spec. Nov. Regni Veg. 12: 546. 1913

http://species-id.net/wiki/Solanum_endoadenium

Figure 41

Solanum endoadenium Bitter var. *robustius* Bitter, Repert. Spec. Nov. Regni Veg. 12: 547. 1913. Type: Argentina. Catamarca: Cuesta de Muschaca, *F. Schickendantz 285* (holotype: B, destroyed; lectotype, designated here: CORD [CORD00004206]).
Solanum incurvipilum Bitter, Repert. Spec. Nov. Regni Veg. 12: 158. 1913. Type: Argentina. Salta: El Paso “Al Alizar” [Alizal?], 2400-2600 m, between Pampa Grande, 1740 m and Cerro Cristal, 2610-1700 m, *E. Nelson in F. Kurtz 12512* (holotype: S [S04-2919]; isotype: CORD [CORD00004229]).

##### Type.

Argentina. La Rioja: Pie de Cuesta, above Vallecito, Sierra Famatina, *G.H.E.W. Hieronymus & G. Niederlein 746* (lectotype, designated by Morton 1976, pg. 88: G [G00070235]; isolectotypes: CORD [CORD00004199, CORD00004200], LE, P [P00335096]).

##### Description.

Shrubs 1–1.5 m tall. Stems erect, sparsely to densely pubescent with patent simple uniseriate trichomes ca. 0.5 mm long, often gland-tipped and the plants viscous; new growth densely pubescent. Bark of older stems yellowish brown, the leaf bases prominent. Sympodial units plurifoliate. Leaves simple, 1.8–6 cm long, 0.5–2 cm wide, lanceolate to more or less linear, slightly fleshy, the upper surfaces uniformly pubescent with sparse to dense simple uniseriate trichomes ca. 0.5 mm long, sometimes gland-tipped, the lower surfaces variable from almost glabrous with the simple trichomes confined to the veins to densely and uniformly pubescent with simple uniseriate trichomes ca. 0.5 mm long; primary veins 6–9 pairs, drying yellowish red; base attenuate; margins entire, densely pubescent; apex acute to acuminate, the ultimate tip rounded; petioles 0.5–1 cm long, sparsely to densely pubescent with simple trichomes like those of the stems, usually not twining. Inflorescences terminal or becoming lateral, 2–4 cm long, simple to several times branched, with 5–30 flowers, glabrous to densely pubescent with simple uniseriate trichomes like the stems; peduncle 1–3 cm long; pedicels 0.6–0.8 cm long, filiform, < 0.5 mm in diameter at the base and apex, erect to somewhat spreading, glabrous to pubescent with simple uniseriate trichomes, articulated at the base from a small sleeve, leaving a small peg on the inflorescence axis; pedicel scars irregularly spaced 1–4 mm apart. Buds globose to slightly ellipsoid, the corolla exserted from the calyx tube before anthesis. Flowers all perfect, 5-merous. Calyx tube 1–1.5 mm long, conical, the lobes 1–1.5 mm long, triangular, sparsely to densely pubescent with simple uniseriate trichomes. Corolla 0.7–1 cm in diameter, dark bluish purple to pale violet with green spots at the base of the lobes, stellate, lobed 1/2 to 2/3 of the way to the base, the lobes 3–3.5 mm long, 2–2.5 mm wide, spreading or perhaps cupped, densely pubescent all along the tips and margins with simple trichomes < 0.2 mm long. Filament tube minute, the free portion of the filaments ca. 0.5 mm long, glabrous or minutely pubescent with glandular trichomes; anthers 1.5–2 mm long, ca. 1 mm wide, ellipsoid, loosely connivent, poricidal at the tips, the pores lengthening to slits with age. Ovary glabrous; style 4–5 mm long, glabrous or occasionally glandular in the lower half; stigma minutely capitate, the surface minutely papillose. Fruit a globose berry, 0.6–0.8 cm in diameter, orange when ripe, the pericarp thin and shiny, glabrous; fruiting pedicels 1.2–2 cm long, ca. 1 mm in diameter at the base, not markedly woody, nodding or spreading. Seeds 12–14 per berry, 3.5–4 mm long, 3–3.5 mm wide, flattened reniform, reddish brown, the surfaces minutely pitted, the testal cells very small, rectangular to square. Chromosome number: n=12 (Moscone 1992).

**Figure 41.**
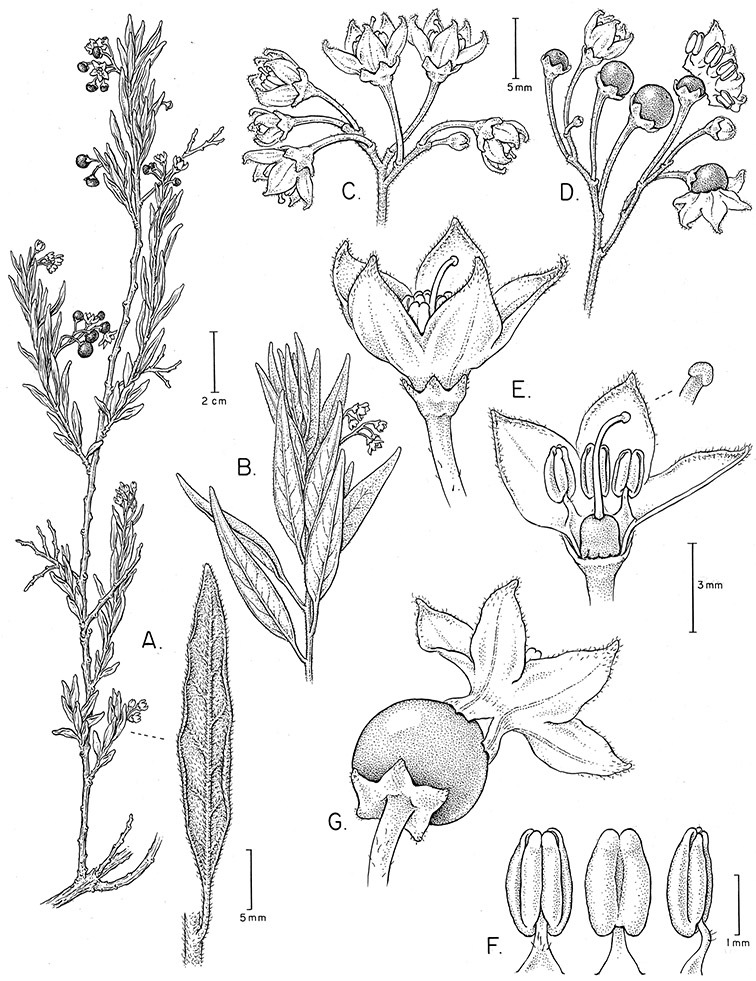
*Solanum endoadenium* Bitter. (**A, C–G** drawn from *Hunziker et al. 25550* **B** *Legname & Vervoorst 154*). Illustration by Bobbi Angell.

##### Distribution

**Figure 42.**
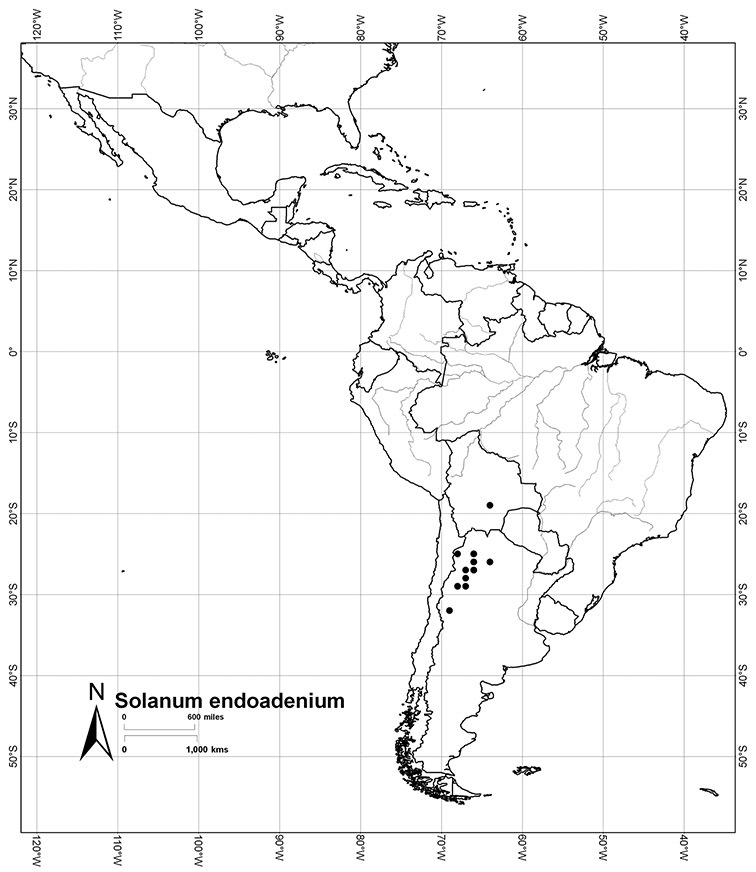
Distribution of *Solanum endoadenium* Bitter.

(Figure 42). Eastern Andean slopes in central to northern Argentina and adjacent Bolivia, from 1500–3000 m.

##### Ecology.

Forests and open areas above treeline; sometimes growing in sandy soil in puna vegetation.

##### Conservation status.

Least Concern (LC); EOO >100,000 km^2^ (LC) and AOO >10,000 km^2^ (LC). See Moat (2007) for explanation of measurements.

##### Discussion.

Unlike many of the members of the Dulcamaroid clade, *Solanum endoadenium* is a shrubby species and is apparently never a vine or even scandent. The leaves are consistently simple, and apparently never lobed or pinnatifid. *Solanum endoadenium* is easily recognised by its purple flowers, orange fruits and dense, often glandular-viscid pubescence of simple trichomes. The older stems are warty from the persistent leaf bases; this distinctive character distinguishes *Solanum endoadenium* from the somewhat similar *Solanum salicifolium* from the same region. *Solanum endoadenium* is further easily distinguished from *Solanum salicifolium* by its larger, usually branched inflorescences and more spreading pubescence (rather than appressed-ascending).

Leaf size in *Solanum endoadenium* is quite variable, and varies within as well as between individuals. In general leaves near the stem tips are smaller than those lower down. Bitter (1913) stated that the epithet was derived from the glandular pubescence on the inside of the filaments. The berry of *Solanum endoadenium* stays on the plant a long time, and apparently cracks to release the seeds (see Figure 6D); whether this is always the case is not clear.

Morton (1976) lectotypified *Solanum endoadenium* with the only syntype (of seven in Bitter’s original description) from La Rioja province, as Bitter (1913) stated he was basing his description on material from La Rioja. In his lectotypification, Morton cites the collection as *Hieronymus & Neiderlein 746*, but the only specimen at G [G00070235] has no collection number, nor do the duplicates at LE and P. Morton’s annotation slip on this G sheet states “isosyntypus” and is dated 1961; he may have made a transcription error later, as this is the only specimen at G that bears this locality and collector information. I have therefore assumed he meant this sheet in his lectotypification and have accepted his designation of the G sheet as the lectotype. I have found no duplicates of *Schickendantz 285* (the type collection cited in the protologue of var. *robustius*) in any of the herbaria where other Schickendantz duplicates have been found (GOET, SI); Bitter cited “herb. Hieronymous” in the original description (probably at B and now destroyed).

##### Specimens examined.

**Argentina**. **Catamarca**: Belén, al incio de la Cuesta de Randolfo, 3009 m, 31 Jan 2008, *Barboza et al. 1997* (CORD); Belén, próximo a Quebrada de Randolfo rumbo a Nacimientos de San Antonio, 12 Feb 2012, *Barboza et al. 3476* (BM, CORD); Pomán, Sierra de Ambato, falda oeste, Mutquin, en Valle Muerto, 4 Feb 1910, *Castillón 1613* (CORD); Andalgalá, Quebrada del Río Pisavil, 26 Nov 1948, *Filipovich 66* (CORD); Ambato, Cumbres de Narváez, falda oeste, Ruta 62, km inmediaciones de Las Chacritas, entre Narváez y Singuil, 1850 m, 10 Dec 1965, *Hunziker et al. 18555* (CORD); Ambato, Sierra de Ambato, falda E, subiendo desde El Rodeo hacia el Cerro Manchado, 2300 m, 23 Feb 1967, *Hunziker 19075* (CORD); Pomán, Sierra de Ambato, falda oeste, subiendo desde El Rincón hacia Las Casitas, rumbo al Cerro Manchado, 2300 m, 18 Feb 1970, *Hunziker & Ariza 20349* (CORD); Ambato, Cumbres de Narváez, falda oeste, bajando hacia el SE por Ruta 62, entre las Narváez y Las Chacritas, 2000 m, 13 Feb 1986, *Hunziker et al. 24909* (CORD); Andalgalá, Nu Mara del Condado, 700 m, Feb 1916, *Jörgensen 1310* (A, GH, SI, US); Santa María, Cerrillos, Sierra de Arconquija, 3000 m, 16 Dec 1933, *Peirano* s.n. (GH); Santa María, Estancia Totoral, 24 Feb 1948, *Reales 1035* (B); Santa María, Los Pabellones, 16 Mar 1949, *Reales 1931* (BH); El Ingenio, 2700 m, 5 Dec 1960, *Roig 3664* (CORD); Belén, Pozo de Piedra, en Puerta de San José, Jan 1955, *Sayago 1944* (CORD); Belén, Belén, Yacutula cerca Bélen, Dec 1879, *Schickendantz 120* (CORD); Belén, Las Mansas, 2300 m, Mar 1938, *Schreiter 10469* (GH); Andalgalá, El Suncho, Río Pisavil, 1900 m, 21 Feb 1951, *Sleumer 1623* (G); **Córdoba**: Quebrada de Choya y Cuesta de Munchaca, Nov 1872, *Schickendantz 30* (CORD, SI, US); **La Rioja**: Famatina, Las Cuevas de Noroña, 2700 m, 20 Feb 2003, *Barboza et al. 578* (CORD); Famatina, Los Cajoncitos, unos pocos km antes viniendo desde Cuevas de Pérez, 2954 m, 2 Feb 2011, *Barboza et al. 2760* (CORD); Famatina, Cuevas de Noroña, en el corral, 2847 m, 2 Feb 2011, *Barboza et al. 2786* (CORD); Felipe Varela, Sierra de Sañogasta, falda oeste, subiendo desde Aicuña hacia el ESE, rumbo al cerro homómino, 2300 m, 17 Dec 1975, *Hunziker & Hunziker 22850* (CORD); Famatina, Esquina Gervasio, Sierra de Famatina, 3000 m, 14 Jan 1949, *Krapovickas & Hunziker 5268* (CORD); Arroyo Salado, Pampas, Chilitanco-Acharil, 10 Mar 1907, *Kurtz 10542* (CORD); Famatina, Río Amarillo, Las Cuevas, 29 Mar 1906, *Kurtz 14056* (CORD); entre Pampas Chilitanco y Achavil, 10 Mar 1907, *Kurtz 14542* (CORD); Famatina, La Hoyada, 11 Jan 1908, *Kurtz 14975* (CORD); Yacuchi, Sierra Velasco, 25 Feb 1908, *Kurtz 15381* (CORD); **Salta**: Chicoana, Cuesta del Obispo, Quebrada de Lapacheta, 2880 m, 19 Mar 1972, *Krapovickas et al. 22056* (MO); Chicoana, Ruta 33 Chicoana-Cachi, 25 km W of the bridge at Aguas Negras (= 48 km W of El Carril, = 4 km above San Martin), 2600 m, 26 Nov 2003, *Leuenberger & Eggli 4865* (B); below Piedra del Molino, Cachi – Salta, below Piedra del Molino, before Escoipe, 2000 m, 10 Nov 1978, *Renvoize 3421* (K); **San Juan**: Zonda, camino a Estancia Maradona, Maradona a Agua del Pinto, 1400 m, *Kiesling 3321* (CORD, MO); **Santiago del Estero**: Pellegrini, Algarrobal Viejo, 28 Apr 1947, *Luna 97* (B); **Tucumán**: Tafí, a 28 km de Tafi rumbo a Amaicha del Valle, 2857 m, 21 Feb 2011, *Barboza et al. 3018* (CORD); Tafí, desde Amaicha del Valle rumbo a Tafí del Valle, entre km 92-91, 13 Feb 2012, *Barboza et al. 3490* (BM, CORD); Tafí, El Molle, en el camino de Tafí del Valle a Amaicha, km 91-93, 2800 m, 12 Dec 1986, *Hunziker et al. 24875* (CORD); Quebrada de Amaicha, boca de la quebrada, 2200 m, 13 Mar 1927, *Schreiter 4826* (GH); Tafí, Quebrada de Amaicha, 2800 m, 22 Nov 1927, *Venturi 5498* (A, GH, MA, SI, US); Tafí, Colalao del Valle, 1800 m, Apr 1926, *Venturi 6671* (GH, US).

**Bolivia**. **Santa Cruz**: Vallegrande, 13 km (air) NE of Pucará, on road from Vallegrande to Pucará, 14.1 km (by road) and 8 km (air) SW of the road junction on the north side of Guadalupe, 2850 m, 14 Nov 2009, *Nee & Linneo F. 56757* (USZ).

#### 17. Solanum flaccidum

Vell., Fl. Flumin. 87. 1829

http://species-id.net/wiki/Solanum_flaccidum

Figure 43

Solanum triphyllum Vell., Fl. Flumin. 88. 1829. Type: Brazil. Rio de Janeiro: Boavista, *Anon*. (no specimens cited; lectotype, designated here: Vellozo, Fl. Flumin. Icones 2, tab. 120. 1831).
Solanum convolvulus Sendtn. in Mart., Fl. Bras. 10: 48. 1846. Type: Brazil. Southern Brazil, *F. Sellow* [46] (lectotype, designated here: P [P00335275]; isolectotypes: C [F neg. 22880], F fragment [F-617553], LE, M [Morton neg. 8689], P [Morton neg. 8165], W [W-1889/0294607, W-0003282]).
Solanum fultum Schrank ex Sendtn. in Mart., Fl. Bras. 10: 49. 1846. Type: Brazil. Minas Gerais: São João Batista, Sept, *C. Martius* [1071] (lectotype, designated here: M [M0171876, F neg. 6531]).
Solanum delilei Dunal, Prodr. [A.P. de Candolle] 13(1): 81. 1852. Type: France: Cultivated in Paris from material said to have come from Mexico, *Anon.* s.n. (lectotype, designated here: G [G00357858, F neg 23109]).
Solanum convolvulus Sendtn. var. *pilosum* Dunal, Prodr. [A.P. de Candolle] 13(1): 83. 1852. Type: Brazil. No locality or specimens cited, only reference is to Sendtner’s original description.
Solanum uncinellum Lindl. var. *atrosanguineum* Dunal, Prodr. [A.P. de Candolle] 13(1): 76. 1852. Type: Brazil. São Paulo: Sin. loc., *C. Gaudichaud 312* (holotype: P [P00335286, Morton neg. 8358]).
Solanum convolvulus Sendtn. var. *heterophyllum* Witasek, Kaiserl. Akad. Wiss. Wien, Math.-Naturwiss. Kl., Denkschr. 79: 328. 1910. Type: Brazil. São Paulo: Sorocaba, 1902, *M. Wacket* s.n. (lectotype, designated here: W [W-1922-0001503]).
Solanum flaccidum Vell. var. *heterophyllum* Witasek, Kaiserl. Akad. Wiss. Wien, Math.-Naturwiss. Kl., Denkschr. 79: 329. 1910. Type: Brazil. Paraná: Rio Cachoeira, Antonina, 1904, *M. Wacket* s.n. (lectotype, designated here: WU [WU0037973]; isolectotype: WU [WU0037974]).

##### Type:

Brazil. Rio de Janeiro: Sin. loc., *Anon*. (no specimens cited; lectotype, designated here: Vellozo, Fl. Flumin. Icones 2, tab. 115. 1831).

##### Description.

Woody vine, twining by petioles. Stems glabrous to densely white pubescent with simple uniseriate trichomes 0.25–0.5 mm long; new growth densely white pubescent with simple uniseriate trichomes ca. 0.5 mm long. Bark of older stems reddish brown. Sympodial units plurifoliate, not geminate. Leaves simple or more rarely pinnatifid with 1–3 lobes, 2.5–9 cm long, 1–4 cm wide, ovate or obovate to triangular or elliptic, membranous, the upper surfaces glabrous to sparsely pubescent with simple uniseriate trichomes, these always present on the midrib, the lower surfaces pubescent with simple uniseriate trichomes ca. 0.5 mm long, these in tufts in the vein axils to densely distributed over the entire lamina; primary veins 7–9 pairs, the midrib pubescent adaxially; base truncate to slightly cordate, often asymmetric; margins entire to lobed, the lobes to 1 cm long, pinched in at the base; apex acute; petioles 1–5 cm long, glabrous to densely white-pubescent, curling and twining. Inflorescences terminal or very occasionally lateral, 1–9 cm long, many times branched, with 10–50 flowers, glabrous to densely pubescent with simple uniseriate trichomes ca. 0.5 mm long, the transition from stem to inflorescence not always clear; peduncle ca. 1 cm long; pedicels 1–1.5 cm long, slender, ca. 0.5 mm in diameter at the base, 0.5–1 mm in diameter at the apex, spreading at anthesis, glabrous to sparsely pubescent with simple trichomes, articulated at the base from a small sleeve; pedicel scars irregularly spaced 0.5–1 cm apart. Buds ellipsoid to fusiform, the corolla strongly exserted from the calyx tube before anthesis. Flowers all perfect, 5-merous. Calyx tube 2–2.5 mm long, conical, the lobes 1–1.5 mm long, broadly deltate, glabrous or sparsely pubescent. Corolla 2.3–2.5 cm in diameter, violet or purple, fading to white, rotate-stellate, lobed ca. 1/2 way to the base, the lobes 6–9 mm long, 5–8 mm wide, spreading or planar at anthesis, densely pubescent abaxially with simple uniseriate trichomes < 0.25 mm long, glabrous adaxially. Filament tube minute, free portion of filaments unequal, one filament 3–3.5 mm long, the others 2–2.5 mm long, the long filament equalling the others early in anthesis, lengthening with flower age, all filaments glabrous; anthers 4.5–5 mm long, 1–1.5 mm wide, ellipsoid, loosely connivent, yellow, poricidal at the tips, the pores lengthening to slits with age or not. Ovary glabrous; style 8–10 mm long, glabrous; stigma clavate, the surface minutely papillose. Fruit a globose berry, 0.9–1 cm in diameter, green (immature?), probably shiny at maturity, glabrous the pericarp thin; fruiting pedicels 2–2.5 cm long, ca. 1 mm in diameter at the base (immature?), woody, pendent. Seeds not observed from mature berries. Chromosome number: not known.

**Figure 43.**
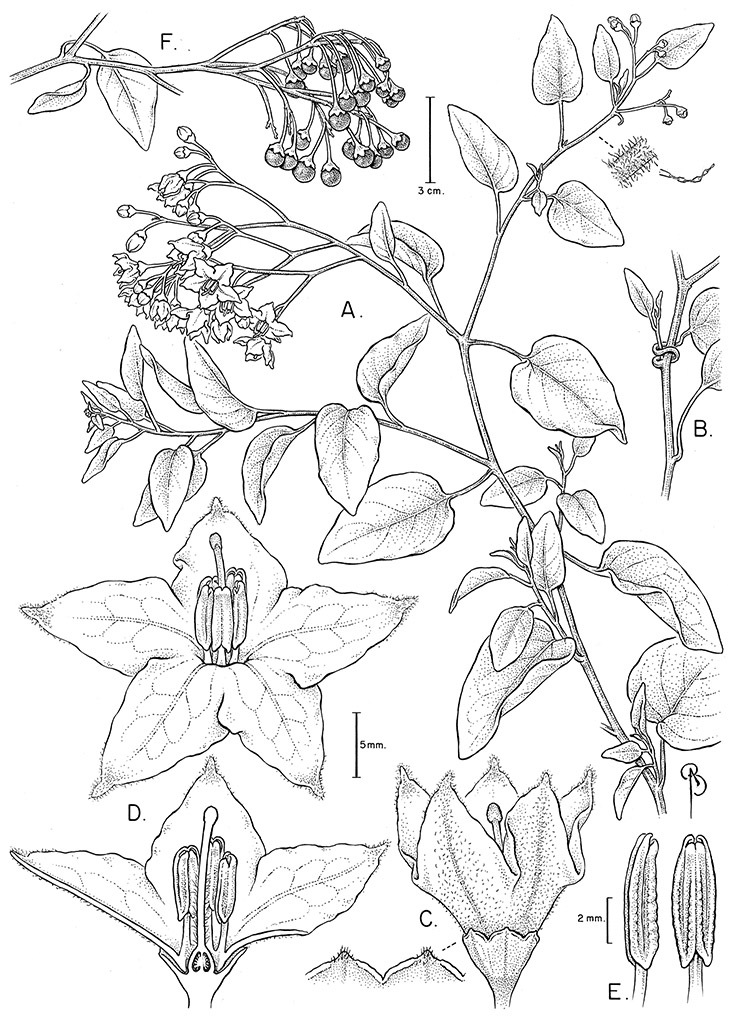
*Solanum flaccidum* Vell. (**A–D** drawn from *Nee & Bohs 50809* **E** drawn from *Nee & Bohs 50768*). Illustration by Bobbi Angell.

##### Distribution

**Figure 44.**
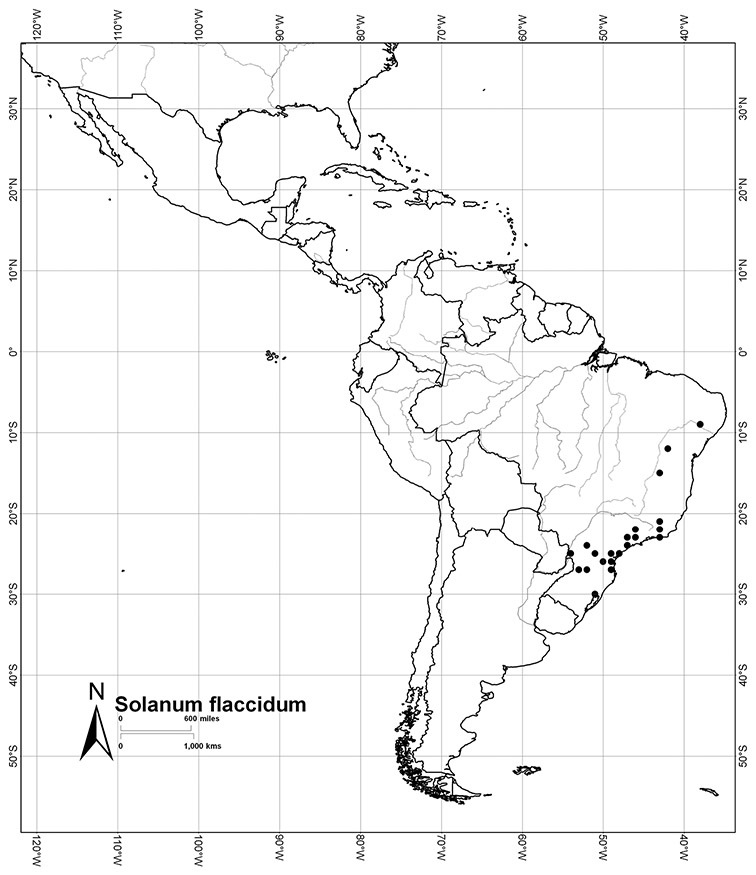
Distribution of *Solanum flaccidum* Vell.

(Figure 44). Coastal eastern Brazil inland to Paraguay; 40–1800 m.

##### Ecology.

In forests and along forest margins and riversides.

##### Common names.

Brazil. Santa Catarina: joá-cipó (Smith and Downs 1966).

##### Conservation status.

Least Concern (LC); EOO >100,000 km^2^ (LC) and AOO >10,000 km^2^ (LC). See Moat (2007) for explanation of measurements.

##### Discussion.

*Solanum flaccidum* is a relatively common species where it occurs, and in Brazil it is found sympatrically with *Solanum odoriferum*, also a vining member of the Dulcamaroid clade. The two species can be distinguished by their leaf morphology (glabrous and shining in *Solanum odoriferum*, variously pubescent in *Solanum flaccidum*), calyx shape (truncate in *Solanum odoriferum*, lobed in *Solanum flaccidum*) and anther morphology. *Solanum flaccidum* has one anther with the filament longer than the other four, while in *Solanum odoriferum*, the filaments are all equal throughout anthesis. In early anthesis in *Solanum flaccidum*, the filaments appear to be more or less equal, so care must be taken to examine fully mature flowers.

In Paraguay *Solanum flaccidum* is sympatric with *Solanum uncinellum*, which also has one anther borne on a longer filament. *Solanum flaccidum* has more rotate flowers than the latter species, and in general is less pubescent with simple, rather than dendritic, trichomes. The long filament in flowers of *Solanum uncinellum* is twice as long as the other filaments, while in *Solanum flaccidum* the long filament is less than twice as long as the others; in addition, the anthers of *Solanum uncinellum* are tapered and pointed, while those of *Solanum flaccidum* are ellipsoid.

As with many species in this group, *Solanum flaccidum* has heteromorphic leaves, with simple and pinnatifid leaves borne on the same stems. Once a stem is flowering, it appears that the leaves are all simple, so very few collections have been made of plants with pinnatifid leaves (but see *Mueller 311* at K). It may be, as with other species of this clade, that juvenile foliage is more likely to be pinnatifid than is foliage on reproductive shoots (see *Solanum dulcamaroides*).

**Figure 45.**
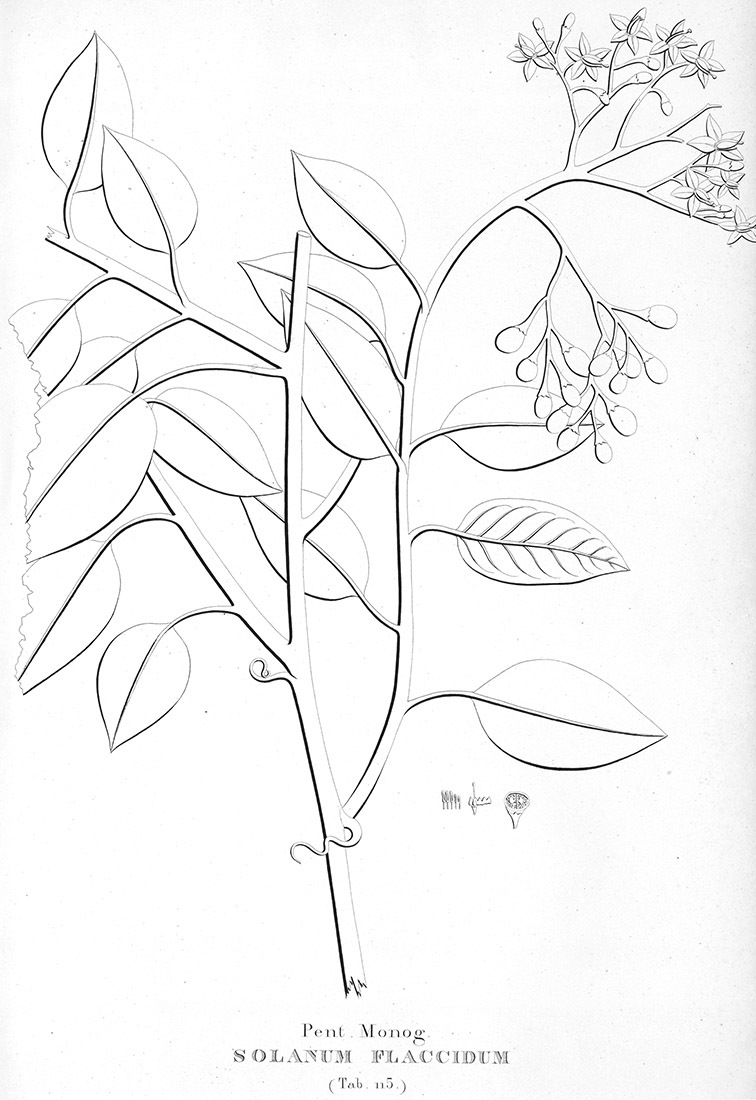
Lectotype of *Solanum flaccidum* Vell. (Vellozo 1831: tab. 115). Reproduced with permission of the Natural History Museum Botany Library.

**Figure 46.**
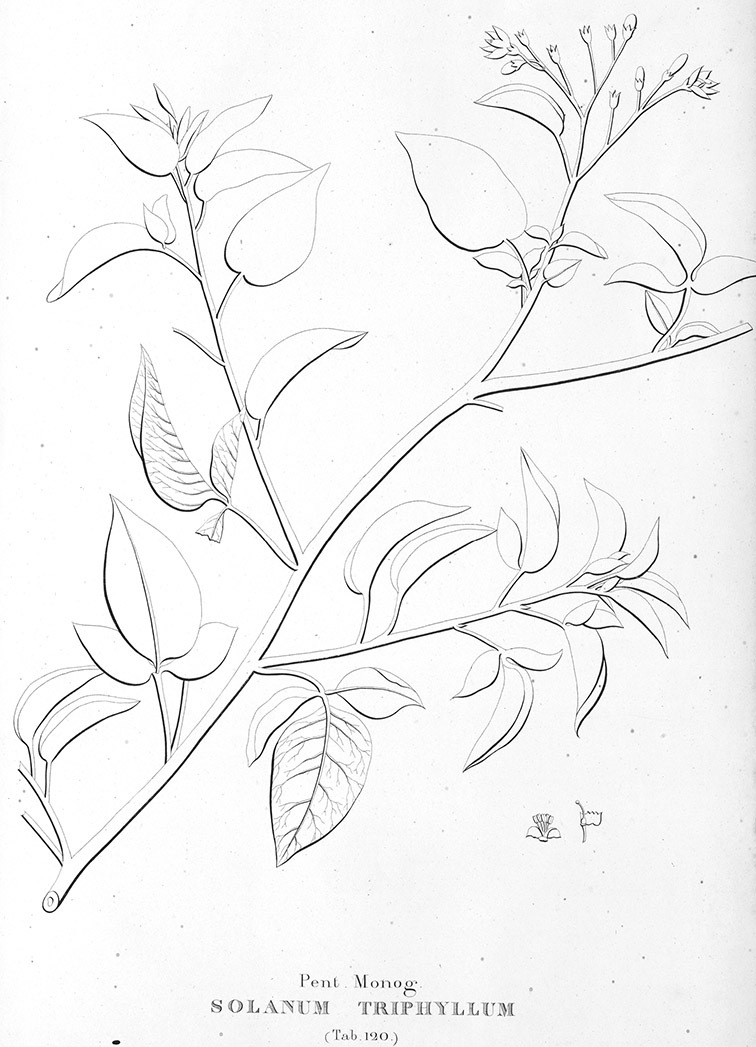
Lectotype of *Solanum triphyllum* Vell. (Vellozo 1831: tab. 120). Reproduced with permission of the Natural History Museum Botany Library.

In the absence of specimens (Carauta 1973), lectypification of Vellozo names is best done using the plates from that work (Vellozo 1831); they are distinctive and in this case have all the distinguishing features of the species (i.e., twining petioles and unequal anthers, see Figure 45)). The name *Solanum flaccidum* has been in more common use (e.g., Mentz and Oliveria 2004) and is therefore to be preferred over the simulataneously published *Solanum triphyllum* (Figure 46). *Solanum convolvulus* is lectotypifed with one of the many sheets of the syntypes that is annotated in Sendtner’s hand.

Although the type of *Solanum delilei* was said to have been grown from Mexican seeds and the description does not mention unequal anthers, the specimen at G has the unmistakable anther morphology and is vegetatively a perfect match for *Solanum flaccidum*. Dunal cited material he had seen cultivated in Paris and Montpellier, but the only herbarium material unambiguously cited is a sheet from “h. Delessert”, here designated as the lectotype. Mix-ups as to the provenance of plants grown in European botanic gardens were common (see Turland et al. 1996).

Several sheets of *Lhotsky 87* in G-DC have labels stating “Solanum uvareaifolium nob. 1819” in Dunal’s hand; this name was never published. Several Martius collections (Martius “745” and “747” from Minas Gerais and Bahia and one Sellow collection (from southern Brazil) are syntypes of *Solanum fultum*; I have chosen the sheet at M (M0171876) of *Martius 745* bearing a label with a description in Sendtner’s hand and with several inflorescences bearing flowers with the diagnostic unequal anthers as the lectotype. The other two syntypes are also from Minas Gerais, but are less complete (*Martius 747*, M0171877; *Sellow* s.n., P00335352).

Witasek (1910) described variety *heterophyllum* under both *Solanum convolvulus* and *Solanum flaccidum*; a sheet of the former at W (W-1922-0001503) is selected as the lectotype (no herbaria were cited in the protologue and these specimens are at either W or WU) and the sheet at WU [WU0037973] with pinnatifid lower leaves as the lectotype of the latter.

The type specimen of *Solanum uncinellum* var. *atrosanguineum* at P has the ellipsoid anthers and rotate-stellate corolla of *Solanum flaccidum*, not the tapered anthers and deeply stellate corolla of the more widespread *Solanum uncinellum*.

##### Specimens examined.

**Brazil**. **Bahia**: Conquista, 1000 m, Jun 1944, *Schury 616* (US); **Espírito Santo**: Itaguaçu, Alto Limoeiro, 10 May 1946, *Brade et al. 18054* (K); Muniz Freire, Rod. BR-262, 1000 m, 21 Jul 1982, *Hatschbach & Guimarães 45169* (US); **Minas Gerais**: Viçosa, Araponga, beyond Areponga, Fazenda do Serra, 1300 m, 3 May 1930, *Mexia 4660* (BM, F, G, GH, K, MO, S, US); Ilhéu, Fazenda da Tabunha, 300 m, 17 Aug 1930, *Mexia 4692* (BH, BM, F, G, GH, K, MO, S, US); Caldas, Rio Capivahy, 25 Nov 1873, *Mosen 983* (S); Caldas, 26 Jul 1869, *Regnell II-1712* (S, US); **Paraná**: Araucária, 13 Nov 1972, *Dombrowski 4246* (US); Roça Nova, 15 Mar 1909, *Dusén* s.n. (F, K, S); Serrinha, Rio Iguassu, 12 Dec 1908, *Dusén 7469* (S); Porto de Cima, 28 Sep 1909, *Dusén 8764* (S); Serra do Mar, desvio Ypiranga, 6 Sep 1911, *Dusén 12147* (S); Piraquara, estrada entre Rio Taquarí e Rio Divisa, 13 Mar 1949, *Hatschbach 1217* (US); Paranaguá, Sertão, 26 Aug 1951, *Hatschbach 2685* (US); Guarapuava, Serra da Esperança, 1135 m, 20 Oct 1960, *Hatschbach 7329* (US); Paranaguá, Encruzilhada, 18 May 1961, *Hatschbach 8026* (US); Porto Amazonas, Fazenda São Luiz, margens do Rio Iguacu, 13 Oct 1963, *Hatschbach 10892* (B, US); Antonina, Rio Cotia, 360 m, 17 Sep 1965, *Hatschbach 12809* (US); Antonina, Cab. Rio Faisqueira, 50 m, 11 Apr 1968, *Hatschbach 19019* (F, L); Campina Grande do Sul, Rio Bonito, rod. Br. 116, 31 Oct 1969, *Hatschbach 22756* (F, S); Jaguariaíva, Rib. Taquaral, 12 Nov 1981, *Hatschbach 44362* (MA); Serrinha, 840 m, 8 Oct 1914, *Jönsson 1084 a* (F, K, S); Pinhães, 30 Oct 1908, *Lange 6972* (S); Pinhães, 16 Nov 1960, *Moreira 36* (US); Curitiba, Parque Iguaçu, mini-pantanal, 12 Nov 1987, *Silva & Cordeiro 399* (S); Curitiba, Campina do Siqueira, Rio Barigui, 20 Nov 1966, *Stellfeld 1644* (US); **Pernambuco**: Floresta, Inajá, Reserva Biológica de Serra Negra, 21 Jul 1995, *Ferraz et al. 273* (BM); Floresta, Inajá, Reserva Biológica de Serra Negra, 21 Jul 1995, *Silva et al. 85* (BM); **Rio Grande do Sul**: Caixas do Sul, São Virgilio, 3a Legua, 780 m, 29 Nov 1999, *Kegler 390* (US); Bom Jesus, Passo da Guarda, 14 Jan 1952, *Rambo 51858* (B); São Francisco de Paula, Iaimbezinho, 20 Feb 1953, *Rambo 53995* (B); Itaimbezinho, pr. S. Fr. de Paula, 12 Feb 1956, *Rambo 58557* (B, S); San Salvador, Montenegro, 600 m, 9 Oct 1946, *Sehnem 2196* (B); **Rio de Janeiro**: Teresópolis, 2 May 1917, *Asampaio 2425* (US); Organ Mts., Feb 1837, *Gardner 558* (K); province of Rio de Janeiro, 1876, *Glaziou 8855* (G, K, LE); environs de Rio de Janeiro, *Glaziou 12100* (K, LE); Serra das Órgãos, Sep 1832, *Lhotsky 87* (G-DC); Organ Mount. Vargem, Jan 1838, *Miers* s.n. (BM); Organ Mts., *Miers 3465* (K); prope Engo, Pasos, km 151 viae BR-2, 23 Oct 1962, *Pabst 7146* (F, US); estrada para Friburgo, 4 Oct 1959, *Pereira 5116* (US); Macaé, 1900, *Ule* s.n. (US); Serra das Órgãos, Dec 1897, *Ule 4302* (CORD); **Santa Catarina**: Itajaí, 5 km W of Itajaí, 100 m, 6 Jan 1974, *Conrad & Dietrich 2124* (MO); Itajaí, Cunhas, 10 m, 27 Oct 1955, *Klein 1731* (B, G, L); Pantano do Sul, Morro do Saquinho, 27 Jul 1967, *Klein & Bresolin 7525* (US); Tapera, Ribeirão, 250 m, 18 Aug 1970, *Klein 8719* (US); Lajes, Encruzilhada, 900 m, 16 Dec 1967, *Lourteig 2286* (US); Ibirama, Horto Florestal I.N.P, 350 m, 2 Nov 1953, *Reitz & Klein 1168* (US); Campo dos Padres, 1800 m, 20 Dec 1948, *Reitz 2571* (US); Brusque, 40 m, 3 Nov 1949, *Reitz 3162* (S); São Joaquim, Bom Jardim, fazenda da Laranja, 1400 m, 13 Dec 1958, *Reitz & Klein 4082* (B, G, K, L, US); Rio do Sul, Serra do Matador, 700 m, 12 Mar 1959, *Reitz & Klein 8514* (K, US); Itajaí, Cordeiros, 5 m, 9 Oct 1959 *Reitz & Klein 9157* (US); São José, Serra da Boa Vista, 1000 m, 24 Jan 1961, *Reitz & Klein 10719* (K); Ipumirim, 19 km S of the Rio Irani, 9 Dec 1964, *Smith & Klein 13922* (US); **São Paulo**: Santa Ana, Nov 1912, *Brade, A.C*., *6031* (US); Jundiahy, Mar 1900, *Campos Novaes 167* (US); Capoeira Grande, Sep 1898, *Campos Novaes 4089* (US); Atibaia, Sep 1910, *Duarte* s.n. (US); sin .loc, 1833, *Gaudichaud 312* (P); proximo a Campinas, margens do Rio Pirapitinguí, 22 Nov 1953, *Grotta SPF 15112* (F); Guararema, 1 Nov 1897, *Guttermans 9* (US); Butantã, 24 Nov 1919, *Hoehne s.n. [3493]* (F, MA, US); estrada de Piraporá a Cabreúva, 4 Dec 1924, *Hoehne, 12910* (US); Guararema, km 348 da Via Dutra, 29 Oct 1956, *Hoehne SPF 15754* (F); Cidade Jardim, 1 Dec 1933, *Kuhlmann* s.n. (US); Jundiaí, ca. 10 km SW, Serra do Japí, 8 Oct 1976, *Leitao Filho et al. 3228* (F); São José do Rio Pardo, 24 Oct 1889, *Loefgren 1441* (US); Xiririca, 29 Oct 1894, *Loefgren 2828* (US); Estacão Sagrado do Caracão, linha Sorocabana, 750 m, 3 Oct 1979, *Mizoguchi 1048* (MO); Iporango, BR-2 a 40 km de Jacupiranga em direcão a Curitiba, 12 Nov 1961, *Pabst 6754 (Pereira 6928)* (F, US); São Paulo, 7 Aug 1941, *Praed 5332* (US).

**Paraguay**. **Amambay**: Sierra de Amambay, Jul 1912, *Hassler 11288* (G, K, P).

#### 18. Solanum imbaburense

S.Knapp, Bull. Brit. Mus. Nat. Hist. (Bot.) 19: 94. 1989

http://species-id.net/wiki/Solanum_imbaburense

Figure 47

##### Type.

Ecuador. Imbabura: road Ibarra-Mariano Acosta, E of the pass, montane forest with large trees, 3500–3600 m, 0°20'N, 78°W, 9 Aug 1976, *B. Øllgaard & H. Balslev 8567* (holotype: F; isotypes: AAU, BM [BM000072707], QCA, QCNE, MO [MO-5080092], NY [NY00172013]).

##### Description.

Shrubs ca. 2 m tall. Stems sparsely pubescent with white, dendritic trichomes, the branches of the trichomes short; leaf scars prominent, the stem strongly winged from the decurrent leaf bases; new growth glabrous and shiny except for a few dendritic trichomes on the revolute margins of the leaves. Bark of older stems reddish-brown, glabrate. Sympodial units plurifoliate, branching dichasial. Leaves simple, 3.5–6 cm long, 1–2 cm wide, narrowly obovate to elliptic, coriaceous, the adaxial surfaces glabrous and shiny, the abaxial surfaces glabrous, with a few dendritic trichomes on the revolute margins; primary veins 6–7 pairs, strongly impressed above; base attenuate, strongly decurrent on to the petiole and from there on to the stem; margins strongly revolute; apex acute, occasionally mucronate; petiole 2–4 mm long, strongly decurrent on to the stem, not twining. Inflorescences terminal, later in the fork of the branches, 4–6 cm long, narrowly pyramidal, branching 2–3 times, with 6–10 flowers sparsely pubescent with dendritic trichomes like those of the young stems; peduncle 1.7–3 cm long; pedicels 0.9–1.2 cm long, somewhat nodding at anthesis, tapering from a basal diameter of ca. 0.5 mm to an apical diameter of ca. 1 mm, densely pubescent with erect, dendritic trichomes, articulated at the base and inserted in a sleeve ca. 1 mm long; pedicel scars closely spaced and clustered at the inflorescence branch tips. Buds obovate to elliptic, the corolla strongly exserted from the calyx tube. Flowers all perfect, 5-merous. Calyx tube ca. 3 mm long, conical, the lobes 4–5 mm long, long-triangular to acuminate, the abaxial surfaces of the lobes sparsely dendritic pubescent along the margins, the adaxial surfaces glabrous or with a few minute, glandular trichomes. Corolla 2.5–3 cm in diameter, violet, lobed 3/4 of the way to the base, the lobes 14–15 mm long, 7–8 mm wide, planar at anthesis, the abaxial surfaces of the lobes densely pubescent with dendritic trichomes, these denser along the veins, adaxially with dendritic trichomes along the petal midveins. Filament tube absent; free portion of the filaments ca. 2 mm long, glabrous; anthers ca. 4.5 mm long, 1.5 mm wide, loosely connivent, poricidal at the tips, the pores becoming slit-like with age. Ovary glabrous; style 8–9 mm long, glabrous; stigma bilobed, the surface minutely papillose. Fruit a globose berry, 1.1–1.2 cm in diameter, black when ripe, with thin pericarp; fruiting pedicels 1.8–2 cm long, ca. 1 mm in diameter at the base woody, erect. Seeds ca. 9 per berry, 3.5–4 mm long, 3.5–4 mm wide, flattened and round in outline, the surfaces minutely pitted. Chromosome number: not known.

**Figure 47.**
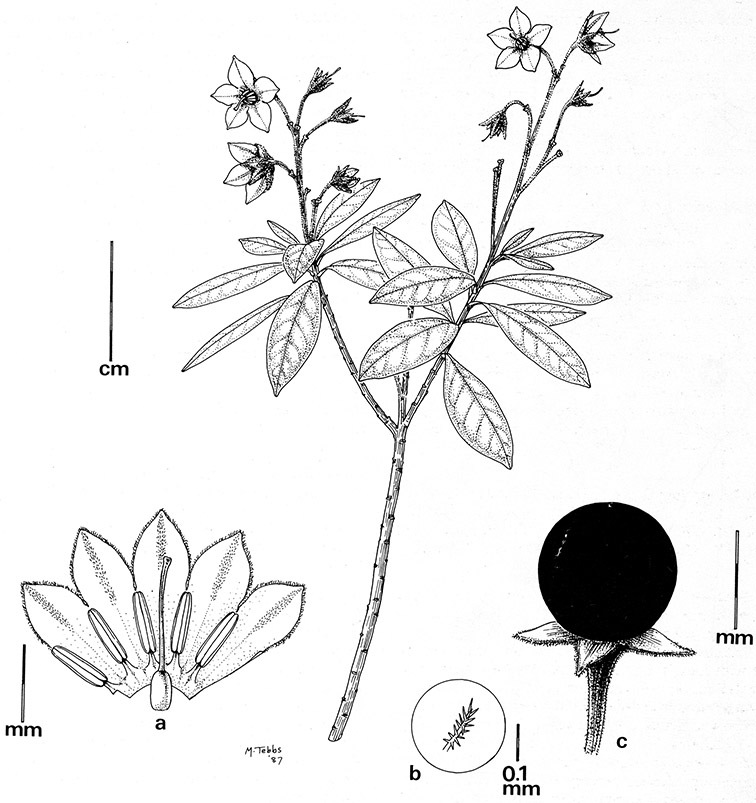
*Solanum imbaburense* S. Knapp. (**A–C** drawn from *Øllgaard & Balslev 8567*). Reproduced from Knapp (1989) with permission of the Natural History Museum Botany Library. Illustration by Margaret Tebbs.

##### Distribution

**Figure 48.**
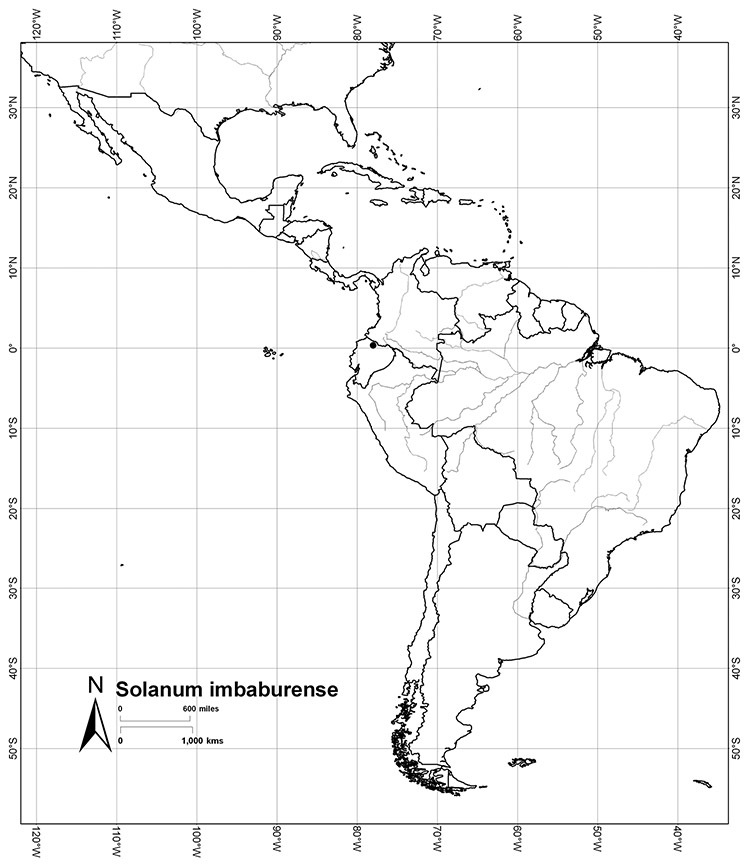
Distribution of *Solanum imbaburense* S.Knapp

(Figure 48). Endemic to N Ecuador; from 3500–3600 m.

##### Ecology.

Moist forests, cloud forests.

##### Conservation status.

Data Deficient (DD); collections too few for assessment. That the species has not been re-collected in 35 years indicates it is certainly of conservation concern and exploration of the type locality to assess its health is a priority.

##### Discussion.

*Solanum imbaburense* is superficially similar to *Solanum stenophyllum* and *Solanum ruizii*, but differs from them in its lax dendritic pubescence, particularly on the inflorescence, its glabrous, coriaceous leave with strongly revolute margins and in its extremely long-acuminate calyx lobes. Some populations of *Solanum stenophyllum* have some glabrous individuals, but unlike *Solanum imbaburense*, these always possess at least some tree-like trichomes on the new growth.

##### Specimens examined.

Known only from the type.

#### 19. Solanum inodorum

Vell., Fl. Flumin. 85. 1829

http://species-id.net/wiki/Solanum_inodorum

Figure 49

Solanum decorticans Sendtn. in Mart., Fl. Bras. 10: 47, tab. 4, Figure 31. 1846. Type:Brazil. “Brasilia australis”, *F. Sellow* s.n. (lectotype, designated here: K [K000438190]; isolectotypes: B [destroyed, F. neg. 2810], F [F-621187, frag. of specimen from B], P [P00336044]).
Solanum decorticatum Dunal, Prodr. [A.P. de Candolle] 13(1): 135. 1852. Type: Brazil. São Paulo, 1833, *C. Gaudichaud 308* (holotype: P [P00336045]).
Solanum laurinum Dunal, Prodr. [A.P. de Candolle] 13(1): 154. 1852. Type: Brazil. São Paulo: “circa promontorium et civitatem”, *J. Bowie & A. Cunningham* s.n. (holotype: BM [BM000517443]; isotypes: G-DC [G00145574, F neg. 6773, IDC microfiche 800-61.2074:III.7], MPU [frag.], S [S07-16646]).
Solanum decorticans Sendtn. var. *laurinum* (Dunal) Witasek, Kaiserl. Akad. Wiss. Wien, Math.-Naturwiss. Kl., Denkschr. 79: 343. 1910. Type: Based on *Solanum laurinum* Dunal

##### Type.

Brazil. Rio de Janeiro: Sin. loc., *Anon*. (no specimens cited; lectotype, designated here: Vellozo, Fl. Flumin. Icones 2, tab. 107. 1831).

##### Description.

Woody vine, climbing with twining petioles. Stems glabrous and shiny; new growth glabrous and shiny, occasionally with a few papillae. Bark of older stems white or creamy white, exfoliating. Sympodial units plurifoliate. Leaves simple, 3–11 cm long, 1–4.5 cm wide, elliptic to narrowly elliptic, coriaceous, slightly discolorous, both surfaces completely glabrous and shiny; primary veins 8–10, only the midrib visible above; base acute; margins entire, strongly revolute; apex acute; petioles 0.4–0.7(-2.6) cm long, glabrous, wrinkling when dry, the outer cells exfoliating like the stems, twining and curling around supports to aid climbing. Inflorescences terminal on axillary short shoots, but leaves on short shoots soon deciduous so the inflorescence appearing axillary, 1–6 cm long, simple to twice branched, with 5–20 flowers, these clustered at the tips or not, glabrous; peduncle 0.7–4 cm long; pedicels 1–1.5 cm long, slender, ca. 0.5 mm in diameter at the base, ca. 1 mm in diameter at the apex, completely glabrous, articulated at the base in a small sleeve; pedicel scars irregularly spaced, some clustered at tips of inflorescence branches, others to 5 mm apart. Buds ellipsoid, the corolla strongly exserted from the calyx before anthesis. Flowers all perfect, 5-merous. Calyx tube 1–1.5 mm long, conical, the lobes 1–1.5 mm long, triangular, glabrous but the tips minutely papillate. Corolla 1.3–2 cm in diameter, white, fragrant (fide Mentz and Oliveira 2004), stellate, lobed 2/3 to 3/4 of the way to the base, the lobes 5–7 mm long, 2.5–4.5 mm wide, spreading at anthesis, densely papillate on the tips and margins, otherwise glabrous. Filament tube minute, the free portion of the filaments 1–1.5 mm long, glabrous, or with a few trichomes at the base (fide Mentz and Oliveira 2004); anthers 4–6.5 mm long, ca. 1 mm wide, ellipsoid, yellow, loosely connivent, poricidal at the tips, the pores usually lengthening to slits with age. Ovary glabrous; style 7–9 mm long, glabrous; stigma minutely clavate, the surface minutely papillose. Fruit a globose or depressed-globose berry, 0.5–0.9 cm in diameter, red or pale reddish pink when ripe, the pericarp thin and shiny, the juice staining scarlet; fruiting pedicels 1–2-1.5 cm long, ca. 1 mm in diameter at the base, slightly woody, pendent. Seeds few per berry, ca. 3 mm long, ca. 3 mm wide, flattened reniform, pale yellow or straw-colored, the surfaces minutely pitted. Chromosome number: not known.

**Figure 49.**
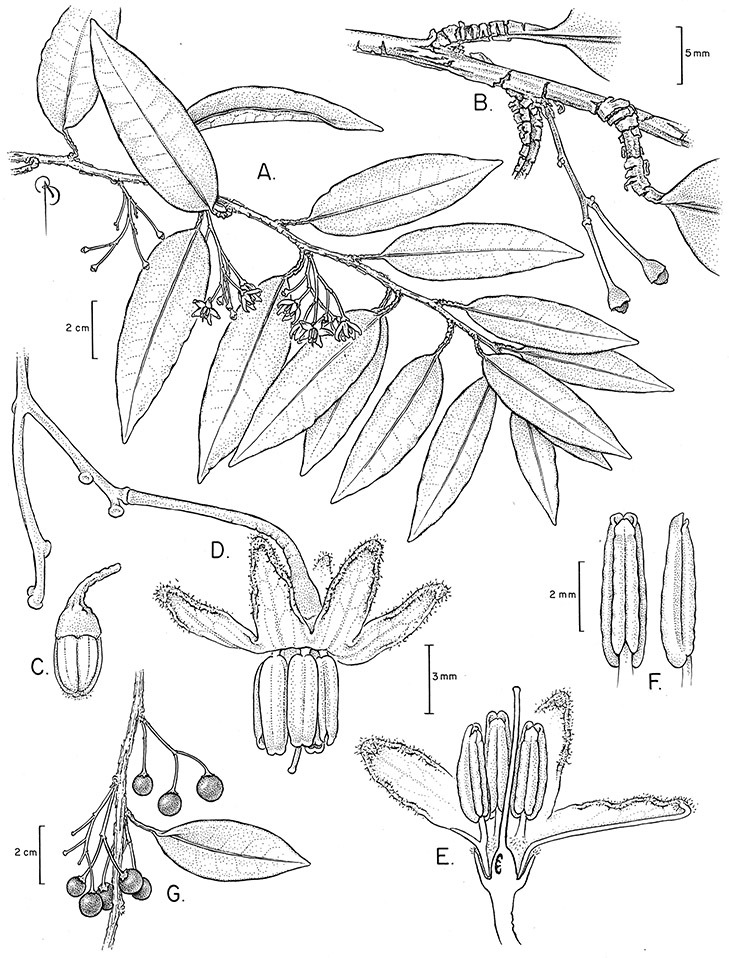
*Solanum inodorum* Vell. (**A–F** drawn from *Hatschbach 26839* **G** drawn from *Reitz & Klein 5290*). Illustration by Bobbi Angell.

##### Distribution

**Figure 50.**
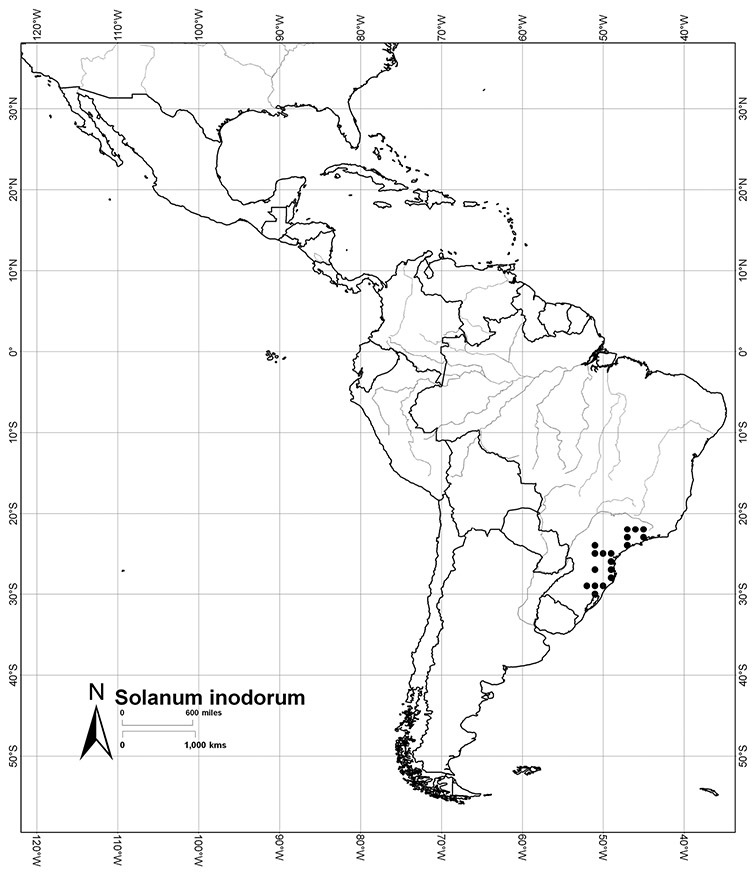
Distribution of *Solanum inodorum* Vell.

(Figure 50). Endemic to southeastern Brazil from the states of São Paulo to Rio Grande do Sul, from 800-1600 m.

##### Ecology.

Occurring in Atlantic rainforest (mata atlântica), *Araucaria* forests and in secondary forests.

##### Conservation status.

Least Concern (LC); EOO >100,000 km^2^ (LC) and AOO >10,000 km^2^ (LC). See Moat (2007) for explanation of measurements.

##### Discussion.

*Solanum inodorum* has long been considered an isolated species in *Solanum*. Bitter (1919) put it (as *Solanum decorticans*) in his large and heterogenous section *Anthoresis* as subsection *Lysiphellos*, a reference to the striking exfoliating bark. *Solanum inodorum* is a striking plant, not easily confused with any other species of *Solanum*. The yellowish, exfoliating bark and the pseudo-axillary inflorescences (see below) make it easily distinguished from *Solanum flaccidum* and *Solanum odoriferum*, with which it is broadly sympatric. The specific epithet is a misnomer, as both this species and *Solanum odoriferum* have fragrant flowers (Mentz and Oliveira 2004).

The inflorescence of *Solanum inodorum* appears to be axillary like the inflorescences of members of section *Pteroidea* (the *Solanum mite* group of the Potato clade, see Knapp and Helgason 1997), but is in fact terminal on a fore-shortened axillary shoot. The leaves of this shoot are vestigial and soon deciduous, but can occasionally be seen in young shoots. This morphology of inflorescences borne terminally on short shoots is found in other members of the Dulcamaroid clade such as *Solanum valdiviense* of Chile.

**Figure 51.**
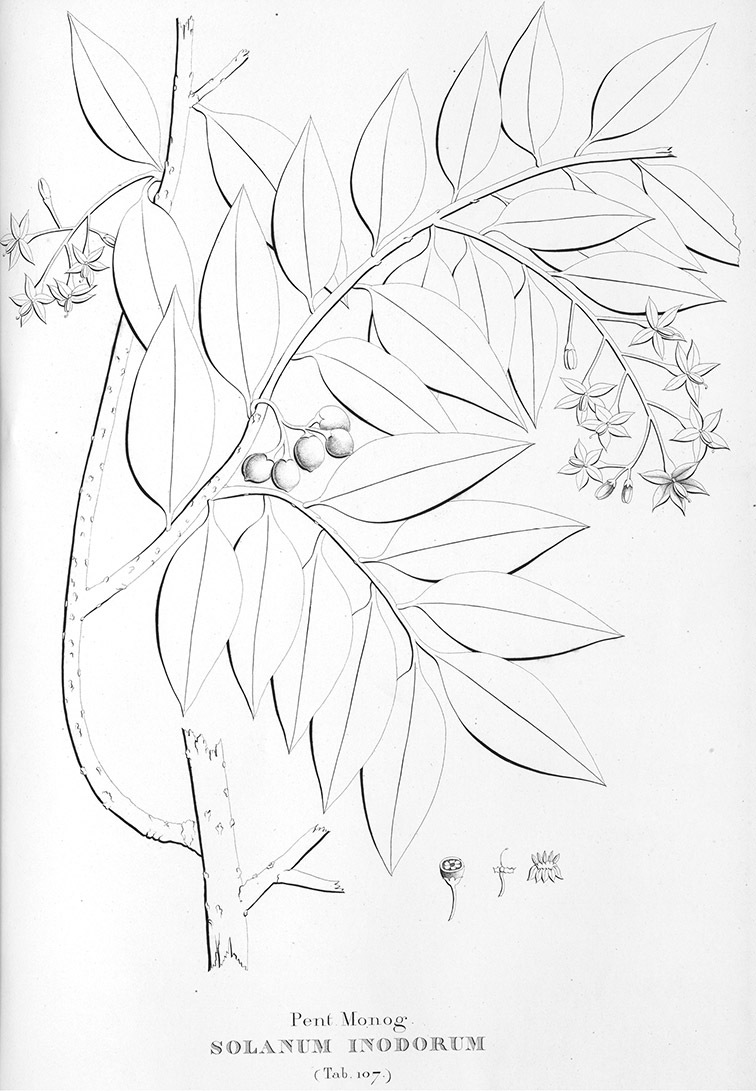
Lectotype of *Solanum inodorum* Vell. (Vellozo 1831: tab. 107). Reproduced with permission of the Natural History Museum Botany Library.

Lectotypification of Vellozo names, in the absence of specimens (Carauta 1973), is best done with the plates from that work (Vellozo 1831); in this case the plate is easily identifiable as *Solanum inodorum* (Figure 51). Sendtner’s epithet “*decorticans*” is represented by two syntypes (*Schott* s.n. from “Sebastianopolis” and *Sellow* s.n. from “Brasilia australis”). I have only been able to find the Sellow collection and select a well-preserved sheet (K000438190) as the lectotype of *Solanum decorticans*.

##### Specimens examined.

**Brazil**. **Minas Gerais**: Poços de Caldas, 25 Mar 1920, *Hoehne 3831* (US); Ouro Fino, 10 May 1927, *Hoehne 19543* (US); Delfim Moreira, São Francisco dos Campos, 12 Jun 1950, *Kuhlmann 2504* (F); Caldas, 18 Apr 1886, *Regnell II 221* (K, LE, S, US). **Paraná**: Tijucas do Sul, Palermo, 20 Aug 1999, *Barbosa et al. 355* (B, MO); roadside of highway 116 about 40 km NE of Curitiba, 1000 m, 5 Jan 1974, *Conrad & Dietrich 2070* (MO); Campina Grande do Sul, Cerne, 17 Aug 1988, *Cordeiro & Budziak 562* (S); Adrianópolis, Parque das Lauráceas, próximo ão Rio Caratuva, 28 Jul 1999, *Cruz & Abe 136* (B, G); Ipiranga, Serra do Mar, 2 Sep 1911, *Dusén 12114* (F, GH, S, SI); São José dos Pinhais, Vossoroca, 15 Aug 1951, *Hatschbach 2465* (US); Palmeira, Colonia Wietmarsum, 950 m, 23 Sep 1962, *Hatschbach 9827* (B, US); Campina Grande do Sul, Serra do Lapinha, 4 Aug 1963, *Hatschbach 10151* (B); Palmas, Santa Bárbara, 19 Oct 1966, *Hatschbach 15018* (F); Mandirituba, Areia Branca dos Assis, 1 Sep 1986, *Kummrow & Cordeiro 2796* (G); São José dos Pinhais, Col. Santos Andrade, 15 Jun 1982, *Oliveira 548* (MO); Balsa Nova, Serra São Luis do Purunã, 7 Oct 1996, *Poliquesi & Barbosa 597* (MA); **Rio Grande do Sul**: Canela, Gramado, 13 Jun 1937, *Rambo 2236* (B); São Francisco de Paula, Fazenda Engelbert, 2 Jan 1954, *Rambo 56264* (B); São Francisco de Paula, Taimbé, 1000 m, 18 Dec 1950, *Sehnem 5148* (B, US); São Francisco de Paula, Aratinga, Oct 1984, *Sobral 3211* (F); **Rio de Janeiro**: Itatiaia, km 12, 1700 m, 29 May 1935, *Brade 14654* (B); Itatiaia, Parque Nacional de Itatiaia, Trilha para os Tres Picos, 19 Oct 2010, *Fraga et al. 2885* (K); Parque Nacional de Itatiaia, Donat, Jul 1953, *Pereira et al. 74* (F); Rio do Funil, 2 Oct 1952, *Pereira* s.n. (US); Nova Friburgo, Macaé de Cima, caminho para a Pedra Bicuda, 3 Aug 1989, *Pessoa et al. 474* (BM, F); **Santa Catarina**: Azambuja, Brusque, 70 m, 11 Aug 1953, *Klein 540* (B); Pilões, Palhoça, 200 m, 2 Aug 1956, *Reitz & Klein 3549* (B); Brusque, 100 m, 20 Sep 1948, *Reitz 3550* (F); Pirão Frio, Sombrio, 10 m, 11 Jul 1959, *Reitz & Klein 8920* (G); Rio dos Patos, Lebon Régis, 900 m, 13 Jul 1962, *Reitz & Klein 13224* (B); **São Paulo**: Serra do Japi, Jundiaí, 27 Jun 1988, *Buzato 20633* (K); Cantareira, 28 Jun 1919, *Hoehne 3375* (US); Mata do Governo, 8 May 1924, *Hoehne 9715* (US); Parque do Estado, 26 Oct 1931, *Hoehne 29800* (BH, US); Interlagos, 12 Jul 1954, *Hoehne* s.n. (G); Interlagos, 12 Jul 1954, *Hoehne SPF 15383* (F); Osasco, Cemeterio Parque de Paz, 26 Jul 1985, *Leitao Filho & Rodrigues 17694* (CORD); Campos de Jordão, Dec 1948, *Leite* (US); São Carlos do Pinhal, 17 Mar 1888, *Loefgren 719* (US); Campos de Bocaina, 19 Apr 1894, *Loefgren & Edwall 2462* (US); Pico de Jaraguá, 25 km NW from Sao Paulo City, 29 Sep 1979, *Mizoguchi 985* (MO); Jardim Botânico, nativa no Jardim Botanico e Parque do Estado, area 30, 21 May 1974, *Silva 297* (F); Avenida Paulista, 21 Oct 1906, *Usteri 15a* (US); Serra do Japi, 13 Jun 1988, *Vasconcellos Neto 20401* (K).

#### 20. Solanum kulliwaita

S.Knapp, PhytoKeys 1: 35. 2010

http://species-id.net/wiki/Solanum_kulliwaita

Figure 52

##### Type.

Peru. Cusco: Prov. La Convención, Dist. Ocobamba, Mesa Pelada, 12°54'13"S, 72°37'06"W, 2613 m, 23 March 2004, *L. Valenzuela, E. Suclli & G. Calatayud 3163* (holotype: USM; isotypes: CUZ [n.v.], MO, MOL [n.v.], NY [NY00824906]).

##### Description.

Woody vine or scandent shrub, the height not recorded, the branches arching. Stems sparsely pubescent with simple uniseriate multicellular trichomes 0.5–1 mm long, glabrescent, slightly winged from the decurrent leaf bases; new growth pubescent with simple uniseriate trichomes 0.5–1 mm, these occasionally dendritic. Bark of older stems dark reddish brown, shiny. Sympodial units plurifoliate. Leaves simple, (2-)3.5–8.5 cm long, 1–3 cm wide, narrowly elliptic to lanceolate, slightly fleshy, the upper surfaces sparsely pubescent with simple or occasionally furcate or branched trichomes on the lamina, more densely pubescent on the midvein, the lower surfaces glabrous or with a few scattered simple uniseriate trichomes along the midvein; primary veins 7–9 pairs, the veins often drying blackish brown; base acute to attenuate; margins entire, sometimes revolute, densely pubescent in the basal quarter to third with simple trichomes extending from the petiole; apex acute; petioles 0.7–2 cm long, adaxially densely pubescent along the groove with golden simple or occasionally furcate uniseriate trichomes, not apparently twining. Inflorescences terminal or lateral, 9–11 cm long, 3–5 times branched, with 10–20 flowers, densely pubescent with simple uniseriate trichomes mostly 0.3–0.5(-1) mm long, purple in live plants and retaining pigmentation in dried material, the cells of the trichomes small and weak-walled, usually collapsing and tangled, the lateral cell walls dark-pigmented, the terminal cells spheroidal and apparently glandular; peduncle 1.5–3.5 cm long; pedicels 1–1.2 cm long, ca. 0.5 mm in diameter at the base, ca. 1 mm in diameter at the apex, slender, erect to nodding, densely pubescent like the rhachis, articulated at the base and inserted into a short sleeve or above the base and leaving a peg ca. 2 mm long; pedicel scars irregularly spaced 0.5–5 mm apart, usually grouped. Buds ellipsoid, the corolla strongly exserted from the calyx tube before anthesis. Flowers all perfect, 5-merous. Calyx tube 2–2.5 mm long, cup-shaped, narrowing gradually to the pedicel, the lobes 2.5–3.5 mm long, the lower portion broadly deltate, the distal part an apiculate tip to 2 mm long, densely pubescent with simple uniseriate trichomes like those of the inflorescence rhachis, these apparently glandular, the adaxial surface glabrous. Corolla 2.3–2.5 cm in diameter, purple, stellate, lobed 2/3 to 3/4 of the way to the base, the lobes 9–12 mm long, 4–5 mm wide, spreading, the tips and margins densely pubescent on the abaxial surface with weak, collapsing simple uniseriate trichomes like those of the inflorescence, but smaller and not apparently glandular, adaxially glabrous. Filament tube minute, the free portion of the filaments 1–2 mm long, glabrous; anthers 3.5–4.5 mm long, 1–1.5 mm wide, ellipsoid, loosely connivent, poricidal at the tips, the pores lengthening to slits with age. Ovary glabrous; style 7–8 mm long, glabrous; stigma capitate, the surface minutely papillose. Fruit a globose berry, ca. 1 cm in diameter (immature?), black when ripe, the pericarp thin, not shiny, glabrous; fruiting pedicels 1.5–1.7 cm long, ca. 1.5 mm in diameter at the base, woody, more or less nodding. Seeds not known. Chromosome number: not known.

**Figure 52.**
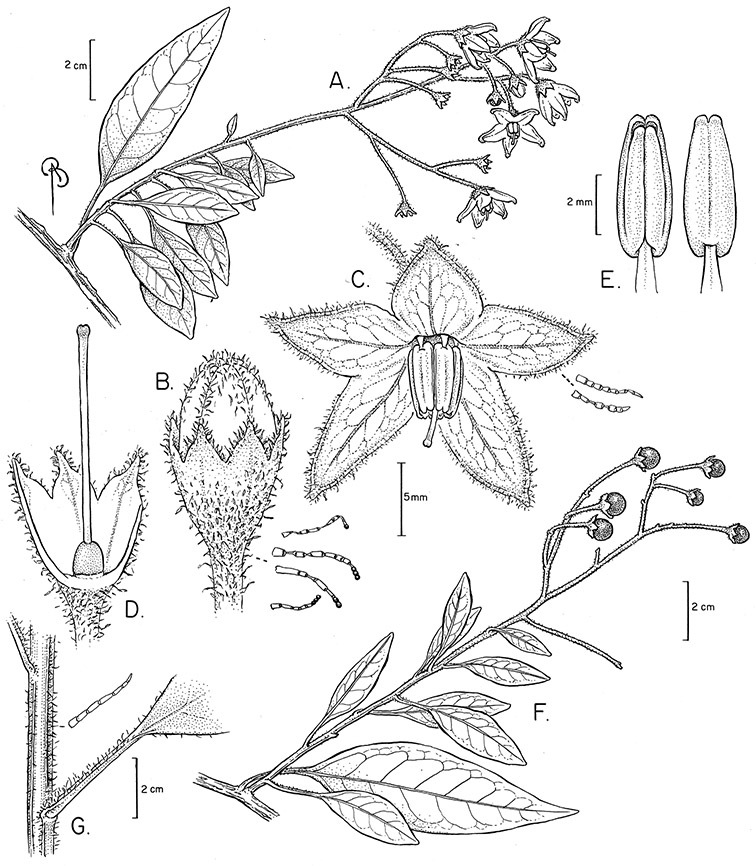
*Solanum kulliwaita* S.Knapp. (**A–E** drawn from *Valenzuela 3163* **F–G** drawn from *Galiano 6137*). Illustration by Bobbi Angell.

##### Distribution

**Figure 53.**
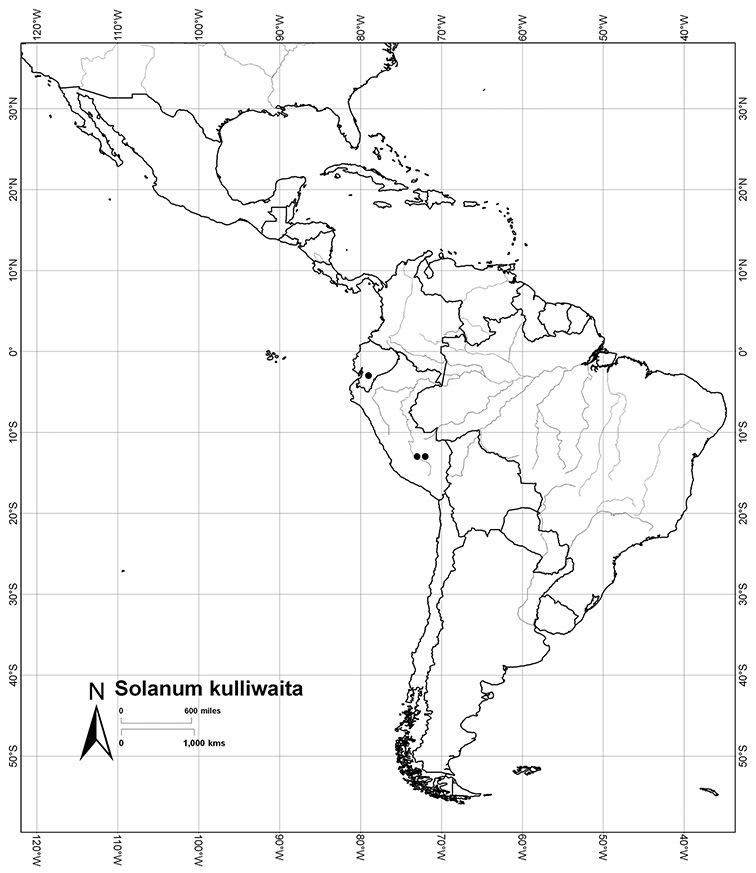
Distribution of *Solanum kulliwaita* S.Knapp.

(Figure 53). Disjunct between Azuay province in southern Ecuador and the type locality in the valley of the Río Urubamba in the Department of Cusco in southern Peru (Mesa Pelada), from 2400–2600 m.

##### Ecology.

Cloud forests.

##### Conservation status.

Possible Near Threatened (possible NT); EOO <45,000 km^2^ (NT) and AOO >10,000 km^2^ (LC). See Moat (2007) for explanation of measurements. Known only from a few widely disjunct collections in a very narrow geographical area outside any protected areas, this species can be considered of conservation concern.

##### Discussion.

*Solanum kulliwaita* is most similar morphologically to *Solanum sanchez-vegae* from northern Peru. It can be distinguished from that species by its leaves that are glabrous beneath and with a dense covering of uniseriate trichomes on the adaxial surface of the petiole, the ciliate lower leaf margins and the distinctive uniseriate glandular trichomes of the inflorescence. The inflorescence trichomes are unusual in the Dulcamaroid clade in having three globular cells at the apex and in drying purple (and being purple in live plants, fide *Valenzuela et al. 3163*). Trichomes of the rest of the plant (i.e., leaves) are not glandular. Specimens from Ecuador have glabrous leaves, but the trichomes of the inflorescence are identical to those found in Peruvian populations.

##### Specimens examined.

**Ecuador**. **Azuay**: Cuenca-Loja highway, km 43, 15 Jul 1977, *Boeke & Loyola 2148* (BM, NY); Cumbe, 3000 m, 22 Apr 1968, *Harling et al. 8732* (BM); Road Cuenca-Loja, ca 20 km south of Cumbe, 3000 m, 19 Mar 1974, *Harling & Andersson 12671* (BM).

**Peru**. **Cusco**: La Convención, Maranura, Dist. Maranura, Mesapelada, 2450 m, 19 Apr 2004, *Galiano et al. 6137* (MO, NY, USM).

#### 21. Solanum laxum

Spreng., Syst. Veg. 1: 682. 1824

http://species-id.net/wiki/Solanum_laxum

Figure 54

Solanum jasminoides Paxton, Paxton’s Mag. Bot. 8: tab. 5. 1841. Type: Cultivated in Great Britain, “from seed sent to Messrs. Young of Epsom from the Glasgow Botanic Garden”, probably from southern Brazil (see discussion below), (no specimens cited, lectotype, designated by Symon 1981, pg. 65: Paxton, Paxton’s Mag. Bot. 8: tab. 5. 1841).
Solanum boerhaviifolium Sendtn. in Mart., Fl. Bras. 10: 48, fig 11. 1846, as "boerhaviaefolium" Type: Brazil. “in Brasilia australi”, *F. Sellow* s.n. (lectotype, designated here: P [P00324767, Morton neg. 8153]; isolectotype: K [K000590026]).
Solanum jasminoides Paxton var. *boerhaviifolium* (Sendtn.) Kuntze, Revis. Gen. Pl. 3(2): 226. 1898. Type: Based on *Solanum boerhaviifolium* Sendtn.
Solanum jasminoides Paxton var. *glaberrimum* Kuntze, Revis. Gen. Pl. 3(2): 226. 1898. Type: Bolivia. Cochabamba: Prov. Chapare, Río Juntas, 3500-3600 m, *O. Kuntze* s.n. (lectotype, designated here: NY [NY00172053]).
Solanum jasminoides Paxton var. *pilosum* Kuntze, Revis. Gen. Pl. 3(2): 226. 1898. Type: Bolivia. Cochabamba: Prov. Chapare, Río Juntas, 3500-3600 m, *O. Kuntze* s.n. (lectotype, designated here: NY [NY00172054]).
Solanum jasminoides Paxton var. *pubinerve* Kuntze, Revis. Gen. Pl. 3(2): 226. 1898. Type: Bolivia. Cochabamba: Prov. Chapare, Río Juntas [cult?], 3500-3600 m, 13-21 Apr 1892, *O. Kuntze* s.n. (lectotype, designated here: NY [NY00172055]).
Solanum dietrichiae Domin, Biblioth. Bot. 89: 1130. 1929. Type: Australia, Queensland, Brisbane River, *A. Dietrich 2789* (holotype: PR [PR-530859]).
Solanum boerhaviifolium Sendtn. var. *calvum* C.V. Morton, Revis. Argent. Solanum 66. 1976. Type: Argentina. Misiones: Posadas, Bonpland, 6 Jan 1908, *E. Ekman 817* (holotype: US [US-1574743]; isotype: S [S07-16710]).

##### Type.

Uruguay. Montevideo: Montevideo, *F. Sellow* s.n. (holotype: B, destroyed; no duplicates traced). Uruguay. Montevideo: Montevideo, 1826-1830, *J. Anderson* s.n. (neotype, designated here: BM [BM000935924]).

##### Description.

Woody vine, the base sometimes to > 10 cm in diameter, twining by petioles. Stems strongly zig-zag, glabrous or puberulent when young with white simple uniseriate trichomes < 0.5 mm long; new growth glabrous to minutely and sparsely puberulent. Bark of older stems green or reddish green or often purplish green when growing in bright sunlight. Sympodial units plurifoliate. Leaves simple or very occasionally deeply pinnatifid with 1–4 irregular lobes, 1–5 cm long, 0.5–2 cm wide, ovate or elliptic to narrowly elliptic (occasionally pinnatifid with up to 5 lobes), widest in the basal 1/3, membranous to chartaceous, the upper surfaces glabrous or with a few simple uniseriate trichomes along the midrib, the lower surfaces glabrous or with variously dense tufts of simple uniseriate trichomes to 1 mm long in the vein axils and occasionally extending sparsely to the lamina; primary veins 4–6 pairs, with a strong and obvious intramarginal vein, the midrib often keeled; base truncate, often oblique and asymmetrical; margins entire, if lobed the lobes reaching nearly to the midrib, the basal ones smaller; apex acute to acuminate; petioles 0.4–2.5(+) cm long, glabrous or minutely puberulent in the groove on the adaxial surface, twining. Inflorescences terminal, or later lateral, to 5 or more cm long, to many times branched, but usually only 2–3 times branched, with up to 50 flowers, glabrous; peduncle 0.5–4 cm long, very variable in length depending on the size and age of the inflorescence; pedicels 1–1.5 cm long, < 0.5 mm in diameter at the base and apex, filiform, nodding or spreading at anthesis, glabrous, articulated at the base from a small sleeve, leaving a small peg on the inflorescence axis; pedicel scars irregularly spaced 2–6 mm apart. Buds somewhat inflated, ellipsoid, broadest in basal part, the corolla strongly exserted from the calyx before anthesis. Flowers all perfect, 5-merous. Calyx tube 1–1.5 mm long, conical to somewhat flattened, the lobes 1–1.5 mm long, deltate with an elongate tip to 1 mm, often drying black, glabrous or the tip with a few minute trichomes. Corolla 1.8–2.2 cm in diameter, white or pale violet, rotate-stellate, lobed ca. 1/2 way to the base, the lobes 7–9 mm long, 5–6 mm wide, planar or spreading at anthesis, glabrous to minutely pubescent with tiny simple uniseriate trichomes abaxially, these < 0.2 mm long, glabrous adaxially. Filament tube minute, the free portion of the filaments 1–1.5 mm long, glabrous or minutely puberulent within; anthers 3.5–4 mm long, 1–1.5 mm wide, ellipsoid, loosely connivent, sagittate at the base, poricidal at the tips, the pores lengthening to slits with age. Ovary glabrous; style 7–8 mm long, pubescent with simple uniseriate trichomes < 0.2 mm long within the anther tube, glabrous where exserted beyond the anthers; stigma a minutely papillate area on the tip of the style, occasionally somewhat bilobed to clavate. Fruit a globose berry, ca. 1 cm in diameter, blackish purple when ripe, the pericarp thin and shiny, glabrous; fruiting pedicels 1.2–1.5 cm long, ca. 0.5 mm in diameter, not markedly woody, pendent. Seeds 10–20 per berry, ca. 3 mm long, ca. 2.5 mm wide, flattened reniform, pale tan, the surfaces minutely pitted, the testal cells pentagonal. Chromosome number: n=12 (Moscone 1992, as *Solanum boerhaviifolium*).

**Figure 54.**
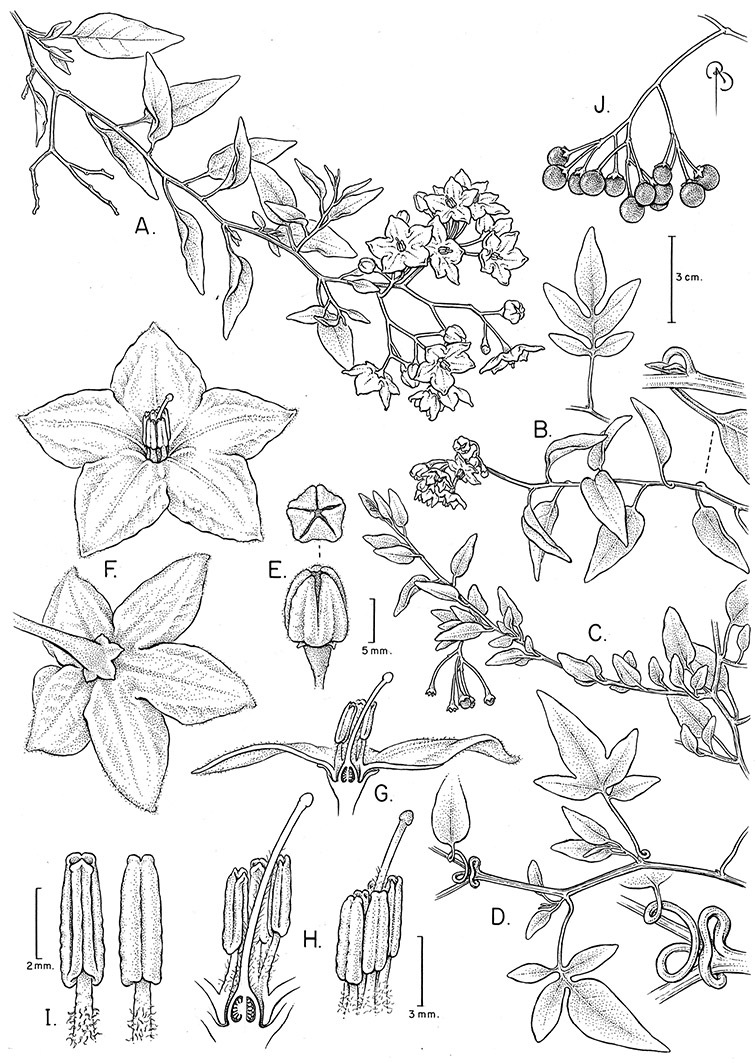
*Solanum laxum* Spreng. (**A** drawn from *Nee 36730* and M.Nee photographs of plant in the field **B** drawn from *Bohs & Nee 3191* **C** drawn from *Hatschbach 22971* **D** drawn from *Steyermark 53983* **E–I** drawn from plants cultivated in London). Illustration by Bobbi Angell.

##### Distribution

**Figure 55.**
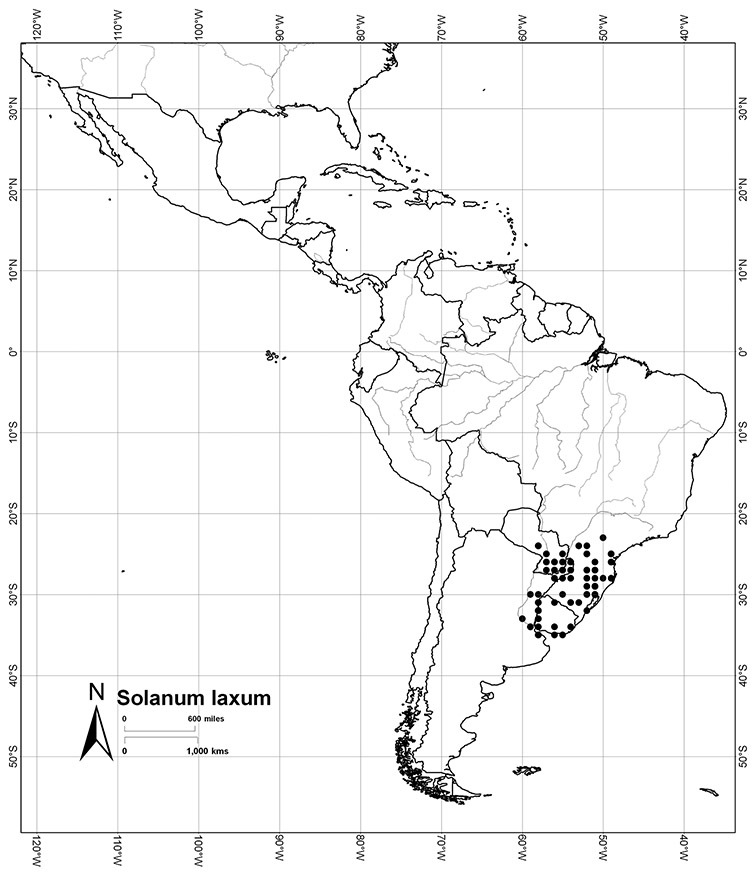
Distribution of *Solanum laxum* Spreng.

(Figure 55). Native to southeastern Brazil from the states of Minas Gerais to Rio Grande do Sul to the mouth of the Río de la Plata in Argentina and Uruguay, and into Paraguay, from nearly sea level to above 500 m elevation. Widely cultivated worldwide in both temperate and subtropical zones, often escaped and naturalised.

##### Ecology.

Growing as a native in Atlantic rainforest, *Araucaria* forests, gallery forests and open forest margins. *Solanum laxum* is escaped and naturalised throughout the tropics and subtropics in suitable habitat, it has a wide tolerance of light freezing (as evidenced by its common cultivation in Great Britain).

##### Common names.

Cultivated plants from Colombia: manto de la virgin, manto de la reina (*Duque-Jaramillo 3006*); Ecuador: martiño enrededera (*Steyermark 53983*); UK and USA: potato vine.

##### Conservation status.

Least Concern (LC); EOO >100,000 km^2^ (LC) and AOO >10,000 km^2^ (LC). See Moat (2007) for explanation of measurements.

##### Discussion.

**Figure 56.**
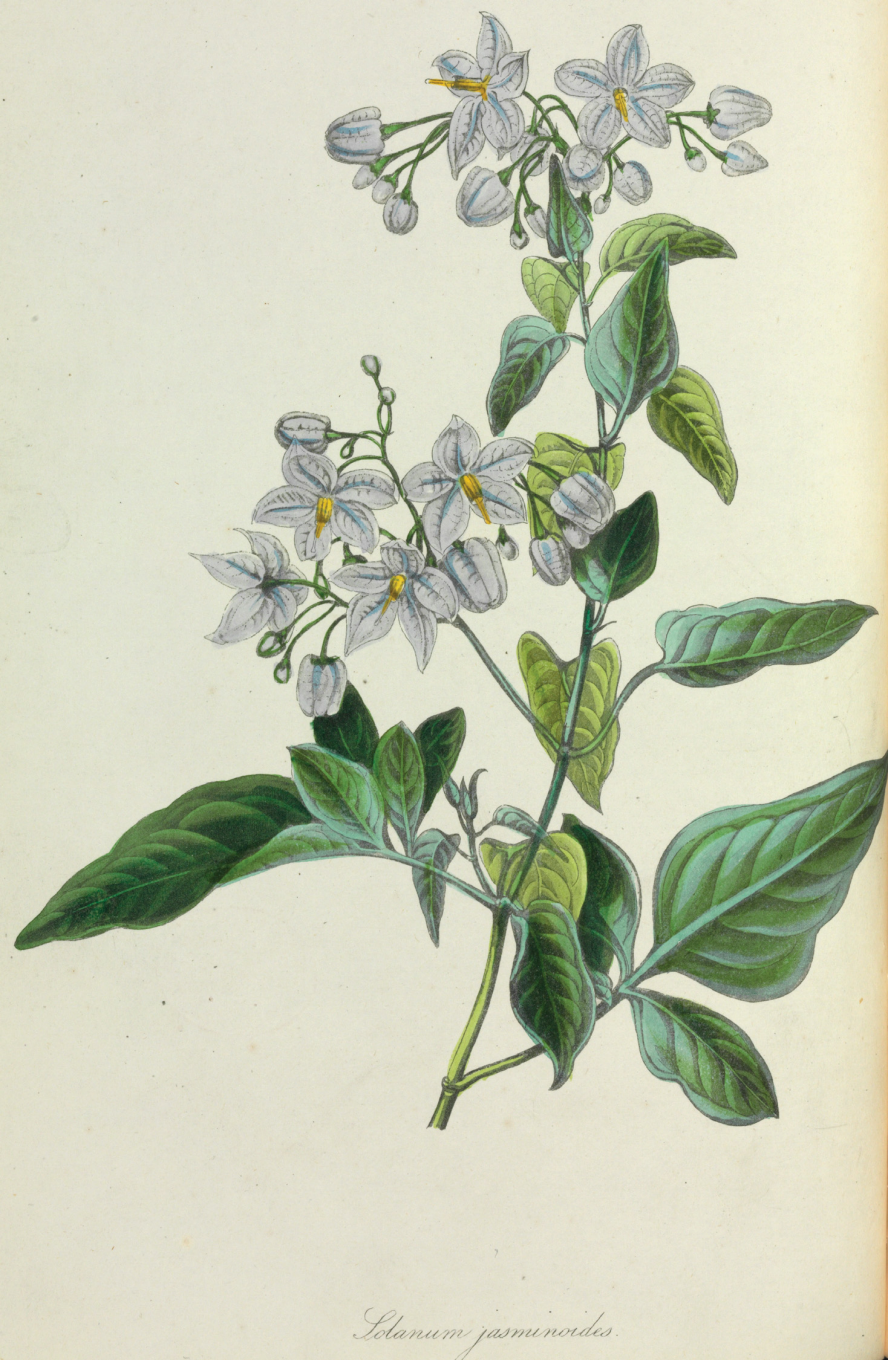
Lectotype of *Solanum jasminoides* Paxt. (Paxton 1841: tab. 5) Reproduced with permission of the Natural History Museum Botany Library.

*Solanum laxum* was long known in cultivation as *Solanum jasminoides* (see Figure 56), but the former name has priority and is slowly becoming accepted in botanical (see Mentz and Oliveira 2004) and horticultural circles (see Royal Horticultural Society (RHS) Plant Finder, http://apps.rhs.org.uk/rhsplantfinder/index.asp ). The original source of material cultivated in Great Britain was southern Brazil, and in England the plant overwinters well even in very cold winters (hardy to USDA zone 8), where it dies back from frost, but quickly resprouts. In cultivation vines of *Solanum laxum* grow very large; a vine growing up the south-facing wall of the Chelsea Physic Garden in London is over 10 cm in diameter at the base.

*Solanum laxum* is similar, and probably closely related, to the sympatric *Solanum viscosissimum* with which it shares rotate-stellate corollas, pubescent styles and purplish black berries. It differs from that species in its almost always simple leaves and in the tufts of trichomes in the vein axils of the leaf undersurfaces; *Solanum viscosissimum* usually has at least some deeply pinnatifid leaves and has long, glandular trichomes evenly spread over the leaf surfaces. Flowers of *Solanum laxum* are usually white, but can have a purplish tinge, especially when growing in strong light. *Solanum laxum* could also potentially be confused with *Solanum flaccidum*, also sympatric, which differs in its larger purple or violet flowers with anthers borne on unequal filaments and in its more evenly distributed pubescence on leaf surfaces and stems. A few collections (e.g., *Stuckert 11592*, G) have deeply pinnatifid leaves, but can be distinguished from other similar species such as *Solanum seaforthianum* (also common in cultivation) by the conspicuous tufts of trichomes in the vein axils on the leaf undersides. These pinnatifid leaves are probably juvenile, and are very occasionally found on stems mixed with simple leaves (e.g., *Osten 8265*) as is common in other species of the Dulcamaroid clade.

Although the tufts of trichomes in the vein axils of the leaf undersides are a good diagnostic character for *Solanum laxum*, some populations and individuals appear to lack pubescence of any sort. These plants have been called var. *calvum*, and they differ from other populations only in pubescence density; there are usually a few trichomes, but they can be difficult to see without a microscope. This variation is common in the Dulcamaroid clade, and in many other non-spiny solanums.

The Sellow collection used by Sprengel to describe *Solanum laxum* was destroyed in Berlin; I have found no Sellow collections of *Solanum laxum* from Uruguay. Many of Sellow’s collections were sent to Berlin, and plants were grown there from seeds sent from Uruguay in 1823 (Krausch 2002). The plant used by Sprengel may have been one grown in the Botanic Garden in Berlin rather than a herbarium specimen collected in Uruguay. I have selected as a lectotype for *Solanum laxum* a specimen (BM000935924, the only one I have seen in several hundred collections examined) collected in Montevideo, Uruguay attributed to James Anderson, the botanist aboard HMS Adventure. The ship, captained by Philip Parker King, surveyed the complex area around the Straits of Magellan from 1826 to 1830, but little information as to the orgins of the plants from the voyage exists (see entry for King on JSTOR Plants, http://plants.jstor.org/person/bm000063285 ). This specimen, although a unicate and not collected by Sellow, is almost contemporary with Sellow’s time in Uruguay and matches in the protologue in having glabrous leaves.

An un-numbered Sellow collection from Brazil at B (“Brasilia australi”was used by Sendtner (1846) in describing *Solanum boerhavifolium*; this is probably no longer extant. Another Sellow specimen at P (P00324767) donated by Berlin, and annotated “S. bôrhaaviaefolium Sendt.” in Sendtner’s hand, is selected as the lectotype. It bears a number “250” in a different handwriting. Another Sellow sheet from B at P (P00324768) is sterile and does not have the specific name written in Sendtner’s handwriting, but may be a duplicate; a sheet at K (K000590026, also with the species name in Sendtner’s hand, but labelled on the sheet “Klotsch”) is probably also be a duplicate, but in the absence of specific locality information or collection number it is impossible to tell.

The origin of the plants described as *Solanum jasminoides* is discussed by Harley (1970), who speculates that the plants grown in the Young Nursery in Epsom, Surrey, were originally from Sir William Hooker, who received material from Brazil collected by Tweedie. A specimen at Kew collected in 1832 in Rio Grande do Sul (K000545751, labelled “Parana”) by Tweedie is thought by Harley (1970) to probably be the origin of both the Kew and the Epsom material; Tweedie collected both specimens and seeds for Hooker. I am not sufficiently convinced of this chain of custody to use the K sheet as epitype material.

The epithet *boerhaviifolium* was spelled “boerhaviaefolium” by Sendtner (1846) and although the name honours Hermann Boerhaave, the original spelling with a single ‘a’ is retained, following Article 60.1 of the Code (McNeill et al. 2012).

##### Specimens examined [native range].

**Argentina**. **Buenos Aires**: Campana, 25 May 1951, *Boelcke 4910* (SI); Las Palmas, 11 Sep 1952, *Boelcke 6358* (SI); Arroyo Brazo Largo, Delta Paraná, 27 Mar 1937, *Burkart 8330* (F, SI); Punta Lara, ribera de la Río de la Plata, 25 Oct 1931, *Cabrera 1812* (SI); Buenos Aires, Recoleta, 10 Feb 1912, *Dieckmann* s.n. (SI); Buenos Aires, *Fox 182* (K); Cruz Colorada, 21 Nov 1911, *Hicken* s.n. (SI); Cuy Colonda, Tigre, 21 Nov 1911, *Hicken 24941* (SI); Hurlingham, Pdo. Morón, 23 Oct 1976, *Krapovickas 29707* (G); Delta, Arroyo Méndez Grande, 31 Oct 1929, *Scala* s.n. (F); Delta, Nov 1914, *Scala* s.n. (SI); Punta Lara, La Plata, 29 Oct 1946, *Sparre 14* (K); San Fernando, *Stuckert 11592* (G); Barrancas al Sud, 18 Sep 1902, *Venturi 177* (S, SI); **Corrientes**: Santo Tomé, Gobernador Virasoro, 11 Oct 1969, *Pedersen 9255* (K); Monte Caseros, Rta. 127 y Arroyo Curuzú Cuatiá, 20 Feb 1979, *Schinini et al. 17440* (K); Santo Tomé, Estancia Timbó, 38 km N de Santo Tomé, ruta 40, 5 Nov 1996, *Vanni & Maruñak 3731* (GH); **Córdoba**: Córdoba, *Castellanos 74* (SI); **Entre Ríos**: Concordia, Salto Grande, 21 Sep 1951, *Boelcke 4843* (K); Gualeguaychú, 6 Jan 1932, *Burkart 4134* (SI); Gualeguaychú, 20 Sep 1961, *Burkart 22718* (SI); Concepción del Uruguay, Concepción del Uruguay a Colón, 15 Dec 1963, *Burkart 24920* (SI); Feliciano, Paso Yunque, 18 May 1964, *Burkart et al. 25452* (SI); La Paz, Paso Yunque, Río Guayquiraró, 13 Apr 1968, *Burkart et al. 27072* (SI); Concordia, Salto Grande, 21 Sep 1951, *Correa & Bacigalupo* s.n. (SI); Gualeguaychú, Río Brazo Largo, Vivero Experimental del Delta, 15 Aug 1946, *Krapovickas 3067* (BH, K); Concepcion del Uruguay, Oct 1878, *Lorentz* s.n. (BM, GH); Uruguay, Arroyo La China, 24 Dec 1941, *Nicora 3294* (SI); Concordia, River Ayui on road from Concordia to Salto Grande, 5 Oct 1978, *Renvoize et al. 2911* (F, F, K, K); **Mendoza**: Mendoza, 20 Aug 1913, *Sanzin 35* (SI); **Misiones**: Concepción, Santa María, Oct 1977, *Cabrera et al. 28674* (SI); San Pedro, San Pedro, 1 Nov 1958, *Gamerro & Toursarkissian 67* (SI); Apostoles Pueblo del Sur, 4 Nov 1944, *Ibarrola 1001* (K); Apóstoles, Río Chimiray, 9 Nov 1944, *Ibarrola 1137* (A); Posadas, Bonpland, *Lillieskold* s.n. (F, G., S); Gral. Manuel Belgrano, Bernardo de Irigoyen, 29 Sep 1970, *Maruñak 137* (F); El Dorado, 8 Mar 1944, *Meyer 6805* (S); Candelaria, Santa Ana, 13 Dec 1945, *Montes 1545* (K, S); Apóstoles, Río Chimiray, Ruta Prov. 94, 2 km de Garruchos camino a Azara, 100 m, 10 Oct 1996, *Morrone et al. 1108* (SI); Iguazú, 20 km E de Wanda a J.J. Lanusse, 24 Jan 1973, *Schinini & Fernández 6053* (G); Candelaria, Santa Ana, rutas 12 y 4, Santa Ana, 22 Aug 1987, *Schinini 25460* (F, GH, K); San Ignacio, km 110, 26 Dec 1945, *Schwarz 1739* (G); San Ignacio, Colonia Corpus, 20 Oct 1948, *Schwarz 6445* (K); Cainguás, Puerto Mineral, 24 Aug 1950, *Schwarz 10698* (BH, G); Iguazú, Parque Nacional Iguazú, ruta 101, 13 Oct 1993, *Tressens et al. 4439* (BH, GH); Gral. Manuel Belgrano, San Antonio, 4 km NW de San Antonio (Ayo. Rolador), 29 Mar 1996, *Tressens et al. 5596* (GH, K); Guaraní, Predio Guaraní, ruta 15 y arroyo afluente del Itá Pirú, a ca. 2 km del limite con Papel Misionero, 3 Nov 1999, *Tressens et al. 6465* (F); General San Martín, Arroyo Tabay y ruta 7, 27 Aug 1987, *Vanni 734* (F); Iguazú, Ruta Nac. 101, Arroyo Santo Domingo, 5 Mar 1995, *Zuloaga et al. 5278* (MO).

**Brazil**. **Bahia**: Abaíra, Mata do Barbado, 1600 m, 2 Jan 1992, *Harley et al. 50631* (K); **Minas Gerais**: Sin. loc, *Asampaio 374* (US); **Paraná**: Serrinha, 14 Oct 1909, *Dusén 8549* (F, F, GH, K, S); Calmon, 12 Mar 1910, *Dusén 9284* (S); Barigui, 890 m, 29 Oct 1914, *Dusén 15756* (F, GH, K); Laranjeiras do Sul, Laranjeiras do Sul, 16 Feb 1963, *Hatschbach 9724* (US); Laranjeiras do Sul, Virmond, 12 Apr 1965, *Hatschbach 12539* (K); General Carneiro, Cab. Iratim, 11 Feb 1966, *Hatschbach et al. 13704* (US); Bituruna, Rio Jangada, 17 Oct 1966, *Hatschbach 14962* (F, US); Tapejara, 26 Aug 1967, *Hatschbach 16991* (F); Mun. Curitiba, Guabirotuba, 14 Dec 1978, *Hatschbach 41897* (F); Novas Tebas, rod. PR-460, proximo do trevo para Nova Tebas, 26 Aug 2001, *Hatschbach et al. 72322* (SI); Curitiba, 900 m, 7 Sep 1914, *Jönsson 894 a* (F, GH, K); São José dos Pinhais, Ponte Sinco, 3 Nov 1972, *Kuniyoshi 3309* (US); just N of Campo Novo, 600 m, 5 Nov 1966, *Lindeman & Haas 2837* (KLaranjeiras do Sul, 16 Nov 1963, *Pereira & Hatschbach 7922* (US); Curitiba, Campina de Siqueira, Rio Baragui, 30 Sep 1967, *Stellfeld 1690* (US); sin. loc, *Tweedie* s.n. (K); **Rio Grande do Sul**: Neu-Württemberg, 450 m, 20 Aug 1904, *Bornmüller 135* (A, G, GH); Centro de Lozer e Recrecão Santa Rita-Farroupilha, 12 Sep 1978, *Bueno 1026* (MA); Marcelino Ramos, estrada prox. Rio Uruguai, 370 m, 6 Oct 1995, *Butzke et al. 7743* (F); Pelotas, I.A. Sul, mato do Horto Botânico do I. Agr, 4 Jun 1954, *Costa Sacco 147* (US); Pelotas, Pedreira Sta. Cecilia, 20 May 1959, *Costa Sacco 1231* (F); São José, Farroupilha, 21 Oct 1984, *Dal Pont 493* (US); Farroupilha, Sao Roque, 25 Aug 1985, *Dal Pont 1091* (US); Flores da Cunha, Linea 60, 29 Jul 1984, *Grazziotin 191* (US); Fazenda Faxinal, Arroio dos Ratos, 31 Oct 1979, *Hagelund 13164* (F); Fazenda Mauqueira das Pedras, Arroio dos Ratos, 25 Sep 1984, *Hagelund 15346* (F); São Marcos, Formigueiro, 800 m, 27 Oct 1999, *Kegler 305* (G, US); Caixas do Sul, São Luiz, 3a Legua, 780 m, 11 Mar 2000, *Kegler 843* (US); Quinta, 5 Nov 1901, *Malme 225* (S); Santo Ângelo, prope Cachoeira, 2 Feb 1892, *Malme 534b* (S); Guaíba, Fazenda São Maximiano, BR-116, km 307, 30 Sep 1990, *Matzenbacher 61* (F); São Jerônimo, Pólo Carboquímico, mata de Ingá, 18 Oct 1982, *Neves 137* (F); Vacaria, 14 km de Vacaria, rumo Bom Jesus, 25 Oct 1963, *Pabst 6320* (F); Pinheiro Machado, Coxilho Pedras Altas, 11 Nov 1976, *Pedersen 11442* (A, K x2, MO); Rio dos Sinos, 8 May 1949, *Rambo 44283* (K, US); São Francisco de Paula, 18 Dec 1949, *Rambo 44854* (G); Vila Manresa, prope Porto Alegre, 18 Sep 1955, *Rambo 57339* (B, S); Canoas, 9 Oct 1897, *Reineck & Czermak 89* (E, GOET); Pantano Grande, 1 Oct 1988, *Rossato 4586* (US); Colônia de Santo Ângelo, Sep 1899, *Schwarzer* s.n. (S); Caixas do Sul, Sta. Justina, 780 m, 27 Nov 1999, *Scur 190* (US); San Pedro, Montenegro, 550 m, 1 Sep 1949, *Sehnem 3843* (B, US); Pessegueiro, Camaquã, 12 Oct 1983, *Sobral, M*., *2418* (F); Mina Volta Grande, Lavras do Sul, 5 Oct 1984, *Sobral 3115* (F); Amaral Ferrador, Encruzilhada do Sul, Sep 1985, *Sobral 4206* (F, K); Esmeralda, E. Aracuní, Jan 1984, *Stehmann 307* (F); Rio Grande, woods of the Uruguay, *Tweedie* s.n. (K); São Francisco de Paula, RS-235, 850 m, 20 Sep 1999, *Wasum 128* (B, MO, US); Flores da Cunha, Linea 100, 29 Jul 1984, *Wasum 232* (US); Caixas do Sul, Estrada São Virgilio, 26 Aug 1984, *Wasum 259* (US); São Roque, Farroupina, 25 Aug 1985, *Wasum 1080* (G, K); Fazenda Taleira, Caçapava do Sul, 21 Sep 1986, *Wasum 2081* (US); Nova Prata, 30 May 1987, *Wasum 3069* (US); Bento Gonçalves, estrada para Guaporé, 750 m, 24 Aug 1998, *Wasum et al. 12747* (US); **Rio de Janeiro**: Petrópolis, 13 Mar 1924, *Bailey & Bailey 1312* (BH); **Santa Catarina**: Marombas, Curitibanos, 900 m, 6 Dec 1962, *Klein 3290* (US); Campos Novos, 1000 m, 29 Oct 1963, *Klein 4209* (US); Lajes, Passo do Socorro, 700 m, 31 Oct 1963, *Klein 4373* (US); Lajes, Passo do Socorro, 700 m, 31 Oct 1963, *Klein 4378* (US); Bom Retiro, Campo dos Padres, Bom Retiro, 1800 m, 20 Dec 1948, *Reitz 3614* (S); Bom Retiro, 950 m, 26 Oct 1957, *Reitz & Klein 5484a* (F, GH, US); São Joaquim, Serra do Oratorio, Bom Jardim, 1400 m, 23 Oct 1958, *Reitz & Klein 7420* (US); São Joaquim, Fazenda de Laranja, Bom Jardim, 1400 m, 13 Dec 1958, *Reitz & Klein 7859* (US); Ponte Alta do Sul, Curitibanos, 900 m, 24 Oct 1962, *Reitz & Klein 13352* (G, K, US); Campos Novos, 1000 m, 20 Dec 1962, *Reitz & Klein 14339* (US); Bom Retiro, Campo dos Padres, between Fazenda Campo dos Padres and Fazenda Santo Antonio, 1450 m, 21 Nov 1956, *Smith & Klein 7796* (K, S, US); Lajes, bank of Rio Canoas, N of Lajes, 800 m, *Smith et al. 8244* (LE, US); Caçador, 8 km north of Caçador, 950 m, 21 Dec 1956, *Smith & Reitz 8954* (US); Porto União, Rio Negro, west of Porto União, 750 m, 4 Feb 1957, *Smith & Klein 10791* (US); Irani, Campo de Irani, 700 m, 13 Oct 1964, *Smith & Reitz 12469* (US); São Miguel d’Oeste, Canela Gaúcha, 8 km northwest of São Miguel d’Oeste, 700 m, 20 Oct 1964, *Smith & Reitz 12754* (GH, US); Xanxerê, Xanxerê, by quarry and Rio Xanerê, 800 m, 16 Dec 1964, *Smith & Klein 14027* (US); **São Paulo**: São Paulo, 15 Feb 1924, *Bailey & Bailey 929* (BH); São Paulo, 11 May 1942, *Bento & Pickel 1192* (US); Apiahy, *Puggiari* s.n. (P); Campinas, Fazenda Santa Eliza, Instituto Agronomico de Campinas, Secção de Floricultura, 7 Jun 1969, *Texeira 228* (A).

**Paraguay**. **Alto Paraná**: Colonia 13 Tujutí, 31 km al N de Hernandarias, 14 Oct 1984, *Brunner et al. 911* (G); Reserva Itabó e Itaipú Binacional, 16 Oct 1984, *Brunner et al. 925* (G); sin. loc, 1910, *Fiebrig 5731* (G, GH, US); Reserva Biológica Limoy, cerca del Río Limoy y embalse de Represa Raipú en el Río Paraná, 15 Oct 1996, *Schinini et al. 31413* (G); **Caazapá**: San Agustín, 16 Apr 1984, *Hahn 2286* (BH); **Canindeyú**: San Estanislao, Yerbales, Sierra de Maracayú, in regione vicine San Estanislao, Aug, *Hassler 4131* (G, GH, K, S); Ipehuí, Sierra de Mbaracayú, Nov 1896, *Hassler 5342* (G); Jejui-mí, a 23 km al E de Ygatimi, rumbo norte, 18 Apr 1996, *Jiménez & Marín 0183* (BM); Jejui-mí, a 23 km al E de Ygatimí, sendero principal, 4 Dec 1996, *Jiménez 1742* (BM); **Cordillera**: Caacupé, Aug 1914, *Chodat 22* (G); Cerros de Tobatí, 14 Jan 1903, *Fiebrig 740* (E, F, G); **Guairá**: Villarrica, Dec 1931, *Jörgensen 3667* (A, F, S, US); Colonia Independencia, Arroyo Guazú, camino a San Gervasio, 26 Mar 1993, *Schinini et al. 27997* (G); Cordillera de Ybytyruzú, road to Cantera Jhú, 8 km S of Coronel Oviedo, 16 Oct 1989, *Zardini 14976* (G); **Itapúa**: después de General Delgado, 11 km antes de Coronel Bogado, 16 Sep 1980, *Fernández-Casas & Molero 3692* (MA); Encarnación, Sep 1915, *Rojas 1392* (G); **Misiones**: San Ignacio, Oct 1914, *Chodat 23* (G); **Paraguarí**: Parque Nacional Ybycuí, 5 Feb 1984, *Hahn 1952* (BH); Sapucai, Dec, *Hassler 1608* (G); Macizo Acahay, 5 Sep 1988, *Zardini & Florentin 7036* (G); **San Pedro**: Colonia Nueva Germania, Trinidad-Asunción, Dec 1916, *Rojas 10475* (SI); Primavera, 14 Jan 1959, *Woolston 1056* (K, S, US).

**Uruguay**. **Canelones**: Puerto Jackson, Río Santa Lucia, 31 Oct 1948, *Anonymous B-5209* (MA); **Colonia**: Riachuelo, 11 Oct 1936, *Cabrera 3845* (, F); Río San Juan, 2 Nov 1962, *Torres & Ancibor 1084* (MA); **Durazno**: Durazno, sobre el Río Negro, Dec 1934, *Legrand 331* (F); **Florida**: Arroyo Maldonado, 29 Sep 1926, *Herter 19182* (S); **Rivera**: Cuñapiru, 182 m, 1928, *Wright* s.n. (BM); **Rocha**: Castillos, 10 m, 30 Oct 1914, *Herter 1516b* (G, GH); Castillos, 50 m, 31 Nov 1931, *Herter 1516c* (F, GOET); **Soriano**: John Jackson, Estancia Santa Elena, Nov 1942, *Gallinal H. et al. PE-4500* (GH); **Tacuarembó**: Gruta de los Cuervos, 17 Dec 1907, *Berro 4797* (K).

##### Specimens examined [introduced/cultivated or naturalised]:

**China**. Hong Kong, Botanic Garden, May 1879, *Ford* s.n. (K).

**Colombia**. **Antioquia**: Rionegro, Dec 1936, *Brother Daniel* s.n. (F); San Antonio, Nov 1936, *Brother Daniel 969* (GH); **Cundinamarca**: Bogotá, Ciudad Universitaria, 2600 m, 8 Sep 1942, *Cuatrecasas 13641* (F, GH, US); Bogotá, hills 1 km S of Suba, 13 km N of center of Bogotá, 2700 m, 12 Jun 1944, *Fosberg 21986* (US); La Palma, Murca valley, Cordillera de Heliconia, 10 kilometers south east of Gachalá, 2200 m, 29 Sep 1944, *Grant 10300* (US); Páramo de Retiro, Macizo de Bogotá, 27 Jul 1942, *Schultes 3173* (US); **Santander**: Salazar, Jan 1880, *Kalbreyer* s.n. (SI).

**Costa Rica**. Sin. loc., 1949, *León* s.n. (F).

**Ecuador**. **Bolívar**: Atio de Telimbela, descenso de Cordillera Occidental, 1500 m, 18 Nov 1943, *Acosta-Solís 6867* (F); **Chimborazo**: Huigra, mostly on Hacienda Licay, 18 Aug 1918, *Rose & Rose 22200* (GH, US); **Pichincha**: La Magdalena, en el jardin de las H.H, 2800 m, 25 Dec 1927, *Firmin 304* (US); Cantón Quito, Quito, 6 May 1920, *Holmgren 577* (A, US); **Tungurahua**: Highway Ambato-Baños, town of Pelileo, 2700 m, 9 Aug 2005, *Clark et al. 9098* (QCNE, US).

**France**. Montpellier, “Hort. Monspel.”, 1851, *Anonymous* s.n. (B); Nantes, Jardin Brusseau, de Bresil, 7 Jun 1890, *Gadeceau* s.n. (BM).

**Germany**. Breslau, Königl. Botanischer Garten, 120 m, 27 Aug 1910, *Baenitz 30303* (B).

**Greece**. **Achaias**: Patron, Ano Arachovitika, by Cape Drepanono, ca. 15 km NE of Patras, 20 m, 17 Sep 1996, *Nielsen 11614* (B).

**India**. **Assam**: Mawphlang, 1829 m, 31 Aug 1952, *Koelz 31248* (L); **Kodaikanal region**: Pulney Hills, 304 m, *Anglade* s.n. (A); **Madras**: Loyola College, Jul 1958, *Anonymous* s.n. (LE); **Tamil Nadu**: Ooty, 2134 m, 11 Jan 1962, *Krishnappa 161* (K); Dindigul, Kodaikanal taluk, Bombay shola, 2100 m, 9 Oct 1989, *Matthew et al. 42889* (K).

**Kenya**. **Laikipia**: Nanyuki, FTEA region: K4 Maringo Gawen, 2134 m, 22 Sep 1952, *Starseuslli 108* (EA); **Nairobi**: Nairobi National Museum Grounds, FTEA region K4, 1650 m, 6 Dec 1989, *Querie & Wege 81* (EA); **Nakuru**: Njoro, FTEA region K3, Egerton University, 2256 m, 25 Jun 1976, *Gitonga 285* (EA); **Nyandarua**: South Kinangop, FTEA region K4, 2606 m, 11 Dec 1960, *Verdcourt 3026* (EA).

**Mexico**. **Distrito Federal**: Coyoacán, Quevedo‘s garden wall, 27 Oct 1930, *Reddick 179* (BH); **Hidalgo**: Tlanchinol, 6 km al sur del Tlanchinol, hacia Apantlaso, 1600 m, 7 Nov 1980, *Hernández M. 5331* (MEX); Cuernavaca, 1524 m, 25 Jun 1898, *Pringle 6901* (BM, F, G, GOET, LE); **Puebla**: San Ignacio, 15 Jul 1912, *Arsène 10014* (B, MEXU); **Veracruz**: Tonayán, 1700 m, 29 Dec 1975, *Avendaño Reyes 51* (F); Tonayán, entre Monte Real y Pocitos, 1900 m, Nov 1979, *Chazaro B. 1251* (F); Cóatepec, 4 Dec 1981, *Flügel & Geiseler 6075* (B); Huayacocotla, 2100 m, 22 Jul 1982, *Nee & Diggs 25175* (F).

**Portugal**. **Alto Alentejo**: Elvas, E.M.P, 16 Oct 1971, *Gueua 839* (MA); **Madeira**: Cabo Girão, 550 m, 29 May 1954, *Malato-Beliz 726* (MA).

**Russian Federation**. St. Petersburg Botanic Garden, *Anonymous* s.n. (LE).

**Spain**. Madrid, Jardin Botánico, 20 Sep 1978, *Barra* s.n. (MA); **A Coruña**: Ames, Lens, Pazo de Lens, 190 m, 16 Sep 1988, *Dacal* s.n. (MA); **Cadiz**: valle de la Miel, sentier pres du moulin, 150 m, 22 Apr 1987, *Charpin 20976* (MA); **Canary Islands**: Tenerife, La Orotava, 400 m, 20 Apr 1966, *Faber 116* (B); Tenerife, Anaga, westlich von Caserio la Cumbre, 600 m, 14 Dec 1985, *Hagemann 2883 B* (B); La Palma, El Paso, Feb 1958, *Stoffer* s.n. (L); **Cantabria**: Santillana del Mar, 11 Nov 1983, *Loriente* s.n. (MA); **Catalunya**: Barcelona, 19 Jul 1922, *Sennen 4895* (BM, MA, SI); **Malaga**: Torrox, 80 m, 19 Jun 2000, *Elorza* s.n. (MA); **Pontevedra**: Coiro, Cangas de Morrazo, 30 Oct 1969, *Castroviejo* s.n. (MA); Bueu, Seixo, Piñeiro, 8 Aug 1985, *Castroviejo 9648* (MA); Figueirido, 8 Apr 1948, *Rodríguez* s.n. (MA).

**Sri Lanka**. **Central**: Nuwara Eliya, 1880 m, 2 Feb 1968, *Comanor 904* (K); Nuwara Eliya, Ohiya, 15 May 1975, *Cramer 4471* (K); Nuwara Eliya, 2000 m, 28 Jan 1974, *Jayasuriya et al. 1458* (US).

**United Kingdom**. Royal Botanic Gardens Kew, 1848, *Houston* s.n. (BM); Chiswick, Oct 1976, *Whitefoord* s.n. (BM).

**United States of America**. **California**: Alameda County, Berkeley, 25 Feb 1980, *Fosberg 59460* (US); Berkeley, 27 May 1881, *Krause & Krause* s.n. (CORD); Oakland, 19 Jul 1877, *Savatier* s.n. (P).

#### 22. Solanum leiophyllum

Benth., Pl. Hartw. 146. 1844

http://species-id.net/wiki/Solanum_leiophyllum

Figure 57

Solanum benthamii Dunal, Prodr. [A.P. de Candolle] 13(1): 101. 1852, nom. nov. superfl. Type: Based on *Solanum leiophyllum* Benth.

##### Type. 

Ecuador. Loja: Chuquiribamba, *K.T. Hartweg 812* (holotype: K [K00585549]; isotypes: BM [BM000815948], F (F0073110, frag.), G [G00070187], K [K000545697], LD [LD-1088980], NY [NY00172061], P [P00324725**,** Morton neg. 8152], W [W-0022642, [F neg. 33049]).

##### Description.

Shrubs to 3 m tall, the stems often scandent. Stems and leaves densely pubescent with loosely branching, lax, dendritic trichomes, these sometimes mixed with simple trichomes, later deciduous; leaf scars prominently raised, the stem strongly winged from the decurrent leaf bases; new growth densely pubescent with long, loose, dendritic trichomes to 2 mm long. Bark of older stems grey-brown, glabrate or sparsely pubescent with long dendritic or simple trichomes. Sympodial units plurifoliate, branching monochasial. Leaves simple, 1–4.5 cm long, 0.8–3 cm wide, broadly elliptic or ovate, shiny, fleshy, and coriaceous, the adaxial surfaces of the blades shiny and glabrous, the abaxial surfaces loosely pubescent with long dendritic trichomes along the veins and lamina, with some trichomes on the revolute margins; primary veins 3–6 pairs, strongly impressed above; base broadly acute, winged on to the petiole; margins entire, strongly revolute; apex broadly acute; petiole 1–2 mm long, winged from the decurrent leaf bases onto the stem. Inflorescences terminal, appearing lateral from overtopping shoot growth, 1.5–7 cm long, flattened globose in outline, branching 1–4 times, with 8–10(-30) flowers, the axis densely pubescent with loose dendritic trichomes; peduncle 0.8–2 cm long; pedicels 1.1–1.6 cm long, tapering from a basal diameter of 0.5–1 mm to an apical diameter of 1–1.5 mm, nodding at anthesis, sparsely pubescent with long simple and/or dendritic trichomes, articulated at the base and inserted in a sleeve ca. 1 mm long; pedicel scars spaced 1–2 mm apart. Buds ellipsoid, the corolla strongly exserted from the calyx tube. Flowers all perfect, 5-merous, nodding at anthesis. Calyx tube conical, 1.5–2 mm long, the lobes long-triangular, 2–2.5 mm long, with scattered dendritic trichomes abaxially, these denser at the tips of the lobes, sparsely pubescent adaxially with simple trichomes along the veins. Corolla violet or a darker purple, 1.8–2 cm in diameter, stellate to slightly campanulate, lobed 3/4 of the way to the base, the lobes 8–10 mm long, 4–5 mm wide, planar at anthesis, densely pubescent abaxially with tiny dendritic trichomes, these denser at the tips of the lobes, glabrous adaxially. Filament tube absent; free portion of the filaments ca. 1 mm long, glabrous; anthers 5.5–6 mm long, 1.5–2 mm wide, loosely connivent, the bases caudate, poricidal at the tips, the pores becoming slit-like with age. Ovary glabrous; style 0.8–1 cm long, glabrous; stigma minutely bilobed, the surface minutely papillose. Fruit a globose berry, 0.8–1.3 cm in diameter, black with thin pericarp; fruiting pedicels 1.8–2 cm long, ca. 1 mm in diameter at the base woody, deflexed. Seeds ca. 7 per fruit, 3.5–4 mm long, 3–4 mm wide, reddish-brown, flattened lenticular, the surfaces minutely pitted. Chromosome number: not known.

**Figure 57.**
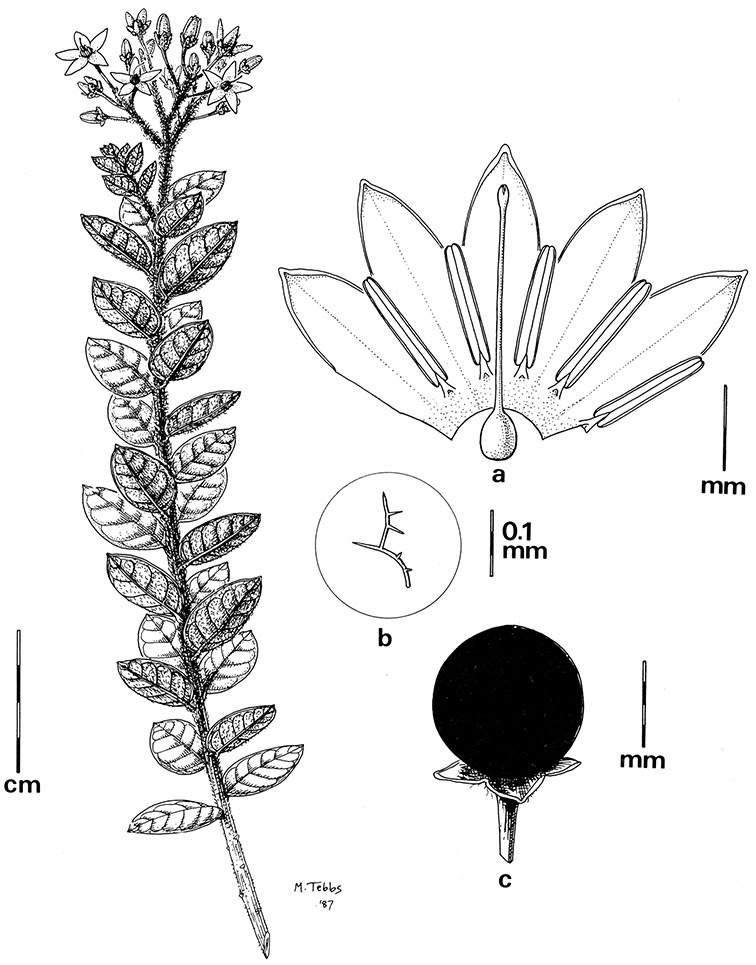
*Solanum leiophyllum* Benth. (**A–C** drawn from *Hartweg 812*). Reproduced from Knapp (1989) with permission of the Natural History Museum Botany Library.

##### Distribution

**Figure 58.**
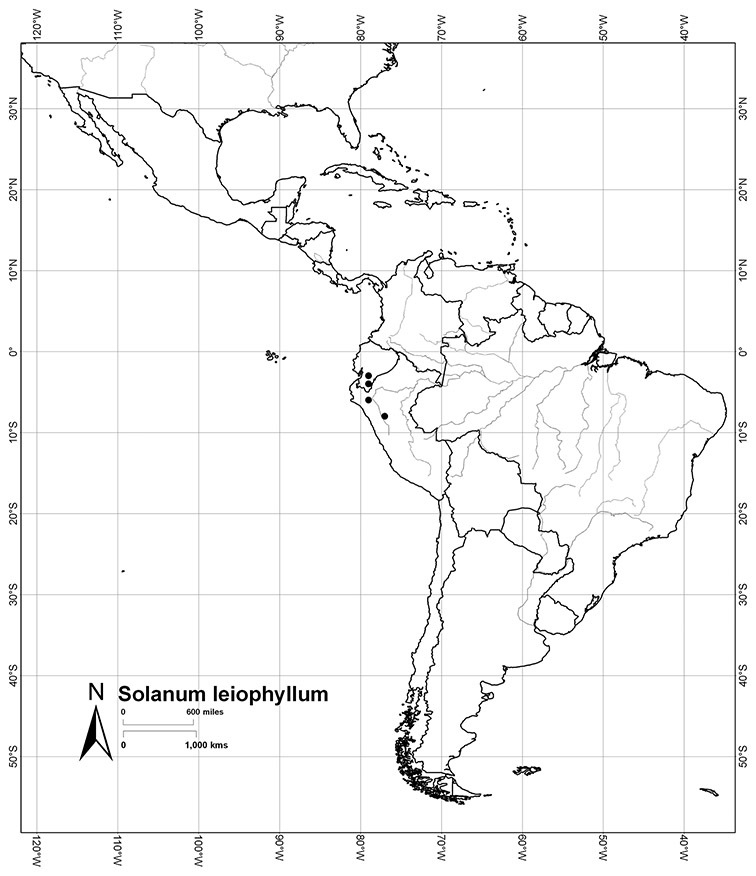
Distribution of *Solanum leiophyllum* Benth.

(Figure 58). Southern Ecuador and northern Peru; 2000–4000 m.

##### Ecology.

In open areas in *Polylepis* Ruiz & Pav. (Rosaceae) cloud forest and in grass páramo above timberline.

##### Conservation status.

Near Threatened (NT); EOO <45,000 km^2^ (NT) and AOO >10,000 km^2^ (LC). See Moat (2007) for explanation of measurements.

##### Discussion.

Knapp (1989) only knew *Solanum leiophyllum* from the type collection. Since then, intensive collecting in southern Ecuador by botanists from the University of Loja (LOJA) has greatly increased out knowledge of the distribution of this endemic species. It is still relatively rare geographically, but can be locally common where it is found. Specimens from Peru (*León et al. 5562*, *5581*) have long simple trichomes mixed with the loose dendritic trichomes typical of Ecuadorian populations of *Solanum leiophyllum*. The shiny leaf uppersides, nodding purple flowers and black fruits are the same.

##### Specimens examined.

**Ecuador**. **Azuay**: Road Gualaceo-Limón (General Plaza Gutiérrez), km. 25.2, side road at the pass to antennas and military post 1.5 km, 3540 m, 12 Jan 2000, *Jørgensen et al. 1863* (BM); Eastern Cordillera, between Ona and the Río Yacuambi, 3000 m, 10 Sep 1945, *Prieto 309* (MO); Cantón Gualaceo, Carretera Cuenca-Gualaceo-Limón Indanza, km 23-25, 3500 m, 10 Jul 1995, *Ávarez & Tirado 1480* (BM); **Loja**: Loma del Oro, , 2800 m, 4 Aug 1986, *Jaramillo et al. 8810* (AAU); road Loja-Cuenca, km 50, track to Fierro Urco, km 11, 3600 m, 25 Oct 1996, *Lewis & Lozano 2726* (K).

**Peru**. **Cajamarca**: Jaén, Paramillo de Pomahuaca, antes del pajonal, 3200 m, 8 Nov 1999, *Díaz & Campos 10908* (BM, MO); **San Martín**: Mariscal Cáceres, Laguna Verde, entre el campamento y el Concejo de Laguna Verde, 3760 m, 21 Jun 2010, *León & Ullilen 5562* (USM); Mariscal Cáceres, Laguna Verde, alrededores, Dist. Huicungo, 3599 m, 23 Jun 2010, *León et al. 5581* (USM); Mariscal Cáceres, Chochos, 3350 m, 24 Nov 1985, *Young 2407* (MOL).

#### 23. Solanum luculentum

C.V.Morton ex S.Knapp, PLoS ONE 5(5): e10502. 2010

http://species-id.net/wiki/Solanum_luculentum

Figure 59

##### Type. 

Colombia. Antioquia: Mpio. Sonsón, Vereda Manzanares, Finca La Montañita, Cerro de la Vieja, páramo de Sonsón, 2600–3100m, 11 Jan 1995, *J. Betancur & S.P. Churchill 5912* (holotype: COL [COL000057871]; isotype: HUA).

##### Description.

Woody vines or lianas, occasionally apparently epiphytic, to 6 m long; stems glabrous and shiny; new growth almost completely glabrous, with a few (1 or 2), minute, golden multiseriate trichomes < 0.5 mm long; bark of older stems pale tan and markedly exfoliating (“shreddy” fide *Nee & Callejas 32546*). Sympodial units plurifoliate. Leaves simple, 2–11 cm long, l–5 cm wide, elliptic to narrowly elliptic, coriaceous, the upper surfaces glabrous and shiny, the veins not apparent, the lower surfaces glabrous, the veins yellowish cream; primary veins 5–7 pairs, prominent below, obscure above; base cuneate to acute to truncate and occasionally slightly cordate; margins entire, strongly revolute in both dry and live (fide *Steyermark et al. 100777*) plants; apex acute or occasionally long acuminate; petioles 0.7–3 cm long, glabrous or with a few scattered glandular papillae, wrinkled when dry, twining. Inflorescences terminal, 3–11 cm long, more or less ellipsoid in outline, many times branched, with 20–50 flowers, glabrous; peduncle 0.5–2 cm long, the branches very near the base; pedicels 1.2–1.5 cm long, slender, ca. 0.5 mm in diameter at the base, ca. 1 mm in diameter at the apex, glabrous, apparently somewhat erect at anthesis, articulated just above the base, leaving a prominent swelling on the axis; pedicel scars irregular spaced 2–10 mm apart. Buds globose, becoming ellipsoid to turbinate, the corolla strongly exserted from the calyx tube early in expansion. Flowers heterostylous, the plants probably dioecious, with long-styled and short-styled flowers on different plants but of similar overall morphology. Calyx tube 1.5–2 mm long, conical, the lobes 0.5–1 mm, broadly deltate, glabrous with the tips minutely papillate. Corolla 1.5–1.7 cm in diameter, white or occasionally tinged with lavender, stellate, lobed 2/3 to 3/4 of the way to the base, the lobes 6–7 mm long, 3–4 mm wide, planar at anthesis, mostly glabrous on both surfaces but densely papillose on tips and margins with golden simple trichomes, these occasionally extending along the midvein of the abaxial surface. Filament tube minute, the free portion of the filaments ca. 1 mm long, glabrous; anthers of long-styled flowers ca. 4 mm long, 1 mm wide, occasionally slightly shrivelled, in short-styled flowers ca. 5 mm long, 1.5 mm wide, ellipsoid to pointed ellipsoid, poridical at the tips, the pores lengthening to slits with age. Ovary glabrous, vestigial in short-styled flowers; style in long-styled flowers 5–6 mm long, exserted beyond the anthers, glabrous, in short-styled flowers 2.5–3 mm long, included in the anther tube, glabrous; stigma clavate, the surface densely papillose in long-styled flowers. Fruit a globose berry, to 2 cm in diameter, green (immature?), the pericarp quite thin but not markedly shiny; fruiting pedicels 1.5–2 cm long, ca. 2–3 mm in diameter at the apex, woody and nodding. Seeds 10–12 per berry, 6–8 mm long, 4–6 mm wide, flattened reniform, pale tan, the surfaces minutely pitted, the testal cells rectangular at the margins, deeply sinuate with rib-like thickenings on the lateral walls in the seed center. Chromosome number: not known.

**Figure 59.**
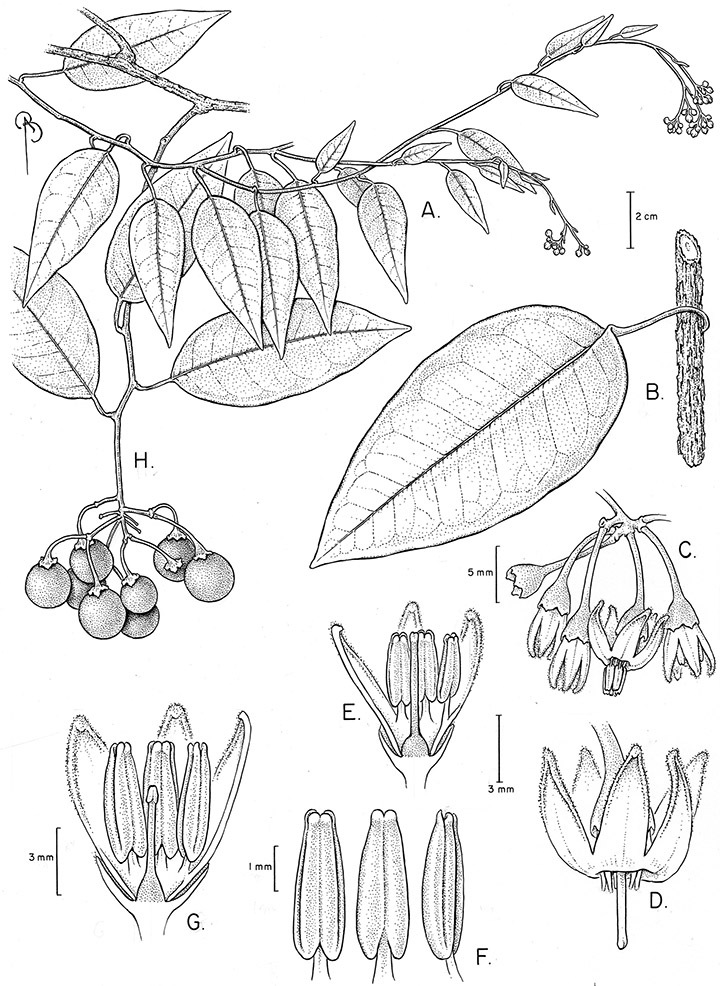
*Solanum luculentum* S.Knapp. (**A–B** drawn from *Nee & Callejas 32546* **C–F** from *Steyermark et al. 127855*, G from *Steyermark & Dunsterville 100777* **H** from *Killip & Smith 15952*). Originally published in Knapp (2010). Illustration by Bobbi Angell.

##### Distribution

**Figure 60.**
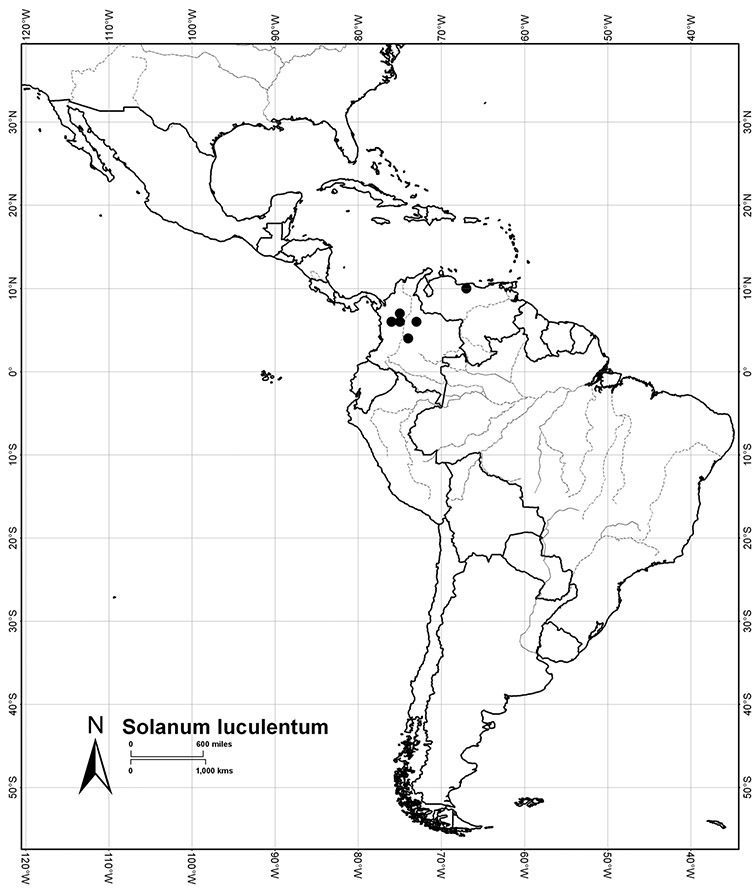
Distribution of *Solanum luculentum* S.Knapp.

(Figure 60). *Solanum luculentum* occurs in the Andes of Colombia (Depts. Antioquia, Cundinamarca and Nariño) and Venezuela (from the Colombian border to the Federal District around Caracas); 1500 to 3200 m.

##### Ecology.

Cloud forests, forest margins.

##### Conservation status.

Least Concern (LC); EOO >100,000 km^2^ (LC) and AOO >10,000 km^2^ (LC). See Moat (2007) for explanation of measurements.

##### Discussion.

*Solanum luculentum* was identified as a new species by the Solanaceae specialist Conrad V. Morton in the 1940s on herbarium annotation slips on specimens in US (*Archer 1153*, *1227*), but his appropriate name was never published. I decided to use it, as it perfectly describes the distinguishing characteristic of this species; its coriaceous, lustrous and shining leaves. *Solanum luculentum* has long been confused with *Solanum dichroandrum*, with which it is sympatric, but differs from *Solanum dichroandrum* in its completely glabrous leaves and inflorescences, revolute leaf margins and heterostylous flowers.

Specimens of *Solanum luculentum*, to my knowledge, either bear short-styled flowers and no fruits or long-styled flowers and fruits; this may be indicative of a dioecious species of *Solanum*, one of very few outside the Leptostemonum clade (see Knapp et al. 1998; Martine et al. 2009), and the first record for this breeding system in the Dulcamaroid clade. Field confirmation of the breeding system of *Solanum luculentum* will be interesting; pollen of this species has not yet been examined to ascertain if it is inaperturate, as is pollen of other dioecious solanums (Knapp et al. 1998) or if the flower morphology is related to plant age.

##### Specimens examined.

**Colombia**. **Antioquia**: Caldas, Finca La Zarza, Vereda La Corrala, al lado del camino al la cascada, 2500 m, 21 Sep 1987, *Albert de Escobar et al. 7939* (MO); Santa Elena, 1500 m, 28 Dec 1930, *Archer 1153* (US); Santa Elena, 1500 m, 1 Jan 1931, *Archer 1227* (US); La Ceja, en los alrededores, 2500 m, 21 Aug 1948, *Barkley & Johnson 264* (US); Campamento, Vereda El Mango, 6 km NO de Campamento de la via a la mina de LAs Brisas, 1820 m, 8 Sep 1989, *Callejas et al. 8339* (US); Monte del Diablo, (La Ceja), 21 Jul 1944, *Brother Daniel 3281* (US); Boquerón, near Medellín, 2680 m, *Brothers Daniel & Arsènio 3486* (US); San José de Cuerquía, camino del páramo, 31 Jul 1958, *Garganta 2167* (US); midway between Medellín and Río Negro, 2500 m, 8 Jul 1986, *Nee & Callejas 32456* (US); Salgar, km 15 of road Salgar-Hacienda El Dauro (Dpto. Chocó), 2380 m, 14 Mar 1987, *Zarucchi & Echeverry 4753* (K); Jardín, Alto de Ventanas, km 20 of road Jardín-Riosucio (Dept. Caldas), ca. 15 km SSE of Jardín, 2700 m, 29 Oct 1988, *Zarucchi et al. 6928* (K, US); **Boyacá**: Cordillera Oriental, near Laguna Seca in valley of Río de los Pajaros, 2650 m, 26 Aug 1957, *Grubb et al. 737 a* (K); **Cundinamarca**: San Miguel, S of Sibaté on road to Fusagasugá, between km 35 and 36, 2600 m, 12 May 1972, *Barclay et al. 3404* (US); San Miguel, carretera a Fusagasugá, 2800 m, 9 May 1949, *García-Barriga 13335* (US); **Santander**: Las Vegas, in vicinity, 2600 m, 21 Dec 1926, *Killip & Smith 15952* (A, GH, US).

**Venezuela**. **Aragua**: Colonia Tovar, 1856, *Fendler 2099* (G, GOET, K, MO); Colonia Tovar, 4 km SW by air, on road to Capachal 2 km east from road between Colonia Tovar and La Victoria, 1600 m, 7 Apr 1982, *Liesner & Medina 13496* (MO); **Distrito Federal**: Libertador, a lo largo del camino Costa de Maya, noroeste de la Colonia Tovar, 3-5 kms desde la carretera principal La Victoria-Colonia Tovar, 2100 m, 9 Dec 1982, *Steyermark et al. 127855* (MO); **Táchira**: cabeceras del Río Quinimari, entre el pié del peñasco de la Peña de Pata de Judio (debajo del páramo del Judio), y el pié del salto de Chorrejón de la Mota de la Peña de Ventana, arriba de Las Copas, 18-20 kms al sur de San Vicente de la Revancha, 32-35 kms al sur de Alquitrana, suroeste de Santa Ana, 2500 m, 12 Jan 1968, *Steyermark et al. 100777* (F, US).

#### 24. Solanum lyratum

Thunb., Fl. Jap. 92. 1784

http://species-id.net/wiki/Solanum_lyratum

Figure 61

Solanum lyratum Thunb. ex Murray, Syst. Veg., ed. 14: 224. 1784. Type: Based on *Solanum lyratum* Thunb.
Solanum dichotomum Lour., Fl. Cochinch. 129. 1790. Type: China. “Cantone Sinarum” (no specimens cited).
Solanum dulcamara L. var. *pubescens* Blume, Bijdr. 698. 1825. Type: Based on *Solanum lyratum* Thunb.
Solanum dulcamara L. var. *chinense* Dunal, Prodr. [A.P. de Candolle] 13(1): 79. 1852. Type: China. “Kianang Prov.”, *L. Macartney* s.n. (lectotype, designated here: BM [BM000942424]).
Solanum dulcamara L. var. *lyratum* (Thunb.) Seibold & Zucc., Observ. Bot. 4: 317. 1915. Type: Based on *Solanum lyratum* Thunb.
Solanum septemlobum Bunge var. *indutum* Hand.-Mazz., Oesterr. Bot. Z. 83: 234. 1934. Type: China. S-Schansi [Shanxi], Sunnandschen, 23 Jul 1916, *E. Licent 2286* (holotype: W [W-1933/0006499]; isotype: K [K000658180], BM [BM000942501]).
Solanum lyratum Thunb. var. *filamentaceum* Hayashi, J. Geobot. 22: 4. 1974. Type: Japan. Hondo: prov. Musashi, Mt. Takao, 23 Sep 1973, *R. Mineo* s.n. (holotype: TNS [originally cited from TUAT], not located).
Solanum cathayanum C.Y.Wu & S.C.Huang, Fl. Reipubl. Popul. Sin. 67(1): 84. 1978. Type: Based on *Solanum dulcamara* L. var. *chinense* Dunal
Solanum kayamae T.Yamaz., J. Jap. Bot. 68: 339. 1993. Type: Japan. Ryuku: Okinawa Island, Mabuni, 1 Dec 1992, *K. Kayama 6658* (holotype: TI [n.v.]; isotype: BM [BM000647002]).
Solanum lyratum Thunb. forma *purpuratum* Konta & Katsuy., Bull. Natl. Sci. Mus., Tokyo, B 31(1): 23. 2005. Type: Japan. Honshu: Shizuoka, Suzaki, Shimoda City, Suzaki Peninsula, ca. 40 m, 2 Sep 2003, *F. Konta 23381-a* (holotype: TNS [photograph]; isotype: TNS [n.v.]).

##### Type.

Japan. Nagasaki, *C. Thunberg* s.n. (holotype: UPS).

##### Description.

Sprawling shrub or herbaceous vine, woody at the base. Stems flexuous, not winged, sparsely to densely pubescent with translucent, glandular, simple uniseriate trichomes to 4 mm long, with 4–6 cells, the gland 1-celled, these overtopping shorter glandular simple trichomes ca. 0.5 mm long, the trichomes weak and tangled; new growth densely pubescent with trichomes like those of the stems. Bark of older stems pale yellowish tan, glabrescent, the longer trichomes usually breaking off and only the shorter ones remaining. Sympodial units plurifoliate. Leaves simple or pinnatifid, usually only with 2 lyrate lobes at the base, 2–6(-9) cm long, 0.5–5(-7) cm wide, cordate to lyrate, widest in the basal third, thin and membranous, both surfaces uniformly pubescent with weak, translucent simple uniseriate trichomes to 4 mm long, these usually glandular like those of the stems; primary veins 5–7 pairs, usually yellowish in dry material; base cordate, occasionally truncate, not decurrent on the petiole; margins entire or lobed, the lobes usually only a pair at the base, these sometimes completely divided and the leaf apparently pinnate, occasionally with up to 4 pairs of lobes; apex acuminate or acute; petiole 1–3 cm long, pubescent like the stems, twining. Inflorescences terminal or lateral, 2.5–10 cm long, open and many times branched, with 10–40+ flowers, usually only a few open at a time, pubescent with simple uniseriate glandular trichomes like those of the stems; peduncle 2–6 cm long; pedicels 7–11 mm long, ca. 0.5 mm in diameter at the base, ca. 1 mm in diameter at the apex, slender and spreading, glabrous or short-glandular pubescent, articulated at the base in a small sleeve 1–2 mm long; pedicel scars irregularly spaced 2–9 mm apart, more congested distally. Buds ellipsoid, the corolla strongly exserted from the calyx tube before anthesis. Flowers all perfect, 5-merous. Calyx tube 1–1.5 mm long, conical, the lobes 0–1 mm long, deltate or mere enations on the calyx rim, glabrous, papillate on the tips. Corolla 8–13 mm in diameter, white to pale lavender, with green and white spots at the base of each lobe, stellate, lobed 3/4 of the way to the base, the lobes 3–6 mm long, 2–3 mm wide, strongly reflexed at anthesis, the tips and margins densely papillose. Filament tube minute, the free portion of the filaments 1–1.5 mm long, glabrous; anthers 3–3.5 mm long, 1–1.5 mm wide, ellipsoid, loosely connivent, often dark blue or black, the base sagittate, poricidal at the tips, the pores lengthening to slits with age. Ovary glabrous; style 5–7 mm long, glabrous; stigma capitate, the surface minutely papillate. Fruit a globose berry, ca. 1 cm in diameter, bright red, translucent and shiny when ripe, the juice staining scarlet; fruiting pedicels 1–1.5 cm long, ca. 0.75 mm in diameter at the base, spreading. Seeds >30 per berry, ca. 2.5 mm long, ca. 1.5 mm wide, flattened reniform, pale yellowish tan, the surfaces minutely pitted, when mature the seed apparently hairy from the elongate lateral testal cell walls, these to 0.5 mm. Chromosome number: not known.

**Figure 61.**
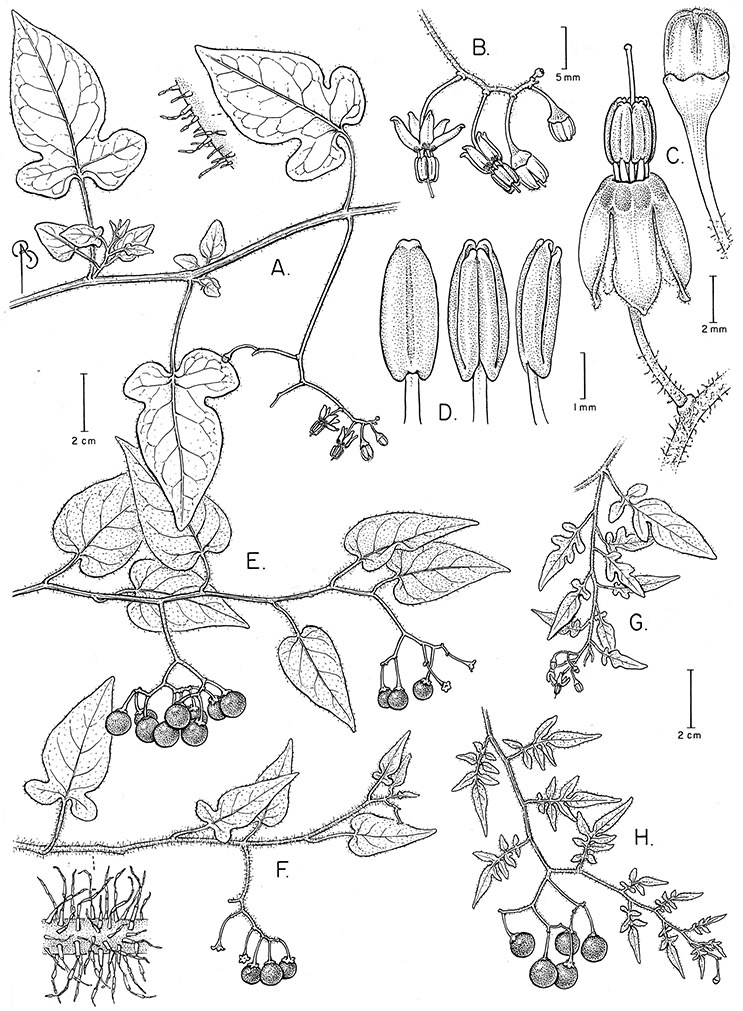
*Solanum lyratum* Thunb. (**A–D** drawn from *Smith 6432* and *Knapp 10142* **E–G** drawn from *Peixing Tan 57179* **G** drawn from *Smith 2265* **H** drawn from *Junsheng Ying 0907*). Illustration by Bobbi Angell.

##### Distribution

**Figure 62.**
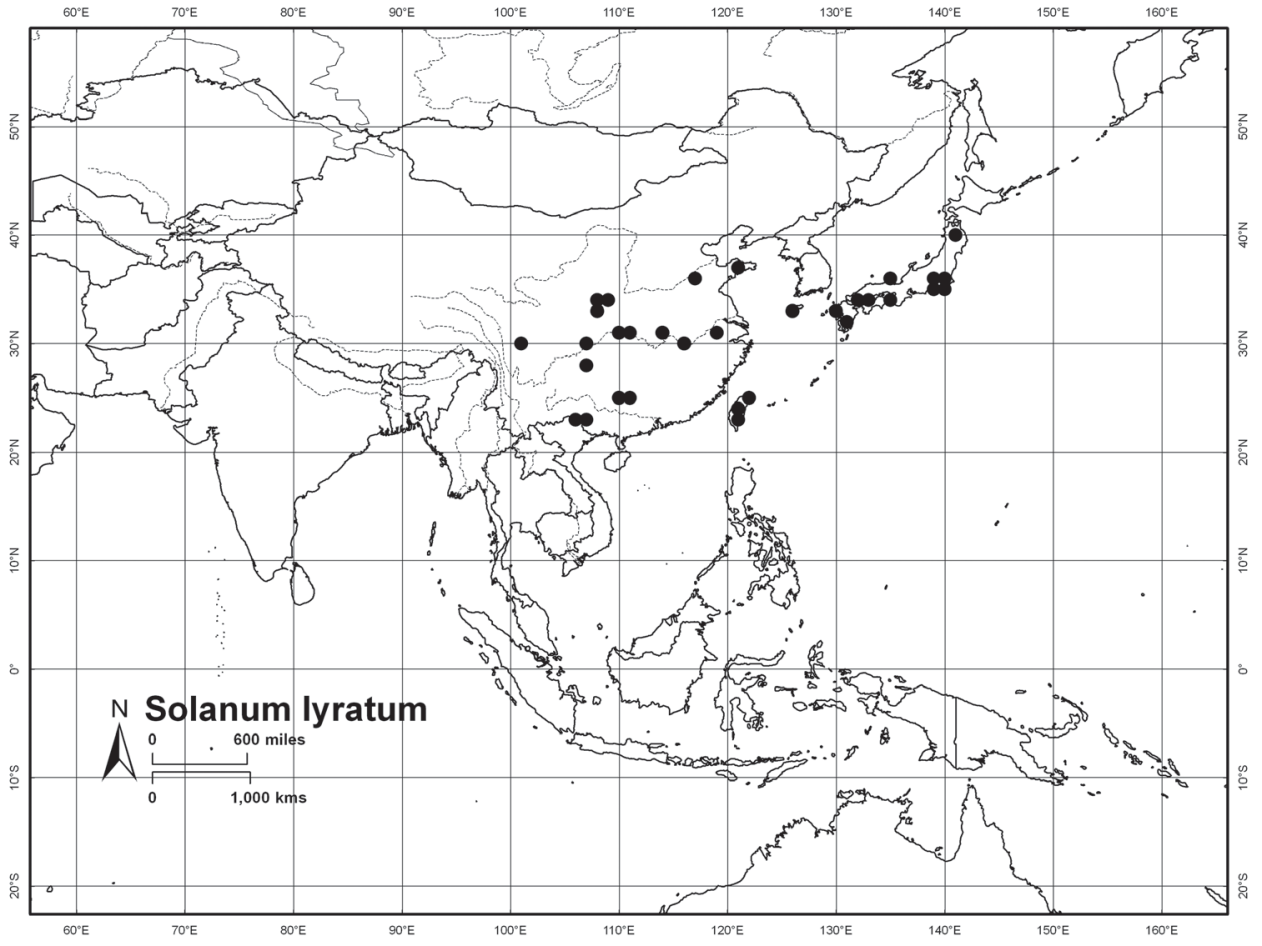
Distribution of *Solanum lyratum* Thunb.

(Figure 62). *Solanum lyratum* occurs in a wide variety of habitats in China, Japan and north Vietnam, from sea level to 1500 m.

##### Ecology.

Usually growing in secondary situations along roadsides and in waste ground; often in urban areas.

##### Common names:

China: bai ying (Zhang et al. 1994); Japan: murasaki-hiyodorijogo (Konta et al. 2007).

##### Conservation status.

Least Concern (LC); EOO >100,000 km^2^ (LC) and AOO >10,000 km^2^ (LC). See Moat (2007) for explanation of measurements.

##### Discussion.

Like the other temperate zone species in the Dulcamaroid clade (*Solanum dulcamara*, *Solanum pittosporifolium*), *Solanum lyratum* is widely distributed, somewhat weedy and grows in many different habitats. It is sympatric with and very similar to *Solanum pittosporifolium*; the species can be differentiated by leaf pubescence. *Solanum lyratum* always has long glandular trichomes on all vegetative parts, especially the new growth, while *Solanum pittosporifolium* is glabrous or has very short uniseriate trichomes on the leaves and young stems. Leaf shape in *Solanum lyratum* is extremely variable; most specimens have lyrate leaf bases and ternate leaves with a very large central lobe (see Figure 2), but simple leaves are common and some specimens (e.g., *Licent 2286*, the type of *Solanum septemlobum* var. *indutum*) have pinnatifid leaves with 5-7 pairs of leaflets. Pubescence, however, is very consistent and is an excellent diagnostic character for *Solanum lyratum*. Flower color also varies from dark purple to white, but there are always green spots at the base of the corolla lobes. Although most *Solanum* species have bright yellow anthers, *Solanum lyratum* occasionally has blackish purple anthers; it is not clear whether this is genetic or is due to an environmental effect. Anthers in *Solanum lyratum* are never tapered like those of *Solanum dulcamara*; due to pubescence differences these two species are not difficult to distinguish (but see *Solanum pittosporifolium*).

Two specimens at “herb. Banks” (*Macartney* s.n. and *Staunton* s.n. both now at BM) were cited by Dunal in his description of *Solanum dulcamara* var. *chinense*. The Macartney collection (BM000942424) has here been selected as the lectotype in order to preserve usage, as it is a specimen of *Solanum lyratum*; the Staunton sheet (BM000942515) is *Solanum septemlobum*.

##### Specimens examined.

**China**. **Anhui**: Wangshan, *Ching 8698* (US); Hsi, 3 Jun 1935, *Migo* s.n. (A); Huoshan County, Fozlingshuiku, 12 Sep 1985, *South Team 1429* (MO); Baijianshan, Xigou, Xuancheng, 400 m, 2 Nov 1998, *Wang E-8* (MO); **Chongqing**: Chengkou County, 600 m, 31 Aug 1958, *Dai 102263* (MO); Chengkou County, Qiganshan, 1750 m, 6 Sep 1958, *Dai 102476* (MO); Chengkou County, Shuanghekou, Baichishan, 1100 m, 17 Sep 1958, *Dai 102779* (MO); **Fujian**: Huxingshan, 2 May 1959, *Huang 190030* (MO); Hinghwa and vicinity, 1 Sep 1926, *Lin Pi 6434* (A); O-Boih Village, Hinghwa and vicinity, 11 Sep 1926, *Lin Pi 6549* (A); Shaowu, Wong To plain, 18 Aug 1926, *Niao 9475* (A); Mount Wuyi, road from Sangang to Hongdu, Wuyi Mt, 700 m, 2 Aug 1980, *Wuyi Shan Team 80-0529* (MO); **Gansu**: Wudu County, Weizigou forest district, 2250 m, 27 Aug 1989, *Ying et al. 0907* (MO); **Guangdong**: Ruyuan County, Tianjingshan Forest District, 21 Sep 1974, *Anonymous 93909* (MO); Ruyuan County, Baishuizhai, Tixia village, 14 Aug 1935, *Chen 10764* (MO); Lokchong, Kwangtung: Lokchong District, 25 Nov 1930, *Chen 42264* (MO); 10 li from Nan Shung, 15 Dec 1927, *Chun 5700* (A); Changjiangxiang, Renhua County, 800 m, 4 Sep 1958, *Deng 7490* (MO); Meikengxiang, Xinlai County, 300 m, 1 Nov 1958, *Deng 8306* (MO); Baishikeng, Jiu keng, Dinghu Mt, 200 m, 11 Jun 1964, *Ding & Shi 1512* (MO); Boluo County, Luofushan, 26 Apr 1978, *Guangdong Prov. 1978 Team 5908* (MO); Gaoyao County, Yankengxiang, 29 Mar 1957, *Huang 162696* (MO); Samshui, near Dam-shui, May 1875, *Lamont 1084* (BM); Yung-Yun City, Kai ngan tsz, 29 Nov 1932, *Lau 635* (A); Mo Po Lo, Tung Koon, 6 Apr 1935, *Lau 20766* (MO); Longmeng County, Shangping, vicinity of Shangping village, Nankunshan, Yonghan, 26 Dec 1956, *Li Xuegen 200363* (MO); Wengyuan, Wengyuan County, 22 Sep 1935, *Liu 24502* (MO); Gaoyao County, Hetaixiang, 6 Sep 1958, *Liu 01965* (MO); Lian County, Tianguangshan, Yao’anxiang, 2 Nov 1958, *Tan 60191* (MO); Yangshan County, Daping, Xiluxiang, 700 m, 27 Nov 1958, *Tan 60451* (MO); Ding-hu Shan, 11 Jun 1964, *Ting 1512* (A); Yam Na Shan [Yit nga shan] (Mei [Kaying] District), 4 Aug 1932, *Tsang 21496* (GH, K); Lokchong, 18 Oct 1928, *Tsiang 1403* (K); Jingyingsuo, Laoshan, Tianlin County, 600 m, 26 Nov 1957, *Zhang 11000* (MO); Dongxing, 15 May 1959, *Zhou 02801* (MO); **Guangxi**: Damiaoshan, Xiaosangxiang, Antai district, 480 m, 16 Nov 1958, *Chen 17286* (MO); Danyashan, Guilin City, 27 Oct 1958, *China Germany Team 026* (MO); Guilin City, 28 Oct 1958, *China Germany Team 135* (MO x2); Nor Yut, Tai Ching Shan, 19 Jun 1935, *Ko 55334* (A); Dabu, Guilin City, 130 m, 24 Oct 1993, *Li 13078* (MO); Lingui County, 185 m, 25 Oct 1994, *Li 14226* (MO); Haiyang, Haiyang shan, Lingchuan City, 500 m, 10 Jul 1995, *Li 14773* (MO); Nanbianshan, Lingui, 185 m, 7 Aug 1997, *Li 15968* (MO); Zhongfengxiang, Ziyuan county, 230 m, 13 Oct 1998, *Li 489* (MO); Mianliang Cun, Bai sheng xiang, Napoxian, 800 m, 7 Nov 1998, *Qin 2394* (MO); Ting-hsing-chai Shan, opposite Liang mao-shan, vicinity Pai-chou city, 20 Aug 1937, *Taam Ying-Wah 39* (A); Changtan, Longjin County, 30 Nov 1959, *Tan 57159* (MO); Daqingshan District, Longjin County, 550 m, 20 Dec 1957, *Tan 57478* (MO); Tang Lung Village, Shap Man Taai Shan, SW of Shang-sze, Kwangtung [Guangdong] border, 3 Sep 1934, *Tsang 24190* (A); Yao Shan, 1 Dec 1936, *Wang, C.W*., *40508* (A); Longsheng County, Huaping, Huaping Forest district, 780 m, 20 Sep 1984, *Wei Yuzhong* & *Lu Qinghua 20474* (MO); Jingxi County, Wupingxiang, Wupingxiang, 29 Dec 1958, *Zhang 14857* (MO); **Guizhou**: Yinjiang County, Zhangjiaba, Yinjiang Xian, along the trail between Zhangjiaba and Huguoshi on the W side of Fanjang Shan mountain range, 1200 m, 25 Sep 1986, *Bartholomew et al. 1651* (BM); 29 Jul 1936, *Deng Shiwei 90622* (MO); Jiangkou Xian, Heiwan River, on the SE side of the Fanjing Shan mountain range in the vicinity of the Ecological Station of the Guizhou Academy of Sciences, 560 m, 20 Aug 1986, *Sino-American Guizhou Bot. Expedition 78* (A); Songtao Xian, Lengjiaba, in the vicinity of the confluence of the Xiaohe and Dahe Rivers, NE side of the Fanjing Shan mountain range, 820 m, 5 Oct 1986, *Sino-American Guizhou Bot. Expedition 2159* (A); Tsunyi Xian, Liang Feng Yah, 10 Aug 1931, *Steward et al. 263* (A); Tsao-Feng-San, Tsingchen, 29 Jul 1936, *Teng 90622* (A); **Henan**: Teng-feng, Shao lin ssu im Kreise Teng fong, 670 m, Aug 1907, *Schindler 165* (BM, G, L, S); Xinyang County, Huoshao Temple, 600 m, 17 Jul 1985, *South Team T-0120* (MO); Xinyang County, Yuhuangshan, Yuhuangshan, 400 m, 17 Jul 1985, *South Team T-0180* (MO); Shangcheng, Mao’ershishan, Yangqiaoxiang, 200 m, 8 Sep 1998, *Wang G-73* (MO); **Hong Kong**: Chinese University of Hong Kong, 2 Nov 2004, *Li Ming 43* (A); **Hubei**: Yichang, Ichang, (Patung District), *Henry 110* (K); Yichang, Ichang, *Henry 635* (K); Yichang, Nan-t’o and mountains to northward. Ichang, Feb 1887, *Henry 2198* (K, US); Wuhan, along road near Wuhan Botanic Garden, 46 m, 30 Sep 2007, *Knapp et al. 10142* (BM); Songbai, Songbaizhen, Shennongjia Natural Reserve, 950 m, 2 Sep 1996, *Shi Shigui S-0950* (MO); Shennongjia Forest District, between Biacaoping and Bajiaomiao, 1400 m, 20 Sep 1980, *Sino-American Expedition 1576* (A); Jianshi County, 800 m, 15 Aug 1997, *Wang 97004* (MO); **Hunan**: Yizhang, Liyuan bao, 100 m, 13 Sep 1942, *Chen 2392* (MO); Dafobin, 20 Aug 1950, *Dahlström 255* (S); Xinning, Ma-Ling-Tung, Sinning Hsien, 21 Sep 1935, *Fan & Li 523* (A, BM, ECON); Hangsha, Datankeng, Hangshan, 980 m, 21 Aug 1964, *Huang 112416* (MO); Dongan, Shunhuangshan, 280 m, 1 Sep 2004, *Liu Jin-Kui 667* (US); Yongshun County, west of Hunan Province, 600 m, 15 Aug 1958, *Liu 9582* (MO); Hengshang, Fangguang Temple, Hengshan (Nanyue), 560 m, 11 Aug 1948, *Liu 00373* (MO); Yizhang, Mt. Mangshan, 700 m, 9 Aug 2005, *Xiao Bai-Zhong 4525* (K, US); Wugang County, Yunshan, Sanliting, Yunshan, 4 Sep 1950, *Zhang 4495* (MO); **Jiangsu**: Soochow, 23 Sep 1926, *Chiang 8236* (BH); Nanjing, Kiangsu province, 22 Oct 1966, *Chiao 12926* (BH, G, K); Chiu-yong, 21 Jul 1926, *Ling 12166* (GH); Nanjing, Nanking Kiangsu, 3 Oct 1929, *Sun 206* (K); Nanjing, Nanking Kiangsu, 4 Oct 1930, *Sun 727* (K); **Jiangxi**: Pailou County, Jiujiang, 110 m, 3 Sep 1995, *Ce-ming 95683* (BM); Jiujiang, Lushan, Kiangsi Prov. Lu Shan. Huang Yen Ssu, 4 Sep 1932, *Cheo 251* (ECON, K); Lu-shan-hsien, 1100 m, *Chu 4085* (BM); Lushan, Shimenjian Gorge, W slopes of Lushan Mountains, 698 m, 27 Sep 2007, *Knapp et al. 10139* (BM); Lushan, San Die Quan, (3 Steps Waterfall), Lushan Mountain, 661 m, 28 Sep 2007, *Knapp et al. 10141* (BM); Kiennan, Sai Hang Cheung, near Tung Lei Village, 1 Aug 1934, *Lau 4279* (BM, G, GH, S, US); Kiennan, Sai Hang Cheung, near Tung Lei Village, 1 Sep 1934, *Lau 4379* (BM, G, GH, S, US); Wuning County, Wuningyan, Wuningyan, 350 m, 23 Oct 1994, *Tan 941258* (MO); De-an Xian, Dingfeng, 160 m, 6 Nov 1996, *Tan 11003* (MO); Fenyi City, Dagangshan, Fenyi city, 26 Aug 1985, *Yao 9233* (A, K); **Shaanxi**: Mian County, from Changbazi to Xiasbianhe, 750 m, 2 Oct 1952, *Fu 6130* (MO); T’ai-hua Shan, 1 Aug 1932, *Hao 3832* (K); Huashan, Shensi, Hua-shan, 26 Aug 1932, *Hao 4192* (K); Zhenba County, 820 m, 20 Aug 1999, *Wang 522* (MO); Zhouzhi Xian, 1200 m, 28 Jul 1999, *Zhu et al. 2386* (MO); Yang Xian, 1600 m, 8 Jul 1999, *Zhu et al. 2909* (MO); Taibai Xian, 1800 m, 22 Jul 2000, *Zhu et al. 3166* (MO); **Shandong**: Meng Shan, Fei Hsien, 350 m, 25 Jul 1936, *Cheo & Yen 184* (BM, G, GH); Tai Ming Hu, 4 Sep 1920, *Chiao 3030* (B, K, PE, US); Shan Ling, Gaaoshan, 500 m, 29 Sep 2006, *Guo Chengyong 5470 -5* (US); Ningyang, *Guo Chengyong 51386 -1* (HITBC); Zoucheng, *Guo Chengyong 52414 -1* (HITBC); Yantai, Bamboo temple, 8 Oct 1980, *Herb. Forbes 8780* (BM); **Shanghai**: British Consulate, 26 Sep 1982, *Carles* s.n. (BM); Shanghai, 1887, *Faber 8* (K); **Shanxi**: Tongshan gongshe, Yuangu County, 800 m, 14 Oct 1982, *Fu 18757* (MO); Yüan-chü Distr., Ye-cho-shan, Fu-chis-ho, 1200 m, 18 Jul 1924, *Smith 6432* (MO); **Sichuan**: Yajiang Xian, along first tributary fo the Yalong Jiang E of the city of Yajiang, 541 m, 6 Aug 2006, *Boufford et al. 35933* (A); Xinlong Xian, confluence of the nearly dry tributary and Yalong Jiang at intersection of road from Ganzi to Xinlong (highway 217) and road to Baiyu, just downstream from village of Acha (Echa), 1244 m, 25 Aug 2006, *Boufford et al. 37306* (A); Omei Shan, 1100 m, 22 Aug 1938, *Chiao & Fan 358* (A, US); Omei Xian, Mt. Omei, Dec 1942, *Chow 7261* (A); Mt. Omei, Tapingssu, 16 Aug 1938, *Chow 8152* (A); Kiating, Pasientung, 8 Dec 1938, *Chow 8986* (A); Kiating, outside of the west gate, 20 Nov 1938, *Chow 8920* (A); Mount Omei, Omei Hsien, 18 Aug 1928, *Fang 3164* (A, K, US); Mowchow, Mow Hsien, 20 Sep 1928, *Fang 5503* (A, K, US); Lifan, Mung-twin-ko, Aug 1941, *Hu 1982* (A); Taking, 1958, *Li 78542* (A); Ta-tien, 1900 m, 2 Jul 1922, *Smith 2265* (A, MO); Chengkow, 1958, *Tai 102263* (A); Dujiangyan, Qingchengshan, Si Chuan, Guan Xian, 800 m, 20 Aug 1987, *Wang Zhong-tao 870423* (K); Kwang-yun Hsien, and vicinity, 11 Oct 1930, *Wang 22655* (GH); Mount Emei, 800 m, 2 Sep 1995, *Xu 4773* (MO); Jeui-shen Xian, 10 Jun 1932, *Yu 1015* (A); Dujiangyan, *Zhu Dahai 2232* (HITBC); **Tibet**: Chi-na-tung, Tsa-wa-rung [Sikang province], 2500 m, Aug 1935, *Wang 65289* (A); **Yunnan**: Momien, 30 May 1868, *Anderson* s.n. (K); Lijiang, Sashiba, 29 Aug 1939, *Ching 21378* (A); Lan ngy Tsin, 14 Jul 1904, *Brother Ducloux 2728* (A); Muli, Consinling near Ngerya on the border of Chungtien, in side valley, 2300 m, 24 Aug 1939, *Feng 2778* (A); Si-chour Xian, Faa-doou, 1400 m, 13 Sep 1947, *Feng 11662* (A); Mar-li-po, Hwang-jin-in, 1100 m, 13 Nov 1947, *Feng 13234* (A); Lichiang, 2743 m, Jun 1910, *Forrest 5922* (BM, K); Tengyueh, hills around Tengyueh, 1829 m, Jul 1912, *Forrest 8630* (K); Gongshan, Bingzhonglou, Nujiang Lisa Aut. Pref., Dan Dong village outskirts on road to Bingzhonglou, near Gongshan, 1450 m, 9 Sep 1997, *Gaoligong Shan Expedition 1997 8738* (E); Mephyr, 1524 m, *Henry 11855* (A, K, US); Chengjiang Xian, 18 Jun 1940, *Hou 74591* (MO); Lijiang, Judian, *Qingzhang Team*, *425* (HITBC); Lijiang, Yangtze watershed, prefectural district ft Likiang [Ligiang], slopes of Likiang Snow Range, May 1922, *Rock 4311* (A, US); Ho-ch’ing, inter Hoching et Singquch, 2600 m, 27 Sep 1914, *Schneider 2705* (GH, K); Lan-ping Xian, 17 Aug 1933, *Tsai 53712* (A); Chiu-kang, W of Chamutung, 2000 m, Oct 1935, *Wang 67159* (A); Wei-si Xian, Yeh-Chih, 2500 m, Jul 1935, *Wang 67927* (A); A-tun-tze, 2700 m, Sep 1935, *Wang 70376* (A); Lung-kai, Cheng-kiang, 2350 m, 4 Aug 1939, *Wang 41503c* (A); Atuntze, Mt Kaakerpu, 2800 m, 20 Sep 1937, *Yu 10384* (A, BM); Muli, Muli, Wachin, Buraton, 2900 m, 2 Nov 1937, *Yu 14713* (A, BM); Chungtien, Haba, 2600 m, 22 Nov 1937, *Yu 14941* (A, BM); Salwen Valley, Dara, 1800 m, 24 Jun 1938, *Yu 22007* (A); **Zhejiang**: Hangzhou City, Oct 1957, *Jin Yilang 322* (MO); Hupaoma’er shan, Hangzhou City, 25 Sep 1953, *Wu 1306* (MO); Hangzhou, *Zhu Qiugui 292* (HITBC).

**Japan**. **Hokkaido**: Ishikari, 10 Sep 1903, *Arimoto* s.n. (GH); **Honshu**: Toyko, Koishikawa, 22 Aug 1893, *Anonymous* s.n. (US); Toyko, Jul 1887, *Anonymous 338* (US); Yamaguchi, Hakusan Shrine, in vicinity of Kika Park, base of east slope of Castle Mountain(Shiro-yama) National Forest, west bank of Nishiki River, Yokoyama Village in Iwakuni City, 25.5 miles west of Hiroshima City, 18 m, 19 Sep 1953, *Charette 1409* (S, US); Shizuoka, Mt. Takakusa-yana, Uchinoya, Okabe-cho, Shida-gun, a mountain 5 km from the seashore of the Pacific Ocean and facing the sea, 150 m, 4 Sep 2006, *Konta 35512* (US); Tokyo, 18 Aug 1878, *Matsumura* s.n. (US); Wakayama, Takaike, Kozagawa, Wakayama Pref., Higashimuro-gun, Kozagawa-cho, Takaike, 20 m, 5 Nov 1964, *Matsushita 125* (US); Shinano, Okayama-mura, Shimo-minochi-gun, 14 Aug 1953, *Mizushima 12628* (S); Kyoto-fu, Oomoto-honbu, Ten’on-kyo, Kameoka-shi, 19 Sep 1991, *Murakami 134* (A); Miyagi Pref., Sendai-shi, Katahira-cho, N Honshu, 11 Aug 1972, *Sato 72811* (B); route from Mizundo to summit of the Mt. Atago, Saga, Ukyo-ku, Kyoto-shi, 330 m, 10 Oct 1991, *Takahashi 2215* (A); Kyoto, Is. Anja, Obase, Nishi-oura-chiku, Maiduru-shi, 10 m, 9 Nov 2000, *Takahashi & Sawada 30261* (A); Kii, Mt. Koya, en route from Daimon to Yatate, 650 m, 9 Sep 1962, *Tamura 9362* (S); Ishikawa, Oosugidani, Oosugi-uemachi, Kamatsu-shi, 360 m, 11 Oct 1992, *Tsugaru & Murata 17048* (A); Kyoto, Ashiodani, Yotsuya, Hiyoshi-cho, Funai-gun, 250 m, 1 Sep 1994, *Tsugaru & Takahashi 20836* (A); Kyoto, Mt. Kunimiyama, Onyu, Nishi-Oura-chiku, Maidzuru-shi, 10 m, 9 Nov 1997, *Tsugaru & Takahashi 25940* (A); en route from Kobama to Kaketsu, Amino-cho, Takeno-gun, 10 m, 12 Nov 1998, *Tsugaru & Takahashi 27273* (A); Mt. Takahura, Okayamayen, 15 Aug 1952, *Uno* s.n. (A); **Kyushu**: Miyazaki Pref., Miyakonojo City, Yamanokuchi, 17 Aug 1950, *Hatusima 14243* (US); Yoshino, Kugoshima City, Kyusyu, 4 Oct 1930, *Naito* s.n. (US); Nagasaki, Is. Tsushima, Shimoagata-gun, Izuhara, 22 Oct 1973, *Ohashi et al. 27* (A, US); **Shikoku**: Tokushima, 1919, *Krug 239* (B); Tokushima, 1919, *Krug 663* (B).

**Republic of Korea**. Quelpaert, Nov 1906, *Faurie 775* (BM); Soonchun, Schulla Prov, 3 Sep 1934, *Smith* s.n. (GH); sin. loc, 300 m, Oct 1907, *Taquet* s.n. (G); 27 Aug 1908, *Taquet 1146* (K); Quelpaert, 25 Jun 1908, *Taquet 1147* (G, K).

**Taiwan**. Pa-hsien-shan Forest Recreation Area, T’ai-chung Hsien, Ho-p’ing Hsiang, 800 m, 27 Nov 1997, *Bartholomew et al. 7613* (GH); Yangmingshan, Taipei County, 18 Feb 1962, *Chuang & Lin Pi 4824* (A); Biyoritne [?], 27 Mar 1903, *Faurie 322* (A, BM, G); Kaohsiung, Takao [?], Jan 1914, *Faurie 638* (BM, G); Hualien, Yen-tzu-k’ou, 14 Dec 1990, *Huang 15045* (A); Compartment 114, Wushikon, Taichung, 14 Oct 1957, *Liu* s.n. (A); sin. loc., 1864, *Oldham 340* (BM); Hsiulin Hsiang, Taroko National Park, 500 m west of Loshao, at road marker 153.5 along Prov. Hwy. 8 between Loshao and Songshan Tunnel, 1100 m, 15 Jun 1999, *Peng & Anderberg 17285* (S); Mosha, 9 Jul 1912, *Price 816* (K).

**Vietnam**. **Cao Bang**: Distr. Ha Lang, municipality Dong Loan, vicinity of Ban Lung and Lung Phuc, about 50 km to E from Cao Bang town, 500 m, 25 Nov 1998, *Averyanov CBL-657* (MO); Tonkin, base du massif calcaire au pied du camp militaire, Aug 1943, *Pételot 8464* (A).

#### 25. Solanum macbridei

Hunz. & Lallana, Lorentzia 4: 17. 1981

http://species-id.net/wiki/Solanum_macbridei

Figure 63

##### Type.

Peru. Cuzco: Quispicanchis, Marcapata, 12000 ft, 17 Oct 1937, *D. Stafford 984* (holotype: K [K000585558]; isotypes: BM [BM000815938], F [F-1498721]).

##### Description.

Shrubs with many erect dichasial branches, 1–1.5 m tall, the branches and branchlets erect and densely packed. Stems densely pubescent with dendritic trichomes, these persistent; leaf scars very prominent, the stems strongly winged from the decurrent leaf bases; new growth purplish green, glabrous except for a few dendritic trichomes on the revolute leaf margins. Bark of older stems grey-brown, sparsely pubescent with dendritic trichomes. Sympodial units plurifoliate. Leaves simple, 0.9–2.3 cm long, 0.2–0.7 cm wide, narrowly elliptic, shiny, fleshy and coriaceous, both surfaces glabrous except for a few dendritic trichomes on the revolute margins and on the midvein of the adaxial surface; primary veins obsure, relatively few; base attentuate; margins entire, strongly revolute; apex acute, somewhat apiculate; petiole very short to absent, usually 1–2 mm long. Inflorescences terminal, appearing lateral from overtopping shoot growth, 0.2–1 cm long, compact and more or less globose, branching 2–4 times, with 2–5 flowers, the axis completely glabrous and shiny; peduncle 0.3–0.5 cm long, often absent and the inflorescence appearing to arise directly from the stem; pedicels 4.5–5 mm long, tapering from a basal diameter of 0.5 mm to an apical diameter of ca. 1 mm, deflexed and nodding at anthesis, glabrous, inserted in a prominent wrinkled sleeve 1–1.5 mm long; pedicel scars tightly spaced. Buds globose, later elliptic, the corolla strongly exserted from the calyx tube. Flowers all perfect, 5-merous. Calyx tube conical, ca. 0.5 mm long, the lobes broadly deltate, ca. 0.5 mm long, glabrous on both surfaces with a few simple trichomes to 0.5 mm long on the margins of the lobes. Corolla 1.4–1.7 cm in diameter, violet, rotate-campanulate, cupulate and nodding at anthesis, lobed 1/2 way to the base or less, the lobes 2–3 mm long, 2–3 mm wide, densely pubescent abaxially with dendritic trichomes, adaxially glabrous. Filament tube absent; free portion of the filaments ca. 1 mm long, glabrous; anthers ca. 2 mm long, 0.5–1.5 m wide, loosely connivent, poricidal at the tips, the pores becoming slit-like with age. Ovary glabrous or occasionally with a few scattered dendritic trichomes near the apex; style ca. 6 mm long, more or less densely pubescent with dendritic trichomes near the middle; stigma obscurely bilobed. Fruit a globose and slightly apically pointed berry, 6–9 mm in diameter, purplish-black, fleshy with thin pericarp; fruiting pedicels ca. 6 mm long, ca. 0.5 mm in diameter at the base, woody, deflexed. Seeds ca. 8 per fruit, ca. 5 mm long, 3 mm wide, pale reddish-brown, flattened lenticular, the surfaces minutely pitted. Chromosome number: not known.

**Figure 63.**
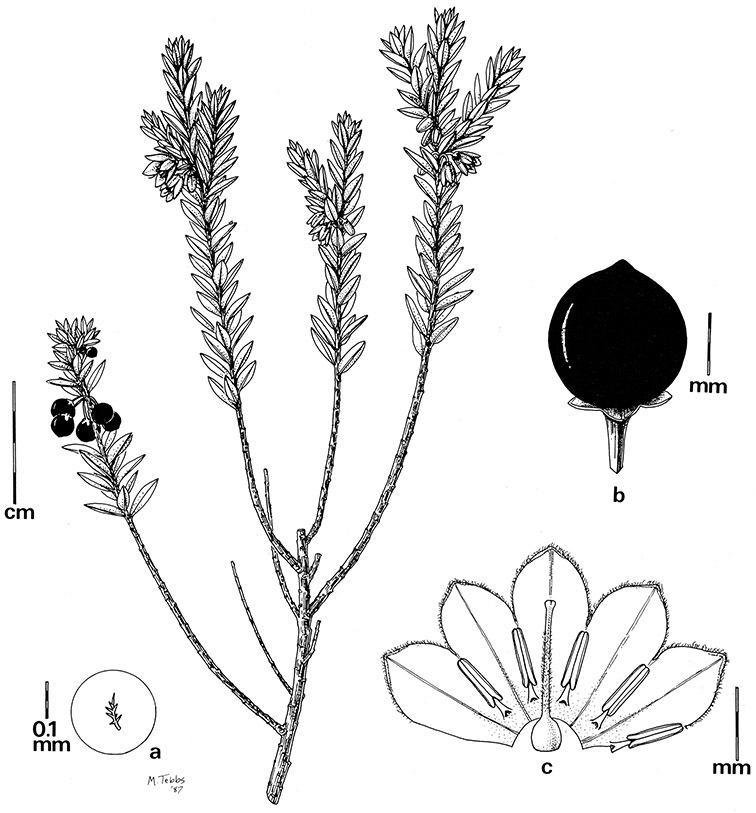
*Solanum macbridei* Hunz. & Lallana. (**A–C** drawn from *Solomon & Stein 11660*). Reproduced from Knapp (1989) with permission of the Natural History Museum Botany Library. Illustration by Margaret Tebbs.

##### Distribution

**Figure 64.**
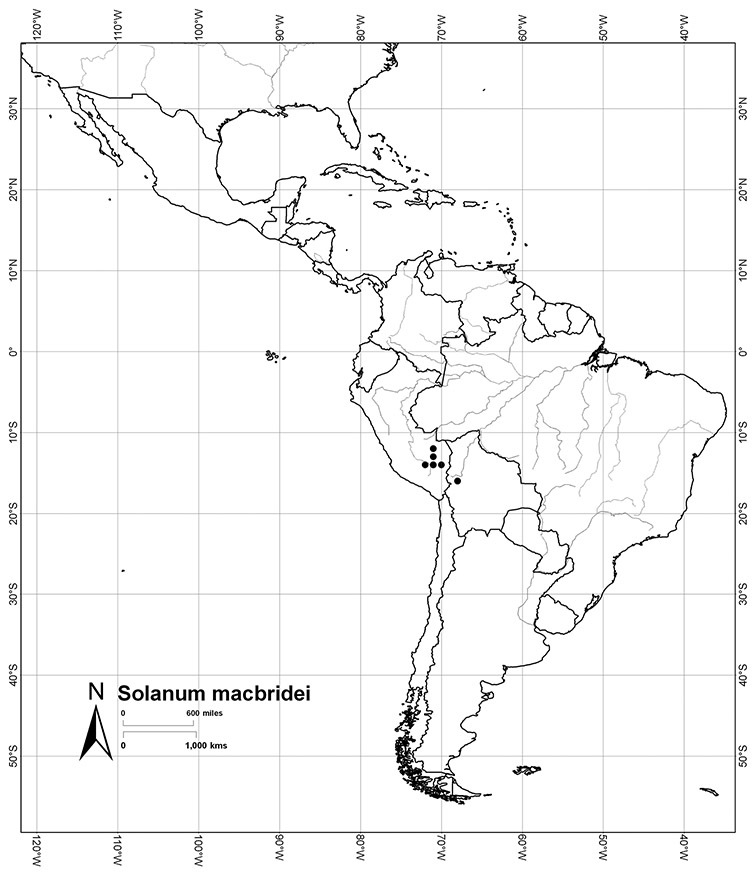
Distribution of *Solanum macbridei* Hunz. & Lallana.

(Figure 64). S Peru to N Bolivia, 3800–4600 m.

##### Ecology.

Above timberline in boulder fields and among rocks in moist puna.

Conservation status. Possible Near Threatened (possible NT); EOO <75,000 km^2^ (LC) and AOO >10,000 km^2^ (LC). See Moat (2007) for explanation of measurements.

##### Discussion.

*Solanum macbridei* is morphologically one of the most distinctive members of the entire genus *Solanum*. It is unique in its campanulate corolla and in its small, coriaceous leaves. Sheets of this species have been identified as members of the Gentianaceae, Ericaceae and as the genus *Fabiana* Ruiz & Pav. (Solanaceae). The pedicel sleeve characteristic of the *Solanum nitidum* species group is very pronounced in *Solanum macbridei*, partly because the inflorescence is completely glabrous and thus the character is more easily visible than in other more pubescent species in the group.

In his original description of *Solanum macbridei*, Hunziker (1981) speculated that it was closely related to *Solanum albescens* (Britton) Hunz. (= *Solanum leptocaulon* van Huerck & Mull.-Arg.). Hunziker thought the anthers of both species dehisced by slits rather than pores (the synapomorphy for *Solanum*), but he was incorrect in the case of *Solanum macbridei*, whose anthers are typically solanum-like, dehiscing by terminal pores that are more or less tear-drop shaped. The coriaceous leaves of *Solanum leptocaulon* are almost certainly related to its high elevation habitat, as are those of *Solanum macbridei*. *Solanum leptocaulon* is a species of uncertain affinities in *Solanum* (L. Bohs, pers. comm.), but is clearly also a *Solanum* with initially poricidal anthers that only later dehisce by slits.

##### Specimens examined.

**Bolivia**. **La Paz**: La Fabulosa, tin mine at the head of the Challana valley 80 miles north of La Paz, 4024 m, 3 May 1950, *Brooke 6355* (BM, F); Larecaja, Cacacani, 4500 m, Dec 1860, *Mandon 1499* (BM, K); Pedro Domingo Murillo, side valley 0.5 km up from Pongo on gravel road to Mina Alaska (=Mina Copacabana), 6.5 km W of Unduavi, 3850 m, 29 Oct 1984, *Nee & Solomon 30182* (BH, GH, K); Pedro Domingo Murillo, 0.5 km up from Pongo on gravel road to Mina Alaska (Mina Copacabana), 3900 m, 9 May 2001, *Nee et al. 51758* (BM, NY); Pedro Domingo Murillo, 3.5 km W of Pongo on road to Unduavi, 3850 m, 8 Mar 1984, *Solomon & Stein 11660* (K).

**Peru**. **Cusco**: Quispicanchis, Marcapata, leaving village and heaving back to Cuzco, 11 km from peak, 4350 m, 23 Jul 1978, *Aronson & Berry 541* (F, MO, USM, USM); Paucartambo, Qollatambo, Parque Nacional Manu, 3800 m, 8 Sep 1990, *Cano 4180* (F, MO, USM); Paucartambo, Huáscar, Parque Nacional Manu, 3900 m, 9 Sep 1990, *Cano 4225* (USM); Quispicanchis, Marcapata, 4000 m, 20 May 1954, *Hincher P-1270* (F); 55 km E of Ocongate, 4310 m, 19 Jan 1973, *Madison 1038* (GH); Quispicanchis, entre Abra Walla Walla y Marcapata a 210 km de Cusco, 2800 m, 21 Apr 1988, *Núñez V. et al. 8999* (F, MO); Quispicanchis, Marcapata, Checta cuchi, 4250 m, 11 Dec 1938, *Vargas 1361* (F); Quispichanchis, Marcapata, Ccompi-pampa, on the grade from Huaillai to Hualla-hualla, 4100 m, 11 Dec 1938, *Vargas 9716* (F, K); Paucartambo, Escalerayoc, Dist. Marcachea, 3800 m, 31 Jul 1939, *Vargas 11178* (F); **Puno**: Carabaya, Macusani, 1854, *Lechler 2685* (K).

#### 26. Solanum muenscheri

Standl. & Steyerm., Publ. Field Mus. Nat. Hist., Bot. Ser. 22: 275. 1940

http://species-id.net/wiki/Solanum_muenscheri

Figure 65

##### Type.

Guatemala. Sololá: on mountain slope near Santa María, 10500 ft, 14 April 1937, *W.L.C. Muenscher 12360* (holotype: F [F-905753, F neg. 49440]; isotype: BH [BH000039462]).

##### Description.

Shrubs or small trees, 1–10 m tall. Stems densely pubescent with greyish dendritic trichomes; new growth densely pubescent with greyish-yellow dendritic trichomes. Bark of older stems sparsely pubescent, greyish-yellow. Sympodial units plurifoliate, branching monochasial or dichasial. Leaves simple, 5.5–15 cm long, 1.2–3.5 cm wide, narrowly elliptic, the adaxial surfaces densely to sparsely pubescent with dendritic trichomes, these denser along the veins in sparsely pubescent individuals, the abaxial surfaces densely pubescent with dendritic trichomes on the veins and lamina; primary veins 10–20 pairs, pubescent; base acute, not winged on to the petiole; margins entire; apex acute to acuminate; petiole 1–2 cm long, not winged from the leaf bases. Inflorescences terminal, occasionally in the fork of branches, later appearing lateral from overtopping of shoots, 1–5 cm long, branching 2–4 times, with 5–25 flowers, densely pubescent with greyish-yellow dendritic trichomes like those of the stem and leaves; peduncle 0.5–1 cm long; pedicels 0.7–1.1 cm long, tapering from a basal diameter of 0.75–1 mm to an apical diameter of 2–3 mm, densely pubescent with dendritic trichomes, these somewhat longer than the trichomes of the leaves and stems, nodding at anthesis, articulated at the base and inserted into a sleeve ca. 1 mm long; pedicel scars uneven spaced to 1 cm apart, but clustered distally. Buds ellipsoid, the corolla strongly exserted from the calyx tube. Flowers all perfect, 5-merous. Calyx tube conical, 1.5–2 cm long, the lobes 2–2.5 m long, deltate to long triangular, densely pubescent with dendritic trichomes adaxially and abaxially. Corolla 1.5–2 cm in diameter, violet to deep purple, stellate to rotate-stellate, lobed 1/2 to 2/3 of the way to the base, the lobes 7–10 mm long, 4–5 mm wide, planar or slightly reflexed at anthesis, densely pubescent with dendritic trichomes abaxially, sparsely pubescent with dendritic trichomes on the veins and petal surfaces adaxially, the trichomes denser at the tips of the lobes. Filament tube absent; free portion of the filaments 0.5–1 mm long, glabrous; anthers 2–2.5 mm long, ca. 1 mm wide, loosely connivent, poricidal at the tips, the pores becoming slit-like with age. Ovary glabrous or with a few dendritic trichomes at the apex; style 4–6 mm long, densely pubescent with dendritic trichomes from the base to 3/4 of its length; stigma clavate, the surface minutely papillose. Fruit a globose berry, 0.8–1 cm in diameter, black with thin pericarp; fruiting pedicels 1.5–1.8 cm long, ca. 1.5 mm in diameter at the base, woody, deflexed; calyx lobes somewhat accrescent in fruit, 3–5 mm long. Seeds ca. 20 per berry, ca. 3.5 mm × 2.5 mm, reddish-brown, lenticular, the surfaces minutely pitted. Chromosome number: not known.

**Figure 65.**
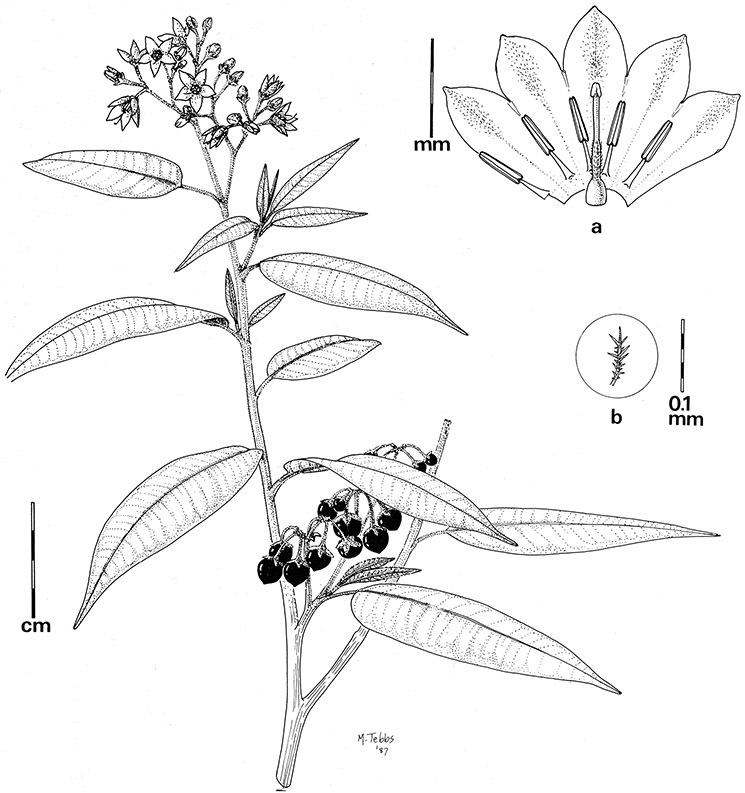
*Solanum muenscheri* Standl. & Steyerm. (**A–B** drawn from *Steyermark 35633*). Reproduced from Knapp (1989) with permission of the Natural History Museum Botany Library. Illustration by Margaret Tebbs.

##### Distribution

**Figure 66.**
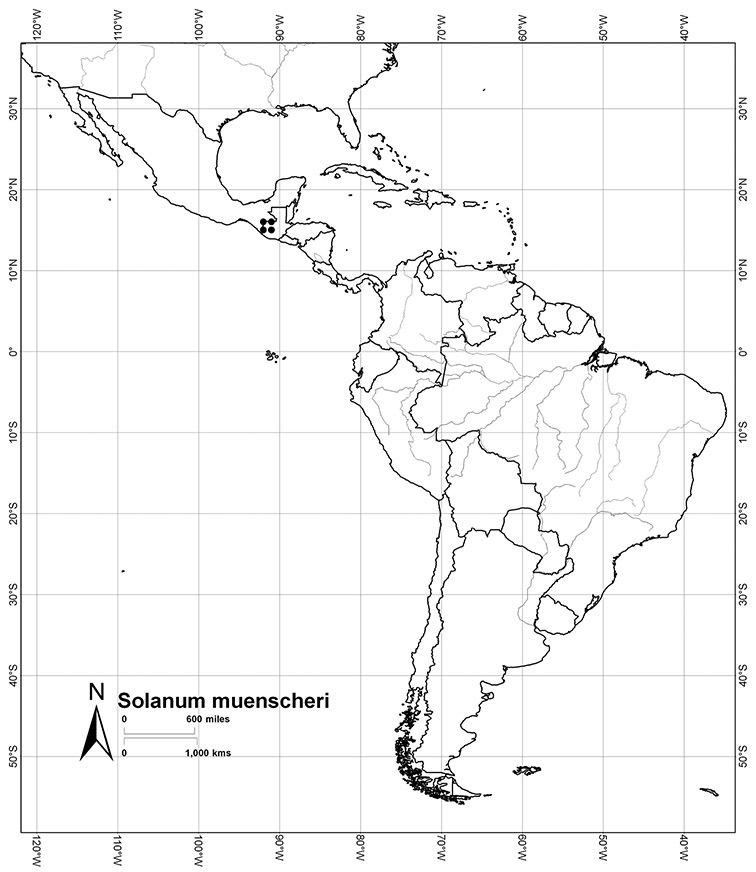
Distribution of *Solanum muenscheri* Standl. & Steyerm.

(Figure 66). Mountains of NW Guatemala and SW Mexico, from 2500-4000 m. Most collections come from the Sierra de Cuchumatanes in Guatemala.

##### Ecology.

Montane or cloud forests; dry slopes and *Juniperus* L. (Cupressaceae) forests.

##### Conservation status.

Endangered (EN); EOO <5,000 km^2^ (EN) and AOO <2,000 km^2^ (VU). See Moat (2007) for explanation of measurements.

##### Discussion.

*Solanum muenscheri* is a species of very limited distribution, found only in the mountains of N Guatemala and adjacent S. Mexico. It is apparently common locally and grows in mixed woodland and grassland near the tree line. Flowering specimens have been collected throughout the year, but there appears to be a peak in flowering in December.

*Solanum muenscheri* is similar to the Andean species *Solanum nitidum*, with which it is similar and perhaps closely related (Knapp 1989), in its soft dendritic trichomes and parallel veined leaves. It differs from that species in having black mature berries, yellowish indument and in its generally somewhat smaller inflorescences and in being only found in Guatemala and Mexico.

##### Specimens examined.

**Guatemala**. **Chimaltenango**: Chemal, 31 Dec 1940, *Johnston 1727* (F); **Huehuetenango**: Sierra de los Cuchumatanes, El Mirador, 3000 m, 12 Jan 1966, *Molina R. et al. 16503* (F); Sierra de los Cuchumatanes, between Paquix and Lllanos San Miguel, road to San Juan Ixcoy, 3300 m, 17 Nov 1967, *Molina R. 21215* (F); Sierra de los Cuchumatanes, Chemal, 4000 m, 13 Sep 1971, *Molina R. & Molina 26410* (F); Huehuetenango, road from San Juan Ixcoy to Huehuetenango, high plateau in the center of the Sierra de los Cuchumatanes, 3450 m, 13 Mar 2003, *Schneider et al. 104* (B); Sierra de los Cuchumatanes, between the first cumbre and La Pradera, 3500 m, 28 Dec 1940, *Standley 81147* (F); Sierra de los Cuchumatanes, along road beyond La Pradera, km 32, 3300 m, 31 Dec 1940, *Standley 81724* (F); Sierra de los Cuchumatanes, El Mirador, at the summit of the road leading from Huehuetenango to Sierra de los Cuchumatanes, 3300 m, 31 Dec 1940, *Standley 81886* (F); Sierra de los Cuchumatanes, between Chemal and Calaveras, 2800 m, 9 Aug 1942, *Steyermark 50333* (F); Sierra de los Cuchumatanes, just below Calaveras, 3000 m, 4 Dec 1962, *Williams et al. 22388* (F); **San Marcos**: Volcán Tajumulco, along road between San Sebastian at km 21 and km 8, 8-18 miles NW of San Marcos, 2700 m, 15 Feb 1940, *Steyermark 35633* (F); **Sololá**: Volcán Zuñil, 3000 m, 27 Dec 1976, *Schwabe* s.n. (B); **Totonicapán**: La Cumbre, between kms 150-158 vicinity of La Cumbre of Totonicapan, 2500 m, 1 Dec 1969, *Molina R. & Molina 25054* (F); Chiu Jolom, mountains above Totonicapán on road to Desconseulo, 2800 m, 23 Jan 1941, *Standley 84478* (F); Sierra Madre mountains about 8-10 km airline S of Totonicapán, 3100 m, 13 Dec 1962, *Williams et al. 22919* (F).

**Mexico. Chiapas**. Near summit of Volcán Tacaná, 2200 m, 30 July 1972, *Breedlove 26699* ( CAS, NY).

#### 27. Solanum nitidum

Ruiz & Pav., Fl. Peruv. 2: 33, tab. 163. 1799

http://species-id.net/wiki/Solanum_nitidum

Figure 67

Solanum calygnaphalum Ruiz & Pav., Fl. Peruv. 2: 31. 1799. Type: Peru. Junín and Huánuco: Tarma and Acomayo, *H. Ruiz & J. Pavón* s.n. (neotype, designated by Knapp 2008c, pg. 312: MA [MA-747146]).
Solanum gnaphaloides Pers., Syn. 1: 223. 1805, nom. nov. superfl. Type: Based on *Solanum calygnaphalum* Ruiz & Pav.
Witheringia angustifolia Dunal, Solan. Syn. 2. 1816. Type: Ecuador. Cotopaxi: Mt. Cotopaxi, *A. Humboldt & A. Bonpland 3069* (holotype: P-Bonpl. [P00136351, Morton neg. 8171]).
Solanum heteranthera Willd., Syst. Veg. ed. 15 bis [Roemer & Schultes] 4: 663. 1819. Type: Ecuador. Cotopaxi: Cotopaxi, Jul 1802, *A. Humboldt & A. Bonpland* s.n. (holotype: B-W [B-W-4347, F neg. 2893, IDC microfiche 271-315.298:III.5]).
Solanum nitidum Ruiz & Pav. var. *angustifolium* Dunal, Prodr. [A.P. de Candolle] 13(1): 93. 1852. Type: Bolivia. Sin. loc., *A. D’Orbigny 1536* (holotype: P [P00366857]; isotypes: G [G00343475, Morton neg. 8618]).
Solanum rhamnoides Dunal, Prodr. [A.P. de Candolle] 13(1): 100. 1852. Type: Bolivia. Palea [Palca?], *A. D’Orbigny 293* (holotype: P [P00507312]).
Solanum cotopaxense Dunal, Prodr. [A.P. de Candolle] 13(1): 139. 1852. Type: Based on *Witheringia angustifolia* Dunal
Solanum theresiae Zahlbr., Beih. Bot. Centralbl. 13: 83. 1902. Type: Bolivia. La Paz: La Paz, Oct 1898, *Prinzessin Therese von Bayern* s.n. (holotype: M [M0166048, F neg. 6547]).

##### Type.

Peru. Junín: Tarma, May, June, *H. Ruiz & J. Pavón* s.n. (lectotype, designated by Knapp 2008c, pg. 320: MA [MA-747147]; isolectotypes: F [F-844722, frag.], G, MA [MA-747146, F neg. 29726]), P [P00366843, P00366844]).

##### Description.

Shrubs or small trees, 1–4 m tall. Stems densely pubescent with fine, grey, dendritic trichomes, these soon deciduous; leaf scars somewhat raised; new growth sparsely to densely pubescent with fine, grey, dendritic trichomes. Bark of older stems grey, glabrous. Sympodial units plurifoliate. Leaves simple, 6–9 (12) cm long, 1.5–2.5 (5) cm wide, narrowly elliptic, larger and broader in plants growing in shade (see discussion), often shiny above, both surfaces glabrous to densely pubescent with fine dendritic trichomes, these denser along the veins, with most specimens glabrous to sparsely dendritic pubescent; primary veins 12–15 pairs, prominent and parallel; base acute, somewhat winged on to the petiole; margins entire, not revolute; apex acute; petiole 1–1.5 cm long. Inflorescences terminal, later appearing lateral from overtopping of the shoots, 3–7 cm long, pyramidal, branching 8–10 times, with 10–20 flowers, sparsely to densely dendritic pubescent; peduncle 0.5–1.5 cm long, the branching often beginning just distally to the last leaf; pedicels 0.7–1.2 cm long, tapering from a basal diameter of 0.5 mm to an apical diameter of 1 mm, densely pubescent with fine, grey, dendritic trichomes, deflexed or horizontal at anthesis, articulated at the base and inserted in a sleeve ca. 0.5 mm long; pedicel scars closely spaced and clustered near the inflorescence branch tips. Buds globose, later elliptical, strongly exserted from the calyx tube. Flowers all perfect, 4–5-merous. Calyx tube 1–2.5 mm long, conical, the lobes 1–3 mm long, deltate to long-triangular, densely pubescent abaxially with fine dendritic trichomes, pubescent with fine dendritic trichomes in the upper 1/2 adaxially. Corolla 1.5–2.4 cm in diameter, violet or occasionally white, stellate and slightly cupped, lobed 3/4 of the way to the base, the lobes 9–12 mm long, 5–7 mm wide, planar at anthesis, densely pubescent abaxially with fine dendritic trichomes, glabrous or sparsely pubescent along the midvein adaxially, the trichomes denser at the tips of the lobes. Filament tube absent; free portion of the filaments ca. 1 mm long, glabrous; anthers 2.5–3 mm long, ca. 1 mm wide, loosely connivent, poricidal at the tips, the pores becoming slit-like upon drying. Ovary glabrous or with a few dendritic trichomes near the apex, glabrate in fruit; style 6–8 mm long, sparsely to densely pubescent with dendritic trichomes at the base or along the entire length; stigma capitate to clavate, the surface minutely papillose. Fruit a globose berry, 0.7–1 cm in diameter, greenish black when immature and becoming bright red at maturity, with thin pericarp, the calyx lobes to 4 mm long and somewhat accrescent and woody in fruit; fruiting pedicels 1.2–1.5 cm long, ca. 1 mm in diameter at the base, woody, deflexed. Seeds ca. 20 per berry, 2–2.5 mm long, 1.5–2 mm wide, reddish-brown, flattened lenticular, the surfaces minutely pitted. Chromosome number: not known.

**Figure 67.**
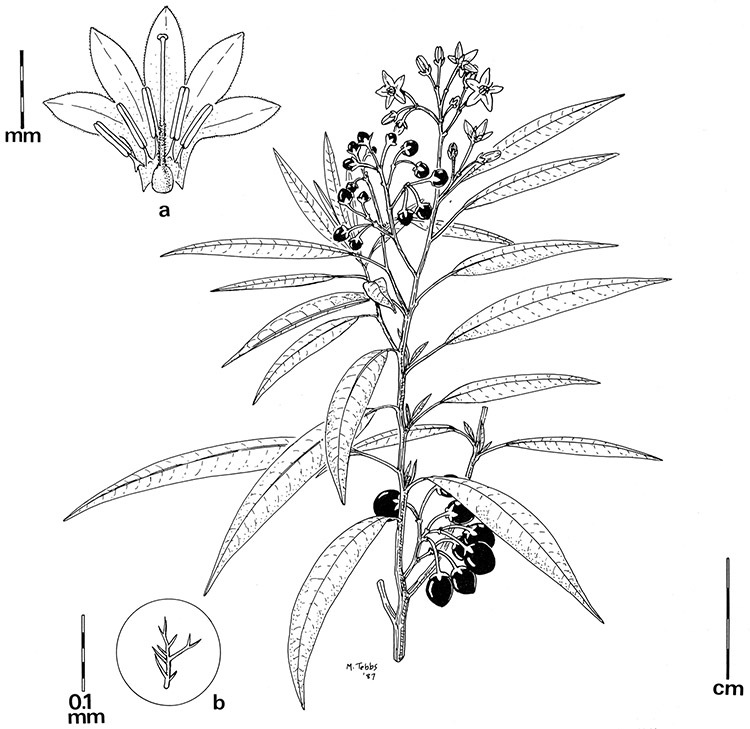
*Solanum nitidum* Ruiz & Pav. (**A–B** drawn from *D.N. Smith 1622*). Reproduced from Knapp (1989) with permission of the Natural History Museum Botany Library. Illustration by Margaret Tebbs.

##### Distribution

**Figure 68.**
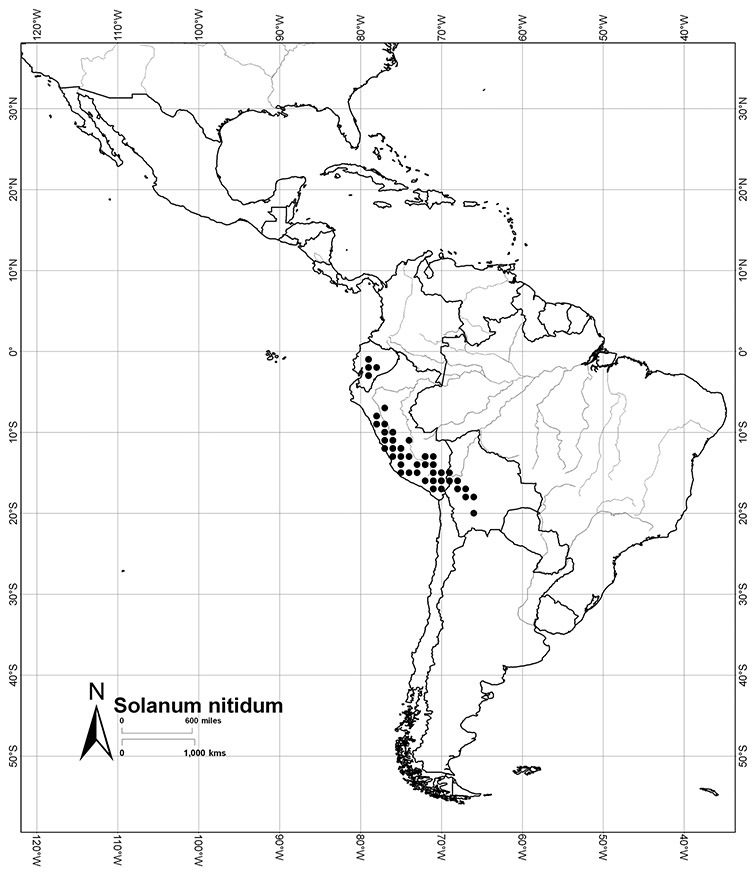
Distribution of *Solanum nitidum* Ruiz & Pav.

(Figure 68). From central Ecuador to Bolivia, 3000–4000(-4500) m.

##### Ecology.

Moist microhabitats in puna (high elevation grassland) and montane cloud forests; along streams and at the edges of forest patches.

##### Common names:

Peru: yurah nuñumia, ñuñumia, nunumaya, ñuñuccai, ñuñunquia, rapace, huisacassa, campucassa, cahuincho, catruincho, illauru, tacachilla. Bolivia: chinchichinchi, nuñumaya, takachilla (see Knapp 1989).

##### Conservation status.

Least Concern (LC); EOO >100,000 km^2^ (LC) and AOO >10,000 km^2^ (LC). See Moat (2007) for explanation of measurements.

##### Discussion.

*Solanum nitidum* is a common component of high elevation areas in Peru and Bolivia, and it is also commonly grown in household gardens for its medicinal properties. It is most closely related (assessed using morphology, see Knapp 1989) to *Solanum muenscheri* of Guatemala and S Mexico, differing from that species in its red berries and its Andean distribution. Pubescence is quite variable in *Solanum nitidum*, but many intermediates exist and there appears to be no geographical component to the variation.

Broad repand leaves are found in young plants of *Solanum nitidum*, and leaf shape is quite variable across the species range. Polymorphism for flower colour exists in *Solanum nitidum*, with some populations consisting of only plants with white flowers, while others are of mixed white and purple flowered plants. White-flowered plants are much rarer than those with purple flowers. This colour polymorphism is common in the spiny solanums and does occur throughout the genus.

*Solanum nitidum* is commonly grown in household gardens in highland communities for its medicinal properties as well as for its attractive purple flowers. The most widespread vernacular names are variations of ñuñumaya: ‘ñuñu’ = breast or teat in Quechua. The term ñuñumiya as an entry in one Quechua dictionary (Cusihuamán 1976) refers to [transl.] ‘ñoñomia, shrub with grape-like berries, but that are very bitter’. Herrera (1941) records the use of the bitter berries as an emetic and sudorific in the treatment of various diseases, and the use of the juice of the berries being smeared on the breasts of women wanting to wean their children (probably the origin of the common name in Quechua). In Puno (label data from *Mullins 7*), the bright red berries are used as a dye. Ruiz and Pavón record the use of poultices of the leaves to open wounds and draw out splinters (‘espinas’) and infections (a suppurating agent). In Bolivia, the boiled berries are used as an insecticidal wash against ticks and fleas for both humans and domestic animals (label data from *Alvárez 84*) and are also used in the treatment of a variety of contagious skin diseases. Crushed leaves are also used as a compress for skin conditions. Most of these uses and treatments depend upon the extreme bitterness of the berries; this is probably due to their high alkaloid content.

*Solanum nitidum* was chosen over the simultaneously published *Solanum calygnaphalum* Ruiz & Pav. by J.F. Macbride (1962) in his treatment of Solanaceae for the Flora of Peru and his choice is followed here.

Many monographers in *Solanum* (including me) have stated that holotypes or lectotypes for Ruiz and Pavón names were in the Madrid herbarium (MA), but without specifying a particular sheet. This is relatively straightforward in some cases, but in others multiple sheets in the Ruiz and Pavón herbarium at MA means that these type designations are not sufficiently precise. Knapp (1989) lectotypified *Solanum nitidum* and *Solanum calygnaphalum* citing only single sheets in MA; this was rectified in 2008 by citation of the particular sheet (Knapp 2008c).

*Witheringia angustifolia* and *Solanum heteranthera* are likely to be based on two duplicates of the same Humboldt and Bonpland collection from Cotopaxi in Ecuador. There are no duplicates of “Bonpland 3069” in B-W, and the near simultaneous publication of names based on Humboldt and Bonpland’s collections was common (see Knapp 2007b).

##### Specimens examined.

**Bolivia**. **Cochabamba**: Aguas Calientes Station, about half-way between Oruro and Cochabamba, 3506 m, 31 Jan 1949, *Brooke 5177* (BM, F); Arque, Bombeo, along the highway from Cochabamba to Oruro and La Paz, 3860 m, 7 May 2001, *Nee et al. 51755* (BM); Ayopaya, Kami on road from Pongo to Independencia. Alt. 3900 m, 10 Mar 2000, *Wood 15976* (K); **La Paz**: Murillo, entre Ovejuyo y Huni, 3990 m, 16 Aug 2007, *Aedo et al. 14739* (MA); Omasuyos, Chejepampa, 4060 m, 17 Aug 2007, *Aedo et al. 14783* (MA); La Paz, in a basin 1,500 ft. below the Altiplano plateau, 3841 m, 13 Dec 1948, *Brooke 5039* (BM, F); Saavedra, Curva, above village of Charazani, 3900 m, Apr 1978, *Carter 158* (F); Pedro Domingo Murillo, La Paz, zona de la autopista, camino detras de la fabrica Forno, 3700 m, 26 Feb 1982, *García 151* (F); near La Paz, San Pedro, 3700 m, May 1855, *Mandon 414* (BM, LE); Omasuyos, Titicaca, eastern part of village of Huatajata, near Lago Titicaca, 3820 m, 16 May 2001, *et al. 51818* (BM); La Paz, 3500 m, 19 May 1925, *Pennell 14213* (F); near La Paz, 3048 m, Apr 1885, *Rusby 797* (BM, LE); Lake Titicaca, Copacabana, 1 km east of Copacabana, 3810 m, 24 Nov 1981, *Thomas 38-3* (K); La Paz, 19 Aug 1901, *Williams 2354* (BM); Eliodoro Camacho, 10-15 km N of Ancorraimes on road to Escoma. Alt. 3800 m, 13 Jun 2000, *Wood & Wendleburger 16441* (K); Inquisivi, Camino de Quime a La Paz a unos 5-6 km arriba de Quime, 3719 m, 17 Mar 2003, *Wood & Ortuño 19422* (K, LPB); **Potosí**: San Diego, 3800 m, Aug 1932, *Cordeus 182* (CORD); Cornelio Saavedra, c. 3 km W of Karachi Pampa on road to Sucre, 4000 m, 16 Nov 1998, *Wood 14238* (K).

**Ecuador**. **Azuay**: Cantón Cuenca, Parque Nacional Cajas, road Cuencas-Sayausí-Molleturo, beyond the pass, km 47.8, 3720 m, 3 Jan 2000, *Jørgensen et al. 2067* (BM); Cantón Cuenca, Area Recreacional Cajas, carretera Cuenca-Sayausi-Tres Cruces, entre Sayausi y Quinoas, 3650 m, 16 Aug 1987, *Zak 2466* (F); carretera Cuenca-Sayausi-Area recreacional Cajas-Tres Cruces, entre el paso y Miguir, 3400 m, 16 Aug 1987, *Zak 2469* (F, S); Páramo de Soldados, at highest point of road W of Soldados, 3700 m, 3 Mar 1985, *Øllgaard et al. 58555* (BM); **Chimborazo**: Alao, Cordillera Oriental, 3200 m, 5 Feb 1944, *Acosta Solís 7172* (F); 13 km from the meeting of Carretera Whymper and Carretera Guaranda-Riobamba, 3700 m, 18 Feb 1983, *Brandbyge & Holm-Nielsen 42071* (BM); Riobamba, limite sur de la Reserva Faunistica Chimborazo, Parroquia San Juan, ceraca a la comunidad Santa Lucía, sector Cachipamba, 3400 m, 18 Jul 1992, *Cerón & Gallo 19514* (BM); **Cotopaxi**: Hacienda Sumbagua, 3700 m, 14 Nov 1939, *Haught 2934* (F); Cotopaxi, W of town, 3500 m, 19 Apr 1942, *Haught 3250* (F); **Pichincha**: Illiniza, 2 Feb 1968, *Schwabe* s.n. (B); **Tungarahua**: Cantón Ambato, Ambato, Río Ambato valley W of Ambato, 1 Nov 1952, *Fagerlind & Wibom 959* (S); Cantón Mocha, Mocha, Alrededores del Pueblo, 2900 m, 30 Sep 1995, *Villacres 282* (F).

**Peru. Ancash:** Bolognesi, Popca, Llamac y Pallca, 3350 m, 18 Jul 1998, *Cano et al. 8513* (USM); Bolognesi, Chiquián, 3890 m, 19 Apr 1949, *Ferreyra 5837* (USM); Yungay, Laguna Llanganuco, 3500 m, 2 May 1961, *Ferreyra 14349* (USM); Yungay, Laguna Llanganuco, vicinity, road from Yungay to Yauya, 3500 m, 10 Jul 1982, *Gentry et al. 37394* (MO, USM); Cordillera Blanca, Quebrada Honda, small valley between Toqllarju and Pallkaraju, 4250 m, 8 Jul 1979, *Gibby & Barrett 172* (BM); Recuay, Paracmarca, Dist. Marca, 3550 m, 18 Aug 1963, *Gómez 144* (USM); Yungay, Laguna Llanganuco, cerca del albergue, 3850 m, 31 Aug 1981, *Pérez 94* (USM); Huari, Mallas, 3490 m, 18 May 1995, *Salas Zuloaga 90* (USM); Huari, Parque Nacional Huascarán, Huascarán National Park. 4-5 km past Cahuish Tunnel, 4350 m, 23 Dec 1984, *Smith & Goodwin 8726* (MO, USM); Carhuaz, Parque Nacional Huascarán, Huascarán National Park, N-side of main valley, Quebrada Honda, 3800 m, 3 Oct 1985, *Smith et al. 11623* (MO, USM); Huari, Parque Nacional Huascarán, Quebrada Rurichinchay, between boundary and Quebrada Pachachaca, 3600 m, 11 Jun 1986, *Smith et al. 12466* (F, MO, USM); Bolognesi, Acas, 3400 m, 17 Nov 1980, *Valencia 961b* (USM); Huaylas, Caráz, Cordillera Blanca encima de Caráz, 3200 m, *Weberbauer 3252* (MOL); **Apurímac**: Abancay, Tacmara, Comunidad Tacmara, 3 horas de Distrito de Huanipaca en combi, 3200 m, 2 Apr 2009, *Daza et al. 5372* (MOL); entre Abancay y Andahuaylas, km 86-87, 3600 m, 21 Nov 1947, *Ferreyra 2792* (USM); Abancay, arriba de Abancay, 3300 m, 5 Aug 1954, *Ferreyra 9796* (USM); north of Curahuasi, 3500 m, 5 Nov 2002, *Monro et al. 3952* (BM, MOL); **Arequipa**: Arequipa, 32 km ENE of Arequipa on highway 30, at km 32, 3600 m, 23 Feb 1994, *Anderson et al. 7930* (F); Arequipa, Miraflores de Chiguata, 3300 m, 19 Feb 1999, *Cáceres & Baldárrago 813 A* (USM); La Unión, Cotahuasi, 30 Jun 2002, *Cáceres, F*., *2857* (USM); Arequipa, Nevado Chachani, ca. 20 km N of Arequipa, 3500 m, 30 Nov 1964, *Hutchison & Wright 7233* (F, LE, MO, USM); Arequipa, Simbral, carretear de Chiguata-Juliaca, 3500 m, 30 May 1999, *Roque & Betancourt 885* (USM); Pichu Pichu, 3048 m, 6 Jul 1937, *Stafford 813* (BM); Chiguata, on Arequipa-Puno road, 4050 m, 23 Oct 1963, *Straw 2314* (USM); **Ayacucho**: Lucanas, Nasca-Puquio road above Nasca (west side of pass), 3500 m, 9 Sep 1957, *Hutchison 1243* (BH, BM, LE, MO, USM); Lucanas, San Juan, on road 2 km NW of Puquio, 14 Dec 1962, *Iltis et al. 455* (USM); near Ninabamba, 3900 m, 26 May 1973, *Mullins 74* (BM); La Mar, Chilcas, arriba de Chilcas, Dist. Chilcas, 3170 m, 20 Jun 2001, *Roque & Arana 3099* (USM); Huamanga, Quebrada Marcapampa, Carretera Los Libertadores, pasando la quebrada, 14 km lineales al SO de Vinchos, Dist. Vinchos, 3650 m, 24 Jun 2001, *Roque & Arana 3247* (USM); Huamanga, below Totorobamba, 3200 m, May 1910, *Weberbauer 5483* (F, F, GH, US); **Cusco**: Cusco, Huacoto, road from San Jeronimo to Huacoto, small street to east, fields near Huacoto, 4130 m, 13 Sep 2002, *Ackermann & Salinas 297* (B, BM, USM); Tambo Machay, road from Cusco to Pisaq near ruins, 19 Apr 1983, *Bohs 2145* (F, USM); Urubamba, Ollantaytambo, Valle de Patacancha, 3500 m, May 1987, *Carter 2* (USM); Urubamba, Chincheros, road from Chinchero albergue to Ashnapuquio through community of Q’erepata, 3800 m, 19 Jan 1982, *Davis et al. 1602* (F, USM); Urubamba, Mantanay, 3350 m, 7 Sep 2002, *Farfán et al. 276* (BM, MO); Cusco, Qorao, Dist. Qorao, abra de Qorao, Bosque Seco Espinoso, 3817 m, 21 Mar 2003, *Galiano et al. 4753* (MO); Quispicanchis, Paucartambo valley, Hacienda Ccapana, 3600 m, Apr 1926, *Herrera 1070* (BM, MA); Urubamba, Maras, 3400 m, 5 May 1983, *Hoogte & Roersch 1045* (F); Mollaca, at Paso de Huillque, watershed between (at head of valley of) Anta and Limatambo, 3420 m, 23 Dec 1962, *Iltis & Ugent 777* (BM, F); Chumbivilcas, Velille, Miraflores, ca. 8 km de Velille, en el camino hacia Santo Tomás, 3700 m, 16 Apr 1987, *Núñez V. & Delgado V. 7918* (F, MO, USM); Coraupampa, between Cusco and Pisac, 3400 m, 30 Apr 1925, *Pennell 13709* (F); ca. 5 km N of Cusco on road to Pisac, 3400 m, 19 May 1977, *Solomon 2978* (F, MO, USM); Canas, Langui, Dist. Langui, Langui-Layo, 3976 m, 8 May 2003, *Valenzuela et al. 1988* (BM, MO); **Huancavelica**: Huaytará, Carretera los Libertadores, km 130, pasando la puente Yuraccasa, 3800 m, 26 Jun 2001, *Roque & Arana 3316* (USM); Huancavelica, 3798 m, 24 Mar 1945, *Soukup 2772* (F); Tayacaja, Pampas, 3250 m, 4 Jan 1939, *Stork & Horton 10241* (F); Huancavelica, 1 km N of town, 3700 m, 9 Mar 1939, *Stork & Horton 10833* (F); Huancavelica, Alauma, entre Conaica y Laria, 3400 m, 17 Mar 1952, *Tovar 786* (USM); Yauli, alrededores, 3500 m, 13 May 1958, *Tovar 3011* (USM); Tayacaja, Llamacancha, borde de camino de herredura, camino hasta Pampas, 28 Nov 1992, *Yarupaitán 368* (USM); **Huánuco**: Huánuco, Chuchos, 3200 m, 7 Sep 1980, *Huapalla 3668* (USM); Mito, 2743 m, 8 Jul 1922, *Macbride & Featherstone 1670* (F); Huamalíes, Punchao, 3600 m, 27 Mar 1999, *Ortiz Adrián 19* (USM); Huánuco, 32 km from Huanuco on Huanuco-La Union road, 2940 m, 25 Jul 1982, *Smith et al. 2182* (MO); Huánuco, Shishmay, 3000 m, 15 Sep 1937, *Woytkowski 117* (F); **Junín**: Huancayo, 1923, *Chávez* s.n. (USM); between Acopalca and Huari, NE of Huancayo, 4024 m, 19 Aug 1977, *Duncan et al. 2736* (BM); Tarma, entre Tarma y La Oroya, 3300 m, 29 Jun 1948, *Ferreyra 3796* (USM); Huancayo, El Tambo, ribera del Río Shulcas, 3217 m, Jan 1962, *García* s.n. (USM); Jauja, Yauli, 3650 m, 16 Aug 1979, *Hastorf 34* (USM); Huancayo, Pampa Cruz, 21.5 km S of Huancayo, 1 km N of Pampa Cruz, 3550 m, 28 Feb 1964, *Hutchison & Tovar 4191* (F, MO); Junín, Ulcumayo, 1 km E of town along river, 3600 m, 30 Jun 1981, *Johns 81-25* (F, USM); Tarma, San Pedro de Cajas, below town at junction of river and road, 3700 m, 5 Aug 1981, *Johns & Pearsall 81-92* (F, USM); Huancayo, 12 Feb 1927, *Juzepczuk 10708* (LE); Ocopa, 3300 m, 25 Apr 1929, *Killip & Smith 22002* (F); Jauja, Concepción, Valle del Mantaro, 3300 m, Mar 1947, *Ochoa 78* (USM); Tarma, Tarma, alrededores de Tarma y Huancayo, 2800 m, 4 Sep 1986, *Reynel 2125* (MOL); **La Libertad**: Santiago de Chuco, Los Quinuales, al norte de Quiruvilca, 3775 m, 24 Mar 1994, *Leiva G*. & *Leiva 1081* (BM, F); Huamachuco, Los Quinales, carretera Yanasara-Huaguil, 3800 m, 24 Jun 1958, *López 1398A* (USM); Pataz, Chirimachaj, 3450 m, 24 Feb 1986, *Young 3002* (MOL, USM); **Lima**: Huarochiri, Huachupampa, 3500 m, 28 Aug 1993, *Albán & Yarupaitán 8078* (USM); Yauyos, Laraos, *Beltrán 388* (USM); Huarochiri, Kolpaykunko, cerca a San Lorenzo, Dist. de Langa, 3660 m, 15 Apr 1968, *Cerrate et al. 4846* (USM); Huarochiri, Chicla, km 113, 3800 m, 30 Jul 1972, *Cerrate 5335* (USM); Lima, along highway between Lima and La Oroya, western slope of Cordillera Occidental, 3 km E of Bellavista, 5.4 km E of Aucla, vicinity of km marker 113, 3950 m, 10 Jun 1998, *Croat & Sizemore 82018* (BM x2, MO, USM); Cajatambo, Oyon, May 1948, *Ferreyra 3536* (USM); Huarochiri, Chicla, 3850 m, 30 Apr 1995, *Llatas Q. et al. 3735* (USM); Huarochirí, R side of Central Valley 117 km from Lima, Dist. of San Mateo, 3567 m, 22 May 1960, *Saunders 490* (BM, F); Canta, Huamantinga, 3400 m, 8 May 1974, *Vilcapoma 119 -2* (USM); Canta, Lachaqui, 3700 m, 21 Dec 1972, *Vilcapoma 119* (USM); **Moquegua**: Moquegua, Cocotea, 3500 m, 5 Sep 1997, *Albán & Malca 10081* (USM); Mariscal Nieto, Cuajone, just above mine, 3700 m, 15 Feb 1983, *Dillon & Matekaitis 3374* (BH, F, USM); General Sánchez Cerro, Tassa, faldas del cerro Ccalo-ccalo, Dist. Ubinas, 3530 m, 14 Sep 2005, *Montesinos 558* (USM); General Sánchez Cerro, Yunga, sendero del puente colgante, 3560 m, 14 Sep 2005, *Montesinos 567* (USM); **Pasco**: entre Huariaca y Cerro de Pasco, 3800 m, 25 Jun 1953, *Ferreyra 9501* (USM); 95 km from Huánuco on road to Cerro de Pasco, 3590 m, 15 Jul 1982, *Gentry et al. 37500* (F, MO, USM); Yanamachay, bajando de Cerro de Pasco hacia Quinua, 3900 m, 1 Apr 1948, *Ochoa 328* (CORD); Pasco, road from Colquijirca to La Quinua, 26 km NE of Colquijirca, 3680 m, 5 Dec 1981, *Plowman & Rury 11074* (BH, F, USM); La Quinua, 3450 m, Dec 1986, *Rivas et al.* s.n. (USM); 22 km N of Cerro de Pasco on road to Huánuco, 30 Sep 1984, *Whalen 843* (BH, USM); **Puno**: Sicuani, 3550 m, 15 May 1920, *Herrera 19* (SI); Huancané, Moho, 3900 m, 2 Mar 1982, *Hoogte & Roersch 2091* (F); Puno, Lago Titicaca, 4000 m, 13 Jul 1954, *Manheim 71* (F); Lampa, Lamparequen, 4130 m, 6 Jun 2009, *Montesinos & Pinto 2670* (USM); Titicaca, Lake Hotel, 22 Jan 1975, *Schwabe* s.n. (B); Puno, 4000 m, Jul 1936, *Soukup 360* (F); Sicuani, Yauri to Cuzco, near Sicuani, 3000 m, 14 Jul 1967, *Tessene & Vargas* s.n. (USM); cerros de Puno, 3800 m, 12 Dec 1961, *Tovar 3522* (USM); Puno, 10 km SW on road to Llave, 3822 m, 13 May 1963, *Ugent & Ugent 5256* (USM); **Tacna**: Tarata, Poma, carretera Tarata-Puno, 3400 m, 25 Mar 1998, *Cano 8062* (USM); Tarata, Estique Palma, 3200 m, 26 Sep 1980, *Müller 3688* (USM).

#### 28. Solanum odoriferum

Vell., Fl. Flumin. 85. 1829

http://species-id.net/wiki/Solanum_odoriferum

Figure 69

Solanum pachyantherum Witasek, Kaiserl. Akad. Wiss. Wien, Math.-Naturwiss. Kl., Denkschr. 79: 331. 1910. Type: Brazil. São Paulo: near São Paulo, Pilar, 750-800 m, *M. Wacket* s.n. (lectotype, designated here: WU [WU0037997, F neg. 30886]; isolectotype: WU [WU0037798]).

##### Type:

Brazil. Rio de Janeiro: Sin. loc., *Anon*. (no specimens extant; lectotype, designated by Morton 1976, pg. 61: Vellozo, Fl. Flumin. Icones 2, tab. 108. 1831).

##### Description.

Woody vine. Stems glabrous and shining; new growth glabrous, minutely papillose. Bark of older stems greenish brown, shiny. Sympodial units plurifoliate. Leaves simple, (4-)7–11 cm long, 1.5–5.5 cm wide, elliptic, coriaceous, both surfaces glabrous and shiny; primary veins 7–9 pairs, the secondary venation fine and strongly parallel between the primary veins; base truncate to obtuse and slightly decurrent on the petiole; margins entire, revolute; apex abruptly acute to acuminate; petioles 1.3–3.5 cm, glabrous, twining around supports and aiding in climbing. Inflorescences terminal, 1–13 cm long, many times branched, with up to 100 flowers, completely glabrous; peduncle 0.5–1 cm long; pedicels 1–1.3 cm long, filiform, < 0.5 mm in diameter at base and apex, spreading at anthesis, glabrous, articulated at the base in a small sleeve, leaving a tiny peg at abscission; pedicel scars irregularly spaced near the tips of inflorescence branches, to 5 mm apart in fruit. Buds turbinate with an apical nipple, the corolla far exserted from the calyx tube long before anthesis. Flowers all perfect, 5-merous. Calyx tube 2–2.5 mm long, shallowly cup-shaped, the lobes < 0.5 mm long, usually forming only an irregularly and shallowly lobed hyaline rim to the tube with tiny apiculae < 0.5 mm long, glabrous, the apiculae with a few papillae. Corolla 1.8–2 cm in diameter, white, fragrant (fide Mentz and Oliveira 2004), stellate, lobed nearly to the base, the lobes 7–9 mm long, 3–3.5 mm wide, planar or spreading at anthesis, the tips cucullate and minutely papillate along the distal portion, otherwise glabrous. Filament tube minute, the free portion of the filaments 1–1.5 mm long, glabrous or minutely papillate; anthers 4.5–5 mm long, 1–1.5 mm wide, ellipsoid, loosely connivent, poricidal at the tips, the pores lengthening to slits with age. Ovary glabrous; style 8–9 mm long, glabrous; stigma clavate, slightly bilobed, the surface minutely papillose. Fruit a globose berry, 1–1.2 cm in diameter, black when ripe, the pericarp thin and shiny, glabrous; fruiting pedicels 1–1.3 cm long, ca. 1 mm in diameter at the base, woody, pendent or spreading. Seeds 10–20 per berry, flattened reniform, 3.5–4 mm long, 2.5–3 mm wide, pale yellowish brown, the surfaces minutely pitted, the testal cells rectangular. Chromosome number: not known.

**Figure 69.**
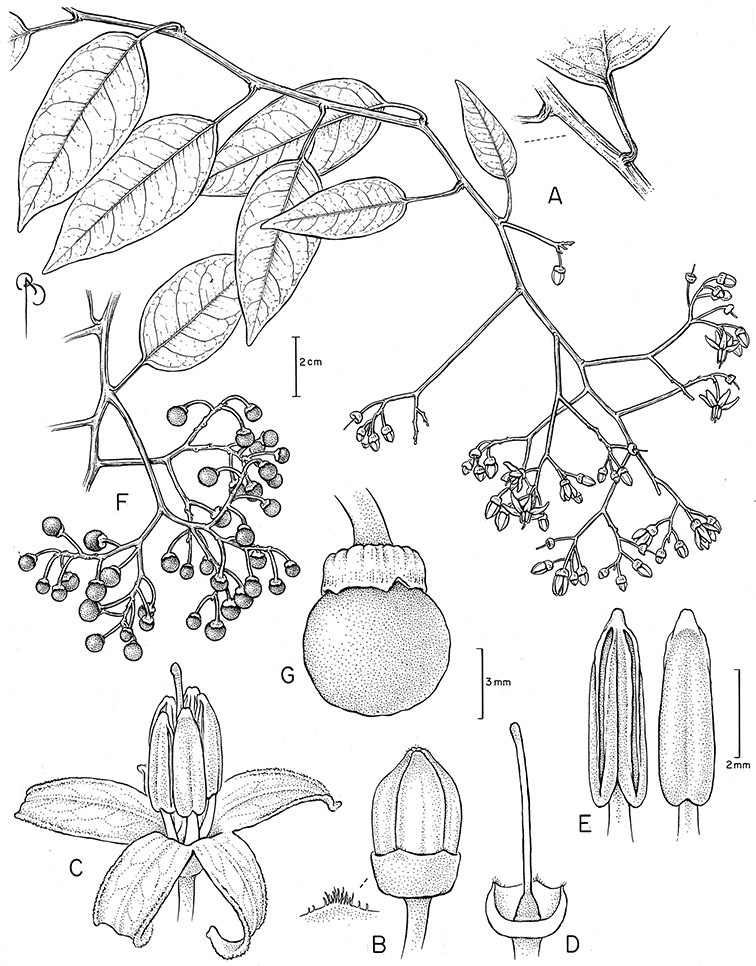
*Solanum odoriferum* Vell. (**A–E** drawn from *Hatschbach 20204* **F–G** drawn from *Reitz & Klein 6343*). Illustration by Bobbi Angell.

##### Distribution

**Figure 70.**
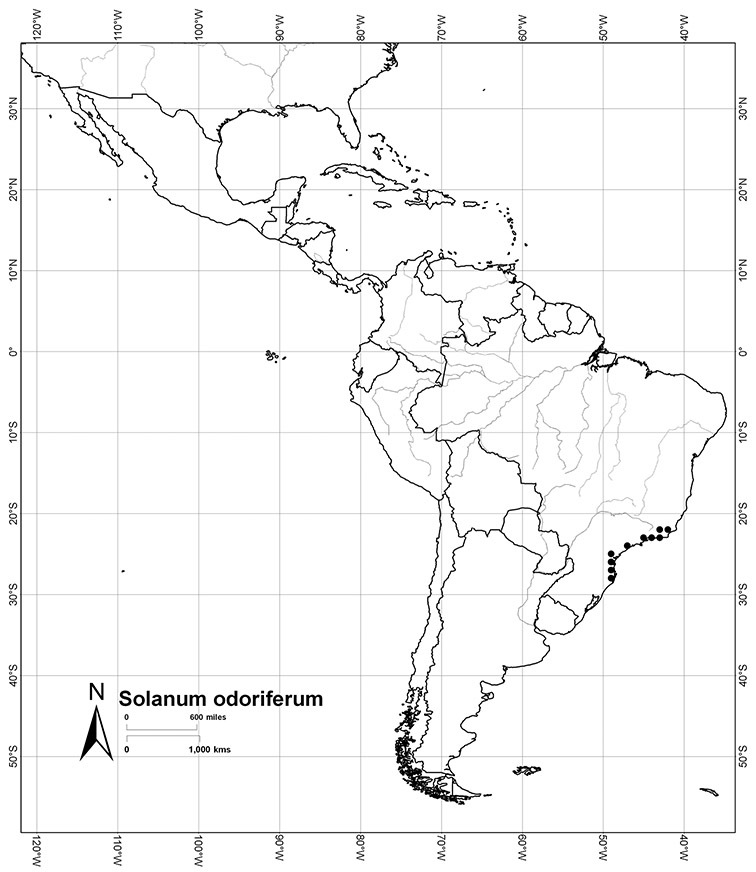
Distribution of *Solanum odoriferum* Vell.

(Figure 70). In the Atlantic rainforests and seasonal deciduous forests of southeastern Brazil from the states of São Paulo to Rio Grande do Sul at elevations from 0–750 m. The species has only been collected once from Misiones, Argentina (Ekman 1975) despite much botanical work in the area, it is likely to come from a cultivated plant but information to that effect is not on the label.

##### Common names:

Brazil. Santa Catarina: joá-cipó-cheiroso (Smith and Downs 1966).

##### Conservation status.

Least Concern (LC); EOO >100,000 km^2^ (LC) and AOO >10,000 km^2^ (LC). See Moat (2007) for explanation of measurements.

##### Discussion.

*Solanum odoriferum* is a striking plant, with its large, multi-flowered inflorescences of aromatic flowers. It is superficially similar to the sympatric *Solanum flaccidum* but is easily distinguished from that species by its coriaceous, shiny, glabrous leaves, truncate calyx and anthers on filaments of equal length.

**Figure 71.**
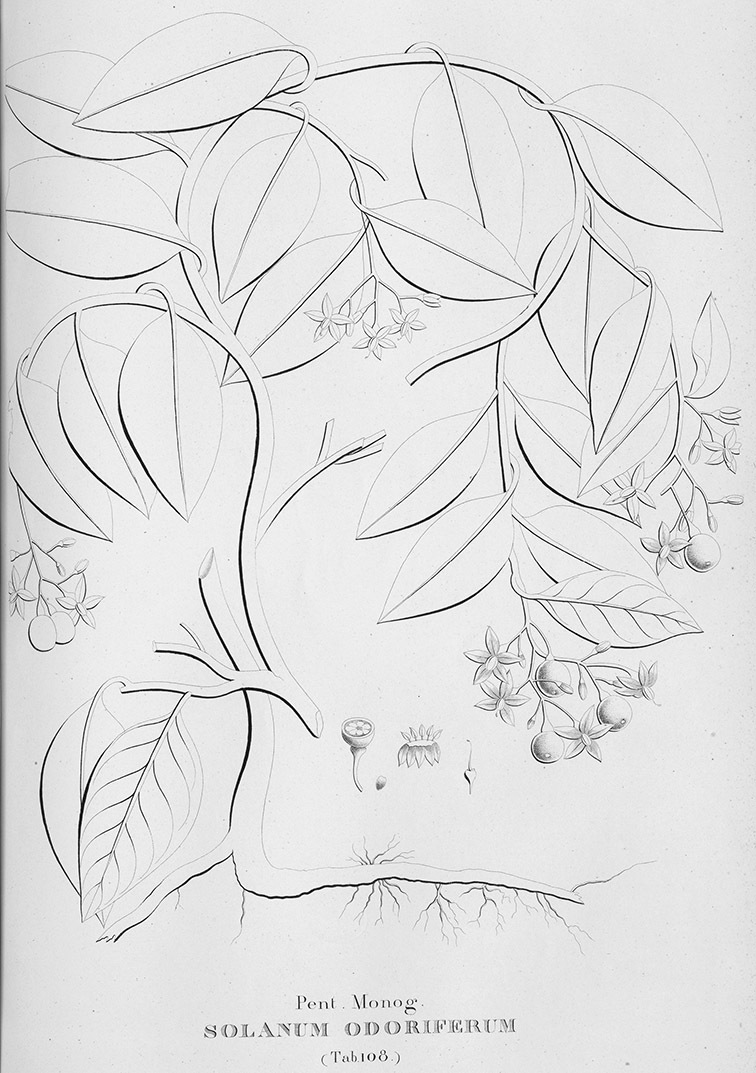
Lectotype of *Solanum odoriferum* Vell. (Vellozo 1831: tab. 108) Reproduced with permission of the Natural History Museum Botany Library.

The illustration in Vellozo (1831) leaves no doubt as to the identity of this species (see Figure 71), and was selected as lectotype by Morton (1976) since no specimens associated with this work have ever been recovered (Carauta 1973). I have lectotypified *Solanum pachyantherum* with the better of two duplicates of *Wacket* s.n. from the type locality at WU (WU0037997).

##### Specimens examined.

**Argentina. Misiones:** Posadas, Bonpland, 1907, *Ekman 1975* (NY).

**Brazil**. **Minas Gerais**: Tejuco, *Schott* s.n. (F); **Paraná**: Alexandra, 5 Jul 1909, *Dusén 8620* (F, S); Morretes, 4 Jan 1914, *Dusén 14277* (F, GH, S); Campina Grande do Sul, Rio Pardinho, 21 Nov 1965, *Hatschbach 13166* (F, P, US); Guaraqueçaba, Rio do Cedro, 20 m, 21 Nov 1968, *Hatschbach 20368* (F); Serra do Mar, Porto do Cima, 200 m, 11 Jul 1914, *Jönsson [Dusén] 658a* (GH, S); Piraquara, Purgatorio, 22 Dec 1981, *Kummrow 1619* (MA); São José dos Pinhais, Serra dos Castelhanos, 22 Dec 1987, *Silva & Hatschbach 451* (US); **Rio de Janeiro**: Petrópolis, Gularte, 6 Nov 1892, *Glaziou 14176* (G, K, LE, P); Alto Macahé, 8 Aug 1895, *Glaziou 18407* (P); Organ Mount, Imbuby lane, Feb 1838, *Miers* s.n. (BM); Tejuco, *Schott 5414* (LE); Serra dos Órgãos, 7 Jan 1883, *Schwacke 4328* (GOET); **Santa Catarina**: Itajaí, Morro da Fazenda, 300 m, 25 Mar 1954, *Klein 766* (US); Florianópolis, Tapera, Ribeirão, 150 m, 17 Dec 1968, *Klein & Bresolin 8013* (US); Florianópolis, Lagoa de Peri, 5 m, 1 Apr 1970, *Klein et al. 8662* (US); Itajaí, Morro da Ressacada, 350 m, 29 Mar 1956, *Reitz & Klein 2922* (G, US); Sabía, Vidal Ramos, 750 m, 29 Jan 1958, *Reitz & Klein 6343* (US); **São Paulo**: Pitagueiras, in regione basali montis Serra do Mar, 6 Oct 1912, *Dusén 14210* (F, S); Ubatuba, 4 May 1892, *Edwall 1816* (US); Cantareira, Horto Botanico, 26 Jul 1902, *Hammar 5865* (US); Rio Preto, 5 Nov 1891, *Loefgren 1685* (US); Santos, Sororocaba, 25 Dec 1874, *Regnell 3029* (S).

#### 29. Solanum pittosporifolium

Hemsl., J. Linn. Soc. 26: 171. 1890

http://species-id.net/wiki/Solanum_pittosporifolium

Figure 72

Solanum dulcamara L. var. *heterophyllum* Makino, Bot. Mag. (Toyko) 24(276): 19. 1910. Type: Japan. Sin. loc. (no specimens cited; none located; synonymy ex descr.).
Solanum japonense Nakai, Fl. Sylv. Kor. 19: 58. 1923. Type: Based on *Solanum dulcamara* L. var. *heterophyllum* Makino
Solanum nipponense Makino, J. Jap. Bot. 3: 20. 1926. Type: Based on *Solanum dulcamara* L. var. *heterophyllum* Makino
Solanum takaoyamense Makino, J. Jap. Bot. 3: 38. 1926. Type: Japan. Hondo: prov. Musashi, Mt. Takao, 1926, *T. Makino* s.n. (holotype: TI?, herb. Makino?).
Solanum schiffnerianum Witasek, Oesterr. Bot. Zeitschr. 80: 163. 1931. Type: Indonesia. Sumatra: Mt. Merapi, 1200 m, 30 Jul 1894, *V. Schiffner 2506* (lectotype, designated here: L [L0003663]; isolectotype: L [L0003664]).
Solanum hidetaroi Masam., Trans. Nat. Hist. Soc. Taiwan 29: 84. 1939. Type: Taiwan [China]. Taihokusyu, Pianan-Kirittoi, 13 Jul 1931, *G. Masamune & K. Mori 1630* (holotype: TI [n.v.]; isotype: PE [PE00031402]).
Solanum pittosporifolium Hemsl. var. *pilosum* C.Y.Wu & S.C.Huang, Acta Phytotax. Sin. 16(2): 72. 1978. Type: China. Yunnan: Weixi, ca. 2500 m, *Guomei Feng 4840* (holotype: KUN; isotype: PE [PE00031396]).

##### Type.

China. Sichuan [Szechuan]: Mount Omei, 5000 ft, *E. Faber* s.n. [*252*] (holotype: K [K000658076]; isotype: NY [NY000172264]).

##### Description.

Small shrub or woody vine, if shrubby, plants usually scandent or trailing to 2 m long. Stems glabrous, slightly ridged, thin and flexuous; new growth glabrous or with a few glandular trichomes. Bark of older stems pale brown. Sympodial units plurifoliate. Leaves simple, 5–14 cm long, 4–5 cm wide, narrowly elliptic, membranous, the upper surfaces glabrous or minutely pubescent with simple uniseriate trichomes < 0.5 mm long, these 1–2-celled, the lower surfaces glabrous or with simple uniseriate trichomes along the midvein; primary veins 5–7 pairs, somewhat impressed above; base attenuate, decurrent onto the petiole; margins entire, slightly undulate; apex acuminate or occasionally acute; petiole 0.5–3 cm long, glabrous of with a few 1–2-celled simple uniseriate trichomes, the occasionally minutely glandular. Inflorescences terminal or lateral, 2–10 cm long, open and usually many times branched, with 10–40 (+) flowers, glabrous; peduncle 2–5.5 cm long; pedicels 6–11 mm long, ca. 1 mm in diameter at the apex, ca. 0.5 mm in diameter at the base, spreading, glabrous or minutely pubescent with simple glandular trichomes, articulated at the base, leaving a short peg 1.5–2 mm long; pedicel scars irregularly spaced 2–10 mm apart, more closely spaced distally. Buds ellipsoid, the corolla strongly exerted from the calyx tube before anthesis. Flowers all perfect, 5-merous. Calyx tube ca. 1 mm long, the lobes 0.5–1 mm long, deltate to triangular, glabrous. Corolla 1–1.4 cm in diameter, white to violet with green spots at the base of each lobe making a green eye, stellate, lobed ca. 3/4 of the way to the base, the lobes 5–6 mm long, 2–3 mm wide, reflexed at anthesis, minutely papillose at the tips and margins, otherwise glabrous. Filament tube < 0.5 mm long, the free portion of the filaments 0.5–0.75 mm long, glabrous; anthers 3–4 mm long, 1–1.5 mm wide, ellipsoid, poricidal at the tips, the pores lengthening to slits with age. Ovary glabrous; style 5–7.5 mm long, glabrous; stigma capitate, the surface minutely papillate. Fruit a globose berry, to 1 cm in diameter, bright red when ripe, juicy, the pericarp thin and shiny; fruiting pedicels 1.2–1.4 cm long, 0.5–1 mm in diameter at the base, not markedly woody, spreading. Seeds > 30 per berry, ca. 3.5 mm long, 2.5 mm wide, flattened reniform, pale yellow, the surface with “hairs” ca. 0.5 mm long from the lateral cell walls. Chromosome number: not known.

**Figure 72.**
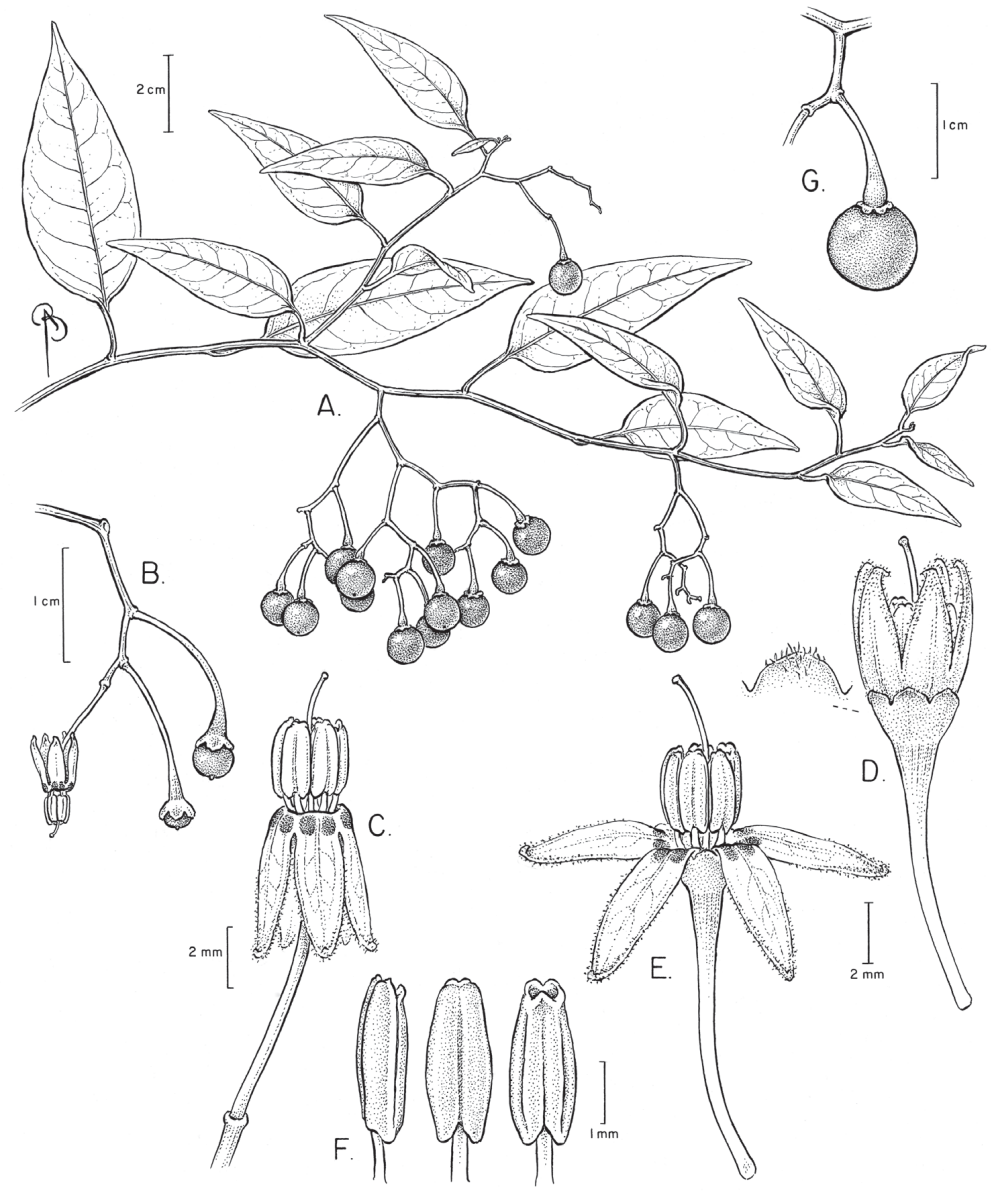
*Solanum pittosporifolium* Hemsl. (**A** drawn from *Li 96079* **B–C** drawn from *Wan et al. 1824* **D–F** drawn from *Zhou 060* **G** drawn from photographs of *Knapp 10136*). Illustration by Bobbi Angell.

##### Distribution

**Figure 73.**
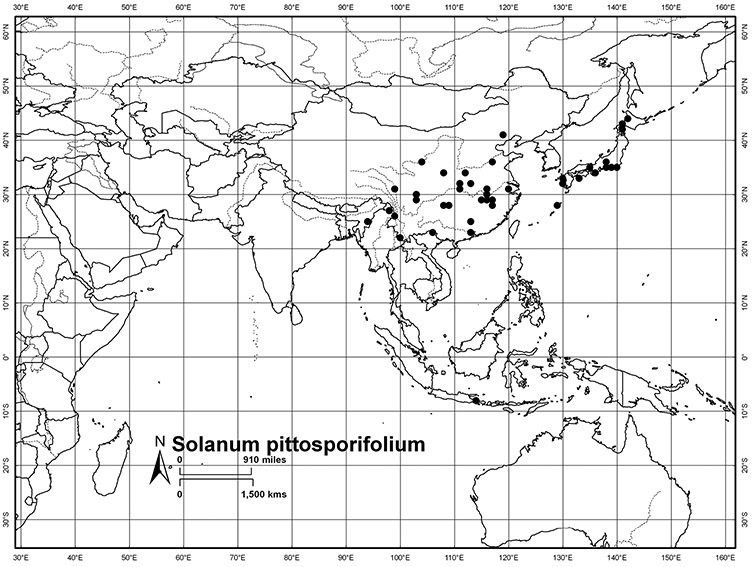
Distribution of Solanum*pittosporifolium* Hemsl.

(Figure 73). *Solanum pittosporifolium* is found at a wide variety of elevations (300–1500 m) from Bhutan and the Kashmir region at the India/Pakistan border through China to Japan, Vietnam and the island of Sumatra in Indonesia.

##### Ecology.

Relatively common in disturbed habitats such as roadsides and riverbeds; often growing at the edge of trails.

##### Common names.

China: tai bai ying, ye hai qie, hai tong ye bai ying (Zhang et al. 1994).

##### Conservation status.

Least Concern (LC); EOO >100,000 km^2^ (LC) and AOO >10,000 km^2^ (LC). See Moat (2007) for explanation of measurements.

##### Discussion.

*Solanum pittosporifolium* is widely distributed throughout temperate and subtropical Asia and is somewhat weedy. It has a more southerly and westerly distribution than does *Solanum lyratum*; it extends south to the Indonesian island of Sumatra and into the frontier area of India and Pakistan, where it is sympatric with *Solanum dulcamara*. In the treatment of *Solanum* for Flora of China (Zhang et al. 1994) many of the names here treated as synonyms of *Solanum pittosporifolium* were recognised at the specific level and considered to be “doubtfully distinct from *Solanum dulcamara*”.The two species can be very difficult to distinguish when in fruit, but *Solanum pittosporifolium* has shorter and sparser pubescence than does *Solanum dulcamara*, and is usually a more delicate plant. The leaves of *Solanum pittosporifolium* are never divided, and usually have undulate margins; those of *Solanum dulcamara* are more variable, but usually have more acute bases than those of *Solanum pittosporifolium*. In flower, the two are easily separated – *Solanum pittosporifolium* as elliptic anthers that are not held tightly together and the buds are ovoid or ellipsoid, never turbinate like those of *Solanum dulcamara*.

The leaf pubescence of *Solanum septemlobum* is similar to that of *Solanum pittosporifolium*, but the two species can be distinguished by flower size (those of *Solanum septemlobum* are larger and the petals are usually not reflexed) and by the shorter, stiffer trichomes of *Solanum pittosporifolium*. In general, *Solanum pittosporifolium* is a more tropical plant than is *Solanum septemlobum*, although they do overlap in central China.

When *Solanum pittosporifolium* grows along roadsides and is cut back regularly it appears to be a shrub, but if left to extend its branches it becomes a flexuous vine. I have collected it growing in river courses where it seems to be behaving as a rheophyte, but populations outside of this sort of habitat have identical almost glabrous, narrow leaves.

Both syntypes of *Solanum pittosporifolium* encountered are labelled as *Faber 252*, but as Hemsley worked at Kew at the time he described this species it is clear the K sheet is that used by him, not the sheet at NY. Of the two sheets of *Schiffner 2506* held at L, I have chosen L0003663 as the lectotype for *Solanum schiffnerianum* as it has a complete locality label and is annotated in Witasek’s hand.

##### Specimens examined.

**Bhutan**. Ruhubje Dorysa, 2591 m, 7 Oct 1914, *Cooper 2318* (BM, E).

**Burma**. Ukhrul, 1676 m, 27 Aug 1948, *Kingdon-Ward 18004* (NY).

**China**. **Anhui**: Wangshan, Wang Shan, 20 Jul 1925, *Ching 8653* (K, US); Wangshan, 1973, *Chow 92* (A, K, L); Chiu-Hua Shan, 500 m, 3 Jul 1934, *Fan & Li 29* (K); Yaoluoping, Yuexi County, 950 m, 14 Jul 2002, *Zhou 060* (K, MO); **Fujian**: Mount Wuyi, Wuyi Mt, 1100 m, 9 Aug 1980, *Wuyi Shan Team 80-0635* (MO); **Gansu**: Lanzhou City, Anningshilidian. Lanzhou city, 1500 m, 1 Sep 1996, *Lian 96964* (MO); **Guangdong**: Daling, Jiaolankeng, Daling, Ruruan County, 10 Sep 1935, *Chen 11023* (MO); Ruyuan County, Xishan, Ruyuan County, 1400 m, 23 Nov 1957, *Deng 5778* (MO); Fan Shiu Shan, Fan Shiu Au and vicinity, Wung Yuen district, 1 Nov 1933, *Lau 2738* (GH); **Guangxi**: Damiaoshan, 600 m, 28 Jul 1958, *Chen 15919* (MO); Jinwanshan, Pingshixiang, Sanfang district, Damiaoshan County, 800 m, 9 Aug 1958, *Chen 16227* (HITBC, MO); Yuanbaoshan, Xiaosangxiang, Antai district, Damiaoshan County, 9 Nov 1958, *Chen 17170* (MO); Hai-Chang Village, Ch’i-fen-shan, Kwei-lin District, 24 Sep 1937, *Tsang 28384* (A, US); Hai-Chang Village, Ch’i-fen-shan, Kwei-lin District, 24 Sep 1937, *Tsang 28400* (A, US); Yao Shan, 1524 m, 18 Oct 1936, *Wang 40268* (A); Qingbao County, Huanglianshan, Qingbao County, 1400 m, 2 Dec 1958, *Zhang 13621* (MO); **Guizhou**: Jiangkou Xian, Heiwan River on the SE side of the Fanjing Shan mountain range in the vicinity of the Ecological Station of the Guizhou Academy of Sciences, 560 m, 20 Aug 1986, *Sino-American Guizhou Bot. Exp. 90* (L); Songtao Xian, Lengjiaba, vicinity of confluence of the Xiaohe and Dahe Rivers, NE side of the Fanjing Shan mountain range, 820 m, 5 Oct 1986, *Sino-American Guizhou Bot. Exp. 2225* (A); Fanjing Mountain, 520 m, Nov 1985, *Sino-British Plant Exped. Team*, *0013* (K); **Henan**: Luoning, *Liu Miao H-30193* (HITBC); Mangchuan, *Liu Miao H-40091* (HITBC); Taibaiding, Tangbai County, 700 m, 1 Aug 1985, *South Team T-0265* (MO); **Hubei**: Shennongjia Forest District, S end of the Loyang River Gorge near Pingqian, 1300 m, 14 Sep 1980, *Bartholomew et al. 1278* (HIB); Yichang, Ichang. Nan-t’o and mountains to northward, Feb 1887, *Henry* s.n. (K); Yichang, Ichang and immediate neighbourhood, Feb 1887, *Henry 2334A* (GH, K); Shennongjia Forest District, S end of Loyang River gorge near Pingqian, 1300 m, 14 Sep 1980, *Sino-American Expedition 1278* (A); Kikungshan, 30 Jul 1925, *Steward 9742* (US); Yang-kia-ping, Jul 1934, *Wang 61841* (A); **Hunan**: Yunshan, prope urbem Wukang, templum Gwanyingo, 1250 m, 5 Aug 1918, *Handel-Mazzetti 12401* (GH); Wuli’an, Yunshan, Wugang County, 720 m, 3 Oct 1963, *Liu & He 16141* (MO); Yizhang, Yizhang County, 1300 m, 23 Oct 1942, *Xu 2799* (MO); **Jiangsu**: Yu-hewnag-tien, Hwang-lung Shan, 20 Aug 1947, *Hsiung 5576* (A); Nanjing, Jiangpu District, Laoshan, Tianlin, 1300 m, 27 Nov 1957, *Zhang 11070* (); **Jiangxi**: Lushan, Douyouping, Lushan mountain, E slope, 1075 m, 27 Sep 2007, *Knapp et al. 10136* (BM); Lushan, Wufeng, (Five Old Man Peaks), Lushan Mountain, E slopes, 1191 m, 28 Sep 2007, *Knapp et al. 10140* (BM); Jiujiang, Lushan, Lushan, 190 m, 1 Oct 1994, *Tan 94-1079* (MO); Wuningyan, Wuning County, 500 m, 21 Oct 1994, *Tan 94-1179* (MO); Luoxi, 1300 m, 31 Aug 1996, *Tan 96-08072* (MO); De An Xian, 200 m, 8 Nov 1996, *Tan 11042* (B); **Shaanxi**: sin. loc., 1600 m, 21 Aug 1957, *Anonymous* s.n. (HIB); **Shandong**: Sishui, *Guo Chengyong 54428 -5* (HITBC); **Shanxi**: Taibai Mountain, 1300 m, 9 Oct 1990, *Liu 90-103* (A); **Sichuan**: Dujiangyan, Qinglongzui, Dujianyan Municipality (formerly Guan Xian), Qinglongzui near the site of Longwangmiao, along the Longxi River, 1900 m, 9 Sep 1988, *Boufford et al. 24748* (A, BM); Baiyu Xian, W of Baiyu along Ou Qu (Ou River) then N along the Jinsha Jiang (upper Chang Jiang) toward Dege, 2965 m, 21 Aug 2006, *Boufford et al. 36963* (A); Mount Emei, Emei [Omei] Mt, 1020 m, 1 Dec 1996, *Li 96-218* (MO); Tsa-wa-rung, Me-kong, 3000 m, 1935, *Wang 66159* (A); **Tibet**: Ta Tsien Lou, (Principauté de Kiala), Thibet Oriental, 20 Jun 1893, *Soulié 753* (B); **Yunnan**: Ma-jo, 1907, *Cavalerie 3147* (K); Gaoligong Shan Region. Gongshan Xian, Dulongjiang Xiang. In the vicinity of Maku, southern region of the Dulong Jiang valley on the W side of the Dulong Jiang, 1680 m, 14 Dec 1990, *Dulong Jiang Investigation Team 1003* (CAS); Wu-too, on Yangtze, S. Chungtien, 2550 m, 13 Nov 1939, *Feng 3343* (A); Mingkwong valley, 1829 m, Jun 1912, *Forrest 8349* (K); Gaoligong Shan Region, Fugong Xian, Lumadeng Xiang. Yaping Cun, along road above old Shibali on N side of S fork of Yamu He, E side of Gaoligong Shan, 2540 m, 22 Aug 2005, *Gaoligong Shan Biodiversity Survey 28858* (CAS, GH); Luzhang, Nujiang Lisu Aut. Pref., Lushui Co., 3 km east of Yao Jia Ping, along road to Lu Zhang, 2440 m, 25 Oct 1996, *Gaoligong Shan Expedition 1996 8039* (E); Xishuangbanna, Menghai, *Tao Guoda 40024* (HITBC); A-tun-tze, 2700 m, 1935, *Wang 70253* (A); Nujiang, Lushui, *Wu Sugong 8066* (HITBC); **Zhejiang**: Mokanshan, 13 Sep 1934, *Read R-1190* (BM).

**India**. **Jammu and** **Kashmir**: Kishtwar, Kashmir, 1829 m, 17 Sep 1878, *Clarke 31365* (LE); below Nagkanda, Aug 1849, *Fleming* s.n. (E); Nara Nag, Wangat Nullah, 2100 m, 10 Sep 1956, *Polunin 56/776* (B, E, F); **Sikkim**: Choongtam, Oct 1827, *Anonymous* s.n. (K); Rumman [Vos], 1829 m, Nov 1881, *Gamble 10042* (K); Daquili, 2134 m, Aug 1882, *Gamble 10557* (K).

**Indonesia**. **Sumatra**: W. Kust, G. Koerintzi, 2200 m, 6 May 1920, *Brummemeyer 10103* (L).

**Japan.** **Hokkaido**: Sapporo, prov. Ishikari, 15 Oct 1890, *Tokukuchi* s.n. (GH); **Honshu**: Mt. Tanzawayama, Sagami: Mt. Tanzawayama, 19 Aug 1910, *Anonymous* s.n. (US); Nagano, Mt. Shizumo, Kiso-gun, Nigiso-machi, 500 m, 18 Nov 1980, *Boufford et al. 23332* (A); Tochigi, Mt. Hiruga-take, to half way up from foot of mountain, prov. Shimotsuke, Shiwobara-machi, Shiwoya-gun, 800 m, 8 Jul 1958, *Furuse* s.n. (A); half-way up Mt. Hiruga-take, Shiwobara-machi, Shiwoya-gun. Province Shimotsuke. Prefecture Tochigi, 800 m, 8 Jul 1958, *Furuse 33992* (K); to Koshibu-yu from up stream Koshibu-river, Ohjika-son Shimo-ina-gun, Prefecture Nagano. Province Shinano, 1100 m, 4 Aug 1966, *Furuse 44346* (K); Fara range above Hegi, 13 Aug 1937, *Hara N-82-37* (K); Shizuoka, Mt. Kenashi-yama, Fujinomiya-shi, Fumoto, 870 m, 3 Jul 1994, *Kobayashi 2590* (A); Shizuota, Tagata-gun, Amagiyugashima-machi, Joren-no-taki Fall, 280 m, 14 Sep 1994, *Kobayashi 2700* (A); Shizuoka, Fumoto, Fujinomiya-shi, 900 m, 30 Oct 1994, *Kobayashi 2802* (A); Ecchu, Mt. Tateyama, 31 Aug 1932, *Maekawa 261* (BH); Shinano, Shidzumo National Forest, Okuwa-mura, Kiso, 23 Aug 1954, *Mizushima 12414* (S); Nara, Wasamata-dani, Kamikitayama-mura, Yoshino-gun, 700 m, 22 Sep 1986, *Murata 67293* (A); Owari, 15 Aug 1929, *Shiota 4805* (GH); Izumi, 15 Nov 1931, *Shiota 5417* (A); prov. Mino, 14 Oct 1933, *Shiota 7242* (A); Kii, Mt. Koya, route from Daimon to Yatate, 700 m, 9 Sep 1962, *Tamura 9361* (S); Nara, Mt. Wasamata, N slope, Kami-kitayama-mura, Yoshino-gun, 1200 m, 20 Sep 1986, *Tsugaru et al. 7400* (A); Asakogun, Hyoyoken, 17 Aug 1939, *Uno 24095* (GH); Shizuoka, Mt. Ogasa-yama, Fukuroi-shi, 250 m, 21 Oct 1999, *Yamashiro 7030* (A); Aichi, Mt. Horaiji-san, Minamishitata-gun, Horaiji-cho, Toshuguu-Okuoin, 22 Oct 1999, *Yamashiro 7041* (A); Nagasaki, Kuroki-Yokomine Pass, Ohmura-shi, Kuroki-cho, 390 m, 20 Aug 1995, *Yonekura 95952* (A); **Kyushu**: Kagoshima, Mt. Shibi, Miyanojo, 20 Oct 1958, *Hara 2771* (A); **Ryuku**: Ryuku Islands, Amamioshima, Mt. Yuwan, 5 Nov 1927, *Saito 3289* (A, BM); **Shikoku**: Tosa, Nanokawa, 11 Jul 1892, *Anonymous* s.n. (US).

**Republic of Korea**. Korean Archipelago, Jul 1863, *Oldham 1039* (K).

**Taiwan**. Alishan, Arizan, Jan 1914, *Faurie 1480* (A, BM).

**Vietnam**. **Cao Bang**: Distr. Nguyen Binh, municipality Ca Thanh, vicinity of Ca Lu village, about 7-8 km to SE from Yen Lac village, about 38 km to NWW from Cao Bang town, 1350 m, 21 Nov 1998, *Averyanov CBL-552* (MO); **Tonkin**: Chapa, 1500 m, Aug 1940, *Pételot 2604* (A).

#### 30. Solanum pubigerum

Dunal, Hist. Nat. Solanum 160, tab. 6. 1813

http://species-id.net/wiki/Solanum_pubigerum

Figure 74

Solanum leptanthum Dunal in Poir., Encycl. Meth. Suppl. 3: 747. 1814. Type: Mexico. Based on an unpublished illustration in the Sessé and Mociño collection (lectotype, designated here: Hunt Botanical Institute 6331.0673).
Solanum cervantesii Lag., Gen. Sp. Pl. 10. 1816. Type: Spain: Cultivated at the Madrid Botanical Garden, originally from Mexico, *Anon*. (lectotype, designated here: MA [MA-308287]; possible isolectotype: G [G00070132]).
Solanum modestum Roem. & Schult., Syst. Veg. ed. 15 bis [Roemer & Schultes] 4: 663. 1819. Type: Mexico. Sin. loc., *Anon*. (holotype: B-W [B-W04373-01 0]).
Solanum glabrum Dunal, Prodr. [A.P. de Candolle] 13(1): 102. 1852. Type: Mexico. Hildago: “prope Morán, Regla et Omitlán”, *A. Humboldt & A. Bonpland* s.n. (lectotype, designated here: P [P00136343]).
Solanum martensii Dunal, Prodr. [A.P. de Candolle] 13(1): 140. 1852. Type: Mexico. Oaxaca: “Yaveziae”, *H. Galeotti 1227* (no herbaria cited; lectotype, designated here: G [G00070133]).
Solanum lineatum Sessé & Moc., Fl. Mex. ed. 2, 51. 1894, nom. illeg., non *Solanum lineatum* Ruiz & Pav., 1799. Type: México. Distrito Federal: Pedregal del San Angel, oeste de volcán Xitle, *M.A. Panti Madero 155* (neotype, designated by Knapp, 2008b, pg. 15: MEXU [MEXU-357951]).
Solanum lineatum Sessé & Moc., Fl. Mex. ed. 2, 53. 1894, nom. illeg., non *Solanum lineatum* Ruiz & Pav., 1799. Type: México. Distrito Federal: Mun. Tlalpan, cerca de Xitle, *N. Herrera C. 129* (neotype, designated by Knapp, 2008b, pg. 15: MEXU [MEXU-357763]).
Solanum irazuense Standl. & L.O. Williams, Ceiba 1: 247. 1951. Type: Costa Rica. Cartago: Chicua, Volcán Irazú, 2750 m, 12 Aug 1950, *J. León 2682* (holotype: US [US-2215911]; isotype: CR).

##### Type.

France: Cultivated in Montpellier, of unstated origin (no specimens cited; lectotype, designated here: Dunal, Hist. Nat. Solanum tab. 6. 1813).

##### Description.

Shrubs or small trees, 1–5 m tall. Stems erect, glabrous to sparsely pubescent with simple uniseriate trichomes ca. 0.5 mm long, glabrescent; new growth glabrous or sparsely pubescent with simple trichomes, usually drying dark. Bark of older stems brown or pale brown to yellowish brown. Sympodial units plurifoliate. Leaves simple, (2-)3–20 cm long, (1-)1.2–7 cm wide, elliptic to narrowly elliptic, membranous, the upper surfaces glabrous or with a very few simple uniseriate trichomes on the veins and occasionally extending to the lamina, the lower surfaces sparsely to densely pubescent all along the midrib and on the veins with simple uniseriate trichomes ca. 0.5 mm long, these usually tangled and with small cells, the trichomes occasionally extending to the lamina; primary veins 12–16(-24) pairs, usually drying yellow; base attenuate, not winged onto the stem; margins entire; apex acute; petioles 0.5–2 cm long, with the leaf base narrowly attenuate to the base, glabrous or with a few simple uniseriate trichomes, never twining. Inflorescences terminal or occasionally lateral, 4–15 cm long, many times branched, with 50–100+ flowers, glabrous or with a few scattered simple trichomes, these denser near the pedicel insertion points; peduncle 1.5–7 cm long; pedicels 0.5–0.6 cm long, ca. 0.5 mm in diameter at the base, ca. 1 mm in diameter at the apex, slender, strongly nodding at anthesis, glabrous, articulated at the base from a small sleeve; pedicel scars clustered at the tips of inflorescence branches in groups of 5–10 as small platforms. Buds ellipsoid, the corolla strongly exserted from the calyx tube before anthesis. Flowers all perfect, 5-merous. Calyx tube 1–1.3 mm long, conical, the lobes ca. 0.5 mm long, deltate, glabrous with the tips and margins densely pubescent with simple uniseriate trichomes ca. 0.2 mm long. Corolla 1–1.4 cm in diameter, white, occasionally tinged violet, stellate, lobed 1/2 to 3/4 of the way to the base, the lobes 4–6 mm long, 3–4 mm wide, spreading or planar at anthesis, densely pubescent on the tips and margins with simple uniseriate trichomes, otherwise glabrous. Filament tube minute, the free portion of the filaments 1–1.5 mm long, glabrous; anthers 1.5–2 mm long, 0.5–0.75 mm wide, ellipsoid, loosely connivent, poricidal at the tips, the pores usually lengthening to slits with age. Ovary glabrous; style 5–5.5 mm long, glabrous; stigma minutely capitate, the surface minutely papillose. Fruit a globose berry, 0.7–0.8 cm in diameter, red when mature, the pericarp thin and shiny, glabrous; fruiting pedicels 1–1.2 cm long, slightly woody, erect. Seeds ca. 10 per berry, 3–3.5 mm long, 2–2.5 mm wide, flattened reniform, reddish brown, the surfaces minutely pitted, the testal cells rectangular. Chromosome number: not known.

**Figure 74.**
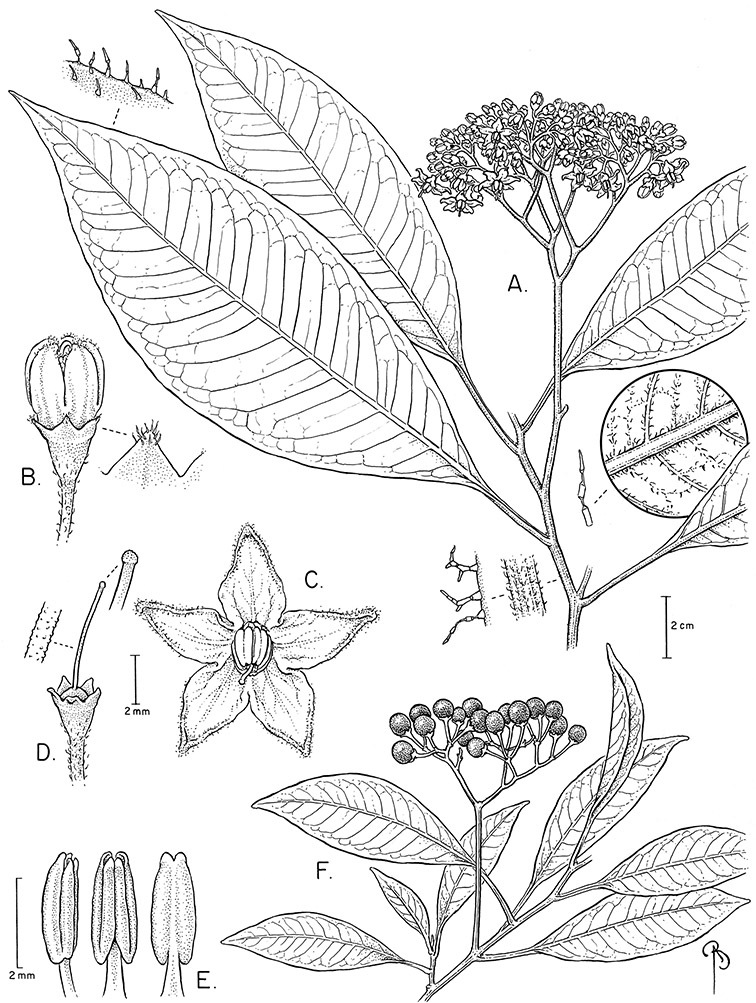
*Solanum pubigerum* Dunal. (**A–E** drawn from *Reveal et al. 4230* **F–G** drawn from *de Avila 178*). Illustration by Bobbi Angell.

##### Distribution 

**Figure 75.**
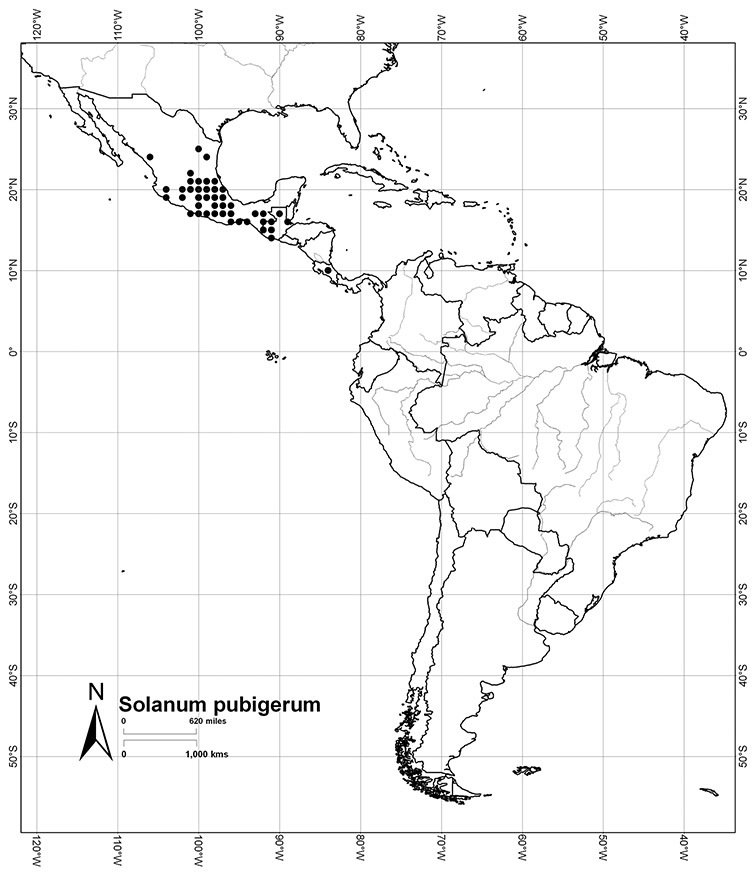
Distribution of Solanum*pubigerum* Dunal.

(Figure 75). From the State of San Luis Potosí, Mexico to Guatemala, with disjunct populations in central Costa Rica, occurring from 2000-3200 m. *Solanum pubigerum* is very common in central Mexico and in the mountains around Mexico City.

##### Ecology.

Common in montane pine-oak forests, secondary forests and forest margins.

##### Common names:

Mexico. Morelos: hierba del negro (*Vázquez S. 1007*)

##### Conservation status.

Least Concern (LC); EOO >100,000 km^2^ (LC) and AOO >10,000 km^2^ (LC). See Moat (2007) for explanation of measurements.

##### Discussion.

*Solanum pubigerum* is extremely similar to *Solanum aligerum*, with which it broadly overlaps in central Mexico and Central America. The two species can be very difficult to distinguish, but *Solanum pubigerum* has simple trichomes all along the midrib, rather than dendritic trichomes concentrated in the veins axils or over the entire lamina. Flowers are smaller in *Solanum pubigerum* with the calyx lobes deltate rather than quadrate (this can be difficult to see), and the berries of *Solanum pubigerum* are also smaller and usually red when ripe (although label data conflict on this point, so some variation may exist). The leaves of *Solanum pubigerum* are in general broader than those of *Solanum aligerum*, but not consistently so. Although leaf size in both these species is quite variable that of *Solanum pubigerum* is more variable than *Solanum aligerum*; specimens of *Solanum pubigerum* have been collected with very small or very large leaves, probably due to habitat conditions. The stems of *Solanum pubigerum* are never winged from the decurrent leaf bases, but those of *Solanum aligerum* are often prominently winged, with the wings persisting in quite old stems. In general the two species appear to not occupy the same forest types where their ranges overlap.

**Figure 76.**
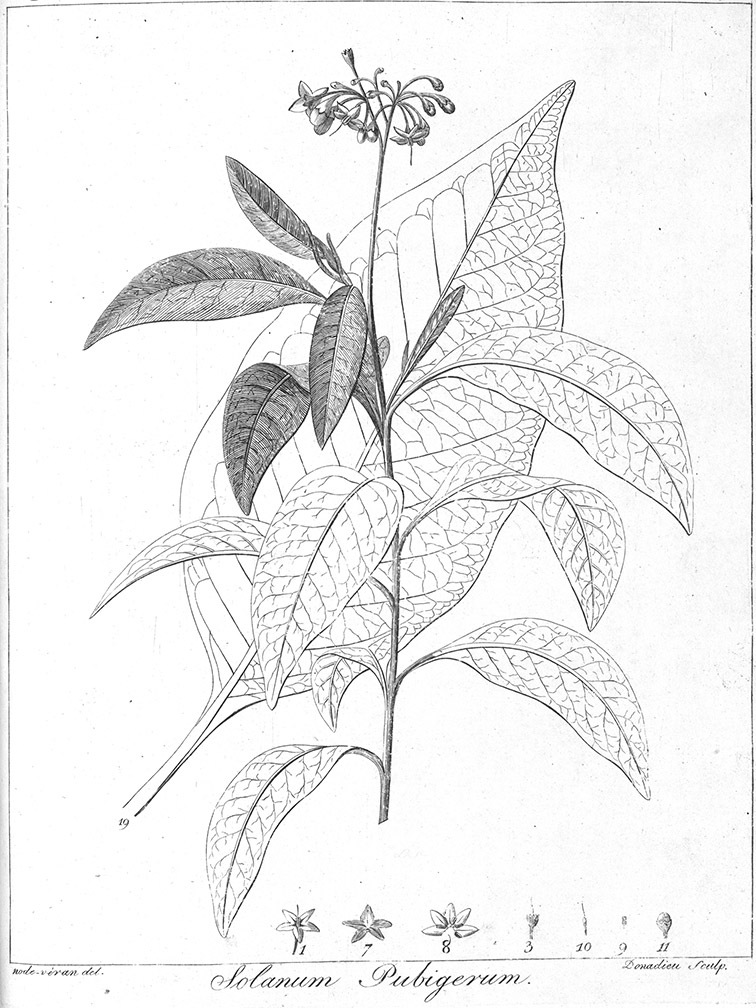
Lectotype of *Solanum pubigerum* Dunal (Dunal 1813: tab. 6). Reproduced with permission of the Natural History Museum Botany Library.

No specimens were cited in the protologue of *Solanum pubigerum*, but a sheet possibly collected by Dunal now housed at MPU [Morton neg. 22273] is a potential epitype; it was apparently grown at “Jardin”, and annotated by Dunal in 1851 as “*Solanum cervantesii* Lag. *pubigerum* Dun.”; other sheets of cultivated plants from the early 19^th^ century held at P and G are also possible original material. I have chosen not to neotypify this name using this material, but instead to use the illustration in Dunal (1813) as the lectotype (see Figure 76), as it is undoubtably original material.

**Figure 77.**
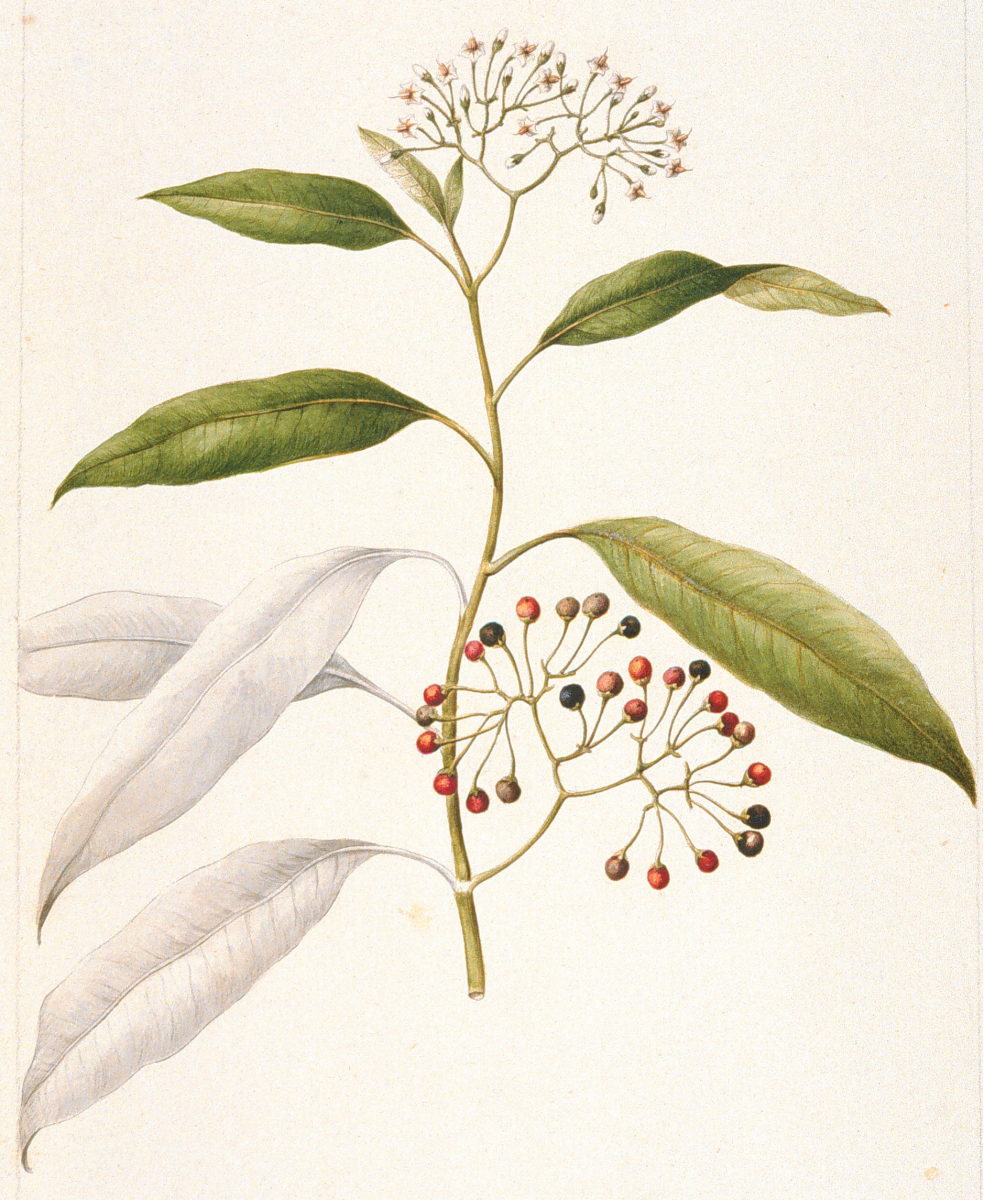
Lectotype of *Solanum leptanthum* Dunal (Torner Collection plate 6331.0673). Reproduced with permission of the Hunt Institute for Botanical Documentation, Carnegie Mellon University, Pittsburgh, PA. Torner Collection of Sessé and Mociño Biological Illustrations. Rights reserved.

I thought for a long time that *Solanum leptanthum* was a synonym of *Solanum corymbosum*, a species in section *Parasolanum* of the Morelloid clade, however a specimen in G cited by Dunal in the *Prodromus* is clearly *Solanum pubigerum*, but has very small flowers and a reduced inflorescence. In coining the epithet *leptanthum* Dunal cited a Sessé and Mocino illustration; as in the case of *Solanum dulcamaroides* (see discussion under that species), he is likely to have seen this in the original set brought by Mocino to Montpellier, thus the plate currently held in the Hunt Botanical Institute (6331.0673, Figure 77) is the only original material associated with this name. Another illustration in that collection (6331.0841, see http://128.2.21.109/fmi/xsl/ArtCat/browserecord.xsl?-lay=Browse&-recid=83319&-find=-find ) is similar, but has black fruits and larger flowers. I suggest that this represents *Solanum aligerum*. Neither of these plates is annotated in Dunal’s hand unlike others in the collection.

*Solanum cervantesii*, the name by which this species was long known, was a rname coined by Lagasca to replace the herbarium name of Cervantes ‘*Solanum microcarpon*’. I have selected a specimen from the Madrid Botanical Garden collected in 1803 annotated as *Solanum cervantesii* in Lagasca’s hand; a possible isolectotype sheet is held at G and is annotated “*Sol. microcarpon Cerv. ex. Lag*.”. The complex neotypification of the Sessé and Mociño epithet “lineatum” (used twice by them) is discussed in detail in Knapp (2008b).

*Solanum glabrum* had been (like *Solanum leptanthum*) considered a member of the Morelloid clade. Dunal (1852) cited his own unpublished illustration (“Dun. ic. ined. t.101*”) intended for publication with his expanded *Synopsis* (Dunal 1816), and a specimen said to be in the herbarium of Humboldt and Bonpland from “Moran, Regla et Omitlan Mexicanorum”. No such specimen is present in P-Bonpl., but a sheet in the general herbarium (P00136343) from Bonpland has a label with the locality “Moran” and is labelled as in the protologue. I have chosen this sheet as the lectotype of *Solanum glabrum*, which represents an unusual almost completely glabrous form of *Solanum pubigerum*.

##### Specimens examined.

**Costa Rica**. **Cartago**: Volcán Irazu, SW side, 2500 m, 8 Jun 1983, *Barringer 3005* (F, PMA); Slope SW of Volcán Irazú, 2500 m, 21 Jan 1983, *Garwood et al. 351* (BM, MO); between Sanatorio and Finca Robert, slopes of Irazu, 2591 m, 4 Oct 1953, *Heiser 3601* (US); Villa Mills area near Cerro de la Muerte, 3000 m, 12 May 1982, *Huft et al. 2144* (MO); Tierra Blanca, 30 Apr 1934, *Orozco 304* (F); Cantón de Cartago, Cuenca del Reventazón, entre Coto Brus y Tierra Blanca, 2060 m, 19 Feb 1997, *Rodríguez et al. 2013* (MO).

**Guatemala**. **Chimaltenango**: Tecpán, Iximche Creek, road to Iximche Ruins, 2500 m, 12 Jan 1966, *Molina R. et al. 16088* (F, NY, US); Los Idolos bridge, 10 kms. from Godines, 2000 m, 21 Sep 1971, *Molina R. & Molina 26702* (F); Tecpan, 2000 m, *Morales Ruano 1266* (F); Chichavac, 2400 m, 29 Jul 1933, *Skutch 507* (US); Above Las Calderas, 1800 m, 15 Dec 1938, *Standley 60004* (F, US); **Huehuetenango**: San Mateo Ixtatán, near the place called Kurus Lemun, 4 miles east of San Mateo Ixtatán along road to Barillas, 2591 m, 7 Aug 1965, *Breedlove 11612* (F, US); San Juan Atitan, 2560 m, 9 Sep 1934, *Skutch 1167* (F, US); Sierra de los Cuchumatanes, above Chiantla, 1950 m, 19 Feb 1939, *Standley 66613* (F); near Soloma, Sierra de los Cuchumatanes, 2400 m, 4 Aug 1942, *Steyermark 49974* (F, NY); Aldea Jolomhuitz, San Juan Ixcoy, 2350 m, 7 Mar 1995, *Véliz 95-4467* (MEXU); Sierra de los Cuchumatanes just below Calaveras, 3000 m, 29 Nov 1962, *Williams et al. 21999* (F, NY, US); **Petén**: Tikal, 3 Nov 1965, *Andrews 501* (NY); **Quetzaltenango**: northside Santa Maria Volcano, 3109 m, 19 Dec 1963, *Eggler 411* (F); 2286 m, 31 Jan 1917, *Holway 815* (US); Cerro Quemado, 8 Feb 1906, *Kellerman 3928* (US); Palmar, 11 Feb 1906, *Kellerman 5801* (MEXU, US); highway km 172 junction Quetzaltenango, Huehuetenango and Totonicapán, 2860 m, 10 Jan 1974, *Molina R. et al. 30184* (F, MO, NY); near Quetzaltenango, 2439 m, 24 Jul 1934, *Skutch 815* (F, US); slopes of Volcán de Zunil, at and above Aguas Amargas, 2430 m, 17 Feb 1939, *Standley 65295* (F); Fuentes Georginas, western slope of Volcán de Zunil, 2850 m, 4 Mar 1939, *Standley 67342* (F, US); slopes of Volcán de Santa Maria, above Palojunoj, 2400 m, 6 Mar 1939, *Standley 67562* (F, US); Cumbre de Tuilacan, southwest of San Martin Chile Verde, 2400 m, 8 Mar 1939, *Standley 67770* (F); Aguas Amargas, on the western slope of Volcán de Zunil, 2450 m, 14 Jan 1941, *Standley 83317* (F, US); region of Boxantin, southeast of San Martín Chile Verde, 2400 m, 16 Jan 1941, *Standley 83821* (F); near Rio Samalá, along road between Zunil and Cantel, 2150 m, 18 Jan 1941, *Standley 83939* (F); Volcán Santa Maria, between Santa Maria de Jesus, Los Mojadas, and summit of volcano, 1500 m, 12 Jan 1940, *Steyermark 33980* (F); Sierra Madre Mountains, about 5 km north of Ostuncalco, 2600 m, 8 Dec 1963, *Williams et al. 25497* (F, NY, US); **Quiché**: Pascual Abaj, west of Chichicastenango, 2500 m, 12 Jan 1966, *Molina R. et al. 16275* (F, NY); **Sacatepéquez**: San Martín, 10 Sep 1938, *Johnston 1352* (F); **San Marcos**: Along road between San Marcos and Quezaltenango, ca 5 miles E of San Marcos, ca. 500m from highway, 2300 m, 14 Jul 1977, *Croat 41032* (BM, MO); Aldea Toniná, volcán Tacaná; sobre camino Talquián (Mexico)-Toniná-cima del volcán, 2700 m, 7 May 1987, *Martínez S. & Ramírez 20814* (MEXU, MO); Camino de Chuco, 2400 m, Jun 1923, *Salas 377* (US); La Cienaga, 2700 m, *Standley 66059* (F); above Rio Tacaná, near San Antonio, 2700 m, 22 Feb 1939, *Standley 66103* (F); Puente de Nahuatl-aa, near San Marcos, 2280 m, 22 Feb 1939, *Standley 66262* (F); El Boquerón, in the mountains at the summit of the road between San Antonio Sacatepéquez and Palestina, 2850 m, 30 Jan 1941, *Standley 85282* (F); between La Vega ridge along Rio Vega and north-east slopes of Volcán Tacaná, to 3 miles from Guatemala-Mexico boundary, in vicinity of San Rafael, 2500 m, 20 Feb 1940, *Steyermark 36196* (F); Palestina de Los Altos, 2400 m, 29 Sep 1992, *Véliz 92.2331* (MEXU); road to Tajumulco Volcano, Sierra Madre Mountains, near San Andres, 2900 m, 2 Jan 1965, *Williams et al. 27007* (F, US); **Sololá**: along Hwy. CA 1, 20 km (by road) SE of Nahualá, just above Colonia María Tucún, 2300 m, 17 Sep 1997, *Nee et al. 47364* (MO, NY); **Totonicapán**: Maria Tecún, 3000 m, 12 Jan 1966, *Molina R. et al. 16362* (F, NY); near Momostenango, 2100 m, 21 Nov 1967, *Molina R. 21420* (F, NY); between kms. 150-158 vicinity of La Cumbre of Totonicapán, 2500 m, 10 Dec 1969, *Molina R. & Molina 25055* (F, MO, US); canyon in the Sierra Madre Mountains, about 5-10 km north of San Carlos, 2800 m, 8 Dec 1962, *Williams et al. 22579* F, (NY, US); Sierra Madre mountains about 8-10 km airline south of Totonicapán, 3100 m, 13 Dec 1962, *Williams et al. 22929* (F); **Totonicapán/Sololá**: Hwy CA1 between Huehuetenango and Chimaltenango, between junction in road to Quezaltenango and Nahualá ca 10 miles SSE of junction to Quetzaltenango, 2950 m, 23 Jan 1987, *Croat & Hannon 63505* (BM, MEXU, MO, NY).

**Mexico**. **Chiapas**: San Cristóbal de las Casas, at north end of San Cristóbal las Casas, 2134 m, 6 Jul 1964, *Breedlove 6053* (F, US); Zinacantán, Zinacantan Center, 2073 m, 30 Mar 1965, *Breedlove 9471* (US); Motozintla de Mendoza, southwest side of Cerro Mozotal, 11 km northwest of the junction of the road to Montozintla along the road to El Porvenir and Siltepec, 2100 m, 27 Jun 1972, *Breedlove 25721* (MEXU, MO); along road between Motozintla de Mendoza and Siltepec; 1.5-5.5 miles N of El Porvenir, 1325 m, 11 Feb 1979, *Croat 47350* (NY); San Cristóbal de las Casas, ravine near Sumidero in the Valley of San Cristóbal las Casas, 2164 m, 17 Feb 1966, *Laughlin 88* (F, US); Mt. Pasitar (Mt. Paxtal), 3 Aug 1937, *Matuda 1707* (F, K, MEXU, MO, NY, US); Vol. Tacaná, Chiquihuite, 27 Mar 1939, *Matuda 2837* (MEXU); Escuintla, Mt Ovando, 14 Nov 1945, *Matuda 16239* (MEXU, US); San Cristóbal de las Casas, Cerro San Felipe, al S de San Cristobal, 11 Sep 1984, *Méndez G. 7920* (MEXU, MO); San Cristóbal de las Casas, Arroyo Las Piedrecitas, en el lado Oriente de San Cristóbal de las Casas, 15 Nov 1985, *Méndez G. 8593* (NY); San Cristóbal de las Casas, Santa Cruz en San Felipe, 15 Nov 1986, *Méndez Ton & López 9780* (MO, NY); Larrainzar, Cruz Quemada, 13 km (by air) NW of San Cristobal Las Casas, 2300 m, 22 Dec 1985, *Nee 32319* (MO); Cerro del Boqueron, Jun 1914, *Purpus 7326* (F, MO, NY, US); Yok Milbil Tulan, 2378 m, 30 Aug 1987, *Santíz Ruiz 86* (MEXU); San Juán Chamula, Paraje Yi’tik, 21 km de Chamula al oeste cerca de San Andres, 2073 m, 14 Jul 1988, *Santíz Ruiz 938* (MEXU); Motozintla, road from Motozintla to Niquivil, 6 km east of Col. Rivera Morelos, 2300 m, 7 Feb 1990, *Stafford et al. 248* (BM, MEXU, MO); Mazapa de Madero, Granaos Talcanaque, 10 km al N, 2500 m, 22 Feb 1987, *Ventura & López 4368* (MEXU); **Colima**: Cuauhtémoc, 400 mts. antes de llegar al Trapiche, 640 m, 25 Feb 1992, *García Torres 30* (MEXU); Cuchilla, northeast of Volcano Colima, 3048 m, 22 Jul 1995, *Goldsmith 64* (US); **Distrito Federal**: San Ángel, Vallee de Mexico, 11 May 1865, *Bourgeau 716* (LE); Tlalpan, N de Volcán Ajusco. ca km 19.4 de Mexico 144, 3200 m, 20 Feb 1983, *Bye 11953* (MO); nine miles west of Parque Nacional Zoquiapan, 2475 m, 28 Jul 1967, *Clarke 612* (NY); Cerro Ajusco, 15 Sep 1978, *D’Arcy 11917* (BM, NY); Tlalpam, 2280 m, 1 Aug 1924, *Fisher* s.n. (MO); 1 km al S del crater del Volcán Xitle, 2400 m, 20 Feb 1987, *García M. & Martínez 2897* (MEXU); Delegación La Magdalena Contreras, cañada que está a 7 km al SO de Contreras, sobre la carretera que va hacia los dinamos, 2950 m, 10 Apr 1978, *García P. 628* (F, MO); Desierto de Los Leones, Dec 1936, *Lyonnet 1369* (MEXU x2, US); Contreras, Sep 1937, *Lyonnet 1626* (MEXU, US); Contreras, Dec 1936, *Lyonnet 3051* (MEXU, US); Cuajimalpa, 1.5 km al NE del Poblado Zacamulpa, 2 Aug 1985, *López 14* (MEXU); Cerro de Sta. Catalina, 2800 m, 19 Mar 1951, *Matuda 21033* (MEXU); Contreras, 4 Feb 1940, *Miranda 97* (MEXU); GA Madero, Sierra de Guadalupe, Cerro Grande, 5 km al NNW de Cuautepec, 30 Jun 1973, *Moreno G. 264* (MEXU); ladera oeste del Volcán Xitle’, 3000 m, 4 Dec 1980, *Panti Madero 476* (MEXU); Tres Cumbres, on road southward from Mexico City to Taxco, 5 Apr 1939, *Perkins & Hall 3376* (BH); near Contreras, 2378 m, 21 May 1901, *Pringle 9489* (BH, K, US); El Desierto, 2 or 3 km beyond La Venta, 6 Oct 1930, *Reddick 4* (BH); S of Contreras, 2700 m, 17 Sep 1930, *Russell & Souviron 192* (US); Xochimilco, San Francisco, 2650 m, 7 Feb 1976, *Rzedowski 961* (MO); Contreras, Aug 1913, *Salazar* s.n. (US); Ladera NO del Cerro Meyuca, Del. M. Contreras, 3000 m, 8 Feb 1992, *Sandoval 61* (MEXU); Contreras, Segundo Dinamo, 2650 m, 24 Aug 1969, *Sereno A. 23* (MEXU); Rio de la Magdalena, between Contreras and the 2nd dynamo, 2652 m, 23 Jul 1944, *Sharp 4412* (MEXU, US); Tlalpan, en San Miguel Ajusco, 1 Sep 1986, *Soto N. & Soto R. 12988* (MEXU); Desierto de Los Leones National Park, 9.3 km from Mex. 15 on road through park (5 km straight SE of La Vemta), 3000 m, 21 Jul 1975, *Steingraeber & Steingraeber 64* (MEXU); Xochimilco, Esquihuil, San Francisco, 2800 m, 5 Feb 1977, *Ventura A. 2565* (MEXU); **Durango**: Tayoltita, Carboneras 54 km al SW de San Miguel de Cruces, Brecha a Toyaltita, 1800 m, 6 Jul 1984, *Tenorio L. et al. 6251* (MEXU); **Guanajuato**: San Luis de la Paz, 2 km de Mesas de Jesus, por el camino al Vergel, 2300 m, 20 Jul 1992, *Díaz B. & García L. 7046* (MEXU); 13 mi N of Cuernavaca along old road to Mexico, 2865 m, 3 Dec 1961, *Gentry et al. 19588* (US); San Luis de la Paz, about 12 km al MW de Mesas de Jesus, camino a San Anton, 2200 m, 24 Apr 1997, *Pérez & Carranza 3609* (MEXU); Ocampo, cerca de La Quebrada, 2000 m, 27 Aug 1994, *Rzedowski 52348* (MEXU); 15 miles north of Guanajuato on old road toward Dolores Hidalgo, 2439 m, 30 Jul 1958, *Straw & Forman 1459* (MEXU); Atarjea, Aldama, 2100 m, 8 Oct 1977, *Zamudio 2510* (MEXU); Guanajuato, 5 km al NE de Santa Rosa, 2580 m, 5 Sep 1998, *Zamudio Murillo 10795* (MEXU); **Guerrero**: Metlatónoc, Xatu Yahta, al W de Coicoyan, terreno de Atzompa, 2700 m, 16 Dec 1987, *Ávila 146* (MEXU); along highway between Millpillas (on highway 95) and Atoyac de Alvavez, 3.7 miles west of turn-off onto road to Chichihualco, 2325 m, 14 Jan 1979, *Croat 45617* (MEXU, MO, NY); Pedro Ascencio Alquisiras, Huixotitla, entre San Juan Tenerias y Puerto Obscuro, 2500 m, 27 Feb 1998, *Cruz Durán 2012* (MEXU); Chichihualco, a 6.5 km W de Puerto del Gallo por camino a Paraiso, 2200 m, 22 Jan 1985, *Hernández & Tenorio L. 853* (MEXU, MO); Chichihualco, Camino Filo de Caballo-Atoyac, 3 km al SO de Filo de Caballo, 2360 m, 17 Oct 1982, *Koch & Fryxell 8290* (BH, BM, F, NY, US); Omiltemi, 30 km al SW de Chilpancingo, 2200 m, 31 Mar 1962, *Lachica & Díaz 4* (US); Chichihualco, 5 km al SE de El Carrizal de los Bravos, camino Filo de Caballo-Chichihualgo, 2480 m, 21 Feb 1983, *Martínez S. et al. 3287* (MEXU, NY); Chichihualco, a 13 km al NW del Puerto Filo de Caballo, 20 Apr 1983, *Martínez S. & Neill 3834* (MEXU); along the road to Filo de Caballo, 43 miles from Mex Hwy 95 near Chilpancingo, 2250 m, 18 Jan 1983, *Miller et al. 487* (BM, MO, NY); Sierra Madre del Sur, along the Milpillas-Atoyac road via Puerto del Gallo, about 40.5 miles SW of Mexico Highway 95, 9 miles SW of Carrazal del Bravo, S of road, 3000 m, 16 Oct 1975, *Reveal et al. 4230* (BM, K); Chilpancingo, camino Ocoxima, El Fresno, 2410 m, 18 Dec 1973, *Sarukhán et al. 3615* (MEXU); Zitácuaro, a 22 km al NE de Zitacuaro, sobre la carretera a Toluca, 2250 m, 18 Jun 1983, *Soto N. 5250* (F, MEXU); Atoyac de Álvarez, 8 km al SO de el Puerto de el Gallo, 2120 m, 24 May 1986, *Soto N. & Solorzano G.12795* (MEXU x2, NY); Ixcateopan de Cuauhtémoc, San Fernardo, 6 km al E de Cruz Alta, 2500 m, 25 Jan 1998, *Soto 7134* (MEXU); Taxco de Alarcón, El Tejocote, 7 km al E de Cruz Alta, 2500 m, 24 Jan 1998, *Soto 7164* (MEXU); General Heliodoro Castillo, 0.5 km NW of La Guitarra, on rd to Toro Muerte 9 km NW of jct. rd from El Paraiso to Pto del Gallo; 48 km NNW of El Paraiso, 2800 m, 9 Jun 1985, *Thomas & Contreras 3757* (NY); Malinaltepec, 1900 m, 24 Jun 1991, *Wegenbreth 642* (MEXU); Zapotitlán Tablas, Ojo de Agua, 1700 m, 15 Oct 1991, *Wegenbreth 792* (MEXU); **Hidalgo**: Ajacuba, La Barranca, localidad al N de poblado Emiliano Zapata, ladera S de la sierra de Chicavasco, ejido E. Zapata, 2180 m, 21 Aug 1968, *Díaz V. & Valverde G. 63* (MEXU); San Agustín Tlaxiaca, Poblado La Victoria, aprox. 5 km despues de Puerto Mexico, rumbo a Chapultepec de Pozos, 2400 m, 15 May 1990, *Díaz V. et al. 930* (MEXU); San Salvador, km 135-137 on Laredo highway, between Actopan and Ixmiquilpan, 8 Oct 1943, *Gilly & Camp 8* (F, MEXU, NY); Epazoyucan, 2 km de Epazoyucan, 2650 m, 25 Jun 1974, *Goruz 81* (MEXU); Zempoala, Tepeyahualco, 2400 m, 2 Jul 1979, *Hernández M. 3270* (MEXU); Apan, 6 kms al noroeste de Apan, 2500 m, 10 Aug 1981, *Hernández M. 6307* (MEXU, MO); Tulancingo, 10 km al NE de Tulancingo, 2300 m, 18 May 1982, *Hernández M.7252* (MEXU); San Miguel Regla, 2090 m, 22 Jun 1978, *Lamy et al. 266* (MEXU); km 231 of highway, between Zimapán and Jacala, 14 Aug 1943, *Lundell & Lundell 12385* (NY, US); margenes de Rio Malila, 6 km al sur de Molango, 1600 m, 15 Jul 1992, *López García 223* (MEXU); Zempoala, Xochihuacan, 13 kms al norte de Zempoala, 2300 m, 3 Nov 1980, *Magaña 5259* (MEXU, MO); Zimapán, La Majada, 20-25 km al noreste de Zimapan, 2100 m, 13 Sep 1981, *Magaña & Hernández M. 6530* (MO); Ixmiquilpan, Las Emes, 20 kms al norte de Ixmiquilpan, hacia la Pechuga, 2200 m, 18 Nov 1981, *Magaña 6659* (MO); Tepeapulco, 2 kms an noreste de Tepeapulco, 2290 m, 2 Mar 1982, *Magaña & Tenorio L. 7059* (MEXU, MO); Zacualtipán, Cerro Corona, 2000 m, 1 Feb 1954, *Matuda 30357* (MEXU); El Chico, Presa Jaramillo, 4.5 km al N de Pachuca, 2750 m, 3 May 1975, *Medina C. 307* (MO); Tlaxcoapan, about 3 miles on road from Tula highway to Tezontepec, 2100 m, 12 Oct 1946, *Moore 1505* (BH); Telles, 21 Sep 1910, *Orcutt 4136* (F, MEXU, MO, NY, US); near Tula, 1920 m, 12 Apr 1940, *Pringle 13134* (BH, F, K, MEXU, MO, SI, US); 4 km al W de Tolcayuca, 2350 m, 23 Aug 1970, *Quintero G. 5* (NY, US); Zontecomate, 3 km SW of Zontecomate, 7 Nov 1930, *Reddick 280* (BH); Real del Monte, 5 Nov 1930, *Reddick 626* (BH); Tolcayuca, ladera N del Cerro de la Cruz, 2650 m, 7 Nov 1975, *Rivera 42* (MEXU); Zimapán, 5 km al Noreste de Trancas, hacia Nicolas Flores, 2200 m, 4 Oct 1980, *Rodríguez 5032* (NY, US); Sierra de Pachuca, 20 Jul 1905, *Rose et al. 8879* (F, NY, US); entre Guerrero y Omitlan, camino a Tampico, 13 May 1985, *Sousa et al. 25* (MEXU, NY); 3 miles N of Puerto Ignacio Isidro Diaz, along MEX 85 N of Zimapan, 20 Aug 1971, *Vaughan et al. 1033* (MO); Actopan, San Juan Solís, Nopalera, 2100 m, 15 Oct 1986, *Velasco & Ojeda 15* (MEXU); Tepeapulco, Cerro de Xihuingo, 2700 m, 8 Nov 1975, *Ventura A. 532* (MEXU); Cerro Maziahua, near Rancho San Lucas. 6 km NNE of APAM, W slopes, 2500 to 2750, 2500 m, 25 Jun 1966, *West E-2* (BM, US); **Jalisco**: Nevado de Colima, 3201 m, 20 Nov 1968, *Boutin & Brandt 2317* (MEXU); Venustiano Carranza, 25 km al SO de Cd Guzman, por carr. a El Grullo, luego 7 km. al SO del Floripondio por brecha a Microondas Las Viboras, 2850 m, 3 Oct 1988, *Fuentes O. 820* (NY); Nevado de Colima, Nevado de Zapotlan, a few miles south of Ciudad Guzman (Zapotlan), 3000 m, 2 Jul 1956, *Gregory & Eiten 292* (MEXU, MO, NY x2); NW slopes of Nevado de Colima, above Jazmín, near upper end of water-line 2-3 km above settlement of El Isote, 2600 m, 26 Mar 1949, *McVaugh & Wilbur 10066* (MEXU, NY, US); NE slopes of the Nevado de Colima, below Canoa de Leoncito, steep cut-over mountainsides in fir zone at head of Barranca de la Rosa, 2800 m, 10 Oct 1952, *McVaugh & Sooby 13403* (BM, MEXU, US); Sierra de Manantlán (15-20 miles southest of Autlán), about 2 miles from Aserradero San Miguel Uno, west and south of divide towards Manzanillo, 2250 m, 4 Nov 1952, *McVaugh & Hoover 13923* (MEXU, US); camino de ascenso al Cerro Viejo, por las Trojes, 2350 m, 9 Sep 1987, *Rodríguez C. 1006* (MEXU); Camino de Atenquique al Nevado de Colima, 2900 m, 10 Jan 1965, *Rzedowski 19381* (MEXU); Tuxpán, 20 km al SO del Fresnillo, brecha Parque Nacional El Nevado, 3350 m, 5 Jan 1990, *Villa C. & Chávez L. 505* (NY); Tonila, ladera de Volcán Nevado de Colima, 2750 m, 9 Aug 1986, *Zamudio 4289* (MEXU); **Michoacán**: Hidalgo, Mil Cumbres, al 3 km al S de Mil Cumbres, cuenca del Río Balsas, 2200 m, May 1985, *Aureoles et al. 8521* (BH); El Agua de la Difunta, Cerro el Cacique, 2800 m, 4 Nov 1978, *Contreras 239* (F); Pátzcuaro, parte alta de Cerro del Burro, cerca de Cuanajo, 3150 m, 24 May 1985, *Díaz Barriga 1028* (MEXU); Pátzcuaro, Huecorio, 2100 m, 25 Nov 1985, *Escobedo 686* (F, NY); 12.5 miles E of Zitacuaro and 3.5 miles E of Macho de Agua along Hwy. 15 between Toluca and Morelia, 1 Nov 1977, *Funk & Hill 2239* (US); Zitácuaro, Jun 1938, *Hinton 11901* (K, NY, US); Zinapécuaro, 2 km al E de Jerahuaro, camino a Huajumbaro, 2380 m, 29 Sep 1988, *Jasso 261* (MO); Tancítaro, Cerro Tancitaro, 3048 m, 19 Aug 1940, *Leavenworth 698* (F); falda W del Cerro Altamirano, al E de Contepec, 2500 m, 8 Jun 1990, *Madrigal Sánchez 4344* (MEXU); Ocampo, en el camino entre El Rosario y El Santuario de las Mariposas Monarca, 2800 m, 26 Feb 1983, *Martínez M. 3331* (MEXU); Contepec, parte alta del Cerro Altamirano, 2570 m, 29 Oct 1991, *Pérez C. & García L. 2570* (MEXU); camino al Norte de Sicuicho, 2350 m, 6 May 1981, *Ramos 173* (MEXU); Santa Clara del Cobre, en la parte alta del Cerro Burro; 30 km al N de Tacambaro, (Cuenca del Rio Balsas y Sierra M del Sur), 2850 m, 15 Mar 1985, *Soto N. & Aureoles 7684* (MEXU); en boca de Cañada, 20 km al NE de Zitacuaro, 2650 m, 22 Mar 1982, *Soto N. & Silva R. 3827* (MEXU, MO); Santa Clara del Cobre, en la parte alta de el Cerro Burro, 2850 m, 15 Mar 1985, *Soto N, J.C*., *Aureoles C, S. 7684* (MEXU); Ocampo, 11 km al SE de Ocampo, en el Cerro El Chivati, 2700 m, 16 May 1986, *Soto N. & Solorzano G. 12611* (MEXU); Zitácuaro, Macho de Agua, 7 km al E de Zitacuaro, Carr. Zitacuaro-Toluca, 2700 m, 27 Aug 1982, *Tenorio L. et al. 1557* (MO); 5 km al S de el Rosario ó 6 km al N de Ocampo, 2600 m, 28 Nov 1985, *Torres C. 7723* (F, MEXU, MO); Tlalpujahua, camino Cerro San Miguel el Alto a Calvario, 2920 m, 21 Oct 1987, *Zamudio 5798* (MO); Salvador Escalante, Cerro La Tapada, ejido Felipe Tzinzun, 3025 m, 21 Jan 1988, *Zamudio 6019* (MEXU); **Morelos**: about 8 miles southwest of Tres Cumbres, 26 Jul 1947, *Barkley et al. 7443* (MEXU); Huitzilac, Atzompa, camino al Tepeite, 5 km al NO de Huitzilac, 2560 m, 6 May 1989, *Bonilla 654* (MEXU); Huitzilac, Parque Nacional Lagunas de Zempoala, 3200 m, 23 Jul 1986, *Cardoso & Estrada 1203* (MEXU); Huitzilac, Rancho San Lorenzo, km 53.5 de la carretera federal (95) Mexico-Acapulco, al SW del poblado Tres Marias, 2660 m, 7 Sep 1989, *Díaz V. 1012* (MEXU); Huitzilac, 7 km al E de Huitzilac (4.5 km por la brecha que parte en el km 7 de la carretera Tres Marias-Zampoala), 2600 m, 10 Mar 1989, *Espejo et al. 3515* (MEXU); Valle del Tepeite, Nov 1933, *Lyonnet 1126* (MEXU x2, US); Lagunas Zempoala, 2506 m, 17 Sep 1938, *Lyonnet 2506* (MEXU, US); **México**: Acuautla, Aug 1988, *Altamirano* (MEXU); Texcoco, Santa Catarina del Monte, 2630 m, 1 Oct 1982, *Ascencio V. 20* (MEXU); San José de Allende, just E of the Michoacán border on the road from Zitacuaro to Toluca, 2750 m, 10 Oct 1985, *Bartholomew 2879* (MEXU, NY); Texcoco, Chapingo, 2240 m, 27 Jun 1991, *Bonilla B. & Monsalvo G.. 162* (MEXU); 0-2W of Encinillas, along Hwy 57, ca. 70 miles NW of Mexico City, 2500 m, 26 Aug 1977, *Croat 44128* (BM, MO); Chalco, 2400 m, 5 Mar 1971, *Ern 515* (B); Texcoco, Cerro Tlaloc, 2300 m, 30 Apr 1974, *García M.* s.n. (MEXU); Amecameca, 15 Feb 1987, *Goodding 2181* (MO); Jilotzingo, 3 km al NW de San Luis Ayucan, 2850 m, 29 Oct 1979, *Gómez C. 91* (MEXU); Texcoco, San Pablo Ixyoc, 21 Jun 1981, *Hahn 544* (F, MEXU, MO); Ixtapaluca, Pueblo Nuevo, 8 km de Coatepec, 2300 m, 15 Nov 1979, *Hernández H.* s.n. (MEXU); Temascaltepec, Comunidad, 2480 m, 8 Jun 1932, *Hinton 855* (BM, G, K, MA); Temascaltepec, Ocotepec, 1500 m, 6 Dec 1932, *Hinton 2879* (BM, F, G, K, MO, US); Temascaltepec, Mesón Vinejo, 2830 m, 2 Jun 1933, *Hinton 3998* (BM, F, G, K, US); lava fields ca. 2 km SSW of La Cima, R.R. station on either side of old highway 95, on top of Serjana de Ajuxco. ca. 1km N of the Morelos border, 3050 m, 23 Jan 1963, *Iltis & Iltis 1690* (BM, US); Amecameca, Cañada de Cerro Venacho, ca. 6 km al Este de Amecameca, 3000 m, 3 Feb 1978, *Koch 784* (F, MO); Amecameca, Dec 1905, *Purpus 1735* (BM, F, MO, NY, US); Tenango de Arista, Parque Nacional Nevado de Toluca, cerro contiguo al NW de San Miguel Balderas, 3100 m, 13 Apr 1985, *Sandoval Bassó 8* (MEXU); San José de Allende, ejido Cuesta del Carmen, cerca del limite con estado de Michoacan, 2500 m, 2 Apr 1985, *Soto N. et al. 7934* (MEXU); Chalco, Tlaxchayote, 2800 m, 13 Jan 1976, *Ventura A. 830* (MEXU); Juchitepec, Pedregal de Pulpito, 2800 m, 20 Apr 1977, *Ventura A. 1321* (MEXU); Texcoco, San Dieguito, 2400 m, 8 May 1984, *Ventura V. 2066* (BH, MEXU); Ocoyoacac, 3 km al NE de La Marquesa, 3200 m, 19 Oct 1980, *Zúñiga G. 128* (MEXU); **Oaxaca**: Paraje “Peña Prieta” (Corral de Piedra), 15 km al norte de la Ciudad de Oaxaca y a 3 km al noroeste del poblado “El Estudiante”, Santa Caterina Ixtepeji, 2700 m, 23 Aug 1997, *Acevedo 49* (MEXU); 19 km NE of Hwy 190 on road to Guelatao (Hwy 175), just below La Cumbre, 2480 m, 12 Oct 1983, *Anderson 13070* (MO, NY); Teotitlán, 21.2 km W of Teotitlán del Camino, 1940 m, 19 Oct 1985, *Bartholomew et al. 3162* (NY); Santiago Juxtlahuaca, El Manzanal, senda para la parcela de Sr. Hemeterio, entrada por Santa Rosa-San Miguel Cuevas, 2060 m, 13 Sep 1996, *Calzada 21361* (MEXU); road from Oaxaca to Papaloapan, 9 miles from hwy 190 (hwy 175) to top, 2332 m, 27 Feb 1960, *Carlson 3693* (F, US); SE slopes of Cerro San Felipe along Mex. Hwy. 175 to Ixtlán de Juárez, 1.8 km below (S of) road summit at La Cumbre, 24 km by road (at jct. with Hwy. 190) and 14 km by air NE of Oaxaca, 2650 m, 10 Jul 1978, *Cochrane et al. 8524* (F, MO); road between Natividad and Talea, 17 km from Natividad, 30 Jun 1983, *Costich & Baldwin 1506* (F); Tlalixtac, ca. 3.8 km from la Cumbre on logging road to NW, 2774 m, 1 Aug 1977, *Davis 806* (MEXU, MO); between Mitla and Cerro San Felipe, 14 Feb 1966, *Ernst 2748* (MEXU, US); Ixtlán, 5 km sobre la brecha La cumbre. Corral de piedra, 2950 m, 31 Jul 1985, *García M. et al. 1740* (MO, NY); Zimatlán, Paraje El Campanario, comunidad de San Pedro El Alto, 2540 m, 18 Sep 1998, *Guizar Nolazco, et al. 4230* (MEXU); trail leading to Cerro San Felipe, overlooking Oaxaca, 3000 m, 1 Apr 1960, *Hale & Soderstrom 20738* (US); Ixtlán, Sierra de Juarez; Ruta 175 entro El Punto y la Cumbre, 2000 m, 20 Apr 1982, *Lorence & Cedillo Trigos 4112* (MEXU, MO); Sierra Juarez, Cerro Corral de Piedra, 12-14 km W of La Cumbre at and below TV tower, 2900 m, 24 Jun 1985, *Luteyn & Lebrón-Luteyn 11666* (K, MEXU, MO, NY); San Juan Mixtepec, Dto Etla. 8 km al NE de San Gabriel Etla, Reg Valles Centrales, 2100 m, 14 Jul 1985, *López Gómez 707* (MEXU); Ixtlán, Atepec, Llano de las Flores, 304 m, 5 Nov 1971, *MacDougall H-71* (F, NY); Ixtlán, Sierra Miguel Aloapam, Sierra Norte, Cuatro Pie, 2300 m, 10 Oct 2001, *Manzano Sosa 4* (MEXU); Ixtlán, Municipio de Atepec: along highway 175 near Llano de las Flores, 3000 m, 24 Mar 1983, *Martin 671* (MEXU, MO, NY, US); Tehuantepec, Jul 1936, *Matuda S-94* (US); 18 miles southwest of the city of Oaxaca, 2286 m, 10 Sep 1894, *Nelson 1348* (US); high ridge west of san Miguel Huantla, 2134 m, 11 Nov 1894, *Nelson 1908* (US); km 23 on road to Guelatao, past La Cumbre, 2347 m, Sep, *Oliver et al. 978* (MO); Sierra de San Felipe, 2896 m, 4 Jun 1894, *Pringle* s.n. (MEXU x2, MO, NY, US); Sierra de San Felipe, 2896 m, 4 Jun 1894, *Pringle 4680* (BM, E, G, GOET, K, LE); Sierra de Clavellinas, 2743 m, 16 Oct 1894, *Smith 697* (MO, NY x2, US); km 29 1/2 del camino Zaachila-Ata. Inés del Monte, 2640 m, 10 Nov 1978, *Solano & Vara 436* (MO); Ixtlán, 26 km, al N de Ixtlán, carretera a Valle Nacional, 24 Apr 1983, *Tenorio L. & Lafrankie 3716* (MEXU, NY); San Pedro Nodón, Loma de Enmedio, al SE de San Pedro Nodón. Dto. Cuicatlán, 8 May 1992, *Tenorio L. 18325* (MEXU, NY); Mihuatlán, Mun. San Juan Ozolotepec. 70 km al S de Rancho Conejo (San Pedro Mixtepec) y 9.1 km al N de San Juan Ozolotepec, 2860 m, 5 Mar 2000, *Torres B. et al. 2022* (MEXU); 14.7 km al N de Diaz Ordaz por la desviacion a Cuajimoloyas, 2520 m, 14 May 1983, *Torres C. et al. 2825* (MEXU, MO, NY); Tlacolula, 13.8 km al N de Diaz Ordaz, camino al Cuajimoloyas, 2670 m, 16 Sep 1988, *Torres C. & Martínez R. 12394* (F, MO, MEXU); Pochutla, 15 Aug 1966, *Ulloa & Hernández M. 247* (MEXU x2); Santiago Laxopa, Distr de Ixtlan, 2000 m, 20 Sep 1986, *Vasquez M. & Martin 0080* (MEXU, NY); Sierra Madre de Sur, near top of Cerro Pilon, c. 70 mi. from Oaxaca by road, 2743 m, 20 Jun 1962, *Webster et al. 11513* (MEXU); Metlatónoc, Xa’a Tuhuni, al W de Coicoyoan, terreno de Atzompa, 2700 m, 8 Feb 1988, *Ávila 178* (MO); **Puebla**: Chignahuapan, Cerro del Papasco, 26 Mar 1948, *Aguirre* & *Reko 532* (MEXU); Totimehuacan, vicinity of Puebla, 2205 m, 8 Oct 1907, *Arsène* s.n. (US); Rancho Posada. vicinity of Puebla, 2194 m, 14 Feb 1909, *Arsène* s.n. (US); Totimehuacán, 2205 m, 8 Apr 1907, *Arsène* s.n. (MO); Morelia, Cerro Azul, 2300 m, Oct 1909, *Arsène* s.n. (G); Maulin de Huexatitla, 2155 m, 23 Sep 1906, *Arsène 325* (US); base de Loreto, 2170 m, Jun 1908, *Arsène 1932* (US); vicinity of Puebla, 2200 m, Nov 1908, *Arsène 3534* (BM, MO, NY, US); vicinity of Puebla, derrière les Saléciens, 2170 m, Oct 1908, *Arsène 3538* (K, MO, US); along the hwy between Puebla and Cordoba near Orrizaba near km 112, just N of Puebla-Veracruz border, 2480 m, 24 Feb 1983, *Miller & Tenorio L.. 692* (BM, MO, NY); Moria, 14 Feb 1909, *Nicholas* s.n. (G); Tepoxachil, 19 Jul 1908, *Nicholas* s.n. (G); Huejotzingo, vicinity of Puebla, 2280 m, 4 Nov 1910, *Nicolas 5559* (US); Boca del Monte, Mar 1905, *Purpus 3010* (F, MO, NY, US); on autopista from Mexico-Puebla, 2470 m, 8 Jul 1993, *Seigler et al. 13787* (MEXU); Tetela de Ocampo, Tilapa, 9 km al S de Tetela, 2000 m, 30 Jun 1987, *Tenorio L. et al. 13764* (MEXU, NY); Cholula, Tapetzingo camino por el lado N de Santiago, Xalitzintla, 2580 m, 8 Oct 1987, *Tlapa A. & Ubierna 772* (MEXU); Tetela de Ocampo, Tilapa, 9 km E de Tetela, 2000 m, 30 Jun 1987, *Toriz A. et al. 528* (MEXU); Zacapoaxtla, Tespilco, 1800 m, 23 Apr 1970, *Ventura A. 957* (F, MO); Zaragoza, El Molino, 1250 m, 17 Mar 1986, *Ventura A. 21852* (MEXU); Zacapoaxtla, 1600 m, 28 Apr 1986, *Ventura A. 21919* (MEXU); **Querétaro**: La Muralla, 2460 m, 6 Jul 1986, *Argüelles 2560* (MEXU); Pinal de Amoles, about 1 km al ENE de El Puerto de El Madroño, 2450 m, 19 Dec 1989, *Carranza 2264* (MEXU); Amealco, Cerro de Don Nica, Cañada del Venado, al E de San Pablo, 2800 m, 24 Nov 1992, *Díaz B. & Carranza 7330* (MEXU); Cadereyta, laderas del Cerro Zamorano, 2400 m, 27 Mar 1985, *Fernández N. 2864* (F); San Joaquín, ruinas Las Ranas, 2 km al N de San Joaquin, 2300 m, 25 May 1986, *Fernández N. 3312* (NY); Landa, Puerto de los Cajones, 4 km al NE de La Vesca, 1980 m, 22 Nov 1988, *González 316* (MEXU); Landa, La Cienega, 3 km al SE de La Florida, 1940 m, 6 Mar 1989, *González 424* (MEXU); between San Juan del Río of Hac. Cierva, 19 Aug 1905, *Rose et al. 9628* (BM, NY, US); cerca de Huazmazontla, 13 km al NE de Pinal de Amoles, sobre la carretera a Jalpan, 1300 m, 12 Mar 1989, *Rzedowski 48405* (MEXU); Cadereyta, 9 km al NE de Vizarron, sobre la carretera a San Joaquin, 2350 m, 11 Mar 1978, *Zamudio 2688* (MEXU); **San Luis Potosí**: 26 miles east of San Luis Potosí, along hiway 86 to Rio Verde, 2439 m, 14 Jul 1963, *McGregor et al. 725* (US); Álvarez, 13 Jul 1994, *Palmer 195* (F, K, MO, NY, US); San Luis Potosí, 1829 m, 1878, *Palmer & Parry 638* (F, F, MO, NY); Alvarez, Sierra de Alvarez, 2200 m, 30 Jul 1934, *Pennell 17839* (US); 22 km W of Santa Catarina on highway 86 at km 49, 2200 m, 29 Sep 1965, *Roe & Roe 2191* (MEXU, US); **Tamaulipas**: Hidalgo, 28.1 millas al SO de San Francisco, 1700 m, 28 Dec 1991, *Estrada et al. 2382* (NY); roadside between Victoria and Antiguo, 335 m, Jun 1937, *Happ 61* (MO); Hidalgo, Puerto Purification, 1695 m, 2 Aug 1994, *Hinton 24534* (MEXU); Hidalgo, Paraje de los Caballos, along road from Sta Engracia to Dulces Nombres, 11.7 mi E of Dulces Nombres, 1820 m, 4 Mar 1995, *Nesom et al. 7866* (K, MEXU, NY, NY); **Tlaxcala**: Tlaxco, margenes de Atotonilco, 2400 m, 8 Sep 1992, *Ruiz T. 291* (MEXU); **Veracruz**: Puerto del Aire, Carretera Puerto del Aire, poblado de la laguna, 2320 m, 16 Dec 1977, *Calzada 4181* (F); Orizaba, carretera de puebla a Orizaba, 2 km del bordo con Puebla, en una barranca, 2450 m, 1 Jul 1977, *Fay & Hernández 00752* (F, US); Coatepec, 8 km al SE del Entronque las Juntas-Coatepec sobre la terraceria Agua Bendita, 2665 m, 9 May 1988, *Flores F. & Terpán A. 837* (MEXU); Huayacocotla, Santiago, 2100 m, 22 Feb 1971, *Hernández M. & Cedillo T. 1062* (F, MO); Acultzingo, 1 May 1937, *Matuda 1113* (K, MO, NY, US); along Mexican hwy 150 at the turnoff to Puente Colorado, hills above Ciudad Mendoza, 2000 m, 3 Jun 1987, *Miller & Torres C. 2970* (BM, NY); Ixhuacán, Barranca La Funda, cerca de Los Laureles,al S del Cofre de Perote, 2750 m, 7 Oct 1983, *Narave F. & Vázquez B. 1048* (MEXU, NY); Maltrata, 2175 m, 1 May 1983, *Nee & Taylor 27037* (F, G, MO, NY); Acultzingo, along hwy Mex. 150, 0.5 km from Edo. Puebla border and 0.8 km SSW of Puerto de Aire, 2250 m, 20 Sep 1986, *Nee 33119* (MO, NY); Pastizal arriba de Santiago, 1980 m, 20 Jul 1971, *Nevling & Gómez-Pompa 1813* (F, MEXU); Chiconquiaco, 6 km approx al NE de Chiconquiaco, camino a Vaqueria, 2050 m, 23 Apr 1990, *Pérez G. & López 432* (MEXU); Huayacocotla, Las Blancas, 2230 m, 29 Sep 1994, *Pérez G. 993* (MEXU); Puerto del Aire, on Hwy. 150 just north of Puebla-Veracruz state line, 12 Jun 1973, *Sallee ES-80* (MEXU); Córdoba, 6 Aug 1961, *Schwabe* s.n. (B); Jalapa, 1894, *Smith 1788* (F); Huayacocotla, “Capadero”, on the west facing slopes 1-2 km W of Kaolin mines near La Carbonera (Los Jacales), 2200 m, 25 Jan 1984, *Taylor & Nee 241* (F, NY); Acultzingo, Sierra Nahuatl de Zongolica, 2400 m, Jul 1994, *Weimann CWEI-204* (MEXU); **Zacatecas**: Pinos, 7 km sobre camino de terraceria, La Pendencia-Pinos, 2200 m, 20 Aug 1977, *García M.* s.n. (MO).

#### 31. Solanum pyrifolium

Lam., Tabl. Encycl. 2: 19. 1794

http://species-id.net/wiki/Solanum_pyrifolium

Figure 78

Solanum domingense Dunal, Prodr. [A.P. de Candolle] 13(1): 80. 1852. Type: Dominican Republic. Sin. loc., *Anon.* s.n. (holotype: G-DC [G00144900, F neg. 33943]).

##### Type.

“Martinica”, sin. loc. [?Hispaniola], *J. Martin* s.n. (holotype: P-LA [P00357682]; possible isotypes: G [G00070193, G00070194]).

##### Description.

Herbaceous or woody vine. Stems sparsely pubescent with minute, simple uniseriate trichomes 0.1–0.3 mm, these denser at the nodes; new growth pubescent with simple uniseriate trichomes ca. 0.2 mm long, especially along the veins. Bark of older stems greyish green. Sympodial units plurifoliate. Leaves simple, 3–7 cm long, 1.4–3.5 cm wide, elliptic or ob-elliptic to narrowly elliptic, membranous, glabrous above except for a few minute simple uniseriate trichomes along the midrib, pubescent along the veins beneath with simple uniseriate trichomes to 0.5 mm long; primary veins 5–6 pairs, joined in a prominent submarginal vein; base truncate and oblique or slightly cordate; margins entire; apex acute to acuminate; petioles 1.2–4 cm long, sparsely pubescent with simple uniseriate trichomes, especially adaxially, twining to aid climbing. Inflorescences terminal or occasionally leaf-opposed, 7–17 cm long, branching many times, with 5–100 flowers, very sparsely pubescent with simple trichomes like those of the stems; peduncle 0.5–1.9(-3) cm long, sparsely pubescent; pedicels gradually tapering, 1.2–1.5 cm long, ca. 1 mm in diameter at the base, 2–2.5 mm in diameter at the apex, nodding at anthesis, glabrous or with a few simple uniseriate trichomes, articulating in the basal 1/3 leaving a peg 0.5–0.8 cm long; pedicel scars (pegs) widely spaced 1–1.3 cm apart. Buds globose, becoming ellipsoid, the corolla strongly exserted from the calyx tube. Flowers all perfect, 5-merous. Calyx tube 1.5–2 mm long, a narrow cup grading into the pedicel apex, the lobes 1–1.5 mm long, quadrate with thickened margins, glabrous or sparsely pubescent with simple trichomes abaxially, densely pubescent adaxially. Corolla 2–2.6 cm in diameter, pale violet or blue, stellate, lobed ca. 2/3 of the way to the base, the lobes 0.8–1 cm long, 0.3–0.4 cm wide, planar to slightly campanulate at anthesis, densely papillate on the abaxial surfaces, margins and tips, glabrous adaxially. Filament tube ca. 0.5 mm long, the free portion of the filaments ca. 1.5 mm, glabrous; anthers 3–3.5 mm long, 1–1.2 mm wide, ellipsoid, loosely connivent, poricidal at the tips, the pores lengthening to slits with age. Ovary conical, glabrous; style 0.7–0.8 cm long, glabrous; stigma clavate, the surface minutely papillate. Fruit a globose berry, ca. 1 cm in diameter, dark blue-black when ripe (fide *Howard & Howard 9423*); fruiting pedicels 1–2 cm long, gradually tapering to the apex, ca. 1 mm in diameter at the base, ca. 3 mm in diameter at the apex, glabrous; calyx lobes in fruit quadrate, paler than pedicel. Seeds >20 per berry, flattened reniform, 2–2.5 mm long, 1–2.5 mm wide, pale yellow, the surfaces minutely pitted. Chromosome number: not known.

**Figure 78.**
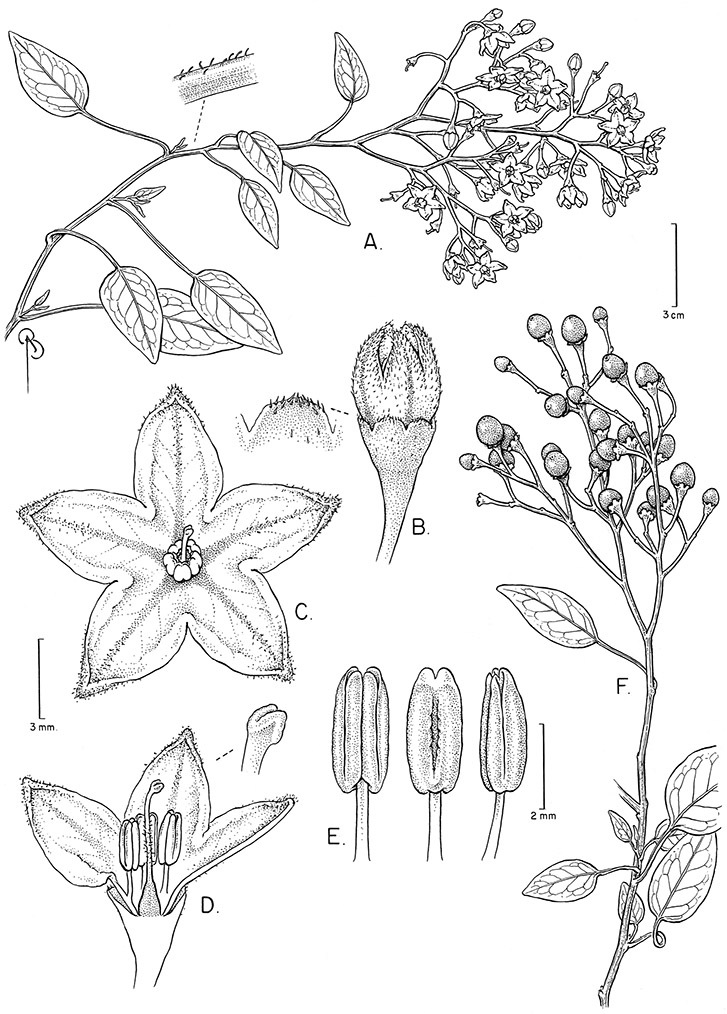
*Solanum pyrifolium* Lam. (**A–E** drawn from *Colella 1361* and M. Nee unvouchered photographs from Hispaniola **F** drawn from *Gastony et al. 525*). Illustration by Bobbi Angell.

##### Distribution

**Figure 79.**
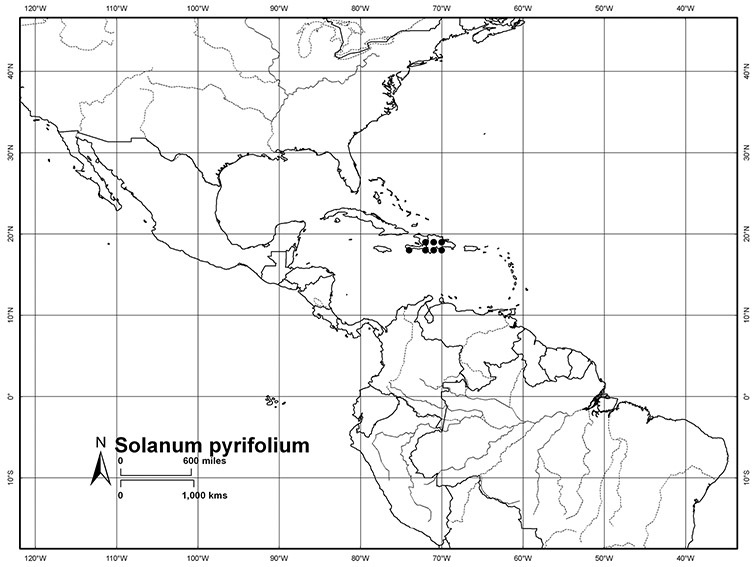
Distribution of Solanum*pyrifolium* Lam.

(Figure 79). Almost endemic to the island of Hispaniola, with a single collection from eastern Cuba; most specimens from the Dominican Republic; 120–1600 m elevation.

##### Ecology.

Along forest edges on limestone and in cloud forests.

Conservation status. Near Threatened (NT); EOO <45,000 km^2^ (NT) and AOO >10,000 km^2^ (LC). See Moat (2007) for explanation of measurements.

##### Discussion.

*Solanum pyrifolium* is similar to the other members of the Dulcamaroid clade growing in the Caribbean, *Solanum boldoense* of Cuba and the widespread and often cultivated *Solanum seaforthianum*. It can be distinguished from those species by its inflorescence that is narrow in outline rather than globose or broadly ellipsoid, its entire leaves that are generally narrower than those of *Solanum boldoense* with a distinct submarginal vein (leaves of *Solanum seaforthianum* are usually pinnatifid) and quadrate calyx lobes with a distinctly thickened margin. Dried specimens of *Solanum pyrifolium* are a distinctive greenish gray color, rather than the more blackened or green specimens of the other two taxa.

The articulation point on the pedicel of *Solanum pyrifolium* is always more basal than that of *Solanum boldoense*, but occasionally a short peg is left on the inflorescence axis at fruit or flower fall.

Although Lamarck’s protologue indicates that the plant collected by Martin came from Martinique (“Martinica”), *Solanum pyrifolium* has never again been collected there. A similar situation exists for *Solanum crotonoides* Lam. (Leptostemonum clade), also collected by Martin from “Martinique”. I suspect that Martin did in fact also visit Hispaniola or that Martin (or those who labelled his collections back in Paris) used Martinique as a broad locality covering much of the Caribbean region (although other collections of his have labels stating “S. Domingo”), and that both these plants are true Hispaniolan endemics. Two sheets of *Solanum pyrifolium* at G collected by Martin are labelled “Domingo” and are probable isotype material.

##### Specimens examined.

**Cuba**. **Oriente**: Bayamesa, Sierra Maestra, Pico de la Bayamesa, on or near the crest, 1493 m, 16 Jul 1955, *Harvard Course in Tropical Botany 671* (GH);

**Dominican Republic**. Arroyo Oro, 10 Oct 1946, *Canela* s.n. (JBSD); *Liogier & Liogier 21981* (JBSD); El Convento, Constanza, 1200 m, 19 Jan 1975, *Liogier & Liogier 22338* (JBSD); Santa Domingo, *Martín* s.n. (G); **Azua**: in Valle Rio Yaque de Sur, 1300 m, Aug 1912, *Fuertes 1901* (A, E); La Sabana, at west edge of Sabana de San Juan, 1600 m, 17 Sep 1980, *Mejía & Zanoni 8187* (JBSD); **Bahoruco**: Sabana de Silencio, Sierra de Neiba, 2012 m, 27 Jun 2005, *Acevedo et al. 13175* (JBSD); Sabana de Silencio, entre los limites de la provincia de San Juan y Bahoruco, Sierra de Neyba, en los alrededores del Valle, 1985 m, 27 Feb 2001, *Veloz et al. 2511* (JBSD); **Barahona**: La Hotte, La Hotte, between La Cueva and Placer Bonita, 1067 m, 1 Aug 1950, *Howard 12292* (A, BM, LE); Morne La Jo, en la cima (nombre actual Firme La Jo como tiene el mapa), Sierra de Baoruco, 1550 m, 6 Jun 1984, *Zanoni & García 30422* (JBSD); **Distrito Nacional**: prope Constanza, 1200 m, Jul 1910, *Türckheim 3262* (BM, E, F, G, GH, K, MA, MO, S); **Independencia**: Sierra de Neiba, along the Carretera Internacional near the crest of the range, near Haitian border, vic. line between Sand Rafael and Independencia, 1700 m, 5 Aug 1967, *Gastony et al. 525* (GH); Jimaní, 44 m, 25 Jul 1985, *Grifo & Matuszak 216* (BH, MO); **La Vega**: El Cajón, extension of Pinar Bonito road just before saw mill of Pinar Parejo, S of Constanza, 1850 m, 16 Oct 1981, *Dod* s.n. (JBSD); La Ciénaga, south bank of Río Los Guanos, just above conflunece with Río La Izquierda, 1100 m, 15 Jul 1967, *Gastony et al. 205* (GH); Constanza, 3 km al sur, en la carretera que va a San José de Ocoa, proximo a la entrada a Pinar Parejo, 1185 m, 12 Feb 1991, *Jiménez & Mione 97* (JBSD); Constanza, Loma Redonda, Cienaga de la Culata, 1700 m, 30 Nov 1969, *Liogier 17152* (GH); Arroyo La Siberia, about 17 km S of Constanza (via El Convento) on road to San José de Ocoa, 1600 m, 24 Jul 1980, *Mejía & Zanoni 7600* (JBSD, MO); Valle Nuevo, c. 4 miles N, 1905 m, 4 Jan 1970, *Terborgh 69* (A); Loma El Campanario, Cordillera Central, 4 km oeste de La Culata de Constanza, 1800 m, 8 Sep 1982, *Zanoni et al. 23227* (JBSD); Loma El Campanario, base norte y subida de la loma (= Pico de Piedra en el mapa), 4 aero kilometros oeste de la Culata de Constanza, un valle entre dos lomas, ladera de El Campanario, 1300 m, 13 Oct 1983, *Zanoni et al. 27520* (JBSD); Pinar Parejo, antes el poblado, 1769 m, 20 Jul 1989, *Zanoni et al. 42952* (JBSD, MO); **Monseñor Nouel**: El Mechecito, Municipio de Bonao, Sección Blanco, cerca del Río Toro Flaco, la cima del bosque nublado, 1400 m, 10 Jun 1998, *Peguero et al. 725* (B, JBSD); El Mechecito, Municipio de Bonao, Sección Blanco, alrededor de los conucos de Ramoncito Canela, 1250 m, 11 Jul 1998, *Peguero et al. 733* (B, JBSD); **Pedernales**: Las Abejas Ravine, Sierra de Bahoruco Mountains, ca. 50 km from Cabo Rojo on the Alcoa Aluminum Road, ca. 10 km W of the end of the paved road (ca. 20 km N of Pedernales), 1100 m, *Fisher-Meerow 804* (JBSD); Pedernales, above Los Arroyos, alogn the International Highway, 1500 m, 8 Nov 1969, *Liogier 16758* (GH); Los Arroyos, 900 m, 25 Jun 1977, *Liogier & Liogier 26973* (JBSD); Los Arroyos, 4 km NNE, 1550 m, 29 Jul 1990, *Thompson et al. 7588* (JBSD); Las Abejas, 50 km from Alcoa Exploartion Company port of Cabo Rojo on road to (Las Mereceds) and Las Abejas, at road end, 1128 m, 15 Sep 1981, *Zanoni & Mejía 16604 A* (JBSD); **Peravia**: Loma Piedra Blanca, Cordillera Central, al oeste de Las Cayas, 3 horas caminando a pies hacia el souroeste de La Horma, San José de Ocoa, nacimiento del arroyo Las Cayas, 1520 m, 27 Jun 1984, *Mejía et al. 931* (JBSD, MO); Loma del Rancho SE de San José de Ocoa, 130 m, 19 Aug 1987, *Pimentel & García 777* (JBSD, MO, S); Cordillera Central, 33.9 km Norte del Parque Central de San José de Ocoa en la carretera a Constanza, sitio arriba del camino en la vecinidad de La Nuez, 1800 m, 7 Jul 1982, *Zanoni et al. 21410* (JBSD, MO); Loma de La Valvacoa, Cordillera Central, lado Norte de la Loma, arriba del poblado rural de El Quinoal, 1300 m, 14 Jul 1982, *Zanoni et al. 21552* (JBSD); El Caliche, Cordillera Central, 15 km N desde el Parque Central y 8-1-km desde el cruce de Los arroyos en el camino a Carmona, zona rural denominado El Caliche o Carrao, 1341 m, 21 Jul 1982, *Zanoni et al. 21868* (JBSD); Cordillera Central, en le camino a La Nuez (de San José de Ocoa) a Tetero de Mejía, 1950 m, 24 Dec 1985, *Zanoni & Cabral 35857* (JBSD); **San José de Ocoa**: Sierra de Ocoa, Prov. de Azua, San José de Ocoa, Bejucal, slope of Loma de los Palos Majados, 1250 m, 5 Mar 1929, *Ekman H-11793* (K, S); **San Juan**: Pinar Grande, Sabana El Silencio, en la loma de Los Magueyes, 1920 m, 3 Dec 2000, *Clase et al. 2403* (JBSD); Loma Mampin, El Carrote or Loma de la Laguna Guardarraya, N of Derrumbadero, of El Vallecito of El Cercado, 1700 m, 28 Sep 1980, *Dod* s.n. (JBSD); Piedra del Aguacate to Rio del Oro, 9 Oct 1946, *Howard & Howard 9423* (A, BM, GH, S); **Santiago**: San José de las Matas, Montes Negros de Río Baito, Cordillera Central, sección Mata Grande, Parque A. Bermúdez, 1430 m, 23 Apr 1999, *Clase et al. 1014* (JBSD, MO).

**Haiti**. Massif de la Selle, 28 Mar 1937, *Bailey 202* (BH); Massif de la Selle, ravine on the Northern slope of Morne Cabaio, bottom of the ravine, 28 Aug 1924, *Ekman H-1697* (A, F, G, GH, MO, S); Massif des Matheux, Grands-Bois, Morne Caya, 1300 m, 15 Mar 1926, *Ekman H 5711* (S); near Mare Boeuf, Mornes des Commissaires, 1600 m, 2 Jan 1942, *Holdridge 935* (BM, F).

#### 32. Solanum ruizii

S.Knapp, Bull. Brit. Mus. Nat. Hist. (Bot.) 19: 91. 1989

http://species-id.net/wiki/Solanum_ruizii

Figure 80

Solanum lanceolatum Ruiz & Pav., Fl. Peruv. 2: 33, tab. 164a. 1799, non *Solanum lanceolatum* Cav. 1795. Type: Peru. Huánuco: Muña, *H. Ruiz & J. Pavón* s.n. (lectotype, designated by Knapp, 2008c, pg. 320: MA [MA-747163]; isolectotypes: G [G00357895], MA [MA-747164, MA-747165]).
Solanum patulum Pers., Syn. 1: 223. 1805, non *Solanum patulum* (L.) Roth, 1800. Type: Based on *Solanum lanceolatum* Ruiz & Pav., non *Solanum lanceolatum* Cav., 1795.
Solanum patulum Pers. var. *pilosistylum* Bitter, Bot. Jahrb. Syst. 54, Beibl. 119: 9. 1916. Type: Peru. Huancavelica: Tayacaja, cerros al lado derecho del Río Mantaro al sur de Surcubamba, 3800 m, 12 Mar 1913, *A. Weberbauer 6477* (holotype: B, destroyed; lectotype, designated by Knapp 1989, pg. 91: MOL; isolectotypes: G [G00070195], MOL [2 sheets]).
Solanum patulum Pers. forma *album* J.F.Macbr., Publ. Field Columbian Mus., Bot. Ser. 8: 111. 1930. Type: Peru. Huánuco: Tambo de Vaca, 3900 m, 10 Jun 1923, *J.F. Macbride 4441* (holotype: F [F-535527]; isotypes: G [G00070196], MA [MA-205962], US [US-1592826], W [W-1936-3811]).

##### Type.

Based on *Solanum lanceolatum* Ruiz & Pav., non *Solanum lanceolatum* Cav. 1795.

##### Description.

Shrubs or small trees, 2–6 m tall. Stems densely pubescent with golden-yellow echinoid trichomes, these often elongate and tree-like; leaf scars somewhat raised; new growth densely pubescent with yellow echinoid and tree-like trichomes both above and below. Bark of older stems pale yellowish-white, glabrate. Sympodial units plurifoliate. Leaves narrowly elliptic, 7–13.5 cm long, 3–4 cm wide, the adaxial surfaces drying black with scattered golden echinoid and tree-like trichomes, these denser along the veins, the abaxial surfaces pubescent with the same echinoid trichomes, not drying as dark as the upper surfaces; primary veins 7–8 pairs, pubescent; base acute, not decurrent on to the petiole; margins entire; apex acuminate; petiole 1–1.5 cm long. Inflorescences terminal, becoming lateral with overtopping of the shoot, very large, 12–15 cm long, narrowly elliptic in outline, branching 8–10 times, with 10–25 flowers, each branch 0.5–3 cm long, densely pubescent with echinoid and tree-like trichomes; peduncle 1–2 cm long; pedicels 1.8–2.2 cm long, tapering from a basal diameter of 0.5–1 mm to an apical diameter of 1–1.5 mm, sparsely to densely pubescent with golden echinoid and tree-like trichomes, deflexed or horizontal at anthesis, articulated at the base and inserted in a sleeve ca. 0.5 mm long; pedicel scars closely spaced and clustered at the inflorescence branch tips. Buds ellipsoid, the corolla strongly exserted from the calyx tube. Flowers all perfect, 5-merous. Calyx tube 4–6 mm long, conical, the lobes 4–5 mm long, narrowly triangular, pubescent abaxially with the same trichomes as those of the pedicels, densely pubescent with golden dendritic trichomes adaxially. Corolla 4–4.8 cm in diameter, rotate-stellate, violet, very large and showy, lobed 1/2 of the way to the base, the lobes 23–25 mm long, 12–15 mm wide, planar or slightly cupulate at anthesis, densely pubescent abaxially with golden dendritic trichomes and with a few dendritic trichomes along the main veins adaxially. Filament tube minute or absent; free portion of the filaments ca. 1 mm long, glabrous; anthers ca. 5 mm long, 1–1.5 mm wide, loosely connivent, poricidal at the tips, the pores becoming slit-like with age. Ovary glabrous or with a few golden dendritic trichomes at the apex; style 1–1.2 cm long, densely pubescent at the base with golden dendritic trichomes; stigma bilobed, the surface minutely papillose. Fruit a globose berry ca. 1.1 cm in diameter (immature?), purplish-black with thin pericarp; fruiting pedicels 1.5–2 cm long, somewhat woody, erect or nodding,. Seeds ca. 30 per berry, c. 3.5 mm long, 3 mm wide, reddish-brown, flattened lenticular, the surfaces minutely pitted. Chromosome number: not known.

**Figure 80.**
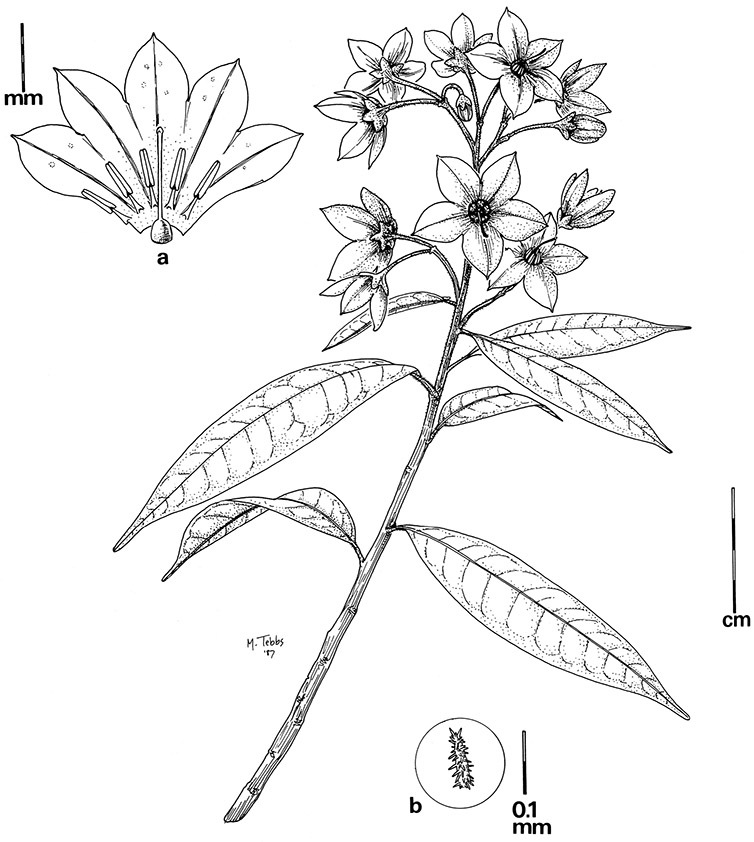
*Solanum ruizii* S. Knapp. (**A–B** drawn from *Macbride 4351*). Reproduced from Knapp (1989) with permission of the Natural History Museum Botany Library. Illustration by Margaret Tebbs.

##### Distribution

**Figure 81.**
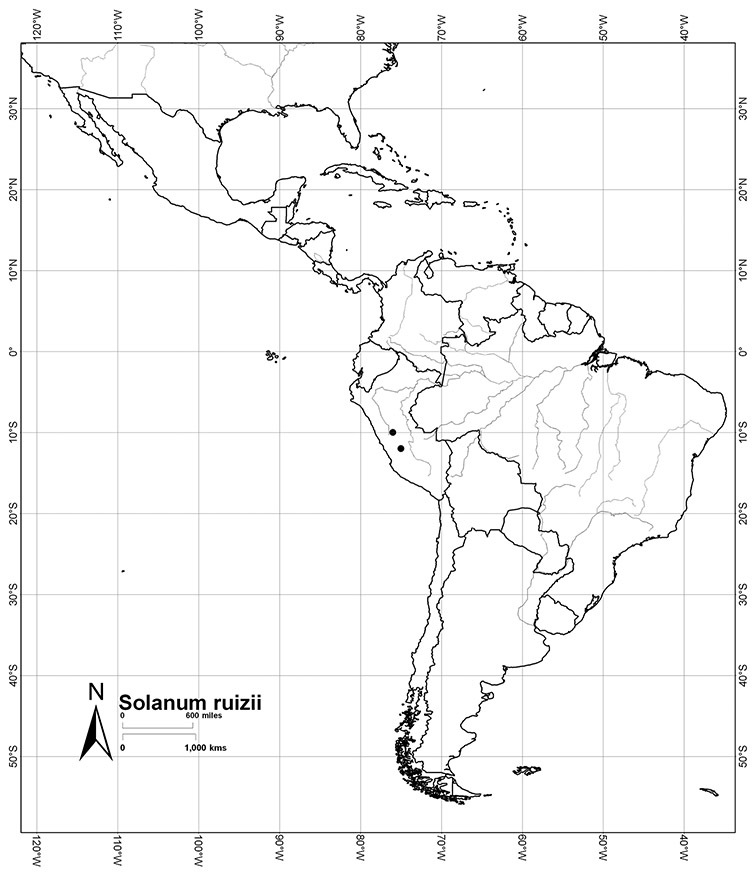
Distribution of *Solanum ruizii* S.Knapp.

(Figure 81). Endemic to central Peru from Depts. Huánuco to Huancavelica; from 3000–4000 m.

##### Ecology.

In moist situations in cloud forests and forest margins; often growing in open grassy areas.

##### Common names.

Peru. uruhuacta (*Macbride 4351*)

Conservation status. Vulnerable (VU); EOO <10,000 km^2^ (VU) and AOO <4,500 km^2^ (NT). See Moat (2007) for explanation of measurements.

##### Discussion.

*Solanum ruizii* is certainly one of the most spectacular members of the *Solanum nitidum* species group with its large, deep purple flowers. All of the specimens of this species characterize it as being very showy. Label data on *Woytkowski 147* (F) state that *Solanum ruizii* is “very common up to 3600 m.a.s level. Sometimes, growing in open places, attains considerable height and forms trees”. *Solanum ruizii* has not been collected since the early part of the 20th century, but large-scale habitat destruction probably accounts for this. Collections of this species come from slightly drier areas than typical ‘ceja de la montaña’ vegetation in which other members of the *Solanum nitidum* group (sensu Knapp 1989) occur. These drier areas are characterized by extensive expanses of high elevation grassland or ‘pajonal’.

*Solanum ruizii* can be distinguished from its closest relative *Solanum cutervanum* by flower size and by the number of seeds in the berries (ca. 30 versus only 7-8 in *Solanum cutervanum*). Mature fruits of *Solanum ruizii* are not known, but the label on *Pearce* s.n. from Muña states they are “large”, suggesting that the fruits I have observed are immature.

Holotypes or lectotypes for Ruiz and Pavón names in the Madrid herbarium (MA) not specifying a particular sheet are not sufficiently precise. Knapp (1989) lectotypified *Solanum lanceolatum* citing only a sheet in MA; this was rectified in 2008 by citation of the particular sheet (Knapp 2008c).

##### Specimens examined.

**Peru**. **Huancavelica**: Tayacaja, Huaribamba, 1 km before Huari, 3170 m, 28 Jul 1968, *Saunders 1173* (F); Tayacaja, Tayacaja, 3700 m, 10 Aug 1949, *Velarde Nuñez 2008* (US); **Huánuco**: Tambo de Vaca, 3963 m, 10 Jun 1923, *Macbride 4351* (G, US); Muña, May 1863, *Pearce 156* (BM); **Junín**: between Acopalca and Huari in steep canyon, NE of Huancayo, 4024 m, 19 Aug 1977, *Duncan et al. 2737* (NY).

#### 33. Solanum salicifolium

Phil., Anal. Univ. Chile 36: 195. 1870

http://species-id.net/wiki/Solanum_salicifolium

Figure 82

Solanum incisum Griseb., Abh. Königl. Ges. Wiss. Göttingen 24: 251. 1879. Type: Argentina. Córdoba: Sierra de Achala, 24-25 Mar 1874, *G. Hieronymus 220* (lectotype, designated by Morton 1976, pg. 99: GOET [GOET003582]; isolectotypes: B [destroyed, F neg. 2779], CORD [CORD00006112]).
Solanum sericeum Ruiz & Pav. var. *strigillosum* Griseb., Abh. Königl. Ges. Wiss. Göttingen 24: 252. 1879. Type: Argentina. Córdoba: Dept. Las Minas, Cerro de Orcosu [Achala in protologue], 20 Feb 1876, *G. Hieronymus 812* (holotype: GOET [GOET003580]; isotypes: CORD [CORD00006115], US [US-2678278]).
Solanum tenuisectum Kuntze, Revis. Gen. Pl. 3(2): 227. 1898. Type: Argentina. western Pampas, 34 degrees, Jan 1892, *O. Kuntze* s.n. (lectotype, designated here: NY [NY00172207]; isolectotype: NY [NY00172206]).
Solanum incisum Griseb. var. *septatopilosum* C.V. Morton, Revis. Argent. Sp. Solanum 100. 1976. Type: Argentina. Catamarca: Dept. Belen, Pozo de Piedra, 1900 m, 25-31 Jan 1952, *H. Sleumer & F. Vervoorst 2375* (holotype: US [US-2173088]; isotype: LIL).
Solanum crebrum C.V.Morton & L.B.Sm., Revis. Argent. Sp. Solanum 80. 1976. Type: Argentina. Catamarca: Dept. Andalgala, Alto de las Juntas y alrededores, 1-16 Jan 1952, 2700-2830 m, *H. Sleumer 2166* (holotype: US [US-2168362]; isotypes: G[G00357861], LIL [LIL-394778]).
Solanum incisum Griseb. var. *tenuisectum* (Kuntze) C.V. Morton, Revis. Argent. Sp. Solanum 100. 1976. Type: Based on *Solanum tenuisectum* Kuntze
Solanum vervoorstii C.V.Morton, Revis. Argent. Sp. Solanum 128. 1976. Type: Argentina. Catamarca: Dept. Belen, Quebrada de los Potrerillos above El Rodeo, Granadillas, 26 Jan 1952, 2700-2830 m, *H. Sleumer & F. Vervoorst 2481* (holotype: US [US-2168145]; isotypes: G, LIL [LIL-394789]).
Solanum restrictum C.V.Morton, Revis. Argent. Sp. Solanum 128. 1976. Type: Argentina. Córdoba: Dept. Punilla, Estancia El Rosario, east of La Cumbre, Sierra de Córdoba, 20 Mar 1943, *H.H. Bartlett 20171* (holotype: US [US-2320061]).
Solanum ratum C.V.Morton, Revis. Argent. Sp. Solanum 130. 1976. Type: Argentina. Córdoba: Dept. Punilla, El Durazno, 18 Mar 1944, *C.A. O’Donell & J.M. Rodriguez V. 805* (holotype: A [A00077745]; isotypes: LIL [LIL-97232]).

##### Type.

Argentina. Mendoza: Villavicencio, *R.A. Philippi* s.n. (lectotype, designated here: SI [SI-26577]; isolectotypes: G [G00070190, F neg. 23156], SGO [SGO-42739, SGO-55501], W [W-0001341]).

##### Description.

Suffrutescent herb to small shrub, 0.5-1.5 m tall, arising from a woody rootstock. Stems slightly angled when young, sparsely to densely pubescent with simple uniseriate trichomes to 0.5 mm long, these strongly ascending and all appressed to stem, occasionally (collections from Famatina in La Rioja province, Argentina) more floccose, the trichome base enlarged and slightly bulbous; new growth glabrous or densely pubescent with simple white trichomes like those of the stems. Bark of older stems yellowish grey, glabrescent. Sympodial units difoliate to plurifoliate, if difoliate, not geminate. Leaves simple to variably pinnatifid, 2.5–10 cm long, 1–7 cm wide, more or less lanceolate to narrowly elliptic in outline, the upper surfaces glabrous or with scattered simple uniseriate trichomes at the base and along the veins, these all appressed and pointing distally, the lower surfaces glabrous to uniformly pubescent with appressed and ascending simple uniseriate trichomes < 0.2 mm long; primary veins 10–20 pairs, drying yellowish grey; base attenuate, winged along the stem; margins entire to 3–5-lobed, the lobes 0.5–3.5 cm long, 0.2–0.7 cm wide, incised to the midrib or very shallowly, in the basal part of the leaf; apex acute to acuminate; petioles very short to apparently absent, sparsely pubescent with ascending appressed trichomes on all surfaces like those of the stems, not twining. Inflorescences terminal or lateral, occasionally leaf-opposed, 1–2.5 cm long, simple or at most furcate, with 4–10 flowers in a pseudoumbel, glabrous to pubescent with ascending appressed simple uniseriate trichomes like those of the stems and leaves; peduncle 1–2.2 cm long; pedicels 0.7–1.2 cm long, filiform, ca. 0.5 mm in diameter, nodding at anthesis, pubescent like the rest of the inflorescence, articulated at the base in a very small sleeve; pedicel scars tightly packed at the tip of the inflorescence on a small platform. Buds ellipsoid to fusiform and elongate, the corolla strongly exerted from the calyx tube before anthesis. Flowers all perfect, 5-merous. Calyx tube 1–1.5 mm long, conical, the lobes 2.5–3 mm long, long-triangular to lanceolate, glabrous to pubescent with appressed white simple trichomes like those of the stems and leaves. Corolla 1–1.6 cm in diameter, violet or white, often with a green or yellowish green eye, stellate, lobed nearly to the base, the lobes 6–8 mm long, 3–4 mm wide, strongly reflexed at anthesis, densely and uniformly pubescent abaxially with minute simple uniseriate trichomes < 0.1 mm long, glabrous adaxially. Filament tube ca. 0.5 mm long, the free portion of the filaments 0.5–1 mm long, densely pubescent adaxially with tangled simple trichomes 0.5–1 mm long; anthers (3-)5–5.5 mm long, ca. 1 mm wide, ellipsoid, loosely connivent to occasionally somewhat spreading, poricidal at the tips, the pores lengthening to slits with age. Ovary glabrous; style 9–11 mm long, glabrous or pubescent with weak simple trichomes in the basal 2/3; stigma capitate, the surface minutely papillose. Fruit a globose berry, 0.5–0.7 cm in diameter, purple or reddish purple when ripe, the pericarp thin and somewhat shiny, glabrous; fruiting pedicels 1–1.5 cm long, ca. 1 mm in diameter, not particularly woody, secund and pendent from weight of fruit. Seeds ca. 20 per berry, ca. 1.5 mm long, ca. 1.5 mm wide, flattened reniform, yellowish brown, the surfaces minutely pitted, the testal cells rectangular; stone cells present. Chromosome number: n=12 (Moscone 1992).

**Figure 82.**
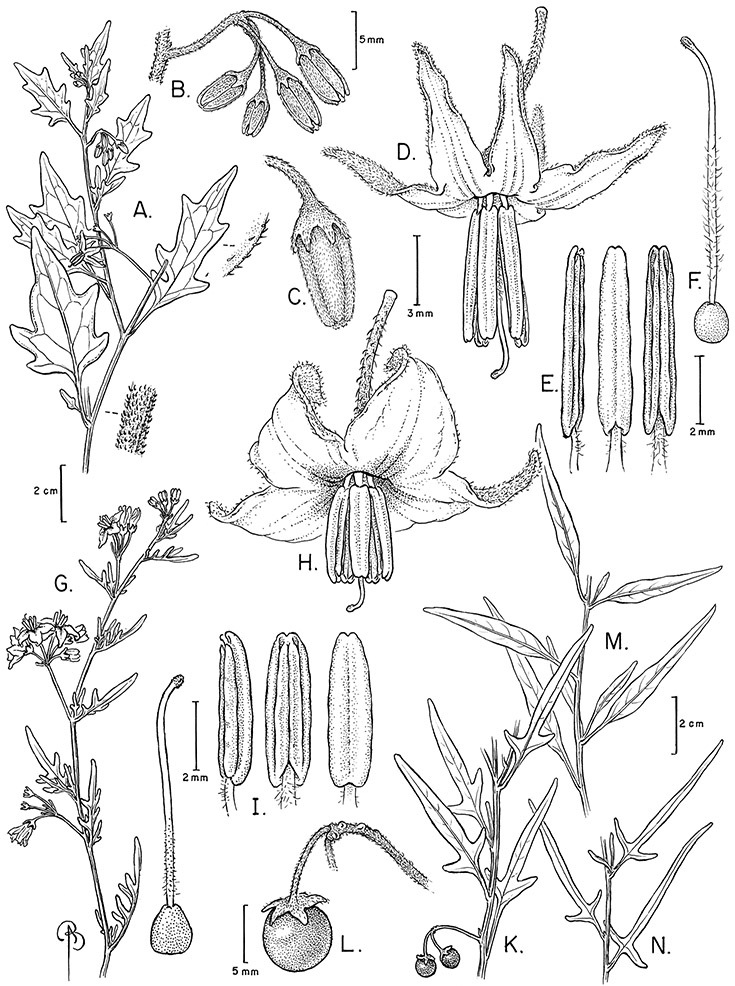
*Solanum salicifolium* Phil. (**A–F** drawn from *Varela 633* **G–J** drawn from *Keisling 7929* **K–L** drawn from *Kuntze* s.n., Dec 1891 **M** drawn from *King 151* **N** drawn from *Hieronymus* s.n., coll. 1878). Illustration by Bobbi Angell.

##### Distribution

**Figure 83.**
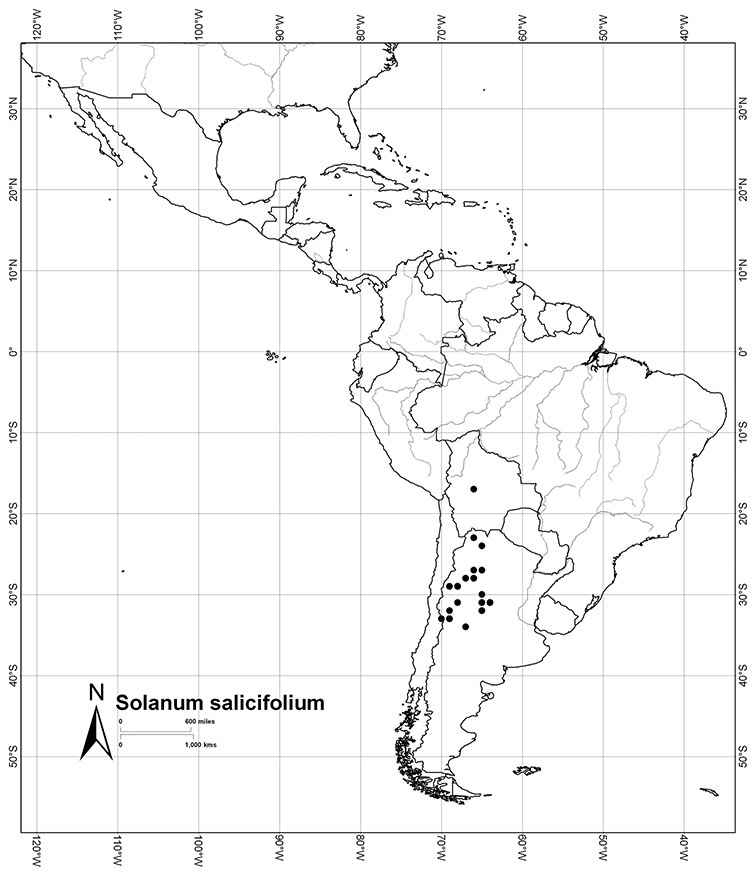
Distribution of Solanum *salicifolium* Phil.

(Figure 83). In the eastern slopes and foothills of the Andes in western Argentina, from Prov. Mendoza north to Jujuy, Córdoba and Tucumán and adjacent Bolivia; from 900–4100 m.

##### Ecology.

In dry forests and open areas.

##### Conservation status.

Least Concern (LC); EOO >100,000 km^2^ (LC) and AOO >10,000 km^2^ (LC). See Moat (2007) for explanation of measurements.

##### Discussion.

*Solanum salicifolium* is extremely (almost incredibly) variable in leaf shape (see Figure 2A), ranging from simple and linear (the type of *Solanum salicifolium*) to deeply pinnatifid with very narrow lobes (the type of *Solanum tenuisectum*). This is the extreme of similar variation in leaf shape found throughout the clade, and has led to confusion over the identity and synonymy of this species. Morton (1976) suggested that leaves with a single pair of lobes sat the base might be a late season growth form. Like *Solanum endoadenium* and *Solanum angustifidum*, also of the eastern Andean slopes in Argentina, *Solanum salicifolium* is a shrubby species, but it never appears to become vining. *Solanum salicifolium* is distinguished from *Solanum endoadenium* by it eglandular, appressed, ascending pubescence of simple trichomes with slightly bulbous bases and its usually simple inflorescences with the flowers clustered near the tip. It differs from *Solanum angustifidum* in its leaves that are lanceolate, rather than elliptic, in outline, its simple inflorescences and its anthers borne on equal filaments.

*Solanum salicifolium* is also quite similar to three species of the Morelloid clade (sensu Weese and Bohs 2007) that are traditionally segregated as section *Parasolanum* and by some included in a wider section *Dulcamara* (Nee 1999), *Solanum tripartitum* Dunal, *Solanum radicans* L.f. and *Solanum palitans* C.V.Morton. *Solanum tripartitum* and *Solanum palitans* have both been included in molecular analyses and are resolved as members of the Morelloid clade (Weese and Bohs 2007). These taxa have pedicels that are flush with the inflorescence axis, rather than inserted into a small sleeve, and the flowers are spaced along the inflorescence axis rather than being clustered at the tip on a small platform. The berries of *Solanum salicifolium* contain stone cells, a characteristic of many members of the Morelloid clade, a few of the collections (e.g., *Del Vitto & Moscone 852* from Mendoza, Argentina) I have examined have many, but more normally fruits have 1-3 of these hard, seed-like bodies. Possession of these hardened bodies in the fruits may indicate a relationship with Morelloid taxa, but the woody habit is more Dulcamaroid. As more taxa of both these groups are included in molecular phylogenetic studies of *Solanum*, their relationships may become more clear.

In describing *Solanum salicifolium* R.A. Philippi did not cite a particular herbarium specimen and potential syntype material annotated with the type locality has been widely found. I have selected the specimen in SI as the lectotype of *Solanum salicifolium*, a largely Argentinian species.

In selecting a lectotype for *Solanum incisum*, Morton (1976) stated that specimens from the locality of *Hieronymus 220*, Sierra de Achala, were a good match for the photograph of the presumed holotype destroyed in Berlin (F neg. 2779), however, the types of Grisebach’s names are generally to be found at GOET, not B. A single sheet of *Hieronymus 220* is present at GOET, making his designation unambiguous. In the Kuntze herbarium at now at NY there are two sheets annotated *Solanum tenuisectum*; I have selected that with the locality of the protologue (“western Pampas”, NY00172207) as the lectotype. Morton’s (1976) *Solanum incisum* var. *septatopilosum* was distinguished from the more typical variety by the possession of spreading trichomes, but plants collected from the same locality (e.g., *Sleumer & Vervoorst 2399*) have the typical appressed pubescence of *Solanum salicifolium*. The type of *Solanum sericeum* var. *strigillosum* (*Hieronymus 812*) represents the narrow-leaved extreme of *Solanum salicifolium*. *Solanum sericeum* Ruiz & Pav. is not a member of the Dulcamaroid clade, but its relationships are not clear.

##### Specimens examined.

**Argentina**. **Catamarca**: Ambato, entre Buena Vista y Singuil, rumbo a Catamarca, km 1438–1439, 2170 m, 29 Feb 2004, *Barboza et al. 877* (CORD); Belén, Pozo de Piedras, 11 Feb 2012, *Barboza et al. 3467* (BM, CORD); Belén, Nacimientos de San Antonio, 12 Feb 2012, *Barboza et al. 3488* (BM, CORD); Andalgalá, Andalgalá, 9 km SE on road to Cuesta de la Chilca, 1000 m, 20 Nov 1972, *Cantino 447* (CORD, GH); Pomán, Sierra de Ambato, falda O, subiendo desde El Rincón rumbo al Cerro Manchado, más arriba de Las Casitas, 2500 m, 19 Feb 1970, *Hunziker & Ariza 20476* (CORD); Belén, Nacimientos de San Antonio, quebrada de Medano Trancado, huella a Laguna Blanca, 2900 m, 22 Feb 1974, *Legname & Vervoorst 142* (LIL); Belén, Yacutula, cerca Belén, Dec 1879, *Schickendantz 45* (CORD); Andalgalá, Capillitas, o Quebrada de Coyo, 1879, *Schickendantz 91* (CORD); Belén, Las Faldas, Sierra de Belén, 2000 m, Mar 1939, *Schreiter* s.n. (GH); Tinogasta, Reales Blancas a La Franca, 3500 m, 4 Feb 1930, *Schreiter 6183* (US); Andalgalá, El Suncho, ascenso a la Sta. Ana frente de El Suncho, 1750 m, 20 Feb 1951, *Sleumer 1633* (LIL, W); Andalgalá, Rosada La Primera, Las Estancias, 1800 m, 11 Feb 1952, *Sleumer 2134* (LIL); Andalgalá, Río Pisavil, 1850 m, 13 Jan 1952, *Sleumer 2208* (LIL); Belén, orilla del Río Blanco arriba de Granadillas, 1950 m, 26 Jan 1952, *Sleumer & Vervoorst 2483* (LIL); Belén, El Rodeo, arriba de Granadillas, Quebrada del Río Blanco, 2400 m, 26 Jan 1952, *Sleumer & Vervoorst 2484* (LIL, US); Belén, Quebrada de Totoral, arriba de Granadillas, 2130 m, 26 Jan 1952, *Sleumer & Vervoorst 2492* (LIL); **Córdoba**: Río Cuarto, Estancia Aguada del Moye, 22 km al N de Achiras, Sierra Comechigones, 1000 m, 7 Oct 1980, *Anderson et al. 3769* (CORD); Cópina, Sierra Grande, 1400 m, 29 Dec 1935, *Burkart 7490* (GH); Sierra Chica de Córdoba, Agua de Oro, 3 Feb 1955, *Castellanos 3223* (CORD); Punilla, Ruta no. 29, entre Tanti y Taninga, entre los departmentos de Punilla y Pocho, 6 Mar 2009, *Chiarini 707* (CORD); Punilla, El Durazno, 3 Feb 2012, *Chiarini et al. 807* (BM, CORD); San Alberto, Parador El Condor, 4 Feb 2012, *Chiarini et al. 819* (BM, CORD); Cruz del Eje, Río Gasta, Sierra Chica, falda E, por ruta provincial 28 (ex nacional 20), 3 Dec 1983, *Cocucci 30* (CORD); Punilla, El Durazno, Sierra Grande, falda E, al sudoeste de Tanti, 1100 m, Feb, *Cocucci 108* (CORD); Cruz del Eje, Sierra Grande, falda O, ruta 20, entre Tala Cañada y refugio de Piedra, 1300 m, 29 Oct 1978, *Di Fulvio & Maldonado 539* (CORD); Ongamira, 5 Jan 1971, *Fosris 7947* (P); Minas, Cerro de Orcosu, Yerba Buena, 20 Feb 1876, *Hieronymus 429* (CORD, GOET); Minas, Cerro de Orcosu, 20 Feb 1876, *Hieronymus 813* (CORD, GOET); Sierra Chica de Córdoba, Pan de Azucar, 29 Sep 1878, *Hieronymus* s.n. (G, K); Colón, Pozo Verde, más alla de la quebrada, 13 Oct 1946, *Hunziker 6942* (G, MO); Santa María, Malagueño, falda E, Sierra Chica, 28 Oct 1947, *Hunziker 7559* (CORD); Punilla, El Dragón, entre La Falda y Huerta Grande, Sierra Chica, 5 Nov 1950, *Hunziker 8455* (CORD); Pocho, Cerro Yerba Buena, falda N, Sierra de Pocho, 17 Feb 1952, *Hunziker 9836* (CORD); Calamuchita, Dique Los Molinos, 10 Apr 1956, *Hunziker 12244* (CORD); Calamuchita, Sierra de los Cóndores, Ruta 36, entre Berrotarán y Dique Río Tercero, 20 Dec 1957, *Hunziker 13434* (CORD); Punilla, Cerro Uritorco, falda occidental, 1600 m, 15 Jan 1965, *Hunziker 18022* (CORD); Punilla, Cerro Los Gigantes, Sierra Grande, bajando desde el refugio del Club Andino, algo más arriba de El Pollito, 14 Feb 1982, *Hunziker 24188* (CORD); Sobremonte, Sierra del Norte, ca. 10 km al oeste de San Francisco del Chañar, rumbo a Lucio V. Mansilla, 680 m, 30 Jan 1987, *Hunziker & Subils 25043* (CORD); Calamuchita, Sierra de Achala, falda E, unos 2 km al NNE de Villa Alpina, cerca del Río Lapuente (=Río La Arenosa), 1300 m, 13 Feb 1987, *Hunziker 25075* (CORD); San Alberto, Cuesta de La Ensenada, 4 Jan 1895, *Kurtz 8324* (CORD); Calamuchita, Potrero Arroyo Calmayo, 6 Nov 1875, *Kurtz 8786* (CORD); Santa María, Malagueño, 19 Mar 1944, *O’Donell & Rodríguez 313* (A); Huerta Grande, Sierra Chica, 17 Feb 1897, *Stuckert 1798* (G); Casa Bamba, Sierra Chica, 7 Nov 1897, *Stuckert 3690* (G); Córdoba, 28 Dec 1898, *Stuckert 5848* (G); Córdoba, 1 May 1899, *Stuckert 6977* (G); Cuesta de Capina, Sierra de Cordoba, 3 Dec 1901, *Stuckert 10372* (G); Sierra Achala, 18 Dec 1901, *Stuckert 10698* (G); Sierra Achala, falda del radice, 20 Dec 1901, *Stuckert 10947* (G, US); Cara Bamba, Sierra de Cordoba, 16 Dec 1908, *Stuckert 19589* (G); Yacanto Calamuchita, “La Tirelire”, 1000 m, 16 Dec 1973, *Tirel 33* (G, P); Tanti, 900 m, 17 Oct 1974, *Tirel 225* (G, P); Santa María, San Clemente, Sierra Chica (faldas O), al noroeste del Dique de los Molinos, 8 Feb 1990, *Zygadlo 11* (CORD); **Jujuy**: Humahuaca, Tres Cruces, 3500 m, 25 Nov 1986, *Charpin & Ahumada 20659* (G); Humahuaca, La Soledad, 2500 m, 21 Jan 1928, *Venturi 9012* (US); **La Pampa**: Lihué Calel, Cerros de Lihué Calel, 6 Mar 1976, *Steibel 3991* (CORD); Lihué Calel, Cerros de Lihué Calel, 7 Jan 1970, *Troani et al. 540* (CORD); **La Rioja**: General Sarmiento, Las Chacritas, alrededores del campamiento de la Empresa Benito Roggio, 2650 m, 7 Feb 2001, *Barboza et al. 221* (CORD); Chilecito, Río Los Manzanos, alrededor, desvio al oeste desde la ruta provincial # 15 entre Guanchín y Sañogasta, 1730 m, 19 Feb 2003, *Barboza et al. 553* (CORD); Famatina, Los Corrales, viniendo desde Los Berros rumbo a Famatina, 2000 m, 20 Feb 2003, *Barboza et al. 588* (CORD); Famatina, entre Los Cajoncitos y Cuevas de Noroña, 3290 m, 1 Feb 2011, *Barboza et al. 2709* (CORD); Famatina, Achavil, justo cruzando Río Achavil, 2392 m, 3 Feb 2011, *Barboza et al. 2845* (CORD); Famatina, Los Corrales, 2093 m, 3 Feb 2011, *Barboza et al. 2960* (CORD); Famatima, Los Cajones, 3100 m, 18 Dec 2011, *Barboza et al. 3224* (CORD); Sanagasta, entre Dique de Los Sauces y el Observatorio del Cerro de la Cruz, 1000 m, 23 Aug 1977, *Biurrun & Pagliari 959* (CORD); General Belgrano, El Toro Muerto, Sierra de Los Qunteros, a tres cuadras del paraje El Toro Muerto, rumbo a Las Barrancas, 26 Feb 1989, *Biurrun & Pagliari 2672* (CORD); General Sarmiento, entre Jagué y Paso Pircas Negras, a 40 km del primero, quebrada en el Río Peñón, 2750 m, 5 Feb 1998, *Biurrun et al. 5121* (CORD); Famatina, Pie de la Cuesta, más arriba del Vallecito, Sierra de Famatina, 15 Jan 1879, *Hieronymus & Niederlein 642* (CORD); Tambillas, más arriba, 7 Dec 1915, *Hosseus 1009* (CORD); Sierra de Velasco, cerca de la Mina El Cantadero (=La Esperanza), 2400 m, 5 Mar 1944, *Hunziker 5193* (CORD); Famatina, Cuesta de Miranda, Sierra de Famatina, 1950 m, 20 Mar 1950, *Hunziker & Caso 4342* (CORD); Famatina, Río Amarillo, 1600 m, 11 Jan 1949, *Krapovickas & Hunziker 5097* (CORD); General Lamadrid, Los Molles, 2400 m, 24 Jan 1949 *Krapovickas & Hunziker 5503* (CORD); Cuesta de Catinsaco, 30 Jan 1906, *Kurtz 13366* (CORD); Famatina, La Mariposa, (Los Ramblones), 2100 m, 10 Feb 1906, *Kurtz 13427* (CORD); Independencia, Quebrada de Guandacol, cerca de Amanao, 10 Jan 1907, *Kurtz 14183* (CORD); Famatina, La Hoyada, 2500 m, 5 Feb 1908, *Kurtz 15062* (CORD); Lavalle, Las Trancas, Cerro Coloradito, 10 Mar 1907, *Tejada 14580* (CORD); **Mendoza**: Luján de Cuyo, Arroyo El Salto, Potrerillos, 1810 m, 27 Feb 1985, *Ambrosetti & Moscone 1477* (CORD); Las Heras, entre el Río Pichueta y Polvoredas, 2050 m, 28 Feb 1985, *Ambrosetti & Moscone 1485* (CORD); Villa Vicencio, path leading up the ridge from hotel, 18 Jan 1943, *Bartlett 19404* (GH, NY, US); Las Heras, entre Polvareda y los Tuneles, 2300 m, 13 Jan 1963, *Boelcke et al. 9873* (US); Uspallata, 2 Feb 1953, *Castellanos* s.n. (US); Las Heras, Quebrada del Toro del Centinela, 2000 m, 25 Jan 1950, *Cuezzo & Balegno 1902* (BM, G); Luján, El Carmelo, Estancia El Salto, 3 Feb 1950, *Cuezzo & Say 2586* (CORD); Las Heras, Puesto Cantera Vieja, Campo de las Hermosas, 31 Jan 1947, *Dawson & Pujals 1501* (US); Luján, El Carrizal, El Carrizal de Abajo, a orilla de calles rurales, 790 m, 27 Feb 1985, *Del Vitto & Moscone 852* (CORD); Chacoico, Feb 1902, *Guevara 6* (CORD); Las Heras, San Ignacio, 1300 m, 25 Dec 1926, *King 151* (BM); Las Heras, Quebrada del Río Toro, 12 Jan 1969, *Krapovickas & Cristóbal 14575* (US); Las Heras, Los Hornillos, 23 Jan 1886, *Kurtz 3437* (CORD); Cuesta de las Minas, 2500 m, 18 Jan 1897, *Kurtz 9389* (CORD); Cachenta, 1400 m, 25 Feb 1900, *Kurtz 10887* (CORD); Nihuel-Cañon del Atuel Proximo-San Rafael, 1250 m, 28 Jan 1975, *Lagiglia 3097* (CORD); Melocotón, 10 km al sur de Villavicencio, 2000 m, 3 Mar 1947, *Moyam* s.n. (B); Las Heras, Villavicencio, via Villavicencio a Chile, 1700 m, 2 Feb 1946, *Nicora 4330* (US); Las Heras, Villa Vicencio, 6 Nov 1951, *Ocanto 9* (CORD); Luján, Refugio San Antonio, a 5 km del Refugio, 2000 m, 3 Feb 1950, *Say & Cuezzo 2506* (CORD); Las Heras, Quebrada Potrero Puerta, 1500 m, 31 Dec 1944, *Semper 269* (A, NY); Luján, Potrerillos, rocas vecinas al Cemeterio, 16 Feb 1945, *Semper 397* (A, NY); **Río Negro**: El Cuy, Aguada Guzmán, Ruta 242, al sur de la Loma El Pangaré, para llegar a la poblacion es necesario seguir el rio, 23 Feb 1990, *Zygadlo 140* (CORD); **San Juan**: Zonda, Cerro Blanco, 28 Jan 1973, *Ariza Espinar 2798* (CORD); Caucete, Pie de Palo, a ca. 4 km del Balcón de la Virgen, rumbo a la Antena, 2974 m, 20 Dec 2007, *Barboza et al. 1929* (CORD); Valle Fértil, Peñas Rosadas, subiendo al Sierra de Valle Fertíl por el Río Durazno, entre Baldecitos e Ischigualasto, cercanias de paraje Peñas Rosadas, 1400 m, 28 Dec 1997, *Biurrun et al. 5045* (CORD); Sarmiento, Col. Pedernal, 4 Dec 1945, *Cuezzo 1658* (B); Iglesia, pasando Arroyo El Aspero por RN 150, a 250 m por camino lateral derecho, 2939 m, 19 Feb 2008, *Filippa et al. 86* (CORD); Angaco, Quebrada del Molle, Sierra Pie de Palo, subiendo por el camino al Mogote Los Corallitos, 1800 m, 28 Nov 1980, *Hunziker et al. 23669* (CORD); Angaco, Quebrada del Molle, Sierra Pie de Palo, subiendo por el camino al Mogote Los Corallitos, 2150 m, 28 Nov 1980, *Hunziker et al. 23759* (CORD); Angaco, La Aguada, Sierra Pie de Palo, subiendo por el camino al Mogote Los Corallitos, en la Quebrada El Molle, 2150 m, 19 Dec 1980, *Hunziker et al. 23865* (CORD); Iglesia, Ruta 150, 8 km al W de Guardia Vieja (Gendarmeria), 3300 m, 26 Mar 1989, *Hunziker & Gamerro 11638* (MO); Angaco, Sierra de Pie de Palo, camino de Mogota a Mogote de los Corallitos, 13 Nov 1982, *Kiesling & Sáenz 4112* (CORD); General Sarmiento, Quebrada de las Flechas y Quebrada del Río Los Sombreros, 14 Nov 1982, *Kiesling & Sáenz 4199* (CORD); Iglesia, Quebrada de Agua Negra, Ojos de Agua, 3250 m, 22 Feb 1992, *Kiesling et al. 7929* (GH, MO, MO, P); Agua Pinta, Agua Pinta-Maradona, Feb 1897, *Kurtz* s.n. (CORD); Iglesia, Quebrada de Agua Negra, Arrequintín, 21 Apr 1980, *Rotman et al. 373* (CORD); camino de San Juan a Calingasta, más o menos en la mitad del camino, *Sayago 1252* (CORD); **San Luis**: Coronel Pringles, Eleodoro Lobos, 3 km al este en Ruta 7, 840 m, 26 Dec 1969, *Anderson 1490* (CORD); Coronel Pringles, Trapiche, arriba de Río Trapiche, 1050 m, 5 Nov 1970, *Anderson 1771* (CORD); Coronel Pringles, entre Los Membrillos y Cerros Largos, 1300 m, 22 Nov 1976, *Anderson et al. 3168* (CORD); General Pedernera, Sierra El Morro, cuenca interior, 1200 m, 10 Dec 1976, *Anderson & Alliney 3268* (CORD); Coronel Pringles, Río de la Carpa, entre Los Membrillos y Paso del Rey, Sierra de San Luis, 1250 m, 20 Feb 1979, *Anderson et al. 3574* (CORD); General Pedernera, Estancia Santa Teresa, al S de Granville, 800 m, 7 Jan 1981, *Anderson 3793* (CORD); Coronel Pringles, Gruta de Incahuasi, alrededores, 1600 m, 4 Dec 1995, *Cerana 1259* (CORD); La Guardia, 23 Mar 1882, *Galander* s.n. (CORD); Quebrada de los Bueyes, 17 Mar 1882, *Galander* s.n. (CORD); Mina Carolina, 18 Mar 1882, *Galander 18* (CORD); Coronel Pringles, Quebrada de los Cóndores, 5 Dec 1979, *Guerrero 15* (CORD); Junín, Sierra de Comechingones, en la mitad superior de la falda O, subiendo frente a El Rincón, 9 Feb 1956, *Hunziker 11798* (CORD); Junín, El Rincón, al E de Merlo, camino a la vieja Calera, vecino a la toma de agua del Arroyo Carpintería, 18 Jan 1983, *Juliani* s.n. (CORD); Capital, Chichaca Grande, Cerro Varela, 9 Jan 1892, *Kurtz 7019* (CORD); Cerro Varela, 9 Jan 1892, *Stuckert 7019* (G, US); Coronel Pringles, Dique del Potrero, 1000 m, 26 Feb 1944, *Varela 633* (A); **Tucumán**: Tafí, Pinar de Los Ciervos, km 70, 6 Mar 1998, *Barboza et al. 140* (CORD); entre Tafí del Valle y Amaicha, 6 Mar 1998, *Barboza et al.. 152* (CORD); Tafí, desde Amaicha del Valle rumbo a Tafí del Valle, entre km 92-91, 13 Feb 2012, *Barboza et al. 3494* (BM, CORD); Tafí, El Molle, en el camino entre Tafí del Valle y Amaicha, km 91-93, 2800 m, 12 Feb 1986, *Hunziker et al. 24876* (CORD); Tucumán, 1 Mar 1900, *Stuckert 8669* (CORD, G); Trancas, Quebrada del Chorro, 3000 m, Apr 1926, *Venturi 7777* (GH, US).

**Bolivia**. **Cochabamba**: Tunari, 3800 m, Mar 1953, *Cárdenas 5447 A* (L); **Potosí**: Tomás Frías, carretera Potosí-Kuchu Ingenio, ca. 20 km S of Potosí, 4020 m, 18 Feb 2004, *Smith et al. 380* (F, MO); Linares, Chaqui village on exit to hot springs and puna, 3400 m, 18 Feb 1996, *Wood 10688* (K).

**Paraguay**. **Alto Paraguay**: Río Paraguay, brazo interior, 1997, *Mereles 7043* (G); **Cordillera**: Ciervo Cuá, Aug 1990, *Mereles 3938* (G); **Presidente Hayes**: Río Pilcomayo, May 1906, *Rojas 193* (G).

**Uruguay**. **Lavalleja**: Parador Salus, to the SE, near railroad, boundary of Campos de Loza, 27 Nov 1943, *Bartlett 20944* (GH).

#### 34. Solanum sanchez-vegae

S.Knapp, PLoS ONE 5(5): e10502. 2010

http://species-id.net/wiki/Solanum_sanchez-vegae

Figure 84

##### Type.

Peru. Amazonas: Prov. Chachapoyas, W side of Cerros Calla-Calla, 45 km above Balsas, mid-way on road to Leimebamba, 3100 m, 19 Jun 1964, *P.C. Hutchison & J.K. Wright 5738* (holotype: USM; isotypes: F [F-163831], K [K000545365], P [P00549320], US [US-246605], USM).

##### Description.

Woody vine or lax shrub, to 6 m tall; stems glabrous to sparsely pubescent with tangled loose dendritic trichomes 1–1.5 mm long, these multi-celled and few branched; new growth pubescent with tangled dendritic trichomes 1–1.5 mm long, occasionally almost completely glabrous; bark of older stems reddish brown, glabrescent. Sympodial units plurifoliate. Leaves simple, (2.5-)5–12 cm long, (1.3-)2.5–5 cm wide, narrowly elliptic, fleshy to chartaceous, the upper surfaces glabrous, the lower surfaces with loose dendritic trichomes to 1 mm long along the veins and occasionally extending to the lamina; primary veins 11–14, with a prominent intramarginal vein looping 1/3 of the way in from the margin, all veins impressed above; base acute to cuneate; margins entire, usually revolute; apex acute; petioles 1–3.5 cm long, stout, glabrous to sparsely pubescent, often drying dark in herbarium specimens, twining. Inflorescences terminal, to 15 cm long and very broad, branched many times, beginning very near the base, with 50–100 flowers, glabrous to sparsely pubescent with loose dendritic trichomes; peduncle to 1 cm long, the inflorescence branching very near the junction with the stem; pedicels 1.2–1.5 cm long, slender, 0.5–1 mm in diameter at the base, 1–1.2 mm in diameter at the apex, spreading at anthesis, glabrous to very sparsely pubescent, articulated at the base, leaving a prominent peg from a sleeve ca. 0.5 mm long; pedicel scars irregularly spaced, often clustered, 0.5–10 mm apart. Buds globose and becoming ellipsoid, the corolla strongly exserted from the calyx tube before anthesis. Flowers all perfect, 5-merous. Calyx tube 1–1.5 mm long, cup-shaped but abruptly narrowing from the pedicel, the lobes 1.5–2 mm long, broadly deltate and irregularly splitting, pubescent at the tips with tiny dendritic trichomes to 0.5 mm. Corolla 1.9–3 cm in diameter, lilac, stellate to stellate-rotate, lobed ca. halfway to the base, the lobes 8–10 mm long, 4–7 mm wide, planar or slightly campanulate at anthesis, densely papillate and pubescent at the tips and margins, these extending slightly along the midvein abaxially, adaxial surfaces glabrous. Filament tube < 0.2 mm long, the free portion of the filaments 0.75–1.5 mm long, glabrous and shiny; anthers 4.5–5 mm long, 1.5–2 mm wide, sagittate at the base, poricidal at the tips, the pores lengthening to slits with age. Ovary glabrous; style 7–10 mm long, glabrous and shiny; stigma capitate and bifid, the surface minutely papillate. Fruit a globose berry, 1.2–1.5 cm in diameter, black, the pericarp thin, dull and matte; fruiting pedicels 1.6–2 cm long, ca. 7 mm in diameter rat the apex with the apex markedly more dilated, apparently nodding in fruit; fruiting calyx lobes to 5 mm, woody, the margins paler. Seeds 4–6 per berry, 5.5–6 mm long, 3–4 mm wide, flattened reniform, reddish brown, the surfaces minutely pitted, the testal cells round-rectangular in outline. Chromosome number: not known.

**Figure 84.**
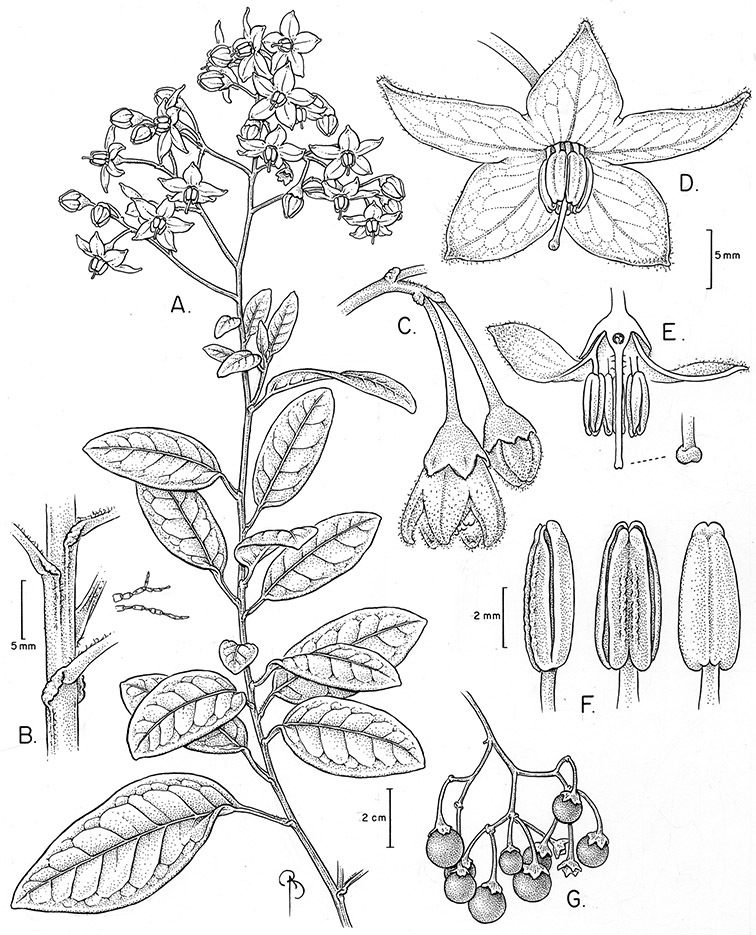
*Solanum sanchez-vegae* S. Knapp. (**A–F** drawn from *Smith & Sanchez-Vega 7524* **G** from *Hutchison et al. 5738*). Originally published in Knapp (2010). Illustration by Bobbi Angell.

##### Distribution

**Figure 85.**
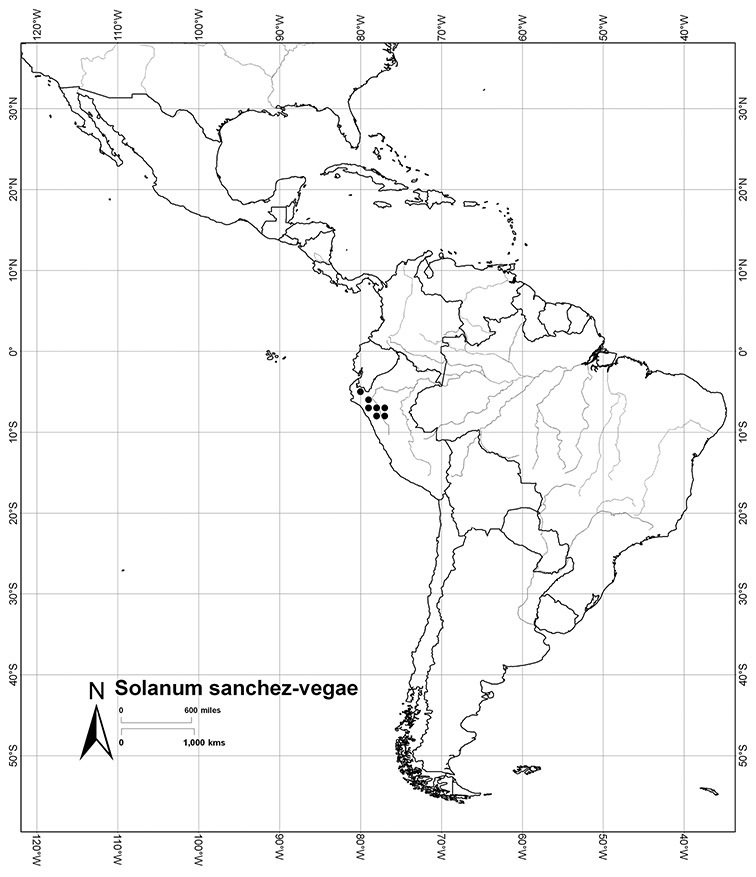
Distribution of *Solanum sanchez-vegae* S.Knapp.

(Figure 85). Endemic to the Andes of northern Peru south of the Huancabamba Depression around the middle Río Marañon valley, from 2500 to 3250 m.

##### Ecology.

*Solanum sanchez-vegae* occurs in cloud forest, montane forest (“ceja de selva” and “jalca”).

##### Conservation status.

Possible Near Threatened (possible NT); EOO <100,000 km^2^ (possible NT) and AOO >10,000 km^2^ (LC). See Moat (2007) for explanation of measurements.

##### Discussion.

*Solanum sanchez-vegae* is a striking species, with large purple flowers and shiny rubbery leaves. It was long subsumed in the more common and widely distributed *Solanum aureum*, with which it is very similar. *Solanum aureum* differs from *Solanum sanchez-vegae* in its smaller flowers, generally denser more congested pubescence of dendritic trichomes with many small, short branches (as opposed to loose dendritic trichomes with larger branches) and more northerly distribution. The ranges of *Solanum aureum* and *Solanum sanchez-vegae* slightly overlap in northern Peru, but in general *Solanum aureum* is an Ecuadorian species. I have previously identified specimens of *Solanum sanchez-vegae* as *Solanum aligerum*, a shrubby member of the clade with similar large, open inflorescences, but *Solanum aligerum* has white flowers and tufts of dendritic trichomes in the vein axils, rather than purple flowers and dendritic trichomes along the veins. *Solanum sanchez-vegae* also resembles the Venezuelan species *Solanum dichroandrum*, with which it shares loose pubescence and relatively large flowers; it differs from *Solanum dichroandrum* in its much larger (to 3 cm rather than to 2.5 cm) purple flowers, glabrous style and few-seeded berries.

##### Specimens examined.

**Ecuador**. **Azuay**: Cuenca-Soldanos road, following the N bank of the Río Yanuncay, 23 km W of San Joaquin, 3110 m, 22 Jun 1989, *Dorr & Valdespino 6415* (US); **Loja**: Nudo de Sabanilla, W slope, ca. 8 km above Yangana on road to Valladolid, 2300 m, 2 Apr 1985, *Harling & Andersson 23518* (NY).

**Peru**. **Amazonas**: Chachapoyas, Ipana, Balsas-Leimebamba road, km 406, 4 Jun 1977, *Boeke 1927* (MO); Chachapoyas, Calla-Calla, 3200 m, 29 Jul 1991, *Mostacero L. et al. 2619* (MO); Chachapoyas, Atuén, Chuquibamba, 3800 m, 18 Jul 1995, *Quipuscoa S. & Bardales 187* (BM, F, MO); Chachapoyas, Cerros Calla-Calla, middle eastern slopes, near kms 411-416 of Leimebamba-Balsas road, 3100 m, 11 Jul 1962, *Wurdack 1314* (K, USM); **Cajamarca**: San Miguel, El Tingo, en los alrededores, Dist. Unión Agua Blanca, 2930 m, 9 Feb 2000, *Alvítez I. et al. 1057* (F); Jaén, Lanchal, La Cocha, Dist. Sallique, 3300 m, 28 Jun 1998, *Díaz et al. 9717* (MOL, USM); San Ignacio, base de Cerro Picorana, Dist. San José de Lourdes, 25 Aug 1999, *Díaz et al. 10743* (MO); San Miguel, El Tingo, alrededores (Agua Blanca), 2950 m, 5 Jul 1986, *Mostacero L. et al. 1326* (BM, F); San Miguel, El Tingo, (Agua Blanca), 2750 m, 12 May 1977, *Sagástegui et al. 8804* (MO); Contumazá, Contumazá-Cascabamba, 2700 m, 12 Jun 1981, *Sagástegui et al. 9994* (BM, MO); Cajamarca, SAIS, José Carlos Mariátegui, km 20-40 on Sunchubamba-San Juan road, 3300 m, 5 Jun 1984, *Smith & Sánchez Vega 7524* (BM, MO, USM); Contumazá, sobre la ruta Salcat, Cascabamba-Pampa de la Sal, 3300 m, 30 Jun 1983, *Sánchez Vega 3142* (F, MO); **La Libertad**: Santiago de Chuco, Cachicadán, Cerro La Botica, 2900 m, 9 Jun 1953, *López M. 1011* (US); Santiago de Chuco, Cachicadán, 2700 m, 14 Jun 1984, *Smith & Sánchez Vega 11894* (MO); Sanchez Carrión, Molino Grande, alrededores de Huamachuco, 3100 m, 22 May 2001, *Sagástegui & Zapata 16535* (BM); Santiago de Chuco, Cachicadán, 2900 m, 9 Jun 2001, *Smith & Sánchez Vega 16631* (BM, F); Bolivar, junction of Quebrada Misquichilca and Quebrada Quisuar, 4 km SE Condormarca, 3500 m, 5 Jun 1986, *Young 3554* (K, USM); **Piura**: Chichinpampa, 2660 m, 25 Jun 2009, *Aedo 16670* (MA, USM); Huancabamba, Procedencia: Cruz Blanca-Turnalina, 2600 m, 5 Sep 1981, *López M. & Ramírez 8926* (BM); **San Martín**: Huallaga, valley of Río Apisoncho [=Abiseo] 30 km above Jucusbamba, 3600 m, 2 Sep 1965, *Hamilton & Holligan 551* (K).

#### 35. Solanum seaforthianum

Andrews, Bot. Repos. tab. 504. 1808

http://species-id.net/wiki/Solanum_seaforthianum

Figure 86

Solanum cyrrhosum Dunal, Solan. Syn. 9. 1816. Type: Venezuela. Sucre: Cumaná, 1799-1800, *A. Humboldt & A. Bonpland* s.n. (lectotype, designated here: P-Bonpl. [P00136348, Morton neg. 8159]; isolectotype: P-Bonpl. [P00136349, Morton neg. 8160]).
Solanum salignum Willd., Syst. Veg., ed. 15 bis [Roem. & Schultes], 4: 663. 1819. Type: “In America meridion.”, *A. Humboldt & A. Bonpland* s.n. (holotype: B-W [B-W-4335, F neg. 2894, IDC microfiche 271-315.298:III.3]).
Solanum prunifolium Roem. & Schult., Syst. Veg., ed. 15 bis [Roem. & Schultes], 4: 662. 1819. Type: Venezuela. Distrito Federal: Caracas, *F. Bredemeyer* s.n. (holotype: B-W 4321 [IDC microfiche 271-315.297:II.8]; isotype: W).
Solanum venustum Kunth, Ind. Sem. Hort. Berol. 1845: 10. 1845. Type: Germany: Cultivated in Berlin, from tropical America (probably destroyed at B, no specimens found).
Solanum tenuifolium Dunal, Prodr. [A.P. de Candolle] 13(1): 87. 1852. Type: Venezuela. Distrito Federal: Caracas, 1830, *Vargas 246* (lectotype, designated here: G-DC [G00144960, F neg. 6741, IDC microfiche 800-61.2068:II.2]).
Solanum botryophorum Ridl., J. Linn. Soc. 27: 50. 1890. Type: Brazil. Pernambuco: Ins. Fernando Noronha, Sapate and east hills, *H.N. Ridley, T.S. Lea & G.A. Ramage s.n. [68]* (lectotype, designated here: BM [BM000887992]; isolectotypes: BM [BM000815913], K [K000590078, K000590079]).
Solanum seaforthianum Andrews var. *disjunctum* O.E.Schulz, in Urb., Symb. Antill. 6: 169. 1909. Type: Cuba. Santa Clara: Distr. Cienfuegos, Cieneguita, *R. Combs 35* (lectotype, designated here: GH [GH00077562]; isolectotypes: K [K000196456], MO [MO-2089893], F [F-357862]).
Solanum kerrii Bonati, Bull. Soc. Bot. Genève, sér. 2, 5: 309. 1914. Type: Thailand. Sirincha, 22 Sep 1911, *A. Kerr* s.n. [2092] (holotype: P [P00055352]; probable isotype: BM [BM000886112, as *Kerr 2092*]).

##### Type.

England. Cultivated from seeds from the West Indies, no specimens known (lectotype, designated by Symon 1981, pg. 67: Andrews, Bot. Repos. tab. 504. 1808).

##### Description.

Woody vines, twining by the petioles. Stems terete, glabrous or sparsely pubescent with white simple uniseriate trichomes ca. 0.2 mm long; new growth glabrous or with a few simple uniseriate trichomes on the stems, often drying black. Bark of older stems brown to reddish brown. Sympodial units plurifoliate. Leaves simple or more often pinnatifid to deeply pinnatifid with up to 4 pairs of leaflets, (2-)3.5–10(-13) cm long, (1-)2–9(-11) cm wide, elliptic to broadly triangular in outline, widest in the basal third, membranous, the upper surfaces glabrous or with tiny simple uniseriate trichomes on the veins and margins, these sometimes extending to the lamina near the base, the lower surfaces glabrous; primary veins 4–6 pairs, in lobed leaves corresponding to the number of lobes, often drying yellowish brown; base acute, truncate or slightly cordate, occasionally oblique and asymmetric; margins less commonly entire, usually 3–7 lobed, the lobes to 5 cm long, 2 cm wide, smaller basiscopically; apex acute to acuminate; petioles 1–4 cm long, adaxially pubescent in a tiny groove with tiny simple trichomes like those of the upper leaf surfaces, twining. Inflorescences terminal, later lateral, to 25 cm long or more, with many open, divaricate branches, with up to 100 or more flowers, glabrous; peduncle to 6 cm long; pedicels 0.8–1.4 cm long, ca. 0.5 mm in diameter at the base, ca. 1 mm in diameter at the apex, glabrous, articulated at the base from a small sleeve, leaving a small peg on the inflorescence axis; pedicel scars irregularly spaced 2–6 mm apart, closer together near the tips of the inflorescence branches. Buds globose, slightly inflated, the corolla strongly exserted from the calyx tube long before anthesis. Buds globose, slightly inflated, the corolla strongly exserted from the calyx tube long before anthesis. Flowers all perfect, 5-merous. Calyx tube ca. 0.5 mm long, flattened and open, the lobes < 0.2 mm long, mere apiculae from the entire rim, glabrous with tufts of tiny simple trichomes to 0.2 mm on the apiculae. Corolla 1.1–2.5 cm in diameter, violet or pale violet, stellate, lobed 1/2 to 2/3 of the way to the base, the lobes 5–9 mm long, 3–4.5 mm wide, spreading or slightly cupped at anthesis, densely and minutely pubescent on the tips and margins, the trichomes simple, uniseriate, tangled, otherwise glabrous. Filament tube minute, the free portion of the filaments markedly unequal, the longest filament 2–3 mm long, elongating as anthesis progresses, the other four 1–1.5 mm long, quite variable in length, occasionally with two filaments longer, all glabrous; anthers 2–3 mm long, 1–1.5 mm wide, occasionally one anther slightly larger, ellipsoid, loosely connivent, yellow, poricidal at the tips, the pores not markedly lengthening to slits with age. Ovary glabrous; style 7–10 mm long, strongly curved away from the anther on the long filament, glabrous, sometimes violet; stigma capitate, the surface minutely papillose. Fruit a globose berry, 0.8–1.4 cm in diameter, bright shiny red when ripe, glabrous, the pericarp thin; fruiting pedicels 0.8–1.4 cm long, ca. 1 mm in diameter at base and apex, pendent from weight of berries, not markedly woody. Seeds 4–20 per berry, 4–4.5 mm long, 2.5–3 mm wide, flattened-reniform, pale yellowish tan, the surfaces minutely pitted, the testal cells pentagonal, the lateral cell walls elongate to 0.5 mm long, leaving a wing of ca. 0.5 mm around the seed and the seed appearing pubescent. Chromosome number: n=12 (Sarkar et al. 1980).

**Figure 86.**
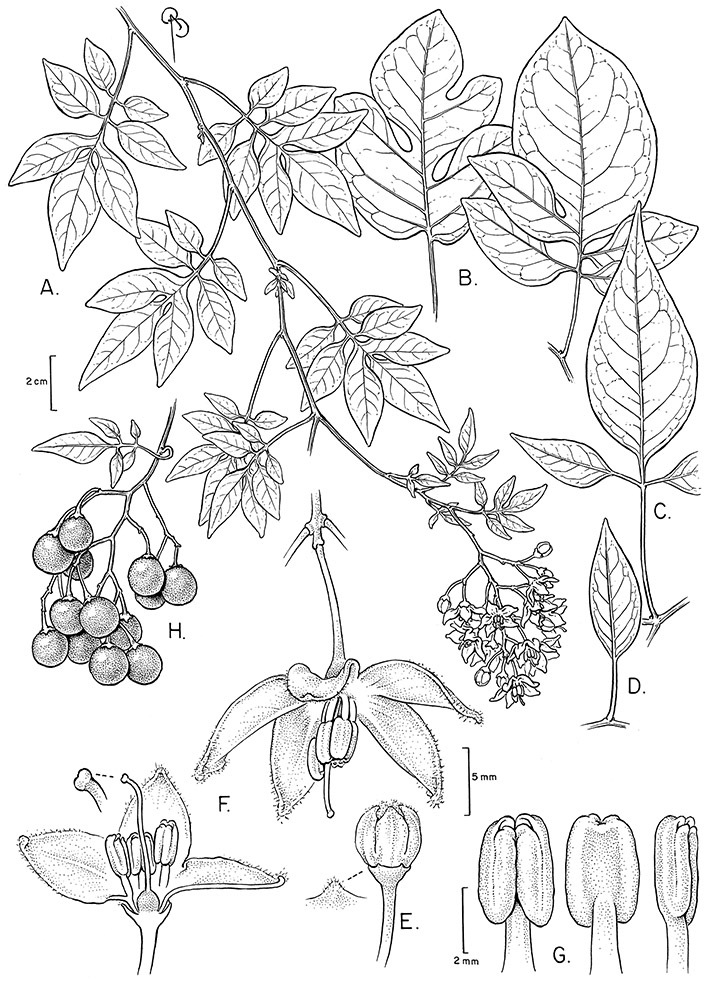
*Solanum seaforthianum* Andrews. (**A** drawn from *Baker 10374* **B** H drawn from *Thompson 947* **C** drawn from *Hatschbach 60388* **D** drawn from *Renderos 517*; E-g drawn from *McVaugh 20220*). Illustration by Bobbi Angell.

##### Distribution

**Figure 87.**
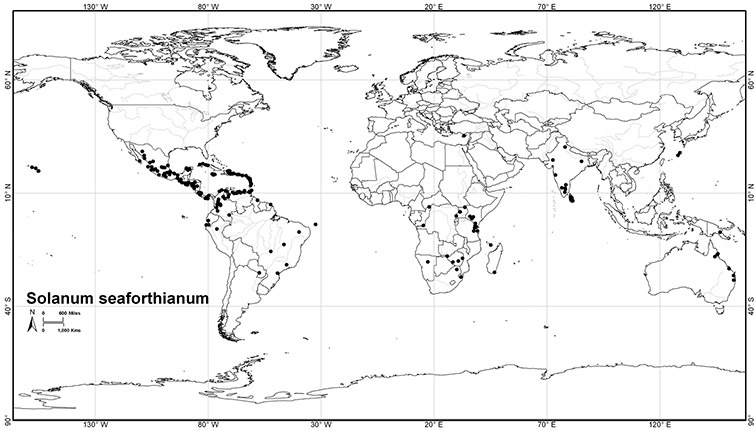
Distribution of *Solanum seaforthianum* Andrews.

(Figure 87). Probably native to the islands of the West Indies and coastal northern South America in Colombia and Venezuela, perhaps also on the Caribbean slope of Central America and Mexico; widely cultivated in the tropics and subtropics (often escaped and apparently naturalised).

##### Ecology.

Dry forests and thorn scrub in its native range; cultivated in the tropics and subtropics in a wide variety of habitats.

##### Common names:

Haiti: lilas (*Ekman H-403*); Colombia: doncenon (*Dugand 280*). Venezuela: alegría de viajero (*Steyermark 62349*)

##### Conservation status.

Least Concern (LC); EOO >100,000 km^2^ (LC) and AOO >10,000 km^2^ (LC). See Moat (2007) for explanation of measurements.

##### Discussion.

*Solanum seaforthianum* is most similar to the Mexican and Central American *Solanum dulcamaroides*, with which it shares large inflorescences, globose, slightly inflated buds, and flowers with fleshy, keeled lobes. *Solanum seaforthianum* is easily distinguished from that species in its mostly pinnate leaves (rather than mostly simple on flowering stems) that are almost completely glabrous, and its unequal filaments. The anthers of *Solanum dulcamaroides* are unusual in that they have a thick, papillate abaxial surface, not present in *Solanum seaforthianum*. Unequal filaments are also found in the somewhat similar species *Solanum flaccidum* of southern Brazil and the widespread but morphologically quite distinct *Solanum uncinellum*. *Solanum seaforthianum* can be easily distinguished from *Solanum uncinellum* by its glabrous pinnate leaves, ellipsoid (rather than elongate and pointed) buds, ellipsoid rather than tapering anthers, and its broader corolla lobes. It differs from *Solanum flaccidum* in its bright red (rather than purple) berries and the glabrous, mostly pinnate or pinnatifid leaves. The native distributions of these taxa do not overlap, but as *Solanum seaforthianum* is often cultivated, it can be found in a variety of regions, both cultivated and naturalised.

The leaves of *Solanum seaforthianum* are most usually pinnate or pinnatifid and only rarely simple, the reverse of the case in many other species of the Dulcamaroid clade, except *Solanum angustifidum*. *Solanum seaforthianum* differs from *Solanum angustifidum* in having broad, rather than narrow, leaf lobes and glabrous filaments. In other taxa juvenile leaves are pinnate (see *Solanum dulcamaroides*), so it may be that the pinnate leaves of *Solanum seaforthianum* that persist on mature stems are neotenic in nature. The mechanism by which leaf shape is regulated in this group, however, has not been investigated.

*Solanum seaforthianum* is widely cultivated in tropical areas for its showy flowers in large inflorescences, as is *Solanum laxum* from southern South America. The two taxa are easily distinguished by leaf shape (pinnate versus simple), pubescence (glabrous versus pubescent with tufts of trichomes in the vein axils) and berry color (red versus purple). The corollas of *Solanum seaforthianum* are more deeply divided than those of *Solanum laxum*, and are usually purple rather than white in cultivation. *Solanum seaforthianum* appears to have escaped from cultivation in both Australia and South Africa, but the extent of its spread is not clear at present.

**Figure 88.**
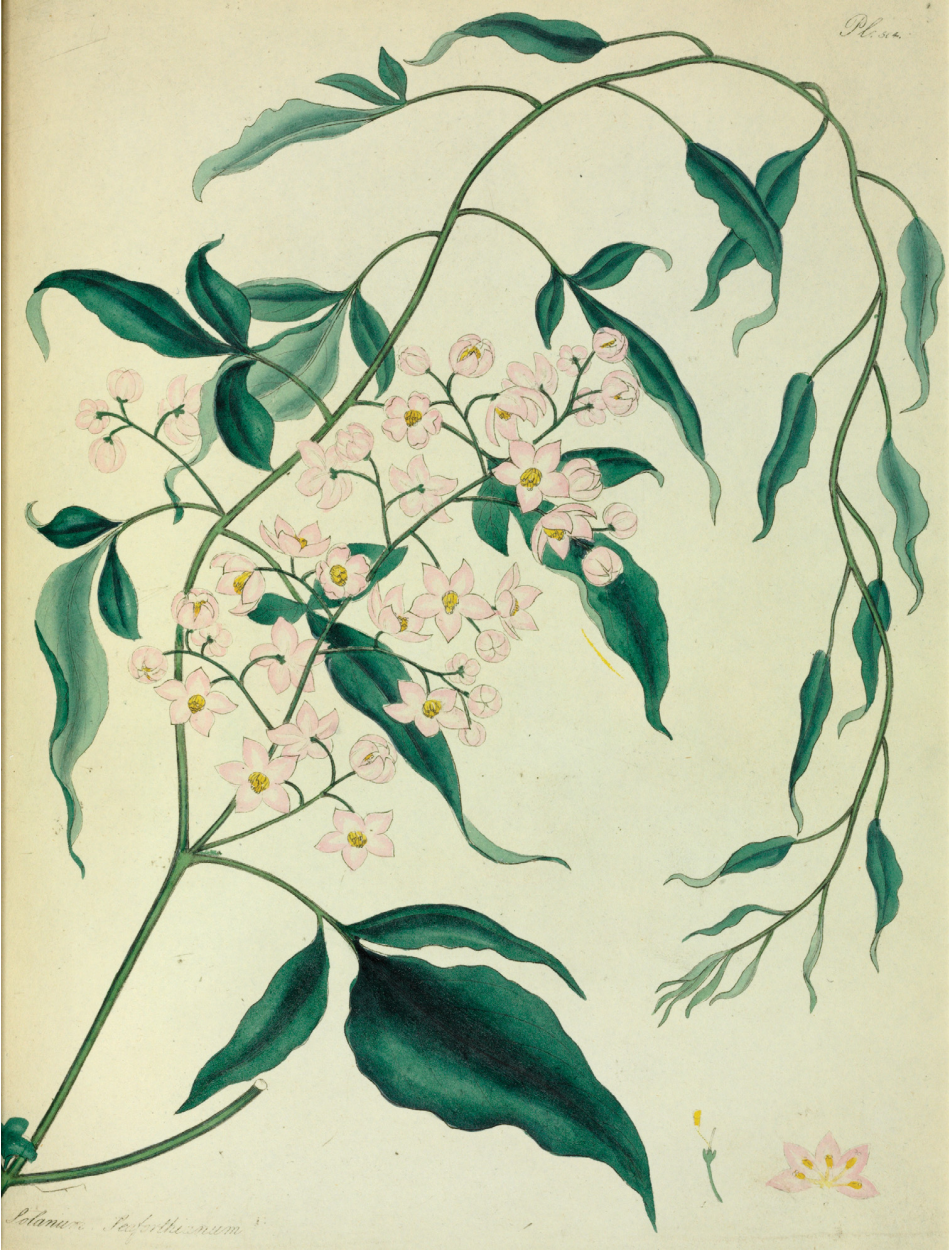
Lectotype of *Solanum seaforthianum* Andrews (Andrews 1808: tab. 504) Reproduced with permission of the Natural History Museum Botany Library.

No original material traceable to the protologue of *Solanum seaforthianum* exists; Symon (1981) used the original plate (see Figure 88) as the lectotype.

Both *Solanum cyrrhosum* and *Solanum salignum* were based on collections made by Alexander von Humboldt and Aimé Bonpland in northern Venezuela. It is likely that these are based on the same gathering (see Lack 2004; Knapp 2007) but in the absence of direct evidence I have considered them to be heterotypic; the sheets in P and B-W differ in leaf morphology and may not be from the same plants. I have lectotypified *Solanum cyrrhosum* with the more completely labelled of two sheets in P-Bonpl. (P000136348). The spelling of *Solanum cyrrhosum* (e.g., Nee 1999) has sometimes been un-necessarily corrected to “cirrhosum”.

Of the many syntypes of *Solanum tenuifolium*, the sheet in G-DC of *Vargas 246*, collected in 1830, has a label in Dunal’s hand saying “Solanum tenuifolium nob.”, making it the logical choice for a lectotype.

Five syntypes were cited in the protologue of *Solanum seaforthianum* var. *disjunctum*, two from Cuba (*Combs 35*, *van Hermann 5080*), two from Haiti (*Buch 80*, *Buch 467*) and one from St. Jan (US Virgin Islands, *Raunkiaer 3127*), all housed in B, all now destroyed; I have found multiple duplicates of only one of these (*Combs 35* from Cuba) and the sheet at GH (GH00077562) is here selected as the lectotype.

The holotype of *Solanum kerrii* in P bears no locality information, but of three duplicates in BM (the first set of Kerr’s collections) only one, *Kerr 2092* collected in 1911, is possible type material; the other two sheets were collected in December 1914 (in Chiang Mai) and 1921 (without locality), too late to have been used by Bonati in 1914.

##### Specimens examined [countries of putative native range].

**Belize**. Bot. Centre, Sep 1894, *Moloney 115* (K);

**Bermuda**. Fort Hamilton, Pembroke, 29 May 1970, *Manuel 1151* (A);

**Brazil**. **Ceará**: Ceará, Brejo Grande, western side of Serra de Araripe, Jul 1839, *Gardner 2427* (BM, K); **Goias**: Aragarças, ca. 3 km N of Barra do Garças, 1 Mar 1968, *Philcox & Fereira 4468* (K); **Minas Gerais**: Três Corações, Rod. 3 Corocoes-S. Tomé das Letras, 4 Feb 1973, *Hatschbach & Ahumada 31248* (F); **Paraná**: Curitiba, Passeio Público, 29 Apr 1969, *Hatschbach 21450* (F); Curitiba, 1 Jun 1974, *Hatschbach 34464* (BH); **Pernambuco:** Fernando do Noronha, from East Hills, 22 Sep 1887, *Ridley et al. 69* (BM); **Rio Grande do Sul**: Porto Alegre, 1955, *Rambo 57199* (B); **São Paulo**: Horto Faculdade Farmácia, 27 Sep 1933, *Kuhlmann SP-10694* (F); **Tocantins**: Aurora de Tocantins, Lavadeira, rodovia a Campos Belos a Taguatinga, 11 Feb 1994, *Hatschbach et al. 60388* (G).

**British Virgin Islands**. **St. Croix**: Mt. Stewart, 19 Jan 1961, *Howard 15285* (A); **St. Kitts**: sin. loc, *Walsh* s.n. (K); **St. Lucia**: Vigie, Castries, 4 Jun 1986, *Pierre 334* (A); Choisuel, 250 m, 26 Feb 1986, *Slane 827* (A); Morne, 243 m, May 1968, *Sturrock A-39* (A).

**Colombia**. **Antioquia**: Medellín, 1500 m, 30 Jun 1930, *Archer 373* (US); Medellín, Aug 1943, *Brother Daniel 2956* (US); Medellín, alrededores, 570 m, 25 Oct 1947, *Gutiérrez V. et al. 1457* (US); Am Rio Medellin, 1500 m, Sep 1884, *Lehmann 231* (BM); Antioquia, 2.5 km NW on road to Turbo, bewteen km 79 and 80, 700 m, 4 Jul 1971, *Nee &Mori 4283* (US); **Atlantico**: Usiacurí, vicinity of Baranquilla, 250 m, Jul 1927, *Brother Elias 243* (US); **Bolívar**: Hacienda de Coloncito, near Turbaco, 200 m, 9 Nov 1926, *Killip & Smith 14344* (US); **Caldas**: Chinchiná, Centro Nacional de Investigaciones de Café, 1360 m, 27 Sep 1948, *Hawkes 408* (US); **Cauca**: Dagua valley, 800 m, *Lehmann 4711* (F, US); **Cundinamarca**: Guaduas, alrededores y orillas del Río San Francisco, 1000 m, 5 Nov 1945, *García-Barriga 11753* (US); Bogotá, La Mesa, 1200 m, *Triana* s.n. (US); Prov. de Tequendama desde Laclusa hasta Anapoima, 700 m, 1854, *Triana 3855 /48* (BM); **Huila**: Altamira, 1920, *Brother Ariste-Joseph A 733* (US); **Magdalena**: Manuare, 700 m, 29 Apr 1944, *Haught 4128* (US); Parque Nacional Tayrona, trail to Pueblito, 150 m, 24 Oct 1972, *Kirkbride 2519* (US); **Meta**: Villavicencio, Martson Bates garden, 16 May 1944, *Fosberg 21924* (US); **Norte de Santander**: Ocaña, *Engel* s.n. (LE); between Pamplonita and Chinacota, Río Pamplonita valley, 1300 m, 17 Mar 1927, *Killip & Smith 20747* (US); **Valle del Cauca**: Lobo Guerrero, Cordillera Occidental, vertiente occidental, 610 m, 9 Sep 1944, *Cuatrecasas 17780* (F); hoya del Río de la Vieja, entre Piedra de Moler y Alto del Dinde (entre Cartago y Alcalá), 1170 m, 17 Nov 1946, *Cuatrecasas 22986* (F, US); Barranquilla, 13 Dec 1932, *Dugand 280* (F); Palmira, 27 Jun 1982, *Murphy & Parra 610* (US).

**Costa Rica**. **Alajuela**: Cuesta Colorada, Río Itiquis, Finca Las Lluvias, 840 m, 19 Nov 1988, *Grayum et al. 9110* (MEXU); Cantón de Alajuela, Valle Central; El Roble a Río Ciruelas; a la par del río, donde cruce el camino a Guácima, 800 m, 29 Sep 1993, *Hammel et al. 19409* (MO); **Heredia**: Belén, Cuenca de Taracoles, San Vicente, 900 m, 23 Dec 2002, *González & González 2481* (G); Cantón de Santo Domingo, Town of Santo Domingo, 1150 m, 2 Sep 1990, *Solomon 19144* (BM); **Limón**: Limón, 14 Dec 1881, *Lehmann 1062* (G); Zu Garten bei Limón, Dec 1881, *Lehmann 1012* (BM, K, US); vicinity of Guápiles, 300 m, 12 Mar 1924, *Standley, P.C*., *37368* (US); **San José**: Cantón de Pérez Zeledón, Cordillera de Talamanca, San Isidro de El General, Repunta, Finca de Miguel Quesada, 700 m, 1 Aug 1993, *Aguilar & Quesada 2075* (MO); San José, Dec 1984, *Pittier & Durand 9097* (BM); San José, 1100 m, Feb 1896, *Smith 6671* (US); San José, près la station de San José, 1135 m, Jun 1893, *Tonduz 8024* (G, US); San José; Jardin de l’Observatoire, Jul 1896, *Tonduz 9097* (G, LE, US).

**Cuba**. **Cienfuegos**: sin. loc, Jun 1928, *Brens* s.n. (L); Cieneguita, 13 Dec 1895, *Combs 35* (K); Harvard Tropical Garden, Soledad, Cienfuegos, 29 Sep 1927, *Jack 5518* (A); **La Habana**: Santiago de las Vegas, 30 Sep 1908, *Baker* s.n. (B); near Morrano, 8 Jan 1905, *Hermann 454* (F); Santiago de las Vegas, 9 Dec 1905, *Hermann 5080* (F); **Pinar del Río**: Viñales, 15 Jul 1950, *Brothers Alain & Clemente 1481* (GH); Mogote de la Bandera, Viñales, 19 Sep 1953, *Brother Alain 3525* (GH).

**Dominica**. Near road at margin of cultivated area between Wesley and Woodford Hill, 19 Aug 1965, *Ernst 2091* (MO); Roseau, Botanic Gardens, 30 May 1940, *Hodge 3913* (GH); sin. loc, 1841, *Hoskin* s.n. (K).

**Dominican Republic**. **Distrito Nacional**: Santo Domingo, 24 Jan 1899, *Millspaugh 858* (F); **La Altagracia**: San Rafael de Yuma, 1 km from bridge over Río Duey, on road to Los Negros, 50 m, 6 Feb 1981, *Zanoni et al. 10811* (JBSD); Loma el Peñon, 21 klm oeste de Higuey en el camino a El Seibo, al norte del pueblo Bejucalito, 200 m, 22 Oct 1981, *Zanoni & Mejía 17351* (JBSD); **La Romana**: La Romana, El Gato, 50 m, 15 Nov 1973, *Liogier 20749* (JBSD); **La Vega**: Constanza, a la derecha del cruce Culo de Maco, Cordillera Central, 1700 m, 15 Apr 1998, *Veloz et al. 1265* (JBSD); Loma El Campanario, Cordillera Central, 4 km oeste de La Culata de Constanza, 1800 m, 8 Sep 1982, *Zanoni et al. 23226* (JBSD); **Monte Cristi**: Distr. of Monción. Cultiv, 30 m, 26 Jun 1931, *Valeur 681* (F, G, GH, K, MO); **Peravia**: Loma Redonda, al N de El Naranjal (de San José Ocoa), Cordillera Central, 600 m, 21 Jun 1984, *Zanoni et al. 30778* (JBSD); **Puerto Plata**: Imbert, Arroyo Seco, area 9, km 2, sitio de la concession de explotación minera La Potranca, 28 Nov 2000, *Bastardo & Clase 1245* (JBSD); Sosúa, Parque Nacional El Choco, secc. Cabarete, en la parte central del parque, 200 m, *Clase et al. 265* (JBSD); Río Muñoz, 20 Jun 1887, *Eggers 2561* (GOET); Isabel de Torres, estribo sur, 780 m, 20 Sep 1975, *Liogier & Liogier 23941* (JBSD); Isabel de Torres, estriba este, 750 m, 11 Feb 1977, *Liogier & Liogier 26442* (JBSD); Cabarete, El Choco, 50 m, 12 Feb 1977, *Liogier & Liogier 26487* (JBSD); Sosúa, Paraje La Catalina, Sección Cabarete, orilla del Río Catalina, 80 m, 10 May 1999, *Ángeles & Clase 119* (JBSD); **San Pedro de Macorís**: en la orilla de la carretera San Pedro-La Romana, lado oriental del puente sobre el Río Soco, 15 m, 29 Oct 1992, *Zanoni et al. 47042* (B, JBSD); **Sánchez Ramírez**: Los Haiteses, 2 hours on foot from Las Cien Tareas of Cotuí, 16 Aug 1980, *Dod* s.n. (JBSD); Los Haiteses, 2 hours on foot from Las Cien Tareas of Cotuí, 16 Aug 1980, *Dod* s.n. (JBSD).

**Ecuador**. **Guayas**: Cantón Guayaquil, Guayaquil, *Mille 971* (F); Cantón Guayaquil, Guayaquil, and vicinity, 1964, *Valverde 123* (US); **Loja**: road Catacocha-Macará, ca. 12 km SW of Catacocha, 1400 m, 8 Nov 1977, *Harling et al. 15217* (MO).

**El Salvador**. **Ahuachapán**: El Imposible, San Francisco Menéndez, montaña de la division, 12 Aug 1994, *López ISF-00388* (LAGU); **La Libertad**: Jardin Botánico La Laguna, zona 1, San Salvador, *Flores 00133* (LAGU); Jardin Botánico La Laguna, zona 24, 805 m, 21 Aug 1998, *Renderos 517* (BM, LAGU, MA); Plan de La Laguna, 805 m, 18 Sep 1989, *Villacorta 403* (LAGU).

**French Guiana**. Cayenne, vic, 7 Jul 1921, *Broadway 833* (US).

**Guadeloupe**. **Basse-Terre**: Route de la Traversée, 17 Feb 1976, *Sastre et al. 4306* (A); **Grande-Terre**: Morne a L’Eau, 14 Jan 1982, *Jeremie 983* (A); **Les Saintes**: Terre-de-Haut, 10 m, 16 Apr 1984, *Defferrard 4506* (G); Terre de Haut, Fort Napoleon, 31 Dec 1986, *Sastre 8297* (K).

**Guatemala**. **Guatemala**: Universidad del Valle de Guatemala, 1500 m, 18 Oct 1994, *Poll & Escobar* s.n. (BM); **Quetzaltenango**: El Palmar, 11 Feb 1906, *Kellerman 5879* (MEXU); **Retalhuleu**: Champerico, Nov 1877, *Bernoulli & Cario 2343* (GOET); San Felipe, 625 m, Apr 1892, *Donnell Smith 2677* (K); **Santa Rosa**: Santa Rosa, 914 m, Sep 1892, *Heyde & Lux DS-3821* (G, K).

**Guyana**. Military Burial Ground, Aug 1912, *Mansfield* s.n. (K); Demerara, *Parker, C.S*., *s.n.* (K x2); Bel’Air, Jun 1923, *Persaud 305* (F, G).

**Haiti**. **Dept. du Sud**: Port-a-Piemont, 27 Jul 1917, *Ekman H-403* (G); **L’Artibonite**: Gros Morne, street of Gros Morne, 235 m, 17 Feb 1926, *Leonard 9844* (GH).

**Honduras**. **Comayagua**: Siguatepeque, 1050 m, 5 Jul 1936, *Yuncker et al. 5685* (G, K); **El Paraíso**: La Lodosa, por el camino hacia el Valle de Jamastrán, ca. 10 km al sureste de Danlí, 900 m, 10 Oct 1994, *Linares & Metsger 1783* (MEXU); **Francisco Morazán**: Ojojona 24 km SO de Tegucigalpa, 1033 m, 3 Aug 1985, *Bustillo 21* (B, BM); Tegucigalpa, Colonia 15 de Septiembre, 950 m, 7 May 1983, *Guerra C. 181* (MEXU); Campus of EAP El Zamorano, 800 m, 6 Jun 2001, *Molina R. & Molina 35157* (MEXU); Tegucigalpa, Barrio Reparto Arriba, 950 m, 12 Oct 1982, *Montoya 75* (MEXU); Montaña La Tigra, 20 km al NE de Tegucigalpa, 1500 m, 4 Oct 1983, *Rodríguez 191* (MEXU); **Lempira**: Ciudad de Gracias, 1000 m, 7 Dec 1971, *Nelson et al. 317* (MEXU); **Ocotepeque**: Cordillera Meredón, El Portillo, 1900 m, 5 Sep 1975, *Molina R. & Molina 31107* (PMA); **Olancho**: Catacamas, Outskirts of town on E side, by stream, 11 Sep 1991, *Chorley 171* (BM).

**Jamaica**. Kingston, Park Lodge Garden, 10 Mar 1889, *Kidder* s.n. (ECON); Pons, Jan 1892, *Lloyd, R.N*., *1076* (MO); **Hanover**: Lucea, 1 Mar 1991, *Hitchcock* s.n. (MO); **Manchester**: Mandeville, Marshalls Pen, 2.25 mi due NW of Mandeville, 701 m, 3 Nov 1963, *Proctor 24153* (BM); **Middlesex**: Moneague, 21 Jan 1952, *Hunnewell 19760* (GH); **Saint Elizabeth**: Prospect, Apr 1926, *Maxwell* s.n. (BM).

**Martinique**. Ste. Pierre, 1853, *Bélanger* s.n. (G); Lavratier, 17 Apr 1934, *Rodríguez 3428* (A); Tivoli, 20 Jan 1939, *Stehlé & Stehlé 4345* (A).

**Mexico**. **Baja California**: La Paz, 20 Jan 1890, *Palmer 74* (GH, K); **Chiapas**: La Trinitaria, La Trinitaria, 1307 m, 8 Aug 1998, *Martínez S. et al. 31083* (MEXU); Acacoyagua, 29 Feb 1948, *Matuda 17531* (MEXU); **Durango**: Pueblo Nuevo, El Zapote, alrededores, 800 m, 12 Oct 1981, *Fernández N. 1921* (MEXU); **Guanajuato**: Arroyo Xichú, cercano al poblado de Xichú, 1380 m, 30 Sep 1987, *Santillán 400* (MEXU); Xichú, Las Adjuntas, 1000 m, 18 Oct 1990, *Ventura & López 8994* (F, MEXU); Xichú, El Tanque, 1300 m, 4 Dec 1991, *Ventura & López 9899* (MEXU); **Guerrero**: Eduardo Neri, Amatitlán, 1520 m, 25 Oct 1994, *Cruz Durán 94* (MEXU); Azueta, Pas de Vallecitos, 2 km al NO de Vallecito de Zaragoza, 530 m, 11 Dec 1985, *Soto N. 11628* (MEXU); Guayameo, Las Guacamayas, 19 km al SE de Guaymeo, camino a Placeres de Oro, 1150 m, 7 Sep 1982, *Soto N. & Silva R. 4426* (MEXU); Xalitla, 26 Mar 1995, *Villa & Hernández 662* (MEXU); Malinaltepec, Malinaltepec, 1600 m, 4 Jun 1989, *Wagenbreth 42* (MEXU); **Jalisco**: Tlajomulco de Zúñiga, camino a Arroyo Hondo, 200 m despues del Aguaje, Cajtitlán, 1555 m, 28 Sep 1997, *Cortés R. & Ortiz C. 95* (MEXU); Talpa, ca. 2 miles N on road to Allende, 1200 m, 14 Sep 1960, *McVaugh 20200* (G); Guadalajara, 12 Jun 1900, *Puga* s.n. (MEXU); Tuito, brecha 3 km al N del Tuito, 980 m, 26 Dec 1976, *Puga et al. 9631* (MEXU); **Michoacán**: Coalcomán, Zarzamora, 1200 m, 26 Sep 1933, *Hinton 12255* (K); **Morelos**: Puente de Ixtla, 3 km al SO de la desv. al Puente de Ixtla, sobre la carr. federal a Cuernavaca-Amacuzac, 3 Nov 1987, *Cabrera & Cabrera 14670* (CICY); Puente de Ixtla, 3 km al SO de la desv. al Puente de Ixtla, sobre la carr. federal a Cuernavaca-Amacuzac, 3 Nov 1987, *Cabrera et al. 14670* (MEXU); **Nayarit**: Islas Marías, Isla Maria Madre, brecha Rehilete-V. Carranza, 4 Dec 1986, *Chiang & Flores F. 1202* (MEXU); Tepic, 4.3 km al E del entronque a la Escondida, carr. vieja Tepic-Mazatlán, 900 m, 17 Sep 1990, *Ramírez R. & Flores C. 677* (MEXU); **Nuevo León**: W of Linares near km 31, 792 m, 20 Oct 1970, *Bates et al. 1702* (BH); **Puebla**: Coxcatlán, San José Tilapa, 700 m from San José on the Tehuacán road for Teotitló and Oaxaca, 950 m, 6 Dec 2003, *Calzada 24284* (K); Matamoros, 8 Oct 1942, *Miranda 2393* (MEXU); **Querétaro**: Jalpan, 2 km al SE de Carrizal, 1000 m, 26 Jan 1989, *Carranza 1409* (MEXU); Jalpan, San Vicente, al N, 1150 m, 1 Sep 1990, *Carranza 2689* (MEXU); Landa, Rincón de la Chirimoya, 2.5 km al SE de Acatitlán de Zaragoza, 1320 m, 26 Jun 1989, *González 698* (MEXU); **Quintana Roo**: Jiquipilas, 550 m, 8 Aug 1993, *Ferrera Sarmiento 212* (MEXU); **Sinaloa**: Sinaloa de Leyva, Los Gatos, al N de Agua Caliente, 12 Oct 1976, *Pérez 59* (MEXU); El Saucito, 52 km al E de Mocorito, brecha a Surutato, 300 m, 7 Oct 1985, *Tenorio L. 10310* (MEXU); **Sonora**: Arroyo Santa Ana, 1 km SW de Santa Ana de Yécora, 760 m, 27 May 1996, *Búrquez et al. 96-205* (MEXU); Santa Ana de Yécora, 850 m, 2 Oct 1997, *Trauba* s.n. (MEXU); **Tabasco**: Huimanguillo, Ocuapan, rumbo a Mecatepec km 9 al sur de Huimanguillo, km 2 rumbo a Fco. Rueda, 16 Jan 1979, *Cowan 1783* (MEXU); **Tamaulipas**: N of Lomas del Real, 7 miles north of main highway on dirt road which turns off just north of Altamira, 27 Oct 1959, *Johnston & Graham 4527* (MEXU); Tula, Laguna de Tula, 7 Dec 1993, *Moro-Olivo et al. 5058* (MEXU); **Veracruz**: Emiliano Zapata, carretera Zalapa-Veracruz desv. para los Baños Carrizal, 8 Oct 1975, *Calzada 2063* (MEXU); Jalapa, 6 Sep 1936, *MacDaniels 933* (BH); Comapa, 1 km al NE de El Coyol, 500 m, 30 Jun 1985, *Medina A. & Acosta P. 290* (MEXU); Xalapa, Jardín Botánico Francisco Javier Clavijero, 1300 m, 12 Oct 1979, *Ortega O. 1391* (MEXU); Dos Ríos, El Carrizal, 400 m, 11 Nov 1979, *Ventura A. 2822* (MEXU); Catemaco, Playa Hermosa, 300 m, 26 Nov 1975, *Ventura A. 12156* (MEXU); **Yucatán**: Valladolid, Xocén, 30 m, 15 Jul 1988, *Acosta 188* (CICY); Sabacché, 25 Aug 1956, *Enríquez 763* (MEXU); Peto, al 2 km al W de Peto, 9 Mar 1992, *Ku Yam 162* (MEXU); Mérida, en el poblado de Molas, 8 m, 2 Feb 1983, *Narváez 936* (CICY); Mérida, Mérida, 8 m, 30 Nov 1983, *Narváez 1186* (MEXU); Yaxcabá, Tixcacaltuyub, 24 m, 27 Jun 1980, *Vargas R. 69* (CICY).

**Montserrat**. Fox’s Bay, 19 Mar 1979, *Howard et al. 19034* (A, BM); Fox’s Bay, 1 m, 6 Feb 1959, *Proctor 19055* (A); Coconut Hill, 21 Jan 1907, *Shafer 82* (F).

**Netherlands Antilles**. **Saba**: Booby Hill, 475 m, 6 Jun 1953, *Stoffers 3161* (A).

**Nicaragua**. **Estelí**: Sábana Larga, 1060 m, 13 Apr 1981, *Moreno 8103* (MEXU); Cerro La Mocuana, faldas del cerro, al E de Trinidad, 750 m, 23 Jun 1981, *Moreno 9460* (MEXU); KM 163 on Hwy 1, 13 km N of Río Estelí, bridge on N side of Estelí, 920 m, 1 Sep 1983, *Nee & Miller 27696* (BM); km 163 on Hwy 1, ca. 11.2 km N of entrance to Estelí, 920 m, 5 Aug 1977, *Stevens 2997* (MEXU); km 163 on Hwy 1, ca. 11.2 km N of entrance to Estelí, 920 m, 29 Jun 1980, *Stevens & Montiel 17722* (MEXU); **Managua**: Esquipulas, 10 km SE of Managua, 13 Jun 1977, *Neill 2119* (MEXU); **Matagalpa**: Carretera al Tuma, 0.5 km antes de llegar a la entrada a “La Cumplida”, 600 m, 8 Sep 1980, *Guzmán et al. 741* (BM); camino a San José de los Remates, 1 km N de La Majada, ribera E de Quebrada La Majada, 400 m, 15 Jun 1980, *Moreno 797* (MEXU).

**Panama**. **Los Santos**: Los Asientos, 15 Aug 1969, *Wendehake 26* (PMA); Los Asientos, 15 Aug 1969, *Wendehake 29* (PMA); **Panamá**: campus principal de la Universidad de Panamó, alrededores de la Facultad de Agronomía, 5 Dec 1980, *Correa A. 4241* (PMA); Farfan Beach Road, 3 Aug 1967, *Kirkbride & Elías 62* (PMA); 0.6-0.9 miles S of intersection Ezra Hurwitz Road and Farfan Road, on E Hurwitz Road, Farfan Beach area and marsh, 2 Nov 1981, *Knapp 1930* (MO, PMA); **Área de Canal**: más o menos 0.5 millas de la carretera de Playa Farfan, en el camino a Palo Seco, 3 Sep 1969, *Correa A. et al. 1584* (PMA).

**Puerto Rico**. Trujillo Plant Station, 14 Jan 1936, *Britton & Britton 9867* (BH); Hato Rey, Pennock Gardens, 28 Feb 1967, *Howard 16337* (A); Hato Rey, Pennock Gardens, 28 Feb 1967, *Howard 16347* (A); Mayaguez, 1916, *Hunn* s.n. (BH); Vega Alta, Road 675, 2 Feb 1982, *Santiago-Blay* s.n. (MO); Saban Grande, 22 Mar 1935, *Sargent 218* (MO); Uluado, ad Caguana, 23 Feb 1887, *Sintenis 6343* (BM, F, G, L, MO).

**Saint Lucia**. Marquis estate, 3 m, 4 Apr 1958, *Proctor 18152* (A, BM).

**Saint Vincent/Grenadines**. **Saint Vincent**: sin. loc., 2 Jan 1823, *Caley* s.n. (BM).

**Trinidad and Tobago**. **Tobago**: Scarborough, The Chateau, 18 Sep 1913, *Broadway* s.n. (G); Old Grange, 28 Aug 1913, *Broadway 4693* (BM, GH); Scarborough, 10 Jan 1953, *Hunnewell 19989* (GH); Friendsfield road, 9 Oct 1937, *Sandwith 1674* (K); **Trinidad**: San Fernando Hill, 152 m, 1 Feb 1953, *Baker & Simmonds 14848* (K); Woodbrook, 3 Sep 1932, *Broadway* s.n. (A, B); San Fernando, San Fernando Hill, 18 Jul 1926, *Broadway 6369* (BM, K); Chancellor Road, Jan 1922, *Corstophine & Corstophine* s.n. (BM); Chacachacare, SE ridge of island, 9 Feb 1950, *Howard 10436* (A); SW Reserve, 4 Nov 1949, *Simmonds 14479* (K).

**United States of America**. **Florida**: Palm Beach County, Drehev Park, 25 Oct 1966, *Cassen 38* (BM); Broward County, Fort Lauderdale, 1 Jan 1964, *Meriläinen & Roe 126* (LE); **Hawaii**: Vaipio, 198 m, 9 Nov 1967, *Herbst 700* (L); Kauai, Haiku, where Aakulkui Road crosses Puhi Stream, Lihue District, 85 m, 17 Nov 1975, *Herbst & Spence 5558* (L); Hawaii, Hilo, 4 Aug 1981, *Schwabe* s.n. (B).

**Venezuela**. **Aragua**: San Juan de los Morros, Near Morro, 400 m, 3 Jan 1939, *Alston 6015* (BM); Maracaibo, 1893, *Mocquerys 792* (US); Parque Nacional Guamitas, 760 m, Jul 1940, *Williams 13493* (F); Guamitas, cerca del Parque Nacional, Jul 1940, *Williams 13493* (US); **Bolívar**: Cuidad Bolivar and vicinity, on the Orinoco, 60 m, 8 Mar 1921, *Bailey & Bailey 1897* (BH); Hato de Nuria, E. of Miamo, Altiplanicie de Nuria; north of road, 400 m, 1 Jan 1961, *Steyermark 88841* (US); **Carabobo**: Punta Palmita, 400 m, 20 Jul 1968, *Benítez de Rojas 366* (BM); alrededores hacienda Macapo, camino de penetracion hacia lo Guacamayo, 600 m, 19 Jul 1969, *Benítez de Rojas 708* (BM); Las Trincheras, 16 Dec 1891, *Warming* s.n. (F); **Distrito Federal**: Las Adjuntas, Dec 1924, *Allart 501* (US); Caracas, *Cochburn*, *s.n.* (BM); Caracas, 1917, *Curran & Haman 1077* (US); Cumbre del Silencio, 17 Jun 1891, *Eggers, H.F.A*., *13126* (US); Caracas, 914 m, 12 Feb 1854, *Fendler 976* (GOET); Caracas, Urb. La Florida, 950 m, 2 Aug 1979, *Nee 17113* (F); Caracas, 800 m, Oct 1921, *Pittier 9871* (US); **Lara**: Duaca, 1893, *Mocquerys* s.n. (US); **Miranda**: Río Chico, 1 Jun 1923, *Jahn 1266* (US); Santa Lucía, 150 m, 6 Mar 1943, *Killip & Smith 37028* (US); 7 km E of Cúpira, new road 1 km S of main road, starting at Río Chupaquire S of El Guacuco, 16 May 1981, *Leisner & González 11909* (F x2); Carenere, 4-5 kms N between Carenere and Chirimena, 10 m, 22 Nov 1969, *Steyermark &Bunting 102316* (US); **Mérida**: Aricagua, vicinity of Cristobal Colon, 5 Jan 1923, *Broadway 492* (US); **Nueva Esparta**: Island of Margarita, El Valle, 18 Jul 1901, *Miller & Johnston . 104* (BM, F, GH, US); **Sucre**: Cumaná, 304 m, Jul 1842, *Funck & Schlim 46* (LE); Sucre, Parque National Mochima, carretera cerca del pueblo de Mochima, 8 Sep 1982, *Garafolo 981* (US); Cocollar, 820 m, 28 Apr 1945, *Steyermark 62349* (F); alrededores de Cumana, 3 Apr 1969, *Trujillo 9374* (BM); **Trujillo**: lower Cotiza, near Caracas, 800 m, 19 May 1917, *Pittier 7169* (US); Cotiza, 22 Mar 1958, *Williams 9958* (F); **Zulia**: Mara, 3 km by air WSW of Corpozulia Campamento Carichuano, 140 m, 30 May 1980, *Steyermark et al. 122933* (F, US);

**Virgin Islands (US)**. **St. Croix**: Scenic Road near Bodkin Mill, 15 Jan 1979, *Fosberg & Hayes 58962* (BM); **St. Thomas**: sin. loc., 1827, *Wydler 67* (G).

##### Specimens examined

**[introduced, cultivated or naturalised]. Australia**. **New South Wales**: Moore Park, Richmond River, 15 miles NW of Kyogle, 27 May 1964, *Constable 4903* (LE); **Queensland**: Far North Queensland, Mount Whitfield, Mt. Whitfield, blue arrow trail. Gap at trailside, 130 m, 19 Jul 2011, *Bohs & Stern 3886* (BRI, CNS, UT); near Lake Euramoo on the road around Lake Tinaroo, 5 km W of Mobo Crater, 16 Aug 1990, *Luckow 3757* (BH); Rockhampton, Jim Crow Mountain, 5 Apr 1963, *McKee 10261* (L); Atherton, SFR 191, 7 miles S of Atherton, 600 m, 26 Feb 1965, *Rudder 3608* (L); Atherton, vicinity, 17 May 1967, *Symon, D.E*., *4754* (B); Mount Surprise, 6 miles E, 26 May 1967, *Symon 4900* (B); Ymbil, 4 Dec 1958, *Walter & Walter 2659* (B).

**Burma**. Yaunghue, southern Shan states, 1000 m, 30 Aug 1934, *Malaise 534* (S).

**Central African Republic**. region de Mbaiki, Station Central de Boukoko (Oubangi-Chari, A.E.F.), 18 Apr 1951, *Tisserant 2083* (BM).

**China**. **Hong Kong**: Chinese University of Hong Kong, University nursery, 8 Feb 2001, *Hu & Yung 317* (A, NY); Chung Chi College, garden of Glora Barretto, 7 Jun 1970, *Hu 10366* (A).

**Congo DR**. Matadi, Kongo da Lemba, 21 Mar 1956, *Dubois 109* (K); Equateur**,** Sala, 1906, *Laurent* s.n. (K); Barumbu, 6 Jan 1927, *Linder 1880* (K); Shaba, Kumcunie, Feb 1975, *Malaisse 8837* (K).

**Cyprus**. Kyrenia, 5 Jul 1955, *Atherton 86* (K).

**India**. Chota Nagpur, Hazaribagh in hedge in St. Columba’s College compound, 609 m, 24 Oct 1953, *Kerr 2343* (BM); Panchagani, Satara District, 4 Nov 1962, *Krishnappa 368* (K); Horsely Hills, Andhrapradesh, Chittor Dist, 23 Sep 1977, *Maesen 2794* (K); Dehra Dun, Mount Abu, 1219 m, 28 Sep 1958, *Raizada 20269* (K); Chandbagh, Dehra Dun and vicinity, 25 Oct 1928, *Singh 413* (F, S); **Mysore**: Hassan, Hassan District, 20 Sep 1971, *Gandhi HFP-2060* (K); Hassan, Hassan, 20 Sep 1971, *Gandhi 2060* (US); Hassan, Kesagodu-Vatehalla road, 15 May 1969, *Saldanha 13521* (US); Hassan, Hassan, outskirts of town, 23 Aug 1969, *Saldanha 14649* (US); **Palacode**: Dharmapuri, Kodagarai village, 22 Jul 1979, *Matthew & Venugopal 23911* (A); **Tamil Nadu**: Yercad, Salem, Servarayans, Yercad Ghat road, near 18th bend, 1200 m, 13 Dec 1977, *Arockiasamy 10038* (L); Dharmapuri, Dist. Denikanikottataluk, Denkanikotta, Forest Rest House, 675 m, 30 Oct 1981, *Matthew et al. RHT-28530* (K); Dindigul, Dist. Kodaikanal, Palni (Pulney) Hills (formerly Madras State), 700 m, 6 Mar 1987, *Matthew & Rajendren RHT-48498* (K).

**Indonesia**. **Java**: sin. loc, 10 Apr 1897, *Moller* s.n. (S); **Timor**: Lahurus, 600 m, 20 Jun 1968, *Schmutz 2221* (L).

**Japan**. **Okinawa**: Shioya, 10 m, 29 Jun 1993, *Leu & Wang 1961* (A); Nakagami, Ishikawa, Ishikawa Museum Grounds, 3 Jul 1951, *Walker & Tawada 5993* (US); Tanujiya, Shana Wan, 20 Jun 1966, *Walker 8192* (US); **Tokunoshima**: Hetono, Ooshima-gun, Amagi-cho, 24 Nov 1994, *Kobayashi 2841* (A).

**Kenya. Central**: Embu, Embu town, police station compound, 1381 m, 17 Apr 2010, *Vorontsova et al. 193* (BM, EA, K, NY); **Kiambu**: Kabete, FTEA region K4, Ngecha road, about 1km past Kitisuru road intersection, 1700 m, 13 Nov 1977, *Kuchar 7797* (EA); **Nairobi**: Nairobi National Museum Botanic Garden, FTEA region K4, Jan 1949, *Bally 6565* (EA, K); Kirichwa Ndogo, FTEA region K4, 1660 m, 10 Apr 1969, *Bally 13282* (EA); Nairobi, FTEA region K4, Forest road, 8 Jun 1972, *Kibuwa 1200* (EA); Chiromo, FTEA region K4, Chiromo Estate, 30 Apr 1970, *Mathenge 601* (EA); Chiromo, FTEA region K4, University of Nairobi, Chiromo Campus, behind the Entomology Department, 1676 m, 26 May 1983, *Mwangangi 2474* (EA); Nairobi, FTEA region K4, Ring River road, 1740 m, 24 Dec 1972, *Sangai 1* (EA); **Nakuru**: Njoro, FTEA region K3, Egerton University, 2256 m, 25 Jun 1976, *Gitonga 284* (EA); **Taita Taveta**: Bura, FTEA region K7, along Bura-Mugange road, 23 Jul 1972, *Cheseny 30-72* (EA); Bura, FTEA region K7, west of Buar River, 4 km south of St. Mary’s Teachers Training College, 970 m, 21 Nov 1997, *Mwachala EW-31* (EA); Taita Hills, FTEA region K7, 850 m, 31 Jul 1998, *Mwachala 1203* (EA, K).

**Lebanon**. Beirut, 12 Jan 1951, *Anonymous* s.n. (G).

**Madagascar**. **Toliara**: Taolagnaro, (Fort Dauphim), region of Manambaro, near Tsiary, 34 m, 9 Apr 2007, *Wen & Randriatafika 9666* (US).

**Malawi**. Dist. Zomba, Mingoli Farm, by Likangala River, 13 Jan 1979, *Blackmore & Dudley 151* (BM).

**Namibia**. Gross Waterburg, near Waterberg in Waterberg Mountains, 11 Nov 1947, *Rodin 2585* (BH, F); Kunene**,** Outjo, Farm Franken, 26 Jan 1953, *Schwerdtfeger* s.n. (B).

**New Caledonia**. Ouen Toro, 15 Jan 1955, *McKee 2006* (L); **Grande Terre**: Mount Mou, above Col de la Pirogue, on southwest side, 400 m, 2 Aug 1981, *Edmondson 3635* (L).

**Papua New Guinea**. **Morobe**: Wau, Ian Fraser’s plantation, 360 m, 13 Jan 1981, *Kerenga & Kerenga 77239* (L); Wau, route de Bulolo, 17 Apr 1987, *Lambinon 87/383* (L).

**Paraguay**. **Alto Paraná**: Distrito Ciudad del Este, Cuidad del Este, Vivero Forestal de Itaipu, 12 Oct 1990, *Schinini & Cabellero Morini 27250* (G); **Central**: Asunción, 28 Jul 1972, *Schinini 5069* (G); **Central-Cordillera**: Lago Ypacaraí, Jardin Steiner, Mar 1913, *Hassler 11990* (E, F, G x2, K, L, P); **Cordillera**: San Bernardino, camino a Altos, 5 Dec 1987, *Schinini & Bordas 25512* (G).

**Peru**. **Lima**: Lima, Miraflores, Lima, 11 Feb 1987, *Dreyfus R. 1* (USM); Lima, Lima, esquina Larrabura y Máximo Abril, Jesús Maria, 25 Nov 1956, *Tillett 661 -41* (USM); Lima, Lince, cuadra 10 de la Av. Arenales, 14 May 1980, *Vilcapoma 337* (USM); **Loreto**: Alto Amazonas, Yurimaguas, Fortaleza, lower Río Huallaga, 155 m, 29 Oct 1929, *Williams 4323* (F); Alto Amazonas, Yurimaguas, on lower Río Huallaga, 155 m, 2 Apr 1930, *Williams 7877* (F); **Tumbes**: Tumbes, mtns. E of Hacienda Chicama, 800 m, 19 Feb 1927, *Weberbauer 7636* (F).

**Philippines**. Puerto Galera, Oriental Mindoro Province, Apr 1953, *Alegaen 206* (L).

**South Africa**. between Grootfontein and Otavi, 1400 m, 2 Mar 1959, *Werdermann & Oberdieck 2391* (B); **Transvaal**: Lowveld Botanic Garden, N of Nelspruit, Crocodile River valley, 650 m, 23 Sep 1989, *Greuter 21464* (B); Letaba, by side of road to Politsi just-SW of Eucalyptus plantations near Merensky Dam, road and railway bridges, 914 m, 18 Apr 1958, *Scheepers 265* (EA).

**Sri Lanka**. Pottuvil, 14 Dec 1975, *Bernardi 16014* (US); Matale-Dambulla road, Before mile marker 44 (A9), 215 m, 12 Jan 1968, *Comanor 718* (GH, K, US); Yala, behind Yala campsite, 23 Jan 1968, *Comanor 831* (K, US); Anuradhapura-Puttalam Road, 29.5 mile post, 3 Feb 1970, *Cooray 20308-R* (K, US); Menik Ganga, on east bank, 100 meters from Yala Bungalow, west of camp site at bathing spot, 20 Oct 1967, *Cooray 102001 R* (US); Polonarruwa, at rest house, 8 Jan 1970, *Fosberg 51912* (US); Siyambalanduwa, 4 miles N, 27 Nov 1970, *Fosberg & Sachet 53074* (US); Amparai, 3 Dec 1977, *Fosberg & Jayasinghe 57171* (K, US); Anuradhapura, Kekirawa, 18 Dec 1973, *Jayasuriya et al. 1385* (K, US); Kandy, Haragama, 500 m, 19 Jan 1974, *Jayasuriya et al. 1409* (K, US); Matara, Naula, 23 Jan 1974, *Jayasuriya et al. 1426* (K, US); Wellawaya road, 21 Aug 1974, *Kostermans 25435* (G); Menik Ganga, north of Kataragama, 1 mile south of Vaddangewadiya, 23 Oct 1968, *Mueller-Dombois 68 102303* (US); Ruhuna National Park, Block I, at Yala campsite, 10 Dec 1967, *Mueller-Dombois & Cooray 121087* (US); Puttlam-Anuradhapura road, 7 Feb 1932, *Simpson 9162* (BM); **Central**: Matale, Dambulla, 280 m, 9 Dec 1971, *Cramer 3550* (US); Matale, Naula, just north, mile 35 on rt. A9, 22 Oct 1976, *Fosberg 56368* (K, US); **Monaragala**: 7 miles N of Bible, 30 Apr 1976, *Jayasuriya 1936* (K, L); **North Western**: Kurunagala, Weundakanda, 12 Aug 1974, *Waas 770* (US); **North-Central**: Polannaruwa, 60 m, 26 Feb 1972, *Cramer 3681* (US); Northgate, Polonnaruwa Sacred Area, 61 m, 27 Aug 1971, *Ripley 458* (US).

**Swaziland**. Ngwavuma, 274 m, 1 Jul 1966, *Bayliss 3447* (BH).

**Taiwan**. Hengchung, Hang Chun Botanical Garden, 26 Dec 1979, *Bernardi 19874* (G); Taihoku, Jardin Botanique, Apr 1915, *Faurie* s.n. (A).

**Tanzania**. Bushiri Estate, Pangani District, 16 Jun 1950, *Faulkner 656* (K); Ngare Nairobi, Moshi district, Northern Prov, 10 May 1949, *Rensberg 495* (K); Morogoro, Uluguru Gebirge, S seite, Kissaki, 200 m, 15 Aug 1933, *Schlieben 4246* (BM); **Handeni**: Kideleko, FTEA region T3, 7 km south of Handeni, bottom of Mt. King’ombe, 610 m, 9 Jul 1974, *Archbold 1871* (EA); **Lushoto**: Amani, FTEA region T3, 20 Mar 1906, *Braun 1122* (EA); Mombo Forest Reserve, FTEA region T3, Tanga region, 22 Jun 1962, *Semsei & Semsei 3487* (EA); **Morogoro**: Morogoro, FTEA region T6, 548 m, 19 Jul 1969, *Harris 2965* (EA); **Moshi**: Kilimanjaro, FTEA region T2, Sere River, Two Bridges, 1006 m, 4 Jun 1967, *Bigger 1100* (EA); Ngare Nairobi, FTEA region T2, Stock Farm, near homestead, 10 May 1949, *Rensburg 495* (EA); **Tanga**: Mzizima, Dar-es-Salaam, Statehouse, 29 Sep 1972, *Ruffo 547* (K); Lushoto, Soni, 1252 m, 20 Aug 2010, *Anna NPGRC 396* (K); Mafi Hill, E slope above Magamba Sisal Estate, SW of West Usambara Mtns., Lushoto District, 750 m, 27 Jan 1985, *Borhidi et al. 85359* (K); Muheza, W of Kwamngumi Forest Reserve, 125 m, 25 May 2000, *Mwangoka & Maingo 1299* (MO); Muheza, Kwemnyese public forest patch, E of forest, 100 m, 30 May 2000, *Mwangoka & Mkufya 1347* (MO); Mambo Forest Reserve, Tanga region, Kosogwe District, 22 Jun 1962, *Semsei 3487* (K); **Uzaramo**: Dar es Salaam, FTEA region T6, State House, 29 Sep 1972, *Ruffo 547* (EA).

**Thailand**. Suen Dawk, Chiang Mai, 22 Dec 1950, *Garrett 1342* (L); **Chiang Mai**: Chiang Mai, 3 Dec 1914, *Kerr* s.n. (BM); Chiang Mai, 20 Jan 1921, *Kerr* s.n. (BM).

**Uganda**. **Bunyoro**: Budongo, FTEA region U2, near main Butiaba road at North end of Busingiro Hill, 1100 m, 27 Jun 1973, *Synnott 1485* (EA); **Karamoja**: Kidepo Valley National Park, FTEA region U1, Opok Rest Camp, 4 Dec 1971, *Katende K 1379* (EA); Kidepo National Park, FTEA region U1, Apoka Park HQ, Dodoth, 1170 m, 4 Sep 1972, *Synnott 1286* (EA); Kidepo National Park, FTEA region U1, Dodoth, 1170 m, 12 Aug 1973, *Synnott 1545* (EA); **Toro**: Queen Elizabeth National Park, FTEA region U2, Mweya, 945 m, 30 May 1969, *Lock 69-139* (EA); **Western**: (U2), Masindi District, W boundary of Budongo Forest Reserve, on E flank of Busingiro Hill, ca. 2 km N of ecotourism camp on road to Butyaba Pier on Lake Albert, 1080 m, 3 Jul 1998, *ATBP 786* (MO).

**Vanuatu.** Melekula Island, S.W. Bay, Wintoua and vicinity, 100 m, 8 Oct 1963, *Chew Wee-Lek 389* (L); Efate, près de la cascade de Maat, 11 Jul 1971, *Raynal 16057* (L).

**Zambia**. Victoria Falls, Devil’s Cataract, Nov 1959, *Armitage 154 /59* (L); Penhalonga, 1219 m, 6 Feb 1934, *Davies* s.n. (BM); Victoria Falls, Feb 1959, *Head* s.n. (BM); Victoria Falls, 903 m, 1 Apr 1974, *Gonde 88/74* (B).

**Zimbabwe**. Masvingo, 16 Jul 1989, *Emanuelsson 460* (S); Matopos, 1402 m, Mar 1918, *Eyles 981* (BM); N’Dola, 3 Jun 1954, *Fanshawe 1262* (S); Harare, north of Lake Robertson on A-road, 31 Jan 1990, *Emanuelsson 860* (S).

#### 36. Solanum septemlobum

Bunge, Enum. Pl. Chin. Bor. 48. 1833

http://species-id.net/wiki/Solanum_septemlobum

Figure 89

Solanum quercifolium L., Sp. Pl. 185. 1753. nom. rej. Type: Sweden: Cultivated in Uppsala, said to be from Peru, *Anon*. (lectotype, designated by Knapp and Jarvis 1990, pg. 355: LINN [LINN 248.8]).
Solanum septemlobum Bunge var. *ovoideocarpum* C.Y.Wu & S.C.Huang, Acta Phytotax. Sin. 16(2): 72. 1978. Type: China. Hebei: Beijing, *Shen-E Liu 2087a* (holotype: PE [PE00031394]).
Solanum septemlobum Bunge var. *subintegrifolium* C.Y.Wu & S.C.Huang, Acta Phytotax. Sin. 16(2): 73. 1978. Type: China. Gansu: Xifeng, *Zuobin Wang 17568* (holotype: PE [PE00031393]).

##### Type.

China. “China borealis”, Jun-Jul 1831, *A.A. Bunge* s.n. (lectotype, designated here: P [P00055357]; isolectotypes: G [G00357887], P [P00055358]).

##### Description.

Woody vine or lax shrub, to several meters long. Stems slightly winged, sparsely to densely pubescent with white, curved, simple uniseriate trichomes to 0.5 mm long, these appressed and all pointing apically, with 4–6 small cells; new growth densely white pubescent with simple uniseriate curved trichomes to 0.5 mm long. Bark of older stems pale brown, glabrescent. Sympodial units plurifoliate. Leaves simple or more commonly variously pinnatifid and lobed, extremely variable in shape and size, (1-)2–9(-10) cm long, (0.9-)1.2–5 (-8) cm wide, ovate in outline, widest in the basal third of the blade, the upper and lower surfaces sparsely to moderately pubescent with simple uniseriate trichomes to 1 mm long like those of the stems, all pointing to the margins (away from the axis); base truncate, then attenuate onto the petiole; margins entire to deeply lobed with 1–4 pairs of lobes to within 1 mm of the midrib, the lobbing irregular towards the leaf apex; apex acute, rounded or if acuminate, the ultimate tip rounded; petiole 0.5–3 cm long, sparsely to densely pubescent like the stems. Inflorescences terminal or lateral, 2.5–16 cm long, open and many times branched, with 10–40 flowers, sparsely pubescent with simple white trichomes like those of the stems, these curved and pointing towards the tip of the inflorescence; peduncle 1–5 cm long; pedicels 7–10 mm long, ca. 0.5 mm in diameter at the base, ca. 1 mm in diameter at the apex, slender, spreading, glabrous, articulated at the base, leaving a small peg to 1 mm long on the inflorescence axis; pedicel scars irregularly spaced 1–10 m apart, closer towards the distal part of the inflorescence. Buds ellipsoid, the corolla strongly exserted from the calyx tube before anthesis. Flowers all perfect, 5-merous. Calyx tube 1–1.5 mm long, conical, the lobes 0.5–1.5 mm long, broadly deltate, the margins usually thickened and white in dry material, glabrous or with a few white, uniseriate curved trichomes. Corolla ca. 2 cm in diameter, violet with a green eye, this whitish green or brown in dry material, stellate to broadly stellate, lobed 1/2 to 2/3 of the way to the base, the lobes 4–6 mm long, 1.5–4 mm wide, reflexed to spreading at anthesis, glabrous, minutely papillate on the tips. Filament tube ca. 0.5 mm long, the free portion of the filaments ca. 1 mm long, glabrous or pubescent with a few weak uniseriate simple trichomes in the sinuses; anthers 3–4 mm long, ca. 1 mm wide, loosely connivent, ellipsoid, poricidal at the tips, the pores lengthening to slits with age. Ovary glabrous; style 4.5–7.5 mm long, glabrous; stigma capitate, minutely papillate, green or white in living material. Fruit a globose or slightly ovate berry, 0.8–1 cm wide, 1–1.2 cm long, bright red when ripe, the pericarp thin and shiny, glabrous; fruiting pedicels 0.8–1.3 cm long, ca. 1 mm in diameter, not particularly woody, spreading. Seeds > 20 per berry, ca. 3 mm long, ca. 2 mm wide, flattened reniform, pale yellowish tan or yellow, the surfaces minutely pitted, the mature seeds appearing pubescent with the elongate lateral cell walls ca. 0.2 mm long, the pitted bases still visible. Chromosome number: not known.

**Figure 89.**
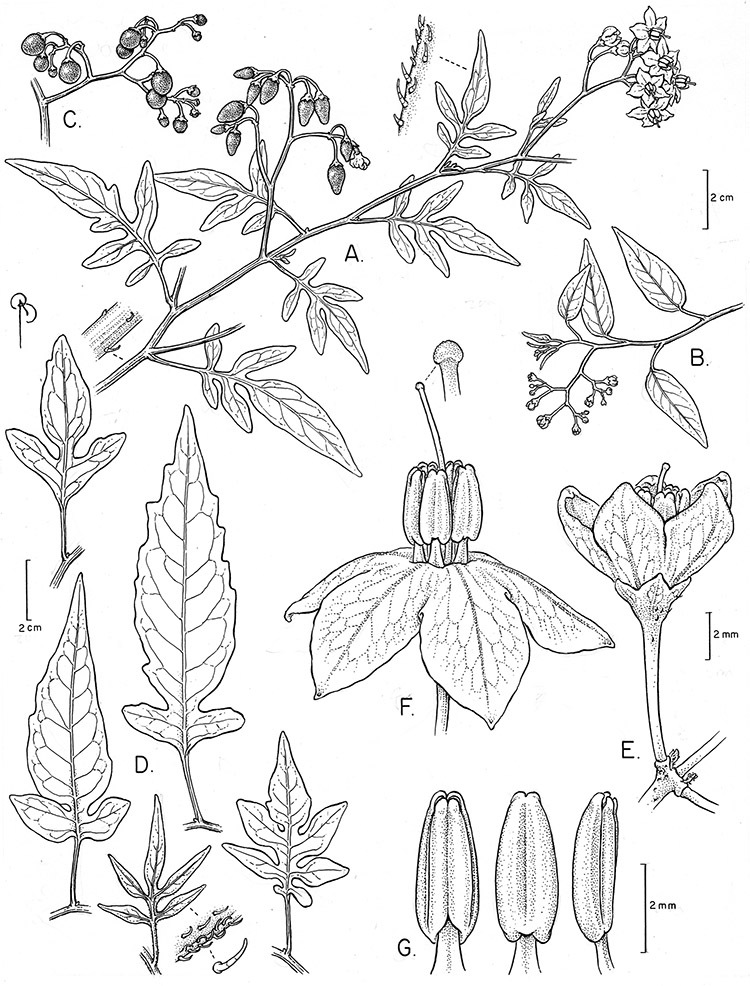
*Solanum septumlobum* Bunge. (**A, E–G** drawn from *Smith 7514* **B** drawn from *Smith 6456* **C** drawn from *Shanxi Team 1627* **D** [leaf variation] from *Li 930033b* in addition to those cited). Illustration by Bobbi Angell.

##### Distribution

**Figure 90.**
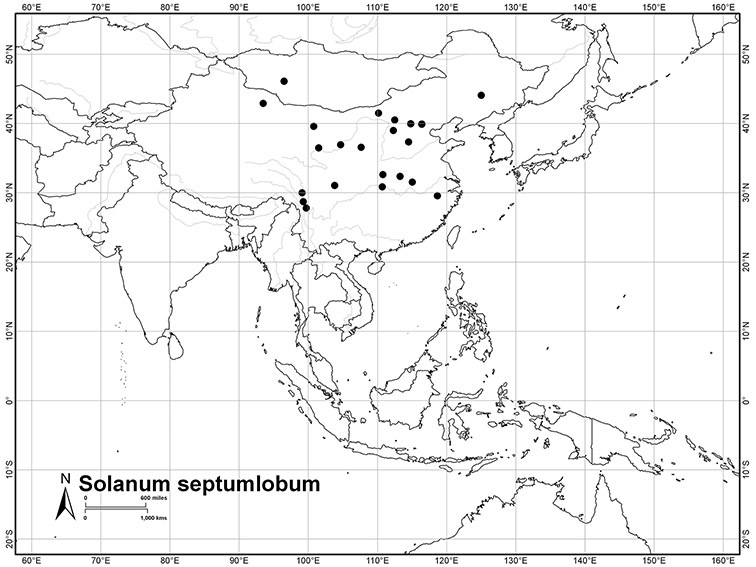
Distribution of *Solanum septumlobum* Bunge.

(Figure 90). *Solanum septemlobum* is a boreal species in China, from sea level to 1200 m; it perhaps extends to adjacent Mongolia but all specimens seen so far are from the Chinese Autonomous Region of Nei Mongol, previously known as “Inner Mongolia”. Its southerly distribution overlaps with *Solanum pittosporifolium*, from which it is sometimes difficult to distinguish.

##### Ecology.

Growing in open areas and secondary forests.

##### Common names.

China: qing qie (Zhang et al. 1994).

##### Conservation status.

Least Concern (LC); EOO >100,000 km^2^ (LC) and AOO >10,000 km^2^ (LC). See Moat (2007) for explanation of measurements.

##### Discussion.

*Solanum septemlobum* usually has seven-parted leaves (as the name implies), but as is common in this clade, leaf division varies from simple to seven-parted. It is not clear from herbarium specimens if these differences in leaf division have an environmental basis, but from field observations on the related *Solanum dulcamara*, their regulation is likely to be complex. *Solanum septemlobum* can be difficult to distinguish from its sympatric close relatives, *Solanum pittosporifolium* and *Solanum lyratum*, but the flowers are in general larger, and pubescence of stems and leaves is of stiff, curving white trichomes, not long glandular trichomes like those of *Solanum lyratum*. *Solanum pittosporifolium* occasionally has a few white trichomes on new stems and leaves, but they are usually very sparse and not as stiff, long or curved as those of *Solanum septemlobum*.

*Solanum septemlobum* is a more northerly species than either *Solanum lyratum* or *Solanum pittosporifolium*, and almost abuts the range of *Solanum dulcamara* in Mongolia and northern Russia.

Bunge’s original herbarium and types are said to be either at LE or P (ex. herb. Cosson); the sheet in P clearly annotated in Bunge’s hand as “*Solanum septemlobum* mihi” from Bunge’s personal herbarium is here chosen as the lectotype. No duplicates of this sheet have been located in LE, so this P sheet is the logical lectotype. The additional sheet of this collection in P [P00055358] is also apparently from Bunge’s personal herbarium, and is annotated by Bunge, but appears to be a duplicate (it is a much smaller specimen and appears to have been broken off from the other sheet) given to Decaisne. The original publication of the name *Solanum septemlobum* is in 1833 (not in 1835 as stated in some indices), in a preprint from *Mém. Acad. Imp. Sci. St.-Pétersbourg Divers Savans* 2:75-148 of 1835.

The specimen cited by Linnaeus in describing *Solanum quercifolium* was used by Knapp and Jarvis (1990) to lectotypify this name; they assumed it was a New World plant following Linneaus’ distribution and the annotation on the sheet. The late Bill D’Arcy later recognised that the specimen in question (LINN 248.8) was actually that commonly known as *Solanum septemlobum*, and the name *Solanum quercifolium* was rejected (Turland et al. 1996). I have seen many sheets of *Solanum septemlobum* labelled as *Solanum quercifolium* in European herbaria from the late 18^th^ and early 19^th^ centuries, this species was clearly in cultivation in botanic gardens at that time, but was later lost in cultivation; there are almost no specimens dating from the mid-19^th^ century to present from European botanic garden collections nor have I seen the species in cultivation in Europe.

##### Specimens examined.

**China**. **Anhui**: Sanyang, She county, 400 m, 2 Jul 1995, *Dong 0621* (MO); **Beijing**: Beijing, Sep 1917, *Andersson 56* (S); mountain west of Peking, 1881, *Bretschneider 1927* (BM); near Peking, province of Shan-Teng, May 1907, *Meyer 392* (GH, US); on city wall, (Peking) (Chi-li), 5 Jul 1913, *Meyer 1009* (GH, K, US); [Beijing], Aug 1863, *Williams 1341* (BM); **Gansu**: Lanzhou City, Xianding, W of Tianmu Mt, 28 Sep 1963, *Hangzhou Bot. Gard. Herb. 29999* (MO); Wenshien, 910 m, 15 Jun 1930, *Hao, K.S*., *418* (S); Minchow, 26 Jun 1930, *Hao 531* (S); Lanzhou City, Baita Mt., 1 Jun 1993, *Li 93-0033 b* (MO); Kansou [Gansu], N.E, 18 Jun 1920, *Licent 6045* (K); **Guangxi**: Xing’an, Maoer Mt., Xingan, 1200 m, 26 Jul 1997, *Li 15191* (MO); **Hebei**: Hsin-Chi, Sulu Hsien, Hsin Chi, 20 Jun 1948, *Beach 27* (G, K, MO, US); Xiaowutai Mountain, 1935, *Liu 10798* (MO); Xiaowutai Mountain, 13 Aug 1935, *Liu 10889* (MO); Xiaowutai Mountain, 18 May 1935, *Liu 11112* (MO); Linchengfeng village, SW Hebei province, 200 m, 14 Jun 1950, *Liu 12777* (MO); on the road from Linchengfeng village to Neiqiu, SE Hebei province, 200 m, 16 Jun 1950, *Liu 12800* (MO); Xiaolingdi village, Neiqiu County, 1200 m, 8 Aug 1950, *Liu 13286* (MO); **Henan**: Sin. loc., 360 m, 1958, *Anonymous 346* (HIB); Teng-feng, Saho lin ssu im Kreise Teng fong, 670 m, Aug 1907, *Schindler 168* (G, K, L, S); Teng-feng, Shao lin ssu im Kreise Teng fong, 670 m, Aug 1907, *Schindler 670* (BM); Tangbai County, Taibaiding, Tongbai Mt., 1 Aug 1985, *South Team*, *T 0265* (MO); Xin County, 600 m, 23 Aug 1989, *South Team*, *D 1046* (MO); Jigongshan, Jigong Mt, 700 m, 28 Jun 1963, *Zheng 209* (MO); **Hubei**: Xingshan, Mengyuan, 800 m, 22 Jul 1956, *Li 3* (MO); Hsiaowutai Shan, Jul 1934, *Wang 62342* (A); Shiyan, Shiyan City, 850 m, 8 Nov 1994, *Zhao 5429* (MO); **Jiangsu**: beside Dufeng Temple, Jiangpu District, Nanjing, 28 Jul 1958, *Jiangpu Team 8292* (MO); **Jilin**: Ching Lung Chiao, near Great Wall, 20 Sep 1930, *Dorsett & Morse 7235* (US); Kirin [Jilin], Kirin to Tsitsihar Manchuria, 1887, *James* s.n. (K); Kirin, Tch’ang tch’ounn, Manchuria, 4 Sep 1928, *Licent 8611* (BM, K); **Nei Mongol**: Basasekulle n. om Katolska missionem, 12 Jul 1919, *Andersson 337 a* (S); Gongruk, 27 Jul 1924, *Eriksson 6* (S); at Congrek west of Dojen [Dojen = Swedish Missionary Station in Inner Mongolia, Dongsheng?], 27 Jul 1924, *Eriksson 6* (US); Mantalte Sume, 10 li to E, 18 Jul 1935, *Eriksson 1087* (S); Beli-Miao, 10 li [measure of distance] W, 30 Jun 1936, *Eriksson 1146* (S); Mongolia, Ordos, Ikenwusu, 4 Aug 1933, *Hsia 3740* (K); Kwei Hua, outskirts, 20 Aug 1938, *Martin & Soderbom* s.n. (A); Dun-tai-pin Shan, Chahar province, 29 Jul 1934, *Kozlov 232* (US); Chahar, Mvolte ama, Darkhan Beile, 1 Jul 1935, *Roerich Expedition 325* (US); Naran Obo, east of Naran Obo, 20 Jul 1935, *Roerich Expedition 402* (US); Madenii Amon, Chahar province, 10 Aug 1935, *Roerich Expedition 766* (US); Madenii Amon, Chahar province, 10 Aug 1935, *Roerich Expedition 779* (US); Naran Obo, Inner Mongolia, Chahar province, 16 Jul 1935, *Roerich Expedition 385* (GH, US); Wanziagou, 26 Jun 1997, *Wu 97-73* (MO); Taipingzhai, Liangcheng, 25 Sep 1998, *Wu 98-164* (MO); Tuo county, 26 Jun 1997, *Wu 97-75* (MO); **Ningxia:** Ho Lan Shan mountains, the mouth of Sis Ye Ku, 1375 m, 10 May 1923, *Ching 172* (GH, US); Ho Lan Shan mountains, Mingshia, 1750 m, 20 Aug 1923, *Ching 1104* (GH, US); Ala Mountains, Ning-Hsia, 1500 m, 27 Aug 1933, *Pai 115* (K); **Qinghai**: Ta’er Temple, Huangzhong, 2800 m, 2 Sep 1980, *Wang 978* (MO); **Shaanxi**: Tungkwan, 29 Jul 1932, *Hao 3804* (K); Wen Shui, W. Lungchuan Tsun, 6 Jul 1925, *Kang 26* (GH); Sjara osso gol, 10 Aug 1922, *Licent 686* (BM, K); Taipeishan, 1910, *Purdom* s.n. (K); Hongshiya, Huanglong county, 900 m, 21 Jun 1985, *Yang 6317* (MO); **Shandong**: Meng Shan, Fei Hsien, 450 m, 24 Jul 1936, *Cheo & Yen 163* (BM, G, GH); Lung Shan, 90 li from Tsinanfu, 200 m, 18 Sep 1930, *Chiao 3129* (GH, K, US); **Shanxi**: Taiyüan-fu, northern direction 40 li from the city, 12 Jun 1919, *Andersson 627a* (S); Tsai yan sban, Makiapou, 21 Jul 1914, *Licent 359* (BM, K); Taiyüan-fu, Jun 1902, *Nyström* s.n. (S); Shuiiu, Yunsheng [label in Swedish is “Shuiio”], 1923, *Otterdahl* s.n. (S); Shueiyu, Shansi australis, 701 m, 15 Aug 1925, *Sandberg 173* (S); Yunching, Shansi australis, 213 m, 30 Aug 1925, *Sandberg 184* (S); close to Ningwu County, 14 May 1957, *Shanxi Team 1627* (MO); Taiyüan-fu, 800 m, 24 May 1928, *Smith 5580* (S); Ye-cho-shan, 1500 m, 18 Jul 1924, *Smith 6456* (MO x2); Chiao-chieng distr., Pa-shui-ko, 1800 m, 4 Sep 1924, *Smith 7514* (MO); near Sihsien, 11 Sep 1935, *Wang 3599* (K); **Sichuan**: Derong, *Anonymous* s.n. (HITBC); Batang, *Qingzhang Team 5189* (HITBC); **Tibet**: Eastern Tibet, Ba Valley [may be in Sichuan], 3018 m, Jun 1926, *Rock 14273* (GH, K, S); Ta Tsien Lou, Thibet oriental, (principaute de Kiala), 1893, *Soulié 872* (G, K); **Xinjiang:** Hami, Turkestaniae, May 1881, *Mesny 10511* (BM); **Yunnan**: Deqen, *Qingzhang Team 2586* (HITBC); **Zhejiang**: Sin. loc., *Barchet* s.n. (MO).

#### 37. Solanum sousae

S. Knapp, PLoS ONE 5(5): e10502. 2010

http://species-id.net/wiki/Solanum_sousae

Figure 91

##### Type.

Mexico. Oaxaca: Mun. San Miguel Chimalapa, Cerro La Culebra, al N del Cerro Guayabitos, ca. 6 km línea recta al NO de Benito Juarez, ca. 42 km en línea recta al N de San Pedro Tapanatepec, 16˚45'N, 94˚11'W, 1600–1800m, 16–18 Jul 1986, *Solanum Maya J. 3602* (holotype: MEXU [MEXU-932219]).

##### Description.

Woody vine with trailing stems; stems sparsely pubescent with simple, uniseriate trichomes to 0.5 mm long, composed of 2–3 cells, the stems soon glabrescent; new growth densely pubescent with simple uniseriate trichomes, these whitish cream; bark of older stems pale greenish brown, glabrescent. Sympodial units plurifoliate. Leaves simple, 2.7–7(+) cm long, 1–5 cm wide, narrowly ovate to elliptic, membranous, the upper surface glabrous to sparsely pubescent with simple, uniseriate trichomes on the lamina, more densely pubescent on the veins, the trichomes to 0.5 mm long, the undersurfaces almost glabrous to densely pubescent with simple, uniseriate trichomes to 0.5 mm long, these denser on the veins; primary veins 5–7 pairs, yellowish; base truncate to broadly acute; margins entire; apex acute to acuminate; petioles 1–4 cm long, glabrous or pubescent like the adjacent stem, twining. Inflorescence 7–10 cm long, terminal, many times branched, more or less broadly triangular in outline, with 30–40 flowers; peduncle 3–4 cm long, pubescent like the stems; pedicels 1–1.5 cm long, ca. 0.5 mm in diameter at the base, ca. 1 mm in diameter at the apex, nodding at anthesis, sparsely pubescent like the rest of the inflorescence, articulated near the base, leaving a small peg ca. 1 mm high, on the rhachis; pedicel scars spaced 0.1–0.5 cm apart, clustered near the tips of the inflorescence branches. Buds narrowly ellipsoid, the corolla strongly exserted from the calyx tube. Flowers all perfect, 5-merous. Calyx tube 1.5–2 mm long, conical, appearing striped from the thickened venation, the lobes <0.5 mm, mere undulations on the margin of the tube, occasionally somewhat quadrate when sinus splitting, sparsely and unevenly pubescent with simple, uniseriate trichomes to 0.5 mm. Corolla 1.5–2 cm in diameter, white, stellate to rotate-stellate, lobed 1/2 to 3/4 of the way to the base, the lobes 5–8 mm long, ca. 4 mm wide, planar or slightly cupped at anthesis, densely pubescent-papillate with minute simple trichomes abaxially, glabrous adaxially. Filament tube minute, free portion of the filaments unequal, with 3 filaments 1–1.5 mm long and 2 filaments 1–5-2 mm long, pubescent near the base adaxially with tangled, simple uniseriate trichomes ca. 0.5 mm; anthers 2.5–3 mm long, 1–1.5 mm wide, ellipsoid, poricidal at the tips, the pores lengthening to slits with age. Ovary glabrous; style 7–9 mm, pubescent with simple uniseriate trichomes 0.5 mm long in the lower half; stigma capitate or somewhat bilobed, the surface densely papillate. Fruit a globose berry to 1.5 cm in diameter, green (immature?), the pericarp thin, matte; fruiting pedicels 1.5–1.7 cm long, ca. 1.5 mm in diameter, woody and pendent. Seeds >50 per berry, ca. 2.5 mm long, ca. 2 mm wide, flattened reniform, golden brown, the testal surface minutely pitted. Chromosome number: not known.

**Figure 91.**
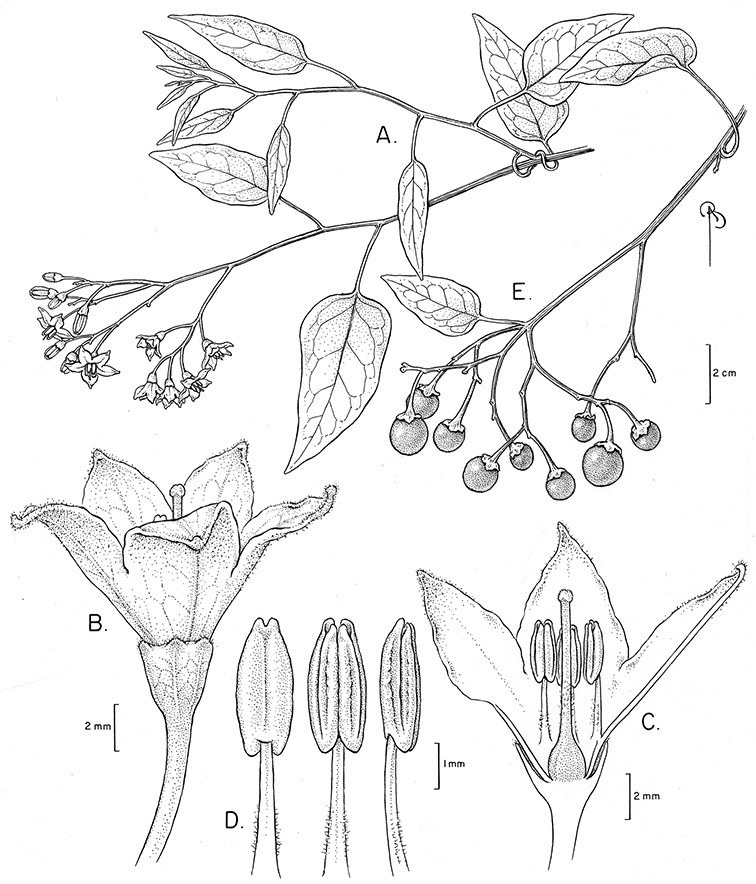
*Solanum sousae* S. Knapp. (**A–D** drawn from *Ventura 2212*, E from *Maya J. 3938*). Originally published in Knapp (2010). Illustration by Bobbi Angell.

##### Distribution

**Figure 92.**
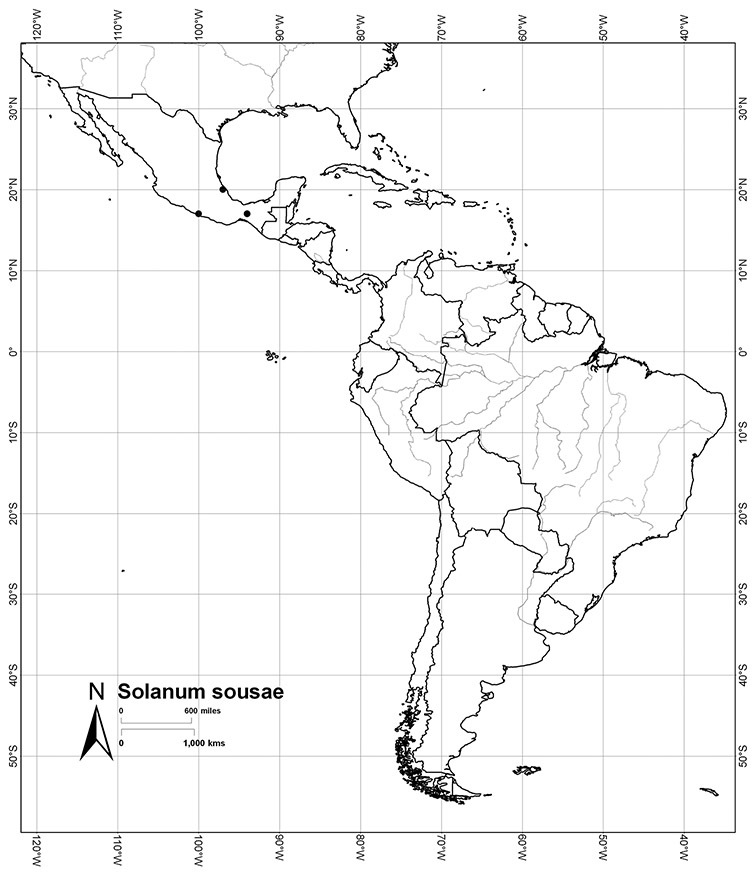
Distribution of *Solanum sousae* S.Knapp.

(Figure 92). *Solanum sousae* is known only from southern Mexico in the states of Puebla, Oaxaca and Veracruz from 1600–1900 m.

##### Ecology.

Occurs in mesophyllous forests and oak-pine-*Liquidambar* L. (Hamamelidaceae) forests on steep slopes with rich soils.

##### Conservation status.

Near Threatened (NT); EOO <100,000 km^2^ (possible NT) and AOO <10,000 km^2^ (LC), with few populations. See Moat (2007) for explanation of measurements.

##### Discussion.

*Solanum sousae* is superficially similar to *Solanum pyrifolium* of Hispaniola, but differs from that species in its more broadly triangular inflorescence outline, minute calyx lobes without thickened margins, anthers borne on unequal filaments and in its lack of a prominent submarginal leaf vein. The leaf pubescence of the two species is very similar, but *Solanum sousae* is in general more densely pubescent on the new growth and abaxial corolla surfaces. *Solanum sousae* differs from the more common and sympatric *Solanum dulcamaroides* in its white flowers, generally simple pubescence (versus more commonly dendritic in *Solanum dulcamaroides*), white rather than purple flowers, and in its anthers that are not markedly thickened and rounded abaxially.

It is likely that the juvenile leaves of *Solanum sousae* are pinnatifid, as are those of most other species in this group; young foliage is only very rarely collected and is often not associated with the flowering stems with simple leaves.

##### Specimens examined.

**Mexico**. **Oaxaca**: San Miguel Chimalapa, Cerro La Culebra, al N del Cerro Guayabitos, ca. 6 km linea recta al NO de Benito Juarez, ca. 42 km en linea recta al N de San Pedro Tapanatepec, 1600 m, 16 Jul 1986, *Maya J. 3602* (MEXU); Santa María Chimalapa, Cerro de los Pavos, al N de Cerro Guayabitos y al O del Rio Portemonedas, ca. 47 km en linea recta al N de San Pedro Tapanatepec, 22 Sep 1986, *Maya J. 3938* (MEXU); **Puebla**: Atempan, Puente Viejo, 1900 m, 8 Jul 1986, *Ventura A. 22129* (MEXU); **Veracruz**: Mun. Jalacingo, Agua Cruz, 1600 m, 5 May 1970, *Ventura A. 1022* (NY).

#### 38. Solanum stenophyllum

Dunal, Solan. Syn. 15. 1816

http://species-id.net/wiki/Solanum_stenophyllum

Figure 93

Solanum bogotense Dunal, Prodr. [A.P. de Candolle] 13(1): 121. 1852. Type: Colombia. Cundinamarca: Bogotá, 1846, *J. Goudot* s.n. (holotype: G-DC [G00145407, F neg. 6789]; isotypes: F [F-679074, frag.], P [P00324773], W [W-0003065]).
Solanum tolimense Wedd., Chlor. And. 2: 106. 1859. Type: Colombia. Tolima: páramo de Tolima, 3900 m, *J.J. Linden 957* (holotype: P [P00371702, Morton neg. 8356]; isotypes: BM [BM000815927], BR, G [Morton neg. 8561], NY [NY00172212], W [W-1889-291731, W-003067]).
Solanum neriifolium Bitter, Repert. Spec. Nov. Regni Veg. 11: 482. 1913. Type: Ecuador. Pichincha: in the western declivity of Pichincha, 13,000 ft., Jul 1863, *W. Jameson* s.n. (lectotype, designated by Knapp 1989, pg. 87: W [W-1889-223028]; isolectotype: US [US-3168293]).

##### Type.

Peru. “andiniis Peruviae”, *Humboldt & Bonpland* s.n. (holotype: P-Bonpl. [P00136326, F neg. 39014]; isotype: F [F-976718, frag.]).

##### Description.

Shrubs or small trees, 1–6 m tall. Stems and leaves densely pubescent with golden yellow tree-like trichomes; leaf scars prominently raised, the stem strongly winged between the nodes; new growth densely pubescent with golden-yellow dendritic and tree-like trichomes. Bark of older stems dark brown, somewhat pubescent. Sympodial units plurifoliate, branching usually dichasial, sometimes monochasial. Leaves simple, 5–9 cm long, 1.1–3 cm wide, narrowly elliptic, the upper surfaces drying black, shiny, sparsely puberulent along the veins with golden dendritic or tree-like trichomes, the lower surfaces densely pubescent with short, matted, golden dendritic and tree-like trichomes, the mesophyll usually not visible, these drying golden, occasional sparser or rarely completely absent; primary veins ca. 11 pairs, these impressed above; base attenuate, winged onto the petiole and then onto the stem; margins entire; apex acute or rounded; petiole 2–5 mm long, strongly winged on to the stem and not clearly differentiated from the leaf base. Inflorescences terminal, sometimes appearing lateral from shoot overtopping, often in the fork of the new branches, 2.5–4 cm long, branching 5–7 times, with 8–10 flowers, pyramidal, the axis densely pubescent with golden dendritic and tree-like trichomes; peduncle 1–2 cm long; pedicels 0.6–1.2 cm long, tapering from a basal diameter of 0.5 mm to an apical diameter of ca. 1 mm, nodding at anthesis, densely pubescent with matted, golden dendritic and tree-like trichomes, articulated at the base and inserted in a sleeve ca. 1 mm long; pedicel scars closely spaced and clustered near the inflorescence branch tips. Buds ellipsoid, the corolla strongly exserted from the calyx tube. Flowers all perfect, 4–5-merous. Calyx tube 2–5 mm long, conical, the lobes 1.5–3 mm long, deltate to long-triangular, densely pubescent abaxially with matted dendritic and tree-like trichomes, densely pubescent adaxially with golden dendritic trichomes. Corolla 2–2.4 cm in diameter, violet or occasionally white (Ecuador), lobed 1/2 to 3/4 of the way to the base, the lobes 8–14 mm long, 7–9 mm wide, planar at anthesis, densely pubescent abaxially with golden dendritic trichomes, these denser at the tips of the lobes, adaxially glabrous. Filament tube less than 0.5 mm long; free portion of the filaments 1.5–2 mm long, glabrous; anthers 3.5–4 mm long, 1–1.5 mm wide, loosely connivent, poricidal at the tips, the pores becoming slit-like with age. Ovary globose, glabrous; style 7–9 mm long, glabrous or finely pubescent near the base in a few Ecuadorian specimens; stigma bilobed, the surface minutely papillose. Fruit a globose berry, 1–1.4 cm in diameter, purplish black with thin pericarp; fruiting pedicels 0.8–1.2 cm long, woody, deflexed or nodding. Seeds ca. 15 per fruit, ca. 3 mm long, ca. 3 mm wide, reddish-brown, flattened lenticular or roundish in outline, the surfaces minutely pitted. Chromosome number: not known.

**Figure 93.**
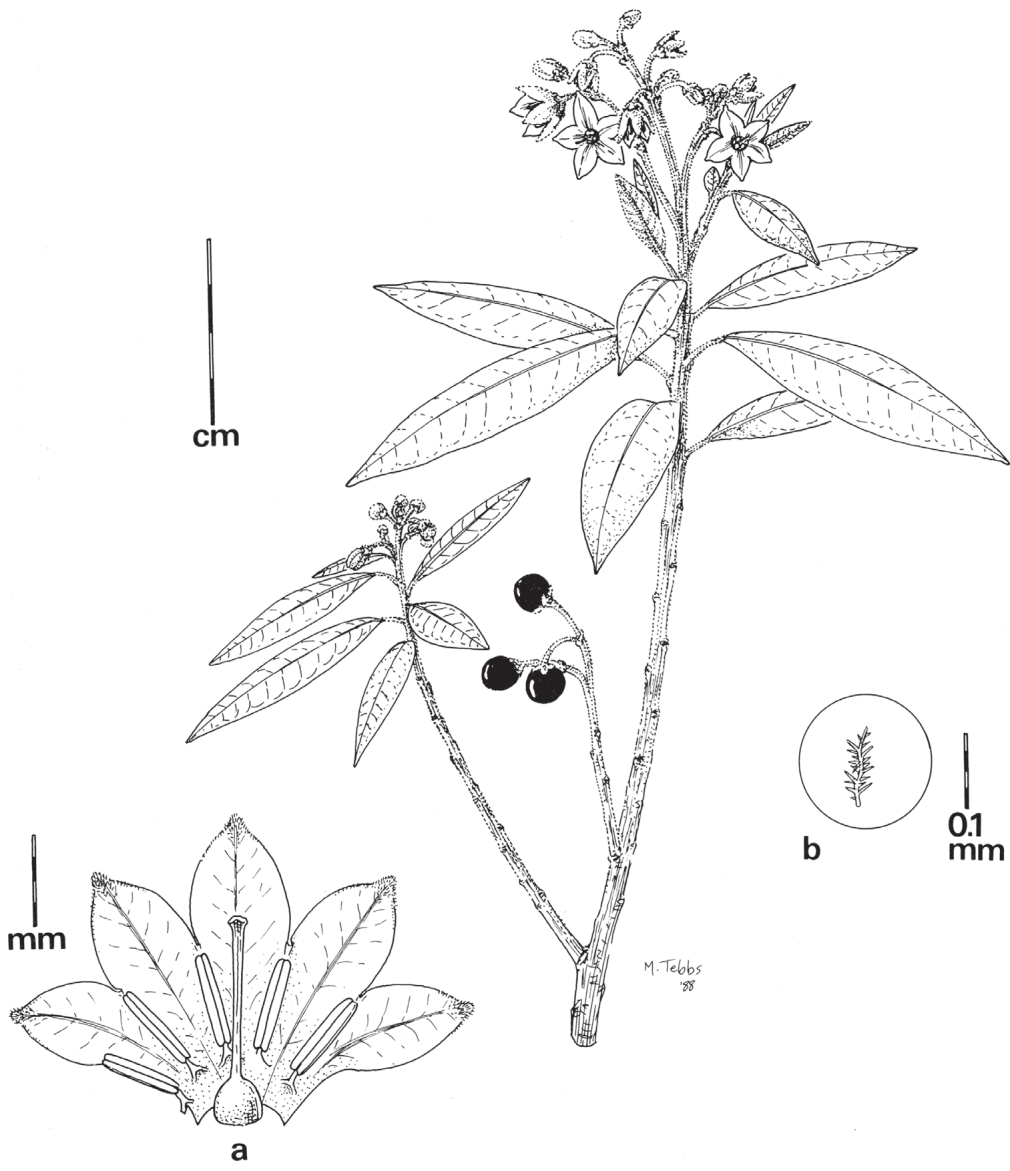
*Solanum stenophyllum* Dunal. (**A–B** drawn from *Pennell 3082*). Reproduced from Knapp (1989) with permission of the Natural History Museum Botany Library. Illustration by Margaret Tebbs.

##### Distribution

**Figure 94.**
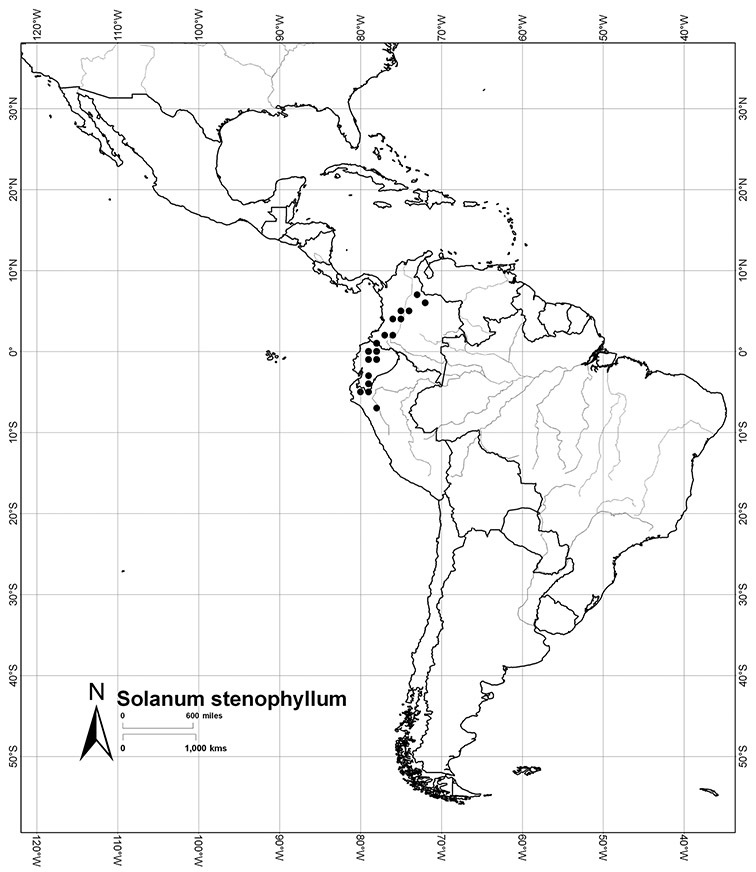
Distribution of *Solanum stenophyllum* Dunal.

(Figure 94). Andes from northern Colombia to southern Ecuador and northern Peru, from 2500–3300 m.

##### Ecology.

Páramo, subáramo and cloud forest.

##### Common names.

Ecuador: pugyán (Knapp 1989).

##### Conservation status.

Least Concern (LC); EOO >100,000 km^2^ (LC) and AOO >10,000 km^2^ (LC). See Moat (2007) for explanation of measurements.

##### Discussion.

*Solanum stenophyllum* is one of the most easily recognised members of the *Solanum nitidum* species group, with its strongly discolorous leaves that are bright yellow beneath. It is most closely related to *Solanum cutervanum* and *Solanum ruizii* (as assessed by morphological cladistics, see Knapp 1989), differing from those species in its winged stems and glabrous styles. In N central Ecuador, near Quito, populations of *Solanum stenophyllum* often have white flowers and occasionally pubescent styles. The leaf pubescence of these plants also appears a brighter shade of yellow when dry than that of other populations. This geographical variant has been called *Solanum neriifolium*. These plants are otherwise identical to the rest of the species range, so these populations are not accorded species status. A few collections of *Solanum stenophyllum* are nearly glabrous and superficially similar to *Solanum imbaburense* and *Solanum coalitum*. Plants of *Solanum stenophyllum*, however, always have at least some tree-like trichomes with very congested branches and appear to be rare regional variants. *Solanum coalitum* is completely glabrous and has swollen calyces, but occurs within the distribution of some of the more glabrous spopulations of *Solanum stenophyllum*. Specimens of *Solanum stenophyllum*, however, always have at least some dendritic and tree-like trichomes on the inflorescence axis or new growth, distinguishing them from the completely glabrous *Solanum coalitum*.

The type locality of *Solanum stenophyllum* cited by Dunal in his original description of the species was “andiniis Peruviae”. Kunth, in his compilation of Humboldt and Bonpland’s collections give the locality as “prope Olleros et Yanta” (in present day Dept. Piura, Peru). Kunth, however, never saw a specimen of this plant, and the type in the Bonpland herbarium at P has three labels attached, the lower two of which give the collection locality as “prope Cuenca”, in present day Ecuador. The plant is a good match for populations around Cuenca, and it is probable that Kunth’s locality citation is in error.

##### Specimens examined.

**Colombia**. **Antioquia**: El Anillo, 3000 m, Feb 1852, *Triana 3855-1* (BM); **Boyacá**: Alto de las Cruces, páramos al NW de Belen, vereda San Jose de la Montaña, Alto de las Cruces y alrededores, 3720 m, 3 May 1973, *Cleef 9708* (K); Nevado de Cocuy, Valle de la Cueva, 3700 m, 10 Sep 1938, *Cuatrecasas 1307* (F); Valle de El Cocuy, La Cueva, Chinchilla, 3800 m, 17 Sep 1969, *Cuatrecasas & Rodríguez 27821* (F); **Caldas**: El Aprisco, Cordillera Central, vertiente occidental, vert. sudoeste del Ruiz, 3500 m, 5 May 1940, *Cuatrecasas 9317* (F); Cordillera Central, vertiente occidental, cabeceras del Río Otun, entre la Laguna del Mosquito y Plan del Villar, 3650 m, 26 Nov 1946, *Cuatrecasas 23251* (F); **Cauca**: Páramo de las Papas, 3100 m, Jan 1947, *Antonio C*. *48* (F); Laguna de San Rafael, Cordillera Central, filo de la Cordillera al N de Volcán Puracé, 3350 m, 29 Jan 1947, *Cuatrecasas 23452* (F); **Cundinamarca**: Páramo de Sumapaz, Chisacá, cabeceras de Río S. Rosa, 3500 m, 11 Dec 1971, *Cleef 212* (K); Páramo de Chisacá, 28 Jun 1967, *Martin & Plowman 64* (ECON, K); Páramo de Chisacá, southeast of Bogotá, 4000 m, Jul 1953, *Schultes 20151* (GH, K); **Nariño**: Pasto, Páramo de las Delicias, near Popayán, 3000 m, 28 Nov 1896, *Lehmann 8686* (F, F, GH, K); Túquerres, Volcán Azufral, eastern slopes, ca. 9-13 km W of Tuquerres, 3500 m, 12 May 1989, *Luteyn et al. 12828* (K); **Santander**: Laguna de Cunta, edge of páramo de Santurbán, 21 Jan 1927, *Killip & Smith 17969* (A, GH); **Tolima**: Páramo del Ruiz, 16 Dec 1917, *Pennell 3082* (GH, US); Páramo del Ruiz, Jul 1846, *Purdie* s.n. (K); **Valle del Cauca**: Quebrada de las Vegas, cabeceras del Río Tuluá, Cordillera central, vertiente occidental, 3400 m, 21 Mar 1946, *Cuatrecasas 20339* (F); Páramo Pan de Azúcar, 3300 m, 23 Aug 1968, *Espinal T. & Ramos 2434* (F).

**Ecuador**. **Azuay**: Páramos de Soldán, Nudo de Cordillera Occidental y Cordillera Oriental, 3350 m, 30 Jul 1959, *Barclay & Juajibioy 8386* (MO); Cumbe, 3300 m, 22 Apr 1968, *Harling et al. 8586* (MO); **Carchi**: El Frailejón, Cordillera Oriental, 3500 m, 11 Aug 1949, *Acosta Solís 13223* (F); Road between Tulcan and Maldonado. Espeletia paramo S of Volcán Chiles, 3800 m, 12 Mar 1985, *Eriksen 59019* (BM); between Tulcan and El Angel, Nov 1952, *Fagerlind & Wibom 1500b* (S); Cantón Tulcán, Tulcán, road to El Angel, 3300 m, 6 Feb 1959, *Harling 4249* (S); El Frailejón, on road Tulcán-El Carmelo (El Pun), 3300 m, 6 Mar 1974, *Harling & Andersson 12534* (MO); Páramo del Ángel, laguna sur de El Voladero, 3750 m, 19 Jan 1983, *Ména V. 80* (MO); Cantón Tulcán, frontera con Colombia, faldas del volcán Chiles, 3900 m, Nov 1993, *Palacios 11873* (BM); San Gabriel-Chutan Alto road, above Chutan Alto, 3500 m, 23 Mar 1989, *Pedersen 95* (AAU); Nudo de Boliche, Voladero, 15 Jun 1939, *Penland & Summers 914* (F, GH); Páramo del Ángel, laguna oriente del Volador, 3700 m, 26 Jan 1967, *Sparre 14163* (S); **Imbabura**: Laguna Mojanda, at the southern part of Laguna Negra, 3700 m, 29 Jun 1983, *Brandbyge 42201* (BM); Otavalo-Lagunas de Mojanda, 3550 m, 23 May 1987, *Jørgensen 61778* (BM); **Loja**: road from Loja to La Tuna, km 14-34, 1600 m, 21 Nov 1961, *Dodson & Thien 1504* (MO); Loja-Saraguro, km 18, 2610 m, 21 Apr 1994, *Jørgensen et al. 477* (BM); Cerro Uritusinga, Loja-La Palma, Km 18-20, 2910 m, 30 Nov 1994, *Jørgensen et al. 1068* (BM); Parque Nacional Podocarpus, trail Cajanuma-Laguna de Compadre, páramo around the first pond, half way to Laguna de Compadres, 3200 m, 26 Dec 1988, *Jørgensen et al. 65663* (AAU); road Loja-Las Achira (Uritusinga), km 9 from Universidad Nacional de Loja, 2800 m, 20 May 2001, *Madsen et al. 8040* (AAU); road Amaluza-Zumba (in construction), ca. km 35, 3400 m, 13 Aug 2001, *Madsen et al. 8323* (AAU); road Universidad Nacional de Loja-Las Achira (Uritusinga), km 10, 2800 m, 25 Oct 2001, *Madsen & Chimbo 8606* (AAU); **Napo**: Los Corrales, near Papallacta, 3900 m, 21 Jul 1960, *Grubb et al. 215* (K); Laguna San Marcos, on the NE slope of Volcán Cayambe, 3600 m, 21 May 1980, *Holm-Nielsen & Balslev 23703* (K); Cordillera de los Llanganates, lower slope of Cerro Crista del Gallo, NW side of Laguna Encantada, 3550 m, 17 Mar 1983, *Holm-Nielsen et al. 41969* (BM); Cayambe, at the Mayorasgo, 4390 m, 1827, *Jameson* s.n. (K); Napo/Pichincha border, páramo de Guamani, small peak c. 6 km S of Paso de la Virgen, 4100 m, 28 Nov 1985, *Laegaard 55710* (AAU); Cocha Seca, carretera Julio Andrade-Playon de San Francisco, 3100 m, 27 Dec 1986, *Zak 1563* (F); páramo de la Virgen, carretera Quito-Papallacta-Baeza, 3900 m, 21 Jun 1987, *Zak 2081* (F); Volcán Cayambe, N slopes, road to the antenna, 3750 m, 9 Jul 1980, *Øllgaard et al. 34231* (K); **Pichincha**: Chaparro de Sebritana, Sec. Oriental de las Hcdas Pedregal y Yanurcu, 3400 m, 7 Jul 1944, *Acosta Solís 8301* (F); SW-slopes of Volcán Atacazo, 3700 m, 28 Oct 1984, *Brandbyge 42819* (BM); Quito-Baeza, near the pass at Papallacta, 3700 m, 30 Oct 1983, *Eriksen & Larsen 45389* (BM); N-NW side of Pichincha, 3600 m, *Fagerlind & Wibom* s.n. (S); Volcán Iliniza, NE slope below the refugio, 4000 m, 13 Aug 1980, *Holm-Nielsen et al. 25017* (F, K); Volcán Atacazo, SW slope, km 19 from San Juan, 2900 m, 25 Aug 1980, *Holm-Nielsen & Asanza 25149* (K); Volcán Pichincha, western slopes of Pichincha, *Jameson* s.n. (GH); Volcán Pichincha, western declivity, 3658 m, *Jameson 48* (K); carretera Cayambe-Olmedo-Laguna San Marcos-Cerro el Mirador, 3700 m, 1 Jan 1988, *Jaramillo 10116* (AAU); Cantón Quito, Quito, *Karsten* s.n. (LE); Quito-Baeza road, between La Virgen (4000 m) and Papallacta (3000 m), 3850 m, Sep 1985, *Priest 263* (K); Laguna de las Hoyas, Paramo de Guarami, 4050 m, 9 Aug 1987, *Ramsay et al. 230* (K); carretera Quito-Guantopugro-Yanacocha, 3400 m, 22 Mar 1987, *Zak 1846* (F); carretera Quito-Nono-Tandayapa, desviación a Yanacocha en al localidad Guanto-pugro, hacienda Alto Peru, estribaciones NO del Volcán Pichincha, 3200 m, 17 Nov 1987, *Zak & Jaramillo 2969* (F, K); Cantón Quito, Parroquia de Tumbaco, area de influencia de la Reserva Ecológica Antisana, Cooperativa Inga Alto Monserrat, 2750 m, 9 Mar 1994, *Ávarez & Columba 1407* (BM); **Tungurahua**: Quebrada Huarcusacha, 3800 m, 27 Jan 1983, *Brandbyge 42041* (BM); Cantón Baños, Cordillera de los Llanganates, crater lake in pass between Río Muyu and Río Topo, 8 km NW of Cerro Hermoso, 4100 m, 8 Nov 1980, *Holm-Nielsen & Jaramillo 28080* (K); Cantón Baños, Cordillera de los Llanganates, loma 3 km SW of Cerro Hermoso, 3700 m, 12 Nov 1980, *Holm-Nielsen & Jaramillo 28691* (K); Cantón Píllaro, Santiago de Pillaro, Parque Nacional Llanganates, base of Cerro Hermoso on western side, 3850 m, 14 Nov 1999, *Neill et al. 12176* (BM); above El Triunfo, páramo El Llanganates, 3700 m, 29 Mar 1989, *Pedersen 42* (AAU); Cantón Patate, Cordillera Los Llanganates, a 12 km del Triunfo, 3100 m, 3 Mar 1995, *Vargas & Sandoval 317* (BM).

**Peru**. **Cajamarca**: Celendín, Sorochuco, subiendo a Michiquillay, 3400 m, 9 Sep 2001, *Sánchez Vega et al. 10957* (BM); **Piura**: Huancabamba, Juzgara, 2900 m, 21 Jan 1994, *Llatas Quiroz 3415* (BM).

#### 39. Solanum storkii

C.V.Morton & Standl., Publ. Field Mus. Nat. Hist., Bot. Ser. 18: 1093. 1938

http://species-id.net/wiki/Solanum_storkii

Figure 95

##### Type.

Costa Rica. San José: Ojo de Agua, 2850 m, June 1932, *H.E. Stork 3023* (holotype: F [F-672907, F neg. 49449]).

##### Description.

Shrubs to medium size (20 cm dbh) trees, 2–10 m tall. Stems and leaves densely pubescent with dull golden echinoid trichomes, the trichome axes rather short, these trichomes deciduous with age; leaf scars somewhat prominent, the stem lightly winged from the decurrent leaf bases; new growth densely pubescent with echinoid and short tree-like trichomes. Bark of older stems grey, sparsely pubescent with a few trichomes of the younger stems. Sympodial units plurifoliate. Leaves simple, 4–10 cm long, 1.3–2 cm wide, narrowly elliptic, the upper surfaces shiny, drying dark, sparsely pubescent with scattered echinoid trichomes on the veins and lamina, the undersurfaces sparsely pubescent with echinoid trichomes, the trichomes slightly sunken beneath the lamina surface; primary veins 8–11 pairs, sparsely pubescent; base attenuate, decurrent on to the petiole; margins entire; apex acute to acuminate; petiole 0.7–1.3 cm long, lightly winged from the leaf bases and on to the stem, not twining. Inflorescences terminal, sometimes appearing lateral from overtopping shoot growth, 3–5 cm long, branching 2–5 times, with 10–15 flowers, pyramidal, densely pubescent with echinoid trichomes; peduncle 1.5–5 cm long; pedicels 0.9–1.1 cm long, tapering from a basal diameter of 0.5 mm to an apical diameter of ca. 1 mm, nodding at anthesis, sparsely to densely pubescent with echinoid and tree-like trichomes, articulated at the base and inserted in a sleeve ca. 0.5 mm long; pedicel scars closely spaced and clustered near the inflorescence branch tips. Buds ellipsoid, the corolla strongly exserted from the calyx tube. Flowers all perfect, 5-merous. Calyx tube 2–3 mm long, conical, the lobes 1–1.5 mm long, deltate, densely pubescent abaxially with echinoid or occasionally tree-like trichomes, sparsely pubescent adaxially with golden dendritic trichomes. Corolla 1.5–2 cm in diameter, violet, lobed ca. 3/4 of the way to the base, the lobes 6–10 mm long, 4–6 mm wide, planar or slightly upturned at anthesis, densely pubescent abaxially with dendritic trichomes, these denser at the tips of the lobes, glabrous adaxially. Filament tube absent; free portion of the filaments ca. 0.5 mm long, glabrous; anthers 4–4.5 mm long, 1–1.5 mm wide, loosely connivent, poricidal at the tips, the pores becoming slit-like with age. Ovary glabrous; style 6–8 mm long, glabrous; stigma minutely bilobed, scarcely distinguishable from the style, the surface minutely papillose. Fruit a globose berry, 1–1.3 cm in diameter, purplish-black with thin pericarp, the juice sticky and very bitter; fruiting pedicels 1.8–2 cm long, ca. 1.5 mm in diameter at the base, woody, erect to slightly nodding. Seeds 8–9 per fruit, 3–4 mm × 1.5–2.5 mm, reddish-brown, flattened or slightly thickened reniform, the surfaces minutely pitted. Chromosome number: not known.

**Figure 95.**
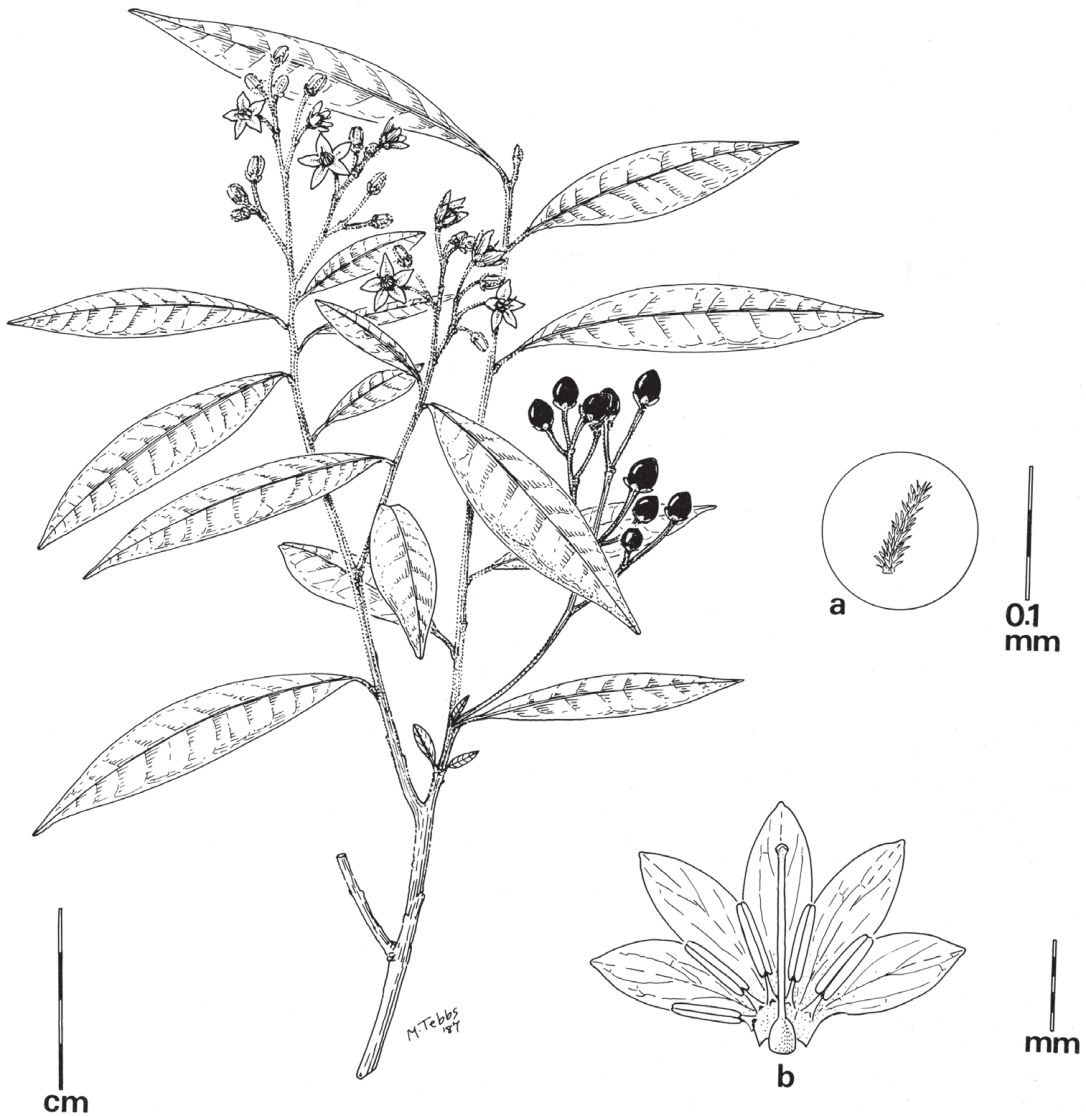
*Solanum storkii* C.V.Morton & Standl. (**A–B** drawn from *Heiser 3489*). Reproduced from Knapp (1989) with permission of the Natural History Museum Botany Library. Illustration by Margaret Tebbs.

##### Distribution

**Figure 96.**
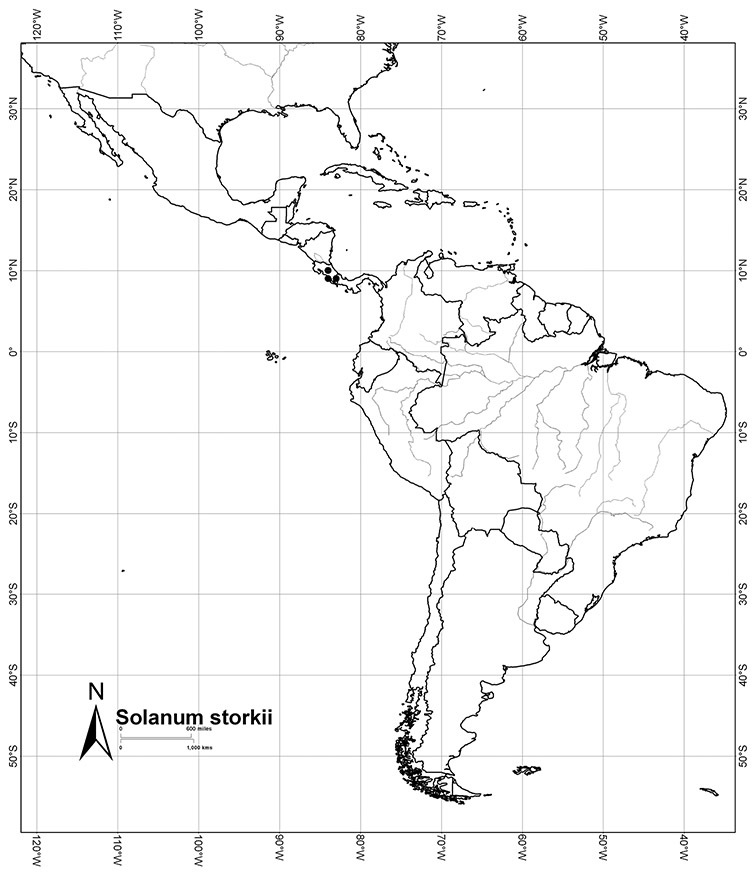
Distribution of *Solanum storkii* C.V.Morton & Standl.

(Figure 96). Cordillera de Talamanca from Volcán Poas in Costa Rica to western Panama, from 2300–3300 m.

##### Ecology.

Páramo, cloud forests and high elevation grasslands, often growing in thickets at the edges of forest patches.

##### Common names.

Costa Rica: quiticirrú (*González 6*).

##### Conservation status.

Vulnerable (VU); EOO <45,000 km^2^ (VU) and AOO >10,000 km^2^ (LC). See Moat (2007) for explanation of measurements.

##### Discussion.

*Solanum storkii*, though of restricted distribution, is locally very common, often forming pure stands above the tree line in the Cordillera de Talamanca in Costa Rica and Panama. Its distribution within its range, however, is somewhat patchy, and stands are commoner in open areas or in the later stages of second growth.

*Solanum storkii* had previously been confused with *Solanum cutervanum* (as *Solanum pulverulentum*, see Standley and Morton 1938), but is quite distinct from that species. It can be distinguished from the rest of the members of the *Solanum nitidum* group in its sparse pubescence of echinoid trichomes, which in dry specimens appear to be in pits on the lower leaf surface, and in its deltate calyx lobes.

##### Specimens examined.

**Costa Rica**. **Alajuela**: Volcán Poás, summit, 2500 m, 1 Dec 1937, *Allen 604* (F); Volcán Poas, Mar 1896, *Donnell Smith 6668* (BM); Volcán Poás, 16 Apr 1973, *Gentry & Burger 2953* (F); Volcán Poás, 2439 m, 6 Jun 1928, *Stork 2515* (F); Poás, 2743 m, 29 Jul 1932, *Stork 3347* (MO); **Cartago**: Cordillera de Talamanca, UCR Reserva along the Carretera Interamericana, 10 Jun 1983, *Barringer 3109* (F); Cerro de la Muerte, near Memorial to Eleazar Barquero Gonzalez, 31 May 1978, *Barrington 581* (MO); near Asunción at the summit of the Interamerican Highway, Provinces of San José and Cartago, 3300 m, 19 Jun 1968, *Burger & Stolze 5972* (F); near Asunción at the summit of the Interamerican Highway, Provinces of San José and Cartago, 3200 m, 6 Aug 1971, *Burger 7941* (F); trail to Cerro Cuerici, E of Villa Mills and Siberia, 2700 m, 8 Feb 1982, *Burger & Barringer 11525* (F); trail to Cerro Cuerici, E of Villa Mills and Siberia, 2700 m, 6 Feb 1982, *Burger & Barringer 11525* (F); Volcán Irazú, 2743 m, 24 Feb 1957, *Carlson 3563* (F); Cerro de la Muerte, Panamerican Highway, 2439 m, 9 Mar 1952, *Carpenter 512* (US); Volcán de Turrialba, Jan 1899, *Donnell Smith 7538* (BM); Cantón de El Guarco, R.F. Río Macho, Cuenca del Savegre, Estación Ojo de Agua, Sendero el Mascarilla, 2950 m, 12 Jan 1996, *Gamboa & Picado 938* (MO); along Interamerican Highway near El Trinidad and km 72, about 20 km SE from Empalme, 2600 m, 15 Mar 1973, *Gentry & Burger 2673* (F); Paraíso, R.F. Los Santos, Cuenca del Savegre, Carretera Interamericana km 88, páramo Buenavista, 3400 m, 25 Nov 1999, *González et al. 973* (MO); S slope of Volcán Irazu, on highway about 5 km NE of Finca Robert, 3000 m, 26 Jun 1949, *Holm & Iltis 142* (BM, F); Cerro de la Muerte, bosque behind Pensión La Georgina, 2600 m, 7 Apr 1994, *Huber & Weissenhofer 1011* (WU); finca ca. 0.5 km E of the Interamerican Hwy. ca. 20 km SE by road from El Empalme, 2500 m, 15 Jul 1970, *Lellinger & White 1152* (F, US); Panamericana Road between Km. 60 and 77, La Trinidad. Cordillera Talamanca, mountain of Cerro La Muerte, 3140 m, 26 Feb 1966, *Molina R. et al. 17845* (US); Cerro de la Muerte, 95.5 km from San José on the Panamerican highway, 3200 m, 19 Jun 1966, *Mori & Anderson* s.n. (F); Hotel Georgina, 100 m W along Carretera Interamericana, common in backyard of hotel, 3200 m, 2 Mar 1971, *Nee & Mori 3542* (F); Volcán de Turrialba, 2800 m, Jan 1899, *Pittier 7538* (F); Cerro de la Muerte, N slopes, 3201 m, 25 May 1971, *Proctor 32074* (F); Volcán Irazú, valley of Río Birrís, S slopes of Volcán Irazu, 2743 m, 11 Jul 1962, *Webster et al. 12131* (F); Cerro de la Muerte, 4.5 mi E of Ojo de Agua, Cordillera de Talamanca, 3033 m, 15 Jul 1962, *Webster et al. 12309* (F); Cerro de la Muerte, km 79 on the Panamerican highway about 17 km NW of Villa Mills, 28 Mar 1967, *Wilbur & Stone 8772* (F); above Hotel Robert, slopes of Volcán Irazú, 3100 m, 14 Mar 1948, *Williams & Molina R. 13878* (F, US); near Ojo de Agua, Cordillera de Talamanca, 3000 m, 26 Jan 1965, *Williams et al. 28276* (US); **Limón**: Cantón de Talamanca, Sabanas de Dúrika, 1 km aguas abajo de la confluencia de los Río Uk y Río Kuk, 2250 m, 20 Oct 1989, *Herrera 3734* (MEXU); **Puntarenas**: Cantón de Coto Brus, P.I. La Amistad, Cordillera de Talamanca. Estación Altamira. Sendero a Casa de Coca al Valle del Silencio, 2100 m, 17 Apr 1995, *Angulo 195* (MO); Cordillera de Talamanca, continental divide halfway between Cerro Dudu and Cerro Nai, 2850 m, 26 Mar 1984, *Davidse et al. 26105* (US); **San José**: Cantón de Pérez Zeledón, Parque Nacional Chirripó, Cuenca Térraba-Sierpe, sendero a Cerro Chirripó, 3100 m, 3 May 1997, *Alfaro 1184* (MO); Cerro Vueltas, Dota, Copey, 2850 m, 8 Apr 2000, *Castroviejo & Sánchez 15128 SC* (MA); northern Cordillera Talamanca, region of Cerro de la Muerte, on Carretera Nacional 2, 2.2 km N of La Georgina Inn, 2934 m, 4 Apr 1978, *Davidson 7241* (F, US); Cantón de Pérez Zeledón, Cuenca Térraba-Sierpe, Estación Cuericí, 2800 m, 24 Sep 1996, *Gamboa R. & Picado 727* (MO); 50 km north of San Isidro de El General on the Carretera Interamericana, Cerro de La Muerte, 12 Jan 1992, *Grant & Rundell 92 1843* (US); Cerro de la Muerte, just off Panamerican highway, 3000 m, 14 Feb 1981, *Knapp 825* (BH); northern slopes of Cerro Buena Vista to south of Interamerican Highway crossing, Cerro de la Muerte, 3250 m, 12 Jul 1994, *Kress & Sawyer 94 4964* (US); foot of Cerro Estaquero, Talamanca Range, 3200 m, 22 Aug 1965, *Lent 728* (F, US); Cantón de Pérez Zeledón, Cord. de Talamanca, Cerro de la Muerte, páramo Buena Vista en los alrededores de las torres del ICE, 3400 m, 19 Oct 1993, *Morales et al. 1891* (MO); Cerro de las Vueltas, 2700 m, 29 Dec 1925, *Standley & Valerio 43729* (US); Cerro de la Muerte, Talamanca Range, high point along Pan American Highway, 3400 m, 8 Aug 1972, *Taylor & Taylor 11724* (US); Dota, páramo de Cerro Buena Vista, en el sitio de las torres del ICE, 3400 m, 12 May 1998, *Valverde 907* (MEXU); slopes of Cordillera de Talamanca near La División north of San Isidro de El General, 2400 m, 6 Feb 1963, *Williams et al. 24390* (F, US).

**Panama**. **Bocas del Toro**: Ridges to W of Cerro Fábrega that lead down to Valle del Silencio, 13.4 km NE from Estacion Pittier, 3200 m, 10 Mar 2006, *Knapp & Monro 9971* (BM); Ridges to E of Cerro Fábrega, 3300 m, 16 Mar 2006, *Knapp & Monro 10053* (BM); Cerro Fabrega, ca. 1.5 km NW of the peak, 3200 m, 17 Mar 2003, *Monro et al. 4150* (BM).

#### 40. Solanum triquetrum

Cav., Icon. Pl. 3: 30, tab. 259. 1795

http://species-id.net/wiki/Solanum_triquetrum

Figure 97

Solanum lindheimerianum Scheele, Linnaea 21: 766. 1848. Type: United States of America. Texas: Comal County, New Braunfels, Aug 1846, *F. Lindheimer s.n. [312/481 III]* (holotype: B?, destroyed; lectotype, designated here: MO [MO-3938181]); isolectotypes: BM [BM000934766], F [F-236914], K [K000438659, K000438657], LE, HAL?).
Solanum triquetrum Cav. var. *lindheimerianum* (Scheele) A.Gray ex Blankinship, Rep. (Annual) Missouri Bot. Gard. 18: 145. 1907. Type: Based on *Solanum lindheimerianum* Scheele.

##### Type.

Spain. Cultivated in Madrid, originally from Mexico, *Anon*. (lectotype, designated by Knapp 2007a, pg. 202: MA [MA-476365]; possible isolectotype: F [F-844667]).

##### Description.

Semi-woody vine or scrambler with an enlarged woody base, to 2 m long, occasionally an erect subshrub to 0.5 m tall. Stems somewhat angled, glabrous to pubescent with weak simple uniseriate trichomes to 0.5 mm long, these usually ascending and pointing to the distal part of the stems; new growth sparsely to densely pubescent with simple uniseriate trichomes to 0.5 mm long, but usually shorter. Bark of older stems pale greenish yellow. Sympodial units plurifoliate. Leaves simple to basally lobed and hastate, (1.1-)1.8–5(+) cm long, (0.3-)1–3.5 cm wide, deltate to hastate, triangular in outline, sometimes linear, slightly fleshy, the upper and lower surfaces pubescent with simple uniseriate trichomes along the margins and on the veins to uniformly pubescent on the veins and lamina, the trichomes to 0.5 mm long, white; primary veins 3–5 pairs, only just visible in dried material, sometimes slightly impressed above; base abruptly truncate to hastate with 2 basal lobes to 1 cm long, these rounded at the tip; margins entire to basally lobed; apex acute to acuminate, the tip sometimes rounded; petioles 0.3–1.2 cm long, glabrous but with a line of simple trichomes adaxially, apparently only sometimes twining. Inflorescences terminal or lateral, 1–3 cm long, usually simple, very occasionally furcate, with 3–6 flowers, glabrous to pubescent like the stems, the pubescence like that of the rest of the plant; peduncle 0.3–1 cm long; pedicels 0.6–1.2 cm long, ca. 0.5 mm in diameter at the base, ca. 1 mm in diameter at the apex, slender, sometimes tinged purple, glabrous to sparsely pubescent with simple uniseriate white trichomes, articulated at the base from a short sleeve 0.5–1 mm deep; pedicel scars irregularly spaced 1–4 mm apart. Buds when young globose, later ellipsoid, the corolla strongly exserted from the calyx tube before anthesis. Flowers all perfect, usually 5-merous. Calyx tube 1–1.5 mm long, conical, the lobes 1.5–2 mm long, triangular with the tips often long-acuminate, often purplish tinged, glabrous to sparsely pubescent abaxially, densely papillate adaxially, pubescent with simple trichomes to 0.2 mm on internal surface of tips. Corolla 1.5–1.9 cm in diameter, white or tinged with purple, often with a shiny green or greenish white eye, stellate, lobed nearly to the base, the lobes 0.8–0.9 mm long, ca. 0.3 cm wide, reflexed at anthesis, the midvein often tinged purple, glabrous to sparsely pubescent abaxially especially along the distal margins and tips, glabrous adaxially. Filament tube minute, the free portion of the filaments ca. 1 mm long, glabrous; anthers 3.5–4 mm long, 1–1.5 mm wide, ellipsoid, loosely connivent, poricidal at the tips the pores lengthening to slits with age. Ovary glabrous; style 6–8 mm long, glabrous; stigma capitate to somewhat clavate, the surface minutely papillose. Fruit a globose to slightly ellipsoid berry, to 1.5 cm in diameter, bright red when ripe, the pericarp thin and shiny, glabrous; fruiting pedicels 1–1.5 cm, ca. 1 mm in diameter at the base, somewhat woody, deflexed or spreading. Seeds (2-)6–10 per berry, ca. 4 mm long, 2.5 mm wide, flattened to thickened reniform, reddish brown, the surfaces minutely pitted, in mature fruits the lateral testal cell walls exposed and the seed prominently silky, the testal cells rectangular to slightly sinuate. Chromosome number: not known.

**Figure 97.**
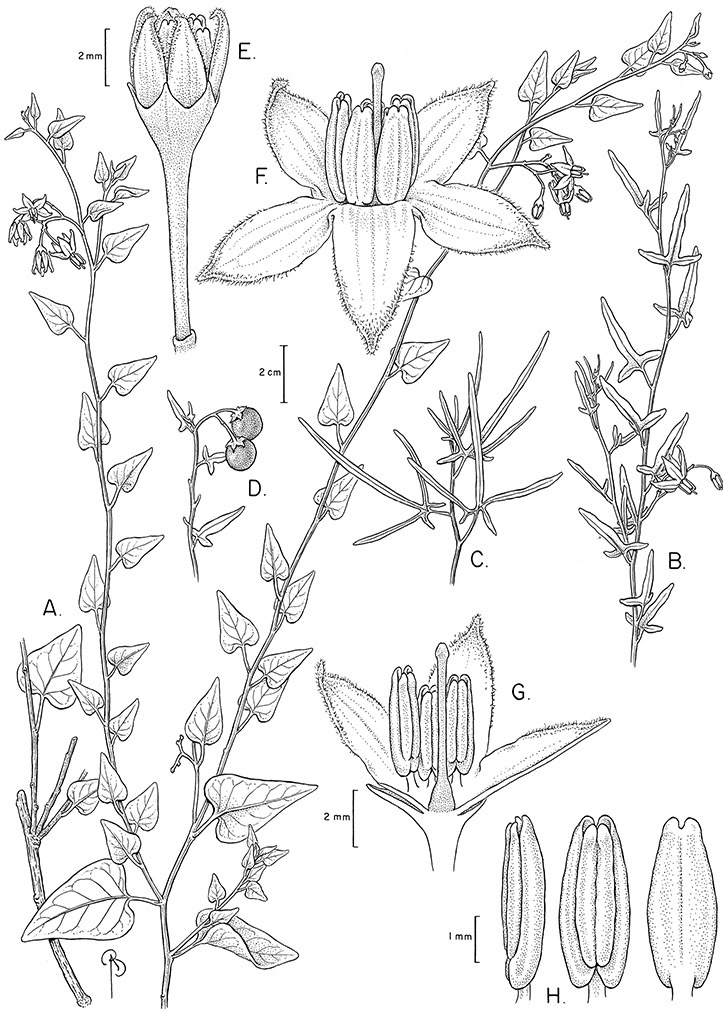
*Solanum triquetrum* Cav. (**A** drawn from *Lindheimer 1044* **B** drawn from *Chiang 7857* **C** drawn from *Waterfall 6278* **D** drawn from *Pringle 153* **E–H** drawn from *Burr 452*). Illustration by Bobbi Angell.

##### Distribution 

**Figure 98.**
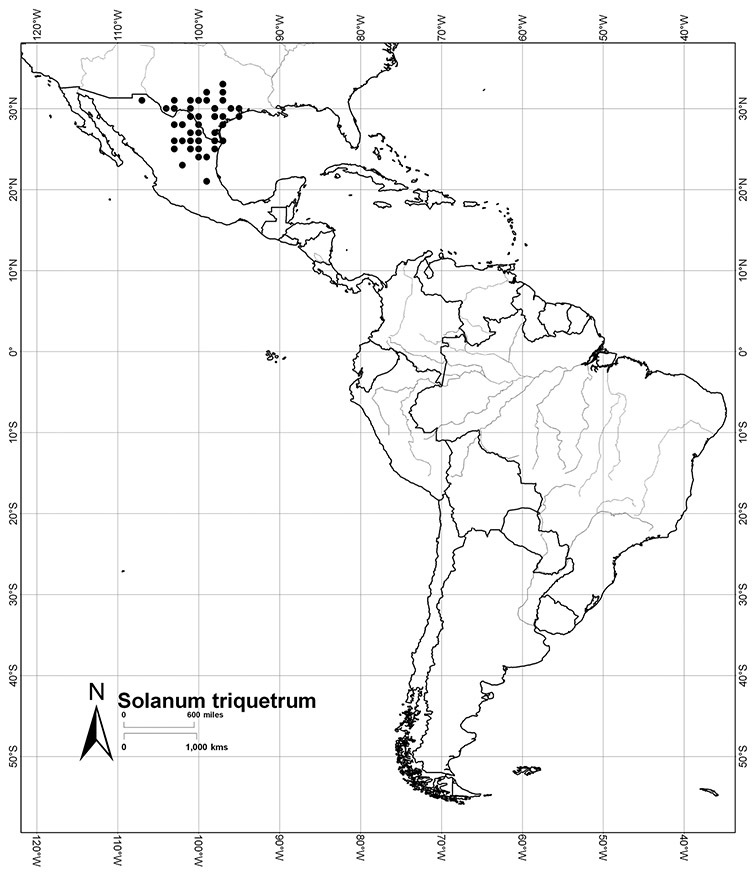
Distribution of *Solanum triquetrum* Cav.

(Figure 98). In the USA in central, south and west Texas and northern Mexico, south to the state of Coahuila, from sea level to 500 m elevation.

##### Ecology.

On slopes and in thickets; often growing in moist places.

##### Common names.

USA. Texas: Texas nightshade (http://plants.usda.gov/java/profile?symbol=SOTR2).

##### Conservation status.

Least Concern (LC); EOO >100,000 km^2^ (LC) and AOO >10,000 km^2^ (LC). See Moat (2007) for explanation of measurements.

##### Discussion.

*Solanum triquetrum*, like most other members of the Dulcamaroid clade, has incredibly variable leaf morphology. Leaves vary from small, triangular in outline (hence the specific epithet meaning three –angled) and simple to quite deeply lobed at the base to very rarely pinnatifid with two or three pairs of lobes. Leaf size also varies a great deal, perhaps in response to moisture, some specimens with particularly large leaves appear to have been collected in wetter microhabitats. The only species in North America with which *Solanum triquetrum* could be confused is *Solanum dulcamara*, an introduced European taxon. *Solanum triquetrum* differs from *Solanum dulcamara* in its free anthers, more pointed leaves, strongly angled stems and smaller flowers. Both species have a green “eye”, often with shiny white margins, at the center of the corolla at the base of the stamens. *Solanum triquetrum* usually has white flowers, while *Solanum dulcamara* has purple flowers, but white-flowered plants of *Solanum dulcamara* occur throughout its range, both native and introduced. Like *Solanum dulcamara*, *Solanum triquetrum* arises from a subterreanean woody rootstock and may die back in winter or in particularly dry seasons.

Knapp (2007) lectypified *Solanum triquetrum* with a sheet cultivated at MA; the fragment at F was taken from the MA sheet and is thus an isolectotype. The location of the type of *Solanum lindheimerianum* is somewhat problematic. Ferdinand Lindheimer and J. J. Roemer collected together in Texas in 1846, and Roemer gave his collections and many duplicates of Lindheimer’s to Scheele, a clergyman from near Hildesheim, Germany. Scheele described many of Lindheimer’s plants before Engelmann and Gray had a chance to do so, and duplicates of these collections were only distributed in the early 20^th^ century (Blankinship 1907). The location of Scheele’s herbarium and types is not known, but it is likely they were retained by Roemer in Berlin. Fortunately many duplicates of Lindheimer’s Texas collections were distributed by Engelmann and Gray. The sheet from the Engelmann herbarium at MO [MO-3938181] has a label in Lindheimer’s hand with the date “August 1846” and the locality “New Braunfels” and is the logical choice for a lectotype for *Solanum lindheimerianum*. A BM sheet dated 1846 is clearly a duplicate of that gathering based on its morphology. The numbering of Lindheimer’s collections is of two sorts: 1) Lindheimer’s field collection numbers, and 2) the exsiccatae numbers given after the plants were organized into taxonomic sequence and applied to plants of the “same” species collected on several dates (Blankinship 1907). Confusion over duplicates can arise when one or the other number is not cited. For *Solanum lindheimerianum* the exsiccatae number is 481, while Lindheimer’s field collection number is 312. Blankinship (1907: 145) published the combination “S. triquetrum Cav. var. Lindheimerianum Gray”, citing *Lindheimer 312* (along with other Lindheimer collections) and pointed out that the two taxa were almost certainly synonyms. Asa Gray apparently did not publish this combination formally, but as Blankinship (1907) made explicit reference to Scheele’s publication of *Solanum lindheimerianum*, the correct citation for the variety is *Solanum triquetrum* var. *lindheimerianum* (Scheele) A. Gray ex Blankinship.

##### Specimens examined.

**Mexico**. **Coahuila**: Saltillo, 1400 m, 1 May 1949, *Hinton 16775* (GH); Saltillo, sierra E of Saltillo, 1500 m, 26 Sep 1949, *Hinton 16855* (GH); San Vicente, along road from San Vicente SW about 7 miles to southern end of Laguna de Jaca, 9 Sep 1940, *Johnston & Mueller 1067* (GH); La Ventura, road from state boundary, north to Saltillo (6 mi N of La Ventura), 12 Sep 1938, *Johnston 7640* (GH); San Antonio de los Álamos, eastern base of the volcanic Sierra de San Antonio, 20 Aug 1941, *Johnston 8259* (GH); Múzquiz, La Mariposa, 5 Dec 1936, *Marsh 1032 A* (F, GH); Monclova, 5 May 1939, *Marsh 1657* (F, GH); La Ventura, 1896, *Nelson 3900* (GH, US); Torreon and vicinity, 13 Oct 1898, *Palmer 476* (BM, F, G, GH, K, S, UC); Perras, 11.5 miles W of Salsillo, 22 Apr 1880, *Palmer 930* (GH, LE, P); Jimulco, 12 May 1885, *Pringle 153* (BM, E, F, G, GH, GOET, K, L, LE, US); Sierras Negras, 9 km S of Parras, 2400 m, 3 Jul 1941, *Stanford et al. 141* (GH); Toboso, flat 4 km W of San Juan, SW of Sierra de las Cruces, 11 Jul 1941, *Stewart 813* (GH); 23 miles SW of Monterrey, 1 Dec 1945, *Warnock & Barkley 148* (GH); Saltillo, 70 miles west, 7 Aug 1957, *Waterfall & Wallis 13286* (F); Monclova, 609 m, 5 Jul 1939, *White 1789* (ECON); Ramos Arizpe, Hacienda La Rosa, 14 Jun 1936, *Wynd & Mueller 34* (GH, US); **Hidalgo**: Zimapán, *Douglas 1229* (K); Zimapán, 22 Jun 1947, *Kenoyer 1104* (GH); **Nuevo León**: Monterrey, Acequia Guadelupe, Jul 1911, *Arsène 6424* (US); Linares, S of Monterrey on Pan-American highway, 28 Apr 1939, *Frye & Frye 2518* (GH, UC, US); Aramberri, La Ascensión, to Sandia, 1800 m, 16 Sep 1890, *Hinton 20406* (GH); Galeana, El Peñuelo, 1725 m, 24 Jul 1891, *Hinton 21115* (GH); Monterrey, Monterrey, South of I.T.E.S.M. campus, 487 m, 14 Jul 1970, *Imboden 39* (EIU); El Carrizo, near Monterrey, 6 Nov 1903, *Lozano* s.n. (US); Monterrey, 11 Sep 1902, *Pringle 11062* (BH, F, GH, GOET, K, US); Mirador, near Monterrey, 1946, *Roybal 748* (US); Icamole, mountains near town, 3 Feb 1907, *Safford 1253* (US); Monterrey, 11 Oct 1895, *Seler & Seler 1070* (GH); Paso de Mamulique, 365 m, 14 Mar 1984, *Thompson et al. 1287* (F); **San Luis Potosí**: sin. loc, 1878, *Parry* s.n. (GH); Salado, 24 mi. S of Salado, 1692 m, 22 Aug 1940, *Shreve & Tinkham 9633* (GH); **Tamaulipas**: Tigre Crossing, south of crossing, 10 Feb 1939, *LeSueur 370* (F); Laguna Anda la Piedra, 14 Feb 1939, *LeSueur 427* (F); Mun. San Fernando, San Fernando, northern outskirts, 3 Jan 1981, *Nee et al. 19434* (F); Ciudad Victoria, southeastern outskirts of city, 400 m, 22 Oct 1981, *Nee 22211* (F); E of Matamoros, 25 Oct 1907, *Rose & Russell 24215* (K); **Zacatecas**: La Pendencia, 8 km NW, 1475 m, 16 Jun 1972, *Chiang et al. 7857* (F).

**United States of America**. **New Mexico**: sin. loc, 1851, *Wright 1591* (F, F, G, GH, K, US); **Texas**: **Bell County**: Little River, near Little River, *Wolff 395* (US); Belton, 12 Feb 1932, *Wolff 3449* (US); **Bexar County**: San Antonio, 19 Oct 1927, *Rose & Rose 24143* (GH, US); Seguin Road, 4 miles east of San Antonio, 14 Aug 1931, *Sister Mary Clare* s.n. (S); Camden Road near Elmendorf Lake, 4 Apr 1940, *Sueltenfuss 174* (BM); Converse, 2 miles N of Converse, 28 Jun 1929, *Wolff 975* (US) **Brewster County**: 9-Point Mesa, summit and upper slopes, on 9-Point Mesa Ranch about 60 miles S of Alpine, 22 Sep 1966, *Correll 33801* (GH); Alpine, 22 miles E of Alpine, 2 Jul 1941, *Smith T-1197* (US); Altuda Mountain, Glass Mtns, 26 Jun 1940, *Warnock 326* (GH); **Brookes County**: Talfurrias, Salt Lake at Gypsum Mine SE of Talfurrias, 23 Nov 1940, *Innes 313* (GH); Brownwood, 30 Oct 1924, *Palmer 26758* (A, S); **Calhoun County**: Port Lavaca, shore of bay, 3 Aug 1946, *Gentry 38* (F); East Kararkawa Point, 9 Sep 1922, *Tharp 1609* (US); **Cameron County**: Rio Hondo, Jul 1913, *Chandler 7011* (GH, US); Las Palmas Plantation, 4 miles SW of Brownsville, 4 Oct 1952, *Correll 14842* (GH); Brownsville, 11 miles NW, 18 Mar 1960, *Gentry & Barclay 18420* (US); Brownsville, by Pan American airport, 26 Feb 1939, *Muenscher & Muenscher 14443* (GH); Laguna Atoscosa National Wildlife Refuge, Unit 2, N side of Impoundment 2, ca. 100 m W of its intersection with road, 1 m, 24 Apr 1959, *Traverse 1098* (F, GH); **Chambers County**: sin. loc, 7 Apr 1936, *Tharp* s.n. (GH); **Comal County**: Comanche Spring: New Braunfels, etc, Jun 1850, *Lindheimer 1045* (BM, E, F, G, GH, K, LE, US); New Braunfels, 1 mile west of Landis Park, 21 May 1946, *Warnock 46352* (S) **Crockett County**: Ozona, 11 3/4 miles E of Ozona, 3 Nov 1937, *Cory 26624* (GH); **Dallas County**: Dallas, 27 Oct 1900, *Bush 1621* (GH, K, US); Dallas County, Dallas, bottom of Trinity River, Jun 1876, *Reverchon* s.n. (US); Dallas County, Dallas, Jun 1877, *Reverchon 671* (US); Dallas County, Fort Worth, 9 Sep 1877, *Ward* s.n. (US); **De Witt County**: Cuero, 22 Mar 1907, *Howell 306* (US); **Edwards County**: Substation #14 Yard, 26 Aug 1940, *Cory 35253* (GH); **Fort Bend County**: Duke, 2 Oct 1914, *Palmer 6703* (F, US); **Galveston County**: field E of road that goes N to “Bob’s Place” from highway 87, 8.7 miles W of Gilchrist, 3 m, 25 May 1961, *Traverse 2330* (A); **Guadelupe County**: Seguin, old Zorn House, 3 Apr 1949, *Cory 55414* (US); **Hays County**: San Marcos, 167 m, 1 Aug 1943, *Fisher 43037* (B, US); **Hidalgo County**: Mission, 10 Nov 1940, *Cory 36042* (GH); 12 miles north of Mission, 4 Apr 1941, *Lundell & Lundell 9948* (US); Red Gate, 1.5 miles S of Red Gate, along side road to Laguna Seca Historical Marker, just W of Hwy. 281, 22 m, 19 Jan 1981, *Nee 20070* (F); McAllen, 18 Dec 1981, *Nee 24056* (F, MA); Santa Ana National Wildlife Refuge, 14 Apr 1977, *Solomon 2705* (LE); **Hudspeth County**: Eagle Mountain, E slopes, about 6 miles S of Hot Wells, 7 Jul 1943, *Waterfall 4925* (GH); Eagle Mountain, in valley running into Eagle Mountains about 1/4 mile S of the flouride mine, 31 Aug 1945, *Waterfall 6278* (GH); Eagle Mountain, slopes between flouride mine and Eagle Peak, 31 Aug 1945, *Waterfall 6330* (GH); **Kimble County**: US routes 183-283, north of Junction, 12 May 1947, *McVaugh 8293* (F, G, GH); **Maverick County**: Eagle Pass, 2 Aug 1931, *Clark 4078* (B, G); Normandy, 5 miles N, 10 Sep 1962, *Scora 2289* (US); **Nueces County:** Corpus Christi, 26 Sep 1958, *Correll 20405* (GH); Corpus Christi, 5 Mar 1894, *Heller 1399* (E, GH, K, LE, US); **Pecos County**: 13.5 miles E of Fort Stockton along Highway 290, 17 Apr 1965, *Correll & Correll 30886* (GH); **Presidio County**: Crawford Tank, on Holland and Meriwether Ranch, NE side of the Sierra Tierra Vieja, ZH Canyon, Espy Miller Ranch, 1500 m, 7 Sep 1941, *Hinckley 2120* (GH); **San Patricio County**: Portland-Gregory, 17 Mar 1929, *Tharp 5608* (US); Welder Wildlife Foundation, 175 m E of Moody’s Camp, 10 m, 4 May 1959, *Traverse 1221* (F, GH, US); **San Saba County**: San Saba, 12 May 1930, *Harris 711* (US); **Sutton County**: Sonora, 7 1/3 miles N of Sonora, 28 Oct 1945, *Cory 50553* (GH, US); **Tarrant County**: Fort Worth, along Trinity River, 31 Oct 1925, *Palmer 29476* (GH); **Taylor County**: Camp Barkeley, 20 Oct 1942, *Tolstead 5833* (GH); **Terrell County**: Spofford, 8 May 1904, *Griffiths 6274* (US); **Tom Green County**: San Angelo, 3 Jul 1916, *Palmer 10356* (S, US); Knickerbocker Ranch, Dove Creek, May 1880, *Tweedy* s.n. (US); along South Concho River near Cristoval, 6 Jul 1933, *Wolff 4189* (F); **Travis County**: Austin, 19 May 1872, *Hall 494* (F, GH, US); Austin, near 51st St and Duval, 20 Feb 1995, *Nesom & Nesom 7* (F, GH, K, US); Zilker Park, in valley of Braton Creek, ca. 2 mi upstream from Barton Springs, 2.5 mi from Colorado River, ca. 0.5 mi NE of Loop 360 bridge over creek, Barton Creek Greenbelt, 182 m, 31 May 1989, *Orzell & Bridges 10186* (S); **Valverde County**: Goodenough Spring, 15 miles south of Comstock near the Rio Grande, 13 Nov 1964, *Correll & Correll 30667* (GH); Del Rio, 289 m, 19 Aug 1932, *Fisher 32248* (US); Del Rio, 13 May 1918, *Palmer 13590* (B, GH, K, US); **Wilson County**: Sutherland Springs, Sep 1879, *Palmer 929* (GH, K, LE); **Zavalla County**: Nueces River, 11.5 miles S of Uvalde, 24 Oct 1934, *Cory 11954* (GH).

#### 41. Solanum umbelliferum

Eschsch., Mém. Acad. Imp. Sci. St. Pétersbourg Hist. Acad. 10: 283. 1826

http://species-id.net/wiki/Solanum_umbelliferum

Figure 99

Solanum genistoides Dunal, Prodr. [A.P. de Candolle] 13(1): 85. 1852. Type: United States of America. California: sin. loc., 1833, *D. Douglas* s.n. (holotype: G-DC [G00144979, Morton neg. 8400]; isotypes: BM [BM000937786], GH [GH00077420], K [K000658255, K000658282]).
Solanum californicum Dunal, Prodr. [A.P. de Candolle] 13(1): 86. 1852. Type: United States of America. California: sin. loc., 1833, *D. Douglas* s.n. (lectotype, designated by McVaugh 2000, pg. 505: G-DC [G00144968, Morton neg. 8401]; isolectotypes: BM [BM000838183], GH [GH00077415], K [K000658276, K000658251]).
Solanum menziesii Dunal, Prodr. [A.P. de Candolle] 13(1): 159. 1852. Type: United States of America. California: Monterey County, Monterey, *A. Menzies* s.n. (holotype: BM [BM000934858]).
Solanum umbelliferum Eschsch.var. *glabrescens* Torr., Pacific Railr. Rep. 7, Pt. 3: 17. 1856. Type: United States of America. California: San Bernardino County; between San Bernardino and San Gabriel, 1854-1855, *T. Antisell* s.n. (lectotype, designated here: NY [NY00821174]).
Solanum umbelliferum Eschsch.var. *incanum* Torr., Pacific Railr. Rep. 7, Pt. 3: 17. 1856. Type: United States of America. California: Monterey County, head[waters] of the San Antonio [River], 1855, *T. Antisell* s.n. (holotype: NY [NY00820618]).
Solanum xanti A.Gray, Proc. Amer. Acad. Arts 11: 90. 1876. Type:United States of America. California: Kern County, Fort Tejon and vicinity, near lat. 35°and long. 119°, 1857-1858, *L.J. Xantus de Vesey 73* (lectotype, designated by Parish 1901, pg. 161: GH [00077430]).
Solanum palmeri Vasey & Rose, Proc. U. S. Natl. Mus. 1888, 11: 532. 1889. Type:Mexico. Baja California: Bahia San Quintin [San Quentin Bay], Feb 1889, *E. Palmer 704* (holotype: US [US00027766]; isotypes: LE, NY [NY00139015, NY00139016]).
Solanum cupuliferum Greene, Erythea 3: 72. 1895. Type: United States of America. California: Napa County, Napa Valley near Oakville, 1 Apr 1895, *E.L. Greene* s.n. (neotype, designated here: NDG [NDG-045023]).
Solanum tenuilobatum Parish, Proc. Cal. Acad. ser. 3, Bot 2: 165. 1901. Type: Mexico. Baja California: near Ensenada, Apr 1882, *C.C. Parry* s.n. (holotype: GH [GH00077504]).
Solanum wallacei (A.Gray) Parish var. *viridis* Parish, Proc. Cal. Acad. Sci. ser. 3, Bot. 2: 166. 1901. Type: United States of America. California: Monterey County, Pacific Valley, May 1897, *A. Eastwood* s.n. (two syntypes cited, CAS, DS, neither found, see discussion).
Solanum xanti A.Gray var. *intermedium* Parish, Proc. Calif. Acad. Sci. ser. 3, Bot. 2: 168. 1901. Type: United States of America. California: San Bernardino County, vicinity of San Bernardino, 12 May 1897, *S.B. Parish 4388* (lectotype, designated here: JEPS [JEPS-12142]; isolectotypes: BM [BM000934851], GH [GH00077431], MO [MO-884365, MO-2045917], US [US-313423]).
Solanum xanti A.Gray var. *glabrescens* Parish, Proc. Calif. Acad. Sci. ser. 3, Bot. 2: 169. 1901. Type: United States of America. California: San Bernardino County, vicinity of San Bernardino, 5 May 1897, *S.B. Parish 4384* (lectotype, designated here: JEPS [JEPS-12146]; isolectotypes: DS [DS-106503], MO [MO-2045937, MO-884367], NY [NY00743231], US [US-313421]).
Solanum umbelliferum Eschsch. var. *californicum* (Dunal) Parish, Proc. Cal. Acad. Sci. ser. 3, Bot. 2: 172. 1901. Type: Based on *Solanum californicum* Dunal
Solanum parishii A.Heller, Muhlenbergia 2: 133. 1906. Type:Based on *Solanum xanti* A.Gray var. *glabrescens* Parish
Solanum xanti A.Gray var. *spencerae* J.F.Macbr., Contr. Gray Herb. 65: 43. 1922. Type: United States of America. California: San Diego County, Torrey Pines near San Diego, 28 Mar 1919, *M.F. Spencer 1069* (holotype: GH [GH00077432]; isotype: NY [NY00138960]).
Solanum arborescens Clokey, Bull. S. Calif. Acad. Sci. 30: 60. 1931, non *Solanum arborescens* Moench (1794). Type: United States of America. California: Santa Barbara County, Santa Cruz Island, Pelican Bay, 23 May 1930, *I.W. Clokey 5047* (lectotype, designated here: UC [UC905430]; isolectotypes; A [A00077418], G [G00357912], GH [GH00077417], JEPS [JEPS12106], POM [POM-199830], RSA [RSA-19599], UC [UC535574]).
Solanum clokeyi Munz, Bull. S. Calif. Acad. Sci. 31: 69. 1932. Type: Based on *Solanum arborescens* Clokey
Solanum xanti A.Gray var. *montanum* Munz, Bull. S. Calif. Acad. Sci. 31: 70. 1932. Type: United States of America. California: San Bernardino County, north end of Bear Valley, San Bernardino Mountains, 6000-8500 ft., 26 Mar 1925, *P. Munz 5718* (holotype: POM [POM-13481]; isotype: UC [UC218202]).
Solanum xanti A.Gray var. *hoffmannii* Munz, Bull. S. Calif. Acad. Sci. 31: 70. 1932. Type: United States of America. California: Santa Barbara County, Gaviota Pass, *P. Munz 9315* (holotype: POM [POM-98450]).
Solanum obispoense Eastw., Leafl. West. Bot. 1: 104. 1934. Type: United States of America. California: San Luis Obispo County, Santa Margarita, El Dorado School, 20 Apr 1933, *M.E. Wall* s.n. (lectotype, designated here: CAS [CAS-204657]; isolectotypes: CAS [CAS-204658], DS [DS-230359], GH [GH00077425], POM [POM-203041], US [US-1651755]).
Solanum wallacei (A.Gray) Parish var. *clokeyi* (Munz) McMinn, Man. Calif. Shrubs 491. 1939. Type: Based on *Solanum arborescens* Clokey.

##### Type.

United States of America. California: [San Francisco County], San Francisco, *J.F.G. von Eschscholtz* s.n. (lectotype, designated here: LE).

##### Description.

Shrubs or subshrubs, erect or somewhat spreading, to 1.5 m tall, spreading to 2 m diameter. Stems glabrous to densely pubescent with simple uniseriate trichomes to 2 mm long, these glandular or not, if shorter than 0.5 mm then sometimes antrorse, often with an enlarged cell at the base, mixed with shorter very delicate simple uniseriate trichomes with a single-celled glandular tip, and/or dendritic trichomes to 0.5 mm long, and glandular papillae with a multicellular head, usually winged from the decurrent leaf bases, in densely pubescent plants the wings not easily visible; new growth sparsely to densely pubescent with trichomes like those of the stems. Bark of older stems pale green or yellowish gray, somewhat glabrescent. Sympodial units plurifoliate. Leaves simple or shallowly pinnatifid with 1 (-3) pairs of lobes at the base, (0.5-)1–4(-9) cm long, 0.5–2(-6.5) cm wide, lanceolate to ovate or obovate, membranous or somewhat thick and leathery, both surfaces glabrous to densely pubescent with a mixture of trichome types, these simple uniseriate trichomes to 2 mm long, the dendritic trichomes to 0.5 mm long, all trichome types sometimes glandular (but less so than the trichomes of the stems), the pubescence somewhat denser along the veins; primary veins 5–8 pairs, often drying yellowish cream; base attenuate to truncate, occasionally slightly cordate; margins entire to crisped, often densely glandular papillate, the papillae with multicellular glands, the leaves sometimes lobed in the basal part, the lobes divided less than halfway to the midrib, the apex acute to rounded; apex acute to rounded; petioles 0.2–1.5(-3) cm long, glabrous to densely pubescent like the leaves and stems, never twining. Inflorescences terminal or lateral, 1–8 cm long, simple or once branched, with 5–20 flowers, only a few open at a time, glabrous to densely pubescent with simple uniseriate trichomes to 2 mm long or dendritic trichomes to 0.5 mm long, the trichomes sometimes but not often glandular; peduncle 0.5–2(-5) cm long, in branched inflorescences flowers only occurring above the branching point; pedicels 1–1.5 mm long, ca. 0.5 mm in diameter at the base, ca. 1 mm in diameter at the apex, slender, spreading, glabrous to densely pubescent with simple or dendritic trichomes, these usually sparser than on the inflorescence axis, in live plants sometimes dark purple, articulated at the base and inserted into a short sleeve 0.5–1 mm long; pedicel scars rather unevenly spaced 1–4 mm apart. Buds obovoid or obovoid-ellipsoid, included in the calyx tube until just before anthesis. Flowers all perfect, 5-merous. Calyx tube 1.5–3 mm long, broadly cup-shaped, the lobes 1–2.5 mm long, broadly deltate, glabrous to sparsely or densely pubescent with simple or dendritic trichomes like those of the pedicels.Corolla (1-)1.3–2.3(-2.6) cm in diameter, violet to deep purple or occasionally white, with green spots edged with white at the base of the lobes, the spots separate or confluent, rotate, lobed < 1/4 of the way to the base, the lobes 0.5–3.5 mm long, 1.5–6 mm wide, apiculate, the apiculae ca. 1 mm long, abaxially sparsely to densely pubescent with tangled weak simple uniseriate trichomes to 0.5 mm long along the lobe midveins, the tissue of the sinuses glabrous on both surfaces. Filament tube minute, the free portion of the filaments 1–2 mm long, occasionally one or two filaments longer than the rest, glabrous or pubescent with minute weak tangled simple trichomes to o0.1 mm long; anthers 3.5–4.5 mm long, 1–1.5 mm wide, ellipsoid, loosely connivent or spreading and not touching, poricidal at the tips, the pores lengthening to slits with age. Ovary glabrous; style 7–9(-10) mm long, minutely glandular papillate with weak unicellular papillae to 0.1 mm long in the basal third, appearing glabrous with a lens; stigma capitate, the surface minutely papillate, green in live plants. Fruit a globose berry, 1–2 cm in diameter, green, greenish black or black when ripe, the pericarp thin and shiny, glabrous; fruiting pedicels 1.2–2 cm long, 1–1.5 mm in diameter at the apex, woody and spreading with the weight of the fruits. Seeds 50–60 per berry, ca. 2 mm long, 1.5 mm wide, flattened reniform, reddish brown, the surfaces appearing minutely pitted with sinuate cells, the cell walls thick at the base, topped with filiform projections ca. 0.05 mm long and the seed appearing hairy when mature, especially at the margin. Chromosome number: not known.

**Figure 99.**
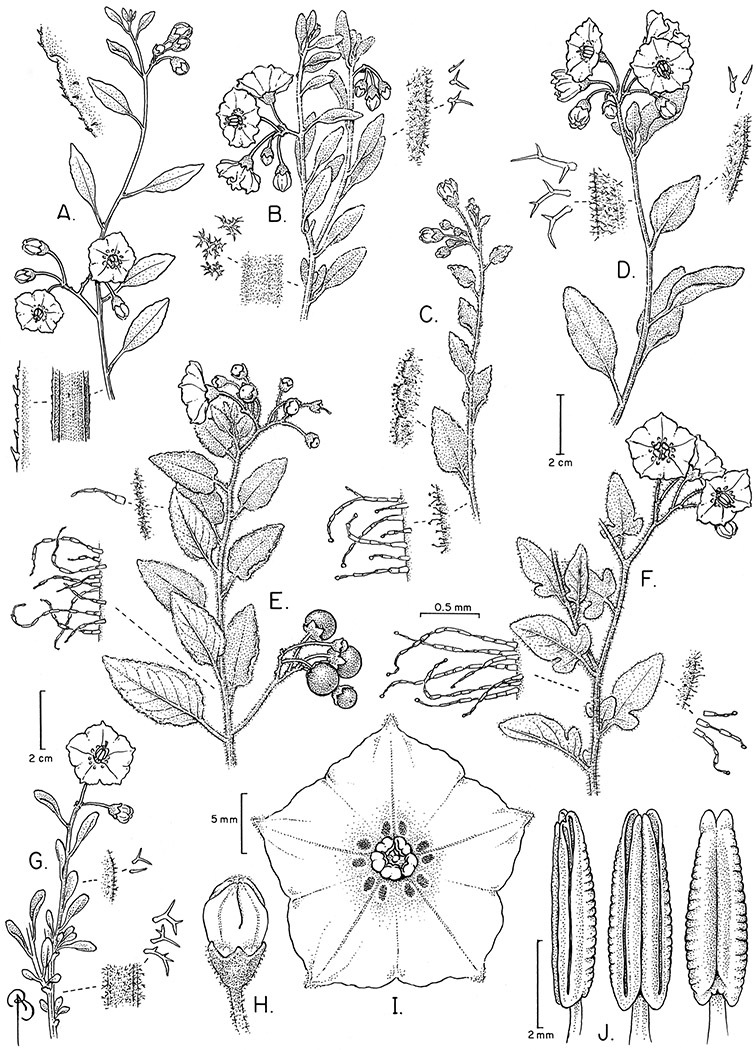
*Solanum umbelliferum* Eschsch. (**A** drawn from *Joyal & Mehlmar 1272* **B** drawn from *Halse 6149* **C** drawn from *Twisselman 3423* **D** drawn from *Anderson 2886* **E** drawn from *Blakley 3954* **F** drawn from *Chamberlain* s.n. **G–J** drawn from *Williams 68-3-10*). Illustration by Bobbi Angell.

##### Distribution

**Figure 100.**
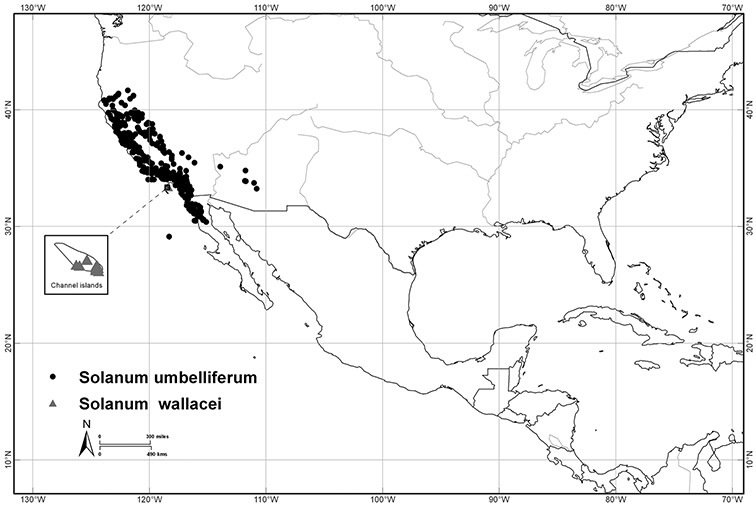
Distribution of *Solanum umbelliferum* Eschsch. and *Solanum wallacei* (A. Gray) Parish (inset)

(Figure 100). Common in the western part of North America from Oregon (USA) to Baja California (Mexico), extending eastwards to Nevada and Arizona, in California absent from the Sacramento valley, from sea level to ca. 2000 m in the Sierra Nevada mountains.

##### Ecology.

*Solanum umbelliferum* occurs in a wide variety of habitats from sand dunes to chaparral, Coastal Sage scrub and pine forests in the Sierra Nevada mountains.

Common names. USA, California: blue witch, bluewitch nightshade (CalFlora, http://www.calflora.org/cgi-bin/species_query.cgi?where-taxon=Solanum%20umbelliferum , USDA Plants, http://plants.usda.gov/java/profile?symbol=SOUM )

##### Conservation status.

Least Concern (LC); EOO >100,000 km^2^ (LC) and AOO >10,000 km^2^ (LC). See Moat (2007) for explanation of measurements.

Discussion. *Solanum umbelliferum* and its close relative *Solanum wallacei* are among the most easily recognised species of the Dulcamaroid clade in being woody shrubs or subshrubs that do not climb with twining petioles with rotate purple (rarely white) corollas with prominent almost ruffled interpetalar tissue. Only *Solanum flaccidum* and *Solanum laxum* of southern Brazil (*Solanum laxum* also in widespread cultivation) have corollas with very short lobes that are almost rotate, but both those species have white flowers and are vines that climb with their petioles. *Solanum viscosissimum*, also of southern Brazil, shares densely glandular pubescence with *Solanum umbelliferum* (at least some populations; see below), but it is a vine with white flowers. The flowers of *Solanum umbelliferum* have two green spots that are edged with white at the base of each petal lobe underneath the anthers. This character is shared with *Solanum dulcamara*, *Solanum lyratum*, *Solanum septemlobum* and *Solanum pittosporifolium*, with which *Solanum umbelliferum* is closely related according to DNA sequence data (L. Bohs, unpublished). Those four species are all herbaceous or slightly woody vines, climbing by twining petioles, and have stellate corollas (except *Solanum septemlobum* with a more pentagonal corolla and consistently lobed leaves). The green spots have variously been interpreted as “pseudonectaries” and pollinator attractants (Buchmann et al. 1977, also see above under *Solanum dulcamara*).

The species I am recognising here as *Solanum umbelliferum* has normally been treated as several taxa, differing only by pubescence density and type. Parish (1901: 163–164) characterized these plants as being those for which “their satisfactory segregation is a matter of no little difficulty. The exercise of that botanical industry that multiplies “species” by the minute description of individuals might reap here an abundant harvest.” He used variation in pubescence as the key character for distinguishing his taxa but also admitted that this character was continuous. For Parish, however, recognition of taxa was of importance and so he recognised as taxa what he called “certain diverging lines of development…without insisting upon definite cleavages, which do not exist.” He considered the treatment of this complex as a single taxon unacceptable, but given the variability in leaf form and pubescence present in many other taxa in the Dulcamaroid clade, I feel that it is better to recognise the variation as interesting and complicated, rather than suggesting, by the recognition of separate taxa, that it is understood. That some of this variation is environmental is clear; a pair of collections from Ventura County (*Abrams & McGregor 154, 160*) in open and shaded habitats show striking differences in leaf size and hairiness. To really understand this group more field work with local populations needs to be undertaken, coupled with transplant experiments and more detailed genetic marker work. A preliminary survey from across the species range revealed little (1 base pair) or no variation at several plastid markers (George 2011; George et al. in prep.), but more detailed studies with microsatellites or AFLP markers may reveal differentiation.

The following extremes of leaf shape and pubescence have been recognised at the species level:

Plants with dense dendritic eglandular stem pubescence (such that the stems are white and no wings can be seen) and small leaves have been called *Solanum umbelliferum*. The types of *Solanum californicum* (with large leaves) and *Solanum genistoides* (with very small leaves) come from these populations; both these were collected by David Douglas in 1833. Gray (1876: 91) considered *Solanum genistoides* as a “small, summer-leaved form” of the former. The type of *Solanum umbelliferum* is from the San Francisco area, and although it has dendritic pubescence, it is not as dense as these extreme populations from Kern and San Luis Obispo counties.

Plants with simple uniseriate glandular trichomes with tangled tips and a relatively large basal cell have been distinguished as *Solanum xanti*; the type of *Solanum xanti* from Fort Tejon at GH consists of two stems, one of which is densely glandular and the other eglandular.

Plants from the Channel Islands (with the exception of Santa Catalina Island) and Isla Guadelupe in Mexico with long simple uniseriate trichomes that are either gland-bearing or not have been occasionally recognised as. *Solanum clokeyi* (or as a variety of *Solanum wallacei*). These plants are very similar to those from coastal populations in Santa Barbara County and without label data cannot be distinguished from them; they are also superficially similar to *Solanum wallacei* from Santa Catalina Island, but have smaller flowers and fruits.

Glabrous plants from northern California and adjacent Oregon with a few simple antrose uniseriate trichomes have been recognised as *Solanum parishii*; these plants also often have narrower leaves with more attenuate bases than populations with glandular pubescence. Glabrous plants from southern California and Baja California have been considered conspecific with these northern populations, based on lack of pubescence.

Plants from San Diego County and northern Baja California with narrow lobed leaves have been recognised as *Solanum tenuilobatum*.

Plants from northern Baja California and adjacent Isla San Martin with more deeply lobed leaves and slightly cordate leaf bases have been distinguished as *Solanum palmeri*; these plants have dendritic pubescence like that of northern populations and are not usually glandular.

Plants with very small leaves with crisped margins and glandular branched pubescence have been recognised as *Solanum obispoense*.

I have lectotypified *Solanum umbelliferum* with a sheet at LE labelled with the locality “San Francisco”; additional sheets bear no locality information, but are likely to have come from similar populations as they are morphologically very similar. These could be isolectotypes, but there is no evidence that they are duplicates.

I have lectotypified *Solanum umbelliferum* var. *glabrescens* with the one of two syntypes at NY that has flowers [NY00821174], the other syntype [NY00821142] is sterile and is a very fragmentary specimen. The only specimen cited in the description of *Solanum umbelliferum* var. *incanum* has been taken as the holotype; Torrey was working at NY when he wrote up the botany of the explorations of the American West for the purposes of the new railway.

Parish (1901) was fortunately quite explicit about his choice of gatherings and the herbaria in which the duplicates were seen as types for his taxa in this group. I have selected lectotypes from amongst the duplicates specifically cited by him based on their completeness (flowers and fruits). The two sheets of *Eastwood* s.n. (“Pacific Valley, Miss Eastwood, May, 1897, Type [A, P]”) cited by Parish (1901) in the protologue of *Solanum wallacei* var. *viridis* have not been found at either DS (P – Parish’s own herbarium, later incorporated into the Dudley herbarium) or CAS (A – his abbreviation for the California Academy) and are possibly no longer extant. I have not neotypified this name in the hopes that a duplicate might be found eventually. If a neotype is selected it should be from among the other paratypes cited: *Coulter 586* (GH), *Plaskett* s.n. (CAS) or *Eastwood* s.n. (CAS, “Mt. Tamalpais, 1896”).

In describing *Solanum cupuliferum*, Greene (1895) did not cite any specimens, but gave a short description of the distribution of his new species: “Hills of the Coast Ranges of California from Marin and Napa counties northward..” Two sheets in the Greene herbarium at NDG are potentially original material; I have selected NDG-045023 as the neotype as it has both flowers and fruits; the other sheet (NDG-045022) is possible a duplicate, as it was also collected on 1 April 1895, but the locality is “hills W of Oakville” and not exactly the same as the locality on the neotype. All other Greene collections I have seen are post-1895 gatherings.

Clokey (1931) explicitly cited a sheet in his own herbarium in as the type of *Solanum arborescens* (“my number 5047 in my own herbarium is designated as the Type” – Clokey 1931). The sheet at UC (UC905430) stamped “Clokey Herbarium” is selected here as the lectotype; duplicates are widely distributed.

In early annotations on herbarium specimens I suggested that *Heller 7899* from Shasta County (cited in the protologue of *Solanum parishii*; Heller 1906) was the type of that name. It is clear, however, that Heller (1906: 134) was proposing a replacement name for Parish’s *Solanum xanti* var. *glabrescens*, not describing a completely new taxon.

Several duplicates of the collection used by Alice Eastwood to described *Solanum obispoense* have been found; I have selected that at CAS [CAS-204658], the institution where she worked, as the lectotype.

##### Specimens examined.

**Mexico**. **Baja California**: Isla San Martín, off the coast from Cabo San Quintin, 12 Jan 1973, *Anderson 3253* (RSA); San Vicente, 15 miles S of San Vicente, 182 m, 28 Apr 1950, *Benson 14313* (POM); Valle Las Palmas, Peninsular Ridges, Cerro Bola, just southwest of summit with communications towers, 1229 m, 9 Apr 2001, *Boyd et al. 10342* (RSA); Isla Todos Santos, 10 Mar 1897, *Brandegee* s.n. (UC); Ensenada, al norte de Ensenada, Sep 1930, *Bravo H. 256-7407* (MEXU); Ensenada, between village of Santa Catarina and lower dam, 64 miles southeast of Ensenada, 1036 m, 19 Aug 1961, *Broder 581* (DS, MEXU); Rincón, 1.5-2.5 miles upstream from Rincón, 4.5 miles northeast of Santa Catarina, 64 miles southeast of Ensenada, 1250 m, 22 Apr 1962, *Broder 732* (DS, MEXU); Isla Guadalupe, 914 m, 17 Jun 1906, *Brown* s.n. (GH); San Agustín, ridge 33.6 km northwest of San Agustin, 18 Jan 1948, *Carter et al. 2557* (UC); Río Santo Tomás, headlands, 6 Mar 1947, *Cooper 2171* (RSA); (RSA); Punta Banda, NE slopes, ca. 10 mi S of Ensenada, 22 Mar 1981, *Dillon et al. 1832* (RSA); Ensenada, 24 miles N of Ensenada, 26 Mar 1949, *Dressler 459* (MO); Valle Redondo, hills NE of Valle Redondo, 30 May 1932, *Fosberg 8395* (DS, POM); Canyon Guadalupe, 7 miles north of San Vicente, 180 m, 4 Jan 1988, *Franklin 5835* (UC); Cañada Grande, 22 Feb 1923, *Gallegos 551* (MEXU); San Matías Pass, 29 Mar 1936, *Harbison* s.n. (RSA, UC); Isla San Martín, above small fishing camp near cove, near mouth of Lava Tube, ca. 200 m W of village towards top of island, 22 Jan 1975, *Henrickson 14540* (RSA); Sierra Juárez, southern end, along dirt road from Valle Trinidad to Arroyo Calentura, 1036 m, 2 Apr 1951, *Hohenthal 1* (UC); Colonet, 15 Apr 1925, *Jones* s.n. (POM); Ensenada, 10 Apr 1882, *Jones 371* (CAS, MO, POM, UC); San Antonio Canyon, mesa above canyon, N of San Quintin, 1 May 1923, *McKeever 28* (US); Rancho San José, 25 miles E of San Telmo, foothills of Sierra San Pedro Martir, 792 m, 1 Mar 1931, *Meling 27* (DS, GH); Sierra San Pedro Mártir, ca. 9.7 miles northeast of Mikes Sky Rancho towards El Burro, 16 May 1983, *Michener et al. 4283* (RSA); 28 mi E of Mexican Highway 1 on the San Telmo road to observatorio, 700 m, 5 May 1992, *Miller et al. 7413* (MEXU, MO); Las Filipinas, 5 miles southeast, Sierra Juarez, 1620 m, 30 Jun 1962, *Moran 9840* (DS, UC); Isla San Martín, east slope of San Martin Island, 20 m, 11 Apr 1963, *Moran 10550* (DS, GH, MEXU, RSA, UC); Sierra San Pedro Martir, Rancho San Matias, 1450 m, 6 May 1963, *Moran 10888* (DS, MEXU, RSA, UC); Punta San José, slopes 2 mi NE of point, 200 m, 29 May 1966, *Moran 13203* (RSA); Portezuelo de Jamau, 1300 m, 20 May 1967, *Moran 13876* (RSA); Isla Todos Santos, south island, 15 m, 23 Jun 1969, *Moran 16230* (RSA, UC); Cerro Matomí, NNE ridge, 1550 m, 3 May 1973, *Moran 20774* (POM, UC); Arroyo Durazno, 3 miles south of San Isidoro, 1100 m, 3 Jun 1975, *Moran 22336* (UC); 3 km SE of Punta Cabras, 50 m, 11 Feb 1979, *Moran 26414* (UC); Tecate, 50 miles SE, 13 May 1925, *Munz 9561* (POM); Colonet, 18 km N on Highway 1 (km 111), 22 Jan 1983, *Prigge & Prigge 4244* (MO); Ejido México, arroyo 4 km south of ejido, 300 m, 4 Apr 1958, *Raven et al. 12221* (GH, UC); Descanso, 3 km south of Descanso, 15 m, 24 Apr 1958, *Raven et al. 12692* (GH, UC); Ensenada, Rancho El Potrero, 40 km al SW de el Observatorio de San Pedro Martír, 1050 m, 1 May 1987, *Tenorio & Romero de T. 13240* (MEXU); Ensenada, Rancho El Coyote, 60 km al W de El Observatorio de San Pedro Martír, 790 m, 2 May 1987, *Tenorio & Romero de T. 13264* (GH, MEXU); Red Rock, above Hamilton Ranch, about 140 miles S of San Diego (CA), 22 Mar 1958, *Thomas 121* (DS); Isla de Todos Santos, S island, 15 Mar 1980, *Thorne et al. 53964* (BH); Los Arbolitos, SW coast 24 km S of Ensenada, 50 m, 1980, *Thorne et al. 53988* (RSA); Laguna Hanson, on road to Ojos Negros, 8 miles SW of Laguna Hanson, 1500 m, 28 May 1983, *Thorne et al. 55925* (RSA); Valle de Trinidad, about 2.3 miles NW of Valle de Trinidad, 900 m, 15 Nov 1983, *Thorne & Wisura 57670* (RSA); Bocana de Santo Tomás, 6.7 mi from junction of Hwy 1 and along road to Bocana de Santa Tomas, along valley of Rio Santo Tomas, 75 m, 16 Nov 1983, *Thorne & Wisura 57696* (RSA); Mesa La Misión, upper edge, N end of mesa, S of Valle La Mision, 3 mi. SE of Hwy 1, bridge over Rio San Miguel, 125 m, 18 Apr 1985, *Thorne & Charlton 60023* (RSA); Cerro Bola, elevation of peak with microwave towers, 600 m, 14 Mar 1987, *Thorne et al. 62136* (RSA); Rancho Loma Linda, Las Animas, S of Hwy 1, 200 m, 15 Mar 1987, *Thorne et al. 62268* (RSA); San Quintín, base of Volcanos to W, N of Pichacho Vizcaino, opposite Isla San Martin, 40 m, 29 May 1987, *Thorne et al. 62650* (E, RSA); Guadalupe, 1 miles south of Guadelupe, about 28 miles north of Ensenada, 8 Sep 1929, *Wiggins & Gillespie 3913* (A, CAS, DS, GH, MEXU, MO, POM, US); San Vicente, 5 miles S of San Vicente, 2 Feb 1935, *Wiggins 7550* (DS, UC, US); Mission Santa Catarina, southern end of Sierra de la Juarez, 1182 m, 28 Sep 1938, *Wiggins 9136* (DS); Valle Trinidad, near summit of grade about 2 1/2 miles northwest of village, 3 Jan 1960, *Wiggins & Wiggins 16074* (DS, GH, K, LE, MEXU).

**United States of America. Arizona**: **Apache County:** Fort Apache, 21 Jun 1890, *Palmer 607* (GH, K); **Cochise County:** Natane Plateau, near Bisbee, 26 Jun 1912, *Goodding 1083* (E); **Coconino County:** Oak Creek Canyon, Coconino National Forest, 2 May 1942, *Cantelow* s.n. (CAS); Oak Creek, 1.5 miles below Oak Creek bridge on State Highway 79, 2 Jul 1940, *Ferris 9896* (DS); Upper Oak Creek, 18 Jun 1934, *Fulton 9674* (DS, POM); Oak Creek Canyon, about 7 miles south of Oak Creek Lodge, S of Flagstaff, 14 Jun 1927, *Goddard 611* (UC); Elden Mountain, mouth of canyon below TV hill and 0.3 miles NW of Elden Spring, 20 yards up the slope, Coconino National Forest, 2189 m, 15 May 1985, *Ricketson 2503* (MO); **Gila County:** Collom Camp, Matzatzal Mountains, 1200 m, *Collom 8* (GH, US); Miami, 1219 m, 10 Jun 1931, *Gillespie 5298* (DS, US); Sierra Ancha Experimental Forest, headquarters, 22 Aug 1968, *Keil 3541* (F); Pine, between Pine and Power House, 11 May 1931, *McKelvey 2151* (GH, US); Payson, Alpine Heights, 26 Apr 1984, *Partch 336* (RSA); Oracle Road, 12 miles S of Globe, 1219 m, 29 Apr 1922, *Wiegand & Upton 4229* (F, MO); Pine, 10 miles northwest of Pine, 1646 m, 1 Jul 1928, *Wolf 2449* (DS, GH, RSA); **Greenlee County:** 27 miles north of Clifton, Coronada Trail, vicinity of Cherry Lodge, 7 Jun 1935, *Maguire* s.n. (GH); Crook National Forest, 16 miles N of Clifton, 7 Jun 1935, *Maguire 11856* (UC); **Maricopa County:** Horseshoe Dam Road, ca. 46 mi NNE, 23 Apr 1960, *Crosswhite 763* (K); Mazatal Mountains, NE of Phoenix, along USFS Rd 143 from Hwy 87 to Four Peaks, along Picadilla Creek, 14 Apr 1984, *Daniel & Wagner 3438* (CAS); Columbine Spring, 21 Apr 1947, *Goodding 17 -47* (UC); Alder Creek, Mazatzal Mountains, 5 May 1931, *Harrison 7806* (DS, US); Tonto National Forest, road to Seven Springs at Camp Creek Wash, ca. 0.3 mi S of Sears-Kay road, 914 m, 7 May 1999, *Landrum et al. 9436* (F, RSA); along road from Carefree to the Bartlett Reservoir, ca. 6.1 miles from the reservoir, 4 Apr 1972, *Smith 1446 A* (F); Camp Creek, 17 Apr 1954, *Wright 23 -54* (RSA); **Mohave County:** Granite Canyon, Hualapai Mountains, below the falls, 1 Nov 1931, *Braem 23* (DS, POM); Martin’s Canyon, Hualpai Mountains, 12 May 1940, *Braem 784* (DS); Sawmill Canyon, Hualapai Mountains, along Hualapai Mountain Road, ca. 10 mi SE of Kingman, 1616 m, 5 Oct 1997, *White 5995* (RSA); Blue Tank Wash, head of wash, Hualapai Mountains, 1783 m, 2 Jun 1972, *Phillips et al. 3068* (UC); **Navajo County:** El Capitan, 5 miles north, 13 May 1935, *Maguire 11333* (RSA, US); **Yavapai County:** Sedona, Verde River valley, Aug 1937, *Gentry 3096* (CAS); 8 miles N of Congress Junction on road to Hillside, 914 m, 9 Apr 1947, *Gould & Darrow 4163* (GH); Prescott, 1 Jun 1927, *Peebles & Harrison 4186* (US); , Oak Creek, 22 Jun 1883, *Rusby* s.n. (E, F, MO, S, UC); **California**: **Alameda County**: valley a few miles southeast of Livermore, 11 Mar 1951, *Dress 3046* (BM, E, LE); Berkeley, Strawberry Canyon, 28 May 1933, *Howell 11352* (CAS, GH, US); Oakland, Oakland Hills, east of Mills College, W slope of Devil’s Punch Bowl, 50 m, 16 Apr 1967, *Kasapligil 4074* (UC); Oakland, Hills in back of the Mountain View Cemetery, 5 Apr 1936, *Lee 1847* (JEPS, MO); Berkeley, on the north side of Strawberry Canyon, 396 m, 8 Apr 1956, *Raven 8980* (CAS); Corral Hollow above Tesla, Mount Hamilton Range, 304 m, 8 Mar 1936, *Sharsmith 3410* (DS, GH, UC); off the south side of Norris Canyon Rd. near the Contra Costa Co. line. 5 miles east of Castro Valley, 152 m, 17 May 1975, *Sinnott et al. 266* (CAS); Hills near Berkeley, San Francisco Bay Region, 121 m, 4 Apr 1902, *Tracy 1338* (DS, UC); Mines road, 5 mi S of Livermore, 457 m, 20 May 1984, *Williams & Roderick 84-6-2* (RSA); Arroyo Mocho, 228 m, 14 Mar 1936, *Yates 5366* (UC); **Alpine County**: Ebbett’s Pass, 18 Jun 1940, *Eastwood & Howell 8503* (CAS, POM); Iceberg Meadow Ranger Station, Clarks Fork Trail on Red Mtn., Stanislaus Forest, 2500 m, 10 Jul 1913, *Eggleston 9583* (US); Noble Canyon, Silver Creek, east slope of Sierra Nevada, 2134 m, 29 Jul 1955, *Munz 21311* (RSA); Silver Creek Public Camp, east base of Ebbetts Pass, 2103 m, 28 Jul 1955, *Munz 21346* (RSA); along Ebbett’s Pass Highway, Markleeville Quadrangle, Mono National Forest, 2225 m, 2 Jul 1935, *Thomas 127* (UC); near Chipmunk Flat Dardanelles Quadrangle, Stanislaus National Forest, 2439 m, 2 Jul 1935, *Yates 5324* (UC); **Amador County**: Sierra Nevada, Along State Hwy 88, 23 miles east of Pine Grove, 1646 m, 14 Jun 1962, *Breedlove 3642* (CAS); Sierra Nevada, above Buck-horn Lodge, 19 miles northeast of Jackson, 914 m, 13 Jun 1937, *Crum 1936a* (B); South bank of Cosumnes River opposite of Clark Creek, 3 May 1928, *Mason 4485* (GH, UC); 5 1/2 miles WSW of Bisbee Peak, Jackson Quadrangle, 152 m, 24 Apr 1936, *Nordstrom 758* (RSA, UC); Buena Vista Pk, Jackson Quadrangle, 228 m, 2 May 1935, *Roseberry 130* (UC); **Butte County**: Bardees Bar Road, south of the small stream, about 1/4 mile southwest of the poor bridge across the North Fork of the Feather River at Bardees Bar, about 2 3/4 miles (air); southwest of Pulga, 405 m, 19 May 2006, *Ahart 12633* (CHSC, JEPS, RSA); Clear Creek, 15 Apr 1897, *Brown 191* (E, GH, MO, US, US); Jonesville, 1600 m, 27 Jul 1930, *Copeland 453* (BM, E, F, LE, UC); Berry Canyon, near Clear Creek, 6 May 1902, *Heller & Brown 5455* (DS, GH, MO, US); Hills 14 miles northeast of Chico near Rock creek in the Quercus Douglasii belt, 12 Apr 1915, *Heller 11814* (A, CAS, GH, MO); W side of Dark Canyon Rd, ca. 3 mi S of its jct with Big Bend Rds jct, ca. 4 air mi S of Jarbo Gap on Hwy 70, N of Oroville, 366 m, 5 Apr 1981, *Taylor 3502* (MO); Ca. 0.5 mi N of Kunkle Reservoir, on E side of Pentz-Magalia Hwy, ca. 3 mi S of Paradise, 6 Apr 1984, *Taylor et al. 5413* (MO, RSA); **Calaveras County**: 4 miles W of Columbia toward Angels Camp. Big Trees Quadrangle, 457 m, 16 Apr 1936, *Belshaw 1876* (UC); Lake Alpine, 30 May 1931, *Branson, I*., *s.n.* (CAS); Angels Camp, 11 Apr 1923, *Eastwood 11656* (CAS); Big Meadows, North Fork Stanislaus River, 1981 m, Jul 1931, *Leonard* s.n. (JEPS); 1/2 mile from Parrots Ferry bridge, on road to Vallecito, 12 Jun 1952, *Quick 52-07* (CAS); 3/4 mi NW of Central Ferry. Copperopolis Quadrangle, 259 m, 25 Apr 1935, *Rutter 164* (UC); about 4 miles NE of Murphy, 914 m, 21 May 1927, *Stanford 393* (DS, GH, MO); Near Burson, 152 m, 12 May 1928, *Stanford 992* (DS, MO, POM, US); **Colusa County**: “Grapevine Grade” to Lodoga, 10 mi NW of Sites, 26 Apr 1950, *Bacigalupi & Holmgren 3175* (JEPS, UC); Arbuckle, Rumsey Road, 14 Apr 1917, *Ferris 590* (DS, MO); near Stonyford, 23 Apr 1926, *Ferris 6591* (DS); Salt Cyn, N side of Salt Creek, Hwy 20, 10 miles WSW of Williams. Wilbur Springs Quadrangle, 143 m, 16 Apr 1977, *Kelly 8* (MO); Timber near College City, 18 Jun 1916, *Stinchfield 299* (DS); **Contra Costa County**: Martinez Mountain, South Coast Range, Sacramento River watershed, 91 m, 18 Mar 1931, *Benson 2658* (POM); Mt. Diablo, Back Creek, 11 May 1975, *Bowerman* s.n. (JEPS); Mt. Diablo, upper end of South Road, 7 Mar 1931, *Bowerman 553* (UC); between Antioch and Marsh Creek, 16 Jun 1907, *Brandegee* s.n. (POM, UC); Antioch, 7 Apr 1895, *Burtt Davy 985* (JEPS, UC); San Leandro, 21 Jun 1915, *Eastwood 4728* (CAS); Antioch, sand hills to the east, 22 m, 16 Apr 1908, *Heller 8889* (DS, E, GH, MO, US); hills east of St Mary’s College, 14 May 1933, *Howell 11291* (A, CAS, POM); 1 mi SE of Oakley, 2 Nov 1933, *Keck 2619* (CAS, DS, POM, UC, US); Marsh Creek Canyon, 31 Mar 1929, *Raven* s.n. (POM); Mt. Wanda, John Muir National Historic Site, 13 Mar 2003, *Smick GAS-068* (JEPS); N of Briones Reservoir, ca. 1.8 km S of Bear Creek Road on Hampton Road in Hampton Road Natural Reserve, 180 m, 22 Mar 1974, *Yorks 118* (JEPS); **Del Norte County**: At Bear Basin Heliport, Six Rivers National Forest, eastern Del Norte County, headwaters of the South Siskiyou Fork of the Smith River, 1463 m, 11 Jul 1964, *van Deventer 1050* (JEPS); **El Dorado County**: At Old Twin ridges, 500 yards above road, ca 44 mi E of Placerville along Hwy 50, 1905 m, 21 Jul 1934, *Belshaw 120* (UC); Along State Hwy 89, 3 miles south of Meyers, 1996 m, 13 Jun 1962, *Breedlove 3554* (CAS); Echo Summit, 2286 m, 1 Jun 1947, *Copeland* s.n. (UC); Aukum, near Mt Aukum, 10 Jun 1903, *Gross 209* (DS); Lake Tahoe region, Eagle Lake Trail, near Emerald Bay, 2134 m, 19 Jun 1925, *Howell 1173* (CAS); El Dorado National Forest, 2 mi SW of Georgetown Junction, Pyramid Pk Quadrangle, 1768 m, 20 Jul 1934, *Johannsen 421* (UC); Placerville, 13 Apr 1939, *Muenscher & Muenscher 15033* (A); Lake Tahoe, above Fallen Leaf Lake, 1890 m, 25 Jun 1958, *Myrick 53* (CAS); Highway 50, west slope, at 42 miles campground, 1768 m, 15 Jul 1956, *Pawek 189* (DS); Institude of Forest Genetics, At western edge of grounds of Institude of Forest Genetics, 792 m, 21 Apr 1943, *Robbins 1017* (CAS, UC); Echo Summit, on old Meyers Grade at hairpin curve below California Alpine Club Lodge, 2210 m, 19 Jul 1971, *Smith 3058* (JEPS); Echo Lake, N ridge of Echo Lake, about half way between lower boat landing and upper dock, 2347 m, 9 Jul 1971, *Smith 3059* (JEPS); trail from Glen Alpine Springs to Lake Gilmore, 2378 m, 20 Jul 1972, *Smith 3315* (JEPS); **Fresno County**: Huntington Lake, 3/4 mile E of Cedar Crest, 30 Jun 1949, *Beane 1495* (DS); Cedarbrook area, 2 1/2 miles NW of Pinehurst on Stony Flat Road, 1310 m, 10 May 1958, *Botkin* s.n. (RSA); Sierra National Forest, NW slope of Black Mountain, Kaiser Quadrangle, 853 m, 6 Jun 1935, *Bullard 40* (UC); Huntington Lake, Jun, *Clark* s.n. (RSA); High Sierras, Jackass Meadows, South Fork San Joaquin River, 2134 m, 10 Jul 1920, *Ferguson 450* (JEPS); Pine Ridge, 1524 m, 15 Jun 1900, *Hall & Chandler 93* (DS, DS, E, K, UC); Region of Dinkey Creek, Sierra Nevada Mountains, 1615 m, 25 Jun 1900, *Hall & Chandler 355* (DS, POM, UC); North Fork of Kings River, 1951 m, Jul 1900, *Hall & Chandler 448* (DS, MO, UC, US); Sierra Nevada, Simpson Meadow, Middle Fork of the Kings River, 1829 m, 25 Jul 1958, *Howell 33912* (CAS); Zapato Chino Canyon, 670 m, 12 Apr 1930, *Jepson 15411* (JEPS); Temperance Flat, S side San Joaquin R, 167 m, 7 May 1955, *Mallory 569* (RSA); Panoche Creek, Bottom down creek from the road crossing east of Panoche Valley, 304 m, 21 Mar 1932, *Quibell 2070* (POM); new road around N side of Hughes Mt, NW of Pine Flat Dam quarter mile or more down W from saddle N of mountain, 304 m, 24 Mar 1953, *Quibell & Quibell 1725* (CAS, RSA); Convict Road between General Grant National Park and the Canyon of the South Fork of the Kings River, 1676 m, 17 Apr 1934, *Quick & Quick 1204* (UC); Florence Lake, 2225 m, 5 Jul 1952, *Raven 4244* (CAS); 1 mile west of Sand Creek and Dunlap Roads, 609 m, 7 May 1969, *Shannon* s.n. (CAS); Sierra National Forest, 5 miles from Auberry toward Pine Ridge, 1128 m, 5 Jun 1965, *Thorne & Blake 34808* (RSA); Hillside 12-13 miles north of Coalinga along road to Hernandes, 9 Apr 1950, *Wiggins 12336* (CAS, RSA); Watts Valley, N end of Watts Valley, 609 m, 21 Apr 1933, *Winblad* s.n. (UC); Sierra Nevada, W slope. 1 mile below Miramonte on road to Dunlap, 853 m, 16 May 1933, *Wolf 4735* (A, CAS, RSA); Sequoia National Forest, Ca. 82 km E of Fresno, 5.5 km W-NW of Boyden Cave, Redwood Creek at Hwy 180, 1015 m, 27 Apr 1996, *York 623* (RSA); **Glenn County**: On the west side of Black Diamond Road, about 2 1/2 miles west of the paved road, about 4 3/4 miles (by road); from Stonyford, 749 m, 15 Jun 2006, *Ahart 12747* (CHSC, JEPS, RSA, SBBG); Black Butte, 16 Jul 1944, *Howell 19818* (CAS, US); SW end of Keller Lake, 1981 m, 7 Jul 1968, *Stebbins et al. 2570* (CAS); St. John Mountain, Along the jeep road ca. 0.4 miles below the summit, 16 Jun 1992, *Taylor 12812* (JEPS); Mendocino National Forest, Keller Lake, SW slope of Black Butte. NE side of the lake, 1707 m, 11 Jul 1982, *Wheeler & Smith 3195* (CAS); **Humboldt County**: along Friday Ridge Road, about 3 miles NE of its Jct. with Titlow Hill Road, 1067 m, 1 Jun 1965, *Anderson 3778* (RSA); Weott Ranger Station, Mt. Range Seaward N Coast, S Fork Eel River, 60 m, 12 Jul 1928, *Benson 463* (POM); Bull Creek Region, South Fork Eel River. Bull Creek road to mouth of Bull Creek, and up Bull Creek, 2 Jun 1934, *Constance 788* (JEPS); Camp Baxter, trail to Dardnells, 20 Jul 1930, *Jussel* s.n. (CAS); Near Hupa, 7 Jun 1901, *Manning 88* (UC); Carlotta, Jun 1915, *Parsons* s.n. (CAS); Orleans Mt., 1219 m, 31 Jul 1948, *Pollard* s.n. (CAS); Dinsmore’s Ranch, In Valley of Van Duzen River, opposite Buck Mountain, 762 m, 20 Jun 1913, *Tracy 4219* (UC); Bear River, 10 mi up stream from the mouth, 152 m, 20 Jun 1937, *Tracy 15340* (JEPS, UC); Carlotta, By old Strong’s District School House, 5 mi E of town, 60 m, 8 May 1949, *Tracy 18293* (UC); Briceland, 18 Jun 1950, *Tracy 18851* (RSA, UC); **Inyo County**: Pinyon Creek, A tributary of Independence Creek, E slope of Sierra Nevada, 1920 m, 16 Jun 1942, *Alexander & Kellogg 2980* (DS, GH, UC); Owens Valley, Sierra Nevada, 1493 m, 22 May 1973, *DeDecker 3212* (CAS, RSA, RSA); Shepherd Canyon, Owens Valley drainage, below Mahogany Flat. Sierra Nevada, 2682 m, 27 Jul 1979, *DeDecker 4877* (RSA, UC); Panamint Mountains, Thorndike’s Ranch, Upper Wildrose Canyon, 2286 m, 7 Jul 1937, *Epling* s.n. (RSA); Junction Nelson and Panamint Range near Jackass spring, 1829 m, 2 Jun 1940, *Jaeger* s.n. (MO); 8 miles up Mt Whitney Trail from Lone Pine, near crossing of Lone Pine Creek, 2103 m, 10 Jun 1935, *Kimber 46* (CAS); Thorndyke’s, Wild Rose Canyon, Panamint Mountains, 2134 m, 7 Jul 1937, *Munz 14836* (GH, MO, POM); Summit Creek, Trail from Olancha Pass, east slope of Sierra Nevada, 2774 m, 12 Jul 1950, *Munz 14960* (RSA); Death Valley National Monument, Thorndyke’s, Telescope Peak Quadrangle, 2286 m, 1 Aug 1938, *Shanteau 21* (UC); **Kern County**: Along State Hwy 166, 1 mi to W of summit of grade and near junction of Carriso Plains road, 26 Apr 1951, *Bacigalupi & Hawkes 3292* (JEPS); Tehachapi, *Brandegee* s.n. (DS, POM, UC); Banducci Rd, 9 miles W of Tehachapi, 1310 m, 4 Jul 1963, *Breedlove 5470* (CAS); Lebec, N side of Tejon Pass, 1000 m, 20 May 1980, *Davis & Lightowlers 67039* (E); S side of Rte. 58 around 0.1 mile E of the Sna Luis Obispo Co. line, about 6.4 mi W of the intersection with Reward Road and about 12.35 miles W of intersection with Rte. 33 S of McKittrick, 835 m, 29 Mar 2005, *Goldman 3174* (GH); Vicinity of Old Fort Tejon, 975 m, 16 Jun 1905, *Hall 6266* (E, UC, US); Tehachapi Mountains, Keene Station, 1 Apr 1931, *Hastings & Darland* s.n. (B, LE, MO, RSA, S, UC); Girard Station, Tehachapi Mountains, 18 Apr 1905, *Heller 7706* (E, GH, MO, US); Fort Tejon State Park, Above fort, 1067 m, 14 May 2002, *Moe 2326* (RSA); 19 miles above Weldon on road to Walker Pass, 1371 m, 21 May 1949, *Munz 13359* (CAS, RSA); Piute Mts, Erskine Creek, 1219 m, 1 Jun 1897, *Purpus 5053* (E, GH, K, MO, UC, US); Tremblor Range, 13.7 miles from McKittrick on road to Pozo (San Luis Obispo Co.), 13 Jun 1951, *Raven 2994* (CAS); 6 mi E of Mouth of Kern River Canyon, 457 m, 12 May 1940, *Rose 40353* (CAS, MO); Tejon Ranch, Grapevine Creek Canyon, section of creek SW of I-5, below Ft. Tejon St. Hist. Park, 1.25 mi due S of Grapevine Peak, 838 m, 18 May 1999, *Sanders et al. 22757* (RSA); Bitterwater Valley, at mouth of Cedar Canyon. Lowest limits of Upper Sonoran, 403 m, 3 Mar 1954, *Twisselmann 855* (CAS); Mt. Abel, 2.6 miles from the summit, San Emigdio Range, 2271 m, 10 Aug 1955, *Twisselmann 2322* (CAS); Bear Mountain, South slope of mountain. Tehachapi Mountains, 1676 m, 21 Jun 1964, *Twisselmann 9626* (CAS); Piute Mountains, Haight Canyon, 3.8 miles E of Havilah, 1432 m, 10 May 1966, *Twisselmann 12132* (CAS); Kern Plateau, Ridge between Little Cannell Meadow and Fay Creek, 2317 m, 16 Jul 1968, *Twisselmann & McMillan 14585* (CAS, GH, RSA); near Bakersfield, 10 Apr 1938, *Winblad* s.n. (CAS); **Kings County**: Tar Canyon, foothills W of Avenal, 23 Mar 1940, *Hoover 4281* (UC); **Lake County**: Bartlett Mt. Grade, 1 mile from Clear Lake, 5 May 1928, *Abrams 12380* (DS, POM); just E. of Clear lake on road to Mineral Springs, 1500 m, 9 May 1952, *Balls 8772* (BM, S); just out of Clear Lake Park on road to Mineral Springs, 9 May 1952, *Balls & Lenz 17137* (RSA); Soda Bay, Middle N Coast Mt Range, Clear Lake, 457 m, 26 May 1928, *Benson 577* (POM); Lower Lake, 2 mi N of Lower Lake, near Cache Creek bridge, 9 Jun 1938, *Jepson 18910* (JEPS); 3 miles east of Bartlett Springs, 7 May 1928, *Kindale 4943* (DS); Long Ridge trail to Snow Mt, 1524 m, 24 Jul 1956, *Munz 22300* (RSA); 1 mi from Clear Lake on the Bartlett Springs Grade, 5 May 1928, *Wolf 1950* (DS, RSA); 3 mi E of Bartlett Springs on the road to Williams, 6 May 1928, *Wolf 2051* (RSA, US); **Los Angeles County**: Liebre Mountains, Oakgrove Canyon, 1067 m, 19 Jun 1908, *Abrams & McGregor 336* (DS, E, GH, US); Claremont, 16 Mar 1903, *Baker 4059* (CAS, DS, GH, K, MO, POM, UC); San Gabriel Mountains, Cold Spring, 2.5 miles southwest of Camp Baldy on the Glendora Road, 1371 m, 5 Jun 1945, *Benson 11759* (POM); Liebre Mountains, Knapp Ranch area at upper end of Castaic Creek drainage in broad alluvial valley at head of Cienaga Canyon, south of Liebre Mountain, 914 m, 31 Mar 1997, *Boyd & Raz* (RSA); Camp at Cahuenga Pass, Feb 1861, *Brewer 189* (K, MO, UC, UC, US); Lower Hungry Valley, S side of Hungry Valley Road, ca 2.7 air km NW of confluence of Gorman Creek and Hungry Valley drainage, 10 air km SSE of Gorman, 922 m, 29 Apr 1989, *Buck & Palmer 1238* (JEPS); San Gabriel Mountains, Big Tujunga Canyon, ca 5 miles NE of Sunland on road 2N78, small side canyon, 685 m, 24 Jun 1975, *Davidson & Gustafson 2877* (RSA); San Gabriel Mountains, Angeles National Forest Glendora Ridge near Baldy Camp along forest service road above ridge road, 29 Apr 1984, *Elías 8332* (RSA); Ca 1 mi SE of Newhall in W part of Whitney Canyon just NE of junction of San Fernando Road and State Highway 14, within 200 ft of Hwy 14, 472 m, 15 Apr 2000, *Henrickson 20711* (RSA); Elizabeth Lake, slope 3 miles E of Elizabeth Lake, 3 May 1931, *Hoffmann 342* (CAS); Los Angeles Basin region, Palos Verdes Hills, 28 Mar 1928, *Kirtland 14754* (RSA); Santa Monica Mountains, Temescal Canyon, 1 Apr 1933, *Lloyd* s.n. (MO, MO, RSA, UC, US); Santa Monica Mountains, Topanga Canyon, 152 m, 17 May 1920, *Munz & Harwood 3980* (POM); Antelope Valley, Near Elizabeth Lake, Jun 1887, *Parish 1888* (DS, MO, UC); San Gabriel Mountains, along south bank of Rock Creek, north slope of San Gabriel Mountains, 1295 m, 15 Jun 1919, *Peirson 1082* (POM, RSA); San Gabriel Mountains, Yerba Buena Ridge, Angeles National Forest, Tujunga Ranger District, 1048 m, 14 May 1990, *Ross & Boyd 2467* (BM, MO, RSA); San Gabriel Mountains, Big Pines Hwy, mid-way between Caldwell Lake and Big Rock Springs, on the NE side of the knoll behind which Shoemaker Canyon turns to the NW, 1402 m, 29 May 1990, *Ross & Boyd 2673* (RSA); Santa Monica Mountains, Triunfo Pass W side of Yerba Buena Road, ca. 360 m NNW of intersection with Mulholland Highway, 487 m, 22 Mar 1992, *Ross & Bell 6049* (K, MO, RSA, UC); San Gabriel Mountains, upper Winter Creek Trail between Chantry Flat and Hoegee Camp (on Winter Creek), on the W side of Santa Anita Canyon, 2560 m, 1 Jun 1993, *Ross 7352* (CAS, F, RSA); Liebre Mountains, Warm Springs Canyon: canyon bottom and adjacent northerly slopes at the confluence of a blueline drainage NNE off the face of Warm Springs Mtn. ca 1250 m WNW from the canyon’s effluence into Elizabeth Lake Canyon, 667 m, 10 May 1994, *Ross & Boyd 7718* (CAS, RSA); Santa Clara River watershed, Newhall Ranch, ca. 0.4 north of Hwy 126, west of Castaic Creek and north of its confluence with the Santa Clara River, 0.6 mi. south of Commerce Center Dr. along Franklin Parkway, vicinity of large water tank, 305 m, 14 Apr 2003, *Sanders et al. 25981* (RSA); San Gabriel Mountains, 1 mile E of Georges Gap on Angeles Crest Highway (Highway 2), 1219 m, 1 May 1992, *Taylor 12495* (JEPS); San Gabriel Mountains, Angeles National Forest, Pinyon Flats 1.6 miles below Alder Saddle along dry branch of S fork of Little Rock Creek, 1585 m, 30 Jun 1971, *Thorne et al. 40782* (CAS, GH, RSA); Mojave Desert, Near Victorville Area, 18 Apr 1964, *Wallace* s.n. (RSA); UCLA Campus, West Los Angeles, 137 m, 20 Feb 1932, *Wheeler 446* (DS); Antelope Valley, Between Lancaster and Highway 99, 4 Jun 1941, *Winblad* s.n. (CAS); Los Angeles Basin region, W Covina. East on I-10 between Barranca and Grand off ramps, 27 Feb 1981, *Zuck 65* (RSA); **Madera County**: Devils Postpile National Monument, UTM Coordinates: 315497 4165036, 2429 m, 22 Jun 2001, *Arnett 8088* (JEPS); San Joaquin Experiment Range, 320 m, 30 Apr 1935, *Biswell 9* (UC, US); near Granite Springs, 3 May 1938, *Eastwood & Howell 5348* (CAS); Sierra Nevada, The Niche, East Fork of Granite Creek, 2439 m, 17 Aug 1958, *Howell 34573* (CAS); Agnew Meadows to Lake Olane, 2682 m, 1 Jul 1951, *Raven 3223* (CAS); San Joaquin River drainage, Along the Shadow Lake Trail, about 1 mi from Agnew Meadow . Sec 15 T3S R26E, 2469 m, 18 Jul 1962, *Reveal & Reveal 430* (UC); **Marin County:** near Drakes Estuary, 19 Apr 1927, *Abrams 11625* (DS); Inverness Ridge, 5 miles W of Pierce Point Rd, 30 Mar 1963, *Bell 17480* (MO); Mount Tamalpais, Blithedale Canyon, 26 Feb 1900, *Chandler* s.n. (UC); Lagunitas, 5 Jun 1909, *Hall 8501* (DS, UC); Lagunitas Creek Canyon, 12 May 1940, *Howell* s.n. (CAS); Mill Valley, Blythedale Canyon, 23 Apr 1939, *Howell 14606* (CAS); Deer Park, Near Fairfax, 13 May 1945, *Howell 20861* (CAS); Inverness, 13 Feb 1900, *Jepson 21257* (JEPS); Ross Valley, 5 Apr 1892, *Jepson 21259* (JEPS); Point Reyes Peninsula, Mar 1960, *Leary & Sherfey* s.n. (CAS); Mt. Tamalpais, trail to Kentfield, 19 Jun 1922, *McMinn 236* (DS); on the Balins Road above Fairfax, 182 m, 13 May 1950, *Raven 2087* (GH); Tomales Bay, Point Julia, 5 Aug 1932, *Schreiber 753* (UC); near confluence of Lagunitas and Nicasio Creeks, 30 m, 21 Apr 1965, *Thorne 34415* (RSA); Mt. Tamalpais, Muir Woods RR Track, 2 May 1909, *Zeile* s.n. (CAS); **Mariposa County**: Yosemite National Park, New Bridal Veil Falls, Yosemite Valley, 1219 m, 12 Jul 1911, *Abrams 4676* (DS, GH); Pipeline near Mariposa, 685 m, 19 Apr 1959, *Ballantyne 263* (CAS); Sierra Nevada, 3 mi NW of Coulterville, Sierra Nevada foothills, 701 m, 25 Apr 1937, *Crum 1912a* (UC); Hell Hollow, 1.1 mi N of Bear Valley (town); on Hwy 49, headwaters of Hell Hollow, 670 m, 23 Apr 1989, *Ertteret al. 8326* (RSA, UC); Yosemite National Park, Little Yosemite Valley, 1890 m, 7 Jul 1911, *Hall 9064* (UC); Sierra Nevada, Mt. Bullion, 640 m, 19 May 1954, *Howell 29911* (CAS); Yosemite Valley, along trail in vicinity of Nevada Falls, 1829 m, 1 Jun 1937, *Lee* s.n. (UC); 3 mi above Coulterville, 15 May 1936, *Mason 11111* (UC); 1 mi N of Mt. Bullion, 24 Apr 1954, *Raven 6739* (CAS); 3/4 mi S of Bridgeport, 518 m, 4 May 1935, *Schlobohm 106* (UC); **Mendocino County**: Russian River, 3 mi N of Ukiah, 21 Jun 1922, *Abrams 8109* (DS, POM); Covelo-Willows Highway, 3 mi W of Summit, 10 Jul 1949, *Baker 12126* (UC); Round Valley, 440 m, 20 May 1898, *Chesnut 69* (US); 6.5 miles W of Oris Hot Springs, 23 May 1936, *Eastwood & Howell* (CAS); Mendocino National Forest, Castle Peak to Middle Eel, Ace Bean Camp, Ace Bean Flat, 25 Jul 1897, *Jepson 21260* (JEPS); Mt. Sanhedrin, Summit, lookout tower, 1886 m, 26 Jul 1981, *Smith 7133* (CAS); Redwood Valley, 6 mi N of Capella, 213 m, 3 Apr 1941, *Tracy 16824* (UC); Mendocino National Forest, Middle Fork Eel River near Salmon River, about 4 mi SE of Round Valley, 304 m, 1 May 1980, *Wheeler 1565* (CAS); Mendocino National Forest, 6 mi E of Middle Fork Eel River on the Mendocino Pass Road, 1036 m, 15 Jun 1981, *Wheeler 2403* (CAS); **Merced County**: Los Baños, 19 Mar 1927, *Eastwood 14110* (CAS); Pacheco Pass, E of summit on old road, 24 Apr 1969, *Hoover 11264* (UC); Twin Peaks, North Fork Los Banos Creek, Inner South Coast Ranges, 5 May 1940, *Mason 12276* (GH, UC); Pacheco Pass, San Luis Gonzaga Grant, 396 m, 2 Apr 1934, *Short & Johnson S 27* (UC); **Modoc County**: Little Hot Spring Valley, 8 Jun 1894, *Baker & Nutting* s.n. (DS, UC); 1.8 km WSW of Day on County Road 94, 1170 m, 23 Jun 1988, *Bartholomew & Anderson 4260* (CAS, RSA); **Mono County**: 1.4 mi. N. of Convict Lake on rd. from U.S. Hwy. 395, 2195 m, 8 Jul 1956, *Balls 10930* (BM, E, S); Sierra Nevada, Leevining Grade, 3.5 mi above camp ground, 2317 m, 29 Jun 1952, *Constance & Bacigalupi 3447* (UC); 1.4 mi N of Convict Lake, on road from U.S. Highway 395, 2195 m, 8 Jul 1956, *Everett & Balls, 21964* (CAS, RSA, UC); Robinson Creek, 12 mi SW of Bridgeport, 2134 m, 18 Jul 1940, *Munz 16106* (CAS, GH, POM, UC); Whiskey Creek, Jul 1938, *Noldeke* s.n. (CAS); Mono National Forest, near Horse Creek above Twin Lakes, N shoulder of Matterhorn Peak, 2399 m, 2 Aug 1945, *Wiggins & Rollins 501* (DS, GH, UC); Inyo National Forest, Road below Twin Lakes, Mammoth Lakes area, 2560 m, 29 Aug 1946, *Willard 108* (DS, RSA); **Monterey County**: Carmel, 17 Aug 1909, *Abrams 4271* (DS); between Bradley and San Ardo, 1 mi S of Salinas Bridge, 5 Apr 1924, *Abrams 10165* (DS, GH, POM, UC); Santa Lucia Mountains, 2 mi SE of Santa Lucia Memorial Camp by road, 10 Apr 1936, *Carter et al. 1073* (UC); Pacific Grove, Salinas Road about 5.5 mi from Pacific Grove, 27 Dec 1919, *Duncan* s.n. (DS); near Asilomar, 28 Jan 1935, *Eastwood & Howell 1923* (CAS, MO, RSA); Del Monte, Apr 1902, *Elmer 3552* (DS, E, GH, K, MO, POM, UC, US); Tassajara Hot Springs, 472 m, 26 Apr 1933, *Ferris 8307* (CAS, DS, GH, RSA, UC); Stone Canyon, 8 mi E of Salinas Valley, 22 Mar 1931, *Howell 5951* (CAS, DS, GH, POM, RSA); Gabilan Range, near H.A. Twisselmann Ranch on Johnson Road about 3 miles E of Gonzales in Salinas Valley, 29 May 1962, *Howitt 1322* (CAS, PGM); Santa Lucia Mountains, near fire tower, Chew’s Ridge, 8 mi N of Tassajara Hot Springs, 1493 m, 30 May 1937, *Mathias 1312* (GH, UC); near trail from Carmel to Monterey, 30 Dec 1909, *Randall* s.n. (DS, POM); Los Padres National Forest, 2.8 mi E of Leigh Ranch, 243 m, 16 Oct 1938, *Simontacchi 672* (UC); Cholame Valley, 0.75 mile W of the Cholame Ranch headquarters, 323 m, 21 Apr 1970, *Twisselmann 16382* (CAS); Toro Creek, 5 miles SSW Salinas, 45 m, 27 May 1944, *Wheeler 5957* (RSA); **Napa County**: Howell Mountains, E slope in the Howell Mountains, 5 mi E of Napa, 457 m, 27 Mar 1938, *Constance 2039* (GH, K, MO, S, UC); Nevada Creek, along Nevada Creek, about 5 mi S of Knoxville, 304 m, 15 May 1965, *Heckard & Bacigalupi . 1460* (JEPS); Zem-Zem, northeastern 28 Jul 1892, *Jepson 21261* (JEPS, US); State highway 128, 28 miles west of Winters, 4 Apr 1970, *MacFarlane 5* (MA); Hill 7 mi E of Napa, on the road to Monticello, 19 Apr 1937, *Osgood* s.n. (UC); Rector Creek, 121 m, 31 May 1952, *Raven 4159* (JEPS); 11.3 miles N of Pope Canyon Rd. on Berryessa-Knoxville Road, 231 m, 22 Apr 1980, *Ruygt 860* (JEPS); San Francisco Bay Region, S side of Mount Atlas, 609 m, 17 Mar 1963, *Sharsmith 5214* (UC); **Nevada County**: Soda Springs, 30 Jul 1881, *Jones* s.n. (POM); Donner Region, Rainbow Tavern, 1829 m, *Pollard* s.n. (CAS); Lake City, Lake City near Bloomfield. Tahoe Forest, 914 m, 30 Apr 1925, *Smith 1571* (CAS, JEPS); Sierra Nevada, Near Grouse Ridge Lookout, 5 miles N of Yuba Gap, 2347 m, 10 Jul 1962, *Stortz 839* (CAS); Sierra Nevada, Woodchuck Flat on Rattlesnake Creek about 2 miles N of Cisco Grove, 1951 m, 23 Jul 1964, *True 1611* (CAS); Relief Hill Road, 10 mi W of Washington, 1128 m, 12 May 1966, *True 2822* (CAS); **Orange County**: Gypsum Canyon, Santa Ana Mountains, S of Corona, 22 Feb 1929, *Crow 169* (RSA); Santa Ana Canyon, Santa Ana River bottom, 152 m, 24 Jul 1927, *Howell 2822* (CAS); Cleveland National Forest, State Hwy 74, 1 mi uphill from the 2000 ft marker, and to the left ca 1 mi on an old paved road, 640 m, 21 May 1992, *Pitzer & Hemstreet 1943* (RSA); vicinity of city of Brea, immediately N of Carbon Canyon Road and E of Placentia Avenue. ±1 mile (airline); S/SE of Tonner Canyon, 182 m, 13 Oct 1992, *White 959* (RSA); Santa Ana Mountains, above Modjeska Canyon, proposed water tank site, 425 m, 28 Mar 1997, *White 4831* (RSA); Trabuco Canyon, 1/2 mi below Trabuco Camp Ground, W slope Santa Ana Mountains, 609 m, 6 Mar 1931, *Wolf 1850* (DS, POM, RSA, UC); Modjeska Grade, 1 mi N of base of Modjeska Grade, W slope of Santa Ana Mountains, 304 m, 6 Mar 1931, *Wolf 1862* (DS, POM, RSA, UC, UCR); **Placer County**: Sierra Nevada, near Baxter’s Hotel, between Colfax and Gold Run, W side of Sierra Nevada, 8 Jun 1935, *Belshaw 1002* (UC); Sierra Nevada Mountains, Squaw Valley, 1920 m, 7 Jul 1915, *Brainerd & Baird 146* (JEPS); about 6 mi SE of Loomis, on the road from Folsom to Auburn, 25 Apr 1937, *Carter 1250* (UC); Donner Pass, W side of Donner Pass near Norden, 2134 m, 25 Jul 1943, *Howell 18806* (CAS, UC); near Tahoe City, 1899 m, 18 Jun 1935, *Lehenbauer* s.n. (UC); 5 mi above Auburn, 28 May 1926, *Mason 3269* (GH, UC); roadbank going into Alpine Meadows, 2073 m, 21 Jun 1972, *Smith 3283* (JEPS); Pacific Crest Trail between old and new Donner Summits, 6 Jul 1985, *Williams 85-63-4* (RSA); **Plumas County**: W side of the dirt road, W of South Fork of the Feather River, about 1/4 mi N of Little Grass Valley Reservoir, 4 1/2 air miles N of La Porte, 1585 m, 4 Jun 2001, *Ahart 8752* (CHSC, JEPS); on the E side of the poor road, about 1/2 mile S of the intersection of Lumpkin Ridge Road and the road to Tamarack Flat, ca 1 mile (air); NW of Little Grass Valley Reservoir dam, about 4 miles (air); NW of La Porte, 1796 m, 26 Jun 2002, *Ahart 9771* (CHSC, JEPS); Upper reaches of trail from Gray Eagle Valley to Long Lake, 7 Jul 1927, *Bacigalupi 1648* (DS); at the mouth of Chambers Creek, just above Tobin, North Fork of the Feather River, 640 m, 25 Apr 1974, *Dakan* s.n. (CAS); Plumas National Forest, 1.5 mi NW of Bucks Mountain, 1524 m, 6 Jun 1934, *Embree 84* (UC); Johnsville, 1585 m, 28 Jun 1951, *Howell 27644* (CAS); Gold Lake, slide just W of Gold Lake, 19 Jun 1924, *Mason 1066* (UC); **Riverside County**: Palomar Mountains, NW Palomar Mountains, Agua Tibia Wilderness Area, N face of Agua Tibia Mtn., Dorland Mtn. area, 805 m, 15 Mar 1995, *Banks & Boyd 75* (RSA, UCR); Santa Ana Mountains, Gavilan Hills region, eastern edge of the Gavilan Plateau at the head of “Santa Rosa Canyon” along Santa Rosa Road, 1/4 mile N of El Nido Road, 609 m, 29 Apr 1981, *Boyd* s.n. (RSA); Vail Lake Area, Temecula Creek Canyon below Vail Lake dam, 417 m, 18 Mar 1989, *Boyd et al. 2991* (RSA); Santa Ana Mountains, San Mateo Canyon Wilderness of the Cleveland National Forest. Lower half of Tenaja Cyn, especially about Fishermans Camp, 2 Feb 1992, *Boyd et al. 6658* (RSA); Banning, Southern Colorado Desert, 701 m, 21 Apr 1944, *Cooper 1140* (RSA); San Jacinto Mountains, Santa Rosa Mts Road from Hwy 74 to Santa Rosa Mt, ca. 2 mi W of junction to Sta Rosa Spgs Campground, 2088 m, 15 Jun 1978, *Davidson 7380* (RSA); White Post Turn on Rt 74, to the W side of the road and NW part of the turn, about 3.9 m W of the intersection with Rt 243, San Bernardino National Forest, 1088 m, 11 May 2005, *Goldman 3283* (K); Cleveland National Forest, Hwy 74 W of lower San Juan Picnic Area at pullout W side of Santa Ana Mountain Range, 495 m, 12 Mar 2005, *Gust & Nye 562* (MO); San Antonio Mountains, Lyttle Creek Canyon, 1219 m, 1 Jun 1900, *Hall 1427* (DS, E, MO, UC); Vicinity of Riverside, 457 m, 21 Apr 1902, *Hall 2929* (E, K, MO, POM, UC); Santa Ana Mountains, W of Lake Elsinore, 487 m, 29 Apr 1922, *Munz 5085* (POM); Anza Bench region, San Carlos Pass, near Anza, 1371 m, 21 May 1927, *Munz 10851* (POM); Cleveland National Forest, San Juan Loop Trail, ca. 4 mi W of El Cariso Village, just across the hwy from Ortega Parks Store, 548 m, 20 Mar 1988, *Pitzer & Misquez 669* (RSA); San Jacinto Mountains, 7.4 miles SE of Banning on road to Idyllwild, 17 May 1958, *Raven 12943* (CAS, GH, RSA); San Bernardino Mountains, Head of Banning (San Gorgonio River); Canyon close to the San Bernardino Co. line, 0.5 mi SE of Oak Glen Conservation Camp, 1402 m, 27 May 2004, *Salvato & Green 630* (RSA); Box Springs Mountains, S end of the range along Gernert Rd. (dirt road paralleling the mountain front), in valley draining to Box Spring Canyon, 457 m, 1 Mar 1997, *Sanders 19749* (RSA); Santa Rosa Plateau, Rancho California, 609 m, 6 Jun 1969, *Thorne & Lathrop 39334* (RSA, UC); San Jacinto Mountains, Idyllwild area, “Strawberry Fuelbreak” between Inspiration Point and Strawberry Creek, SW terminus of Double View Drive W to creek, 1402 m, 30 Jun 2001, *White et al. 8657* (RSA); Temescal Canyon, below and E of Horsethief Canyon immediately S of I-15 Freeway, 366 m, 4 Apr 2003, *White & Honer 8961* (RSA); Perris/Elsinore Basins, Temescal Valley, just N of the I-15 Freeway and E of Indian Truck Trail, S and W of Lee Lake, 335 m, 6 Apr 2004, *White & Honer 10048* (RSA); Santa Ana Mountains, along Ortega Highway, San Juan Canyon, 5/10 mi E of Orange County Line, 365 m, 17 Jun 1936, *Wolf 7953* (DS, GH, RSA, UC); **San Benito County**: Pinnacles National Monument, 4 mi N of Pinnacles Lodge, 4 Apr 1939, *Baker 9271* (POM, UC); Lorenzo Vasquez Canyon, West Gate Ranch, mouth Lorenzo Vasquez Canyon, 8.0 mi from junction with Highway 25 on Coalinga Road, 304 m, 11 May 1982, *Crosby & Morin 14380* (CHSC, JEPS, MO); Upper San Benito River, 4 mi E of Hernandez, 12 May 1947, *Hoffman 1591* (UC); Tres Pinos, 3 miles S of Tres Pinos, 22 Apr 1933, *Howell 11033* (CAS, POM); Pinnacles National Monument, 425 m, 7 Apr 1929, *Mexia 2366* (CAS, POM, UC, US); Panoche Road, off Route J1, ca. 10 km east of junction with Route 25, 300 m, 11 Apr 2000, *Stone &Bodine 2946* (LE, MO); Bitterwater, Hollister Road, 0.3 mile N of Bitterwater, 442 m, 4 Oct 1966, *Twisselmann 12888* (CAS, RSA); Griswold Creek, 5 miles S of Panoche, on New Idria Road, 609 m, 7 Apr 1928, *Wolf 1704 A* (RSA, RSA, US); New Idria, 762 m, 8 Apr 1928, *Wolf 1758* (RSA); **San Bernardino County**: Cajon Pass, N of Cajon Pass, 1189 m, 5 Jun 1941, *Alexander & Kellogg 2297* (UC); San Bernardino Mountains, Mountain Home Creek Canyon, 1356 m, 16 May 1947, *Benson 12426* (POM); San Gabriel Mountains, Lytle Creek Canyon, 701 m, 4 Mar 1934, *Edge 18928* (RSA); San Gabriel Mountains, Head of Lone Pine Canyon, N-slope, 2000 m, 8 Jun 1932, *Fosberg 8571* (POM); San Bernardino Mountains, Sugarloaf, S-slope, 2926 m, 11 Jul 1906, *Grinnell 213* (CAS, US); San Bernardino Mountains, ca. 20 air miles W of San Bernardino, 3 miles NW of Angeles Oaks, near junction of Deer Creek and Santa Ana River near Filaree Flat, 1158 m, 18 May 1985, *Henrickson 19982* (RSA); San Gabriel Mountains, Horse Canyon/Circle Mtn. Rehab Project, in wide drainage W of Circle Mountain, 1798 m, 18 Jul 1994, *Mistretta 1432* (RSA); San Bernardino Mountains, Bear Valley, 1981 m, 25 Jun 1894, *Parish 3382* (MO, US); San Bernardino Mountains, Waterman Canyon, 685 m, 20 May 1908, *Parish 11759* (CAS, MO, UC); San Bernardino Mountains, Holcomb Creek, tributary to Santa Ana River, 2591 m, 23 Jun 1922, *Peirson 4062* (RSA); San Gabriel Mountains, Angeles National Forest, along Glendora Mountain Road, 27.4 km N of the intersection with Boulder Springs Drive (just S of the entrance to the National Forest), 17 May 1993, *Prather & Hempel 1356* (MO, RSA); San Gabriel Mountains, Dirt extension of Almond Ave. (W), ca. 1.0 mile W of Cucamonga Canyon, at SE corner of debris basin. Just E of Frankish Point, 731 m, 23 Apr 1994, *Swinney 2784* (RSA); San Bernardino National Forest, San Gabriel Mountains, divide between Lone Pine and Swarthout Valleys, just E of Wrightwood, 1852 m, 26 Jun 1969, *Thorne & Tilforth 38189* (RSA, UC); San Gabriel Mountains, San Bernardino National Forest: near San Sevaine Cow Camp, 1432 m, 7 Jul 1971, *Thorne et al. 40919* (RSA); San Bernardino National Forest, Cucamonga Canyon along truck trail, San Gabriel Mountains, 792 m, 9 Jun 1971, *Thorne & Thorne 40965* (UC); Mouth of Devil Canyon, 4 mi N of San Bernardino, 731 m, 13 Mar 1935, *True 84* (UC); San Bernardino Mountains, Jeep Trail to Aspen grove off Fish Creek Fork on Coon Creek-Fish Cr. Rd., off Hart Bar Campground Rd., ca. 5 miles off Calif. Hwy 38, San Bernardino National Forest. San Gorgonio Wilderness, Fish Creek to Santa Ana River drainage area, 2225 m, 7 Oct 1968, *Wilder 4435* (POM); Mojave Desert, 3 mi E of summit of Cajon Pass on road to Victorville, 20 May 1926, *Wolf 13* (RSA); **San Diego County**: near Bernardo, 1 May 1903, *Abrams 3362* (A, BM, DS, E, GH, K, LE, MO, POM, US); Witch Creek, 1 May 1894, *Alderson* s.n. (DS, GH, UC); Cleveland National Forest, NW Palomar Mountains, Agua Tibia Wilderness Area on the saddle between the two peaks of Agua Tibia Mountain. In large oak grove at the Dripping Springs Trail and Palomar-Magee Trail junction, 1341 m, 1 Jun 1995, *Banks & Boyd 499* (RSA); 3.7 mi E of Otay Dam along Janul Dulzura Creek on Telegraph Canyon Road, 175 m, 10 Mar 1962, *Breedlove 1821* (DS); Cuyamaca-Laguna Mountains, Barrett, 304 m, 17 Mar 1949, *Cooper 3128* (RSA); Cuyamaca-Laguna Mountains, Potrero, 1067 m, 2 Jun 1949, *Cooper 4057* (RSA); Escondido, 23 mi N of Escondido, 304 m, 25 Apr 1964, *Cronquist 9886* (GH); San Ysidro Mountains, SE side of Otay Mtn, western Marron Valley, S of BM 838, ca. 1/2 km N of Mexican border, slopes above Tijuana River, 183 m, 3 May 2003, *Elvin 2606* (IRVC, RSA, UCR); Oriflamme Canyon, 22 Jun 1932, *Epling et al.* s.n. (BM, L, LE); Cuyamaca-Laguna Mountains region, SW side of Viejas Mountain, at base of mountain, just off West Boundary Truck Trail, just at the end of Anderson Road, SW side of the Cuyamaca Mountains, 795 m, 5 Apr 2005, *Gross et al. 1832* (RSA); 10 mi SE of Temecula, on road to Pala, 16 Mar 1962, *Hitchcock & Muhlick 22170* (DS, F, MO, UC); San Diego, 3 Apr 1882, *Jones* s.n. (POM); Anza Bench region, Near Warner’s Ranch, 1036 m, 17 May 1925, *Keck & McCully 52* (POM); Torrey Pines, 12 Dec 1919, *Millspaugh 4487* (F); Chollas Valley, 20 Mar 1883, *Orcutt, C.R*., *s.n.* (F); Encinitas, 0.5 mile N of Encinitas, 26 Mar 1923, *Peirson 3380* (POM, RSA); South Coastal Plain region, 2 miles E of Solana Beach, 26 Jun 1941, *Ramsey 813* (POM, RSA); Crestridge Ecological Reserve, W of La Cresta between Alpine and El Cajon, east of Rios Canyon, on N-facing slope, 850 m, 1 May 2003, *Rebman & Gregory 8736* (UC); Laguna Mountains, Lucky 5 Ranch, southern portion of new acquisition by California State Parks: E side of Sunrise Hwy (S-1); between Cuyamaca Lake and Mount Laguna, 1675 m, 7 Oct 2003, *Rebman & Gregory 9571* (RSA, UC); San Diego, vicinity of San Diego, 60 m, 5 Mar 1916, *Spencer 148* (GH, K, UC, US); Camp Pendleton Marine Corps Base, Canyon off Ash Road, 30 m, 7 Apr 2000, *Stone & Bodine 2887* (MO); Pine Valley, Corte Madera Ranch, 1219 m, 9 May 1993, *van der Werff 12928* (MO); 5.5 mi S of Ramona on road to Lakeside, 6 May 1927, *Wiggins 2476* (DS, UC); Point Loma, 1/4 mi N of Old Spanish Lighthouse, 60 m, 26 May 1931, *Wolf 2082* (DS, POM, RSA, UC); near Banner, 853 m, 8 Apr 1936, *Yates 5474* (UC); **San Francisco County**: San Francisco, 1853, *Bigelow* s.n. (GH, K); San Francisco, Sep 1865, *Bolander* s.n. (LE, MO); San Francisco, East of Lands End, 27 Apr 1918, *Collins* s.n. (GH); Lone Mountain, 3 Jul 1880, *Engelmann* s.n. (MO); San Francisco, 23 Mar 1869, *Kellogg & Harford 717* (BM, MO, US); San Francisco, Presidio, nothern slope of Lobos Creek Gully between 16th Avenue and Lincoln Boulevard, 9 Nov 1956, *Rübtzoff 3058* (RSA); South San Francisco Airport, 20 Apr 1930, *Sumner 101* (US); **San Joaquin County**: Hospital Canyon, 18 Apr 1938, *Eastwood & Howell 5074A* (A, CAS, UC); Holt, 28 Sep 1930, *Howell 5512* (CAS, GH, POM); Sunol State Park, S of Tracy, 18 Mar 1973, *Kasapligil 4546* (UC); Live Oaks, Mar 1877, *Rattan* s.n. (DS); Hospital Canyon, 5 Apr 1930, *Stanford 1220* (CAS, MO); **San Luis Obispo County**: Los Padres National Forest, La Panza Public Camp, E base of La Panza Range, 655 m, 25 May 1955, *Bacigalupi et al. 5247* (JEPS); Temblor Mountains, Upper Cuyama Valley, San Maria River Watershed, Kern Co. line, 609 m, 9 May 1932, *Benson 3573* (DS, POM, UC); 2 mi SSE of mouth of Osos Creek, 45 m, 14 Jan 1936, *Bolt 532* (UC); Oso Flaco Lake, 5 miles N of Guadelupe, 7 Aug 1962, *Breedlove 4089* (DS); Morro Bay, 5 miles E, 1 Apr 1937, *Cantelow 2071* (CAS); La Panza Mountains, 8 May 1936, *Eastwood & Howell 2316* (CAS, US); Paso Robles, 4 May 1926, *Eastwood 13836* (A, CAS); Templeton, hills about 4 mi SE of Templeton, 304 m, 29 May 1939, *Ferris 9760* (CAS, DS, POM, UC); Luquero Crossing, hills N, Santa Lucia Mountains, 3 Jun 1956, *Hardham 853* (CAS, RSA); Peachy Canyon, Santa Lucia Mountains, 26 Apr 1958, *Hardham 3164* (CAS, JEPS); Outer South Coast Ranges region headwaters of Las Tablas Creek, 19 May 1959, *Hardham 4564* (CAS, RSA); Swift Ranch, SE of Los Osos, starting ca. 2 mi S of Los Osos Valley Road on Clark Valley Road, 107 m, 8 Mar 1997, *Helmkamp 1459* (CAS); Temblor Range, summit, NE of Simmler, 16 May 1953, *Hoover 8304* (CAS); Diablo Canyon, San Luis Range, 18 Feb 1967, *Hoover 10211* (CAS, UC); Camp Roberts, vicinity of Twin Bridges (over Nacimiento River), 16 May 2000, *Keil et al. 28704* (UC); Palo Prieta Canyon, 1 mi N and 8 mi E of Shandon, 25 May 1952, *McMillan 130* (CAS, UC); Paso Robles, 8 Apr 1926, *Munz 10129* (POM, UC); Nacimiento River, Boyscout Road, Camp Roberts and Camp San Luis Obispo, 23 May 1993, *Proctor & Young ROB-0493* (RSA); Morro Bay, S end between bay and ocean, 21 Apr 1963, *Thorne & Everett 31632* (RSA); Palo Prieta Canyon, 3.5 miles S of Cholame, 457 m, 31 Mar 1956, *Twisselmann 2602* (CAS); York Mountain, W slope, Santa Lucia Range, 289 m, 15 May 1956, *Twisselmann 2786* (CAS); Cuyama Valley, 1 mi w of Kern County line, upper end of Cuyama Valley, Santa Maria-Maricopa State Highway, 18 Nov 1932, *Wolf 4416* (A, RSA, UC); **San Mateo County**: Redwood City, Whipple Road, 30 m, 16 Mar 1941, *Arnaud* s.n. (CAS); King’s Mountain, W side, 15 Jan 1902, *Baker 234* (ECON, GH, K, MO, POM, UC); Corte Madero Creek, 30 May 1932, *Blackwelder 168* (MO); Redwood City, 3 miles westward, near intersection of Canada and Edgewood Roads, 18 Jan 1975, *Cahill 395* (DS); Jasper Ridge, Stanford University, Santa Cruz Mtns., San Francisco Bay watershed, 152 m, 21 Apr 1935, *Cohen 605* (POM); Los Trancos, 9 Apr 1932, *Demaree 8981* (MO); Glen Gloaming, near Pedro’s Creek, 16 Feb 1896, *Dudley* s.n. (DS, RSA); Jasper Ridge, Santa Cruz Peninsula, 11 Feb 1900, *Dutton* s.n. (DS); San Bruno Mountains, overlooking Daly City near San Francisco, 14 Apr 1967, *Gorelick* s.n. (RSA); Jasper Ridge, Santa Cruz Mountain peninsula, May 1907, *McGregor* s.n. (MO); Kings Mountain, about 6 mi W of Woodside, E slope of Kings Mountain, Santa Cruz Mountains, 16 Mar 1941, *Rollins 2944* (DS, GH, UC); Brisbane, about 1 mile W, SE slope of San Bruno hills, 30 Apr 1942, *Rollins 3001* (DS, GH, US); La Honda Grade, 0.5 mi E of base of grade, Santa Cruz Mountains, 21 Aug 1931, *Wolf 2334* (RSA); **Santa Barbara County**: Santa Cruz Island, vicinity of Pelican Bay, 26 Apr 1930, *Abrams & Wiggins 76* (CAS, GH, UC); Cañada Honda, 7 miles south of Surf, 5 m, 26 Apr 1951, *Balls & Balls 16351* (RSA); , Ballinger Canyon, ca. 0.5 mi E of Hwy. 399, 853 m, 19 Sep 1962, *Chandler 939* (CAS); Gaviota, adjacent to Santa Barbara, 1 May 1908, *Eastwood 52* (F, GH, K, MO, UC, US); Santa Maria, ca. 53 miles S on highway in western end of Cuyama Valley, 30 Mar 1935, *Ferris 9155* (DS); Santa Cruz Island, Prisoner’s Harbor, 5 m, 6 Mar 1932, *Fosberg 7570* (POM); Carpinteria, 30 m, 18 Apr 1932, *Fosberg 8002* (POM, RSA, UC); Midland School, Santa Ynez Valley, 7 Apr 1957, *Hardham 1628* (CAS); Sycamore Canyon, Santa Inez Mountains, 13 Apr 1921, *Jepson 9151* (JEPS); Purisima Hills, 182 m, 25 Apr 1927, *Jepson 11946* (JEPS); Santa Cruz Island, Alamos Canyon, SW of Peak 611, 0.75 miules inland from beach, 49 m, 12 Apr 1994, *Junak SC-3873* (RSA); Santa Rosa Island, east side of Black Mountain, 304 m, 9 Mar 1950, *Moran 3346* (DS, UC); Lompoc, canyon north of Lompoc, 9 Apr 1938, *Munz 15749* (DS, POM, US); Mission Canyon, Santa Inez Mtns. near Sta. Barbara, 17 Feb 1931, *Müller 2454* (L); Surf, 13 Apr 1938, *Peirson 12386* (CAS, RSA); Los Padres National Forest, Santa Ynez Mountains. Hwy 154 (San Marcos Pass Rd); at intersection with Painted Cave Rd, ca. 4 mi. below San Marcos Pass, 610 m, 13 May 1991, *Pitzer 1523* (RSA); Santa Rosa Island, Lobo Canyon, 122 m, 5 Apr 1960, *Raven et al. 14928* (RSA); Santa Cruz Island, on ridge south of Stanton Ranch, 26 Apr 1960, *Raven & Smith 15254* (RSA); Cuyama Valley, 30 mi. W of Maricopa, 610 m, 18 Mar 1936, *Rose 36061* (CAS, RSA, S); Santa Cruz Island, Fryes Harbor, 31 May 1908, *Sexauer 79* (BM, E, F, GH, K, LE, MO, POM, S, UC); Rattlesnake Creek, 610 m, 23 Feb 1970, *Shevock 20* (RSA); Tajiguas Canyon, above the planted orchards, south foothills of Santa Ynez Mts. near Refugio Pass, 13 Dec 1950, *Smith 2889* (RSA); Vandenburg Air Force Base, Southern Pacific RR right of way between San Antonio Creek and Santa Ynez River (ca. 10 mi), Casmalia, Surf, 24 May 2001, *White & Devries 8512* (RSA); San Raphael Mountain, Figueroa Mt. Road and gate ca. 2 mi. E of Midland Sch. along N-facing Hillside, Santa Ynez River drainage, 396 m, 28 Mar 1965, *Wilder 1708* (POM); Santa Cruz Island, ledge above cove about a 1/2 mile west of Pelican Bay, 7 Jul 1939, *Williams 38* (POM, UC); Lompoc, 3 miles north of Lompoc, 106 m, 14 Apr 1929, *Wolf 3537* (RSA); **Santa Clara County**: Stanford University, 3 May 1902, *Abrams 2390* (DS, MO, POM); Stanford University, 9 May 1902, *Baker 273* (B, C, CAS, DS, ECON, GH, K, LE, MO, POM, UC, UC, US); Los Gatos, 0.2 mile S on Hwy. 17 between Santa Cruz and Los Gatos, 121 m, 6 May 1952, *Balls & Lenz 17085* (RSA); Seeboy Ridge, 1/2 mi. below summit, Mt. Hamilton Range, 3 Apr 1936, *Carter & Sharsmith 1056* (UC); Loma Prieta, summit road, Gov’t Range, Santa Cruz Mountain Peninsula, 30 May 1893, *Dudley* s.n. (DS); ca. 12 miles E of San Jose along raod to Mt. Hamilton, 304 m, 28 Apr 1962, *Gentry 19696* (US); Pacheco Pass, on W side of Pass, 4 May 1936, *Jepson 17464* (JEPS); Los Francos Creek, Santa Cruz Mountains, 4 Apr 1906, *McMurphy 72* (DS, GH, MO, US); , Pacheco Pass, west of the crest along the Pacheco Pass highway, 274 m, 28 Mar 1929, *Quibell 993* (POM); Santa Clara County, Mount Hamilton, above Alum Rock Falls, on W slopes of Mount Hamilton, 365 m, 24 Mar 1935, *Sharsmith 1500* (UC); Canada Valley, near Gilroy, 29 Mar 1915, *Sheldon* s.n. (DS); Page Mill Road, 2.9 miles up in hills from intersection with Alameda de las Pulgas, 21 May 1949, *Thomas 554* (DS); Mayfield, 3 mi sw of Mayfield along Page Mill Road, 31 May 1939, *Wiggins 9216* (DS, LE, UC, US); Monte Bello Ridge, 548 m, 29 Apr 1936, *Yates 5526* (UC); **Santa Cruz County**: Hwy. #17, Santa Cruz to Los Gatos, 2 mi. S of Los Gatos, 6 May 1952, *Balls 8725* (BM, E, S); Glen Gloaming, near Petersen Creek, 16 Feb 1896, *Dudley* s.n. (DS); Año Nuevo Pines, 9 Apr 1933, *Howell 10979* (CAS); Santa Cruz, 22 Jun 1881, *Jones 2223* (BM, CAS, DS, GH, LE, POM, U, UC, US, US); along Pajaro River, just inside county line in SE corner of county Pajaro River, 30 m, 15 Apr 1950, *Thomas 1542* (DS, JEPS, RSA); Rancho del Oso, about 1 mile inland from coast, 8.5 miles NW of Davenport, 1 Oct 1950, *Thomas 2475* (DS, RSA); Saint Mary of the Palms, summer girls camp near Glenwood, 304 m, 23 Mar 1953, *Thomas 2876* (DS); Pajaro River, alogn river near bridge on San Juan Bautista-Watsonville Highway (#67), 91 m, 30 May 1953, *Thomas 3228* (DS); Lomita Ridge, 731 m, 9 Apr 1933, *Wieslander & Yates 298* (UC); **Shasta County**: Goose Valley, 26 May 1894, *Baker* s.n. (UC); Redding, 9 Apr 1911, *Blankinship* s.n. (JEPS); in a meadow about 3 miles northeast of Redding, 30 May 1905, *Heller 7899* (E); 2 mi w of Falls River Mills (below Pit Dam, Power Plant), 1341 m, 25 May 1940, *Hitchcock 6590* (DS, MO, POM, UC); Redding, 28 Apr 1911, *Jones 235* (GH); Ono, 21 May 1925, *Malmsten* s.n. (DS); east of Timbered Crater in lava bed area, 1051 m, 5 Jun 1989, *Schoolcraft 1898* (UC); **Sierra County**: Sierra City, Herrington’s, 1219 m, 12 Jul 1982, *Best 737* (CAS); Webber Lake, *Lemmon* s.n. (GH); Tahoe National Forest, 1.3 mi. west of Sierra City on Hwy 49, 1250 m, 29 May 1988, *Pitzer et al. 941* (RSA); Sand Pond, 2 mi from Highway 49 down Sardine Lake Road at Sand Pond, 20 m n of bath house, 1829 m, 27 Jun 1972, *Zamzow 36* (JEPS); **Siskiyou County**: Klamath River, between Shovel Creek and Fall Creek, 792 m, 15 May 1898, *Applegate 2137* (DS, RSA, US, US); Salmon Mt., SW slopes near summit, 1817 m, 20 May 1936, *Cantelow 1440* (CAS); Castle Lake, in a N-facing cirque with an abrupt 1000 ft. backwall, 14 miles SW of Mt Shasta on N side of a 6600 ft eastern spur of Trinity Mtns, 2000 ft above Sacremento River, within 10 airmiles of its headwaters to the west, 1656 m, 24 Jul 1943, *Culbertson 54* (RSA); Happy Camp-Takilma road at turn off to Bolan Lake, 1524 m, 25 Jun 1988, *Joyal & Mehlman 1272* (RSA, UC, US); Marble Mountain Wilderness Area, high lake basins in the vicinity of English Peak, approx., 0.2 mi below the 13 mile marker along the trail from English to Damas Lake, 2012 m, 11 Jul 1969, *Oettinger 971* (RSA); Salmon Mountains, High Lake basin in the vicinity of English Peak Marble Mountain wilderness area, below seepage on trail from English Lake to Diamond Lake, 1875 m, 15 Jul 1969, *Oettinger 1033* (BM); along trail from English to Diamond Lake (Marble Mountain Wilderness Area, High Lake Basins in vicinity of English Peak) Salmon Mountains, 1874 m, 15 Jul 1969, *Oettinger 1033* (RSA, UC); along trail above Willow Springs (between English Lake and Abbotts, Marble Mountain Wilderness Area, High Lake Basins in the vicinity of English Peak), Salmon Mountains, 1676 m, 12 Aug 1969, *Oettinger 1347* (RSA, UC); Kidder Creek, 914 m, 15 Jun 1950, *Parker 451* (DS); Black Bear Summit, south of Sawyer’s bar on ridge road to Cecilville, 1356 m, 17 Jun 1958, *Quick 58 -32* (CAS); Soda Creek, Klamath Forest, S25 T48N, R9W, Mt. Diablo Mer, 1707 m, 3 Jul 1934, *Wheeler 2853* (CAS, POM, RSA); Yreka, 21 Jun 1870, *Greene 877* (F, GH, MO, US); **Solano County**: Lake Solano, above the lake, 11 Apr 1984, *Crampton 9901* (CAS, UCD); Vacaville, 28 Apr 1902, *Heller & Brown 5407* (DS, E, GH, MO, US); Vaca Mountains, Weldon Canyon, 11 May 1938, *Jepson 18758* (JEPS); Vaca Mountains, Gates Canyon, 25 Apr 1943, *Jepson 20765* (JEPS); Vaca Mountains, Miller Canyon, Napa River basin, 23 May 1897, *Jepson 21262* (DS, JEPS); Cold Canyon Creek, Stebbins Reserve [University of California Nature Reserve], 0.5 mi S of Solano/Yolo county line [Putah Creek] near CA highway 128, 185 m, 21 May 1997, *Rhode & Nemeth 159* (CAS, RSA); Mix Canyon Road, 1.5 miles W of its junction with Pleasants Valley Road, 274 m, 12 Apr 1969, *Tee 4* (RSA); **Sonoma County**: Pine Flat, w slope of the Mayacama Mountain Range Pine Flat-Mayacama Mountain Range, Pine Flat, 25 Mar 1934, *Applegate 8854* (DS, RSA, UC); Mill Creek, N side along trail from Cosper’s Ranch to Cypress Green, 2 Jan 1929, *Baker 3401 b* (CAS); Jensen’s Ranch, 14 Apr 1929, *Baker 3489 b* (UC); Russian River, near Trenton, 16 Mar 1902, *Heller & Brown 5076* (US); Oakville to Glenn Ellen, 274 m, 20 May 1951, *Raven 2862* (CAS, GH); Healdsburg, resort at east base of Fitch Mtn, 9 Jun 1952, *Rübtzoff 1195* (CAS); Duncans Mills Marsh, along streamlet in upper portion of marsh, 16 May 1960, *Rübtzoff 4358* (RSA); **Stanislaus County**: Salada Creek Canyon, 9 mi sw of Patterson, Mount Hamilton Range, 213 m, 11 Apr 1937, *Crum 1766* (UC); Arroyo del Puerto, at mouth of canyon, E margin of Mount Hamilton Range, 76 m, 28 Mar 1935, *Sharsmith 1551* (UC); **Sutter County**: Sutter Buttes, Myer’s Ranch, 50 m, 7 Apr 1977, *Ahart* s.n. (CAS, MO); Sutter Buttes, S slope near the S fence line, ca. 3/4 mi SW of Myers home, Myers Ranch, 50 m, 24 Apr 1982, *Ahart 3384* (CAS); Marysville Buttes, 121 m, 18 Apr 1917, *Ferris 650* (DS); Marysville Buttes, slopes of West Butte, 22 Apr 1926, *Ferris 6372* (DS, POM); Marysville Buttes, E side of North Butte, 9 May 1936, *Lee 2076* (CAS, JEPS, JEPS); Marysville Buttes, 1/2 mi W and 1 mi S of North Butte, 304 m, 30 Mar 1934, *Sindel 39* (UC); **Tehama County**: Mendocino National Forest, about 17 miles E of Covelo, along Covelo-Paskenta road, between Eel River Ranger Station and Low Gap ranger Station, 29 May 1934, *Bacigalupi 2408* (DS, GH, POM, POM, UC); along State Hwy. 32 from Junct with State Hwy 89, 1036 m, 23 May 1957, *Balls 11216* (BM, E, S); Payne’s Creek, 4 miles W of Payne’s Creek, 22 Apr 1934, *Eastwood & Howell 1879* (CAS, DS); Log Spring Ridge, between Log Spring and Government Flat, 9 Jul 1941, *Eastwood & Howell 9714* (CAS, GH, US); Dales Lake Reserve, Cascade Range Foothills, on the W side of Manton Rd. (A6); ca. 1.5 mi N of Dales Station on Hwy. 36, ca. 14 mi NE of Red Bluff, between Manton Rd. and the preserve fence at the level of the S end of the Old Hwy. Pool, 219 m, 3 Apr 1995, *Oswald & Ahart 6606* (JEPS); Plum Creek Rd. 0.1 mi west of Hogback Rd., southeast of the town of Paynes Creek. in area of 1990 fire, 969 m, 14 May 1997, *Oswald & Ahart 8417* (CHSC, JEPS); Payne’s Creek, Red Bluff-Susanville road 2 3/10 miles below Payne’s Creek P.O, 365 m, 16 May 1937, *Wolf 8706* (GH, MO, RSA); **Trinity County**: Dinsmuir, Eureka-Red Bluff Road, 21 Jul 1916, *Abrams 6156* (DS); Hobo Gulch Camp, North Fork Trinity River, 18 miles NW of Weaverville (T36N, R12W, Sec. 25), 945 m, 16 Jun 1971, *Carter 234* (CAS); between Eagle and Bear Creeks, 25 Jun 1937, *Eastwood & Howell 4966* (CAS, GH); Salmon Mountains, Coffee Creek, 1310 m, 1 Jul 1909, *Hall 8631* (UC, US); One Eye Flat, about 1.7 miles N of Carrville, Trinity River Canyon, 756 m, 20 May 1980, *Howell et al. 53504* (CAS); Dedrick, 8 miles below Dedrick, 670 m, 15 May 1949, *Munz 13241* (CAS, RSA); Asbestos Gulch, on Forest Rd. 25 W of Castella, 2 mi W of Forest Rd. 26 to Mt. Shasta (city), 1512 m, 14 Jun 1994, *Oswald 6292* (JEPS); South Fork Mountain, near summit on road to low gap of Mad River, Northern Coast Ranges, 1219 m, 1 Jul 1923, *Tracy 6515* (UC); New River, bluffs at mouth, 426 m, 27 Apr 1924, *Tracy 6657* (UC); White’s Creek, at head of White’s Creek, Devil’s Canyon Mountains, 2073 m, 5 Aug 1935, *Tracy 14576* (UC); Trinity Summit, 2 mi E of Grove’s Prairie, 1524 m, 8 Aug 1936, *Tracy 15132* (JEPS, UC); Van Duzen River, halfway between Kuntz and Hettenshaw, 975 m, 3 Jul 1942, *Tracy 17399* (UC); Mad River, 5 8/10 mile above Eureka, Red Bluff Road on road to Ruth, 457 m, 20 Jun 1937, *Wolf 8924* (DS, RSA); NE of Weaverville, 853 m, 19 May 1914, *Yates 317* (CAS, UC); **Tulare County**: Sequoia National Park, Moro Creek Road above Hospital Rock Camp, 1067 m, 7 Jul 1948, *Bailey & Bailey 2028* (UC); Bear Peak, Southern Sierra Nevada region Chimney Peak Recreation Area, summit of northern high point on Bear Mountain, 2509 m, 27 Jun 2003, *Boyd et al. 10537* (RSA); Government Hill, Giant Forest, Sequoia National Forest, 1981 m, 22 Jun 1940, *Cronquist 2076* (MO); Sequoia National Park, S side of Elizabeth Pass, Kaweah River drainage, Sierra Nevada, 3201 m, Aug 1963, *Grannell 2236* (POM); Kern Plateau, canyon of Poison Meadow Creek on Cherry Hill Road, 2195 m, 13 Jul 1966, *Howell & True 41890* (CAS); Kern Plateau, on Dome Lands Trail about 0.5 miles S of Church Dome, 2317 m, 15 Jul 1971, *Howell & True 48526* (CAS); Slate Mountain, S slopes, above Windy Gap, 2134 m, 28 Jul 1972, *Leskinen 1002* (CAS); Tulare County, Sequoia National Park, Soda Creek on Middle Tule River, 2134 m, Jun 1896, *Purpus 1820* (UC); Sequoia National Forest, along N fork of Middle fork of Tule River, 2.4 miles up Camp Wishon road from power plant, 1158 m, 7 Jun 1967, *Thorne & Everett 36937* (RSA); Kern Plateau, Troy Meadow road on the ridge above Beach Meadow, 2408 m, 26 Jun 1968, *Twisselmann 14489* (CAS, MO, RSA); Kings Canyon National Park, General Grant Grove, 26 May 1935, *Woglum 1096* (RSA); Tule River, North fork of Tule River, 3.6 mi N of Springville, 396 m, 13 May 1933, *Wolf 4648* (A, CAS, RSA); Grapevine Spring, 35 m E of Visalia, head of the San Joaquin Valley, Mar 1898, *Woolsey* s.n. (UC); **Tuolumne County**: Jamestown, 1 mile W of town, 1 Apr 1923, *Abrams 10023* (DS); 4 mi E of Sonora, Sierra Nevada, 29 May 1944, *Alexander & Kellogg 3585* (JEPS, RSA, UC); Phoenix Lake, E side of lake 5 miles SE of Columbia, 13 May 1915, *Grant 51* (MO, POM); Kennedy Meadow, near Kenndey Lake, 3 Sep 1915, *Grant 452* (JEPS, MO, POM); Sonora Pass Road, 10.7 mi E of Pine Crest, 1829 m, 19 Jul 1957, *Hesse 2292* (JEPS); Stanislaus National Forest, 0.5 mi E of Mt. Lewis, 1524 m, 27 May 1925, *Hough 13* (CAS); Columbia, northeast of Columbia on road to Vallecito, 3 May 1960, *Howell 35273* (CAS); Yosemite National Park, W of Vernon Lake, SW end of Moraine Ridge, Sierra Nevada, 2286 m, 24 Jul 1938, *Mason 11985* (CAS, UC); 2.4 mi ESE of Don Pedro Bar, Sonora quadrangle Sec. 29, T2S, R15E, 396 m, 26 Mar 1936, *Nordstrom 643* (UC); Dodge Ridge, near Pinecrest, 1859 m, 22 Jul 1952, *Quick 52-217* (CAS); Red Hills, BLM Management area, 3.6 miles SW of Chinese Camp, 457 m, 31 May 1984, *Stone 615* (CAS); Stanislaus National Forest, Sugarpine on Hwy 108, ca. 12 air miles NE of Sonora, 1341 m, 27 Aug 1997, *Taylor 16195* (JEPS); Chipmunk Flats, about 5 mi W of Sonora Pass, 6 Jul 1935, *Wiggins 8052* (DS, RSA, UC); Penon Blanco mine, near mine above Indian Creek, 365 m, 27 Apr 1919, *Williamson 14* (A, CAS, DS, POM, UC); **Ventura County**: Red Reef Canyon, Topa Topa Mountains, 853 m, 8 Jun 1908, *Abrams & McGregor 154* (DS, E, US); Topa Topa Mountains, Transverse Ranges, Mount Pinos region east of Sespe Canyon near upper end of Tar Creek Road (and trail), 11 May 2004, *Boyd & Morgan 11174* (RSA, UC); Sespe Creek, Transverse Ranges, Mount Pinos region, 1000 m, 2 May 1935, *Clokey & Anderson 6882* (CAS, DS, MO, POM, UC, US); 2 mi S of Chuchupate Forest Station on raod 8N04 to Frazier Mountain, 1509 m, 22 Jun 1975, *Davidson 2822* (RSA); Casitas Lake, Santa Ynez Mountains N of Ventura, Lake Casitas (Matilija 7.5’ Q), 182 m, 5 Mar 1968, *Goeden & Ricker* s.n. (RSA); Simi Valley, Transverse Ranges: Santa Susana Mts. region, north side of mountain, 28 Apr 1948, *Goodfellow* s.n. (RSA); Transverse Ranges, Mt. Pinos region, Piru Creek adjacent to Highway 126, 5 May 1992, *Kegarice 9* (RSA); Wheeler Hot Springs, Ojai Valley, coast, 304 m, 11 Apr 1936, *Livers 94* (POM); Los Padres National Forest, 12 mi S of California Route 166, on state route 33, 945 m, 27 May 1993, *Miller et al. 7974* (MO); ca. 1 mi. west of Ventura River estuary, 30 Mar 1967, *Pollard* s.n. (RSA); Foster Park, N face of Red Mountain, Foster Park, 152 m, 22 Apr 1971, *Pollard* s.n. (CAS, JEPS, RSA); Conejo Mountain, Santa Monica Mountains region, 168 m, 10 May 1959, *Raven & Thompson 14188* (CAS, GH, RSA); Santa Susana Mtns., Western Transverse Range,Newhall Ranch, west side of Salt Canyon watershed, toward Tapo Canyon, 1.2 km west of Los Angeles Co. line, 351 m, 14 May 2003, *Sanders & Wotipka 26640* (RSA); Apache Canyon, Santa Barbara National Forest, 1219 m, 11 Apr 1934, *Sowder 371* (UC); Cuyama Road near the Ventura Co. line, 29 Apr 1939, *Stubblefield* s.n. (RSA); Santa Monica Mountains, lower end of long Grade Canyon ca. 0.5 mi E of Camarillo State Hospital, 8 Jun 1968, *Thorne & Thorne 37726* (RSA); Moorpark, foothills of “Big Mountain” northeast of Moorpark College, 213 m, 23 Apr 2003, *White et al. 9088* (RSA); Santa Ynez Mountains, Ojai to Cuyama Valley Road, North Fork of Ventura River, 2.2 miles below Wheelers Hot Springs, 366 m, 21 May 1935, *Wolf 6885* (CAS, DS, MO, RSA); Point Mugu, 4 Jun 1977, *Zembal* s.n. (RSA); **Yolo County**: Cache Creek Canyon, 8 May 1903, *Baker 2890* (B, ECON, F, GH, K, LE, MO, POM, UC, US); Rumsey, 5 air mi WSW of Rumsey, Rayhouse Road 4.3 mi from Hwy 16, E side of ridge between Blue Ridge and Little Blue Ridge, 0.4 mi below crest at large roadcut, 731 m, 28 May 1989, *Ertter & Carter 8533* (MO, RSA, UC); Putah Canyon, 3.5 miles W of Winters, 5 May 1949, *Hanson 14* (RSA); Rumsey, about 1 mi N of Rumsey (NW of Highway 16), 10 Apr 1947, *Heiser 1902* (UC); Putah Canyon, 2 mi below Yolo-Napa County line, S slope of Putah Canyon, 6 Jun 1948, *Skoss 28* (UC); Buckeye Creek, mouth of creek, 1 Jun 1916, *Stinchfield 342* (DS). **Nevada**: Carson City, 1865, *Anderson* s.n. (GH); **Douglas County**: Minden Highway to Woodford’s near state line, 6 Jun 1937, *Lehenbauer 37* (GH); Taylor Canyon, Sierra Nevada, Carson range, 1.2 road miles NW of Jack’s Valley Road on highway 207 (Kingsbury Grade), 1524 m, 19 Jun 2002, *Tiehm 14027* (CAS, RSA); **Washoe County**: Highway 395 between Reno and Carson City, 22 Jun 1954, *Kruckenberg 3324* (DS, RSA); Davis Creek Campground, ca. 1 mi W of highway 395, ca. 12 mi N of Carson City, 2 Jul 1979, *Taylor 2090* (CAS, MO); **Oregon**: **Curry County**: Solitude Bar, 26 Jun 1917, *Nelson 1530* (GH); **Jackson County**: Siskiyou Camp, along the Pacific Highway, N slope of Siskiyou Mtns, 11 Jun 1928, *Applegate 5451* (DS); Ragsdale Butte Lookout, ca. 12 mi NW of Trail, 1371 m, 29 Jun 1939, *Hitchcock & Martin 5044* (DS, GH, POM, UC); Siskiyou Summit, 1280 m, 23 Jun 1929, *Kildale 8308* (DS); Union Creek, Golden Star Trail, 22 Jun 1928, *Sipe* s.n. (MO); **Josephine County**: Grant’s Pass, 5 miles N on Pacific Highway, 12 May 1924, *Abrams & Benson 10417* (DS); Merlin, 14 May 1887, *Henderson 1379* (MO); Rogue River, near bridge on road to Galice, 18 Apr 1926, *Henderson 6050* (CAS, DS, MO); Jump-Off-Joe, May 1884, *Howell* s.n. (GH); Grave Creek, 21 May 1884, *Howell* s.n. (K, S); Bolan Lake, ridge 2.5 miles W of lake, road between Waldo and Bolan Lake; Siskiyou Mtns, 1371 m, 23 Jun 1950, *Kruckenberg 1917* (CAS, S); Kerby, 13 Jun 1904, *Piper 6101* (K, US); Grant’s Pass, 8 Jun 1912, *Prescott* s.n. (GH). **Washington**: **Lake County**: Mt. Konocti, W slope above Breen’s Lake, 13 Jul 1929, *Benson 1777* (K, POM).

#### 42. Solanum uncinellum

Lindl., Edwards’s Bot. Reg. 26: t.15. 1840

http://species-id.net/wiki/Solanum_uncinellum

Figure 101

Solanum scandens L., Pl. Surin. 6 [5?]. 1775, non *Solanum scandens* Miller, 1768. Type:Surinam. Sin. loc., *Anon*. [*C.G. Dalberg*] (lectotype, designated by Knapp and Jarvis 1990, pg. 356: LINN 248.24 [BH neg. 6807]).
Solanum laetum Miq., Stirp. Surin. Sel. 135. 1851. Type:Surinam. Sin. loc., *H.C. Focke & A. Kappler 616* (holotype: U [U0006807]).
Witheringia pendula Roem. & Schult., Syst. Veg., ed. 15 bis [Roemer & Schultes], 3: 522. 1818. Type: Brazil. Sin. loc., *Anon*. (holotype: B?, destroyed, originally from Link herbarium; no duplicates located).
Solanum pensile Sendtn. in Mart., Fl. Bras. 10: 50. 1846. Type: Brazil. Pará: “in sylvis ad Santarem e alibi prope fluvium Amazonum per prov. Paraënsum”, *C. Martius s.n. [2746]* (lectotype, designated here: M [M0166108]).
Solanum ipomoea Sendtn. in Mart., Fl. Bras. 10: 50. 1846. Type: Brazil. Amazonas: Coari, Rio Negro, Nov, *C. Martius* s.n. (lectotype, designated here: M [M0171831, F neg. 6535].
Solanum leucosporum Dunal, Prodr. [A.P. de Candolle] 13(1): 99. 1852. Type: Surinam. Sin. loc., 1845, *W.R. Hostmann 1100* (holotype: G [G00301650]; isotypes: BM [BM000778127], K [K000196564], LE, OXF [OXF00055157, OXF00055160], U [U0006808], W [1889-291698]).
Solanum sempervirens Dunal, Prodr. [A.P. de Candolle] 13(1): 88. 1852. Type: Guyana. Sin. loc., 1839,* R. Schomburgk 594* (lectotype, designated here: G-DC [G00144693, F neg. 6740, IDC microfiche 800-61.2068:II.3]; isolectotypes: B [F neg. 2698] destroyed, BM [BM000934974, BM000778126],F [F-533357], G [G00070226], K [K000196558, K000590200], L [L-905298-52], TCD [TCD0006846]).
Solanum ipomoeum St.-Lag., Ann. Soc. Bot. Lyon 7: 135. 1880, nom. illeg. superfl. Type: Based on *Solanum ipomoea* Sendtn.
Cyphomandra yungasense Rusby, Bull. Torrey Bot. Club 26: 195. 1899. Type: Bolivia. La Paz: Yungas, 6000 ft, 1885, *H.H. Rusby 2475* (holotype: NY [NY00138678]).
Solanum ipomoeoides Chodat & Hassl., Bull. Herb. Boissier sér. 2, 4: 80. 1903. Type: Paraguay. Cordillera: Caraguatay, Oct 1900, *É. Hassler 3320* (lectotype, designated here: G [G00070177]; isolectotypes: G [G00070176, G00070178], K [K000196523], P [Morton neg. 8218]).
Solanum ipomoea Sendtn. var. *angustifolium* Witasek, Kaiserl. Akad. Wiss. Wien, Math.-Naturwiss. Kl., Denkschr. 79: 333. 1910. Type: Brazil. São Paulo: Rio Paranapanema, Salto Grande, 500 m, *R. von Wettstein & V. Schiffner* s.n. (holotype: W [W-1922-0001512**,** F neg. 33082]; isotype , F [F-871102]).
Solanum ipomoea Sendtn. var. *ipomoeoides* (Chodat & Hassl.) Hassl., Repert. Spec. Nov. Regni Veg. 15: 119. 1918. Type: Based on *Solanum ipomoeoides* Chodat & Hassl.
Solanum ipomoea Sendtn. var. *macrostachyum* Hassl., Repert. Spec. Nov. Regni Veg. 15: 120. 1918. Type: Paraguay. Upper Rio Paraná, *K. Fiebrig 5848* (holotype: G [G00070172, Morton neg. 8600]; isotypes: G [G000701723, G00070174, G00070175], US [US-1175779]).
Solanum penduliflorum Rusby, Descr. S. Amer. Pl. 113. 1920, non *Solanum penduliflorum* Dammer, 1912. Type: Colombia. Magdalena: Santa Marta, 1/4 mile from coast, Don Diego, 5 May 1898-1899, *H H. Smith 2661* (holotype: NY [NY00172127]; isotype: CM [CM-211196]).
Solanum styracioides Rusby, Mem. Torrey Bot. Club 4: 230. 1895. Type: Bolivia. La Paz: Prov. Larecaja, between Tipuani and Guanai, Dec 1892, *M. Bang 1662* (lectotype, designated here: NY [NY00726013]); isolectotypes: BM [BM000778122], E [E00190766], G [G00070208, G0007209], LE, MICH, MO [MO-5468314], NY [NY00726014], W [W-1893_5619]).
Solanum volubile Rusby, Bull. Torrey Bot. Club 26: 194. 1899, non *Solanum volubile* Sw., 1797. Type: Bolivia. Beni: junction of Río Beni and Río Madre de Dios [i.e. Riberalta], Aug 1886, *H. Rusby 839* (holotype: NY [NY00172248]; isotypes: BM [BM000778194], GH [GH0077786], NY [NY00172247, NY00172246], US [US-1324894, US-32591]).
Solanum tinctum C.V.Morton, Contr. U.S. Natl. Herb. 29: 43. 1944. Type: Based on *Solanum penduliflorum* Rusby
Solanum miquelii C.V.Morton, Contr. U.S. Natl. Herb. 29: 43. 1944. Type: Based on *Solanum laetum* Miq.
Solanum scandens L. var. *laetum* (Miq.) Bitter ex Amshoff, Bull. Torrey Bot. Club 75: 655. 1948. Type: Based on *Solanum laetum* Miq.
Solanum granelianum D’Arcy, Ann. Missouri Bot. Gard. 60: 758. 1974 [1973]. Type: Panama. Darién: Cana-Cuasi Trail, Chepigana District, 5500 ft., 17 Mar 1940, *M.E. Terry & R.A. Terry 1605* (holotype: MO [MO-1195589]; isotypes: A [A00077492], BKL [00002338], F [F-1066335]).
Solanum palenquense D’Arcy, Selbyana 2(1): 63. 1977. Type: Ecuador. Los Ríos: Palenque Science Center, halfway between Santo Domingo de Los Colorados and Quevedo, 150-220 m, 26 Oct 1974, *C. Dodson 5674* (holotype: MO [MO-2251894, flowers in packet only, see below]; isotypes SEL [n.v.], US [US-2843963], WIS [frag.], Río Palenque Science Center [n.v.]).

##### Type.

Cultivated in the “garden of the Horticultural Society” [England. Chiswick], July 1837, *Anon*. (holotype: CGE).

##### Description.

Large woody vines, climbing to canopy by means of twining petioles. Stems often hollow, minutely puberulent with tiny simple 1–2-celled trichomes to densely pubescent with dendritic trichomes to 1 mm long, the trichomes sometimes enlarged at the base; new growth minutely puberulent to densely pubescent with dendritic trichomes. Bark of older stems dark reddish brown, glabrescent. Sympodial units plurifoliate, not geminate. Leaves simple or occasionally pinnately 2–3-lobed, (2.5-)6–15(-20) cm long, (1.5-)3–9(-11) cm wide, elliptic to narrowly ovate, widest near the middle or in the basal third, coriaceous to chartaceous, the upper surfaces glabrous and somewhat shiny to pubescent with simple uniseriate trichomes along the veins to evenly pubescent on the veins and lamina with dendritic trichomes to 1 mm long, the lower surfaces glabrous (W Ecuador) to minutely simple puberulent to densely dendritic pubescent, the pubescence denser than that of the upper surfaces; primary veins 6–8 pairs, conspicuously arched; base acute to truncate or cordate; margins entire or pinnatifid, the sinuses to within 0.5 cm of the midrib; apex acute; petiole 1–6 cm long, very variable in length along the stem, minutely puberulent to densely pubescent with dendritic trichomes like those of the stems and leaves, especially in the adaxial groove, twining. Inflorescence terminal or sometimes lateral, 4–20(-30+) cm long, several times branched, the branches very variable in length, with up to 100 flowers, finely and densely puberulent with tiny simple trichomes to densely pubescent with dendritic trichomes; peduncle 1.5–6 cm long, not particularly distinct; pedicels 0.5–1 cm long, 0.5–1 mm in diameter at the base, 1.5–2 mm in diameter at the apex, stout, spreading at anthesis, minutely puberulent to densely pubescent like the rest of the inflorescence, the pubescence sparser distally, articulated at the base from a small sleeve leaving a tiny peg on the inflorescence axis; pedicel scars more or less evenly spaced 2–5 mm apart on the flowering parts of the inflorescence rhachis. Buds narrowly ellipsoid and tapering, with a terminal pointed nipple, the corolla very exerted from the calyx tube early in bud. Flowers all perfect, 5-merous. Calyx tube 1–1.5 mm long, conical or broadly conical, the lobes apparently absent or mere apiculae from the rim to broadly deltate, glabrous to very minutely puberulent, the tips with a few uniseriate simple trichomes. Corolla 2–3 cm in diameter, purple, violet or white, often with a mixture of colours, fleshy, deeply stellate, lobed nearly to the base, the lobes 12–16 mm long, 1.5–2.5 mm wide, planar at anthesis, densely papillate on both surfaces with simple trichomes (papillae) to 0.3 mm, or the adaxial surface glabrous with only a few trichomes along the keeled lobe midvein, these giving the flowers a white cast in dried material, the tips cucullate. Filament tube minute, the free portion of the filaments markedly unequal, one anther with a longer filament 2–5 mm long, the other 4 anthers with filaments 1–2 mm long, glabrous or occasionally minutely puberulent with simple papillae within; anthers 5–8 mm long, 1.5–2 mm wide, tapering, the base markedly sagittate to hastate, the lobes 0.5–1 mm long, tightly connivent, yellow, poricidal at the tips, the pores lengthening to slits with age. Ovary glabrous; style 10–13 mm long, usually equal in length to the longest anther, densely pubescent in the basal 2/3 (within the anther tube) with tiny dendritic trichomes to 0.5 mm long or very occasionally (W Ecuador) glabrous or only minutely puberulent, held tightly against the anther with the long filament; stigma capitate, occasionally somewhat bilobed, the surface minutely papillate. Fruit a globose berry, to 2 cm in diameter, red, violet or metallic blue when ripe, not shiny, glabrous, the pericarp thin and leathery; fruiting pedicels to 1.5 cm long, ca. 2.5 mm in diameter, apparently erect, but probably hanging from the weight of the berry. Seeds 20–30 per berry, 3–7 mm long, 2–5 mm wide, flattened-reniform, the surfaces appearing hairy from the lateral testa cell walls, these to 1 mm long, the testal cells pentagonal. Chromosome number: not known.

**Figure 101.**
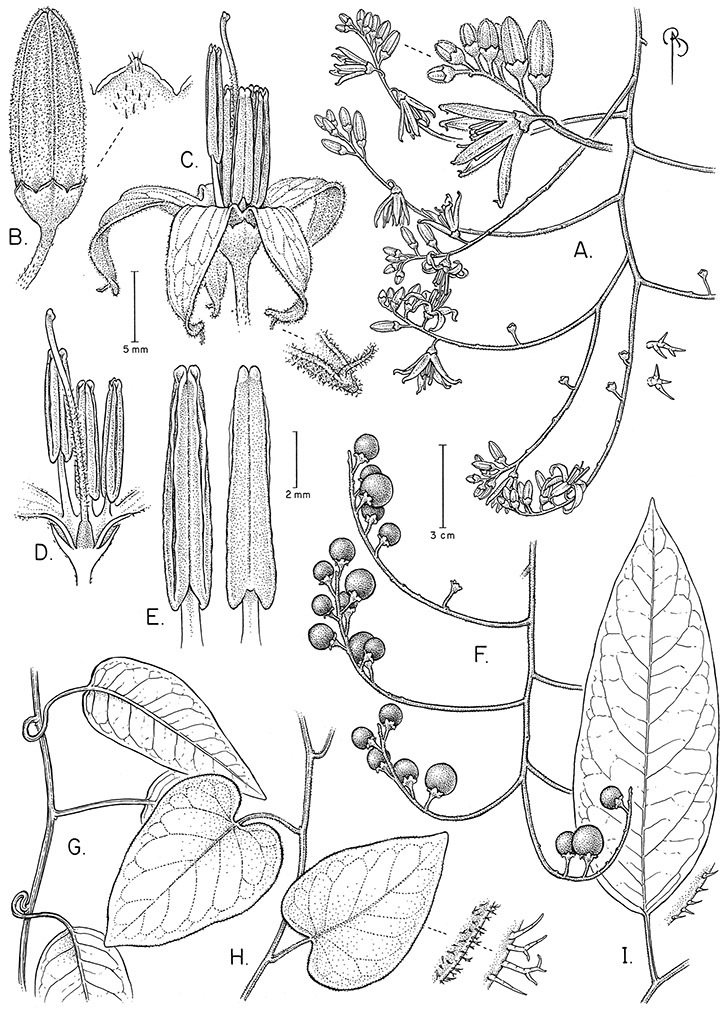
*Solanum uncinellum* Lindl. (**A–F** drawn from *Hammel et al. 21289* **G** drawn from *Nee 37364* **H** drawn from *Nee 36071* **I** drawn from *L.B.B. 9830*). Illustration by Bobbi Angell.

##### Distribution

**Figure 102.**
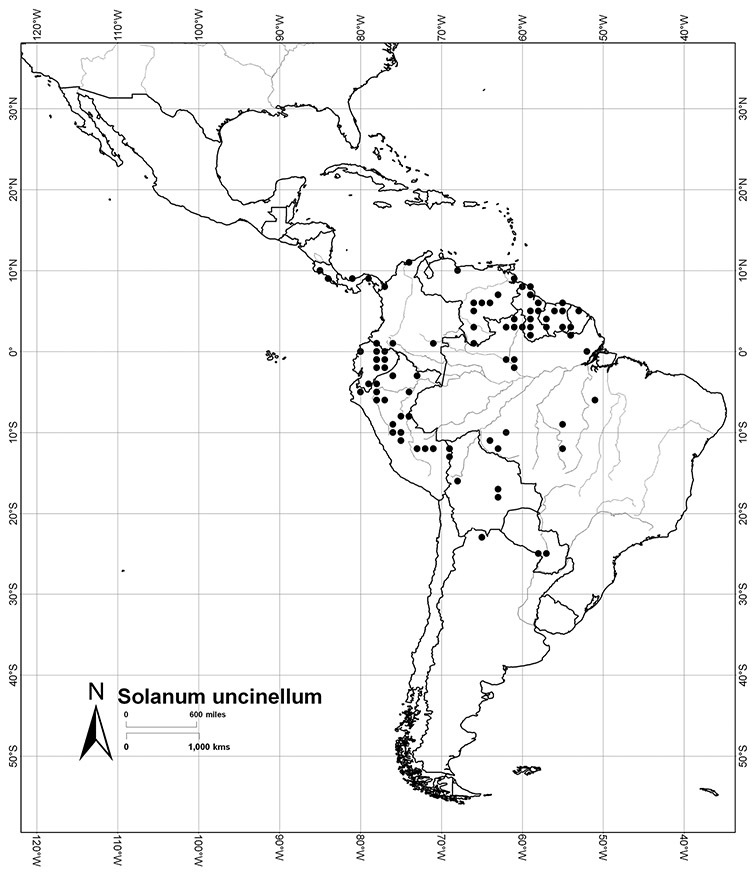
Distribution of *Solanum uncinellum* Lindl.

(Figure 102). Widely distributed throughout tropical America from Costa Rica to Argentina, from 0–2200 m elevation.

##### Ecology.

In a wide variety of open and exposed habitats from lowland rainforest to dry chaco vegetation; usually in open sites either on forest margins or a canopy liana.

##### Common names.

Peru: margarita (*Ferreyra 1022*); Bolivia: cashixopá (Chácobo language, *Boom 4101*).

##### Conservation status.

Least Concern (LC); EOO >100,000 km^2^ (LC) and AOO >10,000 km^2^ (LC). Marginal populations that are morphologically distinct may harbour interesting genetic variation. See Moat (2007) for explanation of measurements.

##### Discussion.

**Figure 103.**
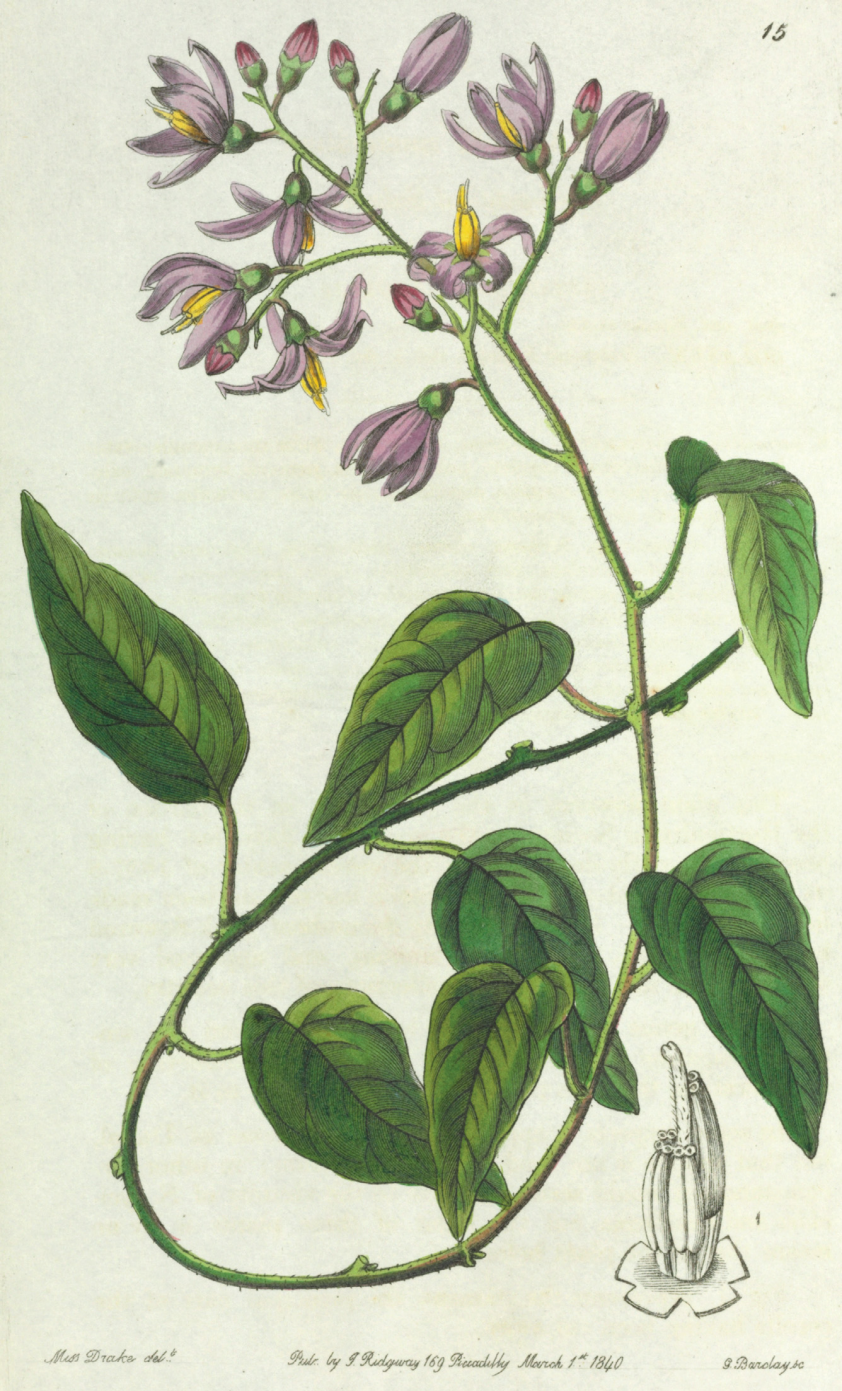
Plate accompanying the original description of *Solanum uncinellum* Lindl. clearly showing the unequal anthers and narrow petals typical for this species (Lindley 1840: tab. 15). Reproduced with permission of the Natural History Museum Botany Library.

*Solanum uncinellum* is the oldest name for the species that has variably been called either *Solanum pensile* or *Solanum ipomoea*, depending upon the type of pubescence (see below). The provenance of the plant grown in the Horticultural Society’s garden in Chiswick (London, England) was not known to Lindley, but from the fact that it was grown outdoors in England it is likely to have been from the southern part of the species range. The lack of branched pubescence may be due to the wet conditions under which it would have been grown in England (see Figure 103). The holotype in the Lindley herbarium at CGE is labelled “HHS July 1837” (Herbarium of the Horticultural Society) and “S. uncinellum, Bot. Reg. 1840 t. 15” in Lindley’s hand.

Rusby (1895) described a new section *Andropedas* for his *Solanum styracioides*, citing its unusual anther morphology, which he considered intermediate between *Cyphomandra* (=*Solanum* section *Pachyphyllum* (Dunal) D’Arcy) and *Solanum*. The tapering anthers on unequal filaments are unique in the Dulcamara clade, and were considered a distinguishing feature by Rusby. *Solanum dulcamara* also has tapering anthers, but they are tightly connivent with the pores operating as a single opening (Glover et al. 2004), while those of *Solanum uncinellum* are more loosely associated. Other species in the clade with unequal filaments (e.g., *Solanum seaforthianum*) have ellipsoid anthers.

*Solanum uncinellum* is a very widespread and variable species, occurring in a huge range of habitats throughout the American tropics. The extremes of pubescence variation in *Solanum uncinellum* look very different, but an entire range of intermediates occur scattered throughout the region; no consistent geographic patterns can be discerned. Even within a single collection (e.g., duplicates of *Schomburgk 594*), almost glabrous and densely pubescent sheets can be seen. Plants from the southern part of the range (the Chaco of Bolivia, Paraguay and Argentina) are more consistently pubescent with dendritic trichomes and often have cordate leaf bases (as do both syntypes of *Solanum ipomoea*), and those from western Ecuador have almost completely glabrous leaves. Specimens from the Amazon and Guianas, however, are mostly dendritic-pubescent with elliptic leaves or have a mixture of dendritic and simple trichomes of varying densities. Pubescence density and type may depend upon the microclimate or exposure status of the plant or part of plant, as has been observed in other species (e.g., *Solanum confertiseriatum*, see Knapp 2002). Flower morphology is remarkably consistent throughout the range of the species, although flower size varies from plant to plant.

Juvenile leaves of *Solanum uncinellum* are not known, but are likely to be pinnate or deeply pinnatifid. A series of sterile specimens from tropical America variously identified as “*Solanum dulcamara*” (MacBride 1962) are probably juvenile specimens of *Solanum uncinellum*. Some specimens (*Rimachi 8110*) from the Iquitos area of Peru are densely pubescent like those from the southern portion of the species range, and have pinnate leaves on reproductive stems. Associating juvenile foliage with adult plants of *Solanum uncinellum* will be difficult, but would be useful in determining if, like many other members of the clade (e.g., see *Solanum dulcamaroides*), this species has pinnate pre-reproductive leaf morphology.

*Solanum uncinellum* is not easily confused with any other Neotropical *Solanum* species; the elongate flower buds with an apical nipple, narrowly stellate corollas and large complex inflorescences are all distinctive. In fruit it could be confused with other members of the Dulcamaroid clade, but short petioles and “hairy” seeds distinguish it from any other South American species. “Hairy” seeds also occur in *Solanum dulcamaroides* and *Solanum seaforthianum*, but those taxa differ from *Solanum uncinellum* in flower shape (neither of them are deeply stellate with narrow corolla lobes) and morphology (only *Solanum seaforthianum* has unequal filaments, but not as pronounced as in *Solanum uncinellum*) and in leaf characters. The leaves of *Solanum seaforthianum* are glabrous and pinnate, while those of *Solanum uncinellum* are usually variously pubescent; the leaves of *Solanum dulcamaroides* have a promounced submarginal vein.

*Solanum flaccidum*, with which *Solanum uncinellum* is sympatric in the southeastern part of its range, also has anthers borne on unequal filaments, but in that species the anthers are ellipsoid, not tapering, and the long filament is less than twice the length of the rest of the filaments, whereas in *Solanum uncinellum* the long filament is approximately two times the length of the rest. The corollas of *Solanum flaccidum* are also more rotate than those of *Solanum uncinellum*, with broader lobes.

From specimen labels it appears that *Solanum uncinellum* has red, purple or metallic blue berries when ripe. Fruiting collections with ripe berries are not especially common, and these color differences do not appear to have a geographic component. It may be that berry color changes through development, as has been observed in other species such as *Solanum nitidum* (also of the Dulcamaroid clade; see Knapp 1989).

Linnaeus described *Solanum scandens* using collections from Surinam he cited as coming from C.G. Dalberg, and although there is no direct evidence on the lectotype specimen that Dalberg actually collected it, it is likely to have been one of his gatherings. In 1781, Linnaeus filius included a *Solanum scandens* in his Supplementum (Linnaeus 1782), which has often been taken as the coining of a new name. Bearing in mind the materials available to Linnaeus filius, his direct citation of a Dalberg specimen and the fact that LINN 248.24 is the only sheet of *Solanum uncinellum* in the Linnaean herbarium, it is clear that rather than coining a new name, Linnaeus filius was including his father’s *Solanum scandens* in his own treatment.

Six syntypes were cited in the protologue of *Solanum pensile*, many of these are un-numbered collections that have been difficult to trace with certainty. The lectotype selected here at M (M0166108) has a long descriptive label in Sendtner’s hand and is numbered as “2746”. Martius’s herbarium name of “Solanum arcuatum” is crossed out and the epithet *pensile* written above it. Two specimens were cited in the description of *Solanum ipomoea*, I have selected as a lectotype that sheet in M (M0171831) from Coari on the Rio Negro that has a label with a description in Sendtner’s hand and is in flower. The other syntype *Martius* s.n. at M (M0171830) from Pará does not have such a label, and is only in fruit.

A label with a note in Dunal’s hand on holotype specimen of *Solanum leucosporum* at G reads – “Il faut ecrire leucoporum Dun! non leucosporum comme dans le Prodromus ou on a corrige mal a propos en mettant une S, ‘antherae poris duobus niveis’ Dun. l.c.” [It is necessary to write leucoporum Dun! not leucosporum as it is written in the Prodromus where the letter S has been inserted incorrectly; ‘anthers with two white pores’] ; he had clearly wanted to name this species S. “leucoporum” for the clearly delineated pores on the attenuate anthers.

Dunal (1852) cited two duplicates of *Schomburgk 594* in his description of *Solanum sempervirens*, one from the Boissier herbarium and another from “herb. DC”. I have selected the G-DC duplicate as the lectotype for this name as it is better preserved. Two Bolivian collections from the Department of La Paz were cited in the original description of *Solanum styracioides*, *Bang 522* of unspecified locality (“Yungas”) and *Bang 1662*, collected two years later; I have selected the latter as the lectotype as it is represented by more duplicates that are more widely distributed.

Two Hassler collections from central Paraguay were cited in the original description of the extremely pubescent form of *Solanum uncinellum* (*Solanum ipomoeoides*), *Hassler 3320* and *Hassler 4093*: the former is represented by more duplicates and is selected here as the lectotype.

The holotype of *Solanum palenquense* at MO and an isotype at US both have stems with markedly bicolorous leaves attached mounted on the sheet and loose flowers (and fruit) in a packet. Michael Nee (pers. comm.) has suggested that the finely ridged stems and petioles plus the sharp tooth-like tips of the nearly entire margin show the leafy stems are from a plant of *Piptocarpha poeppigiana* (DC.) Baker (Asteraceae). The flowers in the packets on both sheets are definitely those of *Solanum uncinellum* and the logical lectotype would be the contents of the packet of the MO sheet. The mixed collection is certainly due to the canopy vine status of both taxa.

##### Specimens examined.

**Argentina**. **Chaco**: General Vedia, Jan 1933, *Meyer 830* (A); **Corrientes**: Capital, Isla Lagraña, in the Río Paraná a little N of the mouth of the Arroyo Sombrero, 14 Dec 1976, *Pedersen 11548* (A, CORD, K, MO); **Formosa**: Monte Tuayalea [?], 1919, *Jörgensen 2986* (GH, MO, SI); Pilcomayo, El Paraíso, 4 Oct 1981, *Valla et al. 17665* (MO); **Salta**: Orán, Finca San Andres. Administracion Las Juntas, 30 Oct 1997, *Schinini et al. 33035* (F, GH, MO); Orán, Campo Grande, 600 m, 24 Nov 1927, *Venturi 5588* (BM, F, GH, S, SI).

**Bolivia**. **Beni**: General José Ballivián, Espíritu, 200 m, 12 Sep 1986, *Beck 5988* (CORD, K); **La Paz**: Nor Yungas, Corocoro, 12 km NE of Caranavi, 1400 m, 16 Jan 1984, *Gentry et al. 44346* (MO); Nor Yungas, 10 km by road (ca. 5 km by air) N and above Caranavi, 1400 m, 1 Nov 1984, *Nee & Solomon 30307* (F, MO); Sud Yungas, al Beni 10 km de Palos Blancos hacia Yucumo, 920 m, 24 Dec 1987, *Seidel & Schulte 3205* (CORD); **Santa Cruz**: Andrés Ibáñez, 12 km E of center of Santa Cruz, on road to Cotoca, 375 m, 28 Jan 1988, *Nee 36071* (G, MO, US); Ñuflo de Chavez, along road from Colonia Okinawa 1 to San Ramon, 2 km SW of Los Troncos, alluvial plain of E side of Rio Grande, 250 m, 2 Dec 1990, *Nee & Coimbra 40147* (MO); Ichilo, Río Yapacani, 1 Oct 1926, *Steinbach 7588* (GH); Ñuflo de Chavez, c. 1 km N of Puerto Rico on road to Trinidad. Alt. 300 m, 27 Sep 1998, *Wood & Mamani 14006* (K).

**Brazil**. **Acre**: 20 km from Rio Branco-Porto Acre road, 11 Oct 1980, *Lowrie et al. 477* (F); Monte Mó, Rio Acre, Dec 1911, *Ule 9759* (G, K); Rletterpfl. Seringal S. Francisco, Apr 1914, *Ule 9762* (K, L, US); **Amapá**: Mazagão, BR156, road under construction which will connect Macapá with Monte Dourado, 81 km WSW of Macapá, ca. 11 km SW of Rio Preto, 20 Dec 1984, *Mori & Cardoso 17473* (K, MO, US); **Amazonas**: Tefé, Paranaguá, basin of Rio Jurua, 22 May 1933, *Krukoff 4535* (A, G, K, S, US); Humaitá, Livramento, near Livramento, on Rio Livramento, basin of Rio Madeira, 12 Oct 1934, *Krukoff 6751* (A, G, K, S); Rio Negro, Delta of the Rio Jauaperi, 11 Jun 1989, *Mori et al. 20468* (MO); Rio Negro, Santo Antonio, 8 Aug 1991, *Mori & Gracie 21971* (US); along the Rio Negro between Manaus and São Gabriel, above and below junction with Rio Branco, E of Carvoeiro, 26 Jun 1979, *Poole 1621* (GH, K, MO, US); Solimoês, Jun 1857, *Spruce 1706* (K); **Bahia**: Itapebí, Faz. Lombardi, rod. Sta. Maria Eteran, 14 Aug 1971, *Santos 1801* (US); **Espírito Santo**: Linhares, km 6-8 ramal do lado L. proximo do Vale do Rio Doce, 2 Oct 1971, *Santos 2041* (US); **Maranhão**: Maracassumé River region, 13 Sep 1932, *Froes 1895* (A, K, S); Island of São Luís, Estrada do Tirical, Feb 1939, *Froes & Krukoff 11538* (F); Maracassumé River region, 13 Sep 1932, *Krukoff 1895* (G); **Mato Grosso**: Sinop, 24 km E of BR163 at Rio Celeste on road to Vera (MT225), 20 Sep 1985, *Thomas et al. 3916* (K, US); Mun. de Novo Mundo, Parque Estadual Cristalino, acampamento 35 km acima de pousada, 257 m, 11 Feb 2008, *Zappi 1192* (K); **Pará**: Belém, south forest of the I.A.N, 22 Dec 1942, *Archer 8020* (US); Boa Vista on the Tapajos river, May 1929, *Dahlgren & Sella 211* (F); Rodovia Belem-Brasilia, km 93, 19 Aug 1959, *Kuhlmann & Jimbo 54* (MO); Districto Acará. Thomé Assú; Maquita, 50 m, 3 Aug 1931, *Mexia 6040* (BM, GH, K, US); Rio Jari, margem dereita, entre S. Melitão e Monte Dourado, 5 May 1968, *Oliveira 4398* (US); BR 163, km 913; Cuiabá-Santarem, north of Rio Pará 13 Nov 1977, *Prance et al. 25324* (F, US); Altamira, km 23 da Transamazonica, Centro de Experimentação de EMBRAPA, 21 Oct 1977, *Silva et al. 3464* (BH); Parque Nacional do Tapajós, km 60 da estrada Itaituba-Jacarecanga, 20 Nov 1978, *Silva & Rosario 3848* (BH); Serra dos Carajás. AMZA camp 3-Alfa, 475 m, 7 Jun 1982, *Sperling et al. 5946* (K, US); **Rondônia**: Boa Vista, Mata alagada na margem do Rio Uraricoeira, margem sul da Ilha de Maracá, 16 May 1987, *Lima & Nelson 752* (K, MO); Costa Marques, Rio Cauterinho along hwy. BR 429, 200 m, 23 Mar 1987, *Nee 34455* (K, US); Ilha 7 de setembro, Ponto III, 13 Oct 1986, *Toledo et al. 246* (F); Mineração Campo Novo BR-421 a 2 km a oeste da Mineração Campo Novo al 20 km de Ariquemes WSW, 14 Oct 1979, *Vieira et al. 462* (K, MO, US); **Roraima**: Alto Alegre, SEMA Estação, Ilha de Maracá, Furo Paraná de Firmino of Rio Uraricuera on S side of island. Within 1 km of end of Nova Olinda, 12 Jun 1986, *Hopkins et al. 674* (GH, K, US); SEMA Ecological Reserve, Ilha de Maracá, Roraima. Ilha de Nova Olinda, 4 Jul 1987, *Milliken & Bowles 400* (K); rodovia Perimetral Norte, Igarapá Paruana, leste de Caracaraí, 1 Jul 1974, *Pires & Leite 14846* (US).

**Colombia**. **Amazonas**: Puerto Nariño, Río Amazonas about 2 km downstream from Puerto Nariño, 28 Jan 1969, *Plowman et al. 2422* (ECON, GH); **Antioquia**: near Río León approx. 20-30 km upstream and south of the river mouth and approx. 15 km west of Chigorodó, 100 m, 16 Mar 1962, *Feddema 1936* (US); Río Palmas, Río Dulce, 1500 m, *Lehmann 7279* (K); Providencia, Anorí, slopes above forest road Providencia-Ahibe, secondary forest along Buenos Aires river, 500 m, 30 Apr 1973, *Soejarto 3965* (F, GH); **Boyacá**: Reg. of Mt. Chapon, extreme western part of Dept. Boyacá, north-west of Bogota, 1932, *Lawrance 400* (F); **Caldas**: Santa Cecilia, Tatamá, 800 m, 24 Nov 1945, *Sneidern 5006* (F, S, US); **Cauca**: Tambo, west flank of Cordillera Occidental, 1500 m, 12 Nov 1946, *Haught 5248* (US); **Chocó**: Río Chintado, 1–2.5 hours above La Nueva, 6 Feb 1967, *Duke 9868* (US); Río Atrato, 2-5 hours below Río Sucion above Loma Teguerre, 16 May 1967, *Duke 11011* (US); Río Truando, gallery between the boom (bun) and Río Salado, 18 May 1967, *Duke 11165* (US); **Cundinamarca**: Pacho-Paime Highway, 2200 m, 13 Aug 1947, *Haught 6071* (US); **Guaviare:** Río Guaviare, (parte alta), 9 Nov 1939, *Cuatrecasas 7579* (F, US); **Magdalena**: Campano, Sierra Nevada de Santa Marta above Minca [transect 3], 1680 m, 16 Jan 1989, *Gentry & Cuadros 64773* (MO); **Meta**: Villavicencio, 700 m, 2 Jan 1876, *André 805* (K); Villavicencio, Apai, 500 m, 12 Nov 1938, *Cuatrecasas 4783* (US); **Nariño**: Reserva Natural La Planada, 7 km above Chucunés on road between Tuquerres and Ricaurte, along Sendero La Vieja, 1780 m, 7 Mar 1990, *Croat 71158* (MO); **Putumayo**: Comisaria de Putumayo: vertiente oriental de la cordillera, bosques entre Mocoa y Sachamates, 29 Dec 1940, *Cuatrecasas 11421* (F, US); Umbría, 325 m, Oct 1930, *Klug 1818* (A, BM, F, GH, S, US); **Tolima**: Über Ligause, Dec 1882, *Lehmann 2318* (BM); **Valle del Cauca**: inter Tolima & Cali, *André 2613* (K); Cordillera Occidental; vertiente occidental; hoya del río Sanquininí, lado izquierdo, La Laguna, 1250 m, 10 Dec 1943, *Cuatrecasas 15693* (F, US); Monte El Tabor, Cordillera occidental; filo de la cordillera sobre Las Brisas, 1970 m, 19 Oct 1946, *Cuatrecasas 22406* (F, US); Cordillera Occidental, vertiente occidental, hoya del río Digua, Río San Juan, abajo de Queremal a la derecha del río entre km 52 y 53, 1300 m, 19 Mar 1947, *Cuatrecasas 23881* (CORD, F); La Cumbre, Cordillera Occidental, 1500 m, 11 Jul 1922, *Hazen 11836* (GH, US); La Cumbre, Cordillera Occidental, 14 May 1922, *Pennell 5716* (GH, US).

**Costa Rica**. **Puntarenas**: Cantón de Puntarenas, R.B. Monteverde, Cordillera de Tilarán, Piedades Norte, Burial, Reserva Biológica de Oberdorsf, 1500 m, 6 Sep 1993, *Bello & Cruz 5347* (BM); Monte Verde area, valley of Río San Luis just S of Monte Verde, 1000 m, 18 Jun 1985, *Hammel & Haber 13920* (BM); Reserva Forestal Golfo Dulce Osa Península, Rancho Quemado, ca 15 km W of Rincón, in bottom of valley along Río Riyito near bridge and in forest along road on ridge above valley, 250 m, 31 May 1988, *Hammel et al. 16925* (BM).

**Ecuador**. **Carchi**: Cantón Tulcán, Reserva Indígena Awá, Parroquia Tobar Donoso. Centro El Baboso, 1800 m, 17 Aug 1992, *Tipaz et al. 1813* (BM); **Esmeraldas**: Cantón San Lorenzo, Reserva Étnica Awá, Parroquia Alto Tambo, centro de la Union. Cañon del Rio Mira, 250 m, 22 Mar 1993, *Aulestia & Aulestia 1395* (MO); Cantón Quinindé, Bilsa Biological Station, Montañas de Mache, 35 km W of Quinindé, 5 km W of Santa Isabel, old Mono road near SE ridge, 400 m, 20 Oct 1994, *Bass et al. 179* (BM); Cantón Quinindé, Bilsa Biological Station, Mache Mountains, 35 km W of Quinindé, 5 km W of Santa Isabel, 8 km southwest of reserve along old Mono road, 400 m, 21 Nov 1994, *Clark & Bergman 316* (BM, US); Cantón Quinindé, Bilsa Biological Station, Montañas de Mache, 35 km W of Quinindé, 5 km W of Santa Isabela, on recently logged property of Sr. Ríos, along old Mono road, 400 m, 20 Oct 1994, *Pitman & Bass 872* (BM, F); **Los Ríos**: Río Palenque Science Center, km 56 Rd Quevedo-Sto Domingo, 150 m, 5 Feb 1979, *Dodson et al. 7568* (F); Río Palenque Field Station, halfway between Quevedo and Santo Domingo de los Colorados, 200 m, 23 Feb 1974, *Gentry 10138* (MO); **Morona-Santiago**: Cantón Taisha, Taisha, 457 m, 15 Feb 1952, *Cazalet & Pennington 7793* (B, K, US); Cantón Morona, Cordillera del Cutucú, Associacion Shuar Sevilla, Comunidad Angel Ruby, pie del Cerro Muchin, 1050 m, 8 Jun 2002, *Suin et al. 1982* (MO); **Napo**: Jatun Sacha Biological Station, 450 m, 12 Jun 1995, *Acevedo & Cedeño 7296* (US); Talag, 15 km SSW from Tena, Cerro Antisana, 609 m, 11 Jul 1960, *Grubb et al. 120* (K); Shinguipino Forest, between Rios Napo and Tena, 8 km SE of Tena, Cerro Antisana, 442 m, 20 Sep 1960, *Grubb et al. 1645* (K); Cantón Quijos, Reserva Ecológica Antisana, Cordillera de los Guacamayos, cruce del oleoducto de la compañia ARCO, entre El Mirador y camino de La Virgen, 2300 m, 12 Jan 1999, *Vargas & Narváez 3582* (BM, MO); **Orellana**: Puerto Francisco de Orellana (Coca), aprox. 40 km SE of the town (Auca oil field), 300 m, 4 Nov 1976, *Balslev & Madsen 10579* (MO, US); Armenia Vieja Río Napo, ca. 12 km SW of Coca (Puerto Francisco de Orellana), 12 Jan 1973, *Lugo S. 2679* (K); carretera Hollin-Loreto-Coca, km 60, 1000 m, 10 Dec 1987, *Neill et al. 8063* (K, MO); Cantón Aguarico, south slopes of Volcán Sumaco. 5 km east of Huamaní, Ridge above west side of valley of Río Pucuno, new road to Galeras under construction, 1100 m, 19 Oct 1989, *Neill & Palacios 9111* (BM); Orellana, Maxus petroleum pipeline road, under construction, 2 km south of Río Napo, Comuna Pompeya, 220 m, 4 Dec 1992, *Neill et al. 10189* (BM); Orellana, Yasuní, Estación Cientifica Yasuní, 25 Jun 2001, *Persson et al. 4488 A* (BM, MA); **Pastaza**: 10-20 km N of Canelos, 12 Nov 1974, *Lugo S. 4573* (K); Veracruz, Indillama, 22 Nov 1974, *Lugo S. 4640* (K); Hacienda San Antonio de Baron von Humboldt, 2 km al NE de Mera, 1100 m, 27 Feb 1985, *Neill et al. 6075* (BM, MO); Cantón Mera, Mera, 2 km al NE, Hacienda San Antonio de Baron von Humboldt, 1100 m, 20 Feb 1985, *Palacios et al. 00026* (US); Cantón Pastaza, Pozo petrolero Ramirez, 20 km al sur de la población de Curaray, 300 m, 21 Feb 1990, *Zak & Espinoza 5034* (BM, K, MEXU); **Pichincha**: Santo Domingo de los Colorados, 20 km W of town, 304 m, 20 Oct 1961, *Cazalet & Pennington 5093* (B, US); ca. 35 km N of Santo Domingo de los Colorados, vicinity of bridge over Rio Blanco, 250 m, 3 Feb 1974, *Gentry 9607* (MO); **Zamora-Chinchipe**: Cantón Nangaritza, Parroquia Zurmi, Comunidad Centro Shaime (along Río Nangaritza). Forest 2-4 km NW of Centro Shaime, 1000 m, 13 Dec 2001, *Clark et al. 6465* (QCNE, US).

**French Guiana**. Akouba Booka goo Soula, Camp #3, bassin du Haut Marouini, 160 m, 9 Sep 1987, *Granville et al. 10046* (US); Haut Litany,15 km en amont de la Koulé-Koulé, monts Tumac Humac, 160 m, 29 Jul 1993, *Granville et al. 11875* (B); Crique Tamanoir, Riviere Mana, 18 Aug 1962, *Hallé 573* (P); Crique Tamanoir, Riviere Mana, 20 Aug 1962, *Hallé 597* (P); Cayenne, *Poiteau* s.n. (W); fluminis La Manaduti, Jul 1824, *Poiteau* s.n. (K); La Mona, *Poiteau* s.n. (LE); Saül, Layon La Fumée, 13 May 1986, *Prévost 2137* (K); Saint-Laurent-du-Maroni**,** Acarouany, May 1898, *Sagot 459* (BM, G, K, S, W).

**Guyana**. North-West, Barima River, 19 Mar 1923, *Cruz 3420* (F, GH, US); Montagne de la Trinité, zone sud, Bassin de la Mana, 100 m, 18 Jan 1998, *Granville & Crozier 13686* (B, K); Mt. Russell District, Mar 1886, *Jenman 2096* (K); Hoobaloo Creek, May 1897, *Jenman 7237* (K); Pomeroon Rover, Jan 1904, *Jenman 7824* (K); Berbice River, New Dageraad, 4 Oct 1981, *Maas et al. 5534* (K, S); Oronogue, New River, 25 Dec 1935, *Myers 5905* (K x2); Oronogue, New River, 25 Dec 1935, *Myers 5905* (K); Demerara, *Parker* s.n. (K); **Barima-Waini**: Waini River, Marabo Shortcut, 2 Feb 1922, *Cruz 1284* (F, GH, US); Waini R., NW District, 4 Apr 1923, *Cruz 3583* (F, GH, US); Assakatta, NW District, 9 Sep 1923, *Cruz 4289* (US); Morawhanna, vic., Barima R, 1 Jan 1920, *Hitchcock 17487* (GH, S, US); Upper Sebai River; 8 km upriver from Sebai Village, 12 Dec 1991, *Hoffman et al. 635* (US); **Demerara-Mahaica**: Hyde Park, 1922, *Warren* s.n. (F); **Northwest Distr.**: Mabaruma-Aruka River, 8 Mar 1945, *Fanshawe 5112* (K); **Pomeroon Distr.**: Kabakaburi, 10 Feb 1923, *Cruz 3261* (K); **Pomeroon-Supenaam**: Pomeroon R, 12 Dec 1922, *Cruz 3226* (GH, US); Kabakaburi, 2 Feb 1923, *Cruz 3261* (F, GH, US); Red Lock ± 5 km WSW of Anna Regina, 4 Apr 1989, *Gillespie & Persaud 1112* (US); Pomeroon River watershed; Issororo River, 9-10 km W of confluence with Pomeroon River, 9 Sep 1992, *Hoffman & Roberts 2673* (US); **Potaro-Siparuni**: Iwokrama Rainforest Reserve, Burro-Burro R. betw. Ounari Rapids and confluence with Siparuni R, 60 m, 9 Sep 1995, *Clarke 288* (US); Iwokrama Rainforest Reserve, Burro Burro River, between Sandstone & confluence of Sipariparu R, 65 m, 3 Mar 1996, *Clarke 1552* (US); Iwokrama Rainforest Reserve, Iwokrama Mts., 0-1 km SE of camp at bottom of gorge, 75 m, 3 Mar 1997, *Clarke et al. 4235* (US); along Essequibo River, upstream from Kurupukari Falls, from 1–3 km S of falls, Iwokrama, 61 m, 9 Sep 1990, *McDowell 3338* (US); Iwokrama Reserve, Essequibo River, Lady Smith Creek, 50 m, 2 Feb 1995, *Mutchnick 857* (US); **Upper Takutu-Upper Essequibo**: Dadanawa, vic., upper Rupununi R, 5 May 1922, *Cruz 1392* (F, GH, US); South Rupununi Savanna, savanna-forest interface ca. 12 km S of Aishalton along road to Marudi, 200 m, 10 Oct 1993, *Henkel et al. 3418* (US); Cool-wind Mt. (Wadi-di-awar), Kanuku Mts, 500 m, 2 Feb 1985, *Jansen-Jacobs et al. 364* (US); Kuyuwini Landing, Rupununi District, Kuyuwini River, 150 m, 30 Oct 1992, *Jansen-Jacobs et al. 3187* (B, F, K, US); along Essequibo River, between Cashew Falls and Apoteri, 68 m, 9 Sep 1990, *McDowell 3391* (US).

**Panama**. **Coclé**: Road from La Pintada to Coclesito, 600 m, 7 Feb 1983, *Hamilton & Davidse 2875* (MO); **Cuna Yala**: San Blas, El Llano-Cartí Road, 19.1 km from Interamerican Highway, 350 m, 5 Mar 1985, *Nevers et al. 4971* (MO).

**Paraguay**. Paraguaria centralis, 1897, *Hassler 3826* (BM); **Chaco**: Santa Rita, orilla del monte cerca de la costa del rio, 29 Mar 1917, *Rojas 2437* (MO); **Paraguarí**: Estero del Ypoa, 29 km W of Carapegua, W of Pacheco, 13 Jan 1990, *Zardini & Velázquez 17492* (G, MO); **Presidente Hayes**: Rio Negro on route to Fortin General Bruguez, 25 Jul 1995, *Zardini & Pietrobón da Silva 43177* (MO); **San Pedro**: Primavera, Alto Paraguay, bordering Rio Tapiracuai, 11 Sep 1957, *Woolston 873* (S, US).

**Peru**. **Amazonas**: Río Cenepa, ridge above Quebrada Chikisinuk, a tributary of Huampami, entering from S about 5 km from confluence with Cenepa, 268 m, 21 Dec 1972, *Berlin 648* (MO, W); Bagua, Yamayakat, Quebrada Kusú, Dist. Imaza, 130 m, 15 Nov 1990, *Díaz et al. 4131* (MO, USM); Bagua, Cerro Apág, comunidad Aguaruna Kusú-Listra, margen derecha de Quebrada Kusú, 600 m, 15 Sep 1996, *Díaz et al. 8140* (MO, USM); Condorcanqui, Rio Cenepa, Rio Cenepa region, orilla de Quebrada Huampami, 18 Jan 1973, *Kayap 154* (MO); Condorcanqui, Rio Santiago, Valle del Rio Santiago, Quebrada Caterpiza, 2-3 km atrás de la comunidad de Caterpiza, 180 m, 1 Jan 1980, *Tunqui 521* (MO); Luya, Tullanya, Quebrada San Francisco, Dist. Camporedondo, 1700 m, 29 Nov 1996, *Vasquez & Rojas 21893* (MO, USM); **Cusco**: Cusco, Campamento Armihuari, Camisea Production Unit, Dist. Camisea, 469 m, 28 Jan 1997, *Acevedo et al. 9255* (US, USM); Cusco, Camisea, Campamento Segakiato, 5 km downriver from Community Segakiato, SI-MAB plot Segakiato, 1400 m, 1 Oct 1997, *Acevedo et al. 10070* (F, K, US); La Convención, Armihuari Sur, Dist. Echarate, 478 m, 10 Feb 2011, *Huamán & Delgado 483* (USM); La Convención, Armihuari, Río Camisea, Dist. Echarati, 535 m, 11 Oct 1998, *Núñez V. et al. 24183* (US, USM); Quispicanchis, San Lorenzo Tiwantari, 550 m, 15 Oct 1960, *Vargas 13476* (US); **Huánuco**: Leoncio Prado, La Divisoria, Plantación Margarita, 1500 m, 15 Aug 1946, *Ferreyra 1022* (US, USM); Leoncio Prado, La Divisoria, 21.8 km E of Puente Pumahuasi (Río Tulumayo) on raod from Tingo Maria to Pucallpa, Dist. Hermilio Valdizán, 1550 m, 27 Dec 1981, *Plowman & Schunke Vigo 11718* (F, USM); Tazo Grande, Monzon River, 893 m, 20 Sep 1965, *Schunke Vigo 865* (F, G, US); Leoncio Prado, Tingo Maria, to the west, 675 m, 19 Sep 1964, *Schunke Vigo 6617* (US); Divisoria, 1700 m, 18 Sep 1946, *Woytkowski 34550* (BM, F); **Junín**: Tarma, Chanchamayo Valley, 1500 m, Nov 1929, *Schunke 135* (F); Chanchamayo, Pampatigre, Fondo Romero, above Santa Ana, SE of La Merced, 1500 m, 7 Mar 1985, *Stein & Todzia 2343* (MO, USM); **Loreto**: Alto Amazonas, Andoas, Río Pastaza near Ecuador border, 230 m, 17 Nov 1979, *Gentry & Díaz 28266* (F, MO); Maynas, Yanomono, Explorama Tourist Camp, 120 m, 19 Feb 1981, *Gentry et al. 31481* (MO); along Río Marañon, near mouth of Río Tigre, 115 m, 19 Aug 1929, *Killip & Smith 27539* (US); Río Morona, lower Marañon valley, 150 m, 20 Aug 1929, *Killip et al. 29164* (US); Mishuyacu, near Iquitos, 100 m, Oct 1929, *Klug 484* (F, US); Florída, Río Putumayo, at mouth of Río Zubineta, 200 m, Mar 1931, *Klug 2071* (A, BM, F, GH, S, US); Maynas, Isla Rondiña, opposite Leticia, Río Amazonas, 18 Mar 1977, *Plowman et al. 6399* (GH, USM); Iquitos, Maynas, Rio Amazonas, Cotillo Islas, in front of Padre Isla, 1 Jun 1978, *Rimachi Y. 3615* (MO, US); Maynas, Río Momón, trocha del caserio de Balcon al caserio de Porvenir, Dtto. Iquitos, 7 Nov 1985, *Rimachi Y. 8110* (MO, USM); Alto Amazonas, Pongo de Manseriche, Dist. Manseriche, 650 m, 25 Nov 1997, *Rojas et al. 681* (MO, USM); Maynas, Gamitana Cocha, Río Mazán, 10 Mar 1935, *Schunke Vigo 357* (A, F, US, USM); Maynas, Esperanza, Río Tahuayo, 140 m, 26 Jan 1981, *Vasquez & Jaramillo 1273* (MO, USM); Alto Amazonas, Capuhari Sur, Campamento Petrolero, 200 m, 25 Mar 1982, *Vasquez et al. 3045* (MO, USM); Alto Amazonas, Cerros Campanquiz, 22 km S of La Vista, 850 m, 12 Feb 1978, *Wasshausen & Encarnación 1012* (USM); **Madre de Dios**: Tambopata, Santuario Nacional Pampas del Heath, Rio Heath, Pto. San Antonio, 210 m, 15 Sep 1996, *Aguilar & Castro 1085* (MO); Tambopata, Quebrada Loboyoc, Dist. Las Piedras, 161 m, 21 Oct 2005, *Farfán et al. 773* (USM); Manu, Parque Nacional Manu, Cocha Cashu Biological Station, 20 Aug 1976, *Foster & Augspurger 3264* (F, US, USM); Manu, Parque Nacional Manu, Cocha Cashu, in vicinity of ox-bow lake of Río Manu, between Panuaga and Tayakome, 17 Aug 1974, *Foster et al. 3365* (USM); Manu, Río Cumerjali, Río Manu, Parque Nacional Manu, 350 m, 25 Oct 1986, *Foster & D’Achille 12047* (USM); Río Madre de Dios, small tributary 1 hour below Puerto Maldonado, 250 m, 22 Apr 1977, *Gentry et al. 19623* (F, MO, USM); Tambopata, Las Piedras, Cusco Amazónico, 200 m, 6 Dec 1991, *Timaná 3659* (MO); **Pasco**: Oxapampa, Pichis Valley, near Paujil, 10 km downriver from Puerto Bermúdez, E side of river across from big bend with large island, 300 m, 24 Sep 1982, *Foster 8900* (MO, USM); Pasco, Río Paucartambo, 30 km SW of Oxapampa, 1860 m, 31 Dec 1972, *Madison 963* (GH); Oxapampa, Pozuzo, Distrito Pozuzo, Puesto de vigilancia Huampal, 1250 m, 23 Sep 2002, *Monteagudo et al. 3986* (BM, MO); Oxapampa, Distrito Huancabamba, Sector Grapanazu, limite Parque Nacional Yanachaga-Chemillen, 2210 m, 15 Oct 2003, *Rojas et al. 1795* (BM, MO); Oxapampa, Comunidad Nativa Alto Lagarto, reserva Comunal Yanesha, Dist. Palcazu, 500 m, 5 Oct 2008, *Rojas & Ortiz 6179* (USM); Oxapampa, Parque Nacional Yanachaga-Chemillén, sector San Alberto, 2200 m, 20 Jan 2003, *Vasquez et al. 27834* (USM); **Piura**: Piura, Montaña de Cuyas, 8 km NE of Ayabaca, transect 1, 2410 m, 25 Sep 1991, *Gentry et al. 75070* (MO, USM); **San Martín**: Lamas, Alonso de Alvarado, Plantano yacu, carretera a Moyabamba, 800 m, 23 Apr 1973, *Schunke Vigo 6015* (US); Tarapoto, Cerro Campana, Dec 1855, *Spruce 4327* (BM, K, W); Rioja, Bosque de Protección, Dist. Nuevo Cajamarca, cerca del Poblado Palestina, camino al la Cueva Palestina, 890 m, 1 Nov 1996, *Sánchez Vega & Dillon 8401* (BM); Rioja, Dist. Pardo Miguel, margen izquierda del Río Serranoyacu, 1250 m, 2 Jul 1999, *Sánchez Vega et al. 9983* (BM); **Ucayali**: Requena, Río Ucayali; Supay Forest Reserve, Jenaro Herrera, 20 Feb 1987, *Gentry et al. 56194* (MO, USM); Purús, Puerto Esperanza, al este del aeropuerto, Dtto. Purús, 190 m, 20 Mar 2002, *Schunke Vigo & Graham 15118* (USM); Coronel Portillo, Sacarita del Río Utiquinia, margen izquierda del Río Utiquinia, Dist. Calleria, 150 m, 24 Mar 2003, *Schunke Vigo & Graham 15403* (BM, USM); Coronel Portillo, Quebrada Pumayacu, margen izquierda del Río Utiquinia, 150 m, 31 Mar 2003, *Schunke Vigo & Graham 15474* (USM); Coronel Portillo, Yarinacocha, Caño a Pucallpa, 250 m, 28 Mar 1981, *Vasquez & Jaramillo 1533* (CORD, F, MO).

**Surinam**. Sipaliwini, vicinitiy of airstrip along Ulemari River, 71 km up Ulemari River from its confluence with Litani River, 150 m, 1 May 1998, *Evans &Peckham 2968* (US); Paramaribo, *Focke 57* (L); Haut Litany, Litany River, confl with Koule-Koule, Monts Tumuc-Humac, 160 m, 7 Jul 1993, *Granville et al. 11875* (K, US x2); along Ulemari River, ca. 13 km upstream from its confluence with Litani River, 150 m, 3 Apr 1998, *Hammel et al. 21289* (BM); Brokopondo, NW Brokopondo Stuwmeer Lake, SE of Brownsberg Nature Reserve, mouth of Whitey Creek, 15 m, 2 Feb 1998, *Hoffman 5268* (US); Sipaliwini, Jacob Kondre Village, 1-2 km S of village on Saramacca River, 40 m, 7 Jul 2000, *Hoffman 5468* (US); Lucie River, 2-10 km below confl. with Oost River, Wilhelmina Gebergte, 225 m, 9 Sep 1963, *Irwin et al.55565* (K x2, US); Marowijne, Nassau Mountains, Plateau C, 500 m, 26 Jan 2003, *Jansen-Jacobs et al. 6290* (US); ad aquas pr. u. Paramaribo, Apr 1844, *Kappler 1599* (G, W); Marieparten, 1857, *Kegel 1294* (GOET); Lely Mts., SW plateaus, 550 m, 18 Sep 1975, *Lindeman et al. 23* (K, MO); Nickerie, Kabalebo Dam project area, 30 m, 9 Sep 1980, *Lindeman et al. 80-528* (US); Nickerie, area of Kabalebo Dam project, bank of Baroeba creek near road km 113, 21 Sep 1980, *Lindeman et al. 528* (S); Kwatta, Paramaribo, 7 Jun 1916, *Samuels* s.n. (US); Groningen, Station, 10 May 1916, *Samuels 63* (L); Pl. Pietersburg, May 1850, *Wullschlägel 373* (GOET, W).

**Trinidad and Tobago**. **Trinidad**: near Siparia quarry, 8 Apr 1921, *Britton & Broadway 2820* (K).

**Venezuela**. **Amazonas**: Manapiare, San Juan de Manapiare, 90 m, 25 Jun 1998, *Fernández 12992* (BH); Brazo Casiquiare, Capibara, 2 Feb 1931, *Holt & Blake 651* (US); rapids of Trapichote, Delta of Ventuari, 126 m, 20 Apr 1942, *Llewelyn 14985* (F); Río Negro, Cerro de La Neblina, Neblina Massif, bongo (dugout) trip along Rio Mawarinuma downstream in NW direction from base camp at mouth of canyon for approx. 4 km, 140 m, 4 Apr 1984, *Stannard 515* (K); a lo largo del rio Coro-Coro, río abajo de la pista de aterrizaje de Yutaje, 150 m, 30 Apr 1978, *Steyermark & Redmond 117089* (F); between Paso el Diablo and Caño de Culebra; 25-30 km SE Puerto Ayacucho, 100 m, 5 May 1980, *Steyermark et al. 122314* (MO, US); **Aragua**: PN Henri Pittier, road Maracay to Ocumare, km 28, ca. 1.5 km above the road, 785 m, 3 Apr 1990, *Edwards et al. 399* (K); Ocumare valley, 3 Apr 1926, *Pittier 12159* (US); Choroní valley, 18 Feb 1937, *Pittier 13917* (US); entre Rancho Grande y la Regresiva, 1000 m, 5 Apr 1947, *Pittier 15376* (US); **Bolívar**: Rio Icutu (Rio Nichare), from 4 km upstream of Icutu Village, 1000 m, 20 Aug 1985, *Horner et al. 418* (MO); Cerro Bolívar, between Pilot Plant and Tunnel E-4, 28 Feb 1953, *Maguire & Wurdack 34446* (MO); Río Chiguao, “El Araguaney”, 5 May 1987, *Stergios 10995* (US); Rio Caura, a la altura del raudal Sejato, 9 May 1988, *Stergios & Delgado 12972* (MO); Matacuchillo, vecindades; 22-25 kilometros oeste-suroeste del aeropuerto de Santa Elena, cerca de los limites entre Venezuela y Brasil, 920 m, 8 Aug 1976, *Steyermark et al. 112281* (US); **Carabobo**: a lo largo de las cabeceras del rio San Gián, arriba de La Toma, al sur de Borburata, 750 m, 30 Mar 1966, *Steyermark & Steyermark 95331* (B, F, GH, K, S, US); Borburata, Feb 1942, *Tamayo 2216* (US); **Delta Amacuro**: Curiapo, 12 Dec 1952, *Gines 4965* (US); Río Cuyubini, vic. of sawmill, between mouth of Río Cuyubini and first main fork at Hacienda Caicarocoro, 90 m, 11 Nov 1960, *Steyermark 87535* (US); **Trujillo**: Boconó, Parque Nacional Guaramacal, Parque Nacional Guaramacal, near Quebrada Honda, 1900 m, 28 Dec 2000, *Dorr & Stergios 8764* (BM); **Zulia**: Lagunillas, cuenca del Embalse Burro Negro (Pueblo Viejo): laderas occidentales de la Serrania de Ziruma o El Empalado, a lo largo del rio Grande, unos 13 km al norte del Embalse, 550 m, 1 Apr 1982, *Bunting et al. 11152* (MO); faldas inferiores, a lo largo de la Quebrada Perayra, afluente del Rio Tocucu, suroeste de la Mision del Los Angeles de Tocucu (Tocucu), al suroeste de Machiques, 450 m, 29 Aug 1967, *Steyermark 99869* (MO, US).

#### 43. Solanum valdiviense

Dunal, Prodr. [A.P. de Candolle] 13(1): 195. 1852

http://species-id.net/wiki/Solanum_valdiviense

Figure 104

Solanum evonymoides J. Rémy in Gay, Fl. Chil. 5: 81. 1849, non *Solanum evonymoides* Sendtn., 1846. Type: No specimens cited (possibly based on the same specimens as *Solanum valdiviense*, but evidence equivocal, see discussion).
Solanum spiraeoides Dunal, Prodr. [A.P. de Candolle] 13(1): 157. 1852. Type: Chile. Región XIV (Los Ríos):Valdivia, *C. Gay 674* (holotype: P [P00371677]).
Solanum subenervium Dunal, Prodr. [A.P. de Candolle] 13(1): 104. 1852. Type: Chile. Región V (Valparaíso): “Valparaíso”, 1831-1833, *C. Gaudichaud 169* (holotype: P [P00371844]).
Solanum cyrtopodium Dunal, Prodr. [A.P. de Candolle] 13(1): 195. 1852. Type: Chile. Sin. loc., 1830, *E. Poeppig 714* (holotype: G-DC [G00145775, F neg. 6762, IDC microfiche 800-61.2077:III.1]); isotypes: BM [BM000935957], P [P00369231, Morton neg. 8306]).
Solanum puberulum Phil., Linnaea 29: 22. 1857-1858. Type: Chile. Región VIII (Bío-Bío): Andes of Chillán, *Germain* s.n. (specimens not traced; synonymy ex descr.).
Solanum krauseanum Phil., Linnaea 33: 204. 1864-1865. Type: Chile. Región XIV (Los Ríos): Valdivia, “prope Corral”, [1861], *H. Krause* s.n. (lectotype, designated here: SGO [SGO000004575]; isolectotypes: B [destroyed, F neg. 2734], CORD [CORD00004231], G [G00070189], GOET [GOET003595], K [K000585546], MA, W [1903_10265]).
Solanum sembarto Kuntze, Revis. Gen. Pl. 3(2): 227. 1898. Type: Based on *Solanum evonymoides* J.Rémy
Solanum sembarto Kuntze var. *varians* Kuntze, Revis. Gen. Pl. 3(2): 227. 1898. Type: Chile. Región IX (Araucanía): Prov. Malleco, Ercilla, *O. Kuntze* s.n. (lectotype, designated here: NY [NY00172171]).
Solanum sembarto Kuntze var. *pubescens* Kuntze, Revis. Gen. Pl. 3(2): 227. 1898. Type: Chile. Región IX (Araucanía): Prov. Malleco, Ercilla, Feb 1892, *O. Kuntze* s.n. (lectotype, designated here: US [US-701233]).

##### Type.

Chile. Región XIV (Los Ríos): Valdivia, Jan 1835, *C. Gay 212* (lectotype, designated here: P [P00335224, F neg. 39167]; isolectotypes: MPU, P [P00335225, P00335226]).

##### Description.

Lax shrub with arching branches, 1-3 m tall, suckering at the base. Stems glabrous to densely pubescent with uniseriate, simple or dendritic trichomes < 0.5 mm long, strongly ridged, the ridges pale; new growth sparsely to densely pubescent with simple or dendritic trichomes. Bark of older stems green to grey, the ridges paler. Sympodial units plurifoliate, the leaves often borne on short shoots. Leaves usually simple, highly variable in size and shape, on non-reproductive stems the leaves 3–6 cm long, 1–1.5 cm wide, lanceolate, occasionally with irregular lobes at the base, on reproductive stems the leaves more often elliptic, 0.9–1 cm long, 0.5–0.7 cm wide, membranous or somewhat fleshy, the upper surfaces glabrous to sparsely pubescent with simple or dendritic trichomes < 0.5 mm long, the lower surfaces glabrous to sparsely or densely pubescent with simple or dendritic trichomes like those of the upper surfaces; primary veins 2–7 pairs, not visible in elliptic leaves; base acute to truncate, in elliptic leaves more usually acute; margins entire, occasionally with one or two basal lobes in lanceolate leaves; apex acuminate to rounded in lanceolate leaves, acute to obtusely rounded in elliptic leaves; petioles 0.5–1 cm long in lanceolate leaves, 0.15–0.2 mm long in elliptic leaves, apparently not twining. Inflorescences terminal on short axillary shoots, 1–3 cm long, simple or occasionally once-branched, with 3–10 flowers clustered at tip, glabrous to densely pubescent with simple or dendritic uniseriate trichomes < 0.5 mm long; peduncle 1–3 cm long; pedicels 1–1.2 cm long, ca. 0.5 mm in diameter at the apex and base, filiform, nodding at anthesis, glabrous, sometimes tinged purple, articulated at the base in a short sleeve on a platform; pedicel scars short pegs clustered at the tips of inflorescence in a small group with the appearance of a platform. Buds ellipsoid, the corolla very exserted from the calyx tube before anthesis. Flowers all perfect, 5-merous. Calyx tube 1–1.2 mm long, conical, the lobes 0.5–1.5 mm long, deltate or quadrate, minutely apiculate, glabrous. Corolla 0.7–1.8 cm in diameter, white or purple, often white tinged with violet, stellate, lobed 3/4 of the way to the base, the lobes 3–5 mm long, 2–3.5 mm wide, strongly reflexed at anthesis, densely pubescent on the tips and distal lobe margins, otherwise glabrous. Filament tube minute, the free portion of the filaments 0.5–1 mm long, glabrous or minutely puberulent with simple trichomes; anthers 3–4 mm long, 1–1.5 mm wide, ellipsoid, loosely connivent, yellow, poricidal at the tips, the pores only partially lengthening to slits with age. Ovary glabrous; style 5–7 mm long, glabrous; stigma capitate, the surface minutely papillose. Fruit a globose berry, 0.5–0.7 cm in diameter, green or red when ripe, glabrous, the pericarp thin, shiny; fruiting pedicels 1.5–2 cm long, more or less woody, ca. 1 mm in diameter at the base, pendent. Seeds ca. 10 per berry, ca. 3 mm long, ca. 2 mm wide, flattened-reniform, reddish brown, the surface minutely pitted, the testal cells square. Chromosome number: not known.

**Figure 104.**
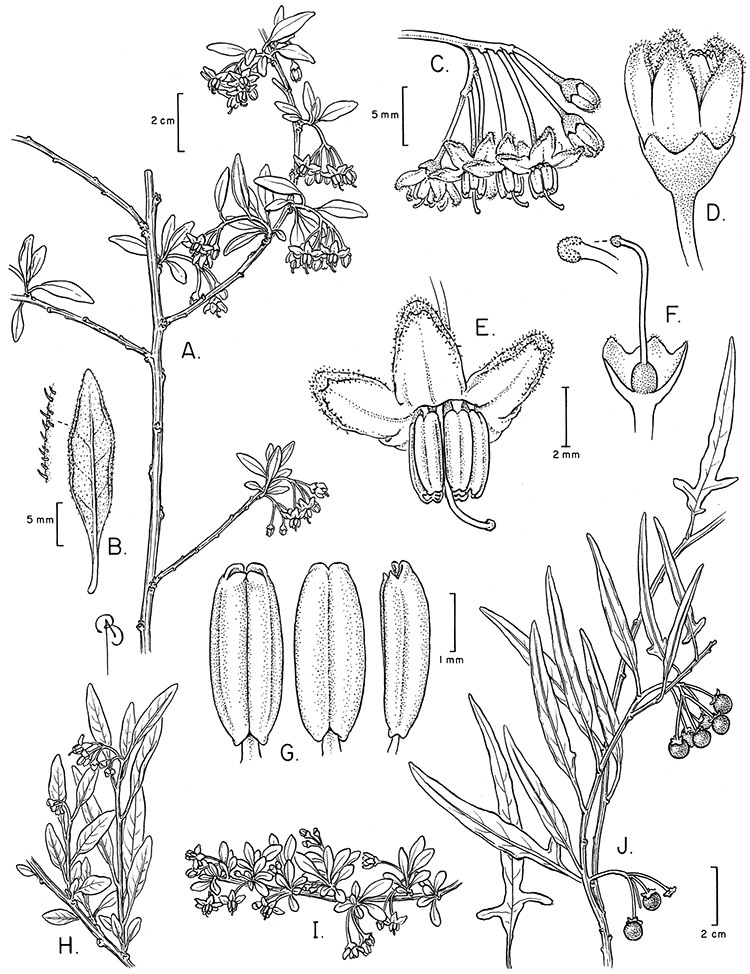
*Solanum valdiviense* Dunal. (**A–G** drawn from *Taylor et al. 10282* **H** drawn from *Taylor & Taylor 10843* **I** drawn from *Werdermann 323*). Illustration by Bobbi Angell.

##### Distribution

**Figure 105.**
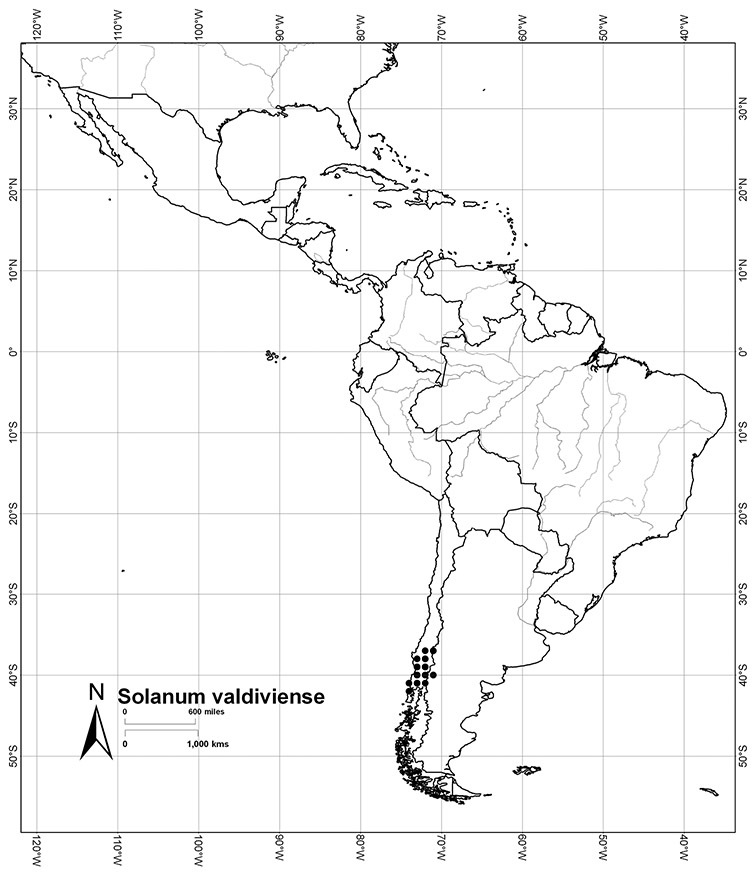
Distribution of *Solanum valdiviense* Dunal.

(Figure 105). *Solanum valdiviense* is found in southern Chile and adjacent Argentina, from 100–2000 m. The altitudinal range of *Solanum valdiviense* is from almost sea level to the high Andes and it is apparently relatively common where it occurs.

##### Ecology.

In *Nothofagus* (Nothofagaceae) forests and woods; cloud forests.

##### Common names:

Chile: huevil, llaguecillo (Muñoz-Pizarro 1966: 142); sembarto (Kuntze 1898: 227, said to come from a B sheet collected by “Oschenius” [B, now destroyed] “Sembarto ist der chilenische Name deiser Art nach Oschenius in einer Notiz zer einem exemplar im Berliner botanischen Museum” [Sembarto is the Chilean name for this plant as noted by Oschenius in a specimen from the Berlin Botanical Museum]).

##### Conservation status.

Least Concern (LC); EOO >50,000 km^2^ (LC) and AOO >10,000 km^2^ (LC). See Moat (2007) for explanation of measurements.

##### Discussion.

Leaf shape in *Solanum valdiviense* is incredibly variable, and ranges from lanceolate and sometimes basally lobed on non-reproductive (and some reproductive) shoots, to minute and almost orbicular or elliptic on reproductive shoots. Kuntze (1898: 227) stated “Es finden ausserdem sowohl bei α als bei β öfters zweierlei Blätter; oblonge stumpfe und acuminate etwas langere auf einer Pflanze” (On both sorts there are often two kinds of leaves, oblong and acuminate, on a single plant). Long sucker shoots invariably have lanceolate leaves, but reproductive shoots may have either type. Leaves of juvenile shoots are sometimes lobed at the base. This variability has led to the relatively many synonyms for this species of quite restricted range; for example, R.A. Philippi described *Solanum puberulum* on the basis of its leaf shape and pubescence.

No herbarium specimens were cited in the description of *Solanum krauseanum*, but Philippi (1864) did cite a collection from Corral (Valdivia) and attributed this to Krause. Because the species was published in the German periodical *Linnaea*, it has been assumed that the type was in Berlin (represented by the F neg. 2734), but it is more likely that it is in Santiago, where Philippi worked. I have therefore seleted the sheet in SGO as the lectotype of this species. Specimens identified as *Solanum krauseanum* are particularly weak and thin plants, and the leaves are membranous and more ovate than is usual. The stems, however, have the characteristic wings and pubescence and the flowers the strongly reflexed petals and ellipsoid anthers of *Solanum valdiviense*. On the destroyed B sheet (F. neg. 2734) the habit is recorded as scandent in trees (“in arbores scandens”).

The inflorescence in *Solanum valdiviense* is borne terminally on short axillary shoots (occasionally leaf opposed or the shoot much reduced), a character shared with the otherwise very different *Solanum inodorum* of southeastern Brazil. In many specimens, the leaves of the short shoots are smaller and more congested than those of the main stems, but not always. Rarely does the short shoot lack well-developed leaves; this leads to the plant having a bushy appearance. Pubescence is also quite variable in *Solanum valdiviense*, varying from nearly absent to dense (see Kuntze’s description of two pubescence varieties from specimens collected near Malleco). This variation does not seem to have an ecological basis, and is quite common in the Dulcamaroid clade in general. Flower color in *Solanum valdiviense* also varies from white to purple, again a common characteristic in the Dulcamaroid clade. The strongly reflexed petals are mentioned often on labels, and appear to be characteristic of *Solanum valdiviense*.

*Solanum valdiviense* could be confused with another species of the Dulcamaroid clade occurring in coastal Chile, *Solanum alphonsei*. *Solanum alphonsei* has consistently lobed leaves that are more deltate in outline, open, many-branched inflorescences and is usually a vine, rather than a lax shrub.

Two collections were cited in the protologue of *Solanum valdiviense*, one in Paris and the other in “herb. mihi” – Dunal’s own herbarium, now held in MPU. I have selected the sheet P00335224 as the lectotype, as it bears Gay’s own label with the annotation “esp. nueva” and a complete locality. It is possible that this same collection formed the basis for *Solanum evonymoides* J.Rémy, bearing in mind the “esp. nueva” annotation on Gay’s original label. There are no specimens cited in the protologue of *Solanum evonymoides* J.Rémy, so this is speculative; I have not typified *Solanum evonymoides* J.Rémy with this sheet, as it a later homonym of *Solanum evonymoides* Sendtn. of the Geminata clade (Knapp 2008) and is thus not available for use in any case.

Smaller leaved individuals of *Solanum valdiviense* have been called *Solanum evonymoides*, but that epithet is a homonym of *Solanum evonymoides* Sendtn., a member of the Geminata clade from southeastern Brazil (see Knapp 2008a). No specimens have been traced that can be definitively linked with Rémy’s protologue.

*Solanum cryptopodium*, a name attributed to F. Philippi in older editions of *Index Kewensis*, is a spelling mistake for *Solanum cyrtopodium* Dunal; Philippi did not cite a type as he did for other names and it is clear he was not intending a new name. Kuntze identified *Poeppig 63 [714]* as “*Solanum quadrifidum*” a name he never published (see P000369231); this name has occasionally appeared in lists. In describing the two varieties of *Solanum sembarto* (his replacement name for *Solanum evonymoides* J.Rémy), *varians* and *pubescens*, Kuntze cited a single collection from Ercilla. I have only found two sheets of this gathering, one at NY the other at US; the gathering consists of several stems with varying leaf shapes and pubescence densities all mounted together. The sheet at US (US-701233) is annotated “Solanum sembarto OK β pubescens OK” in Kuntze’s hand; I have selected this as the lectotype of var. *pubescens*, and the NY sheet (NY00172171), annotated only as “Solanum sembarto OK” as the lectotype of var. *varians*.

##### Specimens examined.

**Argentina**. **Neuquén**: región del Río Aluminé, 1 Apr 1902, *Asp 46* (SI); Pulmarí, 914 m, 2 Jan 1926, *Comber 371* (E, K); San Martín de los Andes, 731 m, 3 Nov 1926, *Comber 735* (E, K); Lácar, Cascada Maipú, San Martín de los Andes, 12 Dec 1946, *Dawson 1360* (US); Parque Nacional Lanín, NW Carrilafquen, 5 Mar 1968, *Eskuche 289* (SI); San Martin de los Andes, 1937, *Rasp 65* (SI); Lago Lacar, 1 Nov 1963, *Schajovsky* s.n. (SI); **Río Negro/Neuquén**: Nahuel Huapi, 1915, *Rothkugel* s.n. (SI).

**Chile**. **Región IX (Araucanía)**: Volcán Llaima, estacion de esqui Las Araucarias, 1170 m, 22 Dec 2001, *Aedo 7222* (MA); Temuco, 20 Oct 1918, *Brother Claude-Joseph 600* (US); Temuco, Oct 1927, *Brother Claude-Joseph 4837* (US x2); Malleco, Curacautín, Cordillera de los Andes, Parque Nacional Conguillío, above carpark at Laguna Verde, 1022 m, 25 Jan 2004, *Brownless et al. DCI-937* (BM, E); Cautín, Villarrica, road from Meseta San Judas to western edge of Lago Colico, 500 m, 20 Dec 2003, *Gardner & Knees 6726* (BM, E); Malleco, Cunco, Cordillera de los Andes, Reserva Nasampulli, 1146 m, 1 Jan 2004, *Gardner & Knees 6914* (BM, E); Cautín, Temuco, road to Cunco, ca. 33 km E of Temuco, 250 m, 25 Oct 1993, *Landrum & Landrum 7991* (MO); Malleco, Reserva Forestal Malleco, orillas del Río Niblinto, 940 m, 29 Oct 1977, *Marticorena & Quezada 1527* (B); Temuco, Maquehue, Oct 1905, *Middleton* s.n. (BM); Cautín, Temuco, Cerro Ñeilol, 150 m, 15 Oct 1957, *Montero 5203* (G); Cautín, Volcán Llaima, 1300 m, 7 Dec 1987, *Rechinger & Rechinger 64219* (B); Cautín, Volcán Villarrica, umbegung des Refugiums, 1250 m, 6 Dec 1987, *Rechinger & Rechinger 64135* (W); Cautín, Volcán Villarrica, 1750 m, 6 Dec 1987, *Rechinger & Rechinger 64160* (W); Cautín, Volcán Llaima, 1300 m, 7 Dec 1987, *Rechinger & Rechinger 64219* (W); Temuco, General Lopez, 106 m, Dec 1939, *Sandeman 356* (BM, K); Malleco, Termas de Tolguaca, 1160 m, 25 Jan 1979, *Solomon & Solomon 4478* (MO); **Región VIII (Bío-Bío)**: Biobío, entre Chillan y las Termas de Chillan, 1500 m, 28 Dec 1993, *Charpin et al. 23904* (G); Ñuble, Chillán, Cordillera de los Andes, Termas de Chillán, slopes below thermal springs, 1857 m, 28 Dec 2003, *Gardner& Knees 6853* (BM, E); Arauco, Reserva Forestal Pino Huacho, Cordillera de Nahuelbuta, 800 m, 14 Sep 1978, *Marticorena et al. 1610* (B); Coronel, 1866, *Ochsenius* s.n. (GOET); Coronel, 1864, *Oschenius* s.n. (GOET); Antuco, *Poeppig 63* (LE); Ñuble, E of Chillán from the Refugio El Aserradero of the Club Andino (at the Puente El Aserradero on the road to Termas de Chillán), 1250 m, 22 Nov 1990, *Taylor et al. 10282* (MO); Ñuble, Termas de Chillán, 1400 m, 20 Nov 1991, *Taylor & Taylor 10843* (MO); **Región X (Los Lagos)**: route Osorno-Bahia Mansa, km 31, 300 m, 10 Nov 1997, *Billiet & Jadin 6972* (MO); Castro, 17 Nov 1950, *Brooke 6971* (BM); Island of Quehui, 21 Nov 1868, *Cunningham* s.n. (K); Park Natural Alerce Andino, 40 km a l’E de Puerto Montt, 125 m, 31 Jan 1985, *Evrard 10597* (BM); Chiloé, Tinuquina, Tramahué, 18 Oct 1931, *Junge 54* (B, SI); Arique, Nov 1851, *Lechler 539* (B, G, LE, P, S); Island of Chiloé, *Miers 7886* (BM); Chiloé, Timiqui, nr. Tramahué, 18 Oct 1931, *Junge 54* (MO); Llanquihue, Maullin, Los Muermos, 19 Jan 1948, *Sparre 4024* (S); Osorno, Pauchue, 16 Jan 1947, *Wall 31* (S); Valdivia, Panguipulli, 180 m, Oct 1924, *Werdermann 323* (B, F, G, MO, SI, US); Llanquihue, near falls of Río Pilmaiquen, 45 km E of Osorno, 180 m, 6 Dec 1935, *West 4667* (MO); **Región XIV (Los Ríos)**: Corral, Cerro de la Marina, 80 m, 1 Dec 1937, *Andreas 197* (B, L); Panguipulli, Oct 1923, *Brother Claude-Joseph*, *2394* (US); Valdivia, Calle-calle, 25 Oct 1897, *Buchtien* s.n. (GOET, SI, S); Valdivia, 20 Sep 1904, *Buchtien* s.n. (B, G, US); Valdivia, road to Curiñanco which eventually turns off to Parque Nacional Oncol, 294 m, 18 Jan 2003, *Gardner et al. DCI-1* (BM, E); Corral, Nov 1969, *Hollermayer 1161* (LE); Valdivia, Panguipulli, 180 m, Oct 1924, *Hollermayer 323* (BM); Valdivia, fundo of the Universidad de Chile ca. 15 km N of Valdivia on road to Lanco, 19 Oct 1991, *Landrum & Donoso 7601* (MO); Valdivia, Parque Nacional Puyehue, 600 m, 5 Dec 1987, *Rechinger & Rechinger 64092* (W); Valdivia, Lago Riñihue, 9 Oct 1940, *Santesson 1119* (S).

#### 44. Solanum viscosissimum

Sendtn. in Mart., Fl. Bras. 10: 14. 1846

http://species-id.net/wiki/Solanum_viscosissimum

Figure 106

Solanum amplexicaule Sendtn. in Mart., Fl. Bras. 10: 14. 1846. Type: Brazil. Minas Gerais: Itambé, *J. Pohl* s.n. [*3627*] (lectotype, designated here: W [W-0001951]; isolectotypes: M [M0171805, Morton neg. 8701], W [W-0001950]).
Solanum jasminifolium Sendtn. in Mart., Fl. Bras. 10: 13. 1846. Type: Brazil. Minas Gerais: M. Morro, Villa Rica [Ouro Preto], *C. Martius 821* (lectotype, designated here: M [M0171829, F neg. 6536]).
Solanum cornigerum Dunal, Prodr. [A.P. de Candolle] 13(1): 75. 1852. Type: Brazil. Rio Janeiro: Novo Friburgo, Nov 1842, *P. Claussen 137* (holotype: P [P00325621, Morton neg. 8166]).
Solanum heteromorphum Dunal, Prodr. [A.P. de Candolle] 13(1): 80. 1852. Type: Brazil. Bahia: Tamandua, *J. Blanchet 3828* (lectotype, designated here: G-DC [G00144822, F negs. 6517, 6743; IDC microfiche 800-61.2067:III.5]; isolectotypes: G [G00070170, G00070171, G00301658], P [P00355664; P00355665, Morton neg. 8222]).
Solanum amplexicaule Sendtn. var. *pubescens* Glaz., Bull. Soc. Bot. France 58 (Mem. 3f): 491. 1911. Type: Brazil. Rio de Janeiro: Novo Friburgo, 18 Sep 1890, *A. Glaziou 18409* (lectotype, designated here: P [P00319628]; isolectotype: P [P00319629]).

##### Type.

Brazil. “Brasilia australis”, *F. Sellow* s.n. (holotype: B [F neg. 3191], destroyed; no duplicates found). Brazil. Santa Catarina: Mun. Ponte Serrado, near Ponte Serrada, 94 km west of Joaçaba, ca. 26°52'S, 52°05'W, 700-900 m, 15 Dec 1964, *L.B. Smith & R.M. Klein 14004* (neotype: HBR [HBR-31751]; isotypes: GH [GH00310386], NY [NY00669393], P [P000384880], US [US-2492240], WIS).

##### Description.

Woody vine with twining petioles, occasionally growing as an erect subshrub or herb. Stems strongly angled, sparsely to densely pubescent with transparent, simple uniseriate glandular-tipped trichomes of varying lengths, 0.5–2 mm long, most commonly 1–1.5 mm long, the glands unicellular; new growth sparsely to densely pubescent like the stems. Bark of older stems greenish brown, glabrescent. Sympodial units plurifoliate. Leaves simple to pinnatifid to deeply pinnate with up to 9 pairs of lobes, the lobes often not paired, (1-)5–8(-10) cm long, (0.5-)2.5–5.5(-9) cm wide, narrowly ovate or elliptic in outline, usually widest in the basal half in simple leaves, coriaceous or membranous, decreasing in size apically on the stems, the upper surfaces sparsely to densely pubescent with simple uniseriate trichomes to 2 mm long, these transparent and usually glandular, the lower surfaces glabrous (Rio de Janeiro) to pubescent with similar trichomes; primary veins 5–7 pairs, usually drying yellowish green; base cordate or truncate; margins entire and revolute or variously lobed, the lobes shallow to deep, the basal sinuses usually deeper, the edges of the margins usually ciliate with a fringe of simple uniseriate trichomes ca. 0.5 mm long; apex acute to acuminate; petioles from 0.2 cm long in distal simple leaves and these distal leaves often clasping the stem, to 5 cm long in larger leaves, sparsely to densely pubescent with simple uniseriate trichomes like those of the upper leaf surfaces, the longer petioles twining. Inflorescences terminal, later lateral, (1-)3–8(-14 +) cm long, simple to 4 times branched, with up to 25 flowers, sparsely to densely pubescent with simple uniseriate glandular trichomes like those of the stems; peduncle (0.5-)1.5–5(-11) cm long; pedicels 1.2–1.5 cm long, ca. 0.5 mm in diameter, filiform, nodding at anthesis, glabrous to pubescent with transparent simple uniseriate trichomes to 2 mm like those of the stems, these often glandular, articulated at the base from a tiny sleeve, leaving a minute peg on the inflorescence axis; pedicel scars irregularly spaced 3–9 mm apart. Buds globose when young and included in the calyx tube, later ellipsoid and the corolla strongly exserted. Flowers all perfect, 5-merous. Calyx tube 1.5–2 mm long, conical, the lobes 1.5–2 mm long, deltate to narrowly deltate, the sinuses splitting irregularly so the lobes irregular in shape and size, the tips elongate, sparsely to densely pubescent with simple uniseriate trichomes to 1.5 mm, these often glandular, the tips with a tuft of minute simple trichomes. Corolla 1.5–1.8 cm in diameter, white or violet, rotate-stellate, lobed 1/2 to 1/3 of the way to the base, the lobes 5–8 mm long, 3–5 mm wide, planar at anthesis, glabrous or with a few simple white trichomes scattered on the abaxial surface, densely papillate on the tips and margins. Filament tube minute, the free portion of the filaments 1–1.5 mm long, glabrous; anthers 3–4 mm long, 1–1.5 mm wide, ellipsoid, loosely connivent, poricidal at the tips, the pores lengthening to slits with age. Ovary glabrous; style 5–8 mm long, glabrous or puberulent in the basal half with tiny 1-celled simple trichomes ca. 0.2 mm long; stigma minutely capitate, the surface minutely papillose. Fruit a globose berry, 1–1.1 cm in diameter, purplish black when ripe, the pericarp thin and shiny, glabrous; fruiting pedicels 1–1.7 cm, ca. 1 mm in diameter, thicker towards the apex, somewhat woody, pendent. Seeds ca. 10 per berry, 3–3.5 mm long, 2–2.5 mm wide, flattened reniform, reddish brown, the surfaces minutely reticulate, the testal cells pentagonal, the lateral cell walls elongate and the seeds appearing pubescent in mature berries. Chromosome number: not known.

**Figure 106.**
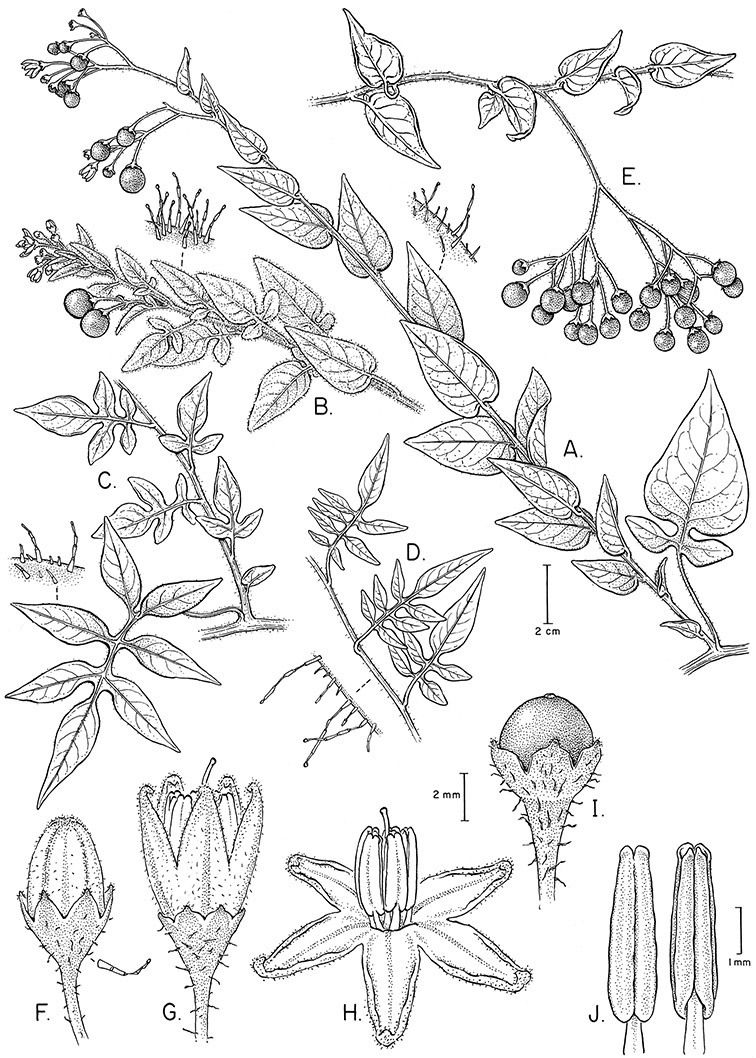
*Solanum viscosissimum* Sendtn. (**A, G, I** drawn from *Ratter 3430* **B** drawn from *Ribas et al. 1154* **C, H** drawn from *Irwin et al. 11355* **D** drawn from *Irwin et al. 18165b* **E, F** drawn from *Irwin 6221*). Illustration by Bobbi Angell.

##### Distribution

**Figure 107.**
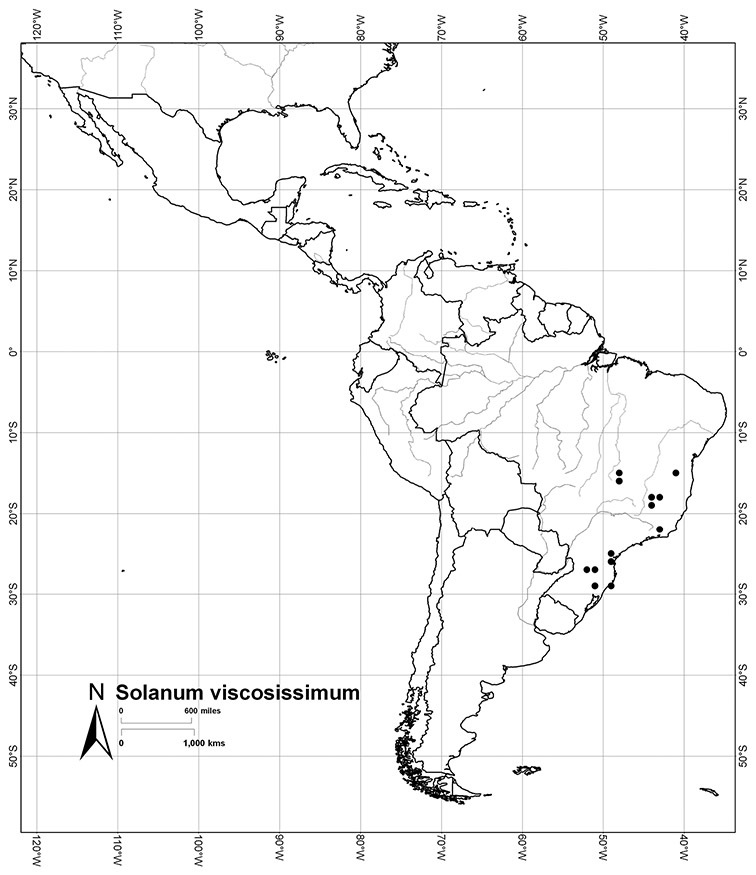
Distribution of Solanum*viscosissimum* Sendtn.

(Figure 107). Endemic to Brazil, from the Federal District south to the state of Rio Grande do Sul, from 500–1200 m.

##### Ecology.

Growing in forests and along forest margins, in the northern part of the range also found in cerrados (Distrito Federal).

##### Common names:

Brazil. Santa Catarina: joá cipó melado, juá (Smith and Downs 1966).

##### Conservation status.

Least Concern (LC); EOO >100,000 km^2^ (LC) and AOO >10,000 km^2^ (LC). See Moat (2007) for explanation of measurements.

##### Discussion.

*Solanum viscosissimum* is one of the most variable South American members of the Dulcamaroid clade in terms of leaf shape and pubescence. The many synonyms reflect this variation, and three of these were simultaneously published. Leaf shape varies from simple to almost completely pinnate both between plants and within plants. Leaves on the main growing stems tend to be more pinnatifid, while those of axillary, inflorescence-bearing shoots are usually simple and often have very short petioles so they clasp the stem. Collections with only the tips of shoots (such as *Sellow* s.n., a syntype of *Solanum amplexicaule*) can look radically different from more complete collections made of stems of varying ages. In addition to extreme variation in leaf shape and dissection, plants of *Solanum viscosissimum* vary in the degree of pubescence, with plants from the Rio de Janeiro area being almost glabrous and those from the more southerly part of the range (including the type of *Solanum viscosissimum*) being more densely pubescent with glandular trichomes. The trichomes of all plants, however, are of the same type – long (to 2 mm), simple and uniseriate, often with a 1-celled glandular tip. *Solanum viscosissimum* is unusual in *Solanum* in having the upper surfaces of the leaves consistently more pubescent than the lower surfaces. In southern Brazil *Solanum viscosissimum* is apparently shrubby (L.A. Mentz, pers. comm.), while more northerly populations are uniformly vining. Further molecular study at the population level may reveal differences in these two extremes.

*Solanum viscosissimum* could potentially be confused with *Solanum flaccidum* and *Solanum laxum*, with both of which it grows sympatrically. It differs from *Solanum flaccidum* in usually having at least some pinnate leaves, its smaller flowers with equal filaments, and in its pubescence of long glandular rather than shorter, non-glandular trichomes that are denser on the upper than lower leaf surfaces. *Solanum viscosissimum* is similar to *Solanum laxum* in its small, chartaceous leaves that are usually widest in the lower third, but those of *Solanum laxum* have short simple trichomes confined to the vein axils or the trichomes are entirely absent, while the trichomes of *Solanum viscosissimum* are evenly distributed over the leaf surfaces. Calyx lobes in *Solanum viscosissimum* are long triangular, while those of *Solanum laxum* are more deltate with a pronounced apical projection.

The names *Solanum viscosissimum*, *Solanum jasminifolium* and *Solanum amplexicaule* were published one after the other in Sendtner’s treatment of *Solanum* for the *Flora Brasilensis* (Sendtner 1846), so no one name has particular priority over the other. I have chosen to use *Solanum viscosissimum* for this taxon rather than any of the others as it was used in the flora of Santa Catarina state (Smith and Downs 1966) and has been recently used in a treatment of *Solanum* from the southern region of Brazil (Mentz and Oliveira 2004). If what I am treating as geographical differences in a widespread *Solanum viscosissimum* are later judged to be important at the specific level, the more northern material should be referred to as *Solanum jasminifolium*.

Many of the epithets associated with *Solanum viscosissimum* are represented by several syntype sheets in the herbaria cited in the original protologues, or are of uncertain provenance. No material traceable to Martius has been found for *Solanum viscosissimum* itself (fide Mentz and Oliveira 2004 and personal searches) so the name has been neotypified here with material that matches the protologue and is from a southern Brazilian herbarium (HBR) with duplicates that are widely distributed.*Solanum amplexicaule* was described using two collections, one from “Brasilia australis” of Sellow and the other from Itambé collected by J. Pohl. I have lectotypified this with the more complete of two sheets of *Pohl 3627* from Itambé held in W [W-0001951], both of which are annotated as “*Solanum amplexicaule* Sendt.”in Sendtner’s hand.

I have found only one duplicate of *Martius 821*, the collection used to describe *Solanum jasminifolium*, and have here selected it as a lectotype (M0166829), as there is no evidence that only one specimen was examined. It has a label in Sendtner’s hand, but no locality information.

Apparent duplicates of *Glaziou 18409* (the type collection of *Solanum amplexicaule* var. *pubescens*) at K (K000196343) and F (F-997974) have different collecting dates to the sheets in P, and are therefore not considered type material here.

##### Specimens examined.

**Brazil**. **Bahia**: sin. loc, 1847, *Blanchet* s.n. (G-DC); Barra do Choça, Barra do Choça, 3-6 km a E, estrada que liga Barra do Choça a Faz. Roda d’Agua (Rio Catolé), 22 Nov 1978, *Mori et al. 11311* (US); **Distrito Federal**: Reserva Ecológica do IBGE. Riacho Roncador, acima da chácara 2, 1100 m, 26 Oct 1995, *Aparecida da Silva 2817* (BM); Fazenda Vargem Bonita, Brasilia, 10 Jun 1963, *Heringer 9139* (US); Fazenda Vargem Bonita, ca. 10 km south of Brasilia, 19 Jul 1966, *Hunt & Ferreira Ramos 6684* (US); Planalto do Brasil, ca. 35 km E of Brasília, 700 m, 21 Aug 1964, *Irwin & Soderstrom 5392* (K, WIS); Planalto do Brasil, Chapada da Contagem, ca. 20 km E of Brasilia, 700 m, 14 Sep 1964, *Irwin & Soderstrom 6221* (K, WIS); Chapada da Contagem, ca. 10 km E of Brasilia (Planalto do Brasil), 1000 m, 17 Dec 1965, *Irwin et al. 11355* (K, MEXU, WIS); Riacho Vicente Pires, ca. 15 km W of Brasilia, 1000 m, 12 Jul 1966, *Irwin et al. 18165* (MO); Planalto do Brasil, riacho Vicente Pires, ca. 15 km W of Brasília, 1000 m, 12 Jul 1966, *Irwin et al. 18166* (K); Planalto do Brasil, Planaltina, 1000 m, 20 Jul 1966, *Irwin et al. 18293* (K); Planaltina, Planalto do Brasil, 20 Jul 1966, *Irwin et al. 18293* (F, MO); Córrego Jatobá, 1240 m, 25 Apr 1983, *Kirkbride Jr. 5243* (US); Fazenda Agua Limpia (University of Brasilia field station), near Vargen Bonita, c. 18 km SSW of Brasilia TV tower, on Corrego Capitipaga, 7 Aug 1976, *Ratter 3430* (F, K); Fazenda Agua Limpia (University of Brasilia field station), near Vargen Bonita, c. 18 km SSW of Brasilia TV tower, cabeceira do Corrego Capitinga, 27 Oct 1976, *Ratter 3867* (K); **Minas Gerais**: Inficionado, Nov 1834, *Lund* s.n. (G-DC); Serra da Ouro Preto, para Belo Horizonte, 27 Nov 1964, *Duarte 8621* (F); Gandarela, 22 Feb 1884, *Glaziou 15304* (P); Grão Mogol, Campo de Aviação, 1050 m, 21 Mar 1980, *Hatschbach 42853* (F); Serra do Espinhaço, ca. 17 km E of Diamantina, road to Mendanha, 1250 m, 29 Jan 1969, *Irwin et al. 22864* (UC, US); Joaquim Felício, 8 km W of Joaquim Felicio, Serra do Cabral, 1200 m, 7 Mar 1970, *Irwin et al. 27093* (MEXU, MO, WIS); Ouro Preto, Stacoloni, 28 Dec 1950, *Macedo 2767* (US); Serra do Cipó, k 132, 2 Sep 1933, *Mello Barreto 7627* (ECON); Santa Luzia, Serra do Cipó, km 132, 2 Sep 1933, *Mello Barreto 7825* (F); Diamantina, Serra dos Cristais, 6 Nov 1937, *Mello Barreto 9549* (F); Serra de Carassa, 1816, *Saint-Hilaire* s.n. (P); Serro, Serra de Monjolos, 18 km north of Sêrro, 5 May 1945, *Williams & Assis 6810* (US); **Paraná**: Calmon, 1000 m, 17 Mar 1910, *Dusén 9379* (GH, K); Calmon, 1000 m, 17 Mar 1910, *Dusén 9379* (L); Campina Grande do Sul, Taquari de Baixo, 18 Oct 1959, *Hatschbach 6348* (HBR, L, MBM, US); **Rio Grande do Sul**: Vila Oliva, pr. Caxias, 7 Feb 1946, *Rambo 31238* (B); Passo do Socorro, prope Vacaria, 27 Dec 1951, *Rambo 51595* (B); Passo da Guarda, prope Bom Jesus, 15 Jan 1952, *Rambo 51897* (US); Passo do Socorro, Vacaria, 800 m, 28 Jan 1951, *Sehnem 5757* (B); **Rio de Janeiro**: environs de Rio de Janeiro et D’Ouro Preto, 1883, *Glaziou 15304* (K); Rio de Janeiro, 1891, *Glaziou 18409* (K); Nova Friburgo, 1 Sep 1890, *Glaziou 18409* (F); **Santa Catarina**: Pinhal da Compania, Lauro Müller-Urussanga, 300 m, 23 Aug 1958, *Reitz & Klein 7040* (HBR, L, UC); Pinhal da Compania, Lauro Müller-Urussanga, 300 m, 21 Feb 1959, *Reitz & Klein 8510* (HBR, L, US); ad Sao Bento, 25 Jan 1890, *Schwacke 6943* (P); São Bento, 18 Jun 1885, *Schwacke 25695* (US); Campo Alegre, Campo Alegre, 4 km south on road to Jaraguá do Sul, 900 m, 6 Nov 1956, *Smith & Klein 7341* (HBR, US); **São Paulo**: Campos de Bocaina, Estação Ecológica de Aracuri, Esmeralda, 1600 m, 25 Nov 1950, *Brade 20553* (F); sin. loc, *Puggiari* s.n. (P); Apiahy, sites de Juan Barbosas, en las Arcias, camino del sitio de Lorenzo de Rosa, 8 Jul 1885, *Puggiari 3141* (P).

#### 45. Solanum wallacei

(A.Gray) Parish, Proc. Calif. Acad. Sci. ser. 3, 2: 166. 1901

http://species-id.net/wiki/Solanum_wallacei

Figure 108

Solanum xanti A.Gray var. *wallacei* A.Gray, Proc. Amer. Acad. Arts 11: 91. 1876. Type: United States of America. California: Los Angeles County, Santa Catalina Island, *W.A. Wallace* s.n. (holotype: GH [GH00077433]).

##### Type.

Based on *Solanum xanti* A.Gray var. *wallacei* A.Gray

##### Description.

Shrubs or small trees 1–3(-3) m tall, often spreading to 2 m diameter (“weakly erect” fide *Thorne & Everett 34931*). Stems densely pubescent with transparent simple uniseriate trichomes of varying lengths, the longest ca. 3 mm long, with weak tangled tips, usually glandular with a single-celled gland, drying brownish tan (“tawny”); new growth densely pubescent with similar simple uniseriate glandular trichomes, these glistening and glandular. Bark of older stems brownish green, somewhat glabrescent. Sympodial units plurifoliate. Leaves simple or very occasionally with two small lobes at the base, highly variable in size along a single stem, 3–11(-14) cm long, 1.6–5.5(-9) cm wide, elliptic to obovate, both surfaces densely and evenly pubescent with long simple uniseriate trichomes to 3 mm long, these usually glandular with single-celled glands on tips, the pubescence slightly denser along the veins; primary veins 7–9 pairs, drying paler than the lamina; base truncate or acute (seemingly depending on age of leaf); margins entire or slightly undulate, occasionally lobed with 1 or 2 small lobes near the base, the lobes very small; apex acute; petioles 1–2.5(-4) cm long, pubescent with simple uniseriate trichomes like the leaves and stems, never twining. Inflorescences terminal or lateral, (2-)4–10 cm long, usually branched once, occasionally more often, with 20–30 flowers, only a few open at a time, densely pubescent with transparent simple uniseriate glandular trichomes to 2.5 mm long like those of the stems; peduncle (1-)2–5 cm, flowers only borne above the lowest branching point; pedicels 1.5–2 cm long, ca. 0.5 mm in diameter at the base, ca. 1 mm in diameter at the apex, slender, spreading, densely pubescent with transparent simple trichomes like those of the inflorescence axis, articulated at the base and inserted into a small sleeve 1–1.5 mm long, this obscured by the dense pubescence. Buds ellipsoid to obovoid, included in the calyx tube until just before anthesis. Flowers all perfect. Calyx tube 3–3.5 mm long, broadly cup-shaped, the lobes 3–3.5 mm long, deltate, densely pubescent like the pedicels and inflorescence axis. Corolla 3–4.5 cm in diameter, violet to purple with or without green spots at the base of the lobes, the spots usually small and not confluent, rotate, lobed <1/4 of the way to the base, the lobes 0.3–0.5 mm long, 1–1.5 mm wide, planar or slightly cupped at anthesis, apiculate, the apiculae ca. 2 mm long, the abaxial surface densely pubescent with tangled simple uniseriate eglandular trichomes along the lobe midvein and laterally, the sinuses and adaxial surfaces glabrous. Filament tube minute, the free portion of the filaments 2–3 mm, pubescent with weak simple uniseriate trichomes near the base; anthers 4.5–5 mm long, 2–2.5 mm wide, ellipsoid, loosely connivent or spreading and not touching, poricidal at the tips, the pores lengthening to slits with age. Ovary glabrous; style 10–13 mm long, minutely papillate with slender glandular papillae or trichomes in the basal half; stigma capitate, the surface minutely papillate. Fruit a globose berry, 3–4 cm in diameter, glabrous, the pericarp shiny, green, turning yellow then black when ripe; fruiting pedicels 1.5–2 cm long, ca. 1.5 mm in diameter at the base, hanging from the weight of the berry. Seeds more than 100 per berry, 1.5–2 mm long, 1–1.5 mm wide, plumpish flattened reniform, reddish brown, the surfaces minutely pitted, the cells sinuate, when mature the seed appearing silky hairy with hair-like projections of the lateral cell walls, these ca. 0.2 mm long over the entire seed surface. Chromosome number: not known.

**Figure 108.**
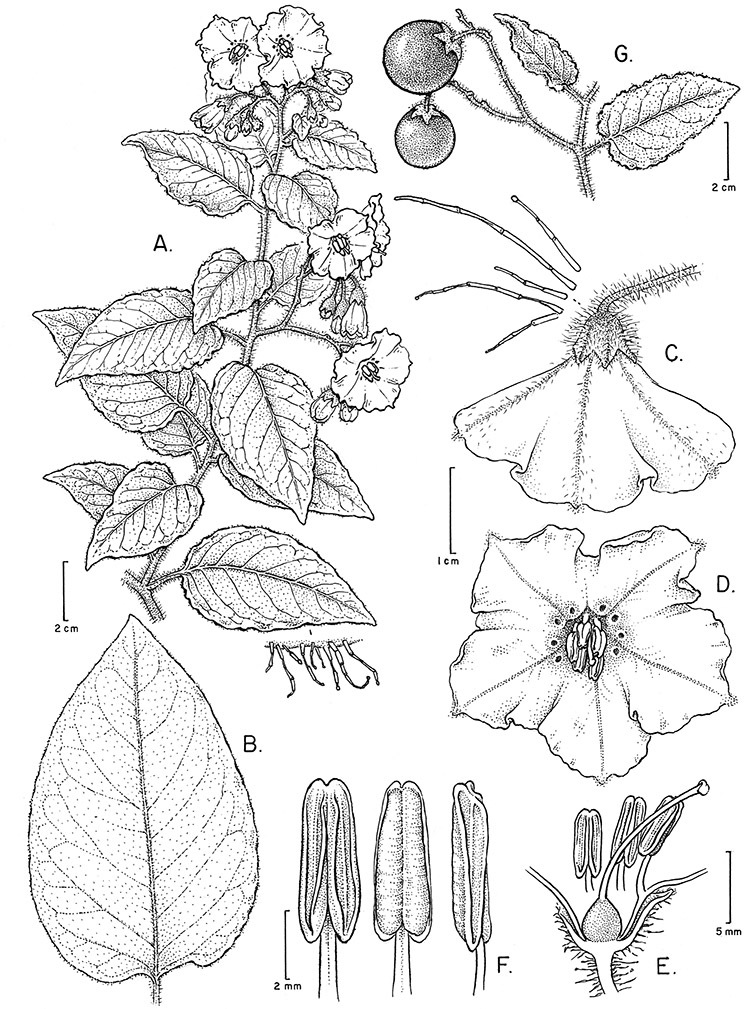
*Solanum wallacei* (A. Gray) Parish. (**A, C–F** drawn from *Moran 687* **B** drawn from *Trask* s.n., coll. May 1896 **G** drawn from *Walker 1433*). Illustration by Bobbi Angell.

##### Distribution

(Figure 100, inset). Endemic to Santa Catalina Island (Los Angeles County) off the coast of California, from sea level to 300 m.

##### Ecology.

*Solanum wallacei* occurs in chaparral, open areas and in canyon bottoms. It was formerly more common, and is now considered threatened, partly due to herbivory from goats.

##### Common names.

USA. California: Greasy nightshade (*Grant & Wheeler* s.n.), Santa Catalina nightshade, Wallace’s nightshade (CalFlora, http://www.calflora.org/cgi-bin/species_query.cgi?where-calrecnum=7665 ).

##### Conservation status.

Critically Endangered (CR); EOO <100 km^2^ (CR) and AOO <500 km^2^ (EN). See Moat (2007) for explanation of measurements.*Solanum wallacei* is on list 1B.1 (seriously endangered in California) in the California Native Plant Society’s *Inventory of Rare and Endangered Plants* (http://www.rareplants.cnps.org/detail/1480.html ). The main threats to this species are feral herbivores such as goats. The confusion over the correct application of the name has in part led to it not being recognised as endangered at the Federal level in the USA or on the IUCN Red List (IUCN 2001).

##### Discussion.

*Solanum wallacei* is quantatively as well as qualitatively distinct from *Solanum umbelliferum*, its closest relative, although DNA sequence data do not unambiguously support a specific level difference (George 2011). Flowers and fruits are larger, and the green spots on the corolla are smaller and often completely lacking in *Solanum wallacei*. Island forms of *Solanum umbelliferum* from the more northerly Channel Islands (sometimes recognised as *Solanum clokeyi*) approach *Solanum wallacei* in pubescence morphology, with long simple uniseriate trichomes, but the flowers and fruit of those island populations fall within the range of the rest of the distribution of *Solanum umbelliferum*.

##### Specimens examined.

**United States of America**. **California**. **Los Angeles County**: Santa Catalina Island, on lower part of Middle Ranch Canyon, 7 May 1971, *Davidson 1516* (UC); Santa Catalina Island, Hay Press, 19 Feb 1928, *Dunkle 1709* (RSA); Santa Catalina Island, Silverado Canyon, 28 Mar 1936, *Dunkle 4713* (RSA); Santa Catalina Island, along Cape Canyon Road, on E side at cliff, where creek comes up to road, 17 Mar 1997, *Elvin & Crockett 377* (RSA); Santa Catalina Island, Hamilton Canyon, 10 m, 18 Mar 1931, *Fosberg S-4285* (F, MO, RSA, UC, UC, US); Santa Catalina Island, Cape Canyon, 200 m, 6 May 1931, *Fosberg S-4812* (POM); Santa Catalina Island, Avalon, entrance of canyon W of Golf Links, 9 Apr 1901, *Grant* s.n. (A); Santa Catalina Island, Echo Lake, 10 Apr 1921, *Knopf 77* (F); Santa Catalina Island, White’s Landing, on hillside by mill, 50 m, 8 Mar 1941, *Moran 687* (MO, RSA, US); Santa Catalina Island, road east of Avalon, 3 m, 2 Jul 1909, *Pendleton & Reed 1366* (POM, UC); Santa Catalina Island, Upper White Landing Road, 381 m, 3 Mar 1973, *Propst & Hoefs 14* (RSA); Santa Catalina Island, Pebbly Beach Road, 15 m, 2 Jul 1909, *Reed 2819* (POM); Santa Catalina Island, SW part of the island in Cape Canyon, just above bed of canyon near roadside, 256 m, 23 Apr 1993, *Ross & Takara 6937a* (RSA); Santa Catalina Island, Middle Ranch Canyon near Eagle Nest Rock, 152 m, 23 Jun 1965, *Thorne & Everett 34931* (BM, RSA); Santa Catalina Island, near mouth of Hamilton Canyon NW of Avalon, 23 m, 23 Jun 1965, *Thorne & Everett 34995* (RSA); Santa Catalina Island, north slope of Black Jack Mountain, 1/8 mile down road to White’s Landing, 412 m, 5 Apr 1966, *Thorne 35798* (RSA); Santa Catalina Island, in canyon leading to White’s Landing, E of Black Jack Mountain, 91 m, 2 Jun 1966, *Thorne 36520* (GH, RSA); Santa Catalina Island, 1 mile beyond Eagle Nest Lodge, Middle Ranch Canyon, 25 May 1965, *Thorne 41570* (RSA); Santa Catalina Island, Avalon, Mar 1901, *Trask* s.n. (E, K, MO, US); Santa Catalina Island, along the road to Renton Mine, about due west of Pebbly Beach, 304 m, 9 May 1932, *Wolf 3491* (RSA, US); Santa Catalina Island, Hamilton Canyon, below turn in road, above airport, 22 m, 4 Oct 1932, *Wolf 4219* (A, RSA, UC); Santa Catalina Island, about 1 mile NE of the Wrigley Mausoleum at head of Avalon Valley, 122 m, 25 Jun 1941, *Wolf 10891* (RSA).

### Doubtful and excluded names and names not validly published

Additional details of the probable identities of names not validly published can be found on Solanaceae Source (http://www.solanaceaesource.org ).

*Solanum* grad. ambig. *Kieseritzkiana* Pojark., Fl. URSS 22: 10. 1955.

Type species: *Solanum kieseritzkii* C.A. Mey. (=*Solanum dulcamara* L.)(not validly published, no Latin).

*Solanum* section *Jasminosolanum* Bitter, Repert. Spec. Nov. Regni Veg. 17: 330. 1920. nomen nudum.

*Solanum amethystinum* Poit. ex Hook.f., Bot. Mag. 115: tab. 7062. 1889. pro. syn. *Solanum pensile* Sendtn. (= *Solanum uncinellum* Lindl.)

*Solanum berteroanum* (J.Rémy) Phil. ex Kuntze, Revis. Gen. Pl. 3(2): 226. 1898. nomen nudum (= *Solanum crispum* Ruiz & Pav.)

*Solanum cynanchoides* Dunal, Prodr. [A.P. de Candolle] 13(1): 82. 1852. pro. syn. *Solanum jasminoides* Paxton (= *Solanum laxum* Spreng.)

*Solanum dulcamara* L. var. *albiflora* Lév., Bull. Soc. Bot. France 55: 205. 1908. nomen nudum (= *Solanum lyratum* Thunb.)

*Solanum dulcamara* L. var. *album* Sweet, Hort. Brit., ed. 2: 385. 1830. nomen nudum. (= *Solanum dulcamara* L.)

*Solanum dulcamara* L. var. *lagodensis* Pobed., herbarium name (= *Solanum dulcamara* L.)

*Solanum dulcamara* L. var. *lyratum* (Thunb.) Bonati, [ined?] I have no evidence of the publication of this combination that appears in various indices.

*Solanum dulcamara* L. var. *plenum* G.Don, Gen. Hist. 4: 409. 1838. = *Nigella sativa* L. (Ranunculaceae). (see discussion of *Solanum dulcamara*)

*Solanum dulcamara* L. var. *variegatum* Sweet, Hort. Brit., ed. 2: 385. 1830. nomen nudum. (= *Solanum dulcamara* L.)

*Solanum dulcamaroides* Rich. ex Dunal, Prodr. [A.P. de Candolle] 13(1): 87. 1852. pro. syn. *Solanum pyrifolium* Lam. (= *Solanum pyrifolium* Lam.)

*Solanum floribundum* Dunal, Prodr. [A.P. de Candolle] 13(1): 92. 1852. nomen nudum, pro. syn. *Solanum congestiflorum* Dunal (= *Solanum crispum* Ruiz & Pav.)

*Solanum kitagawae* Schonb.-Tem. var. *percandidum* Kuv., herbarium name (= *Solanum dulcamara* L.)

*Solanum laurifolium* Deless. ex Dunal, Prodr. [A.P. de Candolle] 13(1): 80. 1852. pro. syn. *Solanum dominguense* Dunal (= *Solanum pyrifolium* Lam.)

*Solanum laxum* Royle, Ill. Bot. Himal. Mts. 279. 1835. nomen nudum; no description (“a new species of *Solanum* (*Solanum laxum* nob.) of a loose spreading habit”, Kunawur [Kinnear]), identity impossible to ascertain, but most likely *Solanum dulcamara* L. or *Solanum pittosporifolium* Hemsl.

*Solanum luridum* Dunal, Prodr. [A.P. de Candolle] 13(1): 88. 1852. pro. syn. *Solanum flaccidum* Vell. (= *Solanum flaccidum* Vell.)

*Solanum lycotonum* Hort. Panorm. ex Dunal, Prodr. [A.P. de Candolle] 13(1): 103. 1852. pro. syn. *Solanum cervantesii* Lag. (= *Solanum pubigerum* Dunal)

*Solanum lyratum* Thunb. var. *glabratum* Maxim., [?ined.] I have no evidence of the publication this name that appears in various indices.

*Solanum microphyllum* Griseb. ex Lechl., Berberid. Amer. Austral. 58. 1857. nomen nudum. (= *Solanum macbridei* Hunz. & Lallana)

*Solanum microphyllum* Pav. ex Dunal, Prodr. [A.P. de Candolle] 13(1): 86. 1852. pro. syn. *Solanum californicum* Dunal (= *Solanum umbelliferum* Eschsch.).

*Solanum pentadactylon* G. Don in Loudon, Hort. Brit. 72. 1830. nomen nudum (?*Solanum seaforthianum* Andrews).

*Solanum prehensile* Pittier, Cat. Fl. Venez. 2: 380. 1947. nomen nudum (= *Solanum uncinellum* Lindl.)

*Solanum pyriforme* Poir., Encycl. (Lamarck) 4: 291. 1797. Clearly a mis-spelling of *Solanum pyrifolium* in indices, the name “pyriforme” does not appear anywhere on page 291, and clear reference made to *Solanum pyrifolium* Lam.

*Solanum sarmentosum* Pav. ex Dunal, Prodr. [A.P. de Candolle] 13(1): 87. 1852. pro. syn. *Solanum macrantherum* Dunal (= *Solanum dulcamaroides* Poir.)

*Solanum scandens* R.H.Schom. ex Dunal, Prodr. [A.P. de Candolle] 13(1): 84. 1852. pro. syn. *Solanum pensile* Sendtn. (= *Solanum uncinellum* Lindl.)

*Solanum scandens* Comm. ex Dunal, Prodr. [A.P. de Candolle] 13(1): 88. 1852. pro. syn. *Solanum pyrifolium* Lam. (= *Solanum pyrifolium* Lam.)

*Solanum scandens* Moc. & Sessé ex Dunal, Prodr. [A.P. de Candolle] 13(1): 87. 1852. pro. syn. *Solanum macrantherum* Dunal (= *Solanum dulcamaroides* Poir.)

*Solanum schneideri* F.Phil., Cat. Pl. Vasc. Chil. 229. 1881. nomen nudum (= *Solanum alphonsei* in list)

*Solanum serpentini* Borbas & Waisb. (1897) – no literature record found (see discussion of *Solanum dulcamara* L.)

*Solanum uvariifolium* Dunal, Prodr. [A.P. de Candolle] 13(1): 88. 1852. pro. syn. *Solanum flaccidum* Vell. (= *Solanum flaccidum* Vell.)

*Solanum xanti* Coville, [ined.?], I have no evidence of the publication this name that appears in various indices; it is likely to be a mere mention of *Solanum xanti* A.Gray in text.

*Witheringia microphylla* Griseb., in Lechler, Berberid. Amer. Austral. 58. 1857. nomen nudum (= *Solanum macbridei* Hunz & Lallana)

## Supplementary Material

XML Treatment for Solanum

XML Treatment for Solanum agnoston

XML Treatment for Solanum aligerum

XML Treatment for Solanum alphonsei

XML Treatment for Solanum amygdalifolium

XML Treatment for Solanum angustifidum

XML Treatment for Solanum aspersum

XML Treatment for Solanum aureum

XML Treatment for Solanum boldoense

XML Treatment for Solanum calileguae

XML Treatment for Solanum coalitum

XML Treatment for Solanum crispum

XML Treatment for Solanum cutervanum

XML Treatment for Solanum dichroandrum

XML Treatment for Solanum dulcamara

XML Treatment for Solanum dulcamaroides

XML Treatment for Solanum endoadenium

XML Treatment for Solanum flaccidum

XML Treatment for Solanum imbaburense

XML Treatment for Solanum inodorum

XML Treatment for Solanum kulliwaita

XML Treatment for Solanum laxum

XML Treatment for Solanum leiophyllum

XML Treatment for Solanum luculentum

XML Treatment for Solanum lyratum

XML Treatment for Solanum macbridei

XML Treatment for Solanum muenscheri

XML Treatment for Solanum nitidum

XML Treatment for Solanum odoriferum

XML Treatment for Solanum pittosporifolium

XML Treatment for Solanum pubigerum

XML Treatment for Solanum pyrifolium

XML Treatment for Solanum ruizii

XML Treatment for Solanum salicifolium

XML Treatment for Solanum sanchez-vegae

XML Treatment for Solanum seaforthianum

XML Treatment for Solanum septemlobum

XML Treatment for Solanum sousae

XML Treatment for Solanum stenophyllum

XML Treatment for Solanum storkii

XML Treatment for Solanum triquetrum

XML Treatment for Solanum umbelliferum

XML Treatment for Solanum uncinellum

XML Treatment for Solanum valdiviense

XML Treatment for Solanum viscosissimum

XML Treatment for Solanum wallacei

## Figures and Tables

**Table:** 

**Column heading**	**Content**
Family	Family name
Collated name	Scientific name with authority
Genus	Generic name
Species	Specific epithet
Authority	Author or author string
Status	Cultivation status (CUL = cultivated plants)
Collector	Principal collector
Prefix	Prefix to Collection number
Collection number	Collection number
Suffix	Suffix to Collection number
Additional collectors	Additional collectors
Herbaria	Herbaria in which duplicates were seen
Collection day	Collection day
Collection month	Collection month
Collection year	Collection year
Collated collection date	Day Month Year
Month name	Collection month as English abbreviation
Continent	Continent
Region	Region
Country	Country
Major area	Major political division
Minor area	Next minor political division
Gazetteer	Nearest town or place on a map
Specific locality	Specific locality
Coordinate resolution	Degree of accuracy of the geo-coordinates
Latitude (decimal)	Decimal degrees latitude
Longitude (decimal)	Decimal degrees longitude
Altitude	Elevation in metres
Altitude maximum	Maximum elevation in metres (if range given)
Altitude range	Range of elevation if given
Common name	Common name
Language of common name	Language in which the common name occurs
Plant description	Description of plant from specimen label
Habitat description	Description of habitat from specimen label
Collated geographical…	Summary of locality information in string
Type summary	Type status of collection

## Index to numbered collections examined

Numbers in parentheses refer to the corresponding species in the text and the numerical list of species above. Collections by anonymous collectors without date or other identifying features are not listed here (ca. 200 gatherings). Only first collectors in collections made by two or more collectors are listed here; pairs of collectors are listed in the Specimens examined section, while groups of three or more are listed as *et al*. Full collector strings can be found on Solanaceae Source (http://www.solanaceaesource.org ) or in the data supplement to this paper.

AAsamaa, H. 11616 (dulcamara)

ATBP 786 (seaforthianum)

Abbott, R.Q. 210, 358 (dulcamaroides)

Abrams, L.R. 76, 149, 154, 160, 336, 342, 380, 597, 1298, 2262, 2367, 2390, 2556, 2586, 3362, 3593, 4139, 4271, 4676, 5073, 5569, 5695, 5899, 6156, 6484, 6530, 6951, 7132, 7816, 8109, 10023, 10128, 10151, 10165, 10417, 11625, 12380, 12514, 13802 (umbelliferum)

Acevedo-Rodríguez, P. 49 (pubigerum); 1213 (aspersum); 4623 (aligerum); 7296, 9255, 10070 (uncinellum); 13175 (pyrifolium)

Ackerman, L. 13 (umbelliferum)

Ackermann, M. 297 (nitidum)

Acleto, C. 294 (cutervanum)

Acosta, L.E. 84, 84, 188 (seaforthianum)

Acosta-Solís, M. BB181 (crispum); 6867 (laxum); 7172 (nitidum); 7460 (uncinellum); 7575A (aureum); 7786 (cutervanum); 8301 (stenophyllum); 8994, 10571, 11148, 11204 (aureum); 13223 (stenophyllum); 14159, 14422 (aureum)

Adams, J.W. 5416 (dulcamara)

Adamson, R.S. 1208 (dulcamara)

Adolfo, Brother 326 (aligerum)

Adrien, F. 2098 (dulcamara)

Aedo, C. 2309, 2469, 2490, 3164 (dulcamara); 7072 (alphonsei); 7222 (valdiviense); 8428, 10491bis (dulcamara); 14489, 14606, 14644, 14734 (aligerum); 14739, 14783 (nitidum); 16670 (sanchez-vegae)

Aellen, P. 4601 (dulcamara)

Agnew, ? W1679 (dulcamara)

Aguilar, I. 669, 713 (pubigerum)

Aguilar, M. 1085 (uncinellum)

Aguilar, R. 2075 (seaforthianum)

Aguilar, R.M. 920 (angustifidum)

Aguirre, ? 532 (pubigerum)

Ahart, L. 3384, 5599, 5674, 8752, 9561, 9771, 12633, 12747 (umbelliferum)

Ahumada, O. 8178 (calileguae)

Aitchison, J.E.T. 861 (dulcamara)

Akey, W.W. 61 (umbelliferum)

Al-Rathi, M.S. 5437 (dulcamara)

al Rawi, A. 12159 (dulcamara)

Alain [Brother] 1481, 3525 (seaforthianum)

Albert de Escobar, L. 7939 (luculentum)

Albertus, C.G. 32 (umbelliferum)

Albán, J. 8078, 10081 (nitidum)

Alegaen, V. 206 (seaforthianum)

Alexander, A.M. 2297, 2980, 3585 (umbelliferum)

Alexeenko, T. 47, 11205, 11289, 11290, 11291, 11304, 14374 (dulcamara)

Alfaro, E. 1184 (storkii)

Allard, H.A. 4653 (dulcamara)

Allart, A. 501 (seaforthianum)

Allen, P.H. 604 (storkii); 7172 (aligerum)

Alm, C.G. 272 (dulcamara)

Almazán Rodriguez, ? 950 (pubigerum)

Almeda, F. 6553 (umbelliferum)

Almquist, E. 5300 (dulcamara)

Alston, A.H.G. 815 (dulcamara); 6015 (seaforthianum)

Altamirano, F. 472 (pubigerum)

Alvítez I, E. 1057 (sanchez-vegae)

Ambrosetti, J.A. 1477, 1485 (salicifolium)

Anderson, B. 2886, 2891 (umbelliferum)

Anderson, D.E. 3778 (umbelliferum)

Anderson, D.L. 1490, 1771, 3168, 3268, 3416, 3574, 3769, 3793 (salicifolium)

Anderson, E.F. 3253 (umbelliferum)

Anderson, J. 25 (valdiviense); 86 (laxum)

Anderson, T. 7930 (nitidum)

Anderson, W.R. 13070 (pubigerum)

Andersson, J.G. 56, 337a, 627a (septemlobum)

Andreas, C.H. 197 (valdiviense)

Andrews, L.M. 501 (pubigerum)

Andrieux, G. 184 (pubigerum); 185 (dulcamaroides); 204 (pubigerum); 395 (dulcamaroides)

André, E. 805, 2613 (uncinellum); 3619 (stenophyllum); 3922 (aureum)

Ángeles, I. de los 119 (seaforthianum)

Angier, B.S. 70 (umbelliferum)

Angulo, L. 195 (storkii)

Anna, M. NPGRC-396 (seaforthianum)

Anthony, A.W. 30, 210, 228 (umbelliferum)

Antonio C., ? 48 (stenophyllum)

Aparecida da Silva, M. 2817 (viscosissimum)

Applegate, E.I. 380, 381, 2137, 5311, 5451, 8854 (umbelliferum)

Arakaki, M. 69 (amygdalifolium)

Araque M, J. 19Ar353 (aligerum)

Archbold, M.E. 1871 (seaforthianum)

Archer, W.A. 373 (seaforthianum); 1153, 1227 (luculentum); 8020 (uncinellum)

Argüelles, E. 802, 1666, 1667 (dulcamaroides); 2560 (pubigerum)

Ariste-Joseph, Brother A418 (dichroandrum); A733 (seaforthianum)

Ariza Espinar, L. 2798 (salicifolium); 2857 (angustifidum)

Armitage, F.B. 154/59 (seaforthianum)

Armstrong, E. 931, 1076, 1152 (umbelliferum)

Arnett, M. 8088 (umbelliferum)

Arockiasamy, D.I. 10038 (seaforthianum)

Aronson, J. 541 (macbridei); 860 (uncinellum)

Arp, G.K. 4532 (aligerum)

Arriaga C, R. 389 (aligerum)

Arsène, G. [Brother] 9, 325, 325a, 1444, 1932 (pubigerum); 2330, 2669, 2894 (dulcamaroides); 3534, 3538, 3538, 5518 (pubigerum); 5970 (dulcamaroides); 6424 (triquetrum); 8492 (dulcamaroides); 8776 (pubigerum); 9669 (dulcamaroides); 10014 (laxum)

Asampaio, ? 374 (laxum); 2425 (flaccidum)

Ascencio V, M.A. 20 (pubigerum)

Ashfield, P. 8 (umbelliferum)

Asp, O. 46 (valdiviense)

Asplund, E. 16993 (stenophyllum)

Astegiano, M. 35 (angustifidum)

Atha, D.E. 5005 (dulcamara)

Atherton, G. 86 (seaforthianum)

Atwood, D. 17663 (umbelliferum)

Aubréville, A. 24 (storkii)

Aulestia, C. 1395 (uncinellum)

Aureoles, S. 8521 (pubigerum)

Austin, R.M. 43, 140, 158 (umbelliferum)

Austin, R.M. (Mrs) 1872 (umbelliferum)

Ávarez, A. 1407 (stenophyllum); 1480 (leiophyllum)

Ávarez, I. 1490 (dulcamara)

Avendaño Reyes, S. 51 (laxum)

Averyanov, L. CBL552 (pittosporifolium); CBL657 (lyratum)

Ávila, A. de 146, 178 (pubigerum); 664 (aligerum)

Axelrod, D. 178, 413, 617 (umbelliferum)

Babcock, E.B. 8, 9 (wallacei)

Babington, C.C. 299 (dulcamara)

Bacigalupi, R. 1648, 2408, 3175, 3292, 5200, 5247 (umbelliferum)

Bacle, C.H. 55 (amygdalifolium)

Bacon, J. 1734 (pubigerum)

Baenitz, C. 30303 (laxum)

Bailey, H. 2028 (umbelliferum)

Bailey, L.H. 202 (pyrifolium); 929 (laxum); 941 (crispum); 1312 (laxum); 1897 (seaforthianum)

Bailey, W.G. 15 (dulcamara)

Baker, C.F. 48, 234, 273, 282, 304, 2890, 4059 (umbelliferum)

Baker, K. 054 (aligerum)

Baker, M.S. 2859b, 3401b, 3489b, 4118, 4868a, 8992, 9271, 12126 (umbelliferum)

Baker, R.E.D. 14848 (seaforthianum)

Balansa, B. 1137 (dulcamara); 2012 (amygdalifolium); 2106, 2106 (laxum)

Baliguo, B. 944 (amygdalifolium)

Ball, C.R. 916 (triquetrum)

Ballantyne, M. 46, 263 (umbelliferum)

Balls, E.K. B5826 (aureum); 7644 (dulcamara); 7871a (umbelliferum); 8184 (wallacei); 8725, 8772, 9123, 10930, 11216, 13930, 14458, 14535, 17085, 17137 (umbelliferum); 19052 (wallacei); 22522, 22872 (umbelliferum)

Bally, P.R.O. 6565, B6565, 13282 (seaforthianum)

Balslev, H. 10579 (uncinellum)

Bang, M. 90 (nitidum); 522, 532, 1662 (uncinellum); 2872 (aligerum)

Bangerter, E.B. 802, 1058 (dulcamara)

Banks, D.L. 75, 180, 499, 1401, 1562, 1829 (umbelliferum)

Barabas, N. 487 (dulcamara)

Barbe, D. 2 (umbelliferum)

Barber, J.H. 68, 1002 (umbelliferum)

Barbosa, E. 355 (inodorum)

Barboza, G.E. 140, 152, 221, 230, 336, 553, 561 (salicifolium); 578 (endoadenium); 588, 877 (salicifolium); 1070 (aligerum); 1566 (amygdalifolium); 1929, 1938 (salicifolium); 1997 (endoadenium); 2709 (salicifolium); 2760, 2786 (endoadenium); 2811, 2845, 2960 (salicifolium); 3018 (endoadenium); 3224, 3225, 3245, 3269 (salicifolium); 3258 (endoadenium), 3467, 3473 (salicifolium); 3476 (endoadenium); 3478, 3485, 3488, (salacifolium); 3489 (angustifidum); 3490 (endoadenium); 3494 (salicifolium); 3498 (aligerum); 3549 (aligerum); 3772, 3774 (valdiviense)

Barchet, S.P. 509 (lyratum)

Barclay, A.S. 3404 (luculentum)

Barclay, G.W. 194 (crispum); 1606 (umbelliferum)

Barclay, H.G. 8386 (stenophyllum)

Barkley, F.A. 189 (salicifolium); 264 (luculentum); 7443 (pubigerum)

Barrera, J.C. H.Luti-8244 (angustifidum)

Barringer, K. 2233, 2922 (aligerum); 3005 (pubigerum); 3109 (storkii)

Barrington, D.S. 581 (storkii)

Barry, M.A. 114 (umbelliferum)

Bartholomew, B. 1278 (pittosporifolium); 1576, 1651 (lyratum); 2185 (umbelliferum); 2879, 3162 (pubigerum); 4260 (umbelliferum); 7613 (lyratum)

Bartholomew, D. 86 (umbelliferum)

Bartlett, H.H. 19404, 19427, 20171, 20623, 20944 (salicifolium)

Bartram, E.B. 24435 (dulcamara)

Bass, M.S. 179, 179 (uncinellum)

Bastardo, R.H. 1245 (seaforthianum)

Bastelleros, L. 421 (aligerum)

Basualto, S. 37, 156 (crispum)

Bates, D. 1702 (seaforthianum)

Bates, V. 83 (dulcamara)

Bauer, H.L. 15383 (umbelliferum)

Baxter, P. 26, 81 (crispum)

Bayliss, R.D.A. 3447 (seaforthianum)

Beach, K.H. 27 (septemlobum); 658 (umbelliferum)

Beaman, J.H. 5316, 6436 (dulcamaroides)

Beane, L. 1495 (umbelliferum)

Bechtel, A.R. 7126 (dulcamara)

Beck, S.G. 3904 (aligerum); 5988 (uncinellum); 7054, 8931, 21713 (aligerum)

Becker, ? 396 (dulcamara)

Becker, A. 63, 263, 336, 344 (dulcamara)

Beliz, M. 5046 (dulcamara)

Bell, C.R. 17480 (umbelliferum)

Bell, S. 393 (umbelliferum)

Bello, E. 5347 (uncinellum)

Bellonio, J.P. 253 (aligerum)

Belshaw, C.M. 19, 57, 120, 197, 1002, 1744, 1876 (umbelliferum)

Beltrán, H. 388 (nitidum)

Benjamine, E. 67 (umbelliferum)

Benke, H.C. 4878, 5661, 6469 (dulcamara)

Bennett, A. 415, 639 (dulcamara)

Bennett, A.W. 873b (dulcamara)

Benny, G.L. 285 (umbelliferum)

Benoist, R. 2211, 3630, 3908 (aureum)

Benson, L. 22, 81, 83, 463, 577, 1777, 1783, 2658, 3573, 4358, 6470, 7598, 7996, 8439, 11714, 11759, 11921, 12426, 14313 (umbelliferum)

Bento, D. 1192 (laxum)

Benítez de Rojas, C.E. 366, 708 (seaforthianum)

Bergman, S. 440 (dulcamara)

Berkheimer, D. 2565 (dulcamara)

Berlandier, J.L. 154 (triquetrum); 353, 792en1 (pubigerum); 1414, 1416, 1479, 2299 (triquetrum)

Berlin, B. 648 (uncinellum)

Bernadello, G. 790 (angustifidum)

Bernardi, L. 10898 (dichroandrum); 15239, 16014, 19874 (seaforthianum)

Bernoulli, K.G. 2343 (seaforthianum)

Bernstein, N. 6 (dulcamara)

Berro, M.B. 4797 (laxum)

Bertero, C.L.G. 634 (crispum); 635, 639 (alphonsei); 640, 1323, 1327, 1329 (crispum)

Best, C. 737 (umbelliferum)

Betancur, J. 5912 (luculentum)

Bickham, S.H. 1134 (dulcamara)

Bigger, M. 1100 (seaforthianum)

Billiet, F. 3063 (amygdalifolium); 6067 (aligerum); 6972 (valdiviense)

Billot, E. 160 (dulcamara)

Biltmore Herbarium 14805 (triquetrum)

Bird, R.M. 1389 (cutervanum)

Bird, W. 43, 1475 (dulcamara)

Bissell, C.H. 22405 (dulcamara)

Bisset, J. 702 (lyratum)

Bissing, D.R. 24, 98 (umbelliferum)

Biswell, H.H. 9 (umbelliferum)

Biurrun, F. 959, 2672, 5045, 5121 (salicifolium)

Blackmore, S. 151 (seaforthianum)

Blackwelder, R.E. 168 (umbelliferum)

Blakley, E.R. 3532, 3863, 4486, 7300, 7608 (umbelliferum)

Blanchet, J.S. 1752 (uncinellum); 3828 (viscosissimum)

Bobrov, A.E. 298 (dulcamara)

Bobrov, E. 168 (dulcamara)

Bodin, J.E. 12 (triquetrum)

Boege, W. 1738 (aligerum)

Boeke, J.D. 595 (aureum); 1927 (sanchez-vegae); 2148 (kulliwaita); 2589 (aureum); 3049 (aligerum)

Boelcke, O. 4843, 4910, 6358 (laxum); 9873 (salicifolium); 10786 (crispum)

Boffa, P. 132 (amygdalifolium)

Bogdanoff, M. 26 (umbelliferum)

Bohs, L. 2145, 2145 (nitidum); 3132 (aureum); 3320 (coalitum); 3886 (seaforthianum)

Bokhari, M.H. 2159 (dulcamara)

Bolt, B. 532, 664 (umbelliferum)

Bonilla B, R. 162 (pubigerum)

Bonilla, J. 654 (pubigerum)

Bono, J. 4066 (dichroandrum)

Borell, A.E. 4 (umbelliferum)

Borhidi, A. 85359 (seaforthianum)

Borieova, A. 472 (dulcamara)

Borisova, A.G. 365, 369 (dulcamara)

Bornmüller, A. 135 (laxum)

Bornmüller, J. 344, 1405, 3073, 13470 (dulcamara)

Bosré, ? 226 (dulcamara)

Botteri, M. 229 (dulcamaroides); 1189 (aligerum)

Bottfreund, ? 226 (endoadenium)

Boufford, D.E. 23332, 24748 (pittosporifolium); 35933 (lyratum); 36963 (pittosporifolium); 37306 (lyratum)

Bourgeau, E. 116, 716, 916, 1256 (pubigerum); 3054 (dulcamaroides)

Boutin, F. 2317 (pubigerum)

Bowden, L.F. 137 (dulcamara)

Bowerman, M.L. 258 (umbelliferum); 553 (umbelliferum)

Bowman, A.M. 253 (umbelliferum)

Boyd, S. 2036, 2553, 2991, 3956, 5510, 6116, 6658, 6741, 7965, 8407, 9422, 9776, 9813, 10342, 10537, 11174, 11478 (umbelliferum)

Boyle, B. 2512, 2513 (aligerum)

Bracelin, N.F. 2583, 2584 (umbelliferum)

Brade, A.C. 6031 (flaccidum); 14654 (inodorum); 18054 (flaccidum); 20553 (viscosissimum)

Braden, G. 9 (umbelliferum)

Bradshaw, K. 39 (umbelliferum)

Braem, S. 23, 784, 785 (umbelliferum)

Brainerd, E. 146 (umbelliferum)

Brandbyge, J. 42041 (stenophyllum); 42071 (nitidum); 42201, 42819 (stenophyllum)

Brandegee, T.S. 1667, 3367 (umbelliferum)

Braun, ? 1122 (seaforthianum)

Braunton, E. 464, 465, 619, 796 (umbelliferum)

Bravo H, H. 256-7407 (umbelliferum)

Bray, W.L. 41 (triquetrum)

Breckon, G.J. 1364 (aligerum)

Breedlove, D.E. 1821, 2056, 2066-A, 2702, 2786, 3554, 3642, 4089, 5470 (umbelliferum); 6053 (pubigerum); 6700, 6879, 8542, 8744 (aligerum); 9471 (pubigerum); 9540, 10435 (aligerum); 11612 (pubigerum); 13575 (dulcamaroides); 14523 (aligerum); 15376, 22976, 25721 (pubigerum); 29167, 31051 (dulcamaroides); 36185 (pubigerum); 38220 (dulcamaroides); 40432 (pubigerum); 40863 (aligerum); 43614 (umbelliferum); 45756 (dulcamaroides); 47054 (pubigerum); 47819 (aligerum)

Brenan, J.P.M. 46297 (dulcamara)

Bretschneider, E. 1927 (septemlobum)

Brewer, W.H. 58, 108, 189, 207, 633 (umbelliferum)

Bridges, T. 184 (umbelliferum); 397 (crispum)

Bright, J.F. 8095 (umbelliferum)

Bringas C, F. 83 (pubigerum)

Brinton-Lee, D. 1346 (dulcamara)

Britton, C.E. 2752 (dulcamara)

Britton, N.L. 2820 (uncinellum); 6674 (boldoense); 9867 (seaforthianum)

Brizuela, J. 884 (amygdalifolium)

Broadway, W.E. 492, 833, 4693 (seaforthianum); 6369 (seaforthianum)

Broder, R.E. 581, 732, 763 (umbelliferum)

Brooke, W.M.A. 5039, 5177 (nitidum); 6355 (macbridei); 6383 (nitidum); 6711 (aligerum); 6951 (crispum); 6971 (valdiviense)

Brotherus, A.H. 765 (dulcamara)

Brown, ? 1638 (aligerum)

Brown, D. 23 (umbelliferum)

Brown, H.E. 191 (umbelliferum)

Brown, H.S. 1056 (umbelliferum)

Brown, W.W. 63 (umbelliferum)

Brownless, P. DCI-937 (valdiviense)

Bruce, C.C. 1872 (umbelliferum)

Bruff, J.R. 1087 (pubigerum)

Brummemeyer, ? 10103 (pittosporifolium)

Brummitt, R.K. 298 (dulcamara)

Brunner, D.R. 911, 925 (laxum); 1218 (amygdalifolium)

Bryan, M.L. 94 (umbelliferum)

Buchtien, O. 21 (valdiviense); 115, 621 (nitidum)

Buck, R.E. 1117, 1238 (umbelliferum)

Bueno, O.L. 355, 1026 (laxum)

Bukinich, D. 45 (dulcamara); 535 (septemlobum)

Bullard, W.E. 40 (umbelliferum)

Bunting, G.S. 11152 (uncinellum)

Burch, D. 2538 (pyrifolium); 4713 (aligerum)

Burchell, W.J. 2269 (uncinellum); 4599 (inodorum)

Bürger, A.O. 48 (crispum)

Burger, W.A. 5972, 7941 (storkii)

Burger, W.C. 6482 (aligerum); 7903 (aligerum); 11521 (aligerum); 11525 (storkii)

Burgess, J. 119, 182, 184 (umbelliferum)

Burgess, T.J.W. 1609, 1609 (dulcamara)

Burkart, A. 4134 (laxum); 7490 (salicifolium); `8330 (laxum); 13224 (aligerum); 22718 (laxum); 23476 (angustifidum); 24920, 25452, 27072 (laxum); 30139, 30151 (angustifidum); 30153 (laxum); 30319 (angustifidum)

Burkill, J.H. 184 (wallacei)

Burns, J.S. 20 (umbelliferum)

Búrquez, A. 96-205 (seaforthianum)

Burtt Davy, J. 254, 985, 1358, 2343, 4132 (umbelliferum)

Bush, B.F. 1621 (triquetrum)

Bush, E. 57, 58 (dulcamara)

Bustillo, C. 21 (seaforthianum)

Butler, G.D. 16, 18 (umbelliferum)

Butler, J. 406 (dulcamara)

Butzke, A. 7743, 10737 (laxum)

Buzato, S. 20633 (inodorum)

Buzbee, M.L. 52 (umbelliferum)

Bye, R.A. 11953 (pubigerum)

Bywater, W.M. 54 (dulcamara)

Cabellero, J. 40 (dulcamaroides)

Cabrera, A.L. 1812, 3845 (laxum); 3966 (amygdalifolium); 16142 (aligerum); 18803 (salicifolium); 21083, 23933, 23943, 24214 (aligerum); 25639 (calilaguae); 28674 (laxum); 29778, 32061 (aligerum)

Cabrera, E. 7255 (dulcamaroides); 14670 (seaforthianum)

Cáceres, F. 813A, 2857 (nitidum)

Cahill, N. 395 (umbelliferum)

Callejas, R. 8339 (luculentum)

Calzada, J.I. 642 (dulcamaroides); 1514 (dulcamaroides); 2063 (seaforthianum); 4178 (aligerum); 4181 (aligerum); 4223 (dulcamaroides); 5010, 5464 (aligerum); 12992, 14291, 14997 (dulcamaroides); 18460, 19590, 20702 (aligerum); 21361 (pubigerum); 24284 (seaforthianum)

Camargo, O. 649, 2061 (laxum)

Camp, W.H. E-3940 (aureum); E-4698 (cutervanum)

Campbell, G.R. 581, 14563 (umbelliferum)

Campbell, G.W. 12821 (wallacei)

Campbell, M.L. 39 (umbelliferum)

Campbell, R.W. 3 (umbelliferum)

Campos Novaes, J. 167, 4089 (flaccidum)

Campos V, A. 4567 (pubigerum); 886 (aligerum); 2457 (dulcamaroides)

Campos, J. 5102, 5183 (cutervanum)

Cannon, J.F.M. 2743 (dulcamara)

Cano, A. 4180, 4225 (macbridei); 7439 (cutervanum); 8062, 8513, 8552b (nitidum); 16766 (cutervanum)

Cantelow, E.D. 1440, 1441, 2071, 4486, 4490 (umbelliferum)

Cantelow, H.C. 468 (umbelliferum)

Cantino, P. 447 (salicifolium)

Capurro, ? 62 (aligerum)

Carballo, R.A. 281 (aligerum)

Cárdenas, M. 188 (angustifidum); 1152, 3035 (aligerum); 3731 (angustifidum); 4136, 4675 (aligerum); 5447A (salicifolium); 5772 (aligerum)

Cardoso, V.L. 1203 (pubigerum)

Carles, W.R. 139, 403 (lyratum)

Carlson, M. 3563 (storkii)

Carlson, M.C. 1466, 3693 (pubigerum)

Carnevali, R. 1746 (angustifidum)

Carpenter, J.B. 503 (aligerum); 512 (storkii)

Carranza, E. 1251 (aligerum); 1409 (seaforthianum); 2264 (pubigerum); 2689 (seaforthianum); 5824 (dulcamaroides)

Carrilo-Reyes, P. 2479 (dulcamaroides)

Carter, A. 1056, 1057, 1250, 2557 (umbelliferum)

Carter, A.M. 1073 (umbelliferum)

Carter, E. 234 (umbelliferum)

Carter, J.E. 375, 952 (dulcamara)

Carter, S. 2 (nitidum)

Carter, W.E. 158 (nitidum)

Cascante, A. 206 (storkii); 757 (aligerum)

Cassen, P.M. 38 (seaforthianum)

Castellanos, A. 23 (angustifidum); 74 (laxum); 822 (amygdalifolium); 3223 (salicifolium); 3353 (angustifidum); 17776 (amygdalifolium)

Castilla, F. 536 (dulcamara)

Castillo C., G. 1243 (aligerum); 1943 (pubigerum)

Castillón, L. 1033 (salicifolium); 1613 (endoadenium)

Castroviejo, S. 9648 (laxum); 15128SC (storkii)

Caumel, J.B. 334 (dulcamara)

Cavalerie, P.J. 3147 (pittosporifolium)

Cayouette, J. 74-440 (dulcamara)

Cazalet, P.C.D. 5093, 7793 (uncinellum)

CDC-CECON 1535 (aligerum)

Ce-Ming, T. 95683 (lyratum)

Cedillo Trigos, R. 1031, 3780 (dulcamaroides)

Censo, ? 12 (salicifolium)

Cerana, M.M. 1259 (salicifolium)

Cerón, C.E. 11842 (aureum); 15542 (cutervanum); 19514 (nitidum)

Cerrate, E. 1902, 4519, 4846, 5335 (nitidum)

Céssac, ? de 32, 128, 132, 132b (umbelliferum)

Chandler, E.R. 939, 3299 (umbelliferum)

Chandler, H.P. 226, 829, 1351, 5059, 5081, 6011 (umbelliferum); 7011 (triquetrum)

Chaney, R.W. 91 (dulcamara)

Chapman, B. 25389 (dulcamara)

Charette, L.A. 1409 (lyratum)

Charlton, D. 2095 (umbelliferum)

Charpin, A. 13451 (dichroandrum); 15109 (dulcamara); 20253 (amygdalifolium); 20518 (aligerum); 20659 (salicifolium); 20976 (laxum); 23904 (valdiviense)

Cházaro, M. 1251 (laxum); 3415, 3416, 5275, 5815 (aligerum)

Chen, H. 10764 (lyratum); 11023 (pittosporifolium)

Chen, N. 42264 (lyratum)

Chen, S. [Shaoqin] 2392 (lyratum); 15919, 16227, 16241, 17170 (pittosporifolium); 17286 (lyratum)

Cheng, W.C. 1177 (lyratum)

Chen Zhao 0960236 (lyratum)

Cheo, H.C. 251 (lyratum)

Cheo, T.Y. 163 (septemlobum); 184 (lyratum)

Chernyakovskaya, E.G. 226 (dulcamara)

Cheseny, C.M.C. 3072 (seaforthianum)

Chesnut, V.K. 69 (umbelliferum)

Chew Wee-Lek 389 (seaforthianum)

Chiang, F. 1200, 1202 (seaforthianum); 7857 (triquetrum)

Chiang, H.C. 8236 (lyratum)

Chiao, C.Y. 358, 3030 (lyratum); 3129 (septemlobum); 12926 (lyratum)

Chiarini, F. 316 (amygdalifolium); 707 (salicifolium); 794, 807, 818, 819 (salicifolium)

China Germany Team 026, 135 (lyratum)

Ching, R.C. 172, 1104 (septemlobum); 8653 (pittosporifolium); 8698, 21378 (lyratum)

Chodat, R.H. 22, 23, 24 (laxum); 38, 39 (amygdalifolium)

Chorley, M. 171 (seaforthianum)

Chow, C.L. 6838, 7261, 8152, 8986 (lyratum)

Chow, H.C. 1896, 8920 (lyratum)

Chow, K.S. 92 (pittosporifolium); 4959 (lyratum)

Christenhusz, M.J.M. 5662 (dulcamaroides)

Christian, S. 64 (umbelliferum)

Chu, K.L. 4085 (lyratum)

Chuang, T.I. 4824 (lyratum)

Chun, W.Y. 5700 (lyratum)

Clare, T.S. 15053 (umbelliferum)

Clark, J.A. 345 (dulcamara)

Clark, J.L. 272, 316, 2121, 6465 (uncinellum); 9098 (laxum); 10597 (boldoense)

Clark, O.M. 1493, 1644, 1770, 1934 (umbelliferum); 4078 (triquetrum); 5031 (umbelliferum)

Clarke, C.B. 31361 (dulcamara); 31365 (pittosporifolium)

Clarke, H.D. 288, 1552, 4235 (uncinellum)

Clarke, O.F. 612 (pubigerum)

Clase, T. 265 (seaforthianum); 1014, 1014 (pyrifolium); 1724 (seaforthianum); 2403 (pyrifolium)

Claude-Joseph [Brother] 600, 2394, 4837, 4838 (valdiviense)

Clausen, J. 467 (umbelliferum)

Claussen, P. 137 (viscosissimum)

Cleef, A. 212, 9708 (stenophyllum)

Clements, F.E. 88 (umbelliferum)

Clokey, I.W. 4432, 4643, 4702, 5047, 5178, 5200, 5348, 5384, 6877, 6878, 6879, 6883 (umbelliferum)

Cobb, D. 19716 (umbelliferum)

Cobbe, T.J. m52 (dulcamara)

Cochrane, T.S. 8524 (pubigerum)

Cocucci, A.A. 30, 106, 108, 109, 110, 111, 112, 113 (salicifolium)

Cohen, A.L. 605 (umbelliferum)

Collins, G.N. 12 (umbelliferum)

Collins, R.S. 239 (umbelliferum)

Collom, R.E. 8 (umbelliferum)

Comanor, P.L. 718, 831 (seaforthianum); 904 (laxum)

Comber, H.F. 371, 735 (valdiviense)

Combs, R. 35 (seaforthianum)

Conceicão, C. de A. 1441 (amygdalifolium)

Congdon, J.W. 135 (umbelliferum)

Conrad, J. 2070 (inodorum); 2124 (flaccidum)

Conrad, M.L. 7193 (umbelliferum)

Constable, E.F. 4903 (seaforthianum)

Constance, L. 412, 788, 2039, 3396, 3447 (umbelliferum)

Contreras, G.I. 239 (pubigerum)

Conzatti, C. 1410 (dulcamaroides); 1602 (pubigerum)

Cook, ? 74 (umbelliferum)

Cooke, P.H. 1423 (dulcamara)

Cooke, W.B. 15180 (umbelliferum)

Cooper, J.G. 316 (umbelliferum)

Cooper, N.C. 2112, 2171, 2201, 2324, 3128, 3229, 3620, 4056, 4057, 4098, 4188, 4390 (umbelliferum)

Cooper, R. 3 (umbelliferum)

Cooper, R.E. 2318 (dulcamara)

Cooper, W.S. 224 (umbelliferum)

Cooray, R.G. 67-102001-R, 70-20308-R (seaforthianum)

Copeland, E.B. 453 (umbelliferum)

Cordeiro, J. 562 (inodorum)

Cordeus, M. 182 (nitidum)

Core, E.L. 1111 (aspersum)

Cornejo Tenorio, G. 2986 (dulcamaroides)

Correa A, M.D. 1584 (seaforthianum); 4241 (seaforthianum)

Correa V, J. 35 (dichroandrum)

Correll, D.S. 14842, 20405, 30667, 30886, 33801 (triquetrum)

Cortés R, C. 95 (seaforthianum)

Cortés, L. 29, 72, 266, 1068 (dulcamaroides)

Cory, V.L. 2367, 11954, 26624, 28410, 35253, 36042, 50553, 55414 (triquetrum)

Costa Sacco, J. da 3, 147, 157, 225, 475, 1231 (laxum)

Costich, D.E. 1506 (pubigerum)

Cotton, J.S. 882 (dulcamara)

Coulter, J.M. 586 (umbelliferum)

Coulter, T. 587, 588, 589, 590 (umbelliferum); 1239 (pubigerum)

Coutrez, G. 3780 (dulcamara)

Covel, P.F. 389 (umbelliferum)

Coville, F.V. 784, 1064 (umbelliferum)

Cowan, C.P. 1783 (seaforthianum)

Cowan, J.M. 2560 (dulcamara)

Cowdry, N.H. 473 (septemlobum)

Craig, T. 214 (umbelliferum)

Cramer, E. 32 (pubigerum)

Cramer, L.H. 3550, 3681 (seaforthianum); 4471 (laxum)

Crampton, B. 9901 (umbelliferum)

Cristóbal, C.L. 1618 (angustifidum)

Croat, T.B. 19352 (uncinellum); 35399 (storkii); 35427 (aligerum); 39360, 41032 (pubigerum); 42337 (aligerum); 44128, 45617, 47350 (pubigerum); 63505 (pubigerum); 64047A (aligerum); 71158 (uncinellum); 73991 (dulcamara); 82018 (nitidum)

Cronemiller, F.P. 3163 (umbelliferum)

Cronise, M.L. 13 (umbelliferum)

Cronquist, A. 2076, 9886 (umbelliferum)

Crosby, M.R. 14380 (umbelliferum)

Crosswhite, F.S. 763 (umbelliferum)

Crow, E. 169 (umbelliferum)

Cruden, R.W. 1231 (dulcamaroides)

Crum, E. 1766, 1912a, 1936a (umbelliferum)

Cruz C., R. 1042 (pubigerum)

Cruz Durán, R. 94 (seaforthianum); 2012 (pubigerum); 2260 (aligerum)

Cruz Espinosa, C.A. 2431 (dulcamaroides)

Cruz, J.M. 136, 191 (inodorum); 229 (laxum)

Cruz, J.S. de la 1284, 1392, 1452, 3226, 3261, 3420, 3583, 4289 (uncinellum)

Cruz, M. 49 (storkii)

Cuadros, H. 3726 (dichroandrum)

Cuamacás, B. 579 (aureum)

Cuatrecasas, J. 1307 (stenophyllum); 4783, 7579 (uncinellum); 9317 (stenophyllum); 11421 (uncinellum); 11471 (aspersum); 13641 (laxum); 15693 (uncinellum); 17780 (seaforthianum); 20339 (stenophyllum); 20605 (aureum); 22406 (uncinellum); 22986 (seaforthianum); 23251, 23452 (stenophyllum); 23881 (uncinellum); 25199 (dichroandrum); 27821 (stenophyllum)

Cuevas, R. 2871 (aligerum)

Cuezzo, A.R. 873 (angustifidum); 1658, 1902, 1952, 2586 (salicifolium)

Culbertson, A.E. 54, 89 (umbelliferum)

Culbertson, J.D. 4323 (umbelliferum)

Cuming, H. 11, 263, 332 (crispum)

Curran, H.M. 31 (angustifidum); 1077 (seaforthianum)

Cutting, C.I. 113 (laxum)

Dahl, H.S. D.45a (dulcamara)

Dahlgren, B.E. 211 (uncinellum)

Dahlström, E. 255 (lyratum)

Dai, T. 102263, 102476, 102779 (lyratum)

Dal Pont, V. 493, 934, 1091 (laxum)

Dammer, U. 903 (aligerum)

Dandy, J.E. 943, 974 (dulcamara)

Daneau, J. 1184 (dulcamara)

D’Angelo, ? 611 (angustifidum)

Daniel [Brother] 969 (laxum); 2956 (seaforthianum); 3281, 3486 (luculentum)

Daniel, T.F. 3438 (umbelliferum)

D’Arcy, W.G. 11917 (pubigerum); 18006 (aligerum)

Davidse, G. 24903 (storkii); 24907 (aligerum); 26105 (storkii)

Davidson, A. 1932, 2303, 2687 (umbelliferum)

Davidson, C. 1516 (wallacei); 1810, 2241, 2653, 2822, 2853, 2877, 3220 (umbelliferum); 3777, 3847, 5095 (aligerum); 5528, 5616 (umbelliferum); 7235 (aligerum); 7241 (storkii); 7380 (umbelliferum)

Davis, A.R. 5, 8 (umbelliferum)

Davis, C.J.S. 10012 (wallacei)

Davis, E.W. 1602 (nitidum)

Davis, J. 1 (wallacei)

Davis, P. 66214, 66416, 66523, 66636, 66666, 67039, 67070 (umbelliferum)

Davis, P.H. 16221, 18783, 21737, 21737, 24854, D37291, 44044, 44905, 47185 (dulcamara)

Davis, T. 806 (pubigerum)

Dawson, G. 1360 (valdiviense); 1501, 1555, 1561 (salicifolium); 1744, 2032 (angustifidum)

Daza, A. 5372 (nitidum)

DCI 903, 1045 (crispum)

DeDecker, M. 1372, 1798, 2494, 2735, 3212, 4877, 6229 (umbelliferum)

Dean, C.C. 46131 (dulcamara)

Debeaux, G. 70 (amygdalifolium)

Defferrard, C. 4506 (seaforthianum)

Degener, O. 16036 (umbelliferum)

Del Vitto, L.A. 852 (salicifolium)

Delgado, A. 3742 (nitidum)

Demaree, D. 8981 (umbelliferum)

Dempster, L.T. 4148, 4271 (umbelliferum)

Deng Shiwei 90622 (lyratum)

Deng, L. 5778 (pittosporifolium); 7490, 8306, 8452 (lyratum)

Dennis, L.R.J. 2323 (dulcamara)

Dennis, R.W.G. 2185 (dichroandrum)

Déséglise, A. 281 (dulcamara)

Desoulavii, N. 322 (dulcamara)

Deventer, R. van 1050 (umbelliferum)

Di Fulvio, T.E. 539 (salicifolium)

Díaz Barriga, H. 1028 (pubigerum); 4680 (aligerum); 6201, 6854 (dulcamaroides); 7046, 7330 (pubigerum)

Díaz, C. 2719 (cutervanum); 4131, 8140 (uncinellum); 8853, 9597, (cutervanum); 9717 (sanchez-vegae); 9779 (cutervanum); 10743 (aureum); 10743 (sanchez-vegae); 10908 (cutervanum)

Díaz V, I. 63 (pubigerum); 207 (dulcamaroides); 440b, 453, 930, 1012, 1068 (pubigerum); 91207 (dulcamaroides)

Dickens, ? 9170 (lyratum)

Diggs, G. 2958 (aligerum)

Dillon, M.O. 1107 (aligerum); 1832 (umbelliferum); 3374 (nitidum)

Ding, G. 1512 (lyratum)

Döbbeler, P. 842 (storkii)

Dobry, K. 408 (umbelliferum)

Dodson, C.H. 1504 (stenophyllum); 5674, 7568 (uncinellum)

Dole, ? 27 (umbelliferum)

Dombrowski, L.T. 4246 (flaccidum)

Domínguez, J.L. 24 (angustifidum)

Dong, J. 0621 (septemlobum)

Donnell Smith, J. 2677 (seaforthianum); 6668, 7538 (storkii)

D’Orbigny, A. 293, 1536 (nitidum)

Dore, W.G. 45555 (dulcamara)

Dorr, L.J. 6207 (aureum); 6415 (sanchez-vegae); 7009 (aligerum); 8764 (uncinellum)

Dorsett, P.H. 1128 (dulcamara); 7235 (septemlobum)

Douglas, D. 1229 (triquetrum)

Douthitt, F.D. 46 (umbelliferum)

Downton, ? 6 (crispum)

Dreisbach, R.R. 3573 (dulcamara)

Dress, W.J. 3046 (umbelliferum)

Dressler, R.L. 459 (umbelliferum)

Dreyfus R, G. 1 (seaforthianum)

Drobov, V.P. 203 (dulcamara)

Drooks, P. 12 (umbelliferum)

Druce, G.C. 1845 (dulcamara)

Drummond, J.R. 95 (triquetrum)

Duarte, A.P. 8621 (viscosissimum)

Dubois, J. 109 (seaforthianum)

Dubugnon, N. 213 (amygdalifolium)

Ducloux [Brother] 2728 (lyratum)

Dudley, W.R. 358, 4379, 4434, 4581 (umbelliferum)

Dugand, A. 280 (seaforthianum)

Dugès, A. 21 (pubigerum); 398 (dulcamaroides)

Duke, J.A. 9868, 11011, 11165 (uncinellum)

Duke, M. 37 (umbelliferum)

Dulong Jiang Investigation Team 1003 (pittosporifolium)

Duncan, C.D. 40 (umbelliferum)

Duncan, T. 2736 (nitidum); 2737 (ruizii)

Duncan, W.B. 166 (umbelliferum)

Dunkle, M.B. 1709 (wallacei); 2533 (umbelliferum); 2873 (wallacei); 2988, 3010, 3570, 3576, 3629, 3894b (umbelliferum); 4713 (wallacei)

Dunn, D.B. 18453, 18975 (pubigerum); 19059 (aligerum); 20703, 20772, 20821, 20899 (umbelliferum)

Duran E, C. 570, 892 (aligerum)

Durkee, L.H. 76-20 (storkii)

Dusén, P. 57 (alphonsei); 658a (odoriferum); 712 (flaccidum); 1575b (laxum); 3258a (flaccidum); 6743 (inodorum); 7203, 7469 (flaccidum); 8156 (inodorum); 8549 (laxum); 8620 (odoriferum); 8764 (flaccidum); 9284 (laxum); 9379, 9379 (viscosissimum); 12114 (inodorum); 12147 (flaccidum); 14210, 14277 (odoriferum); 15756 (laxum)

Eastwood, A. 52, 108, 156, 318, 553, 1772, 1879, 1923, 2316, 2351, 2404, 2727, 2860, 3154, 4028, 4046, 4154, 4186, 4222, 4222A, 4274, 4541, 4728, 4892, 4926, 4966, 4992, 4993, 5074A, 5185, 5348, 5536, 5575, 6441a, 6920, 7599, 8503, 8969, 9146a, 9714, 11656, 12896, 13532, 13602, 13697, 13739, 13836, 13843, 14110, 14120, 14336, 14424, 14942, 15365, 15390, 16799, 16837, 16985 (umbelliferum)

Edge, J.B. 18928 (umbelliferum)

Edmondson, J.R. 3635 (seaforthianum)

Edwall, G. 1816, 1927, 5866 (odoriferum)

Edwards, K.S. 399 (uncinellum)

Edwards, M. 5 (dulcamara)

Edwards, S. 7 (dulcamara)

Egea, J. de 376, 886 (amygdalifolium)

Eggers, H.F.A. 169, 2561, 13126 (seaforthianum)

Eggler, W.A. 411 (pubigerum)

Eggleston, W.W. 9583, 19532 (umbelliferum)

Egido, P. 947 (dulcamara)

Ekman, E.L. 40 (amygdalifolium); H403 (seaforthianum); 816 (amygdalifolium); 817 (laxum); H1697, H5711, H11793 (pyrifolium); H14352 (seaforthianum); 17544 (boldoense)

Elias, Brother 243 (seaforthianum)

Elliott, C. 758 (umbelliferum)

Elmer, A.D.E. 3347, 3552, 3957 (umbelliferum)

Elmore, F.H. 279, 467 (umbelliferum)

Elvin, M.A. 18 (umbelliferum); 377 (wallacei); 2606, 3916 (umbelliferum)

Elword, F.O. 19 (dulcamara)

Elías, T.S. 8332 (umbelliferum)

Emanuelsson, E. 460, 860 (seaforthianum)

Embree, F.W. 84, 229, 260 (umbelliferum)

English, M. 107 (umbelliferum)

Enríquez, O.G. 763 (seaforthianum)

Epling, C. 1850, 6345 (umbelliferum)

Eriksen, B. 45389, 59019 (stenophyllum)

Eriksson, J. 6, 1087, 1146 (septemlobum)

Ern, H. 515 (pubigerum)

Ernst, W.R. 2091 (seaforthianum); 2748 (pubigerum)

Erskine, D. 2384 (dulcamara)

Ertter, B. 8326, 8533 (umbelliferum)

Escobedo, J.M. 465 (dulcamaroides); 686 (pubigerum); 1609 (dulcamaroides); 2850 (pubigerum)

Eskuche, E. 289 (valdiviense)

Espejo, A. 3515 (pubigerum)

Espinal T., S. 2434 (stenophyllum)

Estrada, A. 766 (storkii)

Estrada, E. 2382 (pubigerum)

Estremera, ? 29716 (dulcamara)

Evans, J.B. 80/48 (dulcamara)

Evans, R. 2968 (uncinellum)

Everett, P.C. 21964, 23811 (umbelliferum)

Evrard, C. 10597 (valdiviense); 10636 (crispum)

Ewan, J. 2750 (umbelliferum)

Ewan, J.A. 4029, 8163, 8380 (umbelliferum); 15996 (stenophyllum)

Exp Científica Univ Varsovia 18 (nitidum)

Eyerdam, W.J. 22940 (amygdalifolium)

Eyles, F. 981 (seaforthianum)

Faber, E. 8, 64 (lyratum); 252 (pittosporifolium); 322, 614 (lyratum)

Faber, K. 116 (laxum)

Fagerlind, F. 959 (nitidum); 1500a, 1500b (stenophyllum)

Fan, C.S. 29 (pittosporifolium); 523 (lyratum)

Fang, W.P. 3164, 5503 (lyratum)

Fanshawe, D.B. 1262 (seaforthianum); 5112 (uncinellum)

Farfán, J. 276 (nitidum); 773 (uncinellum)

Farnsworth, E.L. 105 (umbelliferum)

Fassett, N.C. 2783 (dulcamara)

Faulkner, H.G. 656 (seaforthianum)

Faurie, U. 322, 638 (lyratum); 689 (dulcamara); 775 (lyratum); 1088 (pittosporifolium); 1286 (lyratum); 1480 (pittosporifolium); 5536, 5538 (dulcamara); 6001 (lyratum); 7198 (dulcamara); 11506 (lyratum)

Fay, J.J. 00752 (pubigerum)

Fedchenko, B.A. 790, 831 (dulcamara)

Feddema, C. 1936 (uncinellum); 2841 (pubigerum)

Felippone, F. 3083 (amygdalifolium); 3930, 3935 (laxum); 5862 (amygdalifolium); 6949 (laxum)

Fendler, A. 614, 976 (seaforthianum); 2099 (luculentum)

Feng Guomei 4840 (pittosporifolium)

Feng, K.M. 2778 (lyratum); 3343 (pittosporifolium); 11662, 13234 (lyratum)

Ferguson, E. 56 (umbelliferum)

Ferguson, E.V. 270, 450 (umbelliferum)

Ferlatte, W.J. 680 (umbelliferum)

Fernald, M.L. 394, 7789, 24434, 24436 (dulcamara)

Fernández, A. 1503 (aligerum); 12992 (uncinellum)

Fernández Alonso, J.L. 286JF, 2071 (dulcamara)

Fernández Casas, J. 3692 (laxum); 6557, 7793 (aligerum)

Fernández N., R. 1921 (seaforthianum); 2864, 3312 (pubigerum)

Ferraz, E. 273 (flaccidum)

Ferrera Sarmiento, O. 212 (seaforthianum)

Ferreyra, R. 1022 (uncinellum); 2792, 3536, 3796, 5837, 9501, 9796, 14349 (nitidum)

Ferris, R.S. 590, 650, 1869, 4119, 6372, 6591, 6896, 6985, 7558, 8307, 9155, 9760, 9836, 9896 (umbelliferum)

Feuerer, T. 4283b, 10329, 23037 (aligerum)

Fiebrig, K. 740 (laxum); 939, 4079 (amygdalifolium); 5463a, 5731 (laxum); 5848 (uncinellum)

Fiker, C.B. 3540 (umbelliferum)

Filipovich, R. 66 (endoadenium)

Filippa, E. 86 (salicifolium)

Firmin, G. 304 (laxum)

Fisher, G.L. 125 (umbelliferum); 302 (pubigerum); 32248 (triquetrum); 35458 (pubigerum); 43037 (triquetrum)

Fisher-Meerow, L.L. 804 (pyrifolium)

Fleet, C. van 9 (umbelliferum)

Flores F., G. 234 (aligerum); 837 (pubigerum)

Flores M., A. 268, 1054, 1072 (aligerum)

Flores M., D. 234, 268 (aligerum)

Flores, J. JF00133 (seaforthianum)

Flügel, H.J. 6075 (laxum)

Focke, A. 57, 616 (uncinellum)

Fogg, J.M. 10784 (dulcamara)

Foggitt, T.J. 1134b (dulcamara)

Follett, W.I. 117 (umbelliferum)

Font Quer, P. 354 (dulcamara)

Ford, H.C. 1447 (umbelliferum)

Ford, S.A. 42 (dulcamara)

Forest, H. de 17369 (umbelliferum)

Forrest, G. 5922 (lyratum); 8349 (pittosporifolium); 8630, 14603 (lyratum)

Försskal, P. 418 (dulcamara)

Fosberg, F.R. S4285, S4812 (wallacei); S5029, S5079, S5123, 8002, 8003, 8004 (umbelliferum); 8168 (wallacei); 8186, 8395, 8571, 8587 (umbelliferum); 21924 (seaforthianum); 21986 (laxum); 51912, 53074 (seaforthianum); 54980 (umbelliferum); 56368, 57171, 58962 (seaforthianum); 59460 (laxum); 66155 (umbelliferum)

Fosris, H.A. 7947 (salicifolium)

Foster, R.B. 3264, 3365, 8900, 12047 (uncinellum)

Foster, R.C. 32, 119a (umbelliferum)

Fox, ? 182 (laxum)

Fox, H.E. 738, 873b, 1200, 1208 (dulcamara)

Fraga, C.N. 2885 (inodorum)

Franceschi, F. 15 (umbelliferum)

Franklin, M.A. 5835 (umbelliferum)

French, N. 24, 55, 159, 261, 341, 368 (umbelliferum)

Fries, R.E. 1617 (amygdalifolium)

Fritchey, ? 9 (umbelliferum)

Froes, R. 1895, 11538 (uncinellum)

Frost, W.T. 429 (umbelliferum)

Frost-Olsen, P. 1748, 4401 (dulcamara)

Frumpkin, G. 2 (dulcamara)

Frye, T.C. 2518 (triquetrum)

Fryxell, P.A. 3224 (dulcamaroides)

Fu, K. 6130, 18757 (lyratum)

Fuentes O., M. 546 (dulcamaroides); 650, 820 (pubigerum)

Fuentes, A. 8507 (cutervanum)

Fuertes, M. 1901 (pyrifolium)

Fulton, H.J. 9674 (umbelliferum)

Funck, H.C. 46 (seaforthianum); 1126, 1621 (dichroandrum)

Funk, V.A. 2239 (pubigerum); 3031 (aligerum)

Furse, P. 8296, 9071 (dulcamara)

Furuse, M. 6434, 8980, 9464, 9465 (dulcamara); 10039 (lyratum); 18771 (pittosporifolium); 19609 (lyratum); 20284 (pittosporifolium); 29993 (dulcamara); 33992 (pittosporifolium); 35686 (dulcamara); 40462 (pittosporifolium); 42671, 42703 (lyratum); 44346, 44636 (pittosporifolium); 47217 (lyratum)

Förther, H. 48 (dulcamara)

Gadeceau, E.J.A. 5106 (dulcamara); 5106 (seaforthianum). [two different plants with the same number; different collection details, see Solanaceae Source]

Galander, C. 18 (salicifolium)

Galeotti, H.G. 1163 (pubigerum); 1183 (dulcamaroides); 1219, 1225R, 1227 (pubigerum)

Galiano, W. 4225 (aligerum); 4753, 5352 (nitidum); 6137 (kulliwaita)

Gallardo Hernández, C. 878, 936, 1016 (aligerum)

Gallardo, G. 1796 (aligerum)

Gallegos, J.M. 551 (umbelliferum)

Gallinal H., J.P. PE-4500 (laxum)

Galán Cera, P. 42, 1361, 1763, 1977, 2182 (dulcamara)

Gamarra, P. 447 (nitidum)

Gamble, J.S. 10042, 10557 (pittosporifolium)

Gamboa, B. 281 (aligerum); 727, 911, 938 (storkii)

Gamerro, J.C. 67 (laxum)

Gander, F.F. 109 (umbelliferum)

Gandhi, K.N. 2060 (seaforthianum)

Gaoligong Shan Biodiversity Survey 28858 (pittosporifolium)

Gaoligong Shan Expedition 1996 8039 (pittosporifolium)

Gaoligong Shan Expedition 1997 8738 (lyratum)

Garafolo, B. 981 (seaforthianum)

García Adá, R. 1053, 1319, 1816, 2738, 3080 (dulcamara)

García Guevara, G. 24 (pubigerum)

García M., A. 581 (aligerum); 1740, 1740, 2897 (pubigerum)

García Mendoza, A. 581, 582, 1287, 1287 (aligerum); 2714 (dulcamaroides)

García P., J. 628 (pubigerum)

García Torres 30 (pubigerum)

García, E. 151 (nitidum)

García, E.M. 360 (salicifolium)

García, R. 1161 (pyrifolium)

García-Barriga, H. 10830 (laxum); 11753 (seaforthianum); 13236, 13295 (dichroandrum); 13335 (luculentum); 20721 (dichroandrum)

García-Mendoza, A. 582, 650 (pubigerum)

Gardner, G. 558 (flaccidum); 2427 (seaforthianum)

Gardner, M.F. DCI-1 (valdiviense); 6723 (crispum); 6726 (valdiviense); 6734, 6776 (crispum); 6853 (valdiviense); 6870 (crispum); 6914 (valdiviense)

Garganta, M. de 2167 (luculentum)

Garrett, H.B.G. 1342 (seaforthianum)

Garwood, N.C. 351 (pubigerum)

Gastony, G.J. 205, 525 (pyrifolium)

Gat, S. 23, 31 (umbelliferum)

Gauba, D.E. 829 (dulcamara)

Gaudichaud, C. 169 (valdiviense); 308 (inodorum); 312 (flaccidum)

Gaumer, G.F. 841 (seaforthianum)

Gay, C. 212, 674 (valdiviense)

Gebhard, H. 63 (amygdalifolium)

Geerinck-Coutrez 3121, 4343 (dulcamara)

Gentry, A.H. 9607, 10138 (uncinellum); 19208 (nitidum); 19623 (uncinellum); 20316, 20390 (pubigerum); 23279 (nitidum); 28266, 31481 (uncinellum); 37394, 37500 (nitidum); 44346 (uncinellum); 50641 (seaforthianum); 56194 (uncinellum); 56194 (uncinellum); 57183 (dichroandrum); 64773 (uncinellum); 74570, 74897 (cutervanum); 75070 (uncinellum)

Gentry, H.S. 38 (triquetrum); 3096 (umbelliferum); 18420 (triquetrum); 19588 (pubigerum); 19696, 19793 (umbelliferum)

Gentry, J.L. 2673, 2953 (storkii)

Gereau, R.E. 2076 (aligerum)

Gerrans, M.B. 31, 1098, 1356, 1476, 1609, 1692 (dulcamara)

Geshetz, E.E. 1412 (dulcamara)

Ghiesbreght, A.B. 100 (pubigerum); 638 (pubigerum)

Gibbons, R.B. 797 (dulcamara)

Gibby, M. 172 (nitidum)

Gibert, E.J. 5 (laxum); 8 (amygdalifolium); 141 (laxum)

Gibert, M. 34 (angustifidum)

Gibson, A.C. 1862 (umbelliferum)

Gifford, A.D. 121, 402 (umbelliferum)

Giles, V.V. 53 (umbelliferum)

Gillespie, J.W. 5298 (umbelliferum)

Gillespie, L.J. 1112 (uncinellum)

Gillett, J.M. 6922 (dulcamara)

Gillis, W.T. 10223 (pubigerum)

Gilly, C.L. 8 (pubigerum)

Gilman, M.F. 1775 (umbelliferum)

Gines, H. 4965 (uncinellum)

Giraldo-Cañas, D.A. 1068 (luculentum)

Gitonga, F. 284 (seaforthianum); 285 (laxum)

Glanville, D. 85 (dulcamara)

Glaziou, A.F.M. 8844 (laxum); 8855, 12100 (flaccidum); 13087 (amygdalifolium); 14176 (odoriferum); 15300 (inodorum); 15304 (viscosissimum); 18407 (odoriferum); 18409, 18409a (viscosissimum)

Gleason, H.A. 123, 269, 1404 (dulcamara)

Gnezdillo, A. 97 (dulcamara)

Goddard, D.R. 611 (umbelliferum)

Godfrey, R.K. 66636 (pubigerum); 66712, 66780 (aligerum)

Godman Dame Alice 218, 247 (umbelliferum)

Goldman, D. 3174, 3283 (umbelliferum)

Goldsmith, P. 64 (pubigerum)

Gómez, J. 144, 144 (nitidum)

Gómez C., G. 91 (pubigerum)

Gonde, P. 88/74 (seaforthianum)

Gonzales, A. 177 (boldoense)

Gonzales-Villarreal, L.M. 4394 (pubigerum)

González Medrano, F. 10678 (dulcamaroides)

González Parini, F. 1254 (uncinellum)

González R., A. 314 (umbelliferum)

González, E. 316, 424 (pubigerum); 698 (seaforthianum)

González, J. 1840 (aligerum); 2481 (seaforthianum)

González, L. 973 (storkii)

González, L.M. 11 (umbelliferum)

González, R. RGM-6 (storkii)

González-Espinosa, M. 1495 (aligerum)

Goodding, L.M. 8-42, 17-47, 1083 (umbelliferum); 2181 (pubigerum)

Goodman, G.J. 3438 (pubigerum)

Gorman, M.W. 3575 (dulcamara)

Goruz, F. 81 (pubigerum)

Gould, F.W. 2116, 2215, 2308, 2325, 4163 (umbelliferum); 13644 (seaforthianum)

Gounelle, ? 47 (viscosissimum)

Graham, C.A. 259 (umbelliferum)

Graham, G.J. 260, 261 (pubigerum)

Grannell, R. 2236 (umbelliferum)

Grant, A.L. 51A, 51, 452 (umbelliferum)

Grant, G.B. 1218 (umbelliferum); 1220, 3725, 3780 (wallacei); 4570, 5378 (umbelliferum)

Grant, J.R. 921843 (storkii)

Grant, M.L. 10300 (laxum); 16064 (dulcamara)

Granville, J.J. de 10046, 11875, 13686 (uncinellum)

Granzow, I. 459, 567 (dulcamara)

Grauer, D. 136 (umbelliferum)

Gray, S. 6 (umbelliferum)

Grayum, M. 9110 (seaforthianum)

Grazziotin, G. 191 (laxum)

Greatrex, F.C. 11435 (lyratum)

Greene, E.L. 877 (umbelliferum)

Greene, J. 224 (umbelliferum)

Gregory, D.P. 292 (pubigerum)

Greuter, W. 16361, 16703, 17348 (dulcamara); 21464 (seaforthianum)

Griffith, W. 5901 (dulcamara)

Griffiths, D. 6274 (triquetrum)

Grifo, F.T. 216 (pyrifolium)

Grigoreev, S.G. 20 (dulcamara)

Grigoriev, Y. 757 (dulcamara)

Grijalva, A. 420 (uncinellum)

Grinnell, F. 321, 420, 534, 3367 (umbelliferum)

Grinnell, H.W. 213, 1158 (umbelliferum)

Groot, S.J. de 219 (umbelliferum)

Gross, C.A. 109, 209 (umbelliferum)

Gross, L. 1832, 2698 (umbelliferum)

Grossheim, A. I27 (dulcamara)

Grotta, A.S. SPF15112 (flaccidum)

Groves, E.W. 2838 (dulcamara)

Grubb, P.J. 120 (uncinellum); 215 (stenophyllum); 737a (luculentum); 1645 (uncinellum)

Grubov, V.I. 32 (dulcamara)

Guan, K. 1824 (pittosporifolium)

Guangdong Prov. 1978 Team 5908 (lyratum)

Gubanof, I.A. 7321 (dulcamara)

Guerra C., I.R. 181 (seaforthianum)

Guerra, J. 1518 (dulcamara)

Guerrero, E. 15 (salicifolium)

Gueua, J.A. 839 (laxum)

Guevara Amórtegui, H. 237 (laxum)

Guevara, R. 6 (salicifolium)

Guillén, R. 393 (amygdalifolium)

Guinea, E. 895 (dulcamara)

Guiol, F.G. 642 (dulcamara)

Guirado, F. 633 (umbelliferum)

Guizar Nolazco, E. 4230 (pubigerum)

Guo Chengyong 5470-5, 51386-1, 52414-1 (lyratum); 54428-5 (pittosporifolium)

Gust, G. 562 (umbelliferum)

Gustafson, R. 963 (umbelliferum); 1896 (nitidum)

Gutiérrez, M. 203 (aligerum)

Gutiérrez V., G. 1457 (seaforthianum)

Guttermans, A. 9 (flaccidum)

Günther, E. 5842 (aligerum)

Guzmán, M. 741, 741 (seaforthianum)

Haasis, F.W. 49.59 (umbelliferum)

Haenke, T. 1158 (pubigerum)

Hagelund, K. 13164, 13305, 13464, 15346 (laxum)

Hagemann, I.H. 2883B (laxum)

Hahn, L. 30 (dulcamaroides)

Hahn, W. 544 (pubigerum); 673, 1474 (amygdalifolium); 1952, 2286 (laxum)

Hale Jr, M.E. 20738 (pubigerum)

Haley, B.R. 208 (dulcamara)

Hall, E. 494, 494 (triquetrum)

Hall, H.M. 7, 93, 141, 355, 420, 436, 448, 930, 1224, 1237.5, 1273.5, 1382, 1427, 1545, 2071, 2263, 2827, 2929, 3000, 3102, 3133, 3165, 3261, 6266, 6487, 7148, 7631, 7789, 7837, 7856, 8501, 8631, 9064, 11243, 12735, 12764 (umbelliferum)

Hallberg, B. 879 (aligerum)

Halliday, G. 163/67 (dulcamara)

Hallé, F. 573, 597 (uncinellum)

Halse, R.R. 6149 (umbelliferum)

Hamilton, A.C. 551 (sanchez-vegae)

Hamilton, C. 2875 (uncinellum)

Hammar, A. 5865 (odoriferum)

Hammel, B. 13920, 16925 (uncinellum); 19409 (seaforthianum); 19815 (aligerum); 21289 (uncinellum)

Handel-Mazzetti, H.F. 12401 (pittosporifolium)

Hangzhou Bot. Gard. Herb. 29999 (septemlobum)

Hansen, G. 117, 1284, 1379 (umbelliferum)

Hansen, L. 917 (dulcamara)

Hanson, S.C. 14 (umbelliferum)

Hao, K.S. 418, 531, 3804 (septemlobum); 3832, 4192 (lyratum)

Happ, G.B. 61 (pubigerum)

Hara, H. 2771 (pittosporifolium)

Hara, T. N8237 (pittosporifolium)

Haradjian, M. 481 (dulcamara)

Hardham, C.B. 154, 158, 218, 853, 1628, 1697, 2174, 2181, 3035, 3047, 3056, 3164, 3343, 4035, 4067, 4268, 4306, 4385, 4607, 4621, 4645, 4812, 4836, 6198, 12214 (umbelliferum)

Harley, R.M. 50631 (laxum)

Harling, G. 4249 (stenophyllum); 8218, 8218 (aureum); 8454 (cutervanum); 8586 (stenophyllum); 8732 (kulliwaita); 12534 (stenophyllum); 12671 (kulliwaita); 13239 (aureum); 15217 (seaforthianum); 23518 (sanchez-vegae); 26713 (cutervanum)

Harmon, W.E. 6126 (aligerum)

Harris, B.B. 711 (triquetrum)

Harris, B.J. 2965 (seaforthianum)

Harris, W. 11959 (seaforthianum)

Harrison, G.J. 1712, 7806 (umbelliferum)

Hart, C. 21, 24, 88 (umbelliferum)

Harthill, M.P. 93, 1693 (umbelliferum)

Hartweg, K.T. 260 (valdiviense); 367 (dulcamaroides); 812 (leiophyllum); 1294 (seaforthianum); 1876 (umbelliferum)

Harvard Course in Tropical Botany 671 (pyrifolium)

Hassler, É. 878 (amygdalifolium); 1608 (laxum); 1624 (amygdalifolium); 1625 (laxum); 1793 (amygdalifolium); 2504 (laxum); 3207 (amygdalifolium); 3320, 3826, 4093 (uncinellum); 4131, 5342 (laxum); 7193 (amygdalifolium); 11288 (flaccidum); 11756 (amygdalifolium); 11990 (seaforthianum)

Hastings, G.C. 73 (umbelliferum)

Hastorf, C. 34 (nitidum)

Hatschbach, G. 1217 (flaccidum); 2465 (inodorum); 2685 (flaccidum); 6348 (viscosissimum); 7329 (flaccidum); 7385 (viscosissimum); 8026 (flaccidum); 9724 (laxum); 9827, 10151 (inodorum); 10892 (flaccidum); 12539 (laxum); 12809 (flaccidum); 13166 (odoriferum); 13704, 14962 (laxum); 15018 (inodorum); 16991 (laxum); 19019 (flaccidum); 20368 (odoriferum); 21450 (seaforthianum); 22756 (flaccidum); 24478 (inodorum); 31248, 34464 (seaforthianum); 41897 (laxum); 42853 (viscosissimum); 44362, 45169 (flaccidum); 47097 (laxum); 60388 (seaforthianum); 72322 (laxum)

Hatusima, S. 14243 (lyratum)

Haught, O.L. 2934, 3250 (nitidum); 4128 (seaforthianum); 5248, 6071 (uncinellum)

Hausmann, F. von 828 (dulcamara)

Hawkes, A.D. 4909 (umbelliferum)

Hawkes, J.G. 408 (seaforthianum)

Hazen, T.E. 11836 (uncinellum)

Heckard, L.R. 1460, 1785, 2454, 2742, 3366, 5144, 5554, 6311 (umbelliferum)

Heerdt, ? van 466 (umbelliferum)

Heiser, C.B. 1902 (umbelliferum); 3489 (storkii); 3601 (pubigerum)

Hellenberg, H. 4484 (aligerum)

Heller, A.A. 1399 (triquetrum); 5076, 5407, 5455, 6451, 7248, 7248, 7706, 7899, 8889, 11361, 11814, 13144, 16040 (umbelliferum)

Helmkamp, G.K. 1459, 1459, 3247, 8445 (umbelliferum)

Hemphill, F. 616, 673, 688, 747, 751 (umbelliferum)

Henderson, L.F. 723, 1379, 6050 (umbelliferum)

Hendrix, T.M. 145, 220, 754 (umbelliferum)

Henkel, T.W. 3418 (uncinellum)

Henrich, J.E. 454 (dulcamaroides); 454 (seaforthianum)

Henrickson, J. 14540, 19908, 19982, 20030, 20073, 20711 (umbelliferum)

Henry, A. 110, 635, 699, 1537 (lyratum); 2048 (pittosporifolium); 2098, 2198 (lyratum); 2334, 2334A, 5824, 7476, 10353 (pittosporifolium); 11855 (lyratum)

Henry, W.R. 530 (dulcamara)

Herb E.S. Gregory 188b (dulcamara)

Herb Sowerby 376 (dulcamara)

Herb. Arthur Bennett 1015b (dulcamara)

Herb. Fischer 1438 (viscosissimum)

Herb. F.B. Forbes 8780 (lyratum)

Herb. H.J. Riddelsdell 1208 (dulcamara)

Herb. J.T.I. Boswell-Syme 738 (dulcamara)

Herb. L.B. Hall 306-1134 (dulcamara)

Herb. Marin 2138 (aligerum)

Herb. Miers 1435 (salicifolium); 7886 (valdiviense)

Herb. Vatke 3855 (pubigerum)

Herb. Willing 4624, 13289a, 13418D, 14091D, 14140D, 15441D, 15543D, 18793a, 21223, 48029, 55434, 59887, 60002, 60612, 60744, 67769, 67904, 68568, 69687, 71725, 86148, 118384, 119179, 119345, 159641 (dulcamara)

Herbert, H. 3 (seaforthianum)

Herbst, D. 700, 5558 (seaforthianum)

Heringer, E.P. 9139 (viscosissimum)

Hermann, H.A. van 454, 5080 (seaforthianum)

Hermanos Cristianos 59 (seaforthianum)

Hernández, R. 1037 (aligerum)

Hernández M., R. 1037 (aligerum); 1062, 3270 (pubigerum); 3610 (dulcamaroides); 4189 (aligerum); 5331 (laxum); 5339, 5364, 5806, 5849a (aligerum); 6307 (pubigerum); 6378, 6378 (aligerum); 7252 (pubigerum); 9537 (dulcamaroides)

Hernéndez, C.G. 878, 1264 (aligerum)

Hernéndez, H.M. 853 (pubigerum); 901 (dulcamaroides)

Herrera C., N. 129 (pubigerum)

Herrera, F.L. 1070 (nitidum); 2986 (aligerum)

Herrera, G. 3734 (storkii)

Herrera, L. 19 (nitidum)

Herter, W.G. 190 (amygdalifolium); 1516c, 1516b, 19182 (laxum)

Hesse, V.F. 2292, 2413, 2909 (umbelliferum)

Hewer, T.F. 1274, 3961 (dulcamara)

Heyde, E.T. DS3821 (seaforthianum)

Hicken, C.M. 98, 24941 (laxum)

Hickernell, R.T. 14 (umbelliferum)

Hieronymus, G. 50 (salicifolium); 78 (endoadenium); 90, 220, 429, 473 (salicifolium); 581 (endoadenium); 622, 642 (salicifolium); 746 (endoadenium); 812, 813 (salicifolium); 868 (aligerum)

Hilend, M. 58, 150 (umbelliferum)

Hill, S.R. 17526 (storkii)

Hillier, M. 1195 (valdiviense)

Hilton, T. 1, 34, 1015 (dulcamara)

Hincher, R. P-1270 (macbridei)

Hinckley, L.C. 2120, 3628 (triquetrum)

Hinton, G.B. 272 (pubigerum); 365 (aligerum); 633, 835 (dulcamaroides); 855 (pubigerum); 1360, 1697 (dulcamaroides); 2879 (pubigerum); 3852 (aligerum); 3998 (pubigerum); 10516, 11857 (dulcamaroides); 11901 (pubigerum); 12255 (seaforthianum); 13951 (dulcamaroides); 16775, 16855, 20406, 21115 (triquetrum); 24534 (pubigerum)

Hitchcock, A.S. 17487 (uncinellum); 20947, 21497 (aureum)

Hitchcock, C.L. 5044, 6014, 6590 (umbelliferum); 6977, 7205 (pubigerum); 22170, 22170, 23103 (umbelliferum)

Hjerting, J.P. 623, 1031 (nitidum)

Hjorih-Olsen, B. 10 (dulcamara)

Hodge, W.H. 3913 (seaforthianum)

Hodgson, E. 738 (dulcamara)

Hoefs, M. 79 (wallacei)

Hoehne, F.C. 724 (flaccidum); 3375 (inodorum); 3493 (flaccidum); 3831, 9715 (inodorum); 12910 (flaccidum); 19543, 29800 (inodorum)

Hoehne, W. SPF15383 (inodorum); SPF15754 (flaccidum)

Hoeltzer, ? 352 (dulcamara)

Hoffman, B. 635, 2673, 5268, 5468 (uncinellum)

Hoffman, F.W. 1334, 1591 (umbelliferum)

Hoffmann, R. 342 (umbelliferum)

Hohenacker, F. 107 (valdiviense); 421, 2231, 2608, 3973 (dulcamara)

Hohenthal, W. 1 (umbelliferum)

Holdridge, L.R. 935 (pyrifolium)

Hollermayer, A. 324 (crispum); 1161 (valdiviense)

Hollermayer, P.A. 323 (valdiviense)

Holm, R.W. 142, 142, 515 (storkii)

Holm-Nielsen, L.B. 1541 (aureum); 5115 (agnoston); 5207, 6321 (aureum); 23703 (stenophyllum); 24654 (aureum); 25017, 25017, 25149, 28080, 28691 (stenophyllum); 29707 (aureum); 41969 (stenophyllum)

Holmgren, I. 577 (laxum)

Holt, E.G. 651 (uncinellum)

Holway, E.W.D. 815 (pubigerum)

Honer, M. 1993 (umbelliferum)

Hoogte, L.D.V. 1045, 2091 (nitidum)

Hoover, R.F. 2161, 2726, 4281, 6111, 6158, 6675, 6705, 6756, 7426, 7879, 8304, 8560, 8636, 8765, 8830, 8987, 9987, 10211, 10216, 11014, 11264, 11536, 11574 (umbelliferum)

Hopkins, M.J.G. 674 (uncinellum)

Hopping, R. 189 (umbelliferum)

Horner, C. 418 (uncinellum)

Hosseus, C.C. 75 (angustifidum); 616, 1009, 1009bis, 1958, 2567 (salicifolium)

Hostmann, F.W.R. 175, 176, 1100, 1599 (uncinellum)

Hotchkiss, N. 6139 (triquetrum)

Hotta, M. 16792 (lyratum)

Hou, K. 74591 (lyratum)

Hough, A.F. 13 (umbelliferum)

Howard, R.A. 9423 (pyrifolium); 10436 (seaforthianum); 12255 (boldoense); 12292 (pyrifolium); 15285, 16337, 16347, 19034 (seaforthianum)

Howell, A.H. 306 (triquetrum)

Howell, J.T. 149, 363, 749, 1173, 2741, 2822, 3775, 4508, 4588, 4793, 5100, 5512, 5951, 6128, 6236, 6507, 6670, 8060, 10979, 11033, 11291, 11316, 11352 (umbelliferum); 12411 (dulcamara); 12596, 14606, 15234, 17251, 18806, 19818, 20780, 20861, 21248, 23686, 24271, 24292, 25377, 27437, 27644, 29766, 29911, 30299, 30564, 30955, 33912, 34103, 34573, 35273, 38258, 39002, 39633, 41890, 42082, 43164, 44368, 47100, 47783, 48526, 51769, 53504, 53825 (umbelliferum)

Howitt, B.F. 143, 1322, 1395, 1852 (umbelliferum)

Hsia, W.Y. 3740 (septemlobum)

Hsiung, Y.K. 5576 (pittosporifolium)

Hu, S.Y. 317 (seaforthianum); 1578, 1982 (lyratum); 10366 (seaforthianum)

Huamán, E. 483 (uncinellum)

Huang, C. 162696 (lyratum)

Huang, M. 111322 (pittosporifolium); 112416 (lyratum); 112640 (pittosporifolium)

Huang, S. 190030 (lyratum)

Huang, T.C. 15045 (lyratum)

Huapalla, J. 3586, 3668 (nitidum)

Huaylla, H. 676 (aligerum)

Hubby, F.W. 127 (umbelliferum)

Huber, W. 1011 (storkii)

Huerta B., V.M. 160 (dulcamaroides)

Huerta M., M. 230 (dulcamaroides)

Huft, M. 2144 (pubigerum)

Hugh, F. 20, 27, 82 (septemlobum)

Humbles, J. 6067 (aureum)

Humboldt, F.W.H.A. von 3069 (nitidum); 3142 (aureum)

Hunn, E. OAX-902 (aligerum); OAX-1493 (dulcamaroides)

Hunnewell, F.W. 7889 (umbelliferum); 17854, 19218 (dulcamara); 19760 (seaforthianum); 19989 (seaforthianum)

Hunt, D.R. 6684 (viscosissimum)

Hunziker, A.T. 161 (amygdalifolium); 2267 (angustifidum); 3616 (amygdalifolium); 4752 (angustifidum); 5193, 5217, 6942, 7559, 7903, 8455, 8543, 9836 (salicifolium); 10005 (aligerum); 10357 (angustifidum); 10372 (amygdalifolium); 11638, 11667, 11798, 12244, 12602, 13434, 13826 (salicifolium); 17773 (amygdalifolium); 18022 (salicifolium); 18555, 19075 (endoadenium); 19509 (aligerum); 20349 (endoadenium); 20476, 20477, 20514, 20533 (salicifolium); 21931 (endoadenium); 22611 (angustifidum); 22850 (endoadenium); 23669, 23759, 23806, 23865, 24188 (salicifolium); 24873 (aligerum); 24875 (endoadenium); 24876, 24882 (salicifolium); 24909 (endoadenium); 25043, 25075, 25321 (salicifolium); 25443 (amygdalifolium); 25550 (endoadenium)

Hunziker, J.H. 1825 (angustifidum); 1888, 1963, 2087, 4342 (salicifolium)

Hunziker, J.J. 11638 (salicifolium)

Hutchison, P.C. 1243 (nitidum); 4191 (nitidum); 5738 (sanchez-vegae); 7233 (nitidum)

Ibarra C., G. 239 (pubigerum)

Ibarrola, T.S. 887 (amygdalifolium); 1001, 1137 (laxum); 3188 (angustifidum)

Iltis, H.H. 111 (dulcamaroides); 116 (pubigerum); 455, 777 (nitidum); 1115 (dulcamara); 1690 (pubigerum); 2218, 2336 (aligerum); 3164 (dulcamaroides)

Imboden, W.H. 39 (triquetrum)

Innes, R.R. 313 (triquetrum)

Inzunza, F.M. 103, 114 (aligerum)

Irigoyen, J. 248 (amygdalifolium)

Irwin, H.S. 5392, 6221, 11355, 18165, 18166, 18293, 22864, 27093 (viscosissimum); 55565 (uncinellum)

Isern, J. 2061 (nitidum)

Izuzquiza, A. 2754 (dulcamara)

Jack, J.G. 3526, 3644 (dulcamara); 5518 (seaforthianum); 6823 (boldoense)

Jackson, A.B. 83, 1134b (dulcamara)

Jahn, A.J. 1266 (seaforthianum)

Jameson, W. 14 (stenophyllum); 46 (aureum); 48, 128, 248, 271 (stenophyllum); 463 (aureum); 654 (stenophyllum)

Jansen-Jacobs, A. 6290 (uncinellum)

Jansen-Jacobs, M.J. 364, 3187 (uncinellum)

Jaramillo, J. 8810 (leiophyllum); 8832 (agnoston); 10116 (stenophyllum); 10606 (coalitum); 12110 (aspersum)

Jaramillo, N. 1310 (uncinellum)

Jardim, A. 2048 (aligerum)

Jasso, M.J. 261 (pubigerum); 532, 1761 (aligerum)

Jayasuriya, A.H.M. 1385, 1409, 1426 (seaforthianum); 1458 (laxum)

Jayasuriya, M. 1936 (seaforthianum)

Jelski, C. von 30 (cutervanum)

Jenkins, ? 2946 (dulcamara)

Jenman, G.S. 2096, 2123, 7237, 7824 (uncinellum)

Jensen, H.A. 25, 73 (umbelliferum)

Jensen, L. 464 (dulcamara)

Jepson, W.L. 5535, 6716, 8464, 9097, 9151, 11839, 11946, 12000, 12109, 12801, 15411, 15884, 17464, 18395, 18758, 18910, 19124, 20666, 20765, 21257, 21258, 21259, 21259, 21260, 21261, 21262 (umbelliferum)

Jeremie, J. 983 (seaforthianum)

Jermy, A.C. 12125 (dulcamara)

Jermy, G. 80, 281 (triquetrum)

Jiangpu Team 8292 (septemlobum)

Jiménez, B. 0183, 1742 (laxum)

Jiménez, F. 97 (pyrifolium)

Jiménez M., A. 2989 (aligerum)

Jin Yilang, Y. 322 (lyratum)

Job, M.M. 548 (angustifidum); 734, 948 (amygdalifolium)

Johannsen, P.L. 46, 224, 238, 421, 1135 (umbelliferum)

Johns, T. 81-25, 81-25, 81-92, 81-92 (nitidum)

Johnson, A.M. 4401 (umbelliferum)

Johnson, E.R. 1344 (umbelliferum)

Johnson, F.W. 1481 (wallacei); 1687 (umbelliferum)

Johnson, M.P. 10051 (umbelliferum)

Johnston, G.R. 14934 (umbelliferum)

Johnston, I.M. 118, 1024 (umbelliferum); 1067 (triquetrum); 2094 (umbelliferum); 7639, 7640, 8259 (triquetrum)

Johnston, J.C. 1727 (muenscheri)

Johnston, J.R. 1101, 1210, 1352 (pubigerum); 1978 (dulcamaroides)

Johnston, M.C. 4527 (seaforthianum)

Jones, M. 20 (dulcamara)

Jones, M.E. 371, 2223 (umbelliferum); 29195 (triquetrum)

Jones, W.W. 235 (umbelliferum)

Jönsson, G. 658a (odoriferum); 894a (laxum); 1084a (flaccidum)

Jordan, C.G. 253 (aligerum)

Jörgensen, P. 298 (pubigerum); 1310 (endoadenium); 1468 (aligerum); 2816 (angustifidum); 2827 (amygdalifolium); 3667, 3668 (laxum); 4657 (amygdalifolium); 10784 (aligerum); 11358 (endoadenium); 88791 (aligerum)

Jørgensen, P.M. 157 (aureum); 477 (stenophyllum); 747, 786 (cutervanum); 1068 (stenophyllum);1397, 1463 (aureum); 1863 (leiophyllum); 2067 (nitidum); 2188 (coalitum); 2986 (uncinellum); 36423, 36424 (aligerum); 61778, 61948 (stenophyllum); 65663 (stenophyllum); 65686 (coalitum); 88791 (aligerum)

Joyal, E. 1272 (umbelliferum)

Juárez G., L.G. 68 (aligerum)

Julio, Brother 121 (nitidum)

Junak, S.A. SC-522, SC-819, SC-1262, SC-1492, SC-2120, SC-3873 (umbelliferum)

Juncosa, A. 2335 (aureum)

Junge, C. 54, 54 (valdiviense)

Jurgensen, C. 298 (pubigerum)

Jury, S.L. 11718 (dulcamara)

Jutila, J. 268 (lyratum)

Juzepczuk, S. 10708 (nitidum)

Kalheber, H. 78-424 (dulcamara)

Kamb, P. 955 (umbelliferum)

Kanai, H. 6262 (pittosporifolium)

Kanda, M. 1 (dulcamara)

Kang, ? 26 (septemlobum)

Kappler, A. 34, 1599 (uncinellum)

Karwinski, W.H. 585, 585bis (seaforthianum)

Kasapligil, B. 4074, 4546 (umbelliferum)

Katende, A.B. K1379 (seaforthianum)

Kawahara, S. 464 (umbelliferum)

Kayama, K. 6658, 6676 (lyratum)

Kayap, R. 154, 160, 376 (uncinellum)

Kearney, C. 132 (dulcamara)

Keck, D.D. 52, 1413, 2176, 2619, 2818, 2976 (umbelliferum)

Keefe, J. 14-005, 15767 (umbelliferum)

Keel, S. K466 (aligerum)

Kegarice, L. 9 (umbelliferum)

Kegel, H. 1294 (uncinellum)

Kegler, A. 305 (laxum); 390 (flaccidum); 843 (laxum)

Kehlor, R.R. 12 (umbelliferum)

Keil, D. 3541, 28704 (umbelliferum)

Kellerman, W.A. 3928, 5801 (pubigerum); 5879 (seaforthianum)

Kellogg, A. 717 (umbelliferum)

Kelly, E.M. 8 (umbelliferum)

Kendrick, L. 30, 231, 346 (dulcamara)

Kenney, P. 506 (umbelliferum)

Kenoyer, L.A. A291, A292 (pubigerum); 1104 (triquetrum); 2032 (dulcamaroides)

Kerber, E. 68 (dulcamaroides); 257 (pubigerum)

Kerenga, K. 77239 (seaforthianum)

Kernan, C. 159 (dichroandrum)

Kerner, A. 3290 (dulcamara)

Kerr, A.F.G. 220 (dulcamara); 2092 (seaforthianum)

Kerr, F.H.W. 2343 (seaforthianum)

Kessler, M. 3235, 9340 (aligerum)

Khan, ? 796 (dulcamara)

Kibuwa, S.P. 1200 (seaforthianum)

Kiesling, R. 3096 (salicifolium); 3321 (endoadenium); 4110, 4112, 4199 (salicifolium); 6455 (amygdalifolium); 7929 (salicifolium); 8332, 8425 (aligerum)

Kildale, D.K. 625, 7119, 8308 (umbelliferum)

Killip, E.P. 14344 (seaforthianum); 15952 (luculentum); 17267, 17313, 17327, 17388, 17910, 17916 (dichroandrum); 17969 (stenophyllum); 20747 (seaforthianum); 22002 (nitidum); 27539, 29164 (uncinellum); 36240 (umbelliferum); 37028 (seaforthianum)

Kimber, C. 46 (umbelliferum)

Kindale, D.K. 4943 (umbelliferum)

King, Captain P. 25 (valdiviense)

King, D.O. 151 (salicifolium); 407 (crispum); 631 (angustifidum)

Kingdon-Ward, F. 18004 (pittosporifolium)

Kirkbride Jr, J.H. 62, 2519 (seaforthianum); 5243 (viscosissimum)

Kirtland, R. 14754 (umbelliferum)

Klein, R.M. 540 (inodorum); 766 (odoriferum); 1731 (flaccidum); 3290, 4209, 4373, 4378 (laxum); 7516, 7525 (flaccidum); 8013, 8662 (odoriferum); 8719 (flaccidum)

Klementz, E. 99 (dulcamara)

Klingen, A.I. 1304121 (triquetrum)

Klug, G. 484, 664, 1818, 2071, 2320 (uncinellum)

Knapp, S. 825 (storkii); 1930 (seaforthianum); 9813 (dulcamara); 9833 (amygdalifolium); 9947, 9971, 10053 (storkii); IM-10083, IM-10088, IM-10091 (laxum); IM-10093 (inodorum); IM-10101, IM-10102 (dulcamara); 10136, 10138 (pittosporifolium); 10139 (lyratum); 10140 (pittosporifolium); 10141, 10142, IM-10144 (lyratum); 10147 (dulcamaroides); 10436 (aligerum); 10492 (salicifolium)

Kneitel, J. 17 (umbelliferum)

Knight, D. 23 (umbelliferum)

Knopf, E.C. 77, 112, 257 (wallacei)

Knorring, O. 604 (dulcamara)

Ko, S.P. 55334 (lyratum)

Kobayashi, S. 2590, 2700, 2802 (pittosporifolium); 2841 (seaforthianum)

Koch, S.D. 784, 8290 (pubigerum)

Koelz, W. 11428, 12171, 13089, 16569, 16790 (dulcamara); 31248 (laxum)

Kolenati, F.A.R. 101, 718, 1753, 2145 (dulcamara)

Komarov, V.L. 569 (dulcamara)

Konta, F. 23381-a, 35512 (lyratum)

Kossinsky, C. 110 (lyratum)

Kostermans, A.J.G.H. 25435 (seaforthianum)

Kotschy, K.G.T. 775, 87270 (dulcamara)

Kovnat, G. 15 (umbelliferum)

Kozlov, I.V. 232 (septemlobum); 483 (dulcamara)

Krapovickas, A. 3067 (laxum); 5097 (salicifolium); 5268 (endoadenium); 5503, 6209, 14575 (salicifolium); 22056 (endoadenium); 26583 (calileguae); 26585 (aligerum); 26741, 26860 (amygdalifolium); 27062, 27684 (angustifidum); 27773 (amygdalifolium); 29707, 33702 (laxum)

Kravchenko, S. 347 (dulcamara)

Kress, W.J. 94-4338, 94-4440, 94-4548 (storkii); 94-4668 (aligerum); 94-4779, 94-4964 (storkii)

Krishnappa, D.G. 161 (laxum); 368 (seaforthianum)

Krotkov, P. 7758 (dulcamara)

Kruckenberg, A.R. 1917, 3324 (umbelliferum)

Krug, B. 239, 663 (lyratum)

Krukoff, B.A. 1895, 4535, 6751 (uncinellum)

Ku Yam, F. 162 (seaforthianum)

Kuchar, P. 7797 (seaforthianum)

Kueppers, L. 179 (uncinellum)

Kuhlmann, J.G. 2504 (inodorum); SP10694 (seaforthianum); SP304533 (laxum)

Kuhlmann, M. 54 (uncinellum)

Kukkonen, I. 9106 (dulcamara)

Kummrow, R. 1619 (odoriferum); 2796 (inodorum)

Kuniyoshi, Y.S. 3309 (laxum)

Kuntay, S.F. 8735 (dulcamara)

Kuntze, O. 2300 (aligerum)

Kuroiwa, N. 2141 (aligerum)

Kurtz, F. 959, 964 (angustifidum); 1604 (amygdalifolium); 3437, 3463, 7019, 8324, 8786, 9322, 9389 (salicifolium); 10542 (endoadenium); 10887, 10900 (salicifolium); 11857.5, 11857bis (angustifidum); 13366, 13427 (salicifolium); 14056 (endoadenium); 14183, 14518 (salicifolium); 14542, 14975 (endoadenium); 15008, 15062 (salicifolium); 15381 (endoadenium); 15421 (salicifolium); 16296, 16688 (amygdalifolium)

Kusnetzov, I.V. 713 (dulcamara)

Kuvaev, V. 954, 2400-1 (dulcamara)

Kuzmanov, B. 801405 (dulcamara)

Kwangtung 78 5908 (lyratum)

Lachica, F. 4 (pubigerum)

Laegaard, S. 55710 (stenophyllum)

Lagiglia, H.A. 3097 (salicifolium)

Lambinon, J. 87/383 (seaforthianum)

Lamont, J. 1084 (lyratum)

Lampinen, R. 14764 (dulcamara)

Lamy, P. 266 (pubigerum)

Landrum, L.R. 7601, 7991 (valdiviense); 9415A, 9436, 9436 (umbelliferum)

Lange, R. 6972 (flaccidum)

Langlassé, E. 507 (dulcamaroides)

Langman, I.K. 2816, 3728 (pubigerum)

Lansing Jr, O.E. 2781 (dulcamara)

Lape, F. 1 (dulcamaroides)

Larsen, K. 106, 6847 (dulcamara)

Lathrop, E.W. 5595, 5624, 6046, 6435, 6913, 7031 (umbelliferum)

Lau, S.K. 635, 2492 (lyratum); 2738 (pittosporifolium); 4279, 4379 (lyratum)

Lau, S.Y. 20766 (lyratum)

Lauderman, C. 227 (crispum)

Laughlin, R.M. 88 (pubigerum); 125 (aligerum); 1285 (dulcamaroides)

Lawrance, A.E. 393, 400 (uncinellum)

LeSueur, H. 370, 427 (triquetrum)

Leatherman, S. 25 (umbelliferum)

Leavenworth, W.C. 351, 698 (pubigerum)

Lechler, W. 539 (valdiviense); 827 (crispum); 2685 (macbridei)

Lee, E. 1830, 1847, 2076 (umbelliferum)

Lee, H.C. 418, 787, 974, 1048 (umbelliferum)

Legname, P.R. 142 (salicifolium); 8623C (aligerum)

Legrand, C.D. 331 (laxum)

Lehenbauer, P.A. 37 (umbelliferum)

Lehmann, F.C. 163, K222 (aureum); 231 (seaforthianum); 364a (aureum); 1062, 1012 (seaforthianum); 2318 (uncinellum); 4592 (nitidum); 4711 (seaforthianum); 4948 (aureum); 7279 (uncinellum); 8686 (stenophyllum)

Leisner, R. 11909 (seaforthianum)

Leitao Filho, H.F. 2543, 3228 (flaccidum); 17694 (inodorum)

Leite, J.E. 13 (inodorum)

Leiva G., S. 1081 (nitidum); 1485 (cutervanum)

Lellinger, D.B. 1152 (storkii)

Lemmon, J.G. 328 (umbelliferum)

Lent, R.W. 728 (storkii)

León, B. 5200 (cutervanum); 5562, 5581 (leiophyllum)

León, Brother 12543 (boldoense)

León, J. 2682 (pubigerum)

León, R. 272 (angustifidum)

Leonard, E.C. 9844 (seaforthianum)

Leskinen, P. 1002, 1108, 1202 (umbelliferum)

Leu, W.P. 1961 (seaforthianum)

Leuenberger, B.E. 4865 (endoadenium); 4932c (aligerum)

Léveillé, A.A.H. 110347 (pittosporifolium)

Lewis, A. 23, 67, 266 (umbelliferum)

Lewis, G.P. 2726 (leiophyllum); 3719 (coalitum)

Lewis, M. 38467, 39145, 39251 (aligerum)

Lhotsky, J. 87 (flaccidum)

Li Ming 43 (lyratum)

Li Xuegen 200363 (lyratum)

Li, C. 96-218 (pittosporifolium)

Li, Cehong 96079 (pittosporifolium)

Li, F. 930033b (septemlobum)

Li, G. 13078, 13347, 14226, 14773 (lyratum); 15191 (septemlobum); 15968, 16399 (lyratum)

Li, H.G. 3 (septemlobum)

Li, M. 489 (lyratum)

Li, S. 77322, 78542 (lyratum)

Lian, Y. 96964 (pittosporifolium)

Liberi, J. 22 (umbelliferum)

Licent, E. 359, 686, 1979 (septemlobum); 2286, 2532 (lyratum); 6045, 6865, 8611 (septemlobum)

Liebmann, F.M. 1388 (pubigerum); 1436 (aligerum)

Liebsch, D. HFC-6597 (viscosissimum)

Liede, S. 3003 (amygdalifolium)

Liesner, R.L. 3496 (luculentum); 111909 (seaforthianum)

Lillo, M. 2030 (angustifidum); 2615, 2671 (aligerum)

Lima, J. 752 (uncinellum)

Lin Pi 6434, 6549 (lyratum)

Linares, J.L. 1783 (seaforthianum)

Lindeman, J.C. 23, 528 (uncinellum); 2837 (laxum)

Linden, J.J. 433 (dichroandrum); 957 (stenophyllum)

Linder, D.H. 1880 (seaforthianum)

Lindheimer, F.J. 481-III, 481-IV, 525, 1044, 1045 (triquetrum)

Lindsay, N. 1024 (dulcamara)

Ling, K. 12166 (lyratum)

Liogier, A.H. 16758, 17152 (pyrifolium); 20749 (seaforthianum); 21981, 22338 (pyrifolium); 23941, 25455, 26442, 26487 (seaforthianum); 26973 (pyrifolium)

Lipskii, V.I. 1807 (dulcamara)

Little Jr, E.L. 6022, 9875 (aligerum)

Litvinov, D. 481 (dulcamara)

Liu Jin-Kui 667, 719 (lyratum)

Liu Miao H30193, H40091 (pittosporifolium)

Liu, L. 9582, 16107 (lyratum); 16141 (pittosporifolium)

Liu Shen-E 2087a (septemlobum)

Liu, X. 24502, 28926 (lyratum)

Liu, X.T. 90-103 (pittosporifolium)

Liu, Y. 00373, 01965, 02655 (lyratum); 10798, 10889, 11112, 12777, 12800, 13286 (septemlobum)

Liu, Y.S. 1664 (pittosporifolium)

Livers, L. 94 (umbelliferum)

Lizama M., J. 1572 (aligerum)

Llamas, A. 517 (laxum)

Llatas Quiroz, S. 3415 (stenophyllum); 3735 (nitidum)

Lloyd, R.M. 2082, 2157, 3568 (umbelliferum)

Lloyd, R.N. 1076 (seaforthianum)

Lock, J.M. 69139 (seaforthianum)

Loefgren, A. 719 (inodorum); 1441 (flaccidum); 1685 (odoriferum); 2462 (inodorum); 2828 (flaccidum)

Lonaczewsky, A. 7272a (dulcamara)

Long, S. 1090 (dulcamara)

Longtan Team 00812 (lyratum)

Lonvalrée, A. 6781 (dulcamara)

Loos, A. 3663 (salicifolium)

López, A. 1398A (nitidum)

López, F. 340 (dulcamaroides); ISF00388 (seaforthianum)

López, G. 8, 845 (dulcamara)

López, J. 14 (pubigerum)

López F., A.R. 733 (dulcamaroides)

López G., J. 433 (aligerum)

López G., R. 275, 707 (pubigerum)

López García, J.L. 57, 113 (aligerum); 223 (pubigerum); 392 (aligerum)

López Gómez, R. 707 (pubigerum)

López M., A. 1011, 8926 (sanchez-vegae); 8926, 8926 (aureum)

López Santos, B.Y. 440 (pubigerum)

López Vargas, C. 363 (dulcamaroides)

Lorence, D.H. 3742 (dulcamaroides); 4074, 4112 (pubigerum); 4238, 4754 (aligerum)

Lorentz, P.G. 81 (amygdalifolium); 85, 188, 325 (laxum); 418 (salicifolium); 648, 868 (aligerum); 1174, 1260, 1382 (angustifidum); 1676 (laxum); 1728 (amygdalifolium)

Lourteig, A. 716 (salicifolium); 2286 (flaccidum)

Low, A. 284 (dulcamara)

Low, M. 20 (umbelliferum)

Lowrie, S.R. 477 (uncinellum)

Luckow, M. 3757 (seaforthianum)

Ludlow, F. 8101 (dulcamara)

Lugo S, H. 2679, 4573, 4640 (uncinellum)

Lujan, M.C. 11 (salicifolium)

Luna, A. 50 (nitidum)

Luna, F.E. 97 (endoadenium); 551 (amygdalifolium)

Luna, F.S. 1354 (amygdalifolium)

Lundell, C.L. 5730, 9948 (triquetrum); 12385 (pubigerum)

Lundh, R.W. 83, 142, 261 (umbelliferum)

Luteyn, J.L. 11666 (pubigerum); 12828 (stenophyllum); 13794 (aligerum)

Lyon, ? 7b (wallacei)

Lyonnet, E. 1126, 1369, 1369, 1626, 2506, 3050, 3051 (pubigerum)

Maas, P.J.M. 5534 (uncinellum)

MacDaniels, L.H. 525, 527 (pubigerum); 933 (seaforthianum)

MacDougal, J.M. 4251 (aspersum)

MacDougall, T. H71 (pubigerum); 449s (dulcamaroides)

MacElwee, A. 1448 (dulcamara)

MacFadden, F.A. 58E, 2642, 13834 (umbelliferum)

MacFarlane, L. 5 (umbelliferum)

MacGregor, R. 21 (umbelliferum)

Macbride, J.F. 60, 730 (dulcamara); 771 (umbelliferum); 846 (wallacei); 1670, 2011, 2515 (nitidum); 4351, 4441 (ruizii)

Macedo, A. 2767 (viscosissimum)

Macoun, J. 1180 (dulcamara)

Madison, M.T. 963 (uncinellum); 1038 (macbridei)

Madrigal Sánchez, X. 992, 4344 (pubigerum)

Madsen, J. 8040, 8293B, 8323, 8502, 8503, 8606 (stenophyllum); 75641, 86333 (coalitum)

Madsen, J.E. 85561 (cutervanum)

Maekawa, F. 261 (pittosporifolium); 329 (lyratum)

Maesen, L.J.G. van der 2794 (seaforthianum)

Magaña, R.H. 5259, 6182, 6530, 6659, 7059 (pubigerum)

Maguire, B. 11333, 11856 (umbelliferum); 34446 (uncinellum)

Maguire, D.T. 96 (dulcamara)

Maingay, A.C. 430 (lyratum)

Maire, E.E. 13, 89 (lyratum)

Makino, T. 75314 (dulcamara)

Malaise, G.R. 534 (seaforthianum)

Malaisse, F. 8837 (seaforthianum)

Malato-Beliz, J. 726 (laxum)

Maldonado Vasquez, N. 0080 (pubigerum)

Mallory, R.M. 569 (umbelliferum)

Malme, G.A. 225, 534b (laxum); 918 (amygdalifolium)

Mandon, G. 414 (nitidum); 415 (aligerum); 1499 (macbridei)

Manheim, ? 71 (nitidum)

Manissadjian, A. 345 (dulcamara)

Manning, M.H. 88 (umbelliferum)

Manolovitz, M. 21 (umbelliferum)

Manuel, E.A. 1151 (seaforthianum)

Manzano Sosa, A. 4 (pubigerum)

Manzano, G. 177 (aligerum)

Marcelo Peña, J.L. 1920 (aligerum); 3688 (cutervanum)

Marie-Victorin, Frère 33896 (dulcamara)

Marques, A. 2087 (dulcamara)

Márquez Ramírez, W. 953 (laxum)

Marsh, E.G. 1032A, 1032, 1653, 1657 (triquetrum)

Marticorena, C. 1527, 1610 (valdiviense)

Martin, G.J. 380 (pubigerum); 531 (aligerum); 671 (pubigerum)

Martin, R.T. 64 (stenophyllum)

Martius, C.F.P. 255 (amygdalifolium); 821 (viscosissimum); 1071 (flaccidum); 2746 (uncinellum)

Martín, C.J. 227, 421 (dulcamara)

Martín, J. 531 (aligerum)

Martínez, A.M. 1847 (seaforthianum)

Martínez Calderón, G. 333, 784 (dulcamaroides); 2112 (dulcamaroides)

Martínez Crovetto, R. 4850 (angustifidum)

Martínez Feria, A. 78 (dulcamaroides)

Martínez M., E. 3331 (pubigerum)

Martínez Marino, T. 44, 50 (dulcamaroides)

Martínez S., E. 63 (pubigerum); 272, 386 (aligerum); 521, 3287, 3834 (pubigerum); 4741, 5747 (aligerum); 14166 (dulcamaroides); 20814, 20814 (pubigerum); 28444 (aligerum); 31083 (seaforthianum)

Maruñak, V. 137 (laxum)

Masamune, G. 1630 (pittosporifolium)

Mason, H.L. 382, 453, 500, 1066, 3181, 3269, 4485, 10986, 11111, 11985, 12276, 14049 (umbelliferum)

Massey, J. 1122 (umbelliferum)

Mathenge, S.G. 601 (seaforthianum)

Mathews, A. 663 (nitidum)

Mathias, M.E. 1312 (umbelliferum)

Matsushita, H. 125, 179 (lyratum)

Matthes, B. 333 (triquetrum)

Matthew, K.M. 23911, RHT-28530 (seaforthianum); 42889 (laxum); RHT-48498 (seaforthianum)

Matuda, E. S94, 1113, 1707, 2837 (pubigerum); 4812 (dulcamaroides); 16239 (pubigerum); 17531 (seaforthianum); 17760, 18684, 19067, 19513 (pubigerum); 20864 (dulcamaroides); 21033, 25741, 28231 (pubigerum); 28800 (dulcamaroides); 30357 (pubigerum)

Matveeva, E. 116, 791 (dulcamara)

Matzenbacher, N.I. 61 (laxum)

Maury, P. 5616 (pubigerum)

Maximowicz, C.J. 2838 (dulcamara)

Maya J., S. 3602, 3938 (sousae)

Mazzeo, P.M. 1090 (dulcamara)

McClintock, E. 3 (umbelliferum)

McDowell, T. 3338, 3391 (uncinellum)

McGregor, E.A. 79, 1089 (umbelliferum)

McGregor, R.L. 725 (pubigerum)

McKee, H.S. 2006, 10261 (seaforthianum)

McKeever, H.G. 28 (umbelliferum)

McKelvey, S.D. 2151 (umbelliferum)

McMillan, E. 37-54, 130 (umbelliferum)

McMinn, H.E. 2, 195, 236, 1087, 2742, 3854, 3886 (umbelliferum)

McMurphy, J. 72 (umbelliferum)

McNeal, D. 332 (dulcamara)

McVaugh, R. 8293 (triquetrum); 9917 (aligerum); 10066, 11632, 13403, 13403, 13923 (pubigerum); 20200 (seaforthianum)

Mearns, E.A. 3257, 3549 (umbelliferum)

Medina A, M. 290 (seaforthianum)

Medina C, M. 307 (pubigerum)

Medina G, C. 594 (dulcamaroides)

Meebold, A. 27175 (triquetrum)

Meffert, V.V. 314 (dulcamara)

Mejía, M. 931, 7600, 8187 (pyrifolium)

Melderis, A. 163 (dulcamara)

Melhus, I.E. 3575 (pubigerum)

Meling, A. 27 (umbelliferum)

Mélinon, E.M. 316 (uncinellum)

Mello Barreto, H.L. 7627, 7825, 9549 (viscosissimum)

Ména V., P. 80 (stenophyllum)

Méndez G., A. 7615 (aligerum); 7619, 7920, 7922, 7922bis, 8593 (pubigerum)

Méndez Ton, A. 9743 (aligerum); 9780 (pubigerum)

Mendoza G, A. 1287 (aligerum)

Menitskii, Y.L. 38, 86, 122, 70282 (dulcamara)

Mereira Filho, H. 255 (laxum)

Mereles, F. 1193, 1311 (amygdalifolium); 3938, 7043 (salicifolium); 7990 (amygdalifolium)

Mérida, G. 40 (aligerum)

Meriläinen, J. 126 (seaforthianum)

Mertz, S.M. 191 (dulcamaroides)

Mesny, W. 10511 (septemlobum)

Metcalf, R.D. 30729, 30729 (aligerum)

Mexia, Y. 2366, 2367, 2376 (umbelliferum); 4333 (angustifidum); 4559a, 4660, 4962 (flaccidum); 6040 (uncinellum)

Meyer, C. 280, 437 (umbelliferum)

Meyer, C.A. 107, 984 (dulcamara)

Meyer, F.G. 1359, 1368 (umbelliferum); 1650 (dulcamara)

Meyer, F.N. 392, 1009 (septemlobum)

Meyer, T. 830 (uncinellum); 3067 (angustifidum); 3654 (amygdalifolium); 5475, 6805 (laxum); 13518, 13578, 13602 (angustifidum); 15287 (salicifolium)

Michener, E. 191 (umbelliferum)

Michener, D.C. 4283 (umbelliferum)

Mick, G. 204 (pubigerum); 344 (dulcamaroides)

Mickelson, A.I. 487 (dulcamara)

Miers, J. 230 (crispum); 644 (salicifolium); 1429 (amygdalifolium); 3465 (flaccidum); 3885 (amygdalifolium); 4536 (odoriferum)

Mille, L. 971 (seaforthianum)

Miller 1504, 1855 (pubigerum)

Miller, J.S. 487, 487, 692 (pubigerum); 2626 (aligerum); 2851, 2970 (pubigerum); 7413, 7974 (umbelliferum)

Miller, O.O. 104, 104 (seaforthianum)

Milliken, W. M-400 (uncinellum)

Mills, E. 43 (umbelliferum)

Millspaugh, C.F. 858 (seaforthianum); 4487, 4489 (umbelliferum); 4522 (wallacei)

Miossi, B.E. 172 (umbelliferum)

Miranda, F. 97 (pubigerum); 507 (dulcamaroides); 2393 (seaforthianum)

Mish, C.A. 3 (umbelliferum)

Mistretta, O. 439, 477, 563, 1145, 1346, 1432 (umbelliferum)

Miyazaki, T. 05-8182, 03-10277 (pittosporifolium)

Mizoguchi, K. 985 (inodorum); 1048 (flaccidum)

Mizushima, M. 417 (dulcamara); 2443 (lyratum); 12414 (pittosporifolium); 12628 (lyratum)

Mocquerys, A. 792 (seaforthianum)

Moe, L.M. 2326 (umbelliferum)

Moldenke, A.L. 30510 (umbelliferum)

Molfino, J.F. 25707 (amygdalifolium)

Molina R., A. 12618, 13744 (aligerum); 16088, 16088, 16275, 16362 (pubigerum); 16503 (muenscheri); 16706, 16706 (aligerum); 17845 (storkii); 17897 (aligerum); 21215 (muenscheri); 21420 (pubigerum); 21472 (aligerum); 25054 (muenscheri); 25055, 25055, 26390 (pubigerum); 26410 (muenscheri); 26702, 30184, 30186 (pubigerum); 30376 (dulcamaroides); 30200 (aligerum); 31107, 35157 (seaforthianum)

Moloney, A. 115 (seaforthianum)

Monro, A.K. 3952 (nitidum); 4150, 4154 (storkii)

Monteagudo, A. 3986, 15671 (uncinellum)

Montero, G. 33A (crispum); 5203 (valdiviense)

Monterrosa, J. 215 (aligerum)

Montes, J.E. 1545, 3299 (laxum)

Montesinos T., D. 558, 567, 2670 (nitidum)

Montgomery, F.H. 338 (dulcamara)

Montoya, D. 75 (seaforthianum)

Moore, H.E. 1505, 2630 (pubigerum); 2672 (aligerum); 4940 (dulcamaroides)

Moore, H.O. 929 (dulcamara)

Mora Benítez, A. 827 (dulcamaroides)

Morales Ruano, J. 1266 (pubigerum)

Morales, J.F. 168 (aligerum); 1891 (storkii)

Moran, R. 687 (wallacei); 745, 777, 3054, 3346, 7837, 9840, 10545, 10550, 10888, 13203, 13237, 13876, 14077, 16230, 17508, 17620, 19569, 20774, 22336, 24256, 26414 (umbelliferum)

Morefield, J.D. 2672 (umbelliferum)

Moreira, E.A. 36 (flaccidum)

Morel, I. 6155 (amygdalifolium)

Moreno G, S. 264 (pubigerum)

Moreno, P.P. 797, 8103, 9460 (seaforthianum)

Mori, S.A. 11311 (viscosissimum); 17473, 20468, 21971 (uncinellum)

Moring, P. 1208 (dulcamara)

Moritz, J.W.K. 179, 406 (seaforthianum)

Morley, T. 450 (umbelliferum)

Moro-Olivo, A. 5058 (seaforthianum)

Morong, T. 34 (laxum); 818, 1531 (amygdalifolium)

Morrison, J.L. 16760 (crispum)

Morrone, O. 1108 (laxum)

Morton Middleton, R. 738 (dulcamara)

Moscone, E. 92 (salicifolium)

Mosen, H. 983 (flaccidum)

Mossman, E.L. 56 (umbelliferum)

Mostacero L, J. 1326, 2619 (sanchez-vegae)

Mueller, C.H. 8833 (triquetrum)

Mueller, F. 311 (flaccidum)

Mueller-Dombois, D. 68102303, 67121087 (seaforthianum)

Muenscher, W.C. 8394 (dulcamara); 12360 (muenscheri); 14443 (triquetrum); 15033 (umbelliferum)

Müller, F.M. 2454 (umbelliferum)

Muller, F. 1675 (pubigerum)

Müller, G. 3688 (nitidum)

Muller, K.K. 1019 (umbelliferum)

Mulliken, E. 1, 51 (umbelliferum)

Mullins, B. 74 (nitidum)

Munn-Estrada, X. 291, 1474 (aligerum)

Muñoz, F. 512 (dulcamara)

Munz, P.A. 2062, 2135, 2650, 3250, 3272, 3756, 3980, 4002, 4003, 4601, 5053, 5085, 5718, 6866, 7109, 8092, 8453, 9169, 9187, 9374, 9561, 9995, 10129, 10316, 10582, 10851, 11413, 11473, 11667, 11854, 11957, 12030, 12799, 13225, 13241, 13251, 13359, 14836, 14960, 15749, 16106, 16291, 18008, 19449, 20626, 21311, 21346, 22300, 22600 (umbelliferum)

Murakami, H. 134 (lyratum)

Murata, B. 71 (dulcamara)

Murata, G. 1291 (lyratum); 67293 (pittosporifolium)

Murphy, H. 610 (seaforthianum)

Mutchnick, P. 857 (uncinellum)

Mutis, C. 1030, 2004 (seaforthianum)

Mwachala, G. EW-31, EW-117, EW-1203 (seaforthianum)

Mwangangi, O.M. 2474 (seaforthianum)

Mwangoka, M.A. 1299, 1347 (seaforthianum)

Myers, J.G. 5905 (uncinellum)

Myrick, D. 53 (umbelliferum)

Naito, T. 72912 (lyratum)

Narave F., H. 658 (aligerum); 1048 (pubigerum)

Narváez, M. 936, 1186 (seaforthianum)

Nash, G.V. 259 (dulcamara)

Navarro, C. 4348 (dulcamara)

Navarro, G. 294 (amygdalifolium)

Nealley, G.C. 102 (triquetrum)

Nee, M. 3542 (storkii); 4283 (seaforthianum); 17113 (seaforthianum); 19434, 20070, 22211 (triquetrum); 23133 (pubigerum); 23735 (dulcamaroides); 23837 (dulcamaroides); 24056 (triquetrum); 25175 (laxum); 26785 (laxum); 26870, 26894 (aligerum); 27037 (pubigerum); 27696 (seaforthianum); 30182 (macbridei); 30307 (uncinellum); 32319 (pubigerum); 32456 (luculentum); 33119 (pubigerum); 33320 (nitidum); 34036, 34041, 34075, 34093, 34093 (aligerum); 34455, 36071 (uncinellum); 36219, 36607, 36672, 36718, 37563 (aligerum); 40147 (uncinellum); 44757 (aligerum); 47364 (pubigerum); 50008 (aligerum); 50756 (calileguae); 50757 (aligerum); 50768, 50809 (calileguae); 50815 (aligerum); 50937, 50993 (dulcamara); 51736 (aligerum); 51755 (nitidum); 51758 (macbridei); 51818 (nitidum); 51822, 51833, 51841 (aligerum); 54448 (dulcamara); 56757 (endoadenium)

Neill, D.A. 2119 (seaforthianum); 6075, 8063, 9111, 10189 (uncinellum); 12176 (stenophyllum)

Nekrasova, L. 572 (dulcamara)

Nelson, A. 1952 (umbelliferum)

Nelson, B. 31 (umbelliferum)

Nelson, C. 317 (seaforthianum)

Nelson, E. 12512 (endoadenium)

Nelson, E.H. 60a (umbelliferum)

Nelson, E.W. 1348, 1908 (pubigerum); 3031 (dulcamaroides); 3900 (triquetrum)

Nelson, J.C. 1530 (umbelliferum); 1684 (dulcamara)

Nesom, G. 7 (triquetrum); 7866, 7866 (pubigerum)

Nevers, G. de 4971 (uncinellum)

Neves, M. 137 (laxum)

Nevling, I. 1813, 2170 (pubigerum)

Newlon, L.M. 148, 735 (umbelliferum)

Niao, F.H. 9475 (lyratum)

Nichols, D.G. 31 (umbelliferum)

Nicolas, F. 862, 5559 (pubigerum)

Nicora, E.G. 3294 (laxum); 4330 (salicifolium)

Niederlein, G. 274a (uncinellum)

Nielsen, ? 11614 (laxum)

Nishida, M. 12060 (lyratum)

Njeri, V. 9 (seaforthianum)

Nordstrom, G.T. 394, 643, 699, 758, 1064 (umbelliferum)

Norris, R.A. 4093 (umbelliferum)

Norton, H.E. 29 (umbelliferum)

Novara, L.J. 443 (amygdalifolium); 7100, 8170 (aligerum); 9053 (amygdalifolium); 9107 (aligerum); 9873 (amygdalifolium); 12197 (angustifidum)

Novopokrovskii, I. 346, 951 (dulcamara)

Noyes, R. 564 (umbelliferum)

Nuttall, L.W. 76, 704, 1134 (wallacei)

Núñez V., P. 1835 (uncinellum); 6665 (aligerum); 7918 (nitidum); 8994 (aligerum); 8999 (macbridei); 9252 (nitidum); 24183 (uncinellum)

O’Donell, C.A. 313 (salicifolium); 690, 721 (angustifidum); 805 (salicifolium); 884 (angustifidum); 1015, 1016 (salicifolium); 18158 (aligerum)

Ocanto, N. 9 (salicifolium)

Ochoa, C. 78, 328 (nitidum); 12024 (aligerum)

Oersted, A.S. 1408 (aligerum)

Oettingen, H. von 752, 949, 950 (dulcamara)

Oettinger, F.W. 971, 1033, 1347 (umbelliferum)

Ohashi, H. 27 (lyratum)

Ohwe, J. 9071 (lyratum)

Oldham, R. 163, 246, 340, 574 (lyratum); 1039 (pittosporifolium)

Olea, D. 26 (aligerum)

Oliveira, E. 4398 (uncinellum)

Oliveira, P.I. 548 (inodorum)

Oliver, R.L. 978 (pubigerum)

Øllgaard, B. 8567 (imbaburense)

Ollingworth, J.T. 67 (umbelliferum)

Orcutt, C.R. 4136, 4138 (pubigerum)

Orozco, J.M. 304 (pubigerum)

Ortega O., D. 1391 (seaforthianum)

Ortega O., R.V. 726, 1391 (dulcamaroides)

Ortega, R. 231, 321, 321 (dulcamaroides)

Ortiz Adrián, C. 19 (nitidum)

Orzell, S.L. 10186 (triquetrum)

Osten, C. 8265 (laxum); 13448 (salicifolium)

Oswald, V.H. 6226, 6292, 6606, 8417, 8746, 9458 (umbelliferum)

Ottley, A.M. 924 (umbelliferum)

Pabot, H. 2446 (dulcamara)

Pabst, G. 6320 (laxum); 6754, 7146 (flaccidum)

Paddock, E.F. 417 (umbelliferum)

Pai, Y.Y. 115 (septemlobum)

Painter, W.H. 1134 (dulcamara)

Paiva, M.R.C. FUEL-22980 (viscosissimum)

Palacios, W. 00026 (uncinellum); 6906, 11547 (aureum); 11873 (stenophyllum)

Palibin, I.V. 728 (dulcamara)

Palma G, J. 22 (aligerum)

Palmer, E. 62 (umbelliferum); 74 (seaforthianum); 195 (pubigerum); 206, 260, 427, 428, 429 (umbelliferum); 476 (triquetrum); 607 (umbelliferum); 638 (pubigerum); 704 (umbelliferum); 929, 930, 6703, 10356, 13590 (triquetrum); 22347 (dulcamara); 26758 (triquetrum); 28335 (dulcamara); 29476 (triquetrum); 38360 (seaforthianum); 40391 (dulcamara)

Palmer, K. 32 (umbelliferum)

Panigatti, A. 483 (amygdalifolium)

Panti Madero, M.A. 155, 476 (pubigerum)

Parish, S.B. 322, 1888, 3382, 4384, 4388, 4437, 4692, 4758, 5193, 7111, 7899, 8003, 10597, 11404, 11405, 11759 (umbelliferum)

Parker, D. 451 (umbelliferum)

Parks, H.E. 342 (umbelliferum)

Parodi, L.R. 8832 (angustifidum)

Parratt, M. 179, 205 (umbelliferum)

Parry, C.C. 285 (umbelliferum); 633.5 (triquetrum); 638 (pubigerum); 1012 (triquetrum)

Partch, M. 336 (umbelliferum)

Pascual, J. 16, 630 (dulcamaroides)

Pastore, A. 326 (amygdalifolium)

Pawek, J.L. 113, 189, 241 (umbelliferum)

Pearce, R. 142 (valdiviense); 156 (ruizii)

Pedersen, H.B. 42, 95 (stenophyllum)

Pedersen, T.M. 1231 (amygdalifolium); 1834 (angustifidum); 3716 (amygdalifolium); 3916, 4785, 7150 (angustifidum); 9255 (laxum); 10075 (amygdalifolium); 11442 (laxum); 11548 (uncinellum); 13742 (viscosissimum); 13917, 13971 (amygdalifolium); 15243 (angustifidum)

Pedrol, J. 2137 (dulcamara)

Peebles, R.H. 4186 (umbelliferum)

Peguero, B. 725, 733 (pyrifolium)

Peirson, F.W. 185, 186, 1080, 1082, 2792, 3380, 4062, 4486, 4487, 4831, 5154, 5634, 5654, 5737, 6720, 7954, 8321, 8598, 10369, 11048, 12386, 13550 (umbelliferum)

Peña-Chocarro, M.C. 1940, 2404 (amygdalifolium)

Peñafiel, M. 313, 313, 826 (aureum)

Peñalosa, J. 2678 (umbelliferum)

Pendleton, R.L. 43 (umbelliferum); 1366 (wallacei)

Peng, C.I. 17285 (lyratum)

Peng, Q. 0960236 (lyratum)

Peng, T.C. 188 (lyratum)

Penland, C.W. 761 (aureum); 914, 914 (stenophyllum)

Pennell, F.W. 3082 (stenophyllum); 5716 (uncinellum); 13709 (nitidum); 14148 (aligerum); 14213 (nitidum); 17839 (pubigerum)

Pennington, M.S. 128 (amygdalifolium)

Penseiro, J. 1510 (amygdalifolium); 3262 (angustifidum)

Perdue, R.E. 2016 (dulcamara)

Perea, J. 702 (cutervanum)

Pereira, E. 74 (inodorum); 5058 (uncinellum); 5116 (flaccidum); 7922 (laxum); 8208 (flaccidum)

Perkins, A.E. 3376 (pubigerum)

Perkins, J.R. 129 (dulcamara)

Perkins, R. 42 (umbelliferum)

Persaud, A.C. 305 (seaforthianum)

Persson, V. 4488A (uncinellum)

Pessoa, S. de V.A. 474 (inodorum)

Peters, C. 2107 (umbelliferum)

Petersen, E. 1613 (aligerum)

Peterson, W.A. 165, 214, 672, 803 (umbelliferum)

Petrak, F. 862 (dulcamara)

Philbrick, R.N. B65-132, B65-752 (umbelliferum)

Philcox, D. 4468 (seaforthianum)

Philippi, R.A. 46a (crispum); 107, 195 (valdiviense); 482 (crispum); 539 (valdiviense); 680 (crispum); 928 (alphonsei)

Phillips, A. 3068 (umbelliferum)

Piehl, M.A. 62201a (umbelliferum)

Pierotti, S.A. 4201, 4271 (angustifidum); 6558 (laxum)

Pierre, L.L.J. 334 (seaforthianum)

Pietrellini, F. 52, 56 (nitidum)

Pignal, M. 1074 (seaforthianum)

Pimentel, J. 777 (pyrifolium)

Piper, C.V. 6101 (umbelliferum)

Pires, J.M. 4435, 14846 (uncinellum)

Pitman, N. 872 (uncinellum)

Pittier, H. 805 (storkii); 1049 (seaforthianum); 2275 (aligerum); 5861, 7169 (seaforthianum); 7538 (storkii); 9097, 9871 (seaforthianum); 12159, 13917, 15376 (uncinellum)

Pittillo, D. 3102 (dulcamara)

Pitzer, B. 669, 696, 941, 1523, 1943 (umbelliferum)

Plaskett, R.A. 20 (umbelliferum)

Plowman, T. 2422 (uncinellum); 2640 (crispum); 2656 (valdiviense); 2683 (crispum); 6399 (uncinellum); 11074 (nitidum); 11718 (uncinellum)

Pobedimova, E. 97, 351, 383, 1347 (dulcamara)

Podlech, D. 11899, 32535 (dulcamara)

Poeppig, E.F. 63 (valdiviense); 74(diar. 34), 680 (crispum); 714 (valdiviense)

Pohl, J.B.E. 5414 (odoriferum)

Pojarkova, T.O. 156 (dulcamara)

Poliquesi, C.B. 597 (inodorum)

Polunin, O. 56/776 (pittosporifolium)

Pons Sorolla, A. 616 (dulcamara)

Poole, J.M. 1621 (uncinellum)

Poritskii, A. 505, 902 (dulcamara)

Porter, M. 12, 14 (umbelliferum)

Post, B.V.D. 277 (dulcamara)

Prado, D. 625 (amygdalifolium)

Praed, B. 5332 (flaccidum)

Prance, G.T. P25324 (uncinellum)

Prather, A. 1356 (umbelliferum)

Prendusi, T. 55 (umbelliferum)

Preston, T.A. 1015 (dulcamara)

Price, W.R. 816 (lyratum)

Priest, J. 263 (stenophyllum)

Prieto, P. P-309 (leiophyllum)

Prigge, B.A. 4244, 7473 (umbelliferum)

Pringle, C.G. 153 (triquetrum); 4188 (dulcamaroides); 4680 (pubigerum); 6901 (laxum); 7496 (dulcamaroides); 8835 (aligerum); 9489 (pubigerum); 11062 (triquetrum); 13134 (pubigerum)

Prirevtishii, N.M. 119 (dulcamara)

Proctor, G.R. 818 (umbelliferum); 18152, 19055, 24153 (seaforthianum); 32074 (storkii)

Proctor, M.D. ROB0190, ROB0493 (umbelliferum)

Propst, D. 14 (wallacei)

Provance, M. 615, 1756 (umbelliferum)

Prozorovskii, A.V. 187 (dulcamara)

Pruett, H. 16 (umbelliferum)

Prévost, M.F. 2137 (uncinellum)

Puch, A. 17, 17 (seaforthianum)

Puga, L.M.V. de 6739 (dulcamaroides); 9631 (seaforthianum)

Puggiari, J.I. 3141 (viscosissimum)

Purer, E.A. 6544, 6717, 6739, 15000, 15148 (umbelliferum)

Purpus, C.A. 162 (pubigerum); 1046 (umbelliferum); 1285 (dulcamaroides); 1447, 1731 (umbelliferum); 1734 (pubigerum); 1735 (pubigerum); 1793, 1820, 1906 (umbelliferum); 3010 (pubigerum); 3363 (dulcamaroides); 3836 (pubigerum); 3969 (dulcamaroides); 5053 (umbelliferum); 5836 (pubigerum); 7326 (pubigerum)

Pérez, E. 94 (nitidum); 1344 (dulcamaroides); 3609 (pubigerum)

Pérez, S. 59, 60 (seaforthianum)

Pérez C., E. 2570 (pubigerum)

Pérez G., I. 432, 993 (pubigerum)

Pérez Portilla, E. B9 (aligerum)

Pételot, A. 2604 (pittosporifolium); 8464 (dulcamara)

Qin, H. 2394 (lyratum)

Qingzhang Team 425 (lyratum); 2586 (septemlobum); 2865 (lyratum); 5189 (septemlobum)

Querie, R.W. 81 (laxum)

Quibell, C.H. 702, 816, 993, 1725, 1951, 2070 (umbelliferum)

Quick, A. 1166, 1204 (umbelliferum)

Quick, C.R. 40-46, 52-07, 58-32, 62-8, 52-217, 1928 (umbelliferum)

Quintero G., G. 5 (pubigerum)

Quipuscoa S., V. 187 (sanchez-vegae)

Ragonese, A.E. 2680 (angustifidum)

Rainha, B. 377 (dulcamara)

Raizada, M.B. 20669 (seaforthianum)

Rakvidhyasatra, V. 18 (umbelliferum)

Rambo, B. 2236 (inodorum); 31238 (viscosissimum); 40159 (amygdalifolium); 44283, 44854, 45669 (laxum); 51595 (viscosissimum); 51858 (flaccidum); 51897 (viscosissimum); 53995 (flaccidum); 54621, 56264 (inodorum); 57199 (seaforthianum); 57339 (laxum); 58557 (flaccidum)

Ramos, M. 173 (pubigerum)

Ramos, P.X. 142 (dulcamaroides)

Ramsey, H.J. 203, 206, 526, 568, 658, 671, 684, 813, 963, 968, 1450, 1732, 1740, 1963, 2030, 2433 (umbelliferum)

Ramsay, P.M. 230 (stenophyllum)

Ramírez, D.M. 9 (umbelliferum)

Ramírez, E. 821 (aligerum)

Ramírez, V. 39 (storkii)

Ramírez G., E. 61 (pubigerum + aligerum, mixed collection); 502 (pubigerum); 502, 502 (aligerum)

Ramírez R., C. 677 (seaforthianum)

Randall, A.D. 55, 258 (umbelliferum)

Randall, J.D. 405 (umbelliferum)

Randolph, D. 235 (pubigerum); 351 (dulcamaroides)

Randolph, L.F. 12864 (dulcamara)

Rasp, A.E. 65 (valdiviense)

Ratter, J.A. R3430, R3867 (viscosissimum)

Raup, H.M. 7502 (dulcamara)

Raus, T. 6964 (dulcamara)

Raven, P.H. 1797, 2087, 2162, 2557, 2862, 2994, 3223, 4159, 4244, 6739, 8980, 12221, 12313, 12692, 12943, 13801, 14188, 14928, 15254, 19003 (umbelliferum); 19978, 19978 (aligerum)

Rawi, A. 12159, 23865, 26568 (dulcamara)

Ray, G. 194 (dulcamara)

Raynal, J. 16057 (seaforthianum)

Read, B.E. R1190 (pittosporifolium)

Reales, A. 1035, 1931 (endoadenium)

Rebman, J.P. 8736, 9571, 10076, 11111 (umbelliferum)

Rechinger, K.H. 733, 11027, 41735 (dulcamara); 64092, 64135, 64160, 64219 (valdiviense); 64317 (crispum)

Reddick, D. 4 (pubigerum); 179 (laxum); 280, 294, 626 (pubigerum)

Reed, E.C. 1869 (crispum)

Reed, F.M. 2819 (wallacei)

Regnell, A.F. II-221 (inodorum); I-354, II-1712 (flaccidum); 3029 (odoriferum)

Reid, B. 2 (umbelliferum)

Reineck, E.M. 11, 89 (laxum); 379 (amygdalifolium)

Reitz, P.R. 1168, 2571 (flaccidum); 2922 (odoriferum); 3162 (flaccidum); 3549, 3550 (inodorum); 3614 (laxum); 4082 (flaccidum); 5484a (laxum); 6343 (odoriferum); 7040 (viscosissimum); 7304 (flaccidum); 7420 (laxum); 7525 (viscosissimum); 7767, 7859 (laxum); 8510 (viscosissimum); 8514 (flaccidum); 8920 (inodorum); 9157, 10719 (flaccidum); 13224 (inodorum); 13352, 13626, 14339 (laxum)

Renderos, M.A. 517 (seaforthianum)

Renvoize, S.A. 2911 (laxum); 3421 (endoadenium)

Reveal, J.L. 430 (umbelliferum); 4230 (pubigerum)

Reverchon, J. 671 (triquetrum)

Reyes S., J. 1381 (pubigerum)

Reyes, J.R. 3050 (aligerum)

Reynel, C. 2125 (nitidum)

Rhode, R.M. 159 (umbelliferum)

Ribas, O.S. 1154 (viscosissimum)

Ricards, P.D. 9 (umbelliferum)

Rich, H.H. 930 (laxum)

Ricker, P.L. 4092 (umbelliferum)

Ricketson, J. 2503 (umbelliferum)

Ridley, H.N. 68, 69 (seaforthianum)

Riedel, L. 407 (amygdalifolium)

Riera, J. 19505 (dulcamara)

Riina, R. 867 (dichroandrum)

Rilke, S. 455 (nitidum)

Rimachi Y., M. 3615, 8110 (uncinellum)

Ripley, S. 458 (seaforthianum)

Ritter, L.V. 243 (umbelliferum)

Ritter, N. 919, 2699 (aligerum)

Rivera Reyes, J. 2814, 2910, 3050, 3063 (aligerum)

Rivera, J. 5516 (dulcamara)

Rivera, L. 42 (pubigerum)

Rivet, M. 415 (aureum)

Roadhouse, J.E. 167 (umbelliferum)

Robbins, G.T. 1017 (umbelliferum)

Roberts, ? da 171 (umbelliferum)

Roberts, F.M. 4342, 4622 (umbelliferum)

Robertson, I.J.B. D.11 (dulcamara)

Robertson, R. 70 (umbelliferum)

Robinson, E. 13, 25 (umbelliferum)

Robinson, F. 1208, 1209 (dulcamara)

Robson, J.E. 738 (dulcamara)

Robson, N.K.B. 349, 350 (dulcamara)

Rocabado, D. 148 (aligerum)

Roche, D. 17 (umbelliferum)

Rock, J.F. 4311 (lyratum); 14273 (septemlobum)

Rodin, B. 32 (umbelliferum)

Rodin, R.J. 2585 (seaforthianum)

Rodrigo, A.P. 2405 (angustifidum)

Rodríguez B., D. 102 (pubigerum)

Rodríguez C., A. 591 (dulcamaroides); 939 (aligerum); 1006, 1630 (pubigerum)

Rodríguez, ? 179b (amygdalifolium)

Rodríguez, A. 2013 (pubigerum); 2677 (dulcamaroides); 2704 (aligerum)

Rodríguez, D. 5032 (pubigerum)

Rodríguez, L. 3428 (seaforthianum)

Rodríguez, R. 191 (seaforthianum)

Roe, K. 2191 (pubigerum)

Roerich Expedition 325, 326, 385, 402, 766, 779 (septemlobum)

Rogers, F.A. 538 (dulcamara)

Roig, F.A. 3664 (endoadenium)

Rojas, ? 1624 (amygdalifolium)

Rojas, R. 681, 0980, 1795, 6179 (uncinellum)

Rojas, T. 193 (salicifolium); 1392 (laxum); 2437 (uncinellum); 8563 (amygdalifolium); 10475 (laxum)

Rolland, F. 15680 (dulcamara)

Rollins, R.C. 2944, 3001 (umbelliferum)

Romoleroux, K. 687 (stenophyllum)

Roos, J.C. 3646 (umbelliferum)

Roque, J. 885, 3099, 3247, 3316, 5461 (nitidum)

Rosas R., M. 806 (aligerum)

Rose, J.N. 8305, 8879, 9628 (pubigerum); 9846 (dulcamaroides); 22200 (laxum); 22963 (aureum); 24143, 24215 (triquetrum)

Rose, L.S. 3101, 34024, 36061, 36254, 39016, 40353, 57071 (umbelliferum)

Roseberry, R.D. 130 (umbelliferum)

Ross, T.S. 2467, 2673, 2934, 3169, 3499, 3670, 3728, 3985, 4218, 4606, 4920, 5774, 6049, 6250, 6637, 6704 (umbelliferum); 6937a (wallacei); 7280, 7321, 7352, 7552, 7718, 8129, 8370, 8420, 8534 (umbelliferum)

Rossato, M. 4586, 4586 (laxum)

Rossi, C.J. 22 (umbelliferum)

Rothrock, J.T. 131 (umbelliferum)

Rotman, A. 930 (aligerum)

Rotman, A.D. 373 (salicifolium)

Rousseau, J. 27213, 35346, 35472 (dulcamara)

Rowlee, H.E. 926 (pubigerum)

Roybal, J.J. 748 (triquetrum)

Rübtzoff, P. 1195, 3058, 4358 (umbelliferum)

Rudder, J. 3608 (seaforthianum)

Rueda, R. 16497 (dulcamaroides)

Ruffo, C.K. 547, 547 (seaforthianum)

Rugel, F. 145 (boldoense)

Ruiz, H. 869 (nitidum)

Ruiz de Huidrobo, H. 69 (angustifidum)

Ruiz T., A. 291 (pubigerum)

Rusby, H.H. 781 (aligerum); 797 (nitidum); 839, 2475 (uncinellum)

Russell, J. 66 (umbelliferum)

Russell, P.G. 192 (pubigerum); 218 (dulcamaroides)

Rutherford, W. 1607 (umbelliferum)

Rutter, C. 104 (umbelliferum)

Rutter, J.A. 164 (umbelliferum)

Ruygt, J. 860, 872, 963, 3203 (umbelliferum)

Rydzewski, D. 37 (umbelliferum)

Rzedowski, J. 961 (pubigerum); 18351 (dulcamaroides); 19381, 19408 (pubigerum); 27036 (aligerum); 30761 (pubigerum); 46192 (aligerum); 48405 (pubigerum); 48594, 49012, 49037, 50418 (dulcamaroides); 52348 (pubigerum)

Safford, W.E. 1253 (triquetrum)

Sagot, P.A. 459 (uncinellum)

Sagástegui, A. 3878 (aligerum); 8251 (cutervanum); 8804, 9994, 11894 (sanchez-vegae); 12314 (nitidum); 16535, 16631 (sanchez-vegae)

Saint-Hilaire, A. de 1052 (flaccidum)

Saito, S. 3289, 4374 (pittosporifolium)

Salas, G. 377 (pubigerum)

Salas M., S.H. 4001, 4158 (dulcamaroides)

Salas Zuloaga, E. 90 (nitidum)

Saldanha, C.J. 8777, 13521, 14649 (seaforthianum)

Saldías, M. 635, 4607 (aligerum)

Salinas, N. 3275 (aligerum)

Sallee, E.H. 22, 47, ES80 (pubigerum)

Salvato, T.B. 630 (umbelliferum)

Samokish, N.I. 1373, 1965 (dulcamara)

Samuels, E. 191 (umbelliferum)

Samuels, J.A. 63 (uncinellum)

Samuelsson, V. 1371 (dulcamara)

Sánchez Mejorada R., H. 307 (aligerum)

Sánchez Vega, I. 3142 (sanchez-vegae); 8401, 9983 (uncinellum); 10957 (stenophyllum)

Sandberg, J.T. 173, 184 (septemlobum)

Sandeman, C. 37 (aureum); 356 (valdiviense); 4262 (aureum); 4834 (amygdalifolium)

Sanders, A.C. 11609 (dulcamaroides); 12181, 18135, 19749, 22757, 22774, 23429, 24997, 25837, 25981, 26316, 26640, 27774 (umbelliferum)

Sandoval Bassó, A.J. 8 (pubigerum)

Sandoval, A. 61 (pubigerum)

Sandwith, N.Y. 1674 (seaforthianum)

Saneshka, V. 66 (dulcamara)

Sangai, E. 1 (seaforthianum)

Sangkatavat, W. 225 (umbelliferum)

Santesson, R. 1119 (valdiviense); 13962 (dulcamara)

Santiago, C. 49 (aligerum)

Santillán, R. 400 (seaforthianum)

Santos Martínez, J. 1579, 2150 (dulcamaroides)

Santos, T.S. dos 1801, 2041 (uncinellum)

Santíz Ruiz, C. 7, 86, 591, 938 (pubigerum)

Sanzin, R. 35 (laxum)

Sargent, F.H. 218 (seaforthianum)

Sartori, J. 84 (dulcamara)

Sarukhan, J. 3615 (pubigerum)

Sastre, C. 4306, 8297 (seaforthianum)

Sato, I. 72811 (lyratum)

Saunders, S.G.E. 380, 490 (nitidum); 1173 (ruizii)

Savatier, P.A.L. 878 (lyratum)

Saverkin, A. 413 (dulcamara)

Say, A. 2506 (salicifolium)

Sayago, M. 1252, 1503 (salicifolium); 1944 (endoadenium)

Schaffner, J.G. 2, 547, 558, 690 (pubigerum)

Scheepers, J.C. 265 (seaforthianum)

Schiavone, M.M. 11901C. (aligerum)

Schickendantz, F. 30 (endoadenium); 45, 91 (salicifolium); 120 (endoadenium); 214 (salicifolium); 221, 285 (endoadenium); 329 (salicifolium)

Schiede, C.J.W. 950 (pubigerum)

Schiffner, V. 2506 (pittosporifolium)

Schimpff, H.J.F. 810, 896 (aureum)

Schindler, A.K. 165 (lyratum); 168, 670 (septemlobum)

Schinini, A. 4030 (amygdalifolium); 5069 (seaforthianum); 6053 (laxum); 10117, 10119 (aligerum); 13864 (angustifidum); 17440, 25460 (laxum); 25512, 27250 (seaforthianum); 27997 (laxum); 29206 (angustifidum); 31413 (laxum); 33035 (uncinellum)

Schlieben, H.J. 4246 (seaforthianum)

Schlim, L.J. 831 [also sometimes as Linden 831] (dichroandrum)

Schlobohm, D.F. 106 (umbelliferum)

Schmidt, H.H. 2891 (dulcamara)

Schmitz 159 (pubigerum)

Schmutz, P.E. 2221 (seaforthianum)

Schneider, C.K. 20 (pubigerum); 104 (muenscheri); 710 (dulcamara); 2113, 2705 (lyratum)

Schomburgk, R. 123(66), 594 (uncinellum)

Schoolcraft, G. 1539, 1540, 1542, 1543, 1898 (umbelliferum)

Schott, A.C.V. 5414 (odoriferum)

Schreiber, B.O. 753, 2584 (umbelliferum)

Schreiter, R. 4826, 5778 (endoadenium); 6183 (salicifolium); 6470, 6552, 8812, 9809 (aligerum); 10469 (endoadenium); 10595 (salicifolium)

Schroeter, C. 164 (amygdalifolium)

Schuette, J.H. 96 (dulcamara)

Schuler, A. 98/447 (dulcamara)

Schultes, R.E. 3173, 7082 (laxum); 7536 (aureum); 20151 (stenophyllum)

Schultze-Jena, L. 138 (dulcamara)

Schulz, A.G. 2066, 3906 (angustifidum); 7251 (amygdalifolium)

Schunke Vigo, J. 357, 865, 6015, 6617, 15118, 15403, 15474 (uncinellum)

Schunke, C. 135, 136 (uncinellum)

Schury, R.W. 616 (flaccidum)

Schwacke, A. 1880 (laxum); 4328 (odoriferum); 6943, 25695 (viscosissimum)

Schwarz, G.J. 1739, 6445 (laxum); 9313, 9702 (angustifidum); 10698 (laxum)

Schwindt, E. 1499 (amygdalifolium); 4294 (laxum)

Scolnik, R. 21Tu160 (aligerum)

Scora, R.W. 2289 (triquetrum)

Scott, P.R. 471 (dulcamara)

Scott, R.W. 588 (dulcamara)

Scur, L. 190 (laxum)

Segura Zubizarreta, A. 3862 (dulcamara)

Sehnem, A. 2196 (flaccidum); 3843, 4959 (laxum); 5148 (inodorum); 5757 (viscosissimum); 8353 (inodorum)

Seidel, R. 3205 (uncinellum)

Seigler, D. 13787 (pubigerum)

Seijo, J.G. 2541 (amygdalifolium)

Seler, C. 1070 (triquetrum)

Sellow, F. 15, 44 (laxum); 94 (inodorum); 128 (flaccidum); 130, 132 (laxum); 464, 745 (flaccidum)

Semper, J. 269, 397 (salicifolium)

Semsei, S.R. 3487 (seaforthianum)

Sennen, E.C. 4895 (laxum)

Sereno A., O. 23 (pubigerum)

Sessé, M. 1418 (dulcamaroides); 1503 (boldoense); 1506 (pubigerum); 1509 (triquetrum); 1524, 5358 (pubigerum); 5363, 5381 (dulcamaroides); 5381bis (boldoense); 5385 (triquetrum)

Seto, K. 30076 (pittosporifolium)

Sexauer, F. 79 (umbelliferum)

Seçmen, ? 5767 (dulcamara)

Shafer, J.A. 82 (seaforthianum)

Shanteau, W.E. 21 (umbelliferum)

Shanxi Team 1627, 1627 (septemlobum)

Sharp, A.J. 4412 (pubigerum); 441218 (aligerum)

Sharsmith, C.W. 3570 (dulcamara)

Sharsmith, H.K. 605, 1500, 1551, 3410, 5214 (umbelliferum)

Shaughnessy, M. 7 (umbelliferum)

Shestunov, N. 53, 101 (dulcamara)

Shetler, S.G. 266 (dulcamara)

Shevock, J. 20, 115, 263, 585, 605 (umbelliferum)

Shi Shigui S-0950 (lyratum)

Shilom Ton, A. 454, 462, 578, 874, 1376 (aligerum)

Shiota, K. IYO90 (pittosporifolium); 4000, 4004 (lyratum); 4805, 5417 (pittosporifolium); 6875 (lyratum); 7242 (pittosporifolium); 7299, 7856 (lyratum); 9294 (pittosporifolium)

Shipchinskii, N. 275 (dulcamara)

Shishkin, I.K. 274, 1306 (dulcamara)

Shkhagapsoev, S. 675-2000 (dulcamara)

Short, L.R. S27, 120, 179 (umbelliferum)

Shoshin, A.A. 594 (dulcamara)

Shreve, F. 6831 (umbelliferum); 9633 (triquetrum)

Sidorova, T.V. 155 (dulcamara)

Sieverling, H. 113 (umbelliferum)

Silva Saenz, P. 406 (dulcamaroides)

Silva, D.C. 85 (flaccidum)

Silva, J.M. 399 (flaccidum); 451 (odoriferum)

Silva, J.S. 297 (inodorum)

Silva, M.G. 3348 (uncinellum); 3464, 3848 (uncinellum)

Silva, P.C. 2611 (umbelliferum)

Simmonds, N.W. 14479 (seaforthianum)

Simontacchi, A. 52, 253, 265, 385, 672 (umbelliferum)

Simpson, N.D. 9162 (seaforthianum); 21078, 21516, 23345, 34161, 35620, 52108 (dulcamara)

Sinaca Colin, S. 1016 (dulcamaroides)

Sindel, G.E. 39, 166 (umbelliferum)

Singh, U. 413 (seaforthianum)

Sinnott, Q.P. 266 (umbelliferum)

Sino-American Expedition 1278 (pittosporifolium); 1576 (lyratum)

Sino-American Guizhou Bot. Exp. 78 (lyratum); 90 (pittosporifolium); 2159 (lyratum); 2225 (pittosporifolium)

Sino-British Plant Exped. Team 0013 (pittosporifolium)

Sintenis, P. 637, 2035, 3370, 4266 (dulcamara); 6292, 6343 (seaforthianum)

Siñani, R. 230 (aligerum)

Skoss, J.D. 28 (umbelliferum)

Skottsberg, C. 398 (valdiviense)

Skutch, A.F. 507 (pubigerum); 738 (aligerum); 815, 1167 (pubigerum); 5183, 5183 (storkii)

Slane, V. 827 (seaforthianum)

Sleumer, H. 8 (salicifolium); 18 (aligerum); 505 (salicifolium); 1623 (endoadenium); 1633, 1634 (salicifolium); 1835, 2134 (salicifolium); 2166 (salicifolium); 2208, 2375, 2399 (salicifolium); 2402 (endoadenium); 2481, 2483, 2484, 2487, 2492, 2495 (salicifolium); 3063, 3846 (aligerum)

Smick, G. GAS068 (umbelliferum)

Smith, C.E. 4500 (aligerum)

Smith, C.F. 529, 930, 2889, 4031, 6457, 15214 (umbelliferum)

Smith, C.L. 697, 698, 1788 (pubigerum)

Smith, C.N. 785 (umbelliferum)

Smith, D.N. 1622, 2182 (nitidum); 7524 (sanchez-vegae); 8726, 11083, 11390, 11623, 12466 (nitidum)

Smith, E.M. 1446A (umbelliferum)

Smith, G.L. 1176, 3005, 3058, 3059, 3098, 3283, 3315, 6194, 7133, 8041 (umbelliferum)

Smith, H.H. 1178 (seaforthianum); 1243 (septemlobum); 2265 (lyratum); 2661 (uncinellum); 3959 (lyratum); 4093 (dulcamara); 5045 (wallacei); 5580 (septemlobum); 6432 (lyratum); 6456, 6456, 7514 (septemlobum)

Smith, J.D. 6671 (seaforthianum)

Smith, J.P. 8742, 9371 (umbelliferum)

Smith, L.B. 7341 (viscosissimum); 7724, 7796, 8244, 8244, 8954, 10791, 10960, 11135, 12469, 12754 (laxum); 13922 (flaccidum); 14004 (viscosissimum); 14027 (laxum)

Smith, L.S. 1571 (umbelliferum)

Smith, R. 202 (dulcamara)

Smith, S.D. 380 (salicifolium)

Smith, W.R. T-1197 (triquetrum)

Sneidern, K. von 5006 (uncinellum); 5825 (aspersum)

Soares, Z. 175 (amygdalifolium)

Sobral, M. 2418, 3032, 3115 (laxum); 3211 (inodorum); 4206 (laxum)

Sodiro, A. 114/17 (aureum); 166 (laxum)

Soejarto, D.D. 3965 (uncinellum)

Soihet M., G. 142 (aligerum)

Solano, S.C. 305, 305 (dulcamaroides); 436 (pubigerum)

Solbrig, O.T. 4518 (pubigerum)

Solheim, S.L. 1400 (aligerum)

Solís Neffa, V. 59 (amygdalifolium)

Solomon, J.C. 2705 (triquetrum); 2978 (nitidum); 4478 (valdiviense); 5365, 8431, 10249 (aligerum); 11660 (macbridei); 14386 (aligerum); 19144 (seaforthianum); 30307 (uncinellum)

Sombral, M. 3032 (laxum)

Soper, J.H. 2011 (dulcamara)

Sota, A.V. de la 1546, 1559 (salicifolium)

Soto N., J.C. 1708, 1748 (dulcamaroides); 3827, 4251, 5250 (pubigerum); 5747 (aligerum); 7134, 7164 (pubigerum); 7175 (aligerum); 7684, 7934, 6955, 7684, 8299 (pubigerum); 8345, 8519 (aligerum); 8521, 8654, 9981 (pubigerum); 11628 (seaforthianum); 12611 (pubigerum); 12704 (aligerum); 12795, 12795, 12988 (pubigerum)

Soto N., S. 4426 (seaforthianum)

Soukup, J. 360, 2772 (nitidum)

Soulié, J.-A. 753 (pittosporifolium); 872 (septemlobum)

Soulié, R.P. 1476 (lyratum)

Sousa Peña, M. 577 (aligerum)

Sousa, M. 25 (pubigerum); 1093 (dulcamaroides)

South Team T0120, T0180 (lyratum); T0265 (septemlobum); T0265 (pittosporifolium); D1046 (septemlobum); Da1429 (lyratum)

Souviron, M.J. 81 (aligerum)

Sowder, J.E. 158, 186, 371 (umbelliferum)

Sowerby, B. 1, 4 (dulcamara)

Soza, V.L. 494, 579, 1656 (umbelliferum)

Sparre, B. 14 (laxum); 399, 2628 (crispum); 3168, 3313, 3499, 3672, 4024, 4397 (valdiviense); 13375 (aureum); 14163 (stenophyllum); 14280, 17727 (aureum)

Spegazzini, C.L. 18083 (laxum)

Spellman, D.L. 653 (aligerum); 980 (pubigerum)

Spencer, M.F. 148, 1069, 1789, 1867, 2276 (umbelliferum)

Sperling, C.R. 5946 (uncinellum)

Sphon, G. 332 (umbelliferum)

Splitgerber, F.L. 330 (uncinellum)

Spruce, R. 1706, 4327 (uncinellum); 5741 (aureum)

St. John, R. 136, 337, 347 (umbelliferum)

Stafford, D. 813 (nitidum); 984 (macbridei)

Stafford, P.J. 248 (pubigerum)

Stamatiadou, E. 13382, 13675, 15477 (dulcamara)

Standley, P.C. 37368 (seaforthianum); 42265, 42764 (aligerum); 43558, 43729 (storkii); 44861 (aligerum); 60004 (pubigerum); 65295, 66039, 66059, 66103, 66262, 66281, 66308, 66367 (pubigerum); 66613 (pubigerum); 67328, 67342, 67562, 67563, 67567, 67770 (pubigerum); 81147, 81724, 81886 (muenscheri); 83317, 83322, 83539, 83821, 83939, 84041, 84273 (pubigerum); 84478 (muenscheri); 85282, 85346, 85758 (pubigerum)

Stanford, E.E. 266, 393, 894, 992, 1220 (umbelliferum)

Stanford, L.R. 141 (triquetrum)

Stannard, B. 515 (uncinellum)

Stark, B.D. 4475 (umbelliferum)

Starseuslli, A.R. 108 (laxum)

Stason, M. 7930, 8941 (umbelliferum)

Stauning, O. 91 (seaforthianum)

Stebbins, G.L. 2570 (umbelliferum)

Steele, E.S. 246 (dulcamara)

Stehlé, H. 4345 (seaforthianum)

Stehmann, J. 307 (laxum)

Steibel, P. 3991 (salicifolium)

Stein, B.A. 2343 (uncinellum)

Steinbach, J. 3826, 5152, 5752 (aligerum); 7588 (uncinellum); 8621, 8685, 9550, 9854, 9870 (aligerum)

Steinbach, R.F. 195, 563 (aligerum)

Steingraeber, ? da 64 (pubigerum)

Steinmann, V.W. 662 (umbelliferum)

Stellfeld, C. 1644 (flaccidum); 1690 (laxum)

Stergios, B. 10995, 12972 (uncinellum)

Stevens, W.D. 2997, 17722 (seaforthianum)

Steward, A.N. 263, 263 (lyratum); 9742 (pittosporifolium)

Stewart, R.M. 813 (triquetrum)

Stewart, R.R. 14000, 18104, 24062 (dulcamara)

Steyermark, J.A. 3409 (aligerum); 33980 (pubigerum); 34672 (aligerum); 35633 (muenscheri); 36196 (pubigerum); 49046, 49870 (aligerum); 49974 (pubigerum); 50333 (muenscheri); 52266 (pubigerum); 57101 (dichroandrum); 62349 (seaforthianum); 87535 (uncinellum); 88841 (seaforthianum); 95331, 99869 (uncinellum); 100777 (luculentum); 102316 (seaforthianum); 112281, 117089, 122314 (uncinellum); 122933 (seaforthianum); 127855 (luculentum)

Stinchfield, R.J. 299, 342 (umbelliferum)

Stoffers, A.L. 3161 (seaforthianum)

Stone, J. 2887, 2910, 2946 (umbelliferum)

Stone, R.D. 615 (umbelliferum)

Stork, H.E. 2068, 2515 (storkii); 2916 (aligerum); 3023, 3347 (storkii); 10241, 10833 (nitidum)

Stormay, A.L. H-117 (umbelliferum)

Stortz, V. 839 (umbelliferum)

Straw, R.M. 1459 (pubigerum); 2314 (nitidum)

Stuckert, T. 1747, 1798, 2207 (salicifolium); 2416 (angustifidum); 3690 (salicifolium); 4021, 4501 (angustifidum); 4555, 5848, 6977, 7019 (salicifolium); 7648 (laxum); 7725 (salicifolium); 8184, 8252, 8326, 8646 (angustifidum); 8654, 8669, 8672, 8770, 9257, 10372, 10698, 10883, 10947 (salicifolium); 11207 (angustifidum); 11592 (laxum); 12199, 12322, 12520 (salicifolium); 16464 (angustifidum); 19589, 20771, 20970, 20976, 21228 (salicifolium); 21835 (angustifidum); 22180, 23259, 23276 (amygdalifolium); 24012 (salicifolium)

Sturrock, ? A39 (seaforthianum)

Subils, R. 2352 (angustifidum); 3548 (endoadenium); 4520 (amygdalifolium); 4662 (angustifidum)

Suclli, E. 1277 (aligerum)

Sueltenfuss, C. 174 (triquetrum)

Suin, L. 1982 (uncinellum)

Sullivan, G. 885 (nitidum)

Summers, R.W. 273 (umbelliferum)

Sumner, E.L. 101 (umbelliferum)

Sun, Y.Z. 206, 217, 727 (lyratum)

Sunckel, H. 1161 (valdiviense)

Susanna, A. 185 (dulcamara)

Suzuki, S. 138 (lyratum)

Swartley, J.C. 66 (dulcamara)

Sweeney, J.R. 866 (umbelliferum)

Swinney, D. 2784 (umbelliferum)

Swinney, R.G. 2336, 3834, 4169, 4776, 4965 (umbelliferum)

Symon, D.E. 4754, 4900 (seaforthianum)

Synnott, T.J. 1286, 1485, 1545 (seaforthianum)

Syphers, G. 40, 41 (umbelliferum)

Taam Ying-Wah 39 (lyratum)

Täfur, V. 267 (laxum)

Tai, H.H. 271 (lyratum)

Tai, T.L. 102263 (lyratum)

Takahashi, H. 1650-A (dulcamara)

Takahashi, M. 1705 (lyratum)

Takahashi, T. 2215, 30261 (lyratum)

Tamayo, F. 2216 (uncinellum)

Tamura, M. 9361 (pittosporifolium); 9362 (lyratum)

Tan, C.M. 94-1079, 94-1179 (pittosporifolium); 94-1258 (lyratum); 96-8072 (pittosporifolium); 96-11003 (lyratum); 11019 (lyratum); 11042 (pittosporifolium)

Tan, P. 57159, 57322, 57478, 59685, 60191, 60451 (lyratum)

Tanaka, T. 325 (dulcamara)

Tang, T. 839 (septemlobum)

Tao Guoda 40024 (pittosporifolium)

Taquet, T. 1146, 1147 (lyratum)

Tate, R. 44.H (pubigerum)

Taylor, C.M. 33 (salicifolium); 10282, 10843 (valdiviense)

Taylor, D.W. 12495, 12812, 16195 (umbelliferum)

Taylor, J. 11724, 11793 (storkii); 15605 (umbelliferum)

Taylor, K. 241 (pubigerum)

Taylor, M.S. 492, 1302, 2090, 3502, 4383, 5413 (umbelliferum)

Tee, T.S. 4 (umbelliferum)

Tejada, N. 14580 (salicifolium)

Téllez V., O. 4698, 7262 (aligerum)

Templeton, B.C. 1045, 4859 (umbelliferum)

Teng, S.W. 90622 (lyratum)

Tengwall, T.A. 48, 690 (dulcamara)

Tenorio L., P. 556 (aligerum); 1557, 3716 (pubigerum); 5362 (aligerum); 6251 (pubigerum); 7502, 8630 (aligerum); 9110 (pubigerum); 10310 (seaforthianum); 11383 (aligerum); 13240, 13264 (umbelliferum); 13764 (pubigerum); 14020 (aligerum); 18325 (pubigerum)

Terborgh, J. 69 (pyrifolium)

Terry, M.E. 1605 (uncinellum)

Tessmann, G. 4586 (uncinellum)

Texeira, B.C. 228 (laxum)

Thacher, O. 46 (umbelliferum)

Thackery, F.A. 281, 463 (umbelliferum)

Tharp, B.C. 1609, 5608 (triquetrum)

Thibault, T. 223, 511 (umbelliferum)

Thomas, J.H. 121, 239, 554, 1154, 1238, 1275, 1342, 1425, 1459, 1542, 1696, 1865, 2475, 2510, 2876, 3228, 3800, 3946, 5700 (umbelliferum)

Thomas, S.M. 383 (nitidum)

Thomas, W. 3756, 3757 (pubigerum)

Thomas, W.D. 127 (umbelliferum)

Thomas, W.W. 3916 (uncinellum)

Thompson, I. 55 (umbelliferum)

Thompson, J.W. 5241 (dulcamara)

Thompson, S.A. 1287 (triquetrum); 7588 (pyrifolium)

Thompson, W. 2 (umbelliferum)

Thorne, R.F. 31524, 31632, 32033, 32161, 32327, 34057, 34415, 34808 (umbelliferum); 34931, 34995 (wallacei); 35383, 35595 (umbelliferum); 35798, 36520 (wallacei); 36937, 37726, 38189, 38820, 39334 (umbelliferum); 39358 (wallacei); 39668, 39977, 40782, 40919, 40965 (umbelliferum); 41570 (wallacei); 41608, 47397, 47527, 51910, 52317, 52500, 53136, 53964, 53988, 54254, 55925, 55953, 57670, 57696, 58617, 60023, 60627, 61592, 62136, 62195, 62268, 62479, 62650 (umbelliferum)

Tidestrom, I. 11542 (dulcamara)

Tiehm, A. 14027 (umbelliferum)

Tilforth, C.W. 1287, 1577, 1613, 1763, 2071 (umbelliferum)

Tillett, S.S. 671-43 (uncinellum); 6611-41 (seaforthianum)

Timaná, M. 2693, 3173, 3659 (uncinellum)

Ting, K.C. 1512 (lyratum)

Tipáz, G. 1813 (uncinellum)

Tirel, C. 33, 225 (salicifolium)

Tisserant, C. 2083 (seaforthianum)

Tlapa A, M. 772, 1331 (pubigerum)

Todzia, C.A. 2815, 2815 (aligerum)

Toledo, C.B. 246 (uncinellum)

Tolstead, W.L. 5833 (triquetrum)

Ton, A.S. 454, 874 (aligerum)

Tonduz, A. 1408 (seaforthianum); 2275 (aligerum); 8024, 9097 (seaforthianum); 11928 (aligerum)

Topping, D.L. 137 (dulcamara)

Toriz A., G. 528 (pubigerum)

Torres, A. 1084 (laxum)

Torres, R. 2959 (pubigerum); 13501 (dulcamaroides)

Torres B., E. 2022 (pubigerum); 2022 (aligerum)

Torres C., R. 277 (aligerum); 2825 (pubigerum); 2916 (aligerum); 2959 (pubigerum); 4594, 7499 (aligerum); 7723 (pubigerum); 7985, 8450 (aligerum); 9690, 11034 (dulcamaroides); 12394 (pubigerum); 16202, 16641 (aligerum)

Torres R., A. 46 (dulcamaroides)

Toscani, H.L. 51 (angustifidum)

Tothill, J.C. 137 (nitidum)

Toumey, J.W. 396, 397 (umbelliferum)

Tovar, O. 140 (seaforthianum); 786, 3011, 3522, 5110 (nitidum)

Townsend, C.H.T. 10 (triquetrum)

Tracy, J.P. 1338, 2601, 2787, 4219, 6515, 6657, 6668, 13240, 14325, 14576, 15132, 15313, 15316, 15340, 15406, 15528, 15529, 15855, 16824, 17399, 18105, 18293, 18698, 18851, 19214, 19471 (umbelliferum)

Train, P. 803 (umbelliferum)

Transhel, V. 284 (dulcamara)

Traver, T. 51 (umbelliferum)

Traverse, A. 1097, 1098, 1221, 2330 (triquetrum)

Trécul, M. 1075, 1269 (triquetrum)

Trejo, M. 45 (pubigerum)

Tressens, S.G. 560 (angustifidum); 4439, 5596, 6465 (laxum)

Triana, J.J. 3855/1 (stenophyllum); 3855/48 (seaforthianum)

Troani, H.O. 540 (salicifolium)

Troncoso, N. 3127 (amygdalifolium)

Trott, A.C. 24 (dulcamara)

True, G.H. 35, 84, 385, 1611, 2822 (umbelliferum)

Trujillo, B. 9374 (seaforthianum)

Tsai, H.T. 53712, 53712 (lyratum)

Tsang, W.T. 21496, 22893, 23260, 24190 (lyratum); 28384, 28400 (pittosporifolium); 28841 (lyratum)

Tschá, M.C. 141, 148 (flaccidum)

Tsiang, Y. 1403 (lyratum)

Tsu, L.F. 703 (lyratum)

Tsugaru, S. 7400 (pittosporifolium); 17048, 20836, 21283, 25940 (lyratum)

Tsuguru, S. 25876, 27273 (lyratum)

Tsvelev, N.N. 914, 3220, 4175 (dulcamara)

Tunqui, S. 521, 871 (uncinellum)

Tupayachi, A. 3424 (aligerum)

Türckheim, H. von 3362 (pyrifolium)

Turkevich, C. 82 (dulcamara)

Tutin, T.G. 1124 (nitidum)

Tweedie, J. 327 (laxum); 358 (amygdalifolium); 422 (laxum)

Twisselmann, E.C. 633, 855, 1069, 1674, 2037, 2100, 2210, 2322, 2432, 2602, 2759, 2786, 2838, 3094, 3423, 3670, 4469, 5328, 6068, 6149, 7838, 8214, 9613, 9619, 9623, 9626, 10954, 11052, 11076, 11077, 11154, 11344, 12132, 12251, 12359, 12888, 13273, 13346, 14134, 14202, 14489, 14585, 15044, 15746, 16382, 17599, 17718 (umbelliferum)

USDA 85 (umbelliferum)

Ugent, D. 2466 (aligerum); 5256 (nitidum)

Ule, E. 4302 (flaccidum); 9759, 9762 (uncinellum)

Ulloa, M. 247 (pubigerum)

Uno, K. 24095 (pittosporifolium)

Uotila, P.J. 20137 (dulcamara)

Usteri, A. 15a (inodorum)

Utgoreev, S. 126 (dulcamara)

Utley, J. 3083 (aligerum)

Valencia, N. 961b (nitidum)

Valenzuela, L. 1131, 1236 (aligerum); 1988 (nitidum); 3163 (kulliwaita); 4978 (cutervanum); 6922 (aligerum)

Valerio, M. 1049 (aligerum)

Valeur, E.J. 681 (seaforthianum)

Valla, J.J. 17665 (uncinellum)

Valorado, J.F. 3855 (dulcamara)

Valverde, F.M. 123 (seaforthianum)

Valverde, O. 907 (storkii)

Van Rensberg, H.J. van 495 (seaforthianum)

Vanni, R. 734, 3731 (laxum)

Varela, F. 519 (angustifidum); 633 (salicifolium)

Vargas, ? 246 (seaforthianum)

Vargas, C. 1361, 1606, 9716, 11178 (macbridei); 13476 (uncinellum)

Vargas, H. 317 (stenophyllum); 3043 (aspersum); 3582 (uncinellum)

Vargas, Y.A. 306, 344, 370, 383 (aligerum)

Vargas C., I.G. 2302, 3002, 5006 (aligerum)

Vargas R., C. 69 (seaforthianum)

Vasconcellos Neto, J. 20401 (inodorum)

Vasey, G. 440, 441 (umbelliferum)

Vasilchenko, I.T. 926 (dulcamara)

Vasileev, V.N. 173 (dulcamara)

Vasquez, A. 4634 (aligerum)

Vásquez, L. 1129 (dulcamaroides)

Vásquez, R. 1273, 1533, 3045, 21893, 23963, 27834 (uncinellum)

Vasquez M., N. 0080 (pubigerum)

Vásquez S., J. 1007 (pubigerum)

Vázquez, A. 4626 (aligerum)

Vázquez, J. 3437 (dulcamaroides)

Vázquez V., L. 1001 (dulcamaroides)

Vaughan, J. 1033, 1056 (pubigerum)

Vela G, L. 977 (aligerum)

Velarde Nuñez, O. 2008 (ruizii)

Velasco Gutiérrez, K. 83, 2837 (dulcamaroides)

Velasco, C. 15 (pubigerum)

Velayos, M. 6797, 7547 (dulcamara); 11136 (aligerum)

Veloz, A. 1265 (seaforthianum); 2511, 3130 (pyrifolium)

Venrick, B. 448, 449 (umbelliferum)

Ventura A., A. 532, 613, 830 (pubigerum); 1022 (sousae); 1321, 2565 (pubigerum); 22129 (sousae)

Ventura A., E. 4 (pubigerum)

Ventura A., F. 957 (pubigerum); 1008, 1008 (aligerum); 2822 (seaforthianum); 3505 (dulcamaroides); 3531, 9810, 11146 (aligerum); 12156 (seaforthianum); 12871, 13915, 17096, 18538, 19577 (aligerum); 21852, 21919 (pubigerum)

Ventura V., E. 300, 729, 760, 2066 (pubigerum)

Ventura, E. 4352, 4352 (aligerum); 4368 (pubigerum); 8994, 8994, 9899 (seaforthianum)

Ventura, J. V94.10 (pubigerum)

Venturi, S. 40 (amygdalifolium); 51 (angustifidum); 177 (laxum); 272 (amygdalifolium); 4067, 4632, 5080, 5080 (aligerum); 5498 (endoadenium); 5588, 5588 (uncinellum); 6671 (endoadenium); 7755 (aligerum); 7777 (salicifolium); 8506 (endoadenium); 9012 (salicifolium); 9609, 9934, 10599 (aligerum); 15597 (angustifidum)

Verdcourt, B. 3026 (laxum); 10839 (seaforthianum)

Verdugo, A. 285, 353 (aureum)

Vibrans, H. 7366 (dulcamaroides)

Vieira, G. 462 (uncinellum)

Viereck, H.W. 860 (triquetrum)

Vilcapoma, G. 119-2, 119 (nitidum); 337 (seaforthianum)

Villa, A. 662 (seaforthianum)

Villa C., J. 505 (pubigerum)

Villacorta, R. 403 (seaforthianum)

Villacres, P. 282 (nitidum); 373 (aureum)

Villafaño, N. 828 (amygdalifolium)

Vine, R.S. 181 (laxum)

Vitantonio, ? 1675 (dulcamara)

Volponi, C.R. 69 (salicifolium)

Voronov, Y. 12134 (dulcamara)

Vorontsova, M.S. 193 (seaforthianum)

Voznesenskij, I.G. 186 (umbelliferum)

Vrugtman, F. 610348 (dulcamara)

Véliz, M. 92.2331, 95-4467, MV 2M11115 (pubigerum)

Waas, S. 770 (seaforthianum)

Wade, A.E. 517/1c (dulcamara)

Wagenbreth, I. 42 (seaforthianum); 642, 792, 901 (pubigerum)

Wagner, H.K. 219 (umbelliferum)

Wagner, R.J. 1126 (seaforthianum)

Wahl, H.A. 2514 (dulcamara)

Wainwright, C.M. 594 (umbelliferum)

Walker, E.H. 5993, 8192 (seaforthianum)

Walker, H.A. 41 (umbelliferum)

Wall, E. 31 (valdiviense)

Wallace, E.C. 1412 (dulcamara)

Wallnöfer, B. 4212 (dulcamara)

Walter, D. 10200 (umbelliferum)

Walter, H. 331 (angustifidum); 2659 (seaforthianum)

Wang Zhong-tao 870423 (lyratum)

Wang, C. 40268 (pittosporifolium); 97004 (lyratum)

Wang, C.W. 40508 (lyratum); 61841 (pittosporifolium); 62342 (septemlobum); 65289 (lyratum); 66159 (pittosporifolium); 67159, 67927 (lyratum); 70253 (pittosporifolium); 70376, 70442, 71448 (lyratum)

Wang, F. G73 (lyratum)

Wang, F.T. 21371, 22655 (lyratum)

Wang, H. 41503c (lyratum)

Wang, Q. 522 (lyratum); 978 (septemlobum)

Wang, S. E8 (lyratum)

Wang, T.P. 3599 (septemlobum)

Wang Zuobin 17568 (septemlobum)

Warming, J.E.B. 166 (laxum)

Warnock, B.H. 148, 326, 14861, 46352 (triquetrum)

Wasshausen, D.C. 1012 (uncinellum)

Wasum, R. 128, 148, 165, 232, 259, 1080, 2081, 3069, 4469, 8648, 10392, 12747 (laxum)

Watanabe, M. 28 (pittosporifolium)

Waterfall, U.T. 4925, 6278, 6330, 13286 (triquetrum); 14061 (pubigerum)

Watkins, ? 681 (pubigerum)

Weatherby, R.J. 491, 667 (umbelliferum)

Webb, R.J. 68 (dulcamara)

Weberbauer, A. 3252 (nitidum); 3310 (cutervanum); 5483 (nitidum); 5643 (aligerum); 6411 (aureum); 6477 (ruizii); 6700 (cutervanum); 7636 (seaforthianum)

Webster, G.L. 66 (umbelliferum); 11513 (pubigerum); 12131, 12309 (storkii); 17280 (pubigerum)

Webster, J. 257 (septemlobum)

Wei Yuzhong 20474 (lyratum)

Weimann, C. CWEI204 (pubigerum)

Weir, J. 78 (flaccidum)

Welch, B. 4613 (dulcamara)

Welch, W.H. 6120 (dulcamara)

Welwitsch, F.M.J. 202, 802 (dulcamara)

Wen, J. 9666 (seaforthianum)

Wendehake, C. 26, 29 (seaforthianum)

Werdermann, E. 323 (valdiviense); 324, 439, 479 (crispum); 2391 (seaforthianum)

Werff, H. van der 3775a, 4252, 12928 (umbelliferum); 20706 (nitidum)

Wessels Boer, J.G. 1009 (uncinellum)

West, J. 4667 (valdiviense); 6410 (nitidum)

West, R.C. H-1, E2 (pubigerum)

Wetherell, V.J. 207 (umbelliferum)

Wetzel, C.L. 030, 107, 253, 504, 678, 1010 (umbelliferum)

Whalen, M.D. 843 (nitidum)

Wheeler, C.R. 1565, 2241, 2403, 3195, 3526 (umbelliferum)

Wheeler, G. 6141 (wallacei)

Wheeler, L.C. 267, 446, 1028, 2853, 4447, 5957, 9718, 9824 (umbelliferum)

White, J. 1609 (dulcamara)

White, S. 611 (dichroandrum)

White, S.D. 92-186, 92-233, 959, 4831, 5217, 5995, 7409, 7431, 7562, 7671, 7693, 7713, 7759, 8326, 8512, 8524, 8657, 8853, 8961, 9088, 9190, 9229, 9453, 9564, 9585, 10048, 10305, 10344, 10353, 10359, 10410, 10856, 11310, 11319, 11543 (umbelliferum)

White, S.S. 1789 (triquetrum)

Whittaker, J. 538 (dulcamara)

Widgren, J.F. 877 (amygdalifolium)

Wiegand, M.C. 4229, 4229 (umbelliferum)

Wieslander, A.E. 298 (umbelliferum)

Wiggins, I.L. 501, 1897, 2019, 2071, 2437, 2476, 3376, 3507, 3541, 3746, 3913, 7550, 8052, 9136, 9216, 9778, 9896, 11847, 12336, 13054, 16074, 21276 (umbelliferum)

Wilbur, R.L. 8772 (storkii)

Wilczek, E. 241 (salicifolium)

Wilder, P. 1505, 1558, 1708, 4435, 4570 (umbelliferum)

Wilkinson, J. 3370 (pubigerum)

Willard, B. 108 (umbelliferum)

Williams, Ll. 3382 (uncinellum); 4323, 7877 (seaforthianum); 9958, 13493 (seaforthianum); 14985 (uncinellum)

Williams, L.O. 6810 (viscosissimum); 12803 (aligerum); 13878, 13878 (storkii); 15356 (pubigerum); 16278 (storkii); 20032 (aligerum); 21999, 21999, 22385, 22385 (pubigerum); 22388 (muenscheri); 22579, 22579, 22841 (pubigerum); 22919 (muenscheri); 22929 (pubigerum); 24390 (storkii); 25497, 25497, 25902, 27007 (pubigerum); 28276, 28862 (storkii); 41447 (pubigerum); 41964 (dulcamaroides)

Williams, M.J. 84-6-2, 85-63-4 (umbelliferum)

Williams, R.S. 825 (aligerum); 2354 (nitidum)

Williams, S.W. 1341 (septemlobum)

Williamson, W.J. 14 (umbelliferum)

Willkomm, H.M. 313 (dulcamara)

Wilmott, A.J. 708, 856, 3520, 430616C (dulcamara)

Wilson, E.H. 1281 (lyratum); 4199 (septemlobum)

Wilson, J. MSc2 (nitidum)

Wilson, R.C. 126 (umbelliferum)

Winter, J.M. 278 (dulcamara)

Wislizenus, F. 1296 (umbelliferum)

Wisura, W. 4156, 4464, 5000 (umbelliferum)

Witte, P.F.M. 14547 (dulcamara)

Woglum, R.S. 398, 1009, 1096, 1433, 1456, 1714, 2413 (umbelliferum)

Wolf, C.B. 13, 201, 219, 652, 1403, 1640, 1704A, 1758, 1850, 1862, 1924, 1950, 1965, 2000, 2040, 2051, 2082, 2143, 2171, 2198, 2311, 2334, 2449, 2775, 2815, 2858, 2919 (umbelliferum); 3491 (wallacei); 3496, 3537, 3552 (umbelliferum); 4215, 4219 (wallacei); 4416, 4444, 4648, 4735, 6885, 7953, 7978, 8706, 8924, 9562 (umbelliferum); 10891 (wallacei); 11125, 11166 (umbelliferum)

Wolff, S.E. 395, 975, 3449, 4189 (triquetrum)

Wood, J.R.I. 8545, 8708 (aligerum); 10688 (salicifolium); 14006 (uncinellum); 14238 (nitidum); 15030 (aligerum); 15824 (angustifidum); 15976 (nitidum); 16350, 16371 (aligerum); 16441 (nitidum); 16448 (amygdalifolium); 18479, 19411 (aligerum); 19422 (nitidum); 19700, 22011 (aligerum)

Woodcock, F.G. 905, 905a, 905b (umbelliferum)

Woolston, A.L. 315 (laxum); 486, 873 (uncinellum); 1056, 1505, 1602 (laxum)

Worth, C.R. 16448 (crispum)

Woytkowski, F. 117 (nitidum); 34550 (uncinellum)

Wright, C. 156, 381 (boldoense); 536, 1591 (triquetrum); 2023, 3023 (boldoense)

Wright, J.T. 23-54 (umbelliferum)

Wright, P.A. 65 (dulcamara)

Wright, W.G. 89 (umbelliferum)

Wu Sugong 8066 (pittosporifolium)

Wu, C. 1306 (lyratum)

Wu, Q. 97-75, 97-73, 98-164 (septemlobum)

Wullschlägel, H.R. 373 (uncinellum)

Wurdack, J.J. 1314 (sanchez-vegae); 1704 (cutervanum)

Wuyi Shan Team 80-0529 (lyratum); 80-0635 (pittosporifolium)

Wydler, H. 67 (seaforthianum)

Wynd, F.L. 34 (triquetrum)

Xantus de Vesey, L.J. 73 (umbelliferum)

Xiao Bai-Zhong 4525, 4803 (lyratum)

Xu, H. 4069, 4153, 4600, 4773, 4938, 5234 (lyratum)

Xu, S. 2799 (pittosporifolium)

Yamashiro, T. 7030, 7041 (pittosporifolium)

Yang, J. 6317 (septemlobum)

Yao, K. 9233 (lyratum)

Yarupaitán, G. 38, 368 (nitidum)

Yates, H.S. 317, 3669, 5324, 5366, 5474, 5526 (umbelliferum)

Yates, L. 80 (umbelliferum)

Ying, J. 0907 (lyratum)

Yonekura, K. 5960 (dulcamara); 95952 (pittosporifolium); 96563 (lyratum)

York, D. 623 (umbelliferum)

Yorks, P.F. 118 (umbelliferum)

Young, D.A. P444, P445 (umbelliferum)

Young, D.P. 75, 4008, 4854 (dulcamara)

Young, K. 1573, 1948, 1987, 2357, 2378 (cutervanum); 2407 (leiophyllum); 2511, 2809 (cutervanum); 3002 (nitidum); 3554 (sanchez-vegae); 3763, 3866, 4475 (cutervanum)

Yu, T.T. 1015, 6347, 10384, 14713, 14941, 22007 (lyratum)

Yuncker, T.G. 5685 (seaforthianum)

Zaharova, ? 40 (dulcamara)

Zak, V. 1457 (aureum); 1563 (stenophyllum); 1635, 1741 (aureum); 1842 (stenophyllum); 1845 (aureum); 1846 (stenophyllum); 1857A (aspersum); 2081 (stenophyllum); 2360, 2393, 2400, 2403, 2416 (aureum); 2466, 2468, 2469 (nitidum); 2969 (stenophyllum); 3353, 3698 (aureum); 5034, 5079 (uncinellum)

Zalensky, O. III81-82 (crispum)

Zamora C., P. 1096 (aligerum)

Zamudio [R.], S. 2510, 2688 (pubigerum); 4162 (dulcamaroides); 4289, 5798 (pubigerum); 4971 (dulcamaroides); 6019, 10795 (pubigerum)

Zamzow, G.W. 36 (umbelliferum)

Zanoni, T. 10811 (seaforthianum); 16604A (pyrifolium); 17351 (seaforthianum); 21410, 21552, 21868 (pyrifolium); 23226 (seaforthianum); 23227, 27520, 30422 (pyrifolium); 30778 (seaforthianum); 35857, 42952 (pyrifolium); 47042 (seaforthianum)

Zapata, S.R. 40 (aligerum)

Zappi, D. 1192 (uncinellum)

Zardini, E.M. 2672 (amygdalifolium); 7036 (laxum); 7789 (uncinellum); 14976 (laxum); 17492 (uncinellum); 20317, 21657, 22295, 36385 (amygdalifolium); 43177 (uncinellum)

Zarucchi, J.L. 4753, 6928 (luculentum)

Zhang, H. 4495 (lyratum)

Zhang, Z. 11000 (lyratum); 11070, 13621 (pittosporifolium); 14506, 14857 (lyratum)

Zhao, Z.E. 5429 (septemlobum)

Zheng, Z. 209 (septemlobum)

Zhirov, I.V. 574 (dulcamara)

Zhou, P. 02801 (lyratum)

Zhou, Z. 060 (pittosporifolium)

Zhu Dahai 2232 (lyratum)

Zhu Qiugui 292 (lyratum)

Zhu, G.H. 2386, 2909, 3166 (lyratum)

Zigmond, M. 605, 708 (umbelliferum)

Zimmerer, K.S. 80 (aligerum)

Zimmerman, E. 15 (umbelliferum)

Zohman, N. 44 (umbelliferum)

Zola B., M.G. 552, 552, 790 (dulcamaroides)

Zöllner, O. 9612 (amygdalifolium)

Zorab, ? 718 (dulcamara)

Zuck, C. 65 (umbelliferum)

Zuloaga, F.O. 4882, 5278 (laxum)

Zúñiga G., G. 128 (pubigerum)

Zygadlo, J.A. 11, 140 (salicifolium).

## Appendix

Specimens examined for *Revision of the Dulcamaroid Clade of Solanum L. (Solanaceae)*. (doi: 10.3897/phytokeys.22.4041.app) File format: Microsoft Excel document (xls).

**Explanation note.** This Microsoft Excel file consists of all collection records (with associated herbaria) of species examined for this taxonomic treatment. Full details of all records can be found on Solanaceae Source (http://www.solanaceaesource.org ). Additional records are added to Solanaceae Source as they are seen so it is the most up to date place to see specimens for this group.

Columns in the spreadsheet contain data as follows. All altitudes are in metres (m) and zeros (0) in fields in the latitude, longitude and elevation columns indicated missing data, not the number 0 (zero).
